# The unfinished agenda of communicable diseases among children and adolescents before the COVID-19 pandemic, 1990–2019: a systematic analysis of the Global Burden of Disease Study 2019

**DOI:** 10.1016/S0140-6736(23)00860-7

**Published:** 2023-07-22

**Authors:** GBD 2019 Child and Adolescent Communicable Disease Collaborators

## Abstract

**Background:**

Communicable disease control has long been a focus of global health policy. There have been substantial reductions in the burden and mortality of communicable diseases among children younger than 5 years, but we know less about this burden in older children and adolescents, and it is unclear whether current programmes and policies remain aligned with targets for intervention. This knowledge is especially important for policy and programmes in the context of the COVID-19 pandemic. We aimed to use the Global Burden of Disease (GBD) Study 2019 to systematically characterise the burden of communicable diseases across childhood and adolescence.

**Methods:**

In this systematic analysis of the GBD study from 1990 to 2019, all communicable diseases and their manifestations as modelled within GBD 2019 were included, categorised as 16 subgroups of common diseases or presentations. Data were reported for absolute count, prevalence, and incidence across measures of cause-specific mortality (deaths and years of life lost), disability (years lived with disability [YLDs]), and disease burden (disability-adjusted life-years [DALYs]) for children and adolescents aged 0–24 years. Data were reported across the Socio-demographic Index (SDI) and across time (1990–2019), and for 204 countries and territories. For HIV, we reported the mortality-to-incidence ratio (MIR) as a measure of health system performance.

**Findings:**

In 2019, there were 3·0 million deaths and 30·0 million years of healthy life lost to disability (as measured by YLDs), corresponding to 288·4 million DALYs from communicable diseases among children and adolescents globally (57·3% of total communicable disease burden across all ages). Over time, there has been a shift in communicable disease burden from young children to older children and adolescents (largely driven by the considerable reductions in children younger than 5 years and slower progress elsewhere), although children younger than 5 years still accounted for most of the communicable disease burden in 2019. Disease burden and mortality were predominantly in low-SDI settings, with high and high-middle SDI settings also having an appreciable burden of communicable disease morbidity (4·0 million YLDs in 2019 alone). Three cause groups (enteric infections, lower-respiratory-tract infections, and malaria) accounted for 59·8% of the global communicable disease burden in children and adolescents, with tuberculosis and HIV both emerging as important causes during adolescence. HIV was the only cause for which disease burden increased over time, particularly in children and adolescents older than 5 years, and especially in females. Excess MIRs for HIV were observed for males aged 15–19 years in low-SDI settings.

**Interpretation:**

Our analysis supports continued policy focus on enteric infections and lower-respiratory-tract infections, with orientation to children younger than 5 years in settings of low socioeconomic development. However, efforts should also be targeted to other conditions, particularly HIV, given its increased burden in older children and adolescents. Older children and adolescents also experience a large burden of communicable disease, further highlighting the need for efforts to extend beyond the first 5 years of life. Our analysis also identified substantial morbidity caused by communicable diseases affecting child and adolescent health across the world.

**Funding:**

The Australian National Health and Medical Research Council Centre for Research Excellence for Driving Investment in Global Adolescent Health and the Bill & Melinda Gates Foundation.

## Introduction

The substantial reductions in burden and mortality from communicable diseases among children younger than 5 years have been one of the success stories of global health.^1^, ^2^ Key initiatives contributing to these gains include the WHO Integrated Management of Childhood Illness, the WHO and UNICEF child survival strategy, and the integrated Global Action Plan for Prevention and Control of Pneumonia and Diarrhoea (GAPPD). The Millennium Development Goals also brought a focus to diarrhoea and pneumonia and vaccine-preventable diseases for young children (goal 4), and HIV, malaria, and other communicable diseases at a population level (goal 6). This agenda was carried through to the Sustainable Development Goals (SDGs), in which specific targets include ending preventable deaths among children younger than 5 years (target 3.2), ending the epidemics of AIDS, tuberculosis, malaria, and neglected tropical diseases, and combating hepatitis, water-borne diseases, and other communicable diseases (target 3.3). Global funding initiatives including The Global Fund to Fight AIDS, Tuberculosis and Malaria (HIV, tuberculosis, and malaria), the US Presidents’ Emergency Plan for AIDS Relief, the Global Alliance for Vaccines and Immunisation, and the Grand Challenges programme through the Bill & Melinda Gates Foundation have helped drive international commitments, global policy, and national action against these targets.^3^

**Research in context**

**Evidence before this study**

The 2016 *Lancet* Commission on Adolescent Health and Wellbeing, a subsequent analysis of 12 headline indicators for adolescent health, and recent analyses of adolescent mortality each identified communicable diseases to be a key contributing cause of mortality and morbidity among adolescents globally. In 2016, 40% of the burden of disease (measured in disability-adjusted life-years [DALYs]) among adolescents was accounted for by communicable, maternal, and nutritional diseases. Yet these analyses report communicable diseases as an aggregate group and do not provide estimates of specific communicable disease burden, essential for targeted policy and programming. We could not find any further analyses of communicable disease burden for adolescents, or indeed for older children. In November, 2021, we searched for reports and publications describing the burden of communicable diseases among children and adolescents aged 0–24 years in the past 10 years (2012–21) using the following search terms: “communicable diseases/epidemiology” AND child* OR adoles* OR youth* OR paed* OR ped*. We also searched for specific causes (including pneumonia, diarrhoea, malaria, HIV, and tuberculosis) supplemented by recent Global Burden of Disease (GBD) publications on pneumonia and diarrhoea in young children. We reviewed peer-reviewed and selected grey literature sources: UN agencies including WHO, UNICEF, and UNAIDS; key policy and monitoring agencies, including the Independent Accountability Panel, The Partnership for Maternal, Newborn and Child Health, and Countdown to 2030; and funding bodies such as The Global Fund to Fight AIDS, Tuberculosis and Malaria and the Bill & Melinda Gates Foundation. We screened more than 6000 titles but found no report or systematic analysis of communicable disease burden across childhood and adolescence. Available evidence either focused on specific age groups (particularly children <5 years of age), specific diseases, or both, or on mortality only. Available summary reports of population health (including the WHO Global Health Observatory and the Institute for Health Metrics and Evaluation GBD capstone papers) often describe communicable disease at an aggregate level, which again is insufficient for targeted policy and actions. Countdown to 2030 and associated country profiles and available data dashboards for child and adolescent health through UNICEF and WHO include indicators of some communicable diseases, but again mostly for young children. Formerly known as the WHO and UNICEF's Child Health Epidemiology Reference Group, the Maternal Child Epidemiology Estimation (MCEE) group published global, national, and regional mortality estimates in 2019 for diarrhoea, malaria, tuberculosis, lower-respiratory-tract infections, and HIV and AIDS for children and adolescents aged 0–19 years in 5-year age groups and disaggregated by sex for those aged 15–19 years. These MCEE estimates make an essential contribution to the literature, but they are focussed on mortality. For tuberculosis, the WHO 2022 Global Tuberculosis Report (and other tuberculosis surveillance data) describes data for children and adolescents aged 0–5 years, 5–14 years, and 15–24 years by WHO region; however, there is no country-level age disaggregated data for children and adolescents. The one exception is a paper by Snow and colleagues that reported tuberculosis notification data by 5-year age groups in people aged 10–24 years. For malaria, the Malaria Atlas Project (which informs the WHO World Malaria Report) reports total all-age estimates of cases and deaths in endemic countries and regions, but does not present detailed specific estimates of incidence or burden by age or sex; data are typically reported in children and adolescents younger than 5 years, aged 5–14 years, and aged 15–49 years. For HIV, UNAIDS provides annual estimates on populations living with HIV but does not typically stratify by age or gender across the developmental window (0–24 years) in their annual global updates, with the WHO Global Health Estimates (informed by UN partners, GBD, and other scientific studies) providing global, regional, and country-level estimates for various age bands, although adolescents older than 15 years are typically aggregated with adults. One exception is a paper by Zhang and colleagues based on GBD 2019 data that reported the burden associated with HIV and sexually transmitted infections for adolescents aged 10–24 years at the global, regional, and national level in 1990–2019. For diarrhoeal disease and pneumonia, available data are focussed on children younger than 5 years.

**Added value of this study**

This study provides a systematic and comprehensive analysis of communicable disease across the entire developmental window from birth to 24 years of age. Data are reported at a global level, across the gradient of sociodemographic development, and at a country level, disaggregated by age and sex where possible. Although aggregate data enable advocacy, granular data are essential for targeted action and monitoring of progress. We report data on incidence and mortality (typical metrics for communicable diseases), but add value by also reporting morbidity to illustrate the true effect of these largely preventable diseases; this is especially important for children and adolescents living in settings with high sociodemographic development. To further ensure as complete a picture of communicable disease burden as possible, we reviewed all the 369 causes modelled in GBD and included all communicable diseases, their clinical presentations, or direct sequelae in our reported estimates, resulting in 83 million DALYs that are in addition to the 420 million DALYs traditionally reported as communicable diseases in GBD 2019. We report the burden of vaccine-preventable diseases and the mortality-to-incidence ratio for HIV (a non-curable communicable disease) across available age groups as measures of health system performance.

**Implications of all the available evidence**

Our analysis calls for broader investments in communicable disease control. Although children younger than 5 years must remain a focus, older children and adolescents aged 5–24 years had 71 million DALYs in 2019 caused by communicable disease, a substantial burden of largely preventable disease. Diarrhoea, pneumonia, and malaria must remain a focus of action, but efforts must extend to include tuberculosis and HIV, especially for older children and adolescents. There is evidence that HIV has increased in burden for older children and adolescents, and that adolescents in many settings have excess HIV mortality. We must also extend efforts to address morbidity in addition to mortality; this brings into scope the substantial morbidity from communicable disease in children and adolescents in high-income settings. This new evidence has important implications for global policy, financing, resource allocation, and health systems; in all these efforts we must ensure that policies and services are responsive to the needs of all children and adolescents. This new evidence also requires us to consider the data we collect and report, which provide the essential foundation to accountable action. A shift from mortality to morbidity requires us to move beyond vital registration systems and to invest in strengthened population-based surveillance, which might include household and school-based surveys, but also strengthened health system monitoring. Recommended indicators for adolescents, including those recommended by the Global Action for Measurement of Adolescent health, can go further to include specific indicators of communicable disease. There is also a need to strengthen the evidence base for responsive actions.

An important question is whether these targets of policy and action remain relevant today. Most countries (75%) were forecasted to meet the SDG under-5 mortality goal by 2030 before the COVID-19 pandemic,^4^ and it is important to consider whether current interventions (largely focused on diarrhoea and pneumonia) will continue to be effective at driving sustained mortality reductions in children younger than 5 years.^5^, ^6^ Trends to 2019 have shown that the impressive gains in early childhood mortality have not extended to older children and adolescents.^7^, ^8^, ^9^ We have reported that adolescents have a substantial burden of communicable disease,^10^ that communicable diseases are important drivers of excess disease burden for adolescents in many settings,^11^ and that population growth (also driven by improvements in child survival) is greatest in settings in which adolescents have the greatest burden of communicable diseases, with clear implications for future health systems and resourcing.^11^ Yet the specific communicable diseases that drive disease burden in older children and adolescents have not been described in detail, a barrier to effective actions.^12^

The COVID-19 pandemic (and recent epidemics of mpox [formerly known as monkeypox], H1N1 influenza, Zika virus, Ebola, and severe acute respiratory syndrome, for example)^13^ underscores the urgent need to take stock of communicable disease control. Some of these emergent diseases have impacted adolescents more than younger children,^14^ challenging the almost exclusive focus on younger children within existing communicable disease control. COVID-19 has exposed deficiencies and inequities in our health systems, with resultant public health measures further entrenching some of these inequities through disruption to health and social services, particuarly education.^15^, ^16^, ^17^ Key preventive interventions, such as vaccination and school-based health education and screening, have been impacted, particularly in low-income and middle-income countries.^15^, ^18^, ^19^, ^20^ There are now important opportunities for individual countries to build back better, which include improving health and social services, but to do so, we must understand the foundations upon which we are building.^21^ Here, we use the Institute of Health Metrics and Evaluation (IHME) Global Burden of Disease Study 2019 (GBD 2019) to systematically characterise the burden of communicable disease mortality and morbidity between 1990 and 2019 by age, sex, and sociodemographic development, globally and within 204 countries and territories. We focus on the developmental window from birth to 24 years of age, when disease burden changes markedly, as do opportunities for intervention. This manuscript was produced as part of the GBD Collaborator Network and in accordance with the GBD Protocol.

## Methods

Broader methods relating to GBD 2019, including primary data sources, approaches to disease modelling, and definitions of disease outcomes, are detailed elsewhere.^22^, ^23^ Hereafter we present specific methods and assumptions of relevance to this secondary data analysis.

### Data sources

We used GBD 2019 data accessed from the IHME Global Health Data Exchange. We accessed data between Dec 10, 2021, and Nov 4, 2022 for all causes (at level 4) as absolute numbers and rates per 100 000 population for the following metrics: mortality (deaths and years of life lost [YLLs]); disability (years lived with disability [YLDs]); and disease burden (measured as disability-adjusted life-years [DALYs]). We accessed data for all ages (in 5-year age bands up to 24 years, then for 25 years and older), for males and females, for all years between 1990 and 2019, and for 204 countries and territories. We also accessed the IHME sociodemographic index to group countries at similar levels of sociodemographic development, comprising high, mid-high, middle, mid-low, and low socioeconomic development (appendix pp 1–4). GBD 2019 complies with the Guidelines for Accurate and Transparent Health Estimates Reporting statement and all data input sources and statistical codes are available online.^24^, ^25^

### Definitions

GBD 2019 includes 369 causes of disease and injury organised within three disease groups: communicable, maternal, neonatal, and nutritional diseases (group A); non-communicable diseases (group B); and injuries (group C; appendix pp 5–6). For this analysis we included all causes of communicable disease in group A (cause groups A1 to A5); these causes include specific infectious diseases (eg, cause group A.1.1, HIV) and clinical presentations of communicable disease (eg, cause group A.2.2, lower-respiratory-tract infection). We then reviewed all other causes included within group B and group C in GBD 2019 and their corresponding International Classification of Disease codes to identify other relevant communicable diseases.^26^ Maternal sepsis (A.6.1.2), neonatal sepsis (A.6.2.3), rheumatic heart disease (B.2.1), bacterial skin disease (B.9.3), scabies (B.9.4), fungal skin disease (B.9.5), viral skin disease (B.9.6), liver cancer caused by hepatitis B and C (B.1.7.1 and B.1.7.2), and cirrhosis and chronic liver disease caused by hepatitis B and C (B.4.1.1 and B.4.1.2) were all included within our definition of communicable diseases (appendix pp 5–6). We did not include cervical cancer (B.1.15), given that modelled estimates are not specific to human papillomavirus. Our definition of communicable diseases yielded a total disease burden of 503 296 274 DALYs in 2019 for all age groups compared with 420 392 536 for cause groups A1–A5. All communicable diseases included in our analysis were additionally subcategorised into 16 subgroupings that represent common communicable diseases or clinical presentations (appendix pp 5–6).

This analysis focused on the developmental window of childhood and adolescence. Consistent with newer understandings of neurodevelopment but also global shifts in the timing of key social role transitions (such as completion of education and parenthood), we define childhood as being younger than 10 years and adolescence as being 10–24 years of age.^27^ Data are reported in 5-year age bands within these broad definitions of childhood and adolescence; we did not further disaggregate the under 5-year age band given that these estimates are extensively reported elsewhere. We additionally defined three aggregate groups that represent key target populations within the health sector: young children (younger than 5 years), older children and young adolescents who are still consistently cared for by paediatric services (5–14 years),^28^ and older adolescents (15–24 years).^27^ We use the Socio-demographic Index, a composite indicator of development based on the geometric mean of total fertility (younger than 25 years), mean education (15 years and older), and lag-distributed income per capita.

### Analysis and reporting

Data were reported as absolute count, cases per 100 000 population, and incidence (cases of new disease per 100 000 population per year) across measures of mortality, disability (YLD or morbidity), and disease burden (DALYs). Of note, GBD 2019 does not model deaths caused by scabies, or fungal or viral skin conditions. Estimates were reported for each of 204 locations separately, across sociodemographic development groupings, and for adolescents globally, noting that the global estimate is greater than the sum of 204 individual nations or territories, because it includes people who are stateless and additional nations and territories. We estimated the percentage change between 1990 and 2019 (difference between 2019 and 1990 value divided by the 1990 value and multiplied by 100), reporting this value as an annualised percentage change (dividing by 30 years). Where possible, we also reported the corresponding uncertainty interval (UI) for each estimate. This interval is produced for each estimate by running 1000 draws of the posterior distribution, ordering the draws, and selecting the 25th and 975th draw values.^29^ The code that is used to produce the estimates is available online.^25^ Given that UIs are obtained during modelling, these intervals are not available for some aggregate estimates that we provide in this analysis (eg, the 0–24-year age group for total communicable disease cause). Uncertainty for each cause that contributes to our aggregate estimates by age, sex, and country is detailed in the appendix (pp 87–167).

As a measure of health system response to communicable disease, we reported the mortality-to-incidence ratio (MIR) for HIV.^20^, ^30^ The MIR calculation was only estimated for HIV given that it is a true chronic communicable disease for which a definitive objective diagnosis is typical, and treatment, remission, and several incident infections are not possible. MIR was calculated by dividing the number of cause-specific deaths by the number of new cases for a given year. We reported this metric for children younger than 5 years, adolescents aged 15–19 years, and adolescents aged 20–24 years; HIV incidence for children and adolescents aged 5–14 years was modelled to be negligible by GBD and is excluded here.

Data were analysed in Stata 17.0 and visualisations prepared in Stata and Tableau 2021.3.20.

### Role of the funding source

The funders of the study had no role in study design, data collection, data analysis, data interpretation, or writing of the report.

## Results

### Total burden of communicable disease across childhood and adolescence

In 2019, there were 3·0 million deaths and 30·0 million years of healthy life lost to disability (as measured by YLDs) from communicable diseases globally among people aged 0–24 years, corresponding to a total disease burden of 288·4 million DALYs (table 1). This burden represents 57·3% of the total communicable disease burden across all ages. For children and adolescents specifically, communicable disease accounted for 44·1% of the total 6·9 million deaths in this age group, 16·6% of the total disability, and 37·9% of the total 760·0 million all-cause DALYs (table 1). Globally, the proportion of deaths caused by communicable diseases in 2019 was between 41·2% and 55·9% for those aged 0–14 years, decreasing to between 20·6% and 33·9% among those aged 15–24 years (table 1). This pattern was similar for disability, with the proportion of YLDs attributable to communicable diseases consistently declining with increasing age. Communicable disease burden among children and adolescents was predominantly in countries of low sociodemographic development, with 1·8 million deaths (58·2% of all communicable disease deaths among children and adolescents) and 161·4 million DALYs (56·0% of all communicable disease DALYs among children and adolescents; appendix pp 7–10). More than half of the mortality among children and adolescents in settings of low sociodemographic development was caused by communicable diseases compared with just 5·6% of deaths and 7·1% of DALYs in settings of high sociodemographic development (appendix pp 8–10).

**Table 1:** Age and sex-specific estimates of deaths, YLDs, and DALYs (all-cause and communicable-disease specific), in 2019

	**Population**	**Mortality (deaths)**			**Disability (YLDs)**			**Disease burden (DALYs)**		
		All-cause	Communicable disease	Percentage due to communicable diseases	All-cause	Communicable disease	Percentage due to communicable diseases	All-cause	Communicable disease	Percentage due to communicable diseases
**Under 5 years**										
Female	320 443 936	2 311 567	1 154 730	50·0%	12 885 506	3 340 269	25·9%	216 237 639	104 465 025	48·3%
Male	342 398 784	2 732 001	1 245 653	45·6%	14 182 583	3 600 909	25·4%	254 728 403	112 733 037	44·3%
**5–9 years**										
Female	316 754 496	167 708	93 711	55·9%	13 824 110	3 389 243	24·5%	27 539 772	11 053 467	40·1%
Male	337 949 216	210 032	106 164	50·5%	15 108 232	3 693 489	24·4%	32 272 747	12 370 285	38·3%
**10–14 years**										
Female	310 852 512	129 193	60 936	47·2%	17 981 681	2 794 176	15·5%	27 855 730	7 451 772	26·8%
Male	331 334 176	170 079	70 032	41·2%	16 907 999	2 974 792	17·6%	29 903 595	8 326 975	27·8%
**15–19 years**										
Female	301 758 880	196 977	66 846	33·9%	23 120 691	2 660 741	11·5%	37 193 646	7 436 890	20·0%
Male	317 782 112	302 328	77 986	25·8%	18 794 347	2 490 160	13·2%	40 371 375	8 058 373	20·0%
**20–24 years**										
Female	295 776 256	255 141	81 310	31·9%	27 205 707	2 676 953	9·8%	44 184 802	8 087 317	18·3%
Male	304 368 224	437 271	90 184	20·6%	20 650 899	2 420 924	11·7%	49 742 667	8 421 269	16·9%
**≥25 years**										
Female	2 310 904 064	22 784 061	2 905 445	12·8%	381 599 235	18 658 273	4·9%	837 038 989	92 819 766	11·1%
Male	2 247 142 144	26 830 603	3 589 282	13·4%	298 712 790	18 158 028	6·1%	940 950 706	122 072 101	13·0%
**0–24 years**										
Female and male	3 179 418 560	6 912 296	3 047 551	44·1%	180 661 755	30 041 657	16·6%	760 030 375	288 404 407	37·9%
Female	1 545 586 080	3 060 585	1 457 533	47·6%	95 017 694	14 861 382	15·6%	353 011 588	138 494 470	39·2%
Male	1 633 832 512	3 851 711	1 590 018	41·3%	85 644 061	15 180 275	17·7%	407 018 787	149 909 938	36·8%

DALYs=disability-adjusted life-years. YLDs=years of life lost to disability.

**Figure 1:**
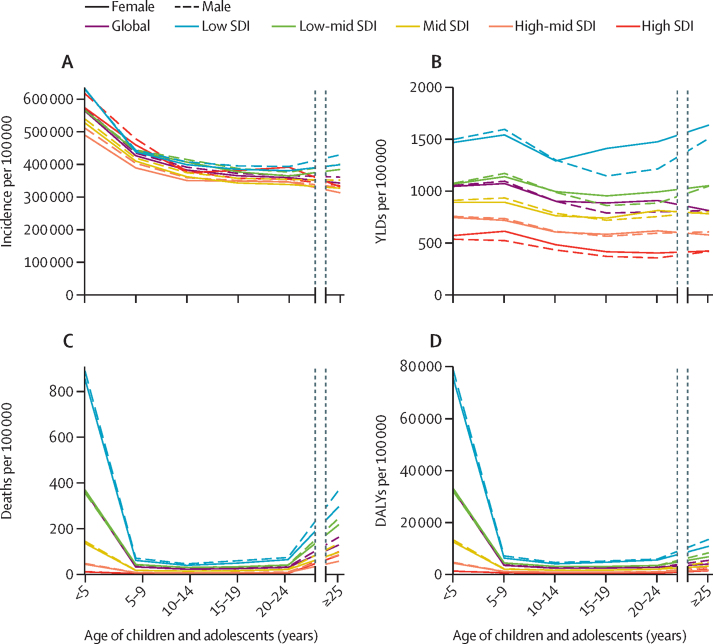
Communicable disease incidence (A), YLDs (B), mortality (C), and DALYs (D) by age, sex, and SDI, in 2019 DALY=disability-adjusted life years. SDI=Socio-demographic Index. YLD=years of life lost to disability.

There were important changes in communicable disease epidemiology across childhood and adolescence (figure 1). Communicable disease incidence (figure 1A; appendix pp 11–12) was highest in children younger than 5 years (569 924 communicable diseases per 100 000 population for both sexes globally) but remained high across childhood (431 375 communicable diseases per 100 000 population for children aged 5–9 years) and for adolescents aged 15–19 years (369 246 communicable diseases per 100 000 population). There appeared to be little difference in all-cause incidence by sex or socioeconomic development. Disability caused by communicable disease as measured by YLDs (figure 1B; appendix pp 13–14) was relatively similar across age and sex, but with marked variation across socioeconomic development. Communicable diseases in children and adolescents aged 0–24 years caused 1407 YLDs per 100 000 population in settings of low sociodemographic development and 458 YLDs per 100 000 population in settings of high sociodemographic development. There was marked variation in mortality (figure 1C; table 1; appendix pp 15–16) by age and sociodemographic development, with mortality caused by communicable diseases greatest for children younger than 5 years (mortality rate 362·1 per 100 000 population and a total of 2·4 million deaths in 2019), especially for children younger than 5 years in settings of low sociodemographic development (869 deaths per 100 000 population for both sexes). However, the relatively low mortality throughout later childhood and adolescence still corresponded to a substantial number of deaths; overall, 647 169 deaths from communicable diseases occurred among children and adolescents aged 5–24 years in 2019, corresponding to 6·8% of total deaths from communicable diseases and 34·6% of all deaths in this age group (table 1). In 2019, DALYs (figure 1D; appendix pp 17–18) were largely driven by mortality (288·4 million DALYs for individuals aged 0–24 years, comprising 30·0 million YLDs and 258·4 million YLLs), and as a result, trends in DALYs largely mirrored trends in mortality.

**Figure 2:**
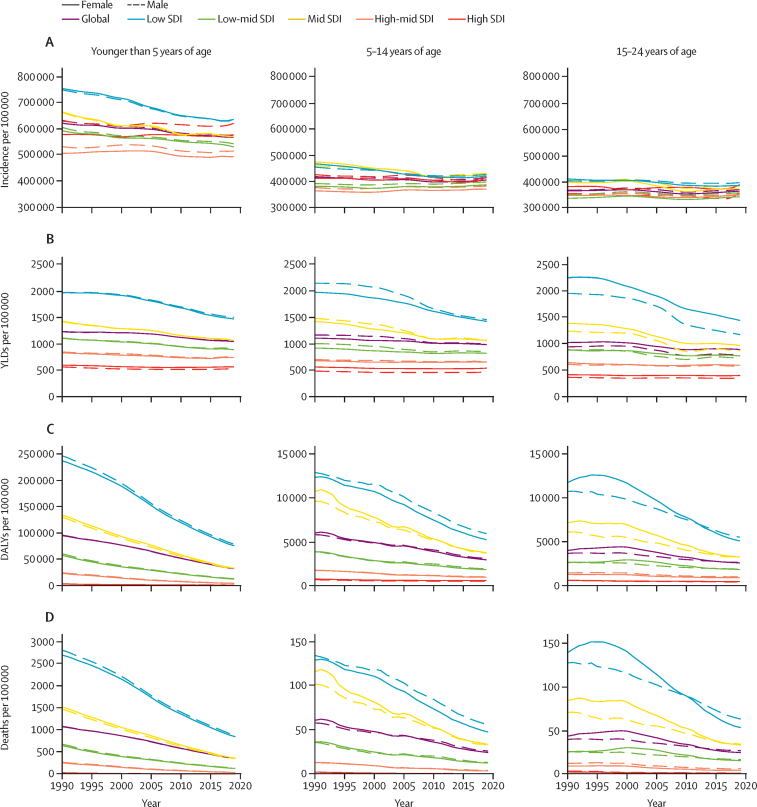
Trends over time (1990–2019) for communicable disease incidence, deaths, YLDs, and DALYs per 100 000 by age, sex, and SDI The incidence graphs start at 300 000 cases per 100 000 per year and the age groups younger than 5 years are on a different y-scale for deaths and DALYs. DALY=disability-adjusted life-years. SDI=Socio-demographic Index. YLD=years of life lost to disability.

Communicable disease epidemiology changed over time from 1990 to 2019 (figure 2). Incidence of communicable diseases (figure 2A) declined most markedly for children younger than 5 years, and especially for those younger than 5 years in settings of low sociodemographic development, with an annual decline of around 0·5% in settings of low sociodemographic development compared with a 0·03% decline in settings of high sociodemographic development. As a result, in 2019 the incidence of communicable diseases for children younger than 5 years in settings of high sociodemographic development (597 655 communicable diseases per 100 000 population) was similar to that in settings of low sociodemographic development (633 509 communicable diseases per 100 000 population). The change in incidence among older children and adolescents across sociodemographic development settings was small. Disability as measured by YLDs (figure 2B) changed little globally for children and adolescents (0·5% for those aged <5 years, 0·4% for those aged 5–14 years, and 0·5% for those aged 15–24 years); however, there were marked reductions in YLDs in settings of low sociodemographic development (1·0% annual decline for individuals aged 0–24 years overall). Mortality caused by communicable diseases (figure 2D; appendix pp 15–16) declined most markedly for children younger than 5 years (2·2% annual decline globally) and those aged 5–14 years (1·9% annual decline globally), with changes for adolescents aged 15–24 years being more modest (1·3% annual decline globally). Declines in mortality between 1990 and 2019 were most marked in settings of low sociodemographic development, where for children younger than 5 years, deaths decreased from 2752 per 100 000 to 869 deaths per 100 000 between 1990 and 2019 (an average decline of 2·3% per year). Shifts in total disease burden (figure 2C) largely mirrored shifts in mortality. At a global level, these relative transitions in epidemiology by age and sociodemographic development resulted in the communicable disease burden increasingly shifting from children younger than 5 years to older children and adolescents between 1990 and 2019 (DALYs in appendix p 20 and mortality in appendix p 21). In 1990, 85% of the communicable disease burden across the developmental window was among children younger than 5 years, decreasing to 75% in 2019.

**Figure 3:**
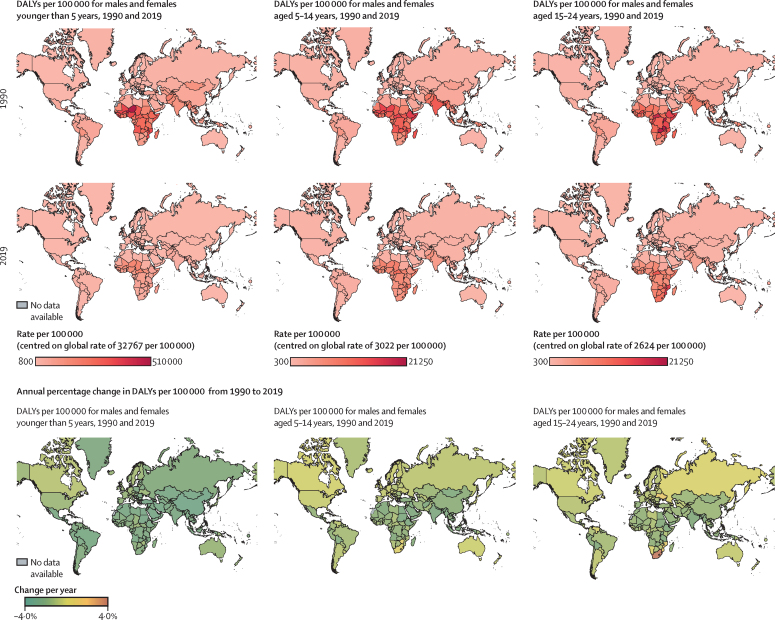
Communicable disease burden in 1990 and 2019, and its annual rate of change by country and age The colour scheme for the DALY rate in 1990 and 2019 is specific for each age group and the colour band is centred around the global rate of DALYs and reported on each legend. DALY=disability-adjusted life-years.

Total disease burden caused by communicable diseases (as measured by DALYs) for children and adolescents at a country level changed from 1990 to 2019 (figure 3). For children younger than 5 years, there were uniform and significant reductions in the communicable disease burden (especially noting the magnitude of reduction among these children under 5 years given the unique axis values for DALYs in figure 3), compared with children and adolescents aged 5–14 years and adolescents aged 15–24 years. Although most countries showed a decline in communicable disease burden, large increases (>2% annual increase) in burden were seen in Eswatini, Lesotho, and South Africa, for the 15–24-year age group. In 2019, countries in sub-Saharan Africa and some countries in Asia had the largest burden of communicable diseases for children and adolescents.

### Cause-specific estimates of communicable diseases in children and adolescents

60% of communicable disease burden (as measured by DALYs) among children and adolescents was accounted for by three cause groups in 2019 (table 2), comprising enteric infections (69·5 million DALYs, 24·1% total), lower-respiratory-tract infections (LRTIs; 64·7 million DALYs, 22·4% total), and malaria (38·3 million DALYs, 13·3% total). These same cause groups together accounted for almost two-thirds of all deaths caused by communicable diseases among children and adolescents: enteric infections (763 545 deaths, 25·1% of deaths caused by communicable diseases), LRTIs (743 546 deaths, 24·4% of deaths caused by communicable diseases), and malaria (427 469 deaths, 14·0% of deaths caused by communicable diseases).

**Table 2:** Cause-specific estimates of DALYs and deaths for each communicable disease in 2019 and annual rate of change since 1990, for children and adolescents aged 0–24 years by sex

	**Mortality (deaths)**			**Disability**			**Disease Burden**		
	Number of deaths	Deaths per 100 000 (percentage change per year)	Percentage of deaths caused by communicable diseases	Number of YLDs	YLDs per 100 000 (percentage change per year)	Percentage of YLDs caused by communicable diseases	Number of DALYs	DALYs per 100 000 (percentage change per year)	Percentage of DALYs caused by communicable diseases
**Enteric infections**									
Female	355 016	23·0 (−2·3%)	24·4%	2 426 525	157·0 (−0·4%)	16·3%	32 420 734	2 097·6 (−2·2%)	23·4%
Male	408 529	25·0 (−2·2%)	25·7%	2 599 957	159·1 (−0·4%)	17·1%	37 102 149	2 270·9 (−2·2%)	24·7%
**Lower-respiratory-tract infections**									
Female	363 980	23·5 (−2·4%)	25·0%	102 760	6·6 (−1·4%)	0·7%	31 693 882	2 050·6 (−2·4%)	22·9%
Male	379 566	23·2 (−2·4%)	23·9%	119 028	7·3 (−1·3%)	0·8%	33 012 492	2 020·6 (−2·4%)	22·0%
**Malaria**									
Female	211 315	13·7 (−1·5%)	14·5%	1 126 500	72·9 (0·0%)	7·6%	19 166 971	1 240·1 (−1·5%)	13·8%
Male	216 154	13·2 (−1·5%)	13·6%	889 966	54·5 (−0·2%)	5·9%	19 172 678	1 173·5 (−1·4%)	12·8%
**Neonatal sepsis and other neonatal infections**									
Female	100 303	6·5 (−0·8%)	6·9%	1 000 143	64·7 (7·9%)	6·7%	9 908 325	641·1 (−0·6%)	7·2%
Male	126 214	7·7 (−0·8%)	7·9%	1 060 359	64·9 (8·8%)	7·0%	12 270 216	751·0 (−0·7%)	8·2%
**Vaccine-preventable disease**									
Female	117 278	7·6 (−2·8%)	8·0%	105 275	6·8 (−2·0%)	0·7%	10 215 674	661·0 (−2·8%)	7·4%
Male	111 480	6·8 (−2·8%)	7·0%	94 315	5·8 (−2·1%)	0·6%	9 703 923	593·9 (−2·8%)	6·5%
**Meningitis and encephalitis**									
Female	82 954	5·4 (−2·2%)	5·7%	257 998	16·7 (−1·1%)	1·7%	7 207 750	466·3 (−2·2%)	5·2%
Male	101 377	6·2 (−2·0%)	6·4%	274 568	16·8 (−1·3%)	1·8%	8 767 715	536·6 (−2·0%)	5·8%
**HIV and AIDS**									
Female	74 914	4·8 (0·2%)	5·1%	345 448	22·4 (2·4%)	2·3%	6 051 479	391·5 (0·2%)	4·4%
Male	64 035	3·9 (0·8%)	4·0%	219 471	13·4 (6·1%)	1·4%	5 226 905	319·9 (0·6%)	3·5%
**Tuberculosis**									
Female	54 313	3·5 (−2·5%)	3·7%	708 837	45·9 (−1·3%)	4·8%	4 985 849	322·6 (−2·4%)	3·6%
Male	62 201	3·8 (−2·3%)	3·9%	492 195	30·1 (−1·1%)	3·2%	5 287 041	323·6 (−2·3%)	3·5%
**Neglected tropical diseases**									
Female	15 834	1·0 (−2·4%)	1·1%	2 330 032	150·8 (−1·4%)	15·7%	3 621 573	234·3 (−1·9%)	2·6%
Male	24 779	1·5 (−2·3%)	1·6%	2 476 188	151·6 (−1·7%)	16·3%	4 465 254	273·3 (−2·1%)	3·0%
**Infectious skin conditions**									
Female	3278	0·2 (−1·3%)	0·2%	3 344 347	216·4 (−0·1%)	22·5%	3 617 997	234·1 (−0·3%)	2·6%
Male	2421	0·1 (−1·5%)	0·2%	3 678 489	225·1 (−0·1%)	24·2%	3 872 745	237·0 (−0·2%)	2·6%
**Sexually transmitted infections excluding HIV**									
Female	37 374	2·4 (−0·9%)	2·6%	75 221	4·9 (0·4%)	0·5%	3 378 042	218·6 (−0·9%)	2·4%
Male	45 258	2·8 (−0·9%)	2·8%	58 207	3·6 (0·2%)	0·4%	4 068 741	249·0 (−0·9%)	2·7%
**Upper-respiratory-tract infections**									
Female	2043	0·1 (−2·8%)	0·1%	1 991 663	128·9 (−0·1%)	13·4%	2 167 122	140·2 (−1·1%)	1·6%
Male	2915	0·2 (−2·7%)	0·2%	2 282 909	139·7 (−0·1%)	15·0%	2 532 781	155·0 (−1·0%)	1·7%
**Other unspecified infectious diseases**									
Female	10 559	0·7 (−1·2%)	0·7%	562 619	36·4 (−0·2%)	3·8%	1 452 672	94·0 (−0·9%)	1·0%
Male	14 644	0·9 (−1·3%)	0·9%	515 999	31·6 (−0·3%)	3·4%	1 723 549	105·5 (−1·1%)	1·1%
**Hepatitis**									
Female	14 837	1·0 (−2·3%)	1·0%	91 598	5·9 (−0·2%)	0·6%	1 249 223	80·8 (−2·3%)	0·9%
Male	21 079	1·3 (−2·1%)	1·3%	105 110	6·4 (−0·3%)	0·7%	1 714 000	104·9 (−2·1%)	1·1%
**Rheumatic heart disease**									
Female	8719	0·6 (−1·9%)	0·6%	351 987	22·8 (0·9%)	2·4%	987 240	63·9 (−1·5%)	0·7%
Male	9366	0·6 (−1·6%)	0·6%	313 517	19·2 (0·9%)	2·1%	989 751	60·6 (−1·2%)	0·7%
**Maternal sepsis and other maternal infections**									
Female	4815	0·3 (−2·3%)	0·3%	40 429	2·6 (−1·2%)	0·3%	369 940	23·9 (−2·2%)	0·3%
Male	..	..	..	..	..	..	..	..	..
**Total communicable diseases**									
Female	1 457 533	94·3 (−2·2%)	100·0%	14 861 382	961·5 (−0·4%)	100·0%	138 494 470	8960·4 (−2·2%)	100·0%
Male	1 590 018	97·3 (−2·2%)	100·0%	15 180 275	929·1 (−0·5%)	100·0%	149 909 936	9175·6 (−2·2%)	100·0%

Results are ordered by largest DALYs burden. For skin disease, only bacterial skin disease contributes to the value for deaths, because there were no deaths recorded for fungal and viral skin diseases or scabies. DALY=disability-adjusted life-years. YLD=years of life lost to disability.

Contributors to communicable disease burden (as measured by DALYs) by age, sex, and sociodemographic development are presented in the appendix (p 19). LRTIs and enteric infections were the leading causes of communicable disease burden across childhood and early-to-mid adolescence globally. For older adolescents aged 20–24 years, HIV and tuberculosis emerged as leading causes. HIV caused 20·9% of the communicable disease burden in females and 10·3% in males, and 28·3% of age-specific deaths in females and 13·2% in males aged 20–24 years (appendix pp 15, 18). Tuberculosis caused 18·7% of the communicable disease burden in males and 14·0% in females, and 22·6% of age-specific deaths in males and 15·9% in females aged 20–24 years. Rheumatic heart disease, maternal sepsis, and sexually transmitted infections (STIs; excluding HIV) were other communicable diseases that predominantly emerged during adolescence, albeit with a considerably smaller burden.

Causes of communicable diseases varied substantially by sociodemographic development (appendix pp 11–19). In settings of low sociodemographic development, enteric diseases and LRTIs were the leading causes of DALYs among children and adolescents (with enteric diseases causing 25·9% of the burden and LRTIs causing 21·6% of the burden in settings of low sociodemographic development), whereas infectious skin conditions and upper-respiratory-tract infections (URTIs) were the leading causes in settings of high sociodemographic development (with infectious skin diseases causing 28·7% of the burden and URTIs causing 22·1% of the burden). Of note, DALYs caused by skin infections and URTIs were mostly caused by YLDs, with little mortality attributed to these causes (total of 10 647 deaths globally, 0·3% of the total communicable disease deaths). Conditions such as neonatal sepsis, maternal sepsis, and meningitis predominantly affected children and adolescents in settings of low and middle sociodemographic development, with rheumatic heart disease and neglected tropical diseases exclusively so. By contrast, hepatitis and STIs affected children and adolescents across all sociodemographic development settings.

There was an overall reduction in burden (as measured by DALYs) for the key causes of communicable diseases globally between 1990 and 2019 (table 2). Annual declines in DALYs of at least 2% were seen for eight cause groups, comprising enteric infections, LRTIs, vaccine-preventable diseases, meningitis and encephalitis, tuberculosis, neglected tropical diseases, hepatitis, and maternal sepsis and other maternal infections. Malaria burden only declined 1·5% annually and declines in infectious skin conditions, neonatal infections, and STIs were less than 1% annually. These declines were seen largely in settings of low and middle sociodemographic development (appendix p 17). The only disease to increase in burden over time was HIV (0·2% annual increase for males and 0·6% for females). HIV burden increased most for children and adolescents in settings of middle sociodemographic development (14·3% annual increase for females and 12·1% for males; appendix p 17), and across settings, increases were most marked for children aged 5–9 years (13·3% annual increase for females and 13·5% increase for males globally), those aged 10–14 years (128·2% for females and 83·6% for males globally), and male adolescents aged 15–19 years (22·3% annual increase; appendix p 18). With respect to incidence, the causes where there has been an increased incidence over time globally included rheumatic heart disease and neglected tropical diseases (particularly among adolescents), and STIs in some settings of low and low-middle sociodemographic development.

### Key findings by leading cause groups for children and adolescents

**Figure 4:**
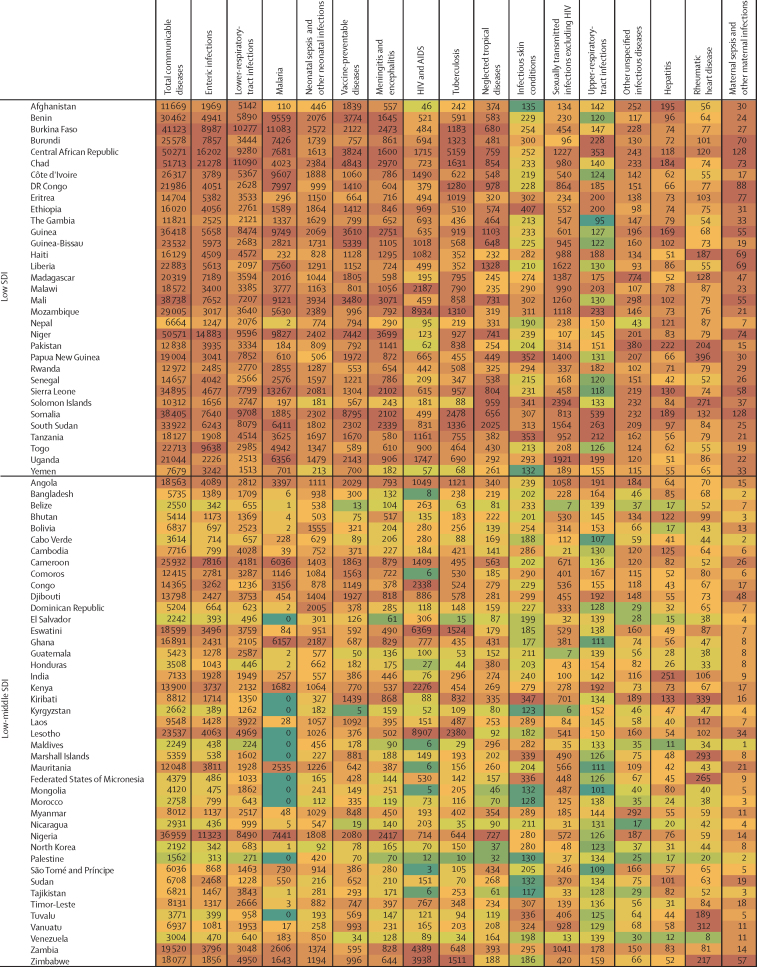

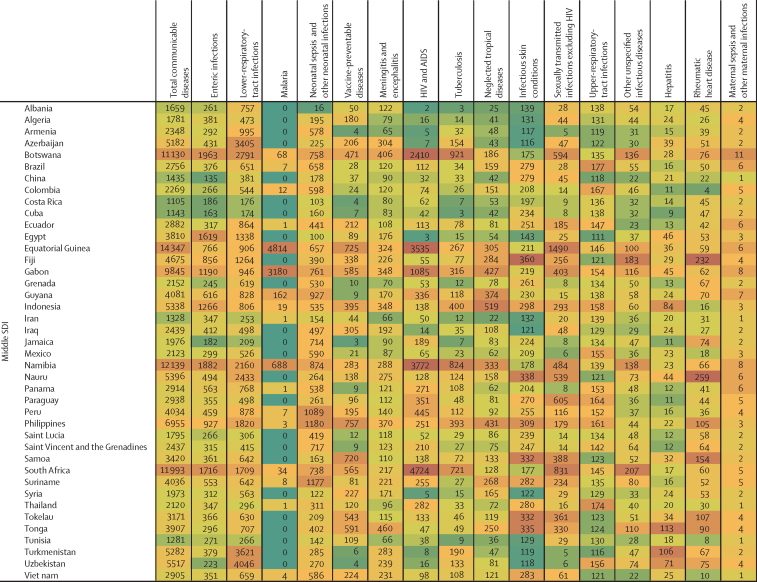

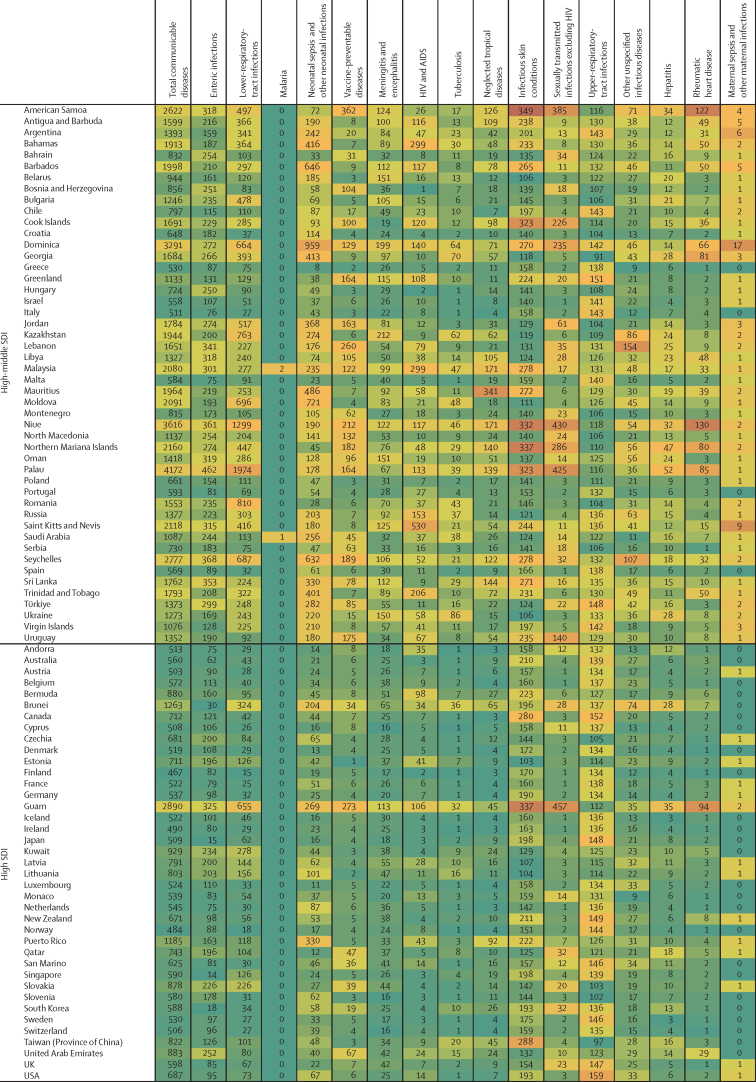
Country-level heatmap of communicable disease DALYs by cause for children and adolescents aged 0–24 years in 2019, grouped by SDI The shading ranges from green, which indicates a low number of DALYs per 100 000 for that country within the disease, whereas the highest rates are shaded in a dark orange colour, which indicates a country has a large DALY burden. DALY=disability-adjusted life-years. SDI=Socio-demographic Index.

In this section we focus on five major contributors to overall communicable disease burden in children and adolescents, comprising enteric infections, LRTIs, and malaria, as well as tuberculosis and HIV which both emerged as key causes of burden in older adolescents; these five conditions accounted for 71·9% of communicable disease-related deaths and 67·3% of DALYs in those aged 0–24 years (69·7% of DALYs for children <5 years, 58·8% for those aged 5–14 years, and 61·2% for adolescents aged 15–24 years; appendix p 18). The countries that contribute the largest burden for these five causes are reported in table 3 (the specific burden in each country is reported in figure 4). It is worth additionally noting the burden of vaccine-preventable disease given that it is a marker of health-system performance. In 2019, globally there were 228 758 deaths in children and adolescents from diphtheria, pertussis, tetanus, measles, and varicella, 153 169 (66·9%) of which occurred in settings of low sociodemographic development.

**Table 3:** The ten countries with the highest percentage burden (DALYs) for enteric infections, HIV and AIDS, lower-respiratory-tract infections, malaria, and tuberculosis, for the three age groups

	**Enteric infections**	**Lower-respiratory-tract infections**	**Malaria**	**Tuberculosis**	**HIV and AIDS**
**<5 years**					
1	Nigeria (27·1%)	Nigeria (19·2%)	Nigeria (26·8%)	Nigeria (14·2%)	Mozambique (19·8%)
2	India (11·2%)	India (19·2%)	Democratic Republic of the Congo (12·4%)	India (10·2%)	Nigeria (12·9%)
3	Pakistan (6·3%)	Pakistan (6·9%)	Uganda (4·9%)	Pakistan (10·0%)	Ethiopia (6·8%)
4	Chad (4·4%)	Ethiopia (2·9%)	Niger (4·6%)	Democratic Republic of the Congo (8·6%)	Zambia (6·2%)
5	Ethiopia (4·4%)	Niger (2·6%)	Burkina Faso (4·6%)	Somalia (4·1%)	Kenya (5·2%)
6	Niger (4·4%)	Tanzania (2·5%)	Côte d'Ivoire (3·9%)	Tanzania (3·2%)	South Africa (3·9%)
7	Democratic Republic of the Congo (3·7%)	Burkina Faso (2·4%)	Mali (3·9%)	Ethiopia (3·1%)	India (3·8%)
8	Cameroon (2·5%)	China (2·2%)	Tanzania (3·7%)	Angola (3·1%)	Uganda (3·6%)
9	Burkina Faso (2·2%)	Somalia (2·1%)	Ethiopia (3·2%)	Chad (3·0%)	Zimbabwe (2·9%)
10	Madagascar (1·9%)	Democratic Republic of the Congo (2·1%)	Ghana (2·8%)	Burkina Faso (3·0%)	Tanzania (2·7%)
**5–14 years**					
1	India (35·6%)	India (21·3%)	Nigeria (24·5%)	India (20·2%)	Mozambique (14·4%)
2	Pakistan (11·7%)	Pakistan (7·5%)	India (15·2%)	Pakistan (17·6%)	South Africa (12·7%)
3	Nigeria (9·9%)	Nigeria (5·7%)	Democratic Republic of the Congo (8·8%)	Democratic Republic of the Congo (7·4%)	Ethiopia (6·0%)
4	Bangladesh (4·0%)	Bangladesh (4·1%)	Mozambique (4·9%)	Nigeria (5·2%)	Kenya (5·8%)
5	Ethiopia (2·7%)	Democratic Republic of the Congo (3·8%)	Pakistan (4·0%)	Indonesia (4·2%)	Uganda (5·4%)
6	Indonesia (2·5%)	Ethiopia (3·3%)	Uganda (3·6%)	Philippines (3·6%)	Nigeria (5·0%)
7	Democratic Republic of the Congo (2·0%)	Philippines (3·2%)	Côte d'Ivoire (3·4%)	Bangladesh (3·4%)	Zimbabwe (4·8%)
8	Tanzania (1·6%)	China (2·6%)	Cameroon (2·7%)	Ethiopia (2·6%)	Tanzania (4·5%)
9	Kenya (1·5%)	Egypt (2·4%)	Niger (2·5%)	Somalia (2·3%)	Malawi (4·3%)
10	Mali (1·3%)	Tanzania (2·4%)	Burkina Faso (2·4%)	South Africa (2·2%)	Zambia (3·5%)
**15–24 years**					
1	India (39·7%)	India (18·3%)	Nigeria (29·2%)	India (26·8%)	South Africa (14·7%)
2	Pakistan (9·7%)	Nigeria (4·8%)	India (6·2%)	Pakistan (9·3%)	Mozambique (12·4%)
3	Nigeria (6·4%)	Democratic Republic of the Congo (4·0%)	Democratic Republic of the Congo (6·0%)	Indonesia (6·2%)	Kenya (6·9%)
4	Ethiopia (3·4%)	Ethiopia (3·5%)	Côte d'Ivoire (4·7%)	Democratic Republic of the Congo (4·8%)	Nigeria (6·6%)
5	Indonesia (3·3%)	Philippines (3·4%)	Yemen (4·5%)	Ethiopia (4·1%)	India (5·4%)
6	Bangladesh (3·0%)	Pakistan (3·2%)	Cameroon (4·0%)	Nigeria (3·8%)	Ethiopia (5·1%)
7	Kenya (1·9%)	China (2·9%)	Ghana (3·9%)	Somalia (2·9%)	Uganda (4·4%)
8	Democratic Republic of the Congo (1·9%)	Brazil (2·7%)	Mozambique (3·7%)	Mozambique (2·8%)	Tanzania (4·2%)
9	Tanzania (1·6%)	Tanzania (2·0%)	Burkina Faso (3·0%)	Tanzania (2·4%)	Zambia (3·5%)
10	China (1·5%)	Kenya (2·0%)	Uganda (2·5%)	Philippines (2·3%)	Cameroon (2·7%)
**0–24 years**					
1	Nigeria (22·4%)	India (19·3%)	Nigeria (26·7%)	India (18·4%)	Mozambique (15·6%)
2	India (17·8%)	Nigeria (18·0%)	Democratic Republic of the Congo (11·6%)	Pakistan (10·8%)	South Africa (10·2%)
3	Pakistan (7·5%)	Pakistan (6·9%)	Uganda (4·6%)	Nigeria (8·6%)	Nigeria (8·7%)
4	Ethiopia (4·0%)	Ethiopia (3·0%)	India (4·3%)	Democratic Republic of the Congo (6·9%)	Kenya (6·1%)
5	Niger (3·5%)	Tanzania (2·5%)	Burkina Faso (4·3%)	Indonesia (4·3%)	Ethiopia (6·0%)
6	Chad (3·5%)	Niger (2·5%)	Niger (4·2%)	Ethiopia (3·4%)	Zambia (4·5%)
7	Democratic Republic of the Congo (3·2%)	Burkina Faso (2·3%)	Côte d'Ivoire (3·9%)	Somalia (3·3%)	India (4·3%)
8	Cameroon (2·0%)	China (2·3%)	Mali (3·5%)	Tanzania (2·6%)	Uganda (4·3%)
9	Indonesia (2·0%)	Democratic Republic of the Congo (2·3%)	Tanzania (3·4%)	Mozambique (2·5%)	Tanzania (3·7%)
10	Burkina Faso (1·9%)	Somalia (2·1%)	Mozambique (2·9%)	Philippines (2·2%)	Zimbabwe (3·2%)

The denominator is the total number of DALYs associated with the condition and age grouping. DALY=daily-adjusted life-years.

In 2019, there were about 69·5 million DALYs caused by enteric infections among children and adolescents (table 2; appendix pp 18, 22–26), of which about 41·9 million occurred in settings of low sociodemographic development and 19·6 million in settings of low-middle sociodemographic development (88·3% of total combined). Globally, almost three quarters of this burden (74·5%) was in children younger than 5 years, but the burden among those aged 5–24 years was substantial (17·7 million DALYs and 190 487 deaths globally). Three countries (India, Nigeria, and Pakistan) together accounted for almost half (47·7%) of the total burden of enteric infection globally for children and adolescents (table 3). The largest burden per capita (figure 4) was in Chad (21 278 DALYs per 100 000 population), Central African Republic (16 202 DALYs per 100 000), Niger (14 883 DALYs per 100 000), and Nigeria (11 323 DALYs per 100 000). Most countries (187 [92%] of 204) saw reductions in enteric disease burden for children and adolescents between 1990 and 2019 (appendix p 22–26), with the greatest reductions seen in Equatorial Guinea (a decrease of 3·3% per year) and Nicaragua (a decrease of 3·2% per year). Among specific groups, boys aged 5–14 years in Zimbabwe (an increase of 2·1% per year, 95% UI –0·13 to 6·0), and boys aged 15–24 years in Puerto Rico (an increase of 1·7% per year) showed the greatest increases.

In 2019, there were a total of 64·7 million DALYs caused by LRTIs among children and adolescents (table 2; appendix pp 18, 22–26). Similar to enteric infections, the burden of LRTIs was greatest in settings of low and low-middle sociodemographic development (54·4 million DALYs) and in children younger than 5 years globally (59·2 million DALYs, 95% UI 48·5 to 72·7). Nigeria, India, and Pakistan accounted for the largest cause-specific burden (44·2% of the global burden of LRTIs for 0–24 year olds), although it is noteworthy that the percentage burden in Nigeria and Pakistan is greater for enteric infection compared with LRTI (table 3). Countries with the largest per-capita burden (figure 4) of LRTIs were Chad (11 090 DALYs per 100 000 population), Burkina Faso (10 277 per 100 000), and Somalia (9708 per 100 000). In six countries across Asia and Europe, more than half of the disease burden for children and adolescents was accounted for by LRTIs (Azerbaijan [65%], Cambodia [51%], Romania [51%], Tajikistan [56%], Turkmenistan [68%], and Uzbekistan [73%]). With the exception of Dominica (with an increase of 0·1% per year), all countries showed a decline in LRTI-related DALYs between 1990 and 2019 (appendix p 22–26), with the greatest being in Türkiye and Equatorial Guinea (both with declines of 3·2% per year). However, increases over time were seen within specific groups, with the greatest increases among adolescent males aged 15–24 years in Argentina (an increase of 2·9% per year), Sao Tome and Principe (increase of 3·0% per year), and Ukraine (increase of 2·9% per year). Given that enteric disease and LRTIs are the focus of combined intervention such as the GAPPD, the relationship between these two diseases at a country level is detailed in the appendix (p 57). Overall, there was a strong relationship between these two diseases (R^2^ 0·7).

There were 38·3 million DALYs in 2019 caused by malaria across 89 countries (115 countries had no malaria burden; table 2, appendix pp 18, 22–26). 299 052 malaria deaths, representing 70·0% of all malaria deaths globally in those aged 0–24 years, occurred in settings of low sociodemographic development (appendix p 15). Although global disease burden from malaria was highest in children younger than 5 years, with 31·6 million DALYs (95% UI 15·1 to 55·3) and 356 363 deaths (95% UI 169 469 to 630 387), there were 6·7 million DALYs and 71 106 deaths among children and adolescents aged 5–24 years in 2019. Nigeria alone accounted for 26·7% of the malaria burden among children and adolescents globally (table 3), with the highest per-capita burden (figure 4) found in Burkina Faso (11 083 DALYs per 100 000 population), Niger (9827 DALYs per 100 000), and Sierra Leone (13 267 DALYs per 100 000). Most countries demonstrated decreasing burden of malaria (appendix pp 22–56). Within countries with low sociodemographic development, the greatest rate of malaria DALY change was observed in Nepal and Bhutan (a decrease in burden of 3·3% per year). However, notable increases in malaria burden (DALYs) were observed in countries with low-to-middle sociodemographic development, such as North Korea (increase of 196·0% per year) and Cabo Verde (increase of 23·1% per year).

In 2019 there were 10·3 million DALYs caused by tuberculosis among children and adolescents (table 2; appendix pp 18, 22–26), most from drug-susceptible tuberculosis (appendix p 79). Tuberculosis-related mortality and DALYs were greatest in children younger than 5 years (globally, 4·6 million DALYs, 95% UI 3·6 to 5·7, and 50 163 deaths, 95% UI 39 248 to 63 385), but incidence and disability from tuberculosis, as measured by YLDs, increased mostly after the age of 14 years. India, Nigeria, and Pakistan together accounted for 38% of the global tuberculosis burden (table 3) across the developmental window (similar to enteric infections and LRTIs). The largest per-capita burden (figure 4) was in the Central African Republic (5159 DALYs per 100 000 population), Chad (1631 DALYs per 100 000), Lesotho (2380 DALYs per 100 000), and Somalia (2478 DALYs per 100 000). Most countries showed decreasing tuberculosis burden between 1990 and 2019 (appendix pp 22–26), except for Ukraine (increase of 3·4% per year) and Zimbabwe (increase of 0·5% per year). These increases were largely driven by male adolescents aged 15–24 years in Ukraine (increase in males of 7·0% and increase in females of 3·8%) and in Zimbabwe (increase in males of 1·7% and increase in females of 0·8%).

In 2019, there were 138 949 deaths and about 11·3 million DALYs caused by HIV and AIDS among children and adolescents (table 2; appendix pp 16, 18, 27–56), predominantly in settings of low-to-middle sociodemographic development, where 11·0 million DALYs were recorded, accounting for 97% of the global HIV burden. HIV burden varied substantially by age and sex (appendix pp 17, 18); it was high among children younger than 5 years (4·3 million DALYs, 95% UI 3·4 to 5·4, and 48 928 deaths, 95% UI 38 663 to 61 262), lower during childhood 5–9 years (1·1 million DALYs, 95% UI 889333 to 1299952, and 12 379 deaths, 95% UI 10049 to 15024), but higher again during adolescence, such that adolescents aged 15–24 years accounted for 4·7 million DALYs and 62 529 deaths. Mozambique, Nigeria, and South Africa together accounted for 34·5% of all HIV burden across the developmental window (table 3), with the largest burden of HIV and AIDS per capita (figure 4) in Eswatini (6369 DALYs per 100 000 population), Lesotho (8907 DALYs per 100 000), and Mozambique (8934 DALYs per 100 000). There were also important sex differences in burden. Countries with the greatest HIV burden for females aged 15–24 years included Lesotho (15 826 DALYs per 100 000 population *vs* 7283 per 100 000 for males), Mozambique (12 503 per 100 000 *vs* 6535 per 100 000 for males), Eswatini (10 992 per 100 000 *vs* 5383 per 100 000 for males), and South Africa (10 253 per 100 000 *vs* 4688 per 100 000 for males). Over time there has been an increased HIV burden in many settings, with HIV burden only declining in 47 (23%) of 204 countries for children and adolescents aged 5–14 years and 65 (32%) of 204 locations for adolescents aged 15–24 years.

The HIV MIR for children younger than 5 years, adolescents aged 15–19 years, and adolescents aged 20–24 years, and the MIR at a country level are shown in the appendix (pp 84–86). The highest MIRs were observed for adolescents aged 15–19 years and mostly for males in settings of low sociodemographic development (MIR >3 in Burkina Faso, Burundi, Côte d’Ivoire, Ethiopia, Eritrea, Somalia, and Togo), with females aged 15–19 years in Syria having an MIR of 32.

## Discussion

Much remains to be done to reduce the 3 million deaths each year from communicable diseases among children and adolescents globally, approximately one death every 10 sec. Our analysis supports a continued focus on mortality reduction among children under 5 years in settings of low sociodemographic development, with a continued focus on gastroenteritis, pneumonia, and malaria.^4^, ^31^ However, policy and programming actions need to be inclusive of older children and adolescents, who accounted for 647 168 deaths from communicable diseases in 2019. Within this action, we also need to shift our focus to other diseases, including HIV and tuberculosis; the marked increases in deaths in older children and adolescents infected with HIV in some settings are at odds with overall reductions in communicable diseases across the developmental window. We also need to look beyond mortality reduction and focus on morbidity reduction; the 30 million years of healthy life lost to disability in 2019 among children and adolescents signifies an opportunity for health gain; this estimated burden does not include effects on education or social engagement, and as such, effects on human capital will be even greater. This reframing also brings into scope the substantial burden of disability related to communicable diseases in countries of high and high-middle sociodemographic development (8·9 million DALYs and 4·0 million YLDs in 2019 alone), often at the margins of communicable disease control.

This analysis documents the substantial unmet needs in communicable disease control before the COVID-19 pandemic. These findings highlight the need for health systems, particularly in settings of low sociodemographic development where disease burden is focussed, to continue to build capacity to respond to communicable diseases across the life course. Excess mortality-to-incidence ratios for HIV, especially for male adolescents in settings of low sociodemographic development, suggests barriers (supply or demand) to quality health care. The findings also suggest the need to strengthen prevention. Required preventive efforts include established interventions, such as immunisation (the large number of vaccine-preventable deaths suggests incomplete coverage), but also investment in broader approaches that address social determinants. For example, the excess burden of HIV among female individuals in some settings suggests harmful gender norms that might drive differential risk exposure (eg, intimate partner violence),^32^ or limit access to quality health care; these same gender norms might be driving the excess mortality-to-incidence ratio for male adolescents.^33^ The findings also highlight the need for communicable disease-focussed programme policies to be inclusive of older children and adolescents. As such, although the replenishment of The Global Fund is welcomed, these resources need to stretch further, and especially if we are to extend our focus while also maintaining efforts where progress has been made.^34^

To our knowledge, this study is the first systematic analysis of all causes of communicable-disease morbidity and mortality across the developmental window. Available estimates of diarrhoea and pneumonia have been largely limited to children younger than 5 years and focussed on mortality.^35^, ^36^, ^37^ Estimates of malaria and tuberculosis have typically not reported disaggregated data for adolescents,^38^, ^39^ and global data coverage for HIV in adolescents remains limited,^33^, ^40^ but is improving. Our HIV results replicate, yet extend, previously published GBD 2019 incidence and DALY data disaggregated for adolescents aged 10–14 years, 15–19 years, and 20–24 years.^41^ We also extend upon currently available HIV data from UNAIDS that are limited to incidence and mortality.^20^ In short, existing reporting frameworks do not consistently disaggregate data for children and adolescents,^42^ focus on conditions in isolation, or are limited to measures of mortality. This incomplete reporting is reflected in key data dashboards, including Countdown to 2030^43^ (dependent on available primary data), and means that there are important areas of data and knowledge scarcity in policy and programming. As an example, the inter-UN agency OneHealth tool, developed to inform national strategical planning and resource allocation, does not model interventions for diarrhoea and pneumonia beyond the age of 5 years.^44^

Our analysis, which explored all causes of communicable diseases for children and adolescents across the globe, identified some clear targets for action. Five cause groups (enteric infection, LRTIs, malaria, tuberculosis, and HIV) account for more than two-thirds of the burden from communicable diseases across the developmental window. There are also some countries that contribute the greatest burden of these conditions, allowing for targeted actions. India, Nigeria, and Pakistan together account for 47·7% of disease burden related to enteric infections among children and adolescents, 44·2% of lower-respiratory-tract infections, and 37·8% of tuberculosis cases. For tuberculosis, these three countries are identified as priority countries in the WHO Global Tuberculosis Report,^45^ but countries such as Chad and Somalia that we identified as having an excess tuberculosis burden for children and adolescents were not included. For malaria, we found that the Democratic Republic of the Congo, Nigeria, and Uganda together account for 42·9% of the malaria burden among children and adolescents, consistent with priority countries in the WHO World Malaria Report.^46^ For HIV, just six sub-Saharan countries (Ethiopia, Kenya, Mozambique, Nigeria, South Africa, and Zambia) contribute to more than half of the global HIV burden for children and adolescents. These findings can help inform where efforts can be focussed, but not at the expense of children and adolescents in other settings, and not at the expense of opportunities to tackle morbidity. In this regard, it is important to also keep in scope the diseases for which the overall burden might be small, but for which the incidence has increased over time (including STIs, rheumatic heart disease, and neglected tropical diseases), because they pose future threats.

We identify that HIV needs to be a particular priority for global health action. Our trend analysis (annualised change over the past 30-year period and less sensitive to recent improvements as reported elsewhere)^41^, ^47^ showed that although incidence has declined, mortality and burden have increased over time for older children and adolescents. These findings probably reflect the success of Prevention of Parent to Child Transmission interventions and early antiretroviral therapy on improved survival in young children, but unmet health-care needs in older children and adolescents living with HIV. For example, we found that male adolescents in Burkina Faso, Burundi, Côte d’Ivoire, Ethiopia, Eritrea, Somalia, and Togo and female adolescents in Syria have an MIR higher than 3, substantially greater than other age groups and greater than the global all-age average of 1·6 as reported by UNAIDS. As such, accessible and responsive health care for adolescents living with HIV must be prioritised along with efforts to prevent HIV transmission and acquisition. High-quality subnational data are central to this endeavour, including data on the mode and timing of HIV acquisition.

Our analysis provides estimates up to 2019, and there is no doubt that the COVID-19 pandemic has radically shifted the landscape for communicable disease control. COVID-19 vaccine hesitancy and disruptions to education and primary care services pose real risks to preventive and promotive interventions for communicable disease.^16^, ^17^ However, the COVID-19 pandemic has also highlighted the need to address social inequity and has highlighted interventions (decreasing social contact when unwell, hand sanitation, and interventions to improve air quality)^48^ that might favourably affect broader communicable disease control.^49^, ^50^, ^51^ There are additional threats that will probably affect communicable disease control. The first is climate change, which increases the incidence and burden associated with numerous communicable diseases, particularly malaria and enteric infections.^52^ Global warming impacts the built environment and natural habitats, causing expansion in the range and movement of wildlife vectors present in populated areas. In response, proven and effective tools to fight malaria will need to be introduced to new areas. The second is population growth, with the global population forecast to peak in 2064.^53^ In 2100 it is forecasted that the majority of the world's population (including children and adolescents) will live in countries of low and low-middle sociodemographic development (eg, Democratic Republic of the Congo, India, and Nigeria),^53^ settings that have an excess burden of communicable diseases. The third is an increasing demand on the shrinking global health budget. Mental health and non-communicable diseases, long neglected, are increasingly included within global health policy, and rightly so; however, these investments must not displace the required efforts to address communicable diseases.

To maximise data coverage and ensure comparability across locations and over time we used modelled data from the GBD 2019 Study. The disease models employed within the GBD 2019 Study are robust for communicable diseases, and a particular strength is that they harmonise what are often disparate (and sometimes conflicting) epidemiological surveillance data.^29^ Indeed, burden of disease data are increasingly being used in global health, including in the UNICEF adolescent health dashboard.

In these analyses we extended the definition of communicable diseases to include all communicable diseases and their direct sequalae, as modelled in the GBD 2019 Study, resulting in 83 million DALYs in addition to the 420 million DALYs traditionally reported as communicable diseases in GBD 2019. There are also some important limitations associated with using GBD data. Notably, the quality of primary data for communicable diseases is dependent on diagnostic accuracy and population-based surveillance; the burden of diseases such as tuberculosis, STIs, rheumatic heart diseases, and neglected tropical diseases might be underestimated.^54^ Data are also limited in settings of low sociodemographic development (in which burden is greatest) and for older children and adolescents; however, we detailed UIs for each cause, and these give some indication of where the data need to be strengthened (appendix p 87–167). Historical data are also limited in quality, and these limitations might have affected our trend analysis. Cause of death data might also underestimate the contribution of some communicable diseases; for example, deaths among people with HIV might be caused by other causes such as tuberculosis. Within GBD, estimates of morbidity are dependent on disease weights, which are not age or gender specific, and do not include educational and social burdens, which are especially relevant for children and adolescents. GBD also does not include the lifelong or intergenerational effects of disease, and so the true burden might be underestimated. However, these modelled estimates do provide guidance on where the burden of disease is and can inform current efforts to strengthen measurement and reporting of child and adolescent health globally.^55^, ^56^

Following the COVID-19 pandemic, communicable disease control among children and adolescents must be central to efforts ensuring sustainable development.^21^ Our findings support the continued focus of policy and action on diarrhoea, pneumonia, and malaria, and on young children. However, widening the scope to include older children and adolescents, extending the disease focus to include tuberculosis and HIV, and investing in actions to reduce morbidity and mortality are needed to ensure that children and adolescents not only survive through this crucial period of development, but thrive and realise their full potential.

## Data sharing

All data used in this analysis are available at http://ghdx.healthdata.org/gbd-results-tool.

Correspondence to: Prof Peter Azzopardi, Centre for Adolescent Health, Murdoch Children's Research Institute, University of Melbourne, Melbourne, VIC 3004, Australia peter.azzopardi@mcri.edu.au

## Declaration of interests

AKD reports payment or honoraria for lectures, presentations, speakers bureaus; manuscript writing or educational events from speakers bureaus, Stryker, Integra, and Safe (orthopedics); leadership or fiduciary roles in board, society, committee, or advocacy groups, unpaid with the European Association of Neurosurgical Societies, the Board of Global Neuro Foundation, and the Steering Committee of AO Spine Knowledge Forum Degenerative, outside the submitted work. JJJ reports payment or honoraria for lectures, presentations, speakers bureaus, manuscript writing, or educational events from Novartis and Adamed, outside the submitted work. JAL reports support for the present manuscript from Base Funding UIDB/00511/2020 of the Laboratory for Process Engineering, Environment, Biotechnology, and Energy, funded by national funds through The Foundation for Science and Technology and Ministry of Science, Technology, and Higher Education. A-FAM reports grants or contracts from MilkSafe (a novel pipeline to enrich formula milk using omics technologies), research cofinanced by the European Regional Development Fund of the EU and Greek national funds through the Operational Program Competitiveness, Entrepreneurship and Innovation, under the call Research, Create, Innovate (project code T2EDK-02222), and from ELIDEK (Hellenic Foundation for Research and Innovation, MIMS-860); payment for expert testimony from serving as external peer-reviewer for Fondazione Cariplo, Italy; leadership or fiduciary roles in board, society, committee, or advocacy groups, paid or unpaid as an editorial board member for the *Systemic Reviews* journal, for the *Annals of Epidemiology* journal, and as Associate Editor for *Translational Psychiatry*; stocks in a family winery; and other financial or non-financial support from the BGI group as a scientific officer, outside the submitted work. MJP reports stock or stock options in Health Ecore, Zeist, NL, and PAG BV, outside the submitted work.
