# Supplementary material for: The unfinished agenda of communicable diseases among children and adolescents before the COVID-19 pandemic, 1990–2019: a systematic analysis of the Global Burden of Disease Study 2019
Source: Lancet. 2023 Jul 22;402(10398):313–35. doi: 10.1016/S0140-6736(23)00860-7 (PMC10375221; doi:10.1016/S0140-6736(23)00860-7)
Supplement: Supplementary appendix [file mmc1.pdf]

THE LANCET

Supplementary appendix

This appendix formed part of the original submission and has been peer reviewed. We post it as supplied by the authors.

Supplement to: GBD 2019 Child and Adolescent Communicable Disease Collaborators. The unfinished agenda of communicable diseases among children and adolescents before the COVID-19 pandemic, 1990–2019: a systematic analysis of the Global Burden of Disease Study 2019. *Lancet* 2023; published online June 29. https://doi.org/10.1016/S0140-6736(23)00860-7.

Supplementary appendix

Supplement to: The unfinished agenda of communicable diseases amongst children and adolescents prior to the COVID-19 pandemic: A systematic analysis of the Global Burden of Disease study from 1990 to 2019.

Table/ Figure	Page number
**S1.** Table of country names and SDI classification	1
**S2.** Global Burden of Disease cause list and codes	5
**S3.** Causes of total Years Lived with Disability (YLDs) and Years of Life Lost (YLLs, mortality) as a percentage of total DALYs by age (0-24 years) and reflected by sex, across SDI in 2019	7
**S4.** Age and sex-specific estimates of deaths (all-cause and communicable-disease specific) and DALYs (all-cause and communicable-disease specific), by SDI in 2019.	8
**S5a.** Incidence in 2019, incidence/100,000 in 2019 and change in incidence/100,000 from 1990-2019, in 0-24 year olds by SDI groups, and globally	11
**S5b.** Incidence in 2019, incidence/100,000 in 2019 and change in incidence/100,000 from 1990-2019, for under 5, 5-9, 10-14, 15-19, 20-24, 0-24 year olds	12
**S6a.** Number of YLDs in 2019, YLD /100,000 in 2019 and change in YLDs/100,000 from 1990-2019, in 0-24 year olds by SDI groups	13
**S6b.** Number of YLDs in 2019, YLDs/100,000 in 2019 and change in YLDs/100,000 from 1990-2019, for under 5, 5-9, 10-14, 15-19, 20-24, 0-24 year olds	14
**S7a.** Number of deaths in 2019, deaths/100,000 in 2019 and change in deaths/100,000 from 1990-2019, in 0-24 year olds by SDI groups	15
**S7b.** Number of deaths in 2019, deaths/100,000 in 2019 and change in deaths/100,000 from 1990-2019, for under 5, 5-9, 10-14, 15-19, 20-24, 0-24 year olds	16
**S8a.** Number of DALYs in 2019, DALY /100,000 in 2019 and change in DALYs/100,000 from 1990-2019, in 0-24 year olds by SDI groups	17
**S8b.** Number of DALYs in 2019, DALYs/100,000 in 2019 and change in DALYs/100,000 from 1990-2019, for under 5, 5-9, 10-14, 15-19, 20-24, 0-24 year olds	18
S9. Proportion of communicable disease DALYs and deaths in 2019 attributed by each specific cause, by age, sex and SDI	19
**S10** Communicable disease burden (DALYs) among 0-24-year-olds borne by each age group over time as (A) proportion (B) number.	20
**S11** Deaths among 0-24-year-olds borne by each age group over time as (A) proportion (B) number.	21
**S12. (A)** Communicable disease DALYs by cause for under 5 year olds by sex in 2019, grouped by SDI.	22
**(B)** Percentage change in DALYs/100,000 for each communicable condition for each location for under 5 year olds by sex.	27
**S13. (A)** Communicable disease DALYs by cause for 5-14 year age group by sex in 2019, grouped by SDI.	32
**(B)** Percentage change in DALYs/100,000 for each communicable condition for each location for 5-14 year age group by sex	37
**S14. (A)** Communicable disease DALYs by cause for 15-24 year age group by sex in 2019, grouped by SDI.	42
**(B)** Percentage change in DALYs/100,000 for each communicable condition for each location for 15-24 year age group by sex	47

**S15. (A)** Percentage change in DALYs/100,000 for each communicable condition for each location for 0-24 year old age group by sex.	52
**S16:** Relationship between enteric and lower respiratory tract disease burden for 0-24 year olds by country, 2019.	57
**S17 (1 to 16):** For each communicable condition: Enteric infections, Hepatitis, HIV, Infectious skin conditions, Lower respiratory infections, Malaria, Maternal sepsis and other maternal infections, Meningitis & Encephalitis, Neglected Tropical Diseases, Neonatal sepsis and other neonatal infections, Other unspecified infectious diseases, Rheumatic heart disease, Sexually transmitted infections excluding HIV, Tuberculosis, Upper respiratory infections, Vaccine preventable diseases<br>**Part A:** Incidence, YLD, death and DALY per 100 000 in 2019 for each age group and rate of change in YLD and deaths for each age group from 1990-2019<br>**Part B:** Contribution of individual causes to the 2019 death/100,000 and DALYs/ 100,000 for the each communicable disease group	58
**S18.** HIV mortality to incidence ratio for males and females, aged <5 years, 15-19 and 20-24, in 2019.	84
**S19.** Incidence mortality ratio by location for HIV, for females and males aged less than 5 years, 15 to 19 years and 20 to 24 years	85
**S20 (1 to 10).** Uncertainty ranges for (part A) deaths/100,000 and (part B) DALYs/100,000, for each country, for under 5, 5-9, 10-14, 15-19, 20-24, for:<br>Enteric infections, HIV, Lower respiratory infections, Malaria, Maternal sepsis and other maternal infections, Neonatal sepsis and other neonatal infections, Other unspecified infectious diseases, Rheumatic heart disease, Sexually transmitted infections excluding HIV, and Tuberculosis<br>All UI data is available at: https://vizhub.healthdata.org/gbd-results/	87
**S21.** Author contributions	167

S1: List of the 204 country/ territories and their SDI classification in 2019

Afghanistan	Low SDI
Benin	Low SDI
Burkina Faso	Low SDI
Burundi	Low SDI
Central African Republic	Low SDI
Chad	Low SDI
Côte d'Ivoire	Low SDI
Democratic Republic of the Congo	Low SDI
Eritrea	Low SDI
Ethiopia	Low SDI
Gambia	Low SDI
Guinea	Low SDI
Guinea-Bissau	Low SDI
Haiti	Low SDI
Liberia	Low SDI
Madagascar	Low SDI
Malawi	Low SDI
Mali	Low SDI
Mozambique	Low SDI
Nepal	Low SDI
Niger	Low SDI
Pakistan	Low SDI
Papua New Guinea	Low SDI
Rwanda	Low SDI
Senegal	Low SDI
Sierra Leone	Low SDI
Solomon Islands	Low SDI
Somalia	Low SDI
South Sudan	Low SDI
Togo	Low SDI
Uganda	Low SDI
United Republic of Tanzania	Low SDI
Yemen	Low SDI
Angola	Low-middle SDI
Bangladesh	Low-middle SDI
Belize	Low-middle SDI
Bhutan	Low-middle SDI
Bolivia (Plurinational State of)	Low-middle SDI
Cabo Verde	Low-middle SDI
Cambodia	Low-middle SDI
Cameroon	Low-middle SDI
Comoros	Low-middle SDI
Congo	Low-middle SDI
Democratic People's Republic of Korea	Low-middle SDI
Djibouti	Low-middle SDI
Dominican Republic	Low-middle SDI
El Salvador	Low-middle SDI
Eswatini	Low-middle SDI
Ghana	Low-middle SDI
Guatemala	Low-middle SDI
Honduras	Low-middle SDI

India	Low-middle SDI
Kenya	Low-middle SDI
Kiribati	Low-middle SDI
Kyrgyzstan	Low-middle SDI
Lao People's Democratic Republic	Low-middle SDI
Lesotho	Low-middle SDI
Maldives	Low-middle SDI
Marshall Islands	Low-middle SDI
Mauritania	Low-middle SDI
Micronesia (Federated States of)	Low-middle SDI
Mongolia	Low-middle SDI
Morocco	Low-middle SDI
Myanmar	Low-middle SDI
Nicaragua	Low-middle SDI
Nigeria	Low-middle SDI
Palestine	Low-middle SDI
Sao Tome and Principe	Low-middle SDI
Sudan	Low-middle SDI
Tajikistan	Low-middle SDI
Timor-Leste	Low-middle SDI
Tuvalu	Low-middle SDI
Vanuatu	Low-middle SDI
Venezuela (Bolivarian Republic of)	Low-middle SDI
Zambia	Low-middle SDI
Zimbabwe	Low-middle SDI
Albania	Middle SDI
Algeria	Middle SDI
Armenia	Middle SDI
Azerbaijan	Middle SDI
Botswana	Middle SDI
Brazil	Middle SDI
China	Middle SDI
Colombia	Middle SDI
Costa Rica	Middle SDI
Cuba	Middle SDI
Ecuador	Middle SDI
Egypt	Middle SDI
Equatorial Guinea	Middle SDI
Fiji	Middle SDI
Gabon	Middle SDI
Grenada	Middle SDI
Guyana	Middle SDI
Indonesia	Middle SDI
Iran (Islamic Republic of)	Middle SDI
Iraq	Middle SDI
Jamaica	Middle SDI
Mexico	Middle SDI
Namibia	Middle SDI
Nauru	Middle SDI
Panama	Middle SDI
Paraguay	Middle SDI
Peru	Middle SDI

Philippines	Middle SDI
Saint Lucia	Middle SDI
Saint Vincent and the Grenadines	Middle SDI
Samoa	Middle SDI
South Africa	Middle SDI
Suriname	Middle SDI
Syrian Arab Republic	Middle SDI
Thailand	Middle SDI
Tokelau	Middle SDI
Tonga	Middle SDI
Tunisia	Middle SDI
Turkmenistan	Middle SDI
Uzbekistan	Middle SDI
Viet Nam	Middle SDI
American Samoa	High-middle SDI
Antigua and Barbuda	High-middle SDI
Argentina	High-middle SDI
Bahamas	High-middle SDI
Bahrain	High-middle SDI
Barbados	High-middle SDI
Belarus	High-middle SDI
Bosnia and Herzegovina	High-middle SDI
Bulgaria	High-middle SDI
Chile	High-middle SDI
Cook Islands	High-middle SDI
Croatia	High-middle SDI
Dominica	High-middle SDI
Georgia	High-middle SDI
Greece	High-middle SDI
Greenland	High-middle SDI
Hungary	High-middle SDI
Israel	High-middle SDI
Italy	High-middle SDI
Jordan	High-middle SDI
Kazakhstan	High-middle SDI
Lebanon	High-middle SDI
Libya	High-middle SDI
Malaysia	High-middle SDI
Malta	High-middle SDI
Mauritius	High-middle SDI
Montenegro	High-middle SDI
Niue	High-middle SDI
North Macedonia	High-middle SDI
Northern Mariana Islands	High-middle SDI
Oman	High-middle SDI
Palau	High-middle SDI
Poland	High-middle SDI
Portugal	High-middle SDI
Republic of Moldova	High-middle SDI
Romania	High-middle SDI
Russian Federation	High-middle SDI
Saint Kitts and Nevis	High-middle SDI

Saudi Arabia	High-middle SDI
Serbia	High-middle SDI
Seychelles	High-middle SDI
Spain	High-middle SDI
Sri Lanka	High-middle SDI
Trinidad and Tobago	High-middle SDI
Turkey	High-middle SDI
Ukraine	High-middle SDI
United States Virgin Islands	High-middle SDI
Uruguay	High-middle SDI
Andorra	High SDI
Australia	High SDI
Austria	High SDI
Belgium	High SDI
Bermuda	High SDI
Brunei Darussalam	High SDI
Canada	High SDI
Cyprus	High SDI
Czechia	High SDI
Denmark	High SDI
Estonia	High SDI
Finland	High SDI
France	High SDI
Germany	High SDI
Guam	High SDI
Iceland	High SDI
Ireland	High SDI
Japan	High SDI
Kuwait	High SDI
Latvia	High SDI
Lithuania	High SDI
Luxembourg	High SDI
Monaco	High SDI
Netherlands	High SDI
New Zealand	High SDI
Norway	High SDI
Puerto Rico	High SDI
Qatar	High SDI
Republic of Korea	High SDI
San Marino	High SDI
Singapore	High SDI
Slovakia	High SDI
Slovenia	High SDI
Sweden	High SDI
Switzerland	High SDI
Taiwan (Province of China)	High SDI
United Arab Emirates	High SDI
United Kingdom	High SDI
United States of America	High SDI

S2: Global Burden of disease cause list for Communicable, maternal, neonatal and nutritional diseases with the group allocation for the current paper

GBD disease	GBD code	Communicable group
**Communicable, maternal, neonatal, and nutritional diseases**	**A**	
HIV/AIDS and sexually transmitted infections	A.1	
HIV/AIDS	A.1.1	
HIV/AIDS - Drug-susceptible Tuberculosis	A.1.1.1	HIV
HIV/AIDS - Multidrug-resistant Tuberculosis without extensive drug resistance	A.1.1.2	HIV
HIV/AIDS - Extensively drug-resistant Tuberculosis	A.1.1.3	HIV
HIV/AIDS resulting in other diseases	A.1.1.4	HIV
Sexually transmitted infections excluding HIV	A.1.2	
Syphilis	A.1.2.1	STI
Chlamydial infection	A.1.2.2	STI
Gonococcal infection	A.1.2.3	STI
Trichomoniasis	A.1.2.4	STI
Genital herpes	A.1.2.5	STI
Other sexually transmitted infections	A.1.2.6	STI
Respiratory infections and tuberculosis	A.2	
Tuberculosis	A.2.1	
Latent tuberculosis infection	A.2.1.1	TB
Drug-susceptible tuberculosis	A.2.1.2	TB
Multidrug-resistant tuberculosis without extensive drug resistance	A.2.1.3	TB
Extensively drug-resistant tuberculosis	A.2.1.4	TB
Lower respiratory infections	A.2.2	LRI
Upper respiratory infections	A.2.3	URI
Otitis media	A.2.4	URI
Enteric infections	A.3	
Diarrheal diseases	A.3.1	Enteric infections
Typhoid and paratyphoid	A.3.2	
Typhoid fever	A.3.2.1	Enteric infections
Paratyphoid fever	A.3.2.2	Enteric infections
Invasive Non-typhoidal Salmonella (iNTS)	A.3.3	Enteric infections
Other intestinal infectious diseases	A.3.5	Enteric infections
Neglected tropical diseases and malaria	A.4	
Malaria	A.4.1	Malaria
Chagas disease	A.4.2	NTD
Leishmaniasis	A.4.3	NTD
African trypanosomiasis	A.4.4	NTD
Schistosomiasis	A.4.5	NTD
Cysticercosis	A.4.6	NTD
Cystic echinococcosis	A.4.7	NTD
Lymphatic filariasis	A.4.8	NTD
Onchocerciasis	A.4.9	NTD
Trachoma	A.4.10	NTD
Dengue	A.4.11	NTD
Yellow fever	A.4.12	NTD
Rabies	A.4.13	NTD
Intestinal nematode infections	A.4.14	NTD
Food-borne trematodiases	A.4.15	NTD

Leprosy	A.4.16	NTD
Ebola	A.4.17	NTD
Zika virus	A.4.18	NTD
Guinea worm disease	A.4.19	NTD
Other neglected tropical diseases	A.4.20	NTD
Other infectious diseases	A.5	
Meningitis	A.5.1	Meningitis/Encephalitis
Encephalitis	A.5.2	Meningitis/Encephalitis
Diphtheria	A.5.3	VPD
Whooping cough	A.5.4	VPD
Tetanus	A.5.5	VPD
Measles	A.5.6	VPD
Varicella and herpes zoster	A.5.7	VPD
Acute hepatitis	A.5.8	
Acute hepatitis A	A.5.8.1	Hepatitis
Acute hepatitis B	A.5.8.2	Hepatitis
Acute hepatitis C	A.5.8.3	Hepatitis
Acute hepatitis E	A.5.8.4	Hepatitis
Other unspecified infectious diseases	A.5.9	Other unspecified infectious diseases
Maternal and neonatal disorders	*A.6*	
Maternal sepsis and other maternal infections	A.6.1.2	Maternal sepsis & other infections
Neonatal sepsis and other neonatal infections	A.6.2.3	Neonatal sepsis & other infections
Nutritional deficiencies	*A.7*	
**Non-communicable diseases**	**B**	
Neoplasms	B.1	
Cardiovascular diseases	B.2	
Rheumatic heart disease	B.2.1	
Chronic respiratory diseases	B.3	
Digestive diseases	B.4	
Neurological disorders	B.5	
Mental disorders	B.6	
Substance use disorders	B.7	
Liver cancer due to hepatitis B	B.1.7.1	Hepatitis
Liver cancer due to hepatitis C	B.1.7.2	Hepatitis
Cirrhosis and other chronic liver diseases due to hepatitis B	B.4.1.1	Hepatitis
Cirrhosis and other chronic liver diseases due to hepatitis C	B.4.1.2	Hepatitis
Diabetes and kidney diseases	B.8	
Skin and subcutaneous diseases	B.9	
Bacterial skin diseases	B.9.3	Infectious skin conditions
Scabies	B.9.4	Infectious skin conditions
Fungal skin diseases	B.9.5	Infectious skin conditions
Viral skin diseases	B.9.6	Infectious skin conditions
Sense organ diseases	B.10	
Musculoskeletal disorders	B.11	
Other non-communicable diseases	B.12	
**Injuries**	**C**	
Transport injuries	C.1	
Unintentional injuries	C.2	
Self-harm* and interpersonal violence	C.3	

*Self harm is grouped with NCD and not injury for this paper

S3. Causes of total Years Lived with Disability (YLDs) and Years of Life Lost (YLLs, mortality) as a percentage of total DALYs by age (0-24 years) and reflected by sex, across SDI in 2019.

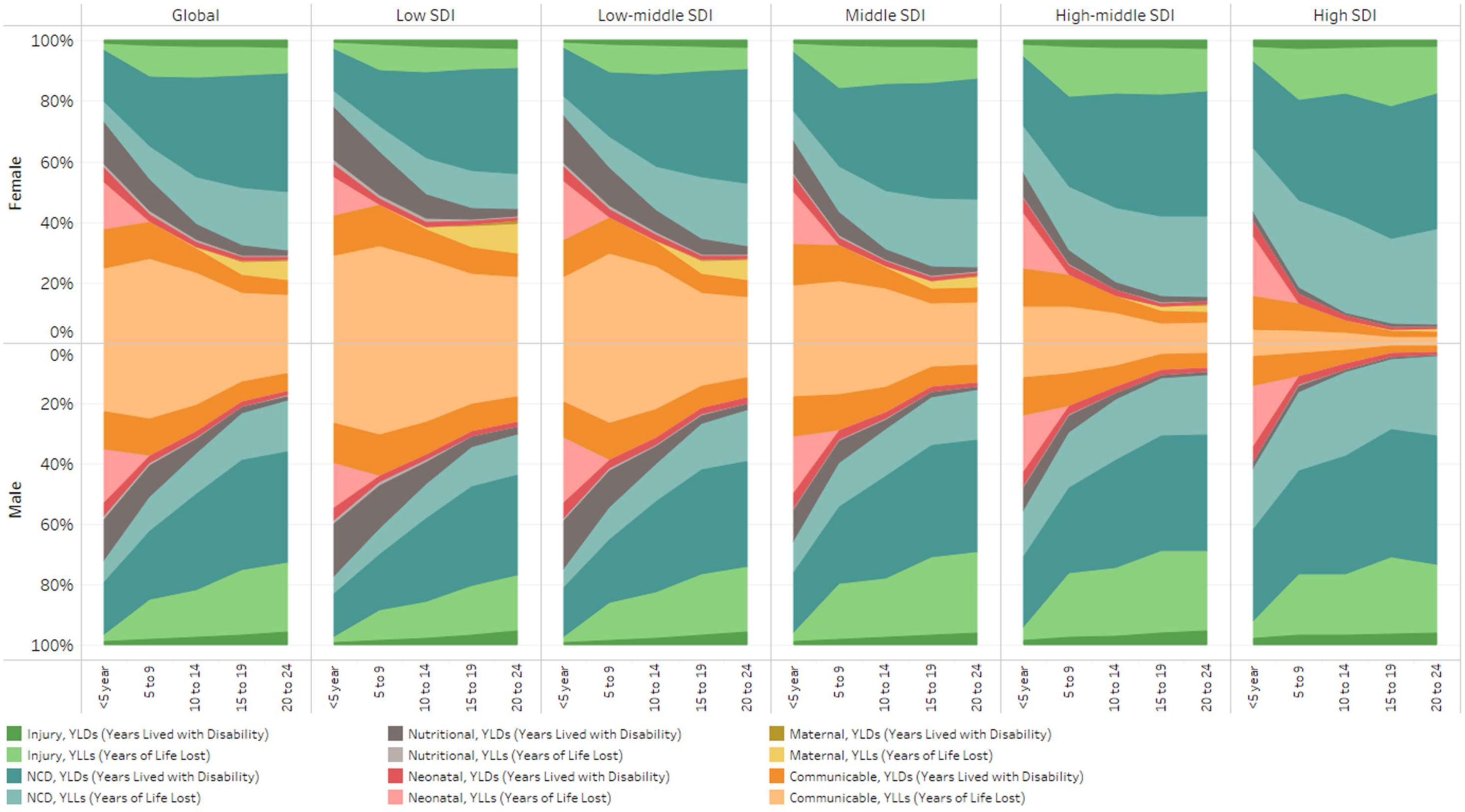

S4. Age and sex-specific estimates of deaths (all-cause and communicable-disease specific) and DALYs (all-cause and communicable-disease specific), by SDI in 2019.

	Age (yrs)	Sex	Population	All cause deaths	Deaths due to communicable disease	Proportion of deaths due to communicable disease (%)	All cause DALYs	DALYs due to communicable disease	Proportion of DALYs due to communicable disease (%)
Low SDI	<5	Female	83,700,856	1,225,678	712,764	58.2	112,155,912	63,558,180	56.7
	<5	Male	87,154,512	1,445,802	772,268	53.4	131,981,608	68,866,531	52.2
	5 to 9	Female	77,689,352	70,355	45,143	64.2	10,116,425	4,890,860	48.3
	5 to 9	Male	80,523,872	89,041	54,629	61.4	11,962,074	5,750,997	48.1
	10 to 14	Female	70,748,832	46,198	25,897	56.1	8,142,353	2,892,205	35.5
	10 to 14	Male	73,058,320	59,902	31,575	52.7	8,972,512	3,362,453	37.5
	15 to 19	Female	61,503,536	61,558	28,429	46.2	9,340,045	2,899,571	31.0
	15 to 19	Male	62,583,668	86,489	35,429	41.0	10,219,094	3,246,351	31.8
	20 to 24	Female	52,213,808	72,856	32,198	44.2	9,862,753	2,913,059	29.5
	20 to 24	Male	51,377,092	101,152	36,473	36.1	10,418,173	3,049,982	29.3
	≥25	Female	216,656,448	2,070,177	636,248	30.7	92,054,952	23,523,064	25.6
	≥25	Male	211,466,352	2,418,497	773,816	32.0	97,953,504	28,583,548	29.2
	0 to 24	***Both***	**700,553,840**	**3,259,029**	**1,774,805**	**54.5**	**323,170,950**	**161,430,189**	**50.0**
	0 to 24	***Female***	**345,856,384**	**1,476,645**	**844,431**	**57.2**	**149,617,488**	**77,153,876**	**51.6**
	0 to 24	***Male***	**354,697,464**	**1,782,384**	**930,373**	**52.2**	**173,553,461**	**84,276,313**	**48.6**
Low-middle SDI	<5	Female	83,335,704	692,997	305,715	44.1	64,724,548	27,711,517	42.8
	<5	Male	88,963,320	792,339	314,554	39.7	73,915,512	28,565,644	38.6
	5 to 9	Female	84,623,288	57,546	34,126	59.3	8,664,503	3,753,306	43.3
	5 to 9	Male	89,890,128	64,778	34,580	53.4	9,631,233	3,876,891	40.3
	10 to 14	Female	85,910,840	46,120	23,483	50.9	8,651,091	2,647,021	30.6
	10 to 14	Male	90,825,264	57,341	25,274	44.1	9,142,098	2,826,780	30.9
	15 to 19	Female	84,247,840	74,141	25,166	33.9	11,801,465	2,598,388	22.0
	15 to 19	Male	87,563,288	92,876	26,802	28.9	11,716,270	2,662,990	22.7
	20 to 24	Female	80,610,000	96,795	29,992	31.0	13,907,715	2,793,074	20.1
	20 to 24	Male	81,268,272	133,584	31,347	23.5	14,273,909	2,800,729	19.6
	≥25	Female	458,777,088	4,703,324	984,382	20.9	188,698,720	31,280,331	16.6
	≥25	Male	447,967,392	5,552,428	1,088,296	19.6	206,863,072	37,353,238	18.1
	0 to 24	***Both***	**857,237,968**	**2,108,517**	**851,039**	**40.4**	**226,428,330**	**80,236,339**	**35.4**
	0 to 24	***Female***	**418,727,672**	**967,600**	**418,482**	**43.2**	**107,749,322**	**39,503,305**	**36.7**

	0 to 24	***Male***	**438,510,272**	**1,140,918**	**432,557**	**37.9**	**118,679,022**	**40,733,034**	**34.3**
Middle SDI	<5	Female	88,195,816	303,539	117,293	38.6	29,673,254	11,090,109	37.4
	<5	Male	95,995,264	381,511	136,282	35.7	36,911,780	12,848,279	34.8
	5 to 9	Female	88,532,296	30,438	12,511	41.1	5,814,004	1,809,415	31.1
	5 to 9	Male	96,635,320	42,417	14,526	34.2	7,157,773	2,085,769	29.1
	10 to 14	Female	88,071,960	27,497	10,015	36.4	6,794,345	1,432,080	21.1
	10 to 14	Male	96,168,424	38,631	11,358	29.4	7,419,528	1,618,488	21.8
	15 to 19	Female	88,295,440	43,017	11,379	26.5	9,371,077	1,461,221	15.6
	15 to 19	Male	94,656,056	81,256	13,300	16.4	11,000,834	1,624,417	14.8
	20 to 24	Female	89,675,616	59,468	16,422	27.6	11,597,764	1,817,493	15.7
	20 to 24	Male	92,668,576	125,687	18,197	14.5	14,215,469	1,904,895	13.4
	≥25	Female	747,909,120	6,451,820	704,403	10.9	250,728,128	23,973,891	9.6
	≥25	Male	729,761,920	8,313,147	970,822	11.7	295,376,864	34,482,370	11.7
	0 to 24	***Both***	**918,894,784**	**1,133,462**	**361,284**	**31.9**	**139,955,831**	**37,692,165**	**26.9**
	0 to 24	***Female***	**442,771,128**	**463,959**	**167,620**	**36.1**	**63,250,444**	**17,610,318**	**27.8**
	0 to 24	***Male***	**476,123,640**	**669,503**	**193,664**	**28.9**	**76,705,384**	**20,081,847**	**26.2**
High-middle SDI	<5	Female	39,467,708	66,277	16,285	24.6	6,987,966	1,723,311	24.7
	<5	Male	43,167,628	83,476	19,295	23.1	8,641,924	2,021,288	23.4
	5 to 9	Female	39,132,164	7,001	1,695	24.2	1,865,445	417,461	22.4
	5 to 9	Male	42,695,556	10,694	2,170	20.3	2,317,001	488,878	21.1
	10 to 14	Female	38,573,080	6,650	1,327	20.0	2,489,718	332,428	13.4
	10 to 14	Male	42,182,176	10,333	1,598	15.5	2,651,519	377,259	14.2
	15 to 19	Female	39,707,344	11,742	1,575	13.4	3,667,815	341,546	9.3
	15 to 19	Male	43,040,240	27,188	2,121	7.8	4,313,348	391,446	9.1
	20 to 24	Female	43,350,672	16,235	2,222	13.7	4,750,821	412,725	8.7
	20 to 24	Male	46,897,140	48,333	3,563	7.4	6,194,820	512,729	8.3
	≥25	Female	515,667,136	5,272,776	279,979	5.3	172,418,272	8,548,008	5.0
	≥25	Male	496,523,008	6,029,484	407,924	6.8	199,688,416	14,042,752	7.0
	0 to 24	***Both***	**418,213,712**	**287,928**	**51,852**	**18.0**	**43,880,376**	**7,019,070**	**16.0**
	0 to 24	***Female***	**200,230,968**	**107,905**	**23,105**	**21.4**	**19,761,764**	**3,227,471**	**16.3**
	0 to 24	***Male***	**217,982,740**	**180,023**	**28,747**	**16.0**	**24,118,611**	**3,791,599**	**15.7**
High SDI	<5	Female	25,536,442	21,592	1,960	9.1	2,557,155	316,947	12.4
	<5	Male	26,900,140	27,018	2,429	9.0	3,104,994	356,435	11.5
	5 to 9	Female	26,577,084	2,259	189	8.4	1,062,152	176,611	16.6
	5 to 9	Male	27,993,492	2,957	206	7.0	1,183,570	161,220	13.6
	10 to 14	Female	27,356,536	2,645	182	6.9	1,760,830	144,024	8.2

10 to 14	Male	28,898,388	3,759	192	5.1	1,698,805	137,408	8.1
15 to 19	Female	27,819,738	6,393	255	4.0	2,989,746	131,512	4.4
15 to 19	Male	29,745,708	14,305	286	2.0	3,094,497	128,101	4.1
20 to 24	Female	29,745,528	9,614	422	4.4	4,037,312	145,609	3.6
20 to 24	Male	31,972,158	28,207	546	1.9	4,606,728	147,446	3.2
≥25	Female	370,639,488	4,273,239	298,855	7.0	132,660,552	5,438,734	4.1
≥25	Male	360,200,096	4,502,127	346,468	7.7	140,535,808	7,540,147	5.4
0 to 24	***Both***	**282,545,216**	**118,748**	**6,669**	**5.6**	**26,095,788**	**1,845,313**	**7.1**
0 to 24	***Female***	**137,035,328**	**42,504**	**3,008**	**7.1**	**12,407,194**	**914,703**	**7.4**
0 to 24	***Male***	**145,509,886**	**76,244**	**3,660**	**4.8**	**13,688,595**	**930,610**	**6.8**

S5a. Incidence in 2019, incidence/100,000 in 2019 and change in incidence/100,000 from 1990-2019, in 0-24 year olds by SDI groups, and globally

		Low		Low-Mid		Middle		High-mid		High		Global	
Population in 2019	F	345,856,384		418,727,616		442,771,136		200,230,976		137,035,328		1,545,586,048	
	M	354,697,440		438,510,272		476,123,648		217,982,720		145,509,888		1,633,832,448	
		Number incidence	Incidence/100,000 (change/pa)	Number incidence	Incidence/100,000 (change/pa)	Number incidence	Incidence/100,000 (change/pa)	Number incidence	Incidence/100,000 (change/pa)	Number incidence	Incidence/100,00 0 (change/pa)	Number incidence	Incidence/100, 000 (change/pa)
Enteric infections	F	410485860	118,686.8(-0.7%)	437648452	104,518.6(-1.0%)	323963560	73,167.3(-0.2%)	141422064	70,629.5(-0.1%)	57513069	41,969.5(0.3%)	1371894096	88,762.1(-0.4%)
	M	425482376	119,956.4(-0.5%)	452228320	103,128.3(-0.8%)	356135872	74,799.0(-0.2%)	155834850	71,489.5(-0.1%)	67368094	46,297.9(0.1%)	1458042144	89,240.6(-0.2%)
HIV/AIDS	F	130214.1341	37.6(-2.6%)	87687.04414	20.9(-1.9%)	103129.875	23.3(5.6%)	13942.80518	7.0(3.8%)	5092.974304	3.7(-0.2%)	340332.1821	22.0(-1.3%)
	M	61767.72751	17.4(-2.4%)	46827.53643	10.7(-1.3%)	75744.5332	15.9(4.5%)	16267.4209	7.5(1.2%)	8773.362045	6.0(-1.2%)	209547.2337	12.8(-0.7%)
Hepatitis	F	25048958	7,242.6(-0.6%)	24207298	5,781.2(-0.7%)	22099622	4,991.2(-0.9%)	8266557	4,128.5(-0.8%)	2965943.75	2,164.4(-0.5%)	82659384	5,348.1(-0.6%)
	M	27081628	7,635.1(-0.6%)	26876016	6,128.9(-0.7%)	24609814	5,168.8(-1.0%)	9336816	4,283.3(-1.0%)	3250954.75	2,234.2(-0.7%)	91232144	5,583.9(-0.7%)
Infectious skin conditions	F	183383680	53,023.1(-0.1%)	195174784	46,611.4(-0.1%)	174206784	39,344.7(0.1%)	63339088	31,633.0(0.0%)	36262604	26,462.2(-0.2%)	652766528	42,234.2(0.2%)
	M	202315632	57,038.9(-0.1%)	211057456	48,130.6(-0.0%)	198402976	41,670.5(0.1%)	74957296	34,386.8(0.0%)	42337780	29,096.2(-0.2%)	729522304	44,651.0(0.2%)
Lower respiratory infections	F	20642821	5,968.6(-1.5%)	19941027.5	4,762.3(-1.6%)	18908052.5	4,270.4(-1.5%)	6759061.5	3,375.6(-1.3%)	3688918.5	2,691.9(-0.4%)	69983776	4,528.0(-1.3%)
	M	21858262.25	6,162.5(-1.4%)	23885605.75	5,447.0(-1.4%)	22248790.5	4,672.9(-1.3%)	7747938.375	3,554.4(-1.3%)	4015144.156	2,759.4(-0.4%)	79803745	4,884.5(-1.2%)
Malaria	F	61718816.5	17,845.2(-1.5%)	23321358.25	5,569.6(-1.4%)	8793625.063	1,986.0(-0.7%)	599896.4141	299.6(-0.4%)	180.9182262	0.1(-3.2%)	94470303.5	6,112.3(-0.5%)
	M	62341386	17,575.9(-1.5%)	23504971	5,360.2(-1.4%)	8903652.063	1,870.0(-0.7%)	588742.2734	270.1(-0.7%)	215.1802807	0.1(-3.2%)	95377068.5	5,837.6(-0.6%)
Maternal sepsis & other maternal infections	F	3169880.316	916.5(-0.9%)	3081629.168	736.0(-1.2%)	2408292.688	543.9(-1.4%)	859718.1519	429.4(-1.7%)	428803.4849	312.9(-1.7%)	9955345.441	644.1(-1.2%)
	M	0	0.0(.%)	0	0.0(.%)	0	0.0(.%)	0	0.0(.%)	0	0.0(.%)	0	0.0(.%)
Meningitis & Encephalitis	F	493144.1563	142.6(-1.6%)	374609.8125	89.5(-1.7%)	238338.9531	53.8(-1.3%)	78698.92969	39.3(-1.2%)	27716.25391	20.2(-1.5%)	1213102.125	78.5(-1.2%)
	M	570901.3125	161.0(-1.7%)	408551.4688	93.2(-1.7%)	280246.125	58.9(-1.4%)	98238.02344	45.1(-1.3%)	33071.82422	22.7(-1.5%)	1391708.375	85.2(-1.3%)
Neglected Tropical diseases	F	2336491.5	675.6(-0.9%)	5869181	1,401.7(-0.1%)	4241308.5	957.9(1.8%)	1332857.375	665.7(3.9%)	141284.6406	103.1(1.0%)	13933122	901.5(0.7%)
	M	2194134.5	618.6(-1.1%)	5694843	1,298.7(-0.1%)	4234562.5	889.4(1.9%)	1432657.5	657.2(4.5%)	145012.1719	99.7(1.3%)	13712658	839.3(0.7%)
Neonatal sepsis & other neonatal infections	F	738003.8125	213.4(-0.4%)	763512.0625	182.3(-0.2%)	845412	190.9(0.2%)	377716.0938	188.6(0.6%)	64790.00391	47.3(-0.2%)	2970031.5	192.2(0.1%)
	M	772716.125	217.9(-0.5%)	828348.625	188.9(-0.4%)	1030298.25	216.4(-0.1%)	426348.3125	195.6(0.2%)	77281.375	53.1(-0.1%)	3340035.5	204.4(-0.2%)
Other unspecified infectious diseases	F	0	0.0(.%)	0	0.0(.%)	0	0.0(.%)	0	0.0(.%)	0	0.0(.%)	0	0.0(.%)
	M	0	0.0(.%)	0	0.0(.%)	0	0.0(.%)	0	0.0(.%)	0	0.0(.%)	0	0.0(.%)
Rheumatic heart disease	F	364613.2949	105.4(0.5%)	358265.9863	85.6(0.6%)	233122.7422	52.7(0.1%)	47479.13672	23.7(0.2%)	1191.652924	0.9(0.2%)	1005337.063	65.0(0.9%)
	M	332141.041	93.6(0.5%)	291587.9492	66.5(0.6%)	218305.1895	45.9(0.0%)	44370.41016	20.4(0.0%)	1048.014206	0.7(0.1%)	888044.0859	54.4(0.9%)
Sexually transmitted infections excluding HIV	F	18139102.13	5,244.7(0.2%)	17355121.19	4,144.7(0.8%)	20397162.67	4,606.7(-0.0%)	8452480.392	4,221.4(-0.1%)	3709436.341	2,706.9(-0.3%)	68115664.53	4,407.1(0.2%)
	M	13409648.43	3,780.6(0.3%)	17085405.66	3,896.2(0.5%)	20783487.42	4,365.1(0.1%)	8957064.403	4,109.1(0.1%)	3586877.83	2,465.0(-0.1%)	63868825.96	3,909.1(0.2%)
Tuberculosis	F	419828.4922	121.4(-1.4%)	458456.2539	109.5(-1.6%)	310747.4023	70.2(-1.6%)	52932.72925	26.4(-1.8%)	8002.725037	5.8(-2.1%)	1250609.359	80.9(-1.4%)
	M	350942.5586	98.9(-1.1%)	354039.5449	80.7(-1.2%)	286913.9238	60.3(-1.4%)	61901.18799	28.4(-1.5%)	8655.449127	5.9(-1.8%)	1062973.82	65.1(-1.1%)
Upper respiratory infections	F	848377664	245,297.7(-0.1%)	1067516288	254,942.9(-0.0%)	1166728832	263,506.1(0.0%)	533899904	266,642.0(0.1%)	486919840	355,324.3(0.0%)	4106167296	265,670.6(-0.0%
	M	885710208	249,708.7(-0.1%)	1153033088	262,943.2(-0.0%)	1302695936	273,604.5(-0.0%)	594819648	272,874.7(0.0%)	506252640	347,916.3(0.0%)	4445416960	272,085.3(-0.1%
Vaccine Preventable disease	F	15159104	4,383.1(-2.0%)	12666785	3,025.1(-2.2%)	12237409	2,763.8(-1.7%)	4881399	2,437.9(-1.3%)	2978366.25	2,173.4(-0.6%)	47956196	3,102.8(-1.7%)
	M	14791477	4,170.2(-2.0%)	12787343	2,916.1(-2.2%)	12659454	2,658.9(-1.7%)	5155952	2,365.3(-1.3%)	3037466.25	2,087.5(-0.6%)	48464560	2,966.3(-1.7%)
Total communicable	F	1590608181	459,904.3(-0.4%)	1808824455	431,981.2(-0.4%)	1755715399	396,528.9(-0.1%)	770383795.5	384,747.6(-0.0%)	594715240.5	433,986.8(0.0%)	6524681124	422,149.3(-0.2%
	M	1657273221	467,235.8(-0.4%)	1928082404	439,689.2(-0.3%)	1952566053	410,096.4(-0.1%)	859478089.9	394,287.3(-0.0%)	630123014.4	433,044.8(-0.0%)	7032332718	430,419.5(-0.1%

S5b. Incidence in 2019, incidence/100,000 in 2019 and change in incidence/100,000 from 1990-2019, for under 5, 5-9, 10-14, 15-19, 20-24, 0-24 year olds

Age group		Under 5		5-9		10-14		15-19		20-24		0-24	
Population in 2019	F	320,443,936		316,754,496		310,852,512		301,758,880		295,776,256		1,545,586,048	
	M	342,398,752		337,949,216		331,334,176		317,782,112		304,368,224		1,633,832,448	
		**Number incidence**	**Incidence/100,000 (change/pa)**	**Number incidence**	**Incidence/100,000 (change/pa)**	**Number incidence**	**Incidence/100,000 (change/pa)**	**Number incidence**	**Incidence/100,000 (change/pa)**	**Number incidence**	**Incidence/100,000 (change/pa)**	**Number incidence**	**Incidence/100,000 (change/pa)**
Enteric infections	F	455,426,592	142,123.6(-0.6%)	276,626,272	87,331.4(-0.4%)	259,943,280	83,622.7(-0.1%)	201,975,104	66,932.6(0.1%)	177,922,848	60,154.5(0.1%)	1,371,894,096	88,762.1(-0.4%)
	M	493,401,504	144,101.4(-0.5%)	298,930,560	88,454.3(-0.1%)	276,538,976	83,462.3(0.2%)	209,889,696	66,048.3(0.3%)	179,281,408	58,902.8(0.3%)	1,458,042,144	89,240.6(-0.2%)
HIV/AIDS	F	62,103	19.4(-1.1%)	153	0.0(.%)	91	0.0(.%)	138,468	45.9(-1.4%)	139,517	47.2(-1.4%)	340,332	22.0(-1.3%)
	M	64,848	18.9(-1.1%)	153	0.0(.%)	91	0.0(.%)	45,518	14.3(-0.3%)	98,936	32.5(-0.5%)	209,547	12.8(-0.7%)
Hepatitis	F	41,206,780	12,859.3(-0.5%)	16,015,513	5,056.1(-0.3%)	9,964,003	3,205.4(-0.5%)	7,733,428	2,562.8(-0.7%)	7,739,657	2,616.7(-0.5%)	82,659,384	5,348.1(-0.6%)
	M	43,633,160	12,743.4(-0.5%)	17,428,534	5,157.1(-0.4%)	11,259,141	3,398.1(-0.6%)	9,158,676	2,882.1(-0.8%)	9,752,641	3,204.2(-0.6%)	91,232,144	5,583.9(-0.7%)
Infectious skin conditions	F	163,923,888	51,155.2(0.2%)	144,684,704	45,677.2(0.3%)	122,477,880	39,400.6(0.3%)	113,848,960	37,728.5(0.2%)	107,831,088	36,457.0(0.1%)	652,766,528	42,234.2(0.2%)
	M	174,435,920	50,945.3(0.1%)	160,113,232	47,377.9(0.3%)	136,359,984	41,154.8(0.3%)	130,386,208	41,030.1(0.2%)	128,226,928	42,128.9(0.1%)	729,522,304	44,651.0(0.2%)
Lower respiratory infections	F	21,537,478	6,721.1(-1.7%)	14,703,271	4,641.9(-1.4%)	11,878,374	3,821.2(-0.9%)	11,136,138	3,690.4(-0.6%)	10,728,515	3,627.2(-0.4%)	69,983,776	4,528.0(-1.3%)
	M	23,546,122	6,876.8(-1.7%)	17,851,324	5,282.3(-1.3%)	15,150,964	4,572.7(-0.6%)	12,371,353	3,893.0(-0.4%)	10,883,982	3,575.9(-0.3%)	79,803,745	4,884.5(-1.2%)
Malaria	F	42,056,712	13,124.5(-0.5%)	18,274,742	5,769.4(-0.5%)	14,881,713	4,787.4(-0.5%)	10,948,187	3,628.1(-0.1%)	8,308,950	2,809.2(-0.4%)	94,470,304	6,112.3(-0.5%)
	M	43,130,064	12,596.4(-0.5%)	18,671,368	5,524.9(-0.5%)	14,996,943	4,526.2(-0.5%)	10,747,997	3,382.2(-0.2%)	7,830,697	2,572.8(-0.5%)	95,377,069	5,837.6(-0.6%)
Maternal sepsis & other maternal infections	F	0	0.0(.%)	0	0.0(.%)	62,939	20.2(-0.3%)	2,887,793	957.0(-1.3%)	7,004,614	2,368.2(-1.3%)	9,955,345	644.1(-1.2%)
	M	0	0.0(.%)	0	0.0(.%)	0	0.0(.%)	0	0.0(.%)	0	0.0(.%)	0	0.0(.%)
Meningitis & Encephalitis	F	741,672	231.5(-1.2%)	194,108	61.3(-1.1%)	110,715	35.6(-0.9%)	85,973	28.5(-0.7%)	80,634	27.3(-0.6%)	1,213,102	78.5(-1.2%)
	M	870,481	254.2(-1.3%)	224,714	66.5(-1.1%)	125,108	37.8(-1.0%)	92,865	29.2(-0.8%)	78,540	25.8(-0.7%)	1,391,708	85.2(-1.3%)
Neglected Tropical diseases	F	1,621,116	505.9(0.0%)	2,960,812	934.7(0.2%)	3,281,057	1,055.5(0.6%)	3,139,488	1,040.4(1.1%)	2,930,649	990.8(1.2%)	13,933,122	901.5(0.7%)
	M	1,573,428	459.5(-0.0%)	2,916,252	862.9(0.2%)	3,270,123	987.0(0.6%)	3,102,372	976.3(1.2%)	2,850,484	936.5(1.4%)	13,712,658	839.3(0.7%)
Neonatal sepsis & other neonatal infections	F	2,970,032	926.8(0.4%)	0	0.0(.%)	0	0.0(.%)	0	0.0(.%)	0	0.0(.%)	2,970,032	192.2(0.1%)
	M	3,340,036	975.5(0.1%)	0	0.0(.%)	0	0.0(.%)	0	0.0(.%)	0	0.0(.%)	3,340,036	204.4(-0.2%)
Other unspecified infectious diseases	F	0	0.0(.%)	0	0.0(.%)	0	0.0(.%)	0	0.0(.%)	0	0.0(.%)	0	0.0(.%)
	M	0	0.0(.%)	0	0.0(.%)	0	0.0(.%)	0	0.0(.%)	0	0.0(.%)	0	0.0(.%)
Rheumatic heart disease	F	73,506	22.9(0.6%)	194,234	61.3(0.7%)	266,788	85.8(0.9%)	263,582	87.3(0.9%)	207,227	70.1(0.8%)	1,005,337	65.0(0.9%)
	M	72,896	21.3(0.6%)	188,715	55.8(0.8%)	241,868	73.0(0.9%)	219,612	69.1(0.9%)	164,952	54.2(0.8%)	888,044	54.4(0.9%)
Sexually transmitted infections excluding HIV	F	0	0.0(.%)	590	0.2(0.1%)	4,435,033	1,426.7(0.3%)	21,251,650	7,042.6(0.0%)	42,428,392	14,344.8(0.0%)	68,115,665	4,407.1(0.2%)
	M	0	0.0(.%)	617	0.2(0.1%)	3,370,051	1,017.1(0.1%)	18,573,102	5,844.6(0.0%)	41,925,056	13,774.5(-0.0%)	63,868,826	3,909.1(0.2%)
Tuberculosis	F	168,994	52.7(-1.8%)	115,054	36.3(-1.9%)	168,785	54.3(-1.6%)	376,732	124.8(-1.1%)	421,044	142.4(-1.1%)	1,250,609	80.9(-1.4%)
	M	128,165	37.4(-1.7%)	84,874	25.1(-1.7%)	123,393	37.2(-1.3%)	327,616	103.1(-0.8%)	398,926	131.1(-0.7%)	1,062,974	65.1(-1.1%)
Upper respiratory infections	F	1,042,244,288	325,250.1(-0.0%)	875,405,312	276,367.1(0.0%)	762,121,408	245,171.4(-0.0%)	728,742,848	241,498.4(-0.0%)	697,653,568	235,872.1(-0.0%)	4,106,167,296	265,670.6(-0.0%)
	M	1,139,803,648	332,887.8(-0.1%)	950,007,616	281,109.6(-0.0%)	835,272,704	252,093.7(-0.0%)	788,471,424	248,117.0(-0.0%)	731,861,632	240,452.7(-0.0%)	4,445,416,960	272,085.3(-0.1%)
Vaccine Preventable disease	F	40,758,324	12,719.3(-1.6%)	4,228,408	1,334.9(-1.6%)	1,403,844	451.6(-1.4%)	833,509	276.2(-0.9%)	732,109	247.5(-0.7%)	47,956,196	3,102.8(-1.7%)
	M	40,910,408	11,948.2(-1.6%)	4,410,571	1,305.1(-1.5%)	1,512,721	456.6(-1.3%)	887,154	279.2(-0.8%)	743,705	244.3(-0.5%)	48,464,560	2,966.3(-1.7%)
Total communicable	F	1,812,717,978	565,689.6(-0.3%)	1,353,208,939	427,210.7(-0.1%)	1,190,729,122	383,052.8(-0.0%)	1,103,098,278	365,556.2(-0.0%)	1,063,921,584	359,704.9(-0.0%)	6,523,675,787	422,084.3(-0.2%)
	M	1,964,837,784	573,844.9(-0.3%)	1,470,639,814	435,165.9(-0.1%)	1,297,980,200	391,743.5(0.0%)	1,184,053,981	372,599.3(0.0%)	1,113,932,934	365,982.0(0.0%)	7,031,444,674	430,365.1(-0.1%)

S6a. Number of YLDs in 2019, YLD /100,000 in 2019 and change in YLDs/100,000 from 1990-2019, in 0-24 year olds by SDI groups

SDI group		Low		Low-Mid		Middle		High-mid		High	
Population in 2019	Female	345,856,384.00		418,727,616		442,771,136		200,230,976		137,035,328	
	Male	354,697,440.00		438,510,272		476,123,648		217,982,720		145,509,888	
		Number of YLDs	YLDs/100,000 (change/pa)	Number of YLDs	YLDs/100,000 (change/pa)	Number of YLDs	YLDs/100,000 (change/pa)	Number of YLDs	YLDs/100,000 (change/pa)	Number of YLDs	YLDs/100,000 (change/pa)
Enteric infections	Female	733,741	212.2(-0.8%)	761,922	182.0(-1.1%)	573,629	129.6(-0.3%)	249,327	124.5(-0.1%)	106,308	77.6(0.3%)
	Male	766,985	216.2(-0.7%)	798,293	182.0(-1.0%)	633,269	133.0(-0.3%)	277,043	127.1(-0.1%)	122,529	84.2(0.1%)
HIV/AIDS	Female	151,021	43.7(-1.1%)	95,800	22.9(2.1%)	87,149	19.7(19.7%)	9,066	4.5(7.6%)	2,138	1.6(-0.8%)
	Male	90,611	25.5(1.3%)	59,330	13.5(7.2%)	57,941	12.2(21.7%)	8,741	4.0(2.9%)	2,669	1.8(-2.0%)
Hepatitis	Female	21,731	6.3(0.1%)	25,924	6.2(0.2%)	27,596	6.2(-0.4%)	11,499	5.7(-0.5%)	4,790	3.5(-0.3%)
	Male	24,612	6.9(0.1%)	29,879	6.8(0.2%)	31,859	6.7(-0.5%)	13,322	6.1(-0.6%)	5,373	3.7(-0.4%)
Infectious skin conditions	Female	790,916	228.7(-0.1%)	867,659	207.2(-0.2%)	979,417	221.2(-0.2%)	409,853	204.7(-0.0%)	294,179	214.7(-0.0%)
	Male	902,252	254.4(-0.1%)	970,669	221.4(-0.1%)	1,123,072	235.9(-0.2%)	446,995	205.1(-0.1%)	233,015	160.1(-0.0%)
Lower respiratory infections	Female	30,492	8.8(-1.5%)	29,534	7.1(-1.6%)	27,573	6.2(-1.5%)	9,749	4.9(-1.4%)	5,348	3.9(-0.4%)
	Male	32,972	9.3(-1.5%)	35,967	8.2(-1.5%)	32,826	6.9(-1.4%)	11,283	5.2(-1.4%)	5,908	4.1(-0.5%)
Malaria	Female	783,064	226.4(-1.1%)	245,897	58.7(-1.0%)	92,041	20.8(-0.8%)	5,071	2.5(-1.5%)	40	0.0(-3.0%)
	Male	619,310	174.6(-1.2%)	191,556	43.7(-1.1%)	74,545	15.7(-0.9%)	4,231	1.9(-1.4%)	33	0.0(-3.0%)
Maternal sepsis and other maternal infections	Female	12,865	3.7(-0.9%)	12,523	3.0(-1.2%)	9,789	2.2(-1.4%)	3,500	1.7(-1.7%)	1,724	1.3(-1.8%)
	Male	0	0.0(.%)	0	0.0(.%)	0	0.0(.%)	0	0.0(.%)	0	0.0(.%)
Meningitis & Encephalitis	Female	101,252	29.3(-1.2%)	85,436	20.4(-1.4%)	52,491	11.9(-1.5%)	14,700	7.3(-1.7%)	3,997	2.9(-1.9%)
	Male	106,403	30.0(-1.3%)	89,179	20.3(-1.5%)	57,056	12.0(-1.6%)	17,072	7.8(-1.7%)	4,726	3.2(-1.8%)
Neglected Tropical diseases	Female	1,106,230	319.9(-1.7%)	698,370	166.8(-1.9%)	416,132	94.0(-1.8%)	93,660	46.8(-1.7%)	14,102	10.3(-1.6%)
	Male	1,134,452	319.8(-2.0%)	762,268	173.8(-2.0%)	466,162	97.9(-2.0%)	99,149	45.5(-1.7%)	12,248	8.4(-1.4%)
Neonatal sepsis and other neonatal infections	Female	160,363	46.4(19.3%)	290,624	69.4(16.6%)	381,708	86.2(7.7%)	153,720	76.8(4.8%)	12,952	9.5(0.6%)
	Male	149,542	42.2(19.0%)	315,649	72.0(19.9%)	417,977	87.8(8.6%)	160,843	73.8(5.4%)	15,569	10.7(0.5%)
Other unspecified infectious diseases	Female	213,218	61.6(-0.3%)	197,944	47.3(-0.6%)	108,391	24.5(-0.8%)	31,608	15.8(-1.0%)	11,161	8.1(-0.9%)
	Male	214,500	60.5(-0.3%)	171,971	39.2(-0.8%)	92,905	19.5(-0.9%)	27,336	12.5(-1.1%)	9,004	6.2(-0.6%)
Rheumatic heart disease	Female	120,077	34.7(0.6%)	120,460	28.8(0.8%)	90,354	20.4(0.1%)	20,040	10.0(0.2%)	805	0.6(0.1%)
	Male	110,584	31.2(0.7%)	100,615	22.9(0.8%)	83,241	17.5(-0.0%)	18,086	8.3(0.1%)	770	0.5(0.1%)
Sexually transmitted infections excluding HIV	Female	24,232	7.0(-0.0%)	20,076	4.8(0.5%)	18,199	4.1(0.2%)	6,893	3.4(-0.0%)	5,766	4.2(-0.3%)
	Male	11,994	3.4(0.2%)	16,576	3.8(0.4%)	20,191	4.2(0.1%)	7,343	3.4(0.2%)	2,059	1.4(-0.0%)
Tuberculosis	Female	254,740	73.7(-1.6%)	264,284	63.1(-1.7%)	161,009	36.4(-1.4%)	25,679	12.8(-1.6%)	2,809	2.0(-2.1%)
	Male	187,002	52.7(-1.3%)	160,532	36.6(-1.4%)	117,671	24.7(-1.4%)	24,473	11.2(-1.5%)	2,287	1.6(-1.9%)
Upper respiratory infections	Female	436,668	126.3(-0.2%)	554,972	132.5(-0.2%)	559,417	126.3(-0.2%)	243,565	121.6(-0.1%)	195,759	142.9(-0.0%)
	Male	493,620	139.2(-0.1%)	637,148	145.3(-0.2%)	659,099	138.4(-0.2%)	283,653	130.1(-0.1%)	207,958	142.9(-0.0%)
Vaccine Preventable disease	Female	39,506	11.4(-2.2%)	28,681	6.8(-2.4%)	24,751	5.6(-2.1%)	7,681	3.8(-2.0%)	4,575	3.3(-1.0%)
	Male	34,534	9.7(-2.2%)	25,775	5.9(-2.5%)	22,575	4.7(-2.2%)	7,213	3.3(-2.0%)	4,147	2.8(-1.1%)
Total communicable	Female	4,980,117	1,439.9(-1.0%)	4,300,105	1,026.9(-0.9%)	3,609,646	815.2(-0.5%)	1,295,611	647.1(-0.2%)	666,450	486.3(-0.1%)
	Male	4,879,374	1,375.6(-1.1%)	4,365,407	995.5(-0.9%)	3,890,389	817.1(-0.6%)	1,406,782	645.4(-0.2%)	628,296	431.8(-0.1%)

S6b. Number of YLDs in 2019, YLDs/100,000 in 2019 and change in YLDs/100,000 from 1990-2019, for under 5, 5-9, 10-14, 15-19, 20-24, 0-24 year olds

Age group		Under 5		5-9		10-14		15-19		20-24		0-24	
Population in 2019	Female	320,443,936		316,754,496		310,852,512		301,758,880		295,776,256		1,545,586,048	
	Male	342,398,752		337,949,216		331,334,176		317,782,112		304,368,224		1,633,832,448	
		Number of YLDs	YLDs/100,000 (change/pa)	Number of YLDs	YLDs/100,000 (change/pa)	Number of YLDs	YLDs/100,000 (change/pa)	Number of YLDs	YLDs/100,000 (change/pa)	Number of YLDs	YLDs/100,000 (change/pa)	Number of YLDs	YLDs/100,000 (change/pa)
Enteric infection	Female	855,993	267.1(-0.6%)	481,395	152.0(-0.5%)	453,017	145.7(-0.2%)	345,128	114.4(-0.0%)	290,993	98.4(-0.0%)	2,426,525	157.0(-0.4%)
	Male	926,662	270.6(-0.6%)	523,366	154.9(-0.3%)	487,418	147.1(0.0%)	364,765	114.8(0.1%)	297,746	97.8(0.1%)	2,599,957	159.1(-0.4%)
HIV/AIDS	Female	26,144	8.2(1.5%)	34,126	10.8(33.7%)	39,615	12.7(742.9%)	80,274	26.6(0.6%)	165,290	55.9(1.4%)	345,448	22.4(2.4%)
	Male	27,652	8.1(1.6%)	35,765	10.6(33.9%)	40,281	12.2(703.7%)	41,890	13.2(7.3%)	73,882	24.3(2.4%)	219,471	13.4(6.1%)
Hepatitis	Female	8,343	2.6(-0.9%)	22,223	7.0(-0.1%)	22,711	7.3(-0.1%)	19,377	6.4(-0.2%)	18,944	6.4(-0.1%)	91,598	5.9(-0.2%)
	Male	9,106	2.7(-1.0%)	24,126	7.1(-0.2%)	25,720	7.8(-0.1%)	22,501	7.1(-0.3%)	23,657	7.8(-0.2%)	105,110	6.4(-0.3%)
Infectious skin conditions	Female	690,380	215.4(-0.0%)	927,862	292.9(-0.0%)	690,126	222.0(-0.0%)	553,956	183.6(-0.2%)	482,023	163.0(-0.3%)	3,344,347	216.4(-0.1%)
	Male	756,778	221.0(-0.0%)	1,021,184	302.2(0.0%)	778,552	235.0(0.0%)	610,340	192.1(-0.1%)	511,635	168.1(-0.2%)	3,678,489	225.1(-0.1%)
Lower respirator infections	Female	35,169	11.0(-1.7%)	20,920	6.6(-1.5%)	16,534	5.3(-0.9%)	15,409	5.1(-0.6%)	14,729	5.0(-0.4%)	102,760	6.6(-1.4%)
	Male	39,720	11.6(-1.7%)	25,056	7.4(-1.4%)	21,440	6.5(-0.6%)	17,628	5.5(-0.3%)	15,183	5.0(-0.2%)	119,028	7.3(-1.3%)
Malaria	Female	289,030	90.2(-0.3%)	277,801	87.7(0.0%)	178,089	57.3(0.1%)	241,923	80.2(0.9%)	139,657	47.2(0.2%)	1,126,500	72.9(0.0%)
	Male	313,894	91.7(-0.3%)	286,229	84.7(0.1%)	168,254	50.8(0.1%)	75,396	23.7(0.3%)	46,193	15.2(0.0%)	889,966	54.5(-0.2%)
Maternal sepsis other maternal infections	Female	0	0.0(.%)	0	0.0(.%)	263	0.1(-0.3%)	11,682	3.9(-1.3%)	28,483	9.6(-1.3%)	40,429	2.6(-1.2%)
	Male	0	0.0(.%)	0	0.0(.%)	0	0.0(.%)	0	0.0(.%)	0	0.0(.%)	0	0.0(.%)
Meningitis & Encephalitis	Female	38,370	12.0(-1.3%)	54,989	17.4(-1.2%)	57,274	18.4(-1.1%)	55,525	18.4(-1.0%)	51,841	17.5(-1.1%)	257,998	16.7(-1.1%)
	Male	43,661	12.8(-1.4%)	59,975	17.7(-1.3%)	60,980	18.4(-1.2%)	57,687	18.2(-1.2%)	52,264	17.2(-1.2%)	274,568	16.8(-1.3%)
Neglected Tropical disease	Female	338,853	105.7(-1.2%)	568,979	179.6(-1.3%)	487,468	156.8(-1.3%)	462,616	153.3(-1.6%)	472,117	159.6(-1.7%)	2,330,032	150.8(-1.4%)
	Male	372,745	108.9(-1.2%)	671,039	198.6(-1.6%)	543,731	164.1(-1.8%)	443,638	139.6(-1.9%)	445,036	146.2(-1.9%)	2,476,188	151.6(-1.7%)
Neonatal sepsis other neonatal infections	Female	218,874	68.3(4.4%)	205,259	64.8(9.2%)	199,165	64.1(9.8%)	191,183	63.4(10.1%)	185,663	62.8(10.4%)	1,000,143	64.7(7.9%)
	Male	236,562	69.1(4.3%)	221,648	65.6(10.6%)	214,178	64.6(11.6%)	200,499	63.1(12.1%)	187,472	61.6(12.5%)	1,060,359	64.9(8.8%)
Other unspecifie infectious diseases	Female	159,555	49.8(-0.5%)	172,117	54.3(-0.0%)	114,346	36.8(0.0%)	70,887	23.5(0.6%)	45,714	15.5(-0.1%)	562,619	36.4(-0.2%)
	Male	177,471	51.8(-0.4%)	185,542	54.9(0.1%)	89,575	27.0(0.0%)	36,781	11.6(-0.1%)	26,630	8.7(-0.5%)	515,999	31.6(-0.3%)
Rheumatic hear disease	Female	5,958	1.9(0.5%)	34,512	10.9(0.6%)	74,094	23.8(0.8%)	110,027	36.5(0.8%)	127,396	43.1(0.8%)	351,987	22.8(0.9%)
	Male	5,906	1.7(0.6%)	33,778	10.0(0.7%)	70,212	21.2(0.7%)	97,989	30.8(0.7%)	105,633	34.7(0.7%)	313,517	19.2(0.9%)
Sexually transmitted infections excluding HIV	Female	0	0.0(.%)	0	0.0(0.1%)	3,015	1.0(0.1%)	18,838	6.2(0.2%)	53,368	18.0(0.2%)	75,221	4.9(0.4%)
	Male	0	0.0(.%)	0	0.0(0.1%)	4,308	1.3(-0.5%)	19,996	6.3(0.1%)	33,903	11.1(0.1%)	58,207	3.6(0.2%)
Tuberculosis	Female	104,816	32.7(-1.6%)	120,677	38.1(-1.7%)	83,177	26.8(-1.4%)	131,732	43.7(-1.0%)	268,435	90.8(-1.0%)	708,837	45.9(-1.3%)
	Male	76,317	22.3(-1.5%)	72,316	21.4(-1.8%)	33,545	10.1(-1.8%)	91,222	28.7(0.0%)	218,795	71.9(-0.7%)	492,195	30.1(-1.1%)
Upper respiratory infections	Female	484,942	151.3(-0.1%)	460,866	145.5(-0.1%)	371,067	119.4(-0.1%)	347,755	115.2(-0.1%)	327,033	110.6(-0.2%)	1,991,663	128.9(-0.1%)
	Male	540,791	157.9(-0.1%)	526,776	155.9(-0.1%)	432,395	130.5(-0.1%)	405,303	127.5(-0.1%)	377,644	124.1(-0.2%)	2,282,909	139.7(-0.1%)
Vaccine Preventable disease	Female	83,843	26.2(-2.0%)	7,516	2.4(-2.0%)	4,217	1.4(-1.4%)	4,431	1.5(-0.7%)	5,267	1.8(-0.4%)	105,275	6.8(-2.0%)
	Male	73,645	21.5(-2.1%)	6,688	2.0(-2.1%)	4,205	1.3(-1.4%)	4,527	1.4(-0.6%)	5,250	1.7(-0.3%)	94,315	5.8(-2.1%)
Total communicable	Female	3,340,269	1,042.4(-0.5%)	3,389,243	1,070.0(-0.4%)	2,794,176	898.9(-0.3%)	2,660,741	881.7(-0.4%)	2,676,953	905.1(-0.5%)	14,861,382	961.5(-0.4%)
	Male	3,600,909	1,051.7(-0.5%)	3,693,489	1,092.9(-0.5%)	2,974,792	897.8(-0.5%)	2,490,160	783.6(-0.5%)	2,420,924	795.4(-0.6%)	15,180,275	929.1(-0.5%)

S7a. Number of deaths in 2019, deaths/100,000 in 2019 and change in deaths/100,000 from 1990-2019, in 0-24 year olds by SDI groups

SDI group		Low		Low-Mid		Middle		High-mid		High	
Population in 2019	Female	345,856,384.00		418,727,616		442,771,136		200,230,976		137,035,328	
	Male	354,697,440.00		438,510,272		476,123,648		217,982,720		145,509,888	
		Number of deaths	Deaths/100,000 (change/pa)	Number of deaths	Deaths/100,000 (change/pa)	Number of deaths	Deaths/100,000 (change/pa)	Number of deaths	Deaths/100,000 (change/pa)	Number of deaths	Deaths/100,000 (change/pa)
Enteric infections	Female	214,947	62.1(-2.4%)	105,530	25.2(-2.7%)	30,876	7.0(-2.7%)	3,296	1.6(-2.8%)	192	0.1(-2.8%)
	Male	258,573	72.9(-2.3%)	110,656	25.2(-2.7%)	34,921	7.3(-2.7%)	3,943	1.8(-2.8%)	224	0.2(-2.8%)
HIV/AIDS	Female	35,107	10.2(-2.0%)	22,305	5.3(0.6%)	15,839	3.6(15.6%)	1,456	0.7(3.0%)	143	0.1(-2.3%)
	Male	30,072	8.5(-1.7%)	18,479	4.2(1.4%)	13,646	2.9(12.9%)	1,598	0.7(0.5%)	184	0.1(-2.7%)
Hepatitis	Female	4,783	1.4(-2.3%)	7,324	1.7(-2.4%)	2,302	0.5(-2.7%)	375	0.2(-2.6%)	49	0.0(-2.3%)
	Male	6,780	1.9(-2.2%)	9,223	2.1(-2.2%)	4,071	0.9(-2.3%)	899	0.4(-2.2%)	99	0.1(-2.1%)
Infectious skin conditions	Female	1,239	0.4(-1.5%)	1,354	0.3(-1.8%)	553	0.1(-1.6%)	107	0.1(-1.9%)	23	0.0(0.9%)
	Male	862	0.2(-1.3%)	939	0.2(-1.8%)	465	0.1(-1.9%)	124	0.1(-1.9%)	27	0.0(0.4%)
Lower respiratory infections	Female	195,468	56.5(-2.3%)	114,118	27.3(-2.6%)	46,247	10.4(-2.8%)	6,939	3.5(-2.9%)	940	0.7(-2.3%)
	Male	204,774	57.7(-2.4%)	110,476	25.2(-2.7%)	54,146	11.4(-2.8%)	8,722	4.0(-2.9%)	1,135	0.8(-2.4%)
Malaria	Female	148,480	42.9(-2.1%)	44,198	10.6(-2.1%)	16,917	3.8(-2.0%)	1,666	0.8(-2.1%)	0	0.0(-3.3%)
	Male	150,572	42.5(-2.1%)	45,458	10.4(-2.0%)	18,221	3.8(-1.9%)	1,850	0.8(-1.9%)	0	0.0(-3.3%)
Maternal sepsis and other maternal infections	Female	3,500	1.0(-2.1%)	1,002	0.2(-2.9%)	261	0.1(-3.0%)	43	0.0(-3.0%)	4	0.0(-2.7%)
	Male		.(.%)		.(.%)		.(.%)		.(.%)		.(.%)
Meningitis & Encephalitis	Female	47,111	13.6(-2.3%)	23,516	5.6(-2.6%)	9,898	2.2(-2.5%)	1,996	1.0(-2.6%)	383	0.3(-2.3%)
	Male	58,549	16.5(-2.1%)	26,952	6.1(-2.4%)	12,758	2.7(-2.5%)	2,606	1.2(-2.6%)	448	0.3(-2.4%)
Neglected Tropical diseases	Female	7,005	2.0(-2.8%)	4,261	1.0(-2.5%)	4,098	0.9(-2.0%)	446	0.2(-1.8%)	15	0.0(-1.9%)
	Male	12,221	3.4(-2.8%)	6,743	1.5(-2.4%)	5,055	1.1(-1.6%)	730	0.3(-1.6%)	18	0.0(-2.3%)
Neonatal sepsis and other neonatal infections	Female	49,429	14.3(-1.2%)	31,526	7.5(-1.5%)	15,374	3.5(-1.0%)	3,195	1.6(-1.2%)	717	0.5(-1.8%)
	Male	62,342	17.6(-1.2%)	38,530	8.8(-1.5%)	20,443	4.3(-1.2%)	3,900	1.8(-1.3%)	911	0.6(-1.8%)
Other unspecified infectious diseases	Female	5,315	1.5(-1.6%)	3,110	0.7(-1.8%)	1,391	0.3(-1.7%)	475	0.2(-1.6%)	263	0.2(-0.6%)
	Male	6,360	1.8(-1.6%)	5,242	1.2(-1.6%)	2,026	0.4(-1.9%)	687	0.3(-1.7%)	319	0.2(-0.8%)
Rheumatic heart disease	Female	2,695	0.8(-2.1%)	4,140	1.0(-1.8%)	1,605	0.4(-2.4%)	227	0.1(-2.6%)	38	0.0(-2.4%)
	Male	3,227	0.9(-1.7%)	4,082	0.9(-1.6%)	1,706	0.4(-2.1%)	302	0.1(-2.3%)	38	0.0(-2.3%)
Sexually transmitted infections excluding HIV	Female	21,752	6.3(-1.8%)	9,361	2.2(-1.6%)	5,363	1.2(-0.8%)	772	0.4(-0.8%)	74	0.1(-0.8%)
	Male	26,236	7.4(-1.7%)	11,039	2.5(-1.5%)	6,866	1.4(-0.8%)	972	0.4(-0.7%)	83	0.1(-0.9%)
Tuberculosis	Female	27,582	8.0(-2.5%)	19,614	4.7(-2.7%)	6,215	1.4(-2.8%)	818	0.4(-2.8%)	54	0.0(-2.9%)
	Male	33,257	9.4(-2.4%)	19,351	4.4(-2.6%)	8,416	1.8(-2.6%)	1,098	0.5(-2.7%)	46	0.0(-3.0%)
Upper respiratory infections	Female	1,390	0.4(-2.8%)	361	0.1(-3.1%)	212	0.0(-3.1%)	61	0.0(-3.1%)	18	0.0(-2.8%)
	Male	2,008	0.6(-2.7%)	478	0.1(-2.9%)	300	0.1(-3.0%)	95	0.0(-3.0%)	31	0.0(-2.7%)
Vaccine Preventable disease	Female	78,629	22.7(-2.9%)	26,764	6.4(-3.1%)	10,469	2.4(-3.0%)	1,231	0.6(-3.1%)	94	0.1(-3.2%)
	Male	74,540	21.0(-2.9%)	24,910	5.7(-3.1%)	10,623	2.2(-3.0%)	1,222	0.6(-3.1%)	97	0.1(-3.2%)
Total communicable	Female	844,431	244.2(-2.4%)	418,482	99.9(-2.6%)	167,620	37.9(-2.6%)	23,105	11.5(-2.7%)	3,008	2.2(-2.4%)
	Male	930,373	262.3(-2.4%)	432,557	98.6(-2.6%)	193,664	40.7(-2.6%)	28,747	13.2(-2.7%)	3,660	2.5(-2.4%)

Note – For infectious skin disease deaths only bacterial skin disease contribute to this value as there are no deaths recorded for scabies, fungal and viral skin diseases.

S7b. Number of deaths in 2019, deaths/100,000 in 2019 and change in deaths/100,000 from 1990-2019, for 5-9, 10-14, 15-19, 20-24, 0-24 year olds

Age group		Under 5		5-9		10-14		15-19		20-24		0-24	
Population in 2019	Female	320,443,936		316,754,496		310,852,512		301,758,880		295,776,256		1,545,586,048	
	Male	342,398,752		337,949,216		331,334,176		317,782,112		304,368,224		1,633,832,448	
		Number of deaths	Deaths/100,000 (change/pa)	Number of deaths	Deaths/100,000 (change/pa)	Number of deaths	Deaths/100,000 (change/pa)	Number of deaths	Deaths/100,000 (change/pa)	Number of deaths	Deaths/100,000 (change/pa)	Number of deaths	Deaths/100,000 (change/pa)
Enteric infection	Female	266,985	83.3(-2.3%)	34,547	10.9(-1.9%)	21,904	7.0(-1.7%)	17,220	5.7(-1.6%)	14,360	4.9(-1.6%)	355,016	23.0(-2.3%)
	Male	306,063	89.4(-2.3%)	40,519	12.0(-1.5%)	25,356	7.7(-1.5%)	20,214	6.4(-1.5%)	16,377	5.4(-1.3%)	408,529	25.0(-2.2%)
HIV/AIDS	Female	23,874	7.5(-1.2%)	5,995	1.9(12.7%)	7,250	2.3(120.9%)	14,814	4.9(2.9%)	22,981	7.8(0.4%)	74,914	4.8(0.2%)
	Male	25,054	7.3(-1.2%)	6,384	1.9(12.9%)	7,863	2.4(78.8%)	12,850	4.0(24.1%)	11,884	3.9(3.0%)	64,035	3.9(0.8%)
Hepatitis	Female	5,680	1.8(-2.6%)	1,734	0.5(-2.5%)	1,730	0.6(-1.9%)	1,840	0.6(-2.0%)	3,854	1.3(-1.6%)	14,837	1.0(-2.3%)
	Male	6,864	2.0(-2.5%)	1,897	0.6(-2.2%)	2,174	0.7(-1.6%)	2,919	0.9(-1.6%)	7,225	2.4(-1.1%)	21,079	1.3(-2.1%)
Infectious skin conditions	Female	2,329	0.7(-1.3%)	200	0.1(-1.6%)	209	0.1(-0.8%)	278	0.1(-0.6%)	261	0.1(-0.5%)	3,278	0.2(-1.3%)
	Male	1,299	0.4(-1.7%)	142	0.0(-1.7%)	211	0.1(-1.2%)	403	0.1(-0.4%)	365	0.1(-0.6%)	2,421	0.1(-1.5%)
Lower respirator infections	Female	330,613	103.2(-2.3%)	13,490	4.3(-2.0%)	7,053	2.3(-1.4%)	6,065	2.0(-1.2%)	6,760	2.3(-1.1%)	363,980	23.5(-2.4%)
	Male	341,315	99.7(-2.4%)	14,050	4.2(-1.9%)	7,794	2.4(-1.4%)	7,473	2.4(-1.1%)	8,934	2.9(-0.5%)	379,566	23.2(-2.4%)
Malaria	Female	182,099	56.8(-1.3%)	10,738	3.4(-2.2%)	6,265	2.0(-1.9%)	6,025	2.0(-1.3%)	6,189	2.1(-1.2%)	211,315	13.7(-1.5%)
	Male	174,264	50.9(-1.3%)	14,259	4.2(-2.0%)	8,288	2.5(-1.8%)	9,523	3.0(-0.7%)	9,820	3.2(-0.2%)	216,154	13.2(-1.5%)
Maternal sepsis other maternal infections	Female		.(.%)		.(.%)	76	0.0(-1.9%)	1,713	0.6(-2.3%)	3,026	1.0(-2.4%)	4,815	0.3(-2.3%)
	Male		.(.%)		.(.%)		.(.%)		.(.%)		.(.%)		.(.%)
Meningitis & Encephalitis	Female	57,742	18.0(-2.3%)	8,923	2.8(-1.9%)	6,512	2.1(-1.3%)	5,146	1.7(-1.2%)	4,631	1.6(-1.1%)	82,954	5.4(-2.2%)
	Male	71,317	20.8(-2.1%)	10,503	3.1(-1.7%)	6,877	2.1(-1.2%)	6,580	2.1(-1.0%)	6,100	2.0(-0.7%)	101,377	6.2(-2.0%)
Neglected Tropical disease	Female	8,169	2.5(-2.4%)	3,145	1.0(-2.4%)	1,762	0.6(-2.2%)	1,321	0.4(-2.1%)	1,437	0.5(-2.0%)	15,834	1.0(-2.4%)
	Male	10,926	3.2(-2.3%)	4,823	1.4(-2.4%)	3,362	1.0(-2.3%)	2,684	0.8(-2.2%)	2,984	1.0(-2.0%)	24,779	1.5(-2.3%)
Neonatal sepsis other neonatal infections	Female	100,303	31.3(-0.5%)		.(.%)		.(.%)		.(.%)		.(.%)	100,303	6.5(-0.8%)
	Male	126,214	36.9(-0.6%)		.(.%)		.(.%)		.(.%)		.(.%)	126,214	7.7(-0.8%)
Other unspecifie infectious diseases	Female	7,816	2.4(-1.2%)	938	0.3(-1.3%)	527	0.2(-0.8%)	644	0.2(-0.6%)	635	0.2(-0.4%)	10,559	0.7(-1.2%)
	Male	9,072	2.6(-1.5%)	1,639	0.5(-0.7%)	1,014	0.3(-0.5%)	1,486	0.5(-0.3%)	1,434	0.5(-0.1%)	14,644	0.9(-1.3%)
Rheumatic hear disease	Female	803	0.3(-2.7%)	833	0.3(-2.1%)	1,465	0.5(-1.6%)	2,498	0.8(-1.7%)	3,120	1.1(-1.7%)	8,719	0.6(-1.9%)
	Male	852	0.2(-2.4%)	610	0.2(-2.0%)	1,322	0.4(-1.4%)	2,923	0.9(-1.3%)	3,659	1.2(-1.3%)	9,366	0.6(-1.6%)
Sexually transmitted infections excluding HIV	Female	36,552	11.4(-0.7%)	208	0.1(-0.4%)	70	0.0(-0.8%)	206	0.1(-1.2%)	337	0.1(-1.3%)	37,374	2.4(-0.9%)
	Male	44,876	13.1(-0.7%)	209	0.1(-0.4%)	63	0.0(-1.9%)	55	0.0(-1.5%)	54	0.0(-1.5%)	45,258	2.8(-0.9%)
Tuberculosis	Female	24,960	7.8(-2.6%)	4,463	1.4(-2.5%)	3,931	1.3(-2.2%)	8,030	2.7(-2.1%)	12,929	4.4(-2.1%)	54,313	3.5(-2.5%)
	Male	25,203	7.4(-2.6%)	3,547	1.0(-2.5%)	3,503	1.1(-2.2%)	9,563	3.0(-1.7%)	20,385	6.7(-1.5%)	62,201	3.8(-2.3%)
Upper respiratory infections	Female	1,787	0.6(-2.8%)	142	0.0(-2.6%)	57	0.0(-2.5%)	30	0.0(-2.7%)	27	0.0(-2.8%)	2,043	0.1(-2.8%)
	Male	2,505	0.7(-2.6%)	161	0.0(-2.5%)	124	0.0(-2.2%)	70	0.0(-2.4%)	54	0.0(-2.6%)	2,915	0.2(-2.7%)
Vaccine Preventable disease	Female	105,018	32.8(-2.8%)	8,357	2.6(-2.8%)	2,125	0.7(-2.8%)	1,014	0.3(-2.8%)	764	0.3(-2.8%)	117,278	7.6(-2.8%)
	Male	99,829	29.2(-2.8%)	7,419	2.2(-2.8%)	2,081	0.6(-2.8%)	1,242	0.4(-2.7%)	909	0.3(-2.6%)	111,480	6.8(-2.8%)
Total communicable	Female	1,154,730	360.4(-2.2%)	93,711	29.6(-2.1%)	60,936	19.6(-1.7%)	66,846	22.2(-1.4%)	81,310	27.5(-1.4%)	1,457,533	94.3(-2.2%)
	Male	1,245,653	363.8(-2.2%)	106,164	31.4(-1.9%)	70,032	21.1(-1.5%)	77,986	24.5(-1.1%)	90,184	29.6(-1.0%)	1,590,018	97.3(-2.2%)

Note – For infectious skin disease deaths only bacterial skin disease contribute to this value as there are no deaths recorded for scabies, fungal and viral skin diseases.

S8a. Number of DALYs in 2019, DALYs/100,000 in 2019 and change in DALYs/100,000 from 1990-2019, in 0-24 year olds by SDI groups

SDI group		Low		Low-Mid		Middle		High-mid		High	
Population in 2019	Female	345,856,384		418,727,616		442,771,136		200,230,976		137,035,328	
	Male	354,697,440		438,510,272		476,123,648		217,982,720		145,509,888	
		Number of DALYs	DALYs/100 000 (change/pa)	Number of DALYs	DALYs/100 000 (change/pa)	Number of DALYs	DALYs/100 000 (change/pa)	Number of DALYs	DALYs/100 000 (change/pa)	Number of DALYs	DALYs/100 000 (change/pa)
Enteric infections	Female	19,075,428	5,515.4(-2.3%)	9,538,265	2,277.9(-2.7%)	3,147,428	710.8(-2.6%)	520,888	260.1(-2.4%)	122,146	89.1(-1.3%)
	Male	22,787,265	6,424.4(-2.3%)	10,013,959	2,283.6(-2.6%)	3,540,307	743.6(-2.6%)	599,575	275.1(-2.5%)	141,070	96.9(-1.3%)
HIV/AIDS	Female	2,853,953	825.2(-2.0%)	1,796,668	429.1(0.6%)	1,261,721	285.0(14.3%)	121,038	60.4(3.2%)	12,885	9.4(-2.3%)
	Male	2,467,002	695.5(-1.7%)	1,509,491	344.2(1.2%)	1,097,235	230.5(12.1%)	132,398	60.7(0.7%)	16,229	11.2(-2.6%)
Hepatitis	Female	398,121	115.1(-2.3%)	600,135	143.3(-2.4%)	202,507	45.7(-2.7%)	39,859	19.9(-2.5%)	8,214	6.0(-1.6%)
	Male	547,914	154.5(-2.2%)	740,650	168.9(-2.2%)	333,885	70.1(-2.3%)	78,758	36.1(-2.1%)	12,256	8.4(-1.8%)
Infectious skin conditions	Female	896,698	259.3(-0.4%)	980,275	234.1(-0.5%)	1,024,194	231.3(-0.3%)	418,377	208.9(-0.1%)	295,983	216.0(-0.0%)
	Male	972,585	274.2(-0.3%)	1,046,302	238.6(-0.4%)	1,159,447	243.5(-0.3%)	456,586	209.5(-0.2%)	235,118	161.6(-0.0%)
Lower respiratory infections	Female	17,035,295	4,925.5(-2.3%)	9,939,668	2,373.8(-2.6%)	4,015,181	906.8(-2.8%)	599,061	299.2(-2.9%)	81,359	59.4(-2.3%)
	Male	17,833,771	5,027.9(-2.4%)	9,616,491	2,193.0(-2.7%)	4,689,280	984.9(-2.8%)	747,635	343.0(-2.9%)	98,033	67.4(-2.3%)
Malaria	Female	13,522,927	3,910.0(-2.1%)	3,986,266	952.0(-2.0%)	1,508,469	340.7(-2.0%)	144,364	72.1(-2.1%)	54	0.0(-3.2%)
	Male	13,424,993	3,784.9(-2.1%)	4,000,548	912.3(-2.0%)	1,585,028	332.9(-1.9%)	157,360	72.2(-1.9%)	43	0.0(-3.2%)
Maternal sepsis and other maternal infections	Female	252,528	73.0(-2.0%)	81,005	19.3(-2.8%)	27,607	6.2(-2.8%)	6,423	3.2(-2.7%)	1,998	1.5(-2.0%)
	Male	0	0.0(.%)	0	0.0(.%)	0	0.0(.%)	0	0.0(.%)	0	0.0(.%)
Meningitis & Encephalitis	Female	4,094,503	1,183.9(-2.3%)	2,028,189	484.4(-2.6%)	866,747	195.8(-2.5%)	179,025	89.4(-2.6%)	34,896	25.5(-2.3%)
	Male	5,063,320	1,427.5(-2.1%)	2,318,504	528.7(-2.4%)	1,108,703	232.9(-2.5%)	230,819	105.9(-2.6%)	40,935	28.1(-2.3%)
Neglected Tropical diseases	Female	1,681,026	486.0(-2.4%)	1,041,461	248.7(-2.2%)	751,721	169.8(-1.9%)	129,699	64.8(-1.8%)	15,362	11.2(-1.6%)
	Male	2,123,772	598.8(-2.6%)	1,293,521	295.0(-2.2%)	874,673	183.7(-1.8%)	156,727	71.9(-1.7%)	13,683	9.4(-1.6%)
Neonatal sepsis and other neonatal infections	Female	4,550,609	1,315.8(-1.1%)	3,090,678	738.1(-1.4%)	1,746,868	394.5(-0.5%)	437,317	218.4(-0.4%)	76,620	55.9(-1.6%)
	Male	5,686,922	1,603.3(-1.2%)	3,737,984	852.4(-1.4%)	2,233,294	469.1(-0.8%)	507,032	232.6(-0.7%)	96,470	66.3(-1.6%)
Other unspecified infectious diseases	Female	665,941	192.5(-1.3%)	456,912	109.1(-1.5%)	224,471	50.7(-1.4%)	71,588	35.8(-1.4%)	32,998	24.1(-0.7%)
	Male	748,059	210.9(-1.4%)	594,670	135.6(-1.5%)	260,096	54.6(-1.7%)	84,113	38.6(-1.6%)	35,419	24.3(-0.7%)
Rheumatic heart disease	Female	319,955	92.5(-1.7%)	419,346	100.1(-1.5%)	206,706	46.7(-2.0%)	36,427	18.2(-2.0%)	3,540	2.6(-2.2%)
	Male	348,531	98.3(-1.4%)	391,372	89.3(-1.3%)	205,762	43.2(-1.7%)	39,493	18.1(-1.9%)	3,497	2.4(-2.2%)
Sexually transmitted infections excluding HIV	Female	1,947,819	563.2(-1.8%)	846,292	202.1(-1.6%)	492,293	111.2(-0.8%)	74,900	37.4(-0.7%)	12,148	8.9(-0.6%)
	Male	2,337,291	659.0(-1.7%)	994,800	226.9(-1.5%)	628,342	132.0(-0.8%)	93,341	42.8(-0.7%)	9,342	6.4(-0.7%)
Tuberculosis	Female	2,482,598	717.8(-2.5%)	1,777,838	424.6(-2.7%)	629,988	142.3(-2.7%)	86,176	43.0(-2.7%)	6,635	4.8(-2.8%)
	Male	2,806,482	791.2(-2.4%)	1,624,346	370.4(-2.6%)	744,067	156.3(-2.6%)	103,754	47.6(-2.7%)	5,544	3.8(-2.8%)
Upper respiratory infections	Female	556,325	160.9(-1.8%)	585,739	139.9(-1.3%)	577,598	130.5(-1.0%)	248,809	124.3(-0.8%)	197,267	144.0(-0.1%)
	Male	666,331	187.9(-1.7%)	677,817	154.6(-1.1%)	684,709	143.8(-1.0%)	291,733	133.8(-0.8%)	210,583	144.7(-0.2%)
Vaccine Preventable disease	Female	6,820,150	1,972.0(-2.9%)	2,334,566	557.5(-3.1%)	926,818	209.3(-3.0%)	113,518	56.7(-3.0%)	12,598	9.2(-3.1%)
	Male	6,462,075	1,821.9(-2.9%)	2,172,580	495.4(-3.1%)	937,017	196.8(-3.0%)	112,274	51.5(-3.1%)	12,386	8.5(-3.1%)
Total communicable	Female	77,153,876	22,308.1(-2.4%)	39,503,305	9,434.1(-2.6%)	17,610,318	3,977.3(-2.4%)	3,227,471	1,611.9(-2.4%)	914,703	667.5(-1.5%)
	Male	84,276,312	23,760.1(-2.3%)	40,733,035	9,289.0(-2.5%)	20,081,847	4,217.8(-2.4%)	3,791,599	1,739.4(-2.4%)	930,610	639.6(-1.6%)

The sum of the four groups is less than the total from the age based table as the age based table is calculated from global numbers which account for misplace people who would not be included in any of the income groups.

S8b. Number of DALYs in 2019, DALYs/100,000 in 2019 and change in DALYs/100,000 from 1990-2019, for 5-9, 10-14, 15-19, 20-24, 0-24 year olds

Age group		Under 5		5-9		10-14	
Population in 2019	Female	320,443,936		316,754,496		310,852,512	
	Male	342,398,752		337,949,216		331,334,176	
		Number of DALYs	DALYs/100,000 (change/pa)	Number of DALYs	DALYs/100,000 (change/pa)	Number of DALYs	DALYs/100,000 (change/pa)
Enteric infections	Female	24,164,588	7,541.0(-2.3%)	3,306,889	1,044.0(-1.8%)	2,127,264	684.3(-1.5%)
	Male	27,646,714	8,074.4(-2.2%)	3,835,011	1,134.8(-1.4%)	2,425,165	731.9(-1.3%)
HIV/AIDS	Female	2,100,134	655.4(-1.2%)	524,405	165.6(13.3%)	593,767	191.0(128.2%)
	Male	2,204,065	643.7(-1.2%)	557,569	165.0(13.5%)	641,350	193.6(83.6%)
Hepatitis	Female	504,063	157.3(-2.6%)	164,003	51.8(-2.4%)	154,941	49.8(-1.7%)
	Male	607,837	177.5(-2.5%)	179,116	53.0(-2.1%)	191,853	57.9(-1.5%)
Infectious skin conditions	Female	894,462	279.1(-0.5%)	944,189	298.1(-0.1%)	706,137	227.2(-0.1%)
	Male	870,211	254.2(-0.4%)	1,032,822	305.6(0.0%)	794,667	239.8(-0.0%)
Lower respiratory infections	Female	29,100,814	9,081.4(-2.3%)	1,124,192	354.9(-2.0%)	555,592	178.7(-1.4%)
	Male	30,061,424	8,779.7(-2.4%)	1,173,333	347.2(-1.9%)	617,011	186.2(-1.4%)
Malaria	Female	16,130,097	5,033.7(-1.3%)	1,156,017	365.0(-2.0%)	656,941	211.3(-1.7%)
	Male	15,463,934	4,516.4(-1.3%)	1,451,711	429.6(-1.8%)	801,713	242.0(-1.6%)
Maternal sepsis & other maternal infections	Female	0	0.0(.%)	0	0.0(.%)	6,037	1.9(-1.9%)
	Male	0	0.0(.%)	0	0.0(.%)	0	0.0(.%)
Meningitis & Encephalitis	Female	5,084,719	1,586.8(-2.2%)	784,736	247.7(-1.8%)	555,021	178.5(-1.3%)
	Male	6,277,221	1,833.3(-2.0%)	918,338	271.7(-1.7%)	586,505	177.0(-1.2%)
Neglected Tropica diseases	Female	1,048,491	327.2(-2.2%)	826,181	260.8(-1.8%)	622,122	200.1(-1.6%)
	Male	1,320,522	385.7(-2.1%)	1,065,237	315.2(-2.0%)	800,639	241.6(-2.0%)
Neonatal sepsis & other neonatal infections	Female	9,127,055	2,848.3(-0.5%)	205,259	64.8(9.2%)	199,165	64.1(9.8%)
	Male	11,446,419	3,343.0(-0.6%)	221,648	65.6(10.6%)	214,178	64.6(11.6%)
Other unspecified infectious disease:	Female	844,424	263.5(-1.1%)	248,799	78.5(-0.5%)	154,586	49.7(-0.3%)
	Male	972,133	283.9(-1.4%)	319,505	94.5(-0.3%)	167,060	50.4(-0.3%)
Rheumatic heart disease	Female	75,049	23.4(-2.6%)	102,645	32.4(-1.7%)	186,079	59.9(-1.1%)
	Male	79,217	23.1(-2.3%)	83,640	24.7(-1.6%)	171,236	51.7(-0.9%)
Sexually transmitte infections excludin HIV	Female	3,243,285	1,012.1(-0.7%)	17,001	5.4(-0.4%)	8,378	2.7(-0.5%)
	Male	3,981,035	1,162.7(-0.7%)	17,112	5.1(-0.4%)	9,147	2.8(-1.5%)
Tuberculosis	Female	2,282,353	712.2(-2.6%)	485,690	153.3(-2.4%)	383,643	123.4(-2.1%)
	Male	2,274,292	664.2(-2.5%)	362,249	107.2(-2.4%)	301,286	90.9(-2.1%)
Upper respiratory infections	Female	640,445	199.9(-1.9%)	472,485	149.2(-0.4%)	375,439	120.8(-0.2%)
	Male	759,398	221.8(-1.7%)	539,926	159.8(-0.3%)	441,900	133.4(-0.3%)
Vaccine Preventable disease	Female	9,225,050	2,878.8(-2.8%)	690,975	218.1(-2.8%)	166,661	53.6(-2.8%)
	Male	8,768,615	2,560.9(-2.8%)	613,069	181.4(-2.8%)	163,265	49.3(-2.8%)
Total communicable	Female	104,465,027	32,600.1(-2.2%)	11,053,467	3,489.6(-1.9%)	7,451,772	2,397.2(-1.3%)
	Male	112,733,037	32,924.5(-2.2%)	12,370,285	3,660.4(-1.7%)	8,326,975	2,513.2(-1.3%)

Age group		15-19		20-24		0-24	
Population in 2019	Female	301,758,880		295,776,256		1,545,586,048	
	Male	317,782,112		304,368,224		1,633,832,448	
		Number of DALYs	DALYs/100,000 (change/pa)	Number of DALYs	DALYs/100,000 (change/pa)	Number of DALYs	DALYs/100,000 (change/pa)
Enteric infections	Female	1,575,404	522.1(-1.4%)	1,246,589	421.5(-1.3%)	32,420,734	2,097.6(-2.2%)
	Male	1,807,909	568.9(-1.3%)	1,387,350	455.8(-1.1%)	37,102,149	2,270.9(-2.2%)
HIV/AIDS	Female	1,138,905	377.4(2.7%)	1,694,269	572.8(0.5%)	6,051,479	391.5(0.2%)
	Male	959,441	301.9(22.3%)	864,480	284.0(3.0%)	5,226,905	319.9(0.6%)
Hepatitis	Female	150,804	50.0(-1.8%)	275,412	93.1(-1.5%)	1,249,223	80.8(-2.3%)
	Male	230,867	72.6(-1.5%)	504,328	165.7(-1.1%)	1,714,000	104.9(-2.1%)
Infectious skin conditions	Female	573,846	190.2(-0.2%)	499,363	168.8(-0.3%)	3,617,998	234.1(-0.3%)
	Male	639,119	201.1(-0.2%)	535,926	176.1(-0.3%)	3,872,745	237.0(-0.2%)
Lower respiratory infections	Female	448,732	148.7(-1.2%)	464,553	157.1(-1.1%)	31,693,883	2,050.6(-2.4%)
	Male	551,105	173.4(-1.1%)	609,620	200.3(-0.5%)	33,012,493	2,020.6(-2.4%)
Malaria	Female	672,479	222.9(-0.8%)	551,438	186.4(-1.0%)	19,166,971	1,240.1(-1.5%)
	Male	755,664	237.8(-0.6%)	699,655	229.9(-0.2%)	19,172,678	1,173.5(-1.4%)
Maternal sepsis & other maternal infections	Female	134,087	44.4(-2.3%)	229,815	77.7(-2.3%)	369,940	23.9(-2.2%)
	Male	0	0.0(.%)	0	0.0(.%)	0	0.0(.%)
Meningitis & Encephalitis	Female	423,224	140.3(-1.1%)	360,050	121.7(-1.1%)	7,207,751	466.3(-2.2%)
	Male	527,518	166.0(-1.0%)	458,133	150.5(-0.7%)	8,767,715	536.6(-2.0%)
Neglected Tropica diseases	Female	557,018	184.6(-1.7%)	567,762	192.0(-1.7%)	3,621,573	234.3(-1.9%)
	Male	635,252	199.9(-2.0%)	643,604	211.5(-1.9%)	4,465,253	273.3(-2.1%)
Neonatal sepsis & other neonatal infections	Female	191,183	63.4(10.1%)	185,663	62.8(10.4%)	9,908,325	641.1(-0.6%)
	Male	200,499	63.1(12.1%)	187,472	61.6(12.5%)	12,270,216	751.0(-0.7%)
Other unspecified infectious disease:	Female	116,917	38.7(0.0%)	87,946	29.7(-0.2%)	1,452,672	94.0(-0.9%)
	Male	142,816	44.9(-0.2%)	122,036	40.1(-0.2%)	1,723,549	105.5(-1.1%)
Rheumatic heart disease	Female	288,440	95.6(-1.2%)	335,027	113.3(-1.3%)	987,240	63.9(-1.5%)
	Male	306,664	96.5(-1.0%)	348,993	114.7(-1.0%)	989,751	60.6(-1.2%)
Sexually transmitte infections excludin HIV	Female	33,591	11.1(-0.6%)	75,786	25.6(-0.4%)	3,378,042	218.6(-0.9%)
	Male	23,936	7.5(-0.4%)	37,512	12.3(-0.2%)	4,068,741	249.0(-0.9%)
Tuberculosis	Female	705,436	233.8(-2.0%)	1,128,727	381.6(-1.9%)	4,985,849	322.6(-2.4%)
	Male	774,020	243.6(-1.6%)	1,575,194	517.5(-1.4%)	5,287,040	323.6(-2.3%)
Upper respiratory infections	Female	349,916	116.0(-0.2%)	328,837	111.2(-0.3%)	2,167,122	140.2(-1.1%)
	Male	410,321	129.1(-0.2%)	381,236	125.3(-0.3%)	2,532,781	155.0(-1.0%)
Vaccine Preventable disease	Female	76,908	25.5(-2.8%)	56,081	19.0(-2.7%)	10,215,674	661.0(-2.8%)
	Male	93,244	29.3(-2.7%)	65,729	21.6(-2.6%)	9,703,923	593.9(-2.8%)
Total communicable	Female	7,436,890	2,464.5(-1.2%)	8,087,317	2,734.3(-1.2%)	138,494,472	8,960.6(-2.2%)
	Male	8,058,373	2,535.8(-1.0%)	8,421,269	2,766.8(-0.9%)	149,909,939	9,175.4(-2.2%)

S9. Proportion of communicable disease DALYs and deaths in 2019 attributed by each specific cause, by age, sex and SDI

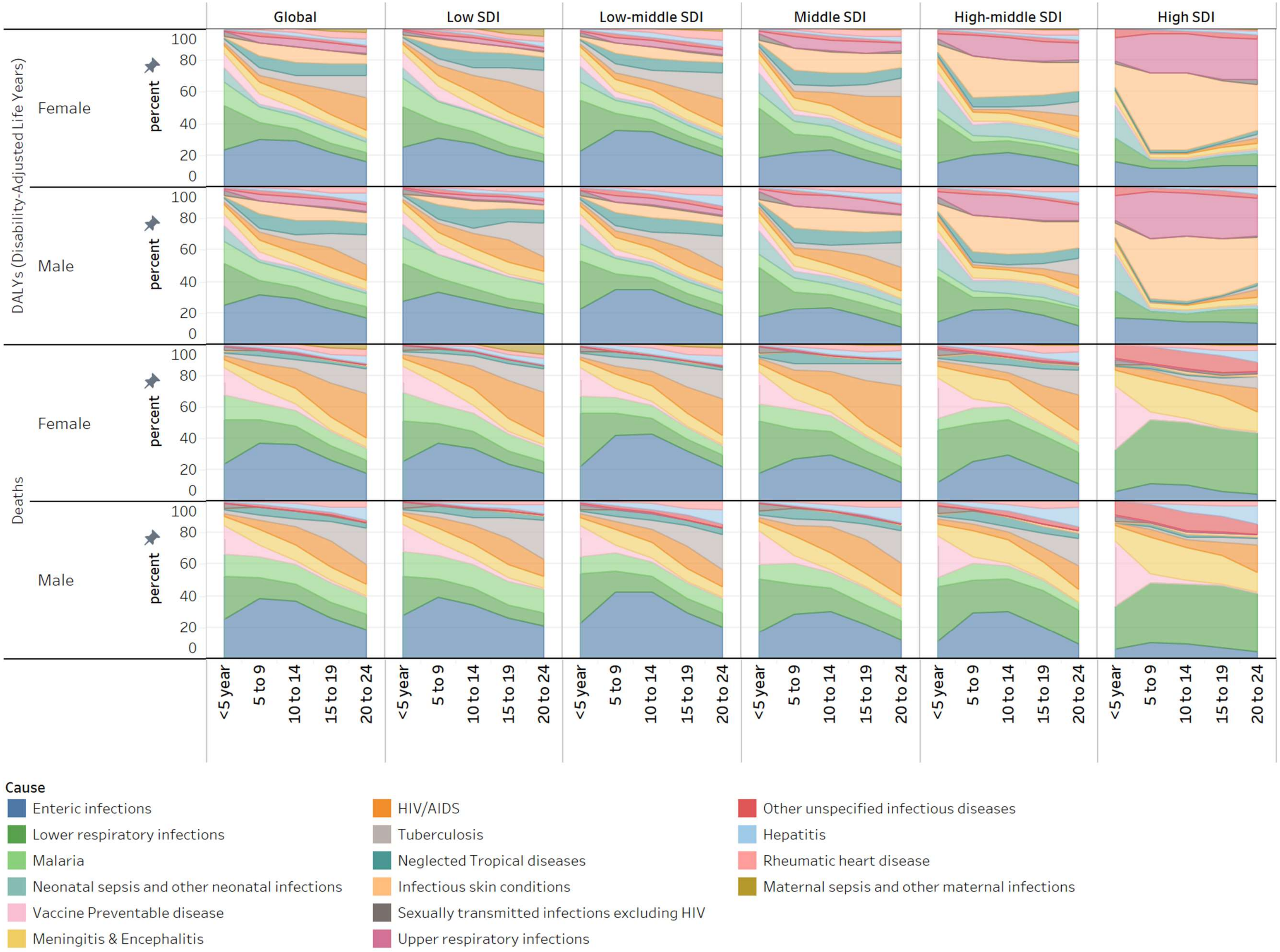

S10. Communicable disease burden (DALYs) among 0-24-year-olds borne by each age group over time as (A) proportion (B) number.

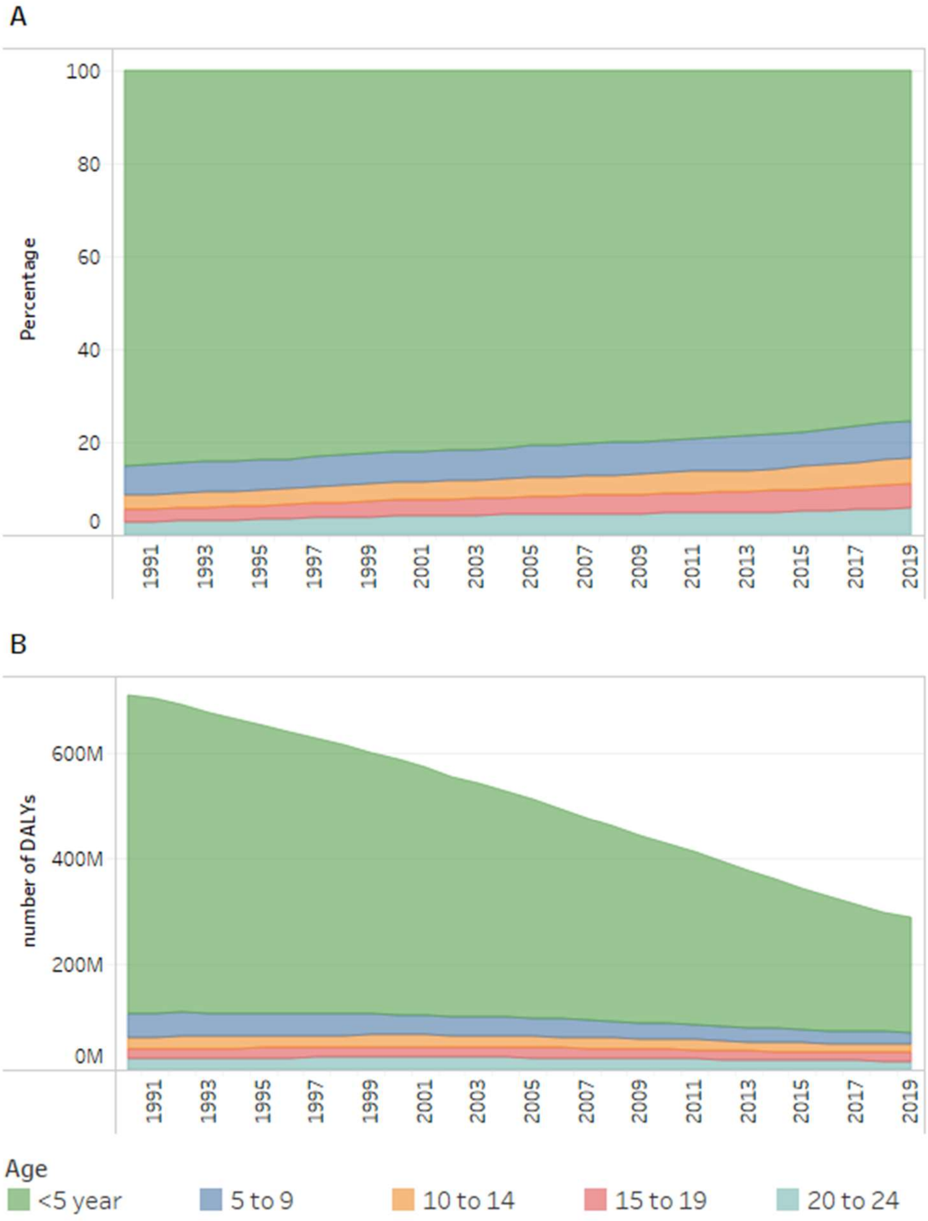

S11 Deaths among 0-24-year-olds borne by each age group over time as (A) proportion (B) number.

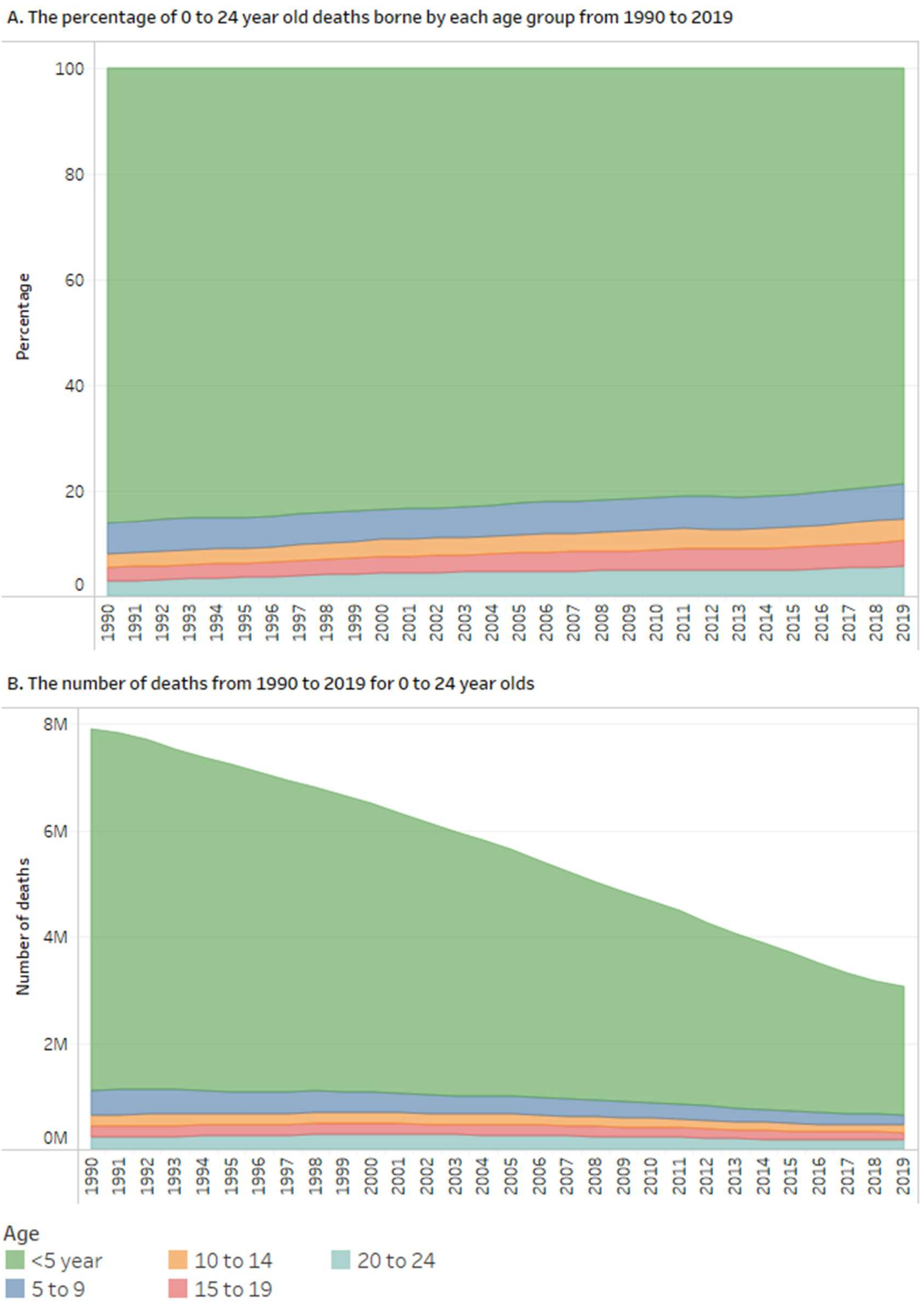

S12. (A) Communicable disease DALYs by cause for under 5 year olds by sex in 2019, grouped by SDI. The shading ranges from green which indicates a low number of DALYs per 100,000 for that country with in the disease, while the highest rates are shaded in a dark orange colour and indicates a country has a large DALY burden.

Under 5 years, DALYs/100,000, for males and females, 2019

SDI	Country	Total commuincable		Enteric infections		Lower respiratory infections		Malaria		Neonatal sepsis and other neonatal infections		Vaccine Preventable disease		Meningitis & Encephalitis	
		Female	Male	Female	Male	Female	Male	Female	Male	Female	Male	Female	Male	Female	Male
Low SDI	Afghanistan	38,220	35,157	6,460	5,954	19,632	16,995	120	155	1,397	1,932	6,643	6,094	1,581	1,490
	Benin	95,402	98,354	15,227	15,527	19,729	21,162	32,258	28,865	7,094	8,105	12,399	13,165	4,073	5,939
	Burkina Faso	134,167	131,923	27,282	29,690	36,222	35,383	37,878	34,062	9,247	9,285	7,703	6,590	7,162	8,077
	Burundi	70,633	80,352	17,709	31,553	11,679	9,649	26,229	23,272	5,602	6,983	2,718	2,156	2,020	1,972
	Central African Republic	150,560	178,766	41,740	74,669	31,123	37,146	29,026	20,245	5,840	6,823	15,096	12,587	4,588	3,735
	Chad	154,551	165,060	68,657	69,075	32,702	39,572	13,203	10,847	6,865	9,382	15,311	14,582	7,142	9,917
	Côte d'Ivoire	82,453	82,998	11,046	12,861	18,930	20,106	33,737	28,251	6,443	8,012	3,790	3,794	1,955	2,242
	Dem. Rep. of the Congo	67,047	73,927	9,408	18,365	9,414	8,764	30,082	27,336	3,448	4,494	5,173	4,882	1,424	1,190
	Eritrea	43,122	48,233	16,537	19,855	12,633	13,238	410	315	4,185	5,477	2,871	2,353	1,846	1,883
	Ethiopia	49,279	54,110	12,733	14,680	10,469	10,204	6,093	5,892	6,268	8,659	5,581	5,085	2,029	2,560
	Gambia	34,898	37,630	7,527	8,543	7,452	7,941	4,023	3,424	6,048	7,329	3,282	2,952	1,377	1,714
	Guinea	111,814	122,941	15,734	17,936	28,011	31,495	33,575	34,027	6,885	8,646	13,141	11,996	7,156	9,923
	Guinea-Bissau	73,111	80,693	19,288	22,979	9,175	9,025	9,978	10,286	6,126	7,868	19,012	19,475	2,568	3,096
	Haiti	53,989	62,548	17,634	19,352	16,091	20,625	275	280	3,053	3,901	5,119	4,052	4,280	5,627
	Liberia	75,650	77,901	18,165	23,912	8,278	7,403	28,987	23,777	5,082	6,182	4,514	4,440	1,802	1,931
	Madagascar	65,186	68,856	23,038	26,707	13,144	12,537	7,408	5,635	3,622	4,739	6,612	6,642	1,465	1,511
	Malawi	58,045	63,332	10,126	12,882	13,193	14,707	14,980	12,529	4,293	5,984	3,449	3,141	2,760	3,445
	Mali	116,761	131,165	16,973	28,125	29,092	21,647	30,194	30,700	13,698	14,679	11,451	11,886	5,066	13,046
	Mozambique	75,077	79,559	8,922	8,974	13,299	12,187	15,582	16,148	7,351	10,761	3,582	3,489	2,277	2,218
	Nepal	24,280	23,631	3,115	2,845	10,464	9,869	2	3	3,673	3,590	3,675	3,680	823	860
	Niger	154,485	158,057	45,070	48,731	31,185	31,487	32,390	27,839	7,512	8,704	23,296	22,589	9,891	12,844
	Pakistan	41,707	37,236	11,414	10,148	15,170	11,852	115	218	3,739	3,099	3,238	2,834	2,697	4,284
	Papua New Guinea	58,413	60,822	8,776	9,417	28,137	30,060	1,070	631	1,489	2,256	7,255	6,583	2,923	2,614
	Rwanda	42,967	46,282	8,030	8,700	10,653	11,442	11,410	10,990	4,808	6,289	2,303	2,152	1,822	2,155
	Senegal	47,245	48,869	12,996	14,885	9,488	9,846	8,941	7,440	6,297	6,965	4,767	4,520	1,789	2,052
	Sierra Leone	116,163	122,852	15,042	14,208	27,763	32,236	47,380	45,450	7,494	9,535	5,218	4,936	6,157	7,651
	Solomon Islands	27,535	29,843	3,798	4,867	8,013	8,849	129	83	485	852	2,087	1,926	413	360
	Somalia	112,462	122,765	19,716	26,594	29,039	37,472	6,607	4,915	7,860	8,860	31,058	27,256	5,819	5,768
	South Sudan	109,435	119,540	21,169	20,705	27,058	35,014	25,506	21,463	6,080	8,151	9,270	8,054	6,737	9,632
	Togo	72,126	79,381	31,311	41,106	10,692	11,152	17,140	13,445	4,986	5,933	2,362	2,007	1,523	1,524
	Uganda	61,240	68,040	5,666	6,817	8,166	8,901	21,702	22,048	4,817	6,415	7,496	7,666	2,330	3,135
	United Republic of Tanzania	55,404	54,794	4,212	4,438	16,742	15,303	13,049	12,274	5,473	7,225	6,333	5,576	1,750	1,505
	Yemen	24,203	25,176	10,700	14,134	6,566	4,923	160	163	862	906	3,017	2,298	628	461

Country	HIV/AIDS		Tuberculosis		Neglected Tropical diseases		Infectious skin conditions		Sexually transmitted infections excluding HIV		Upper respiratory infections		Other unspecified infectious diseases		Hepatitis		Rheumatic heart disease	
	Female	Male	Female	Male	Female	Male	Female	Male	Female	Male	Female	Male	Female	Male	Female	Male	Female	Male
Afghanistan	128	129	431	489	167	174	142	147	460	525	162	178	552	388	326	493	20	16
Benin	735	712	1,294	1,683	888	1,284	304	266	733	905	150	158	357	269	123	228	39	87
Burkina Faso	714	735	3,420	3,274	1,407	1,791	483	295	1,487	1,732	227	217	689	598	152	94	95	102
Burundi	849	861	2,034	1,904	250	377	352	296	277	322	436	561	378	360	69	55	32	30
Central African Republic	1,402	1,335	14,992	14,124	479	613	341	287	4,409	5,150	710	1,011	607	803	151	185	56	53
Chad	1,041	992	4,134	3,992	1,081	1,485	296	254	3,052	3,569	192	186	640	627	180	490	56	89
Côte d'Ivoire	2,020	2,077	1,192	1,569	626	886	256	240	1,842	2,281	147	150	397	388	49	95	23	44
Dem. Rep. of the Congo	611	613	2,902	2,876	507	502	275	255	3,120	3,802	277	335	356	421	34	72	14	20
Eritrea	740	730	1,441	1,644	302	413	383	319	887	1,021	347	490	446	412	64	54	32	30
Ethiopia	1,748	1,782	820	896	398	637	408	393	1,994	2,457	365	496	302	286	49	58	22	23
Gambia	944	911	625	745	682	830	261	235	2,101	2,448	111	120	405	333	43	79	17	27
Guinea	758	743	2,008	2,570	1,228	1,652	302	264	2,039	2,419	175	168	608	597	138	420	57	84
Guinea-Bissau	806	799	859	972	798	1,000	278	248	3,494	4,152	144	150	481	437	75	152	29	54
Haiti	1,641	1,435	887	816	261	265	326	470	3,745	4,706	241	347	198	492	84	73	153	107
Liberia	340	322	607	589	529	701	248	227	6,578	7,808	143	151	311	285	51	146	15	27
Madagascar	432	430	1,048	1,107	141	169	312	268	5,126	6,230	255	377	2,530	2,458	29	19	25	27
Malawi	2,714	2,796	1,312	1,451	216	233	333	293	3,950	4,995	334	474	307	307	55	74	25	21
Mali	728	697	2,607	2,026	1,219	1,892	366	324	4,276	4,735	189	193	707	917	143	186	52	112
Mozambique	16,407	16,906	2,267	2,415	246	291	456	323	3,712	4,738	425	646	478	378	59	75	13	10
Nepal	233	235	288	246	240	318	159	201	1,115	1,295	193	198	76	93	214	182	10	16
Niger	137	134	2,256	2,448	1,149	1,723	304	263	311	363	243	199	537	498	129	114	74	122
Pakistan	180	179	2,013	1,008	273	363	186	218	1,383	1,323	195	199	627	1,078	385	368	92	65
Papua New Guinea	1,272	1,299	799	719	317	246	339	397	5,042	5,586	151	157	291	606	104	33	448	217
Rwanda	589	574	788	957	177	248	374	324	1,291	1,605	295	414	349	349	56	65	22	19
Senegal	233	224	529	600	691	805	265	236	625	740	139	151	439	357	29	25	17	25
Sierra Leone	311	288	2,635	3,029	1,111	1,725	355	295	1,621	2,064	152	172	681	749	191	415	53	98
Solomon Islands	375	379	38	55	2,369	1,499	314	340	8,975	9,958	145	150	323	378	29	15	42	134
Somalia	849	835	5,564	4,361	510	777	400	311	2,732	3,043	1,371	1,548	715	654	160	314	64	58
South Sudan	1,092	1,063	3,235	3,906	1,707	2,876	407	328	5,756	6,617	571	789	667	697	120	197	58	45
Togo	966	872	973	885	592	769	244	225	778	888	142	146	352	317	47	79	19	33
Uganda	2,249	2,168	973	1,284	173	248	333	297	6,637	8,213	285	413	350	373	43	43	19	20
United Republic of Tanzania	1,281	1,243	1,570	1,544	293	347	507	387	3,227	3,950	370	477	490	431	70	65	36	28
Yemen	168	169	145	121	399	378	138	140	749	827	175	186	324	317	102	114	71	40

Under 5 years, DALYs/100,000, for males and females, 2019

SDI	Country	Total communicable		Enteric infections		Lower respiratory infections		Malaria		Neonatal sepsis and other neonatal infections		Vaccine Preventable disease		Meningitis & Encephalitis		HIV/AIDS		Tuberculosis		Neglected Tropical diseases		Infectious skin conditions		Sexually transmitted infections excluding HIV		Upper respiratory infections		Other unspecified infectious diseases		Hepatitis		Rheumatic heart disease	
		Female	Male	Female	Male	Female	Male	Female	Male	Female	Male	Female	Male	Female	Male	Female	Male	Female	Male	Female	Male	Female	Male	Female	Male	Female	Male	Female	Male	Female	Male	Female	Male
Low-middle SDI	Angola	52,739	59,428	11,029	15,425	9,691	9,375	10,363	10,589	3,687	4,593	7,261	7,059	2,095	1,794	1,423	1,453	2,474	2,943	233	342	299	265	3,408	4,551	284	375	440	583	35	60	16	21
	Bangladesh	19,366	18,235	2,825	2,644	9,174	6,706	1	1	3,161	5,103	1,550	1,271	274	140	24	24	324	272	241	221	192	246	1,132	1,268	205	210	102	93	150	30	11	4
	Belize	6,950	8,738	1,035	1,386	2,434	3,214	1	1	2,208	2,710	42	55	316	347	267	319	44	82	95	95	253	260	5	0	170	192	71	67	6	6	5	5
	Bhutan	19,213	18,517	3,348	2,315	6,111	7,170	4	5	2,535	1,978	416	285	1,781	1,689	408	414	489	267	419	427	246	236	2,555	2,909	226	223	331	294	296	269	49	35
	Bolivia	20,634	23,961	2,246	2,016	8,815	9,574	3	3	4,591	7,290	1,324	1,082	548	629	814	815	364	395	158	170	256	254	1,132	1,327	226	228	137	155	11	14	11	10
	Cabo Verde	11,180	10,762	2,009	1,989	2,472	2,407	840	921	2,831	2,582	403	358	458	465	597	530	182	114	391	393	219	203	463	548	122	128	175	105	12	13	5	8
	Cambodia	22,919	29,553	1,771	2,656	14,565	18,751	86	46	2,532	3,574	1,500	1,421	661	913	288	291	539	627	187	219	300	309	67	78	156	171	209	426	47	31	11	40
	Cameroon	85,898	93,326	25,151	35,357	16,366	16,178	23,067	18,174	5,136	6,531	7,121	6,833	2,389	2,744	1,519	1,490	1,115	1,168	578	824	254	237	2,583	3,016	153	160	391	369	55	209	18	36
	Comoros	43,925	41,256	7,894	11,543	16,382	11,325	1,978	1,438	4,426	5,257	7,188	5,869	2,364	1,998	9	11	811	690	150	158	359	298	1,672	1,961	255	319	380	346	40	32	16	11
	Congo	37,396	47,752	7,307	16,533	3,760	4,692	10,067	9,081	3,340	3,822	4,448	4,173	794	760	3,969	4,173	818	1,071	131	164	258	255	2,002	2,439	211	240	260	311	22	25	9	11
	Dem. People's Rep. of Korea	6,346	7,171	1,105	1,343	2,829	3,188	1	1	367	479	356	343	520	562	220	223	134	157	41	43	273	294	213	245	147	155	107	106	21	22	10	10
	Djibouti	40,509	41,785	6,025	8,175	13,124	13,696	912	642	4,960	5,646	8,110	6,177	2,464	2,193	624	611	1,008	1,065	370	560	380	312	1,667	1,791	321	419	468	428	51	52	26	20
	Dominican Republic	15,684	19,529	2,412	2,340	1,984	2,485	0	0	6,755	9,494	1,622	1,577	790	1,142	115	122	123	184	78	80	233	240	1,330	1,589	159	164	61	68	11	29	10	15
	El Salvador	6,519	7,572	1,194	1,385	1,719	2,017	0	0	1,172	1,635	611	559	123	158	1,033	1,099	12	15	104	94	209	213	119	140	165	173	49	70	6	8	3	5
	Eswatini	49,357	55,561	13,185	12,394	12,709	16,853	240	254	3,869	4,595	2,525	2,416	1,018	1,404	9,917	10,104	2,715	3,769	117	144	209	215	2,143	2,555	184	212	477	587	36	46	12	14
	Ghana	53,595	60,446	7,399	8,706	7,690	8,243	21,771	23,360	8,257	10,440	2,729	2,743	1,746	2,336	623	587	701	924	529	784	246	220	1,496	1,772	124	135	224	157	46	20	14	21
	Guatemala	17,743	21,265	4,357	4,850	9,499	11,658	3	3	2,307	3,032	186	227	344	366	188	194	69	83	194	209	235	238	2	1	187	199	152	182	14	16	5	6
	Honduras	10,157	13,582	3,405	4,680	1,560	2,181	2	3	2,175	3,489	774	807	447	703	48	48	40	55	877	687	216	225	148	199	191	222	249	246	23	34	3	3
	India	27,151	21,584	5,934	4,047	10,945	8,568	826	654	3,002	2,710	2,089	1,755	1,488	1,157	144	135	438	356	332	322	375	293	504	523	173	178	275	218	598	647	27	20
	Kenya	41,441	44,853	10,808	15,013	8,855	8,491	6,867	5,993	4,446	4,892	3,208	2,636	1,308	1,311	3,342	3,636	463	441	132	197	337	261	1,143	1,340	264	296	212	292	47	47	10	8
	Kiribati	22,535	23,450	4,613	5,733	4,295	4,161	0	0	1,002	1,725	5,846	5,211	1,912	1,488	277	279	525	549	255	222	326	354	2,811	3,165	163	147	320	301	59	34	129	82
	Kyrgyzstan	7,358	8,740	980	1,163	4,302	5,233	0	0	676	779	18	17	368	419	105	157	200	184	108	118	136	137	1	5	297	343	119	117	43	63	4	4
	Lao PDR	31,070	37,576	4,928	4,744	14,323	18,992	42	41	3,627	5,340	4,590	4,200	1,084	1,468	407	410	891	1,087	196	202	306	317	327	380	179	196	127	143	20	32	22	22
	Lesotho	61,047	72,355	13,579	16,403	19,352	23,196	0	0	4,217	5,732	1,635	1,663	906	1,376	14,067	14,156	3,928	5,656	123	130	205	211	2,318	2,899	191	225	458	629	54	63	15	16
	Maldives	4,964	6,658	918	1,140	823	858	0	0	1,065	2,105	735	671	230	306	11	10	49	58	445	825	294	300	118	135	157	172	103	66	6	4	9	9
	Marshall Islands	14,803	16,365	1,254	1,555	5,269	6,022	0	0	744	1,240	3,792	3,477	374	341	292	299	119	120	200	175	314	340	2,065	2,460	140	144	136	142	11	8	94	41
	Mauritania	41,985	44,485	13,334	15,963	7,989	7,573	8,664	7,544	4,775	5,729	2,634	2,468	925	1,026	6	8	267	272	375	463	222	214	2,323	2,744	124	131	316	305	25	33	7	13
	Micronesia (Fed States of)	11,549	12,862	1,039	1,384	3,225	3,453	0	0	591	992	2,088	1,956	234	216	1,383	1,526	58	65	151	141	307	328	2,168	2,508	140	144	107	111	6	4	52	33
	Mongolia	12,138	12,346	1,550	958	6,530	6,273	0	0	584	1,132	559	468	427	876	10	10	337	233	42	46	152	163	1,629	1,950	128	132	117	49	69	51	3	4
	Morocco	9,361	10,053	2,946	3,279	2,620	2,904	0	0	474	569	1,518	1,393	365	475	215	214	160	39	80	81	139	143	556	661	161	177	83	74	23	35	21	10
	Myanmar	27,699	31,602	3,021	4,630	11,138	12,113	167	42	3,855	5,365	3,950	3,574	2,015	1,792	185	192	756	1,019	655	551	311	322	843	903	179	196	607	775	7	41	10	86
	Nicaragua	8,893	11,443	1,412	1,635	3,720	5,061	6	6	2,106	2,944	78	69	350	445	476	480	47	65	143	125	237	249	114	134	148	155	38	40	14	28	5	6
	Nigeria	126,504	139,698	39,092	44,499	31,755	36,036	25,099	25,312	6,203	8,309	8,504	7,360	7,921	9,406	1,663	1,658	1,843	2,022	1,094	1,380	335	307	2,116	2,496	175	172	552	533	104	131	47	75
	Palestine	4,115	4,399	555	661	638	780	0	0	1,861	1,877	293	265	185	203	31	31	15	7	45	42	139	141	141	160	154	166	44	50	11	13	5	4
	Sao Tome and Principe	21,020	21,256	2,415	2,527	6,058	4,939	2,672	3,201	3,806	4,663	1,707	1,594	1,182	405	5	7	143	122	1,115	1,327	256	225	1,122	1,233	127	129	377	806	22	49	11	30
	Sudan	21,299	24,114	7,215	11,295	4,235	5,034	2,942	314	864	981	2,641	2,442	621	747	186	182	109	181	240	394	139	143	1,479	1,705	153	167	175	214	213	245	87	69
	Tajikistan	20,417	26,194	5,022	5,072	11,817	16,724	0	0	806	1,386	1,210	1,073	446	468	11	10	428	682	59	74	122	127	90	107	267	261	46	53	87	152	4	5
	Timor-Leste	26,674	29,169	4,139	4,456	10,794	11,422	2	2	2,978	4,451	3,223	2,869	1,221	1,433	2,233	2,272	643	690	226	238	323	332	552	649	164	176	133	133	19	20	24	26
	Tuvalu	10,975	11,287	919	965	3,368	3,269	0	0	648	1,142	2,744	2,409	322	268	240	239	52	43	139	120	307	329	1,930	2,235	139	143	103	102	7	5	57	18
	Vanuatu	20,157	21,477	2,845	3,519	6,819	6,876	12	11	778	1,363	4,033	3,657	529	465	335	338	162	153	157	152	302	328	3,789	4,278	141	145	110	120	25	12	120	59
	Venezuela	8,250	9,930	1,504	1,794	2,220	2,951	394	169	2,935	3,667	130	146	329	399	85	91	38	34	138	166	202	207	35	33	162	170	71	94	5	7	2	2
	Zambia	54,213	59,503	11,187	13,725	10,740	11,455	8,222	7,677	4,808	6,005	2,284	2,110	1,870	2,094	9,435	9,374	754	937	268	342	348	305	3,637	4,724	240	341	363	339	40	58	16	18
	Zimbabwe	47,817	54,227	4,855	5,727	18,098	20,238	4,835	5,135	4,129	5,806	4,005	3,618	1,189	1,946	5,864	5,826	2,534	3,187	125	137	204	206	1,570	1,900	220	220	126	163	44	88	20	31

Under 5 years, DALYs/100,000, for males and females, 2019

Middle SDI

	Total communicable		Enteric infections		Lower respiratory infections		Malaria		Neonatal sepsis and other neonatal infections		Vaccine Preventable disease		Meningitis & Encephalitis		HIV/AIDS	
	Female	Male	Female	Male	Female	Male	Female	Male	Female	Male	Female	Male	Female	Male	Female	Male
Albania	5,402	5,269	311	372	3,662	3,384	0	0	46	73	270	213	374	402	6	6
Algeria	4,772	4,976	883	933	1,565	1,601	0	0	667	864	748	633	207	195	38	38
Armenia	6,577	8,817	485	543	3,261	5,001	0	0	2,239	2,527	15	14	122	203	12	6
Azerbaijan	22,076	19,247	1,462	1,049	16,290	14,386	0	0	953	1,089	1,017	879	1,311	798	18	19
Botswana	30,774	32,911	7,538	7,640	10,584	10,761	199	220	2,841	3,962	2,205	1,897	952	1,176	1,120	1,169
Brazil	7,948	10,257	1,180	1,494	2,445	3,106	13	7	2,606	3,699	118	120	344	457	137	171
China	3,320	3,774	329	354	1,453	1,731	0	0	318	368	157	143	257	309	63	63
Colombia	6,516	7,535	823	835	2,215	2,345	36	19	1,961	2,788	90	97	342	360	70	86
Costa Rica	2,239	2,639	456	518	632	846	0	0	367	367	15	15	183	212	65	86
Cuba	2,430	2,683	341	400	682	824	0	0	569	556	17	21	250	262	18	34
Ecuador	8,333	9,453	838	831	2,899	3,632	1	1	1,767	2,090	1,029	864	270	323	120	126
Egypt	13,891	13,591	7,141	6,378	5,037	5,160	0	0	365	505	361	363	441	564	4	4
Equatorial Guinea	42,337	46,079	1,991	3,040	3,336	3,654	16,929	16,556	2,739	3,253	2,917	3,405	832	931	6,014	5,710
Fiji	12,423	14,462	2,314	3,490	4,296	4,703	0	0	1,466	1,825	1,544	1,321	453	579	131	133
Gabon	26,199	33,605	2,794	6,073	2,716	4,137	10,316	10,234	2,843	3,603	2,068	2,754	716	965	2,058	1,926
Grenada	6,592	7,158	620	591	2,847	2,721	0	0	2,039	2,721	43	29	254	179	43	74
Guyana	10,169	13,318	2,104	2,534	2,884	3,612	277	133	3,158	5,374	36	21	467	449	419	321
Indonesia	17,329	21,335	4,286	4,744	3,880	4,188	33	20	1,891	3,157	1,951	1,952	952	1,746	400	447
Iran (Islamic Republic of)	2,851	3,178	610	666	687	769	1	1	521	678	178	157	148	153	179	172
Iraq	8,191	7,880	1,174	1,120	1,936	1,915	0	0	2,320	2,288	1,439	1,212	553	545	44	44
Jamaica	5,675	6,177	520	545	879	956	0	0	3,055	3,485	10	11	302	264	297	278
Mexico	6,345	7,766	997	1,055	2,092	2,489	0	0	2,222	3,077	84	78	232	275	63	66
Namibia	32,540	33,604	6,785	5,805	7,421	8,153	2,079	2,037	3,260	4,128	1,234	1,059	621	717	6,939	6,849
Nauru	15,469	15,720	1,243	1,300	8,606	8,293	0	0	788	1,289	559	505	820	656	260	262
Panama	7,992	9,326	1,928	1,935	2,832	3,607	2	2	1,776	2,227	37	36	353	392	164	183
Paraguay	9,974	10,621	1,260	1,101	2,111	1,895	0	0	1,014	1,467	465	413	338	320	1,255	1,284
Peru	11,950	13,815	1,411	1,522	2,995	3,329	9	9	3,479	4,797	939	782	340	460	1,628	1,618
Philippines	18,746	23,293	2,252	2,970	5,628	7,671	8	4	4,177	5,252	2,963	3,014	809	1,155	454	470
Saint Lucia	5,607	5,137	751	768	1,388	1,476	0	0	2,251	1,691	27	56	399	344	46	31
Samoa	9,311	11,006	760	919	2,028	2,342	0	0	586	1,008	2,870	3,305	188	207	253	271
South Africa	26,804	32,137	6,289	7,235	6,429	7,261	59	75	2,724	3,874	2,316	2,439	505	705	2,969	3,645
St Vincent & the Grenadines	7,978	7,441	1,167	927	1,779	1,855	0	0	3,741	3,070	33	26	347	458	198	196
Suriname	13,140	15,374	2,223	2,028	2,529	3,147	10	7	4,667	6,115	393	351	683	856	426	325
Syrian Arab Republic	5,861	6,118	729	728	2,012	2,128	0	0	631	838	1,378	1,180	432	496	14	15
Thailand	4,921	5,902	683	801	859	1,227	1	1	1,241	1,652	677	632	175	233	495	515
Tokelau	8,082	7,435	792	724	2,073	1,493	0	0	527	936	2,195	1,599	299	148	252	257
Tonga	10,838	11,252	576	548	1,960	2,804	0	0	1,665	1,577	2,547	2,332	1,184	1,279	103	104
Tunisia	2,973	3,778	494	524	734	1,143	0	0	555	724	433	483	143	217	74	74
Turkmenistan	16,127	19,600	1,021	1,152	12,400	15,421	0	0	1,065	1,207	17	20	707	861	9	14
Uzbekistan	17,720	20,331	410	466	14,693	16,782	0	0	966	1,240	14	12	516	605	9	20
Viet Nam	7,695	10,167	561	783	2,188	3,752	4	4	2,351	2,561	1,082	985	550	969	6	6

	Tuberculosis		Neglected Tropical diseases		Infectious skin conditions		Sexually transmitted infections excluding HIV		Upper respiratory infections		Other unspecified infectious diseases		Hepatitis		Rheumatic heart disease	
	Female	Male	Female	Male	Female	Male	Female	Male	Female	Male	Female	Male	Female	Male	Female	Male
Albania	8	5	44	53	151	154	112	144	216	302	161	138	5	7	35	16
Algeria	18	21	59	61	138	141	157	176	153	165	105	108	19	30	14	9
Armenia	58	80	70	92	125	130	0	0	115	126	66	80	4	13	3	3
Azerbaijan	286	255	42	55	125	130	194	207	256	253	84	80	34	44	3	3
Botswana	1,904	2,039	117	126	185	190	2,519	2,984	181	202	377	488	32	39	21	19
Brazil	55	61	201	251	360	356	105	118	230	252	136	146	10	15	7	6
China	42	46	27	27	261	286	186	201	138	151	71	74	14	16	5	5
Colombia	41	40	320	284	245	241	27	39	205	216	132	174	6	9	2	2
Costa Rica	6	8	53	56	208	215	7	20	161	171	78	116	4	5	4	4
Cuba	3	3	49	52	257	270	0	0	175	176	62	79	3	3	4	4
Ecuador	46	72	67	68	250	253	788	939	202	197	45	45	4	6	6	5
Egypt	5	9	42	53	148	152	89	105	117	133	82	80	25	53	35	31
Equatorial Guinea	409	555	127	143	240	245	6,339	8,006	204	228	247	331	8	16	5	7
Fiji	70	79	193	161	340	352	1,090	1,223	137	140	277	368	8	5	105	82
Gabon	329	708	140	230	260	266	1,514	2,094	212	250	217	336	10	20	6	9
Grenada	11	10	149	123	324	372	1	6	167	179	83	141	5	5	6	5
Guyana	83	108	199	174	251	249	20	26	179	174	76	128	9	10	7	6
Indonesia	545	608	1,436	1,721	331	321	1,315	1,856	186	196	96	336	22	39	5	3
Iran (Islamic Republic of)	11	13	38	49	140	144	68	79	162	175	87	98	11	15	10	9
Iraq	44	37	79	87	132	136	196	220	154	167	85	75	25	27	10	7
Jamaica	3	6	101	105	234	245	1	8	168	164	94	103	2	3	8	6
Mexico	19	21	70	76	224	228	2	3	201	223	99	127	35	44	4	4
Namibia	1,405	1,502	174	161	193	197	1,830	2,314	180	198	390	456	20	19	9	10
Nauru	113	113	172	147	317	340	2,116	2,443	138	143	129	149	10	7	198	73
Panama	237	184	64	71	225	227	13	10	212	276	138	164	5	7	6	7
Paraguay	66	59	96	117	332	323	2,728	3,337	209	222	93	77	2	3	3	3
Peru	98	100	62	93	246	249	483	565	190	201	62	81	5	5	4	4
Philippines	458	640	630	613	345	363	735	806	177	196	82	103	5	10	23	26
Saint Lucia	22	24	176	106	275	287	29	26	169	191	64	127	3	3	7	4
Samoa	31	19	108	99	303	325	1,929	2,258	140	143	86	87	5	4	24	20
South Africa	1,073	1,281	41	46	188	191	3,378	4,367	187	202	629	794	10	15	7	8
St Vincent & the Grenadines	16	31	118	99	286	321	1	58	190	208	92	182	4	3	6	7
Suriname	24	39	412	331	373	458	1,071	1,284	169	174	149	243	6	10	5	5
Syrian Arab Republic	22	18	97	109	137	140	142	164	154	170	59	65	8	16	47	52
Thailand	34	43	96	98	294	300	56	65	215	226	88	97	3	4	6	7
Tokelau	27	15	131	110	302	317	1,184	1,605	137	140	89	78	5	4	68	8
Tonga	43	51	520	291	311	329	1,421	1,521	139	143	245	206	88	46	36	23
Tunisia	6	12	42	53	138	142	113	127	154	165	71	82	10	23	5	8
Turkmenistan	317	308	67	74	127	130	0	0	220	182	112	137	60	90	5	4
Uzbekistan	270	280	108	105	125	129	0	0	370	406	192	216	46	66	4	4
Viet Nam	109	115	98	152	293	300	230	332	145	154	67	40	8	7	3	8

Under 5 years, DALYs/100,000, for males and females, 2019

SDI	Country	Total commuincable		Enteric infections		Lower respiratory infections		Malaria		Neonatal sepsis and other neonatal infections		Vaccine Preventable disease		Meningitis & Encephalitis	
		Female	Male	Female	Male	Female	Male	Female	Male	Female	Male	Female	Male	Female	Male
High-middle SDI	American Samoa	7,656	7,593	710	765	1,974	1,582	0	0	199	491	1,793	1,565	331	245
	Antigua and Barbuda	3,811	5,133	707	585	1,688	1,774	0	0	317	1,501	37	18	341	388
	Argentina	3,416	4,124	413	518	999	1,297	0	0	1,068	1,354	85	85	212	274
	Bahamas	5,238	5,944	480	558	1,422	1,999	0	0	2,123	2,164	16	32	249	295
	Bahrain	1,717	1,693	432	451	299	279	0	0	168	162	160	127	79	70
	Barbados	5,783	5,673	543	506	1,325	1,356	0	0	2,768	2,622	32	25	348	318
	Belarus	1,966	2,436	235	234	341	449	0	0	573	720	7	11	416	550
	Bosnia and Herzegovina	1,842	2,003	322	366	232	299	0	0	203	274	577	507	101	122
	Bulgaria	2,937	3,633	383	415	1,580	2,110	0	0	241	310	20	16	287	345
	Chile	1,751	1,841	248	277	414	482	0	0	417	454	65	79	115	140
	Cook Islands	3,473	3,924	419	435	515	643	0	0	256	361	440	443	23	21
	Croatia	1,074	1,292	248	295	112	149	0	0	312	434	16	15	69	73
	Dominica	12,609	13,418	968	924	2,992	3,667	0	0	4,795	4,788	786	614	755	727
	Georgia	3,919	4,928	413	441	1,130	1,493	0	0	1,542	1,975	38	29	232	280
	Greece	1,022	997	227	260	238	257	0	0	24	47	4	5	70	75
	Greenland	2,879	3,303	248	315	478	565	0	0	84	261	759	638	302	547
	Hungary	1,192	1,400	284	333	290	356	0	0	148	213	11	10	86	99
	Israel	1,030	1,055	251	285	126	136	0	0	121	171	17	16	60	66
	Italy	1,029	1,033	229	260	84	104	0	0	195	251	6	5	52	57
	Jordan	6,103	6,361	605	582	2,282	2,207	0	0	1,542	1,962	753	675	243	212
	Kazakhstan	4,917	5,927	343	343	2,403	2,989	0	0	935	1,158	19	20	550	656
	Lebanon	4,187	4,223	687	678	768	636	0	0	607	730	931	899	122	119
	Libya	3,716	3,643	718	758	1,009	929	0	0	396	461	639	521	147	142
	Malaysia	4,570	5,129	576	626	589	756	2	2	820	1,064	561	540	170	222
	Malta	1,210	1,159	201	220	280	313	0	0	121	77	14	15	104	118
	Mauritius	4,475	6,177	845	882	935	1,283	0	0	1,687	2,877	34	29	238	352
	Montenegro	1,828	1,974	205	238	343	367	0	0	402	482	329	285	52	80
	Niue	10,511	12,390	764	1,055	4,702	5,715	0	0	716	939	919	1,028	260	360
	North Macedonia	2,953	3,235	375	453	758	858	0	0	493	606	663	600	164	181
	Northern Mariana Islands	6,244	7,285	748	767	1,596	2,184	0	0	116	309	993	1,092	224	194
	Oman	3,424	3,246	557	636	988	704	0	0	474	556	429	336	350	417
	Palau	13,955	13,342	889	1,059	8,694	6,437	0	0	457	1,204	671	968	281	31
	Poland	1,135	1,299	229	258	270	339	0	0	202	237	12	11	78	81
	Portugal	1,226	1,326	257	281	180	219	0	0	250	367	11	12	56	77
	Republic of Moldova	6,409	7,916	351	340	2,951	3,720	0	0	2,314	3,022	18	16	226	271
	Romania	4,152	5,078	310	363	2,997	3,834	0	0	80	105	24	23	203	207
	Russian Federation	3,156	3,721	379	373	924	1,122	0	0	679	822	20	27	245	283
	Saint Kitts and Nevis	6,468	5,767	1,155	1,081	1,979	1,688	0	0	808	715	24	26	562	247
	Saudi Arabia	2,761	2,953	542	556	172	145	0	0	1,235	1,458	240	212	69	54
	Serbia	1,470	1,622	202	276	238	271	0	0	164	208	335	295	73	102
	Seychelles	7,188	7,655	732	719	2,261	2,373	0	0	2,208	2,470	891	731	208	295
	Spain	1,205	1,211	238	277	104	99	0	0	279	337	23	20	73	77
	Sri Lanka	3,660	4,388	602	674	623	731	0	0	1,065	1,518	377	349	246	306
	Trinidad and Tobago	4,544	4,992	608	651	1,160	1,411	0	0	1,647	1,881	30	25	242	216
	Turkey	4,206	4,885	692	785	910	1,235	0	0	1,358	1,565	452	417	171	195
	Ukraine	2,998	3,154	252	265	725	709	0	0	880	1,057	71	61	475	467
	United States Virgin Islands	3,444	3,596	403	361	307	357	0	0	602	725	841	757	79	76
	Uruguay	2,711	3,357	430	382	784	1,057	0	0	799	1,261	26	39	154	166

SDI	Country	HIV/AIDS		Tuberculosis		Neglected Tropical diseases		Infectious skin conditions		Sexually transmitted infections excluding HIV		Upper respiratory infections		Other unspecified infectious diseases		Hepatitis		Rheumatic heart disease	
		Female	Male	Female	Male	Female	Male	Female	Male	Female	Male	Female	Male	Female	Male	Female	Male	Female	Male
High-middle SDI	American Samoa	61	62	10	8	134	89	304	364	1,889	2,143	136	139	75	125	5	3	34	12
	Antigua and Barbuda	115	146	14	12	86	106	268	304	1	13	167	173	58	102	5	5	7	5
	Argentina	36	37	22	19	61	81	224	119	39	39	181	198	69	92	5	8	4	4
	Bahamas	308	288	18	28	73	70	285	260	2	3	186	163	68	76	4	4	4	4
	Bahrain	9	9	6	6	36	36	167	155	155	178	153	164	41	48	9	7	2	2
	Barbados	79	94	7	6	102	84	334	376	18	11	174	166	46	103	3	3	4	3
	Belarus	23	35	14	15	23	24	116	119	1	2	119	143	79	95	21	39	0	0
	Bosnia and Herzegovina	1	1	3	4	35	35	150	153	75	91	100	107	39	40	2	4	1	0
	Bulgaria	32	9	9	12	38	39	150	155	0	1	106	111	78	89	12	21	1	2
	Chile	16	16	11	7	4	5	221	113	1	3	181	199	55	59	3	4	1	1
	Cook Islands	232	251	3	4	91	86	289	303	1,026	1,192	135	138	39	41	2	2	4	3
	Croatia	8	6	2	1	23	22	147	151	0	0	99	107	36	36	2	2	1	1
	Dominica	151	313	60	89	157	103	377	402	1,242	1,464	222	184	90	132	7	5	8	5
	Georgia	8	32	117	118	97	121	125	129	0	0	83	93	87	134	12	55	36	27
	Greece	11	5	2	2	19	24	223	98	0	0	179	197	21	22	3	4	1	1
	Greenland	448	454	16	37	20	17	212	112	74	83	199	213	33	59	3	2	2	1
	Hungary	5	5	2	1	27	27	149	153	0	0	119	129	68	69	2	2	1	1
	Israel	27	16	2	1	11	14	192	91	0	0	174	193	46	62	1	2	1	2
	Italy	12	8	2	1	8	7	226	101	0	0	179	199	28	33	4	4	3	2
	Jordan	42	42	2	2	40	44	139	142	266	307	123	134	49	41	15	12	1	1
	Kazakhstan	18	15	98	80	70	82	124	127	1	4	120	128	220	301	15	24	1	1
	Lebanon	246	254	7	7	28	32	136	139	120	135	153	165	362	400	18	26	1	5
	Libya	108	109	15	20	76	74	139	142	137	154	153	164	80	79	17	20	81	69
	Malaysia	1,131	1,159	24	22	118	128	286	296	54	62	162	170	70	74	3	4	3	3
	Malta	8	4	1	1	34	45	226	101	0	0	186	211	31	51	2	1	1	1
	Mauritius	47	75	7	9	129	117	287	294	0	1	163	170	97	78	2	5	4	5
	Montenegro	64	64	3	3	41	55	146	150	100	110	106	105	34	31	2	2	0	1
	Niue	237	249	39	66	136	123	307	326	2,169	2,184	137	141	95	126	6	5	24	76
	North Macedonia	42	43	10	9	44	54	146	151	103	117	99	106	49	47	5	9	1	1
	Northern Mariana Islands	111	117	21	18	121	107	329	320	1,700	1,930	134	137	107	93	5	4	38	12
	Oman	33	34	8	8	75	74	141	155	39	46	151	163	146	108	30	6	2	2
	Palau	239	241	23	32	111	105	290	306	2,062	2,726	137	139	70	59	7	11	23	23
	Poland	11	15	2	2	31	31	147	150	0	0	104	112	45	57	3	5	1	1
	Portugal	29	21	3	3	13	13	222	96	0	1	176	197	28	38	1	2	1	1
	Republic of Moldova	7	5	76	63	28	27	129	130	0	1	141	124	167	193	3	4	1	1
	Romania	35	25	109	101	44	45	160	163	1	3	100	110	81	92	6	6	1	1
	Russian Federation	316	411	39	33	23	32	127	132	4	7	151	187	234	277	12	14	1	1
	Saint Kitts and Nevis	1,280	1,303	17	18	100	67	279	294	12	19	170	212	74	94	5	3	2	1
	Saudi Arabia	96	95	8	8	31	34	137	141	48	54	152	163	25	27	5	4	1	1
	Serbia	58	59	2	1	30	29	149	152	75	86	100	107	41	33	3	3	0	0
	Seychelles	67	66	19	17	93	110	287	295	117	133	163	172	135	265	3	5	4	4
	Spain	19	14	2	1	12	25	233	108	0	0	181	200	36	51	2	2	1	1
	Sri Lanka	24	24	22	24	152	192	274	282	48	55	168	175	55	53	2	3	1	2
	Trinidad and Tobago	277	196	15	16	81	94	238	239	0	1	167	164	72	90	3	3	5	6
	Turkey	36	36	16	22	46	44	140	143	97	110	184	192	87	112	17	24	2	3
	Ukraine	101	83	56	43	26	32	117	121	0	0	133	152	146	151	13	14	1	1
	United States Virgin Islands	56	52	6	3	84	66	239	260	609	722	166	162	48	54	3	2	1	1
	Uruguay	11	19	11	8	45	50	217	117	5	4	180	197	44	53	2	3	1	1

Under 5 years, DALYs/100,000, for males and females, 2019

		Total communicable		Enteric infections		Lower respiratory infections		Malaria		Neonatal sepsis and other neonatal infections		Vaccine Preventable disease		Meningitis & Encephalitis	
		Female	Male	Female	Male	Female	Male	Female	Male	Female	Male	Female	Male	Female	Male
High SDI	Andorra	1,120	968	214	266	142	77	0	0	68	102	41	32	58	36
	Australia	947	972	109	150	147	182	0	0	87	99	21	19	71	72
	Austria	993	944	235	277	86	102	0	0	123	103	20	17	62	83
	Belgium	1,172	1,185	291	329	108	147	0	0	162	160	18	21	91	133
	Bermuda	2,077	1,347	358	338	510	284	0	0	353	52	33	31	250	65
	Brunei Darussalam	3,446	3,691	98	135	1,020	1,193	0	0	1,090	1,054	174	143	152	230
	Canada	1,220	1,190	170	304	135	156	0	0	185	211	27	24	65	90
	Cyprus	982	904	255	296	89	87	0	0	61	79	30	25	50	46
	Czechia	994	1,265	239	286	161	264	0	0	184	246	14	12	60	81
	Denmark	1,032	954	303	327	95	120	0	0	66	60	19	13	59	70
	Estonia	1,219	1,363	290	273	386	428	0	0	98	207	3	3	104	102
	Finland	837	806	219	249	39	48	0	0	90	101	19	17	28	37
	France	1,100	1,100	221	257	81	82	0	0	224	286	18	17	73	77
	Germany	995	927	252	278	79	88	0	0	107	133	12	12	44	53
	Guam	8,287	8,888	684	747	2,526	2,306	0	0	731	1,367	1,151	1,090	293	316
	Iceland	1,039	897	271	293	145	109	0	0	85	68	20	18	61	73
	Ireland	909	900	217	255	85	95	0	0	85	124	12	11	54	64
	Japan	873	838	55	65	197	228	0	0	72	93	11	10	44	45
	Kuwait	1,998	2,085	425	457	876	940	0	0	193	176	9	9	105	109
	Latvia	1,507	1,656	275	282	437	479	0	0	205	250	7	16	185	130
	Lithuania	1,706	1,756	296	289	512	525	0	0	419	413	6	7	140	134
	Luxembourg	1,009	950	306	327	90	92	0	0	40	60	14	15	51	49
	Monaco	1,133	1,046	219	247	166	139	0	0	184	178	20	13	35	64
	Netherlands	1,279	1,288	209	240	82	101	0	0	416	432	20	21	104	118
	New Zealand	1,279	1,367	188	240	233	245	0	0	205	280	21	16	98	119
	Norway	902	832	237	273	48	65	0	0	84	74	10	12	49	55
	Puerto Rico	3,014	3,600	461	499	523	633	0	0	1,297	1,755	23	20	130	94
	Qatar	1,643	1,766	417	470	282	340	0	0	48	59	252	221	124	74
	Republic of Korea	1,254	1,231	49	66	104	113	0	0	277	359	95	79	57	66
	San Marino	1,716	1,426	233	250	89	112	0	0	240	262	204	169	142	96
	Singapore	961	865	39	40	294	336	0	0	88	73	15	12	49	41
	Slovakia	1,762	2,035	266	328	706	835	0	0	76	105	190	157	108	166
	Slovenia	864	887	212	243	84	87	0	0	212	181	14	12	33	21
	Sweden	996	974	258	279	84	96	0	0	111	179	11	18	37	42
	Switzerland	998	1,018	281	307	84	106	0	0	135	209	9	9	35	37
	Taiwan (Province of China)	1,529	1,660	316	350	318	369	0	0	246	254	10	9	94	100
	United Arab Emirates	1,677	1,623	386	421	169	106	0	0	191	210	328	291	82	84
	United Kingdom	1,272	1,287	222	254	208	250	0	0	85	116	23	25	93	130
	United States of America	1,210	1,395	151	169	221	288	0	0	305	359	22	20	75	83

		HIV/AIDS		Tuberculosis		Neglected Tropical diseases		Infectious skin conditions		Sexually transmitted infections excluding HIV		Upper respiratory infections		Other unspecified infectious diseases		Hepatitis		Rheumatic heart disease	
		Female	Male	Female	Male	Female	Male	Female	Male	Female	Male	Female	Male	Female	Male	Female	Male	Female	Male
High SDI	Andorra	41	42	1	0	9	7	228	100	68	77	179	199	52	22	17	8	1	0
	Australia	5	3	1	2	17	26	229	117	0	1	180	202	77	97	2	3	1	1
	Austria	9	6	1	1	11	11	223	99	0	0	177	200	45	42	1	1	2	2
	Belgium	18	13	3	1	9	8	222	98	0	0	180	199	66	75	1	1	2	1
	Bermuda	96	107	10	4	34	37	234	234	1	1	166	162	29	30	3	2	1	1
	Brunei Darussalam	75	75	54	33	50	32	221	115	124	130	182	191	179	273	24	85	1	2
	Canada	8	3	1	1	5	6	368	106	0	0	202	219	51	68	1	2	1	1
	Cyprus	1	1	1	2	9	9	220	97	45	45	175	193	43	21	2	1	1	1
	Czechia	6	15	1	1	23	24	147	151	0	0	108	120	48	62	2	2	1	1
	Denmark	8	6	1	1	10	9	256	101	0	0	175	193	39	54	1	1	0	1
	Estonia	15	17	4	3	17	18	112	118	0	0	108	118	80	73	2	2	0	0
	Finland	6	4	1	1	9	9	225	113	0	0	174	190	25	35	1	1	1	1
	France	11	5	2	1	9	8	222	98	0	0	184	206	52	60	1	1	2	1
	Germany	13	8	1	1	5	5	276	113	0	0	176	196	28	37	1	1	1	1
	Guam	263	270	34	20	77	72	320	332	1,962	2,146	133	136	73	65	5	4	35	15
	Iceland	6	4	1	1	10	8	221	98	0	0	178	195	38	29	1	1	2	2
	Ireland	5	4	1	1	8	8	224	99	0	0	175	193	41	46	1	1	1	1
	Japan	7	12	2	1	20	11	227	120	0	0	180	200	52	46	4	6	1	1
	Kuwait	13	5	12	6	27	30	141	141	0	0	152	164	40	43	3	5	1	1
	Latvia	47	43	10	9	37	46	115	121	0	0	108	119	67	149	12	12	0	1
	Lithuania	8	19	16	16	23	23	117	119	0	0	109	122	57	85	2	3	0	1
	Luxembourg	5	3	1	1	11	9	221	97	0	0	176	194	91	101	1	1	1	1
	Monaco	20	22	3	1	9	7	223	98	59	68	174	191	18	16	1	2	1	0
	Netherlands	9	5	1	1	9	8	188	95	0	0	184	209	53	57	1	1	1	1
	New Zealand	4	3	3	1	22	37	230	122	0	0	190	207	83	94	1	1	2	1
	Norway	7	4	1	1	8	9	237	88	0	0	182	200	38	48	1	1	1	1
	Puerto Rico	42	32	3	3	52	55	235	246	1	2	167	163	76	95	3	3	2	1
	Qatar	9	9	5	5	26	17	141	147	141	163	151	163	39	83	4	10	2	3
	Republic of Korea	4	13	13	11	47	9	224	113	152	175	173	192	54	28	3	5	1	1
	San Marino	25	26	2	1	61	27	223	98	57	63	276	229	150	74	11	19	2	1
	Singapore	4	5	3	2	26	16	222	113	0	0	172	190	46	36	2	2	1	1
	Slovakia	12	13	1	2	27	26	147	152	77	88	100	108	46	50	4	4	1	1
	Slovenia	5	12	1	1	22	24	147	153	0	0	98	105	35	46	2	2	1	1
	Sweden	6	5	1	1	8	7	254	101	0	0	182	200	40	45	1	1	1	1
	Switzerland	6	5	1	1	11	6	225	98	0	0	176	193	34	44	1	2	1	1
	Taiwan (Province of China)	8	16	20	15	34	29	275	279	0	1	116	123	86	107	5	5	1	1
	United Arab Emirates	65	65	7	8	39	35	142	145	32	38	152	163	66	43	7	8	13	3
	United Kingdom	14	9	2	2	39	9	222	99	95	116	188	208	78	65	2	3	1	1
	United States of America	12	13	1	1	11	12	99	104	1	1	205	223	103	118	2	2	1	2

S12 (B) Percentage change in DALYs/100,000 for each communicable condition for each location for under 5 year olds by sex. The colours on this heat map are green for values less than 0, the darker the green the greater the reduction in DALYs/100,000 between 1990 and 2019, the orange tones represent positive numbers, an increase in DALYs/ 100,000, with darker shades indicating worse outcome.

<5 year

		Total commuincable		Enteric infections		Lower respiratory infections		Malaria		Neonatal sepsis and other neonatal infections		Vaccine Preventable disease		Meningitis & Encephalitis		HIV/AIDS	
		Female	Male	Female	Male	Female	Male	Female	Male	Female	Male	Female	Male	Female	Male	Female	Male
Low SDI	Afghanistan	-2.7%	-2.7%	-2.4%	-1.9%	-2.6%	-2.7%	-0.4%	-0.1%	-0.3%	-0.3%	-3.0%	-3.0%	-2.7%	-2.7%	6.7%	6.5%
	Benin	-2.0%	-2.0%	-2.3%	-2.4%	-2.2%	-2.2%	-0.7%	-0.6%	-1.1%	-1.2%	-2.6%	-2.5%	-2.3%	-2.0%	6.6%	6.6%
	Burkina Faso	-2.0%	-2.0%	-2.3%	-2.3%	-1.6%	-1.5%	-1.4%	-1.4%	-0.9%	-0.9%	-2.9%	-2.9%	-1.8%	-1.8%	-3.1%	-3.1%
	Burundi	-2.4%	-2.4%	-1.9%	-1.1%	-2.4%	-2.7%	-2.4%	-2.5%	-0.7%	-1.0%	-3.1%	-3.1%	-2.5%	-2.6%	-3.1%	-3.1%
	Central African Republic	-1.4%	-1.4%	-1.4%	-1.2%	-1.5%	-1.4%	-0.4%	-0.7%	0.1%	0.1%	-2.1%	-2.1%	-2.0%	-2.1%	-2.9%	-2.9%
	Chad	-1.6%	-1.6%	-1.0%	-1.0%	-1.6%	-1.5%	-1.7%	-1.9%	-1.3%	-1.0%	-2.6%	-2.6%	-1.4%	-1.1%	-0.7%	-0.8%
	Côte d'Ivoire	-2.0%	-2.1%	-2.3%	-2.4%	-2.0%	-2.0%	-1.4%	-1.7%	-1.2%	-1.1%	-2.9%	-3.0%	-2.4%	-2.3%	-2.6%	-2.6%
	Dem Rep of the Congo	-2.4%	-2.4%	-2.3%	-1.8%	-2.6%	-2.8%	-2.2%	-2.3%	-1.0%	-0.9%	-2.8%	-2.8%	-2.8%	-2.8%	-2.9%	-2.9%
	Eritrea	-2.6%	-2.6%	-2.4%	-2.5%	-2.5%	-2.7%	-2.9%	-2.9%	-0.9%	-0.9%	-3.2%	-3.2%	-2.5%	-2.5%	-2.0%	-2.0%
	Ethiopia	-2.7%	-2.7%	-2.6%	-2.5%	-2.8%	-2.9%	0.1%	0.2%	-1.0%	-1.2%	-3.1%	-3.1%	-2.8%	-2.8%	-2.1%	-2.1%
	Gambia	-2.6%	-2.6%	-2.3%	-2.3%	-2.5%	-2.7%	-3.0%	-3.0%	-1.4%	-1.4%	-3.0%	-3.0%	-2.6%	-2.6%	7.7%	7.5%
	Guinea	-2.2%	-2.1%	-2.4%	-2.3%	-2.3%	-2.3%	-1.2%	-1.0%	-1.2%	-1.2%	-2.8%	-2.8%	-1.9%	-1.6%	2.1%	2.2%
	Guinea-Bissau	-2.5%	-2.5%	-2.2%	-2.3%	-2.6%	-2.8%	-2.7%	-2.7%	-1.5%	-1.3%	-2.6%	-2.6%	-2.7%	-2.8%	0.1%	0.2%
	Haiti	-2.3%	-2.2%	-2.5%	-2.5%	-2.3%	-2.2%	-2.6%	-2.4%	0.3%	-0.0%	-2.5%	-2.2%	-2.3%	-2.1%	-2.5%	-2.6%
	Liberia	-2.7%	-2.7%	-2.7%	-2.6%	-3.0%	-3.1%	-2.1%	-2.3%	-1.8%	-1.8%	-3.1%	-3.1%	-3.0%	-3.1%	-1.5%	-1.6%
	Madagascar	-2.2%	-2.2%	-2.2%	-2.1%	-2.4%	-2.5%	-1.9%	-2.1%	-1.0%	-1.1%	-2.6%	-2.6%	-2.7%	-2.7%	504.3%	504.7%
	Malawi	-2.7%	-2.7%	-2.9%	-2.9%	-2.5%	-2.4%	-2.7%	-2.8%	-1.3%	-1.3%	-3.0%	-3.0%	-2.7%	-2.7%	-2.7%	-2.7%
	Mali	-2.2%	-2.1%	-2.7%	-2.3%	-1.0%	-1.6%	-2.2%	-2.1%	-0.9%	-1.0%	-2.8%	-2.8%	-2.5%	-1.5%	1.0%	0.9%
	Mozambique	-2.6%	-2.6%	-2.9%	-3.0%	-2.6%	-2.7%	-2.7%	-2.5%	-1.1%	-1.0%	-3.1%	-3.1%	-2.7%	-2.7%	14.2%	15.1%
	Nepal	-2.9%	-2.9%	-3.1%	-3.1%	-2.8%	-2.8%	-3.3%	-3.3%	-0.9%	-1.0%	-3.1%	-3.1%	-3.0%	-2.9%	5,676.0%	5,785.6%
	Niger	-2.3%	-2.3%	-2.4%	-2.4%	-2.4%	-2.5%	-1.9%	-1.7%	-1.0%	-0.9%	-2.4%	-2.3%	-2.6%	-2.5%	-2.2%	-2.2%
	Pakistan	-2.3%	-2.4%	-2.2%	-2.3%	-1.7%	-2.0%	-3.0%	-2.9%	-0.7%	-1.1%	-3.1%	-3.1%	-2.1%	-2.1%	19.0%	21.4%
	Papua New Guinea	-1.4%	-1.5%	-1.6%	-1.5%	-1.3%	-1.5%	-1.9%	-2.2%	0.2%	0.2%	-2.1%	-2.2%	-1.9%	-2.1%	467.9%	482.8%
	Rwanda	-2.7%	-2.7%	-3.0%	-3.0%	-2.6%	-2.8%	-2.6%	-2.6%	-1.1%	-1.2%	-3.0%	-3.0%	-2.7%	-2.7%	-2.6%	-2.7%
	Senegal	-2.5%	-2.5%	-2.6%	-2.7%	-2.4%	-2.5%	-2.4%	-2.4%	-1.3%	-1.3%	-2.8%	-2.8%	-2.7%	-2.8%	-1.5%	-1.6%
	Sierra Leone	-2.2%	-2.2%	-2.7%	-2.8%	-2.3%	-2.2%	-1.7%	-1.4%	-1.3%	-1.1%	-2.8%	-2.8%	-2.4%	-2.3%	-0.6%	-0.8%
	Solomon Islands	-1.9%	-1.9%	-2.4%	-2.5%	-2.1%	-2.1%	-2.2%	-2.2%	-0.9%	-1.0%	-2.8%	-2.8%	-2.4%	-2.5%	1.4%	1.5%
	Somalia	-2.1%	-2.0%	-2.0%	-1.4%	-1.8%	-2.0%	-2.6%	-2.7%	-0.3%	-0.6%	-2.3%	-2.4%	-1.4%	-1.4%	28.0%	28.2%
	South Sudan	-1.8%	-1.8%	-1.4%	-1.3%	-2.0%	-2.0%	-1.1%	-1.0%	-0.6%	-0.8%	-2.6%	-2.6%	-1.7%	-1.7%	2.6%	2.6%
	Togo	-2.1%	-2.1%	-1.9%	-2.0%	-2.3%	-2.2%	-1.8%	-1.9%	-1.3%	-1.3%	-3.0%	-3.0%	-2.5%	-2.5%	-1.1%	-1.3%
	Uganda	-2.5%	-2.6%	-2.8%	-2.8%	-2.2%	-2.5%	-2.5%	-2.6%	-0.9%	-1.2%	-2.6%	-2.6%	-2.5%	-2.6%	-3.2%	-3.2%
	United Republic of Tanzania	-2.5%	-2.6%	-3.0%	-3.0%	-2.4%	-2.6%	-2.5%	-2.5%	-0.8%	-0.8%	-2.7%	-2.7%	-2.3%	-2.5%	-3.0%	-3.0%
	Yemen	-2.7%	-2.7%	-2.6%	-2.6%	-2.7%	-2.7%	-3.1%	-3.1%	-0.6%	-0.7%	-3.0%	-3.0%	-2.5%	-2.6%	3.8%	3.9%

		Tuberculosis		Neglected Tropical diseases		Infectious skin conditions		Sexually transmitted infections excluding HIV		Upper respiratory infections		Other unspecified infectious diseases		Hepatitis		Rheumatic heart disease	
		Female	Male	Female	Male	Female	Male	Female	Male	Female	Male	Female	Male	Female	Male	Female	Male
Low SDI	Afghanistan	-2.9%	-2.9%	-2.7%	-2.9%	-0.0%	-0.1%	-0.4%	-0.2%	-0.5%	-0.6%	-1.8%	-2.1%	-2.8%	-2.9%	-2.8%	-2.7%
	Benin	-2.6%	-2.4%	-1.8%	-1.7%	-0.6%	-0.4%	-1.7%	-1.5%	-0.8%	-0.8%	-1.1%	-1.2%	-1.6%	-2.5%	-2.4%	-2.0%
	Burkina Faso	-1.6%	-1.6%	-1.2%	-1.2%	0.4%	0.1%	-0.9%	-0.8%	-0.7%	-1.0%	-0.5%	-0.6%	-1.4%	-2.5%	-1.1%	-1.3%
	Burundi	-2.5%	-2.6%	-2.2%	-2.2%	-0.5%	-0.4%	-1.8%	-1.8%	-2.3%	-2.2%	-2.2%	-2.5%	-2.1%	-2.1%	-2.7%	-2.6%
	Central African Republic	-1.1%	-1.0%	-2.7%	-2.7%	-0.3%	-0.1%	-0.9%	-0.6%	-1.7%	-1.2%	-1.2%	-1.0%	-1.4%	-2.0%	-1.7%	-1.0%
	Chad	-1.8%	-1.6%	-1.5%	-1.6%	-0.5%	-0.3%	-1.4%	-1.4%	-0.7%	-0.8%	-1.4%	-1.6%	-1.0%	-1.8%	-2.1%	-2.0%
	Côte d'Ivoire	-2.2%	-2.0%	-1.7%	-1.8%	-0.6%	-0.3%	-1.7%	-1.7%	-0.6%	-0.8%	-1.3%	-1.7%	-1.7%	-2.6%	-2.1%	-2.1%
	Dem Rep of the Congo	-2.8%	-2.8%	-2.3%	-2.7%	-0.7%	-0.5%	-1.9%	-1.8%	-2.3%	-2.4%	-2.0%	-2.3%	-2.6%	-2.9%	-2.8%	-2.5%
	Eritrea	-2.7%	-2.7%	-2.9%	-2.9%	-0.3%	-0.1%	-1.7%	-1.6%	-2.5%	-2.3%	-2.4%	-2.5%	-2.2%	-2.3%	-2.7%	-2.6%
	Ethiopia	-3.0%	-3.0%	-3.1%	-3.1%	-1.1%	-0.6%	-2.5%	-2.6%	-2.8%	-2.7%	-2.1%	-2.2%	-2.7%	-2.6%	-2.9%	-2.9%
	Gambia	-2.5%	-2.5%	-1.5%	-1.7%	-0.4%	-0.2%	-1.3%	-1.4%	-0.7%	-0.7%	-0.9%	-1.5%	-1.8%	-1.9%	-2.6%	-2.6%
	Guinea	-2.6%	-2.3%	-1.7%	-1.7%	-0.8%	-0.5%	-1.7%	-1.6%	-1.0%	-0.9%	-1.4%	-1.7%	-1.8%	-2.1%	-2.5%	-2.2%
	Guinea-Bissau	-2.8%	-2.9%	-2.0%	-2.3%	-0.8%	-0.6%	-2.1%	-2.1%	-1.1%	-1.1%	-1.3%	-2.0%	-2.2%	-2.8%	-2.8%	-2.8%
	Haiti	-2.6%	-2.7%	-1.4%	-1.2%	-0.6%	-0.4%	-0.4%	-0.5%	-1.9%	-2.0%	-0.7%	-0.7%	-2.8%	-2.5%	-2.6%	-2.3%
	Liberia	-3.1%	-3.1%	-2.6%	-2.7%	-1.3%	-1.1%	-0.8%	-0.8%	-1.2%	-1.2%	-2.2%	-2.6%	-2.8%	-3.0%	-3.1%	-3.0%
	Madagascar	-2.7%	-2.7%	-2.1%	-2.1%	-0.7%	-0.4%	-0.9%	-0.8%	-2.4%	-2.4%	-1.0%	-1.5%	-2.5%	-2.9%	-2.7%	-2.6%
	Malawi	-2.8%	-2.8%	-2.4%	-2.4%	-0.8%	-0.5%	-0.6%	-0.5%	-2.7%	-2.6%	-1.6%	-1.9%	-2.7%	-3.0%	-2.8%	-2.8%
	Mali	-2.0%	-2.1%	-1.7%	-1.5%	-0.5%	-0.3%	-0.7%	-0.8%	-1.2%	-0.9%	-1.4%	-0.9%	-2.1%	-2.8%	-2.4%	-2.0%
	Mozambique	-2.8%	-2.7%	-2.4%	-2.4%	-0.3%	-0.1%	-2.5%	-2.4%	-2.8%	-2.5%	-0.8%	-1.4%	-2.7%	-3.0%	-2.9%	-2.7%
	Nepal	-3.2%	-3.1%	-2.7%	-2.9%	-0.0%	-0.0%	-1.1%	-1.0%	-2.3%	-2.0%	-0.9%	-1.7%	-2.9%	-2.9%	-3.1%	-2.9%
	Niger	-2.7%	-2.6%	-1.9%	-1.8%	-0.8%	-0.5%	-2.1%	-1.9%	-1.3%	-1.0%	-1.8%	-1.8%	-2.3%	-2.6%	-2.6%	-2.4%
	Pakistan	-2.6%	-2.1%	-2.2%	-2.2%	0.0%	0.0%	-1.2%	-1.2%	-1.9%	-1.6%	-1.0%	-1.3%	-2.4%	-2.2%	-2.3%	-1.3%
	Papua New Guinea	-1.9%	-2.0%	-1.1%	-1.2%	0.1%	0.0%	-0.6%	-0.6%	-0.3%	-0.3%	0.4%	0.2%	-1.8%	-2.4%	-0.7%	-0.6%
	Rwanda	-2.9%	-2.9%	-2.3%	-2.3%	-0.4%	-0.4%	-1.4%	-1.3%	-2.2%	-2.2%	-2.2%	-2.4%	-2.3%	-2.8%	-2.8%	-2.8%
	Senegal	-2.8%	-2.8%	-1.9%	-2.1%	-0.7%	-0.5%	-1.7%	-1.7%	-1.0%	-0.8%	-1.3%	-1.5%	-2.7%	-2.9%	-2.8%	-2.9%
	Sierra Leone	-2.4%	-2.4%	-1.9%	-1.9%	-0.6%	-0.3%	-1.5%	-1.4%	-1.0%	-1.0%	-1.1%	-1.4%	-1.6%	-2.5%	-2.6%	-2.4%
	Solomon Islands	-2.5%	-2.4%	-1.4%	-0.6%	-0.0%	-0.1%	0.0%	0.2%	-0.2%	-0.1%	-1.2%	-1.3%	-2.4%	-2.8%	-2.1%	-1.1%
	Somalia	-1.7%	-1.9%	-3.2%	-3.2%	-0.2%	-0.3%	-0.7%	-0.6%	-2.0%	-1.6%	-2.2%	-2.4%	-1.3%	-1.6%	-2.3%	-2.2%
	South Sudan	-2.0%	-2.0%	-2.9%	-2.9%	-0.5%	-0.3%	-0.5%	-0.5%	-2.1%	-1.9%	-2.4%	-2.5%	-1.6%	-1.2%	-2.4%	-2.3%
	Togo	-2.4%	-2.4%	-1.7%	-1.8%	-0.6%	-0.4%	-2.0%	-2.0%	-0.5%	-0.5%	-1.3%	-1.6%	-2.1%	-2.8%	-2.6%	-2.5%
	Uganda	-2.5%	-2.6%	-2.9%	-2.9%	-0.3%	-0.2%	2.1%	2.2%	-2.4%	-2.4%	-2.2%	-2.4%	-1.9%	-2.1%	-2.4%	-2.5%
	United Republic of Tanzania	-2.3%	-2.4%	-2.2%	-2.2%	-0.1%	-0.1%	-2.1%	-2.1%	-2.1%	-2.2%	-1.0%	-1.3%	-2.1%	-2.5%	-2.2%	-2.2%
	Yemen	-3.0%	-2.9%	-1.8%	-2.1%	-0.0%	-0.1%	-0.5%	-0.4%	-0.7%	-0.9%	-0.0%	-0.6%	-3.0%	-3.2%	-2.7%	-2.5%

<5 year

SDI	Country	Total commuincable		Enteric infections		Lower respiratory infections		Malaria		Neonatal sepsis and other neonatal infections		Vaccine Preventable disease		Meningitis & Encephalitis		HIV/AIDS		Tuberculosis		Neglected Tropical diseases		Infectious skin conditions		Sexually transmitted infections excluding HIV		Upper respiratory infections		Other unspecified infectious diseases		Hepatitis		Rheumatic heart disease	
		Female	Male	Female	Male	Female	Male	Female	Male	Female	Male	Female	Male	Female	Male	Female	Male	Female	Male	Female	Male	Female	Male	Female	Male	Female	Male	Female	Male	Female	Male	Female	Male
Low-middle SDI	Angola	-2.7%	-2.8%	-2.9%	-3.0%	-2.9%	-2.9%	-1.6%	-1.2%	-1.5%	-0.9%	-2.8%	-2.7%	-2.8%	-2.7%	13.3%	13.5%	-3.0%	-2.9%	-3.1%	-3.0%	-0.7%	-0.3%	-1.4%	-0.9%	-2.7%	-2.5%	-2.0%	-1.9%	-2.8%	-3.1%	-3.0%	-2.7%
	Bangladesh	-2.8%	-2.8%	-3.1%	-3.1%	-2.7%	-2.8%	-3.2%	-3.2%	-1.4%	-1.1%	-3.2%	-3.2%	-1.4%	-1.7%			-3.1%	-3.2%	-2.6%	-2.7%	-0.2%	-0.7%	-1.7%	-1.7%	-2.2%	-1.7%	-1.9%	-1.7%	-2.8%	-3.1%	-2.5%	-2.6%
	Belize	-2.4%	-2.2%	-2.8%	-2.7%	-2.5%	-2.2%	-3.3%	-3.3%	-0.7%	-0.2%	-2.6%	-2.6%	-2.8%	-2.7%	-2.5%	-2.4%	-3.1%	-3.0%	-1.2%	-0.7%	-0.1%	-0.3%	-2.9%	-2.7%	-0.4%	-1.3%	-1.0%	-0.6%	-3.2%	-3.0%	-2.8%	-2.6%
	Bhutan	-2.9%	-2.9%	-2.7%	-2.6%	-2.7%	-2.8%	-3.3%	-3.3%	-1.3%	-1.6%	-3.3%	-3.3%	-2.5%	-2.5%	9.8%	9.9%	-3.0%	-3.0%	-2.1%	-2.3%	-0.3%	-0.4%	-0.5%	-0.5%	-2.1%	-1.7%	-0.8%	-1.0%	-2.7%	-2.9%	-2.6%	-2.2%
	Bolivia (Plurinational State of)	-2.7%	-2.7%	-3.0%	-2.9%	-2.8%	-2.9%	-3.3%	-3.3%	-0.7%	-0.4%	-2.9%	-2.9%	-2.8%	-2.8%	4.2%	4.1%	-3.2%	-3.2%	-2.1%	-2.2%	-0.4%	-0.4%	-2.1%	-2.0%	-2.4%	-2.3%	-1.0%	-1.2%	-3.0%	-3.0%	-2.9%	-2.8%
	Cabo Verde	-2.9%	-2.9%	-3.1%	-3.1%	-2.7%	-2.7%	382.4%	392.5%	-0.3%	-1.0%	-3.3%	-3.3%	-2.9%	-3.0%	-1.5%	-1.7%	-3.0%	-3.0%	-1.3%	-1.5%	-0.6%	-0.5%	-1.9%	-1.9%	-0.3%	-0.3%	-0.9%	-2.8%	-2.9%	-3.1%	-3.0%	-3.0%
	Cambodia	-2.8%	-2.8%	-3.0%	-3.0%	-2.8%	-2.7%	-3.1%	-3.2%	-0.9%	-1.1%	-3.1%	-3.1%	-2.8%	-2.8%	55.5%	56.3%	-3.1%	-3.0%	-2.2%	-2.2%	-0.2%	-0.2%	-2.2%	-2.1%	-1.2%	-1.9%	-1.7%	-1.3%	-2.5%	-2.3%	-2.8%	-2.8%
	Cameroon	-1.9%	-1.9%	-2.1%	-2.0%	-1.6%	-1.6%	-0.5%	-0.8%	-1.0%	-1.1%	-2.8%	-2.8%	-2.0%	-1.9%	0.4%	0.4%	-1.8%	-1.7%	-1.7%	-1.6%	-0.2%	-0.0%	-2.0%	-2.1%	-0.5%	-0.5%	-1.6%	-1.9%	-1.3%	-2.2%	-2.2%	-1.9%
	Comoros	-2.4%	-2.5%	-2.5%	-2.5%	-2.3%	-2.6%	-2.9%	-3.1%	-1.1%	-1.4%	-2.4%	-2.5%	-2.1%	-2.2%	55.7%	61.7%	-2.6%	-2.8%	-1.5%	-1.7%	-0.5%	-0.3%	-2.0%	-2.0%	-2.4%	-2.4%	-2.1%	-2.3%	-2.0%	-2.1%	-2.6%	-2.6%
	Congo	-2.5%	-2.4%	-2.7%	-2.5%	-2.7%	-2.6%	-2.2%	-2.0%	-0.8%	-0.5%	-2.3%	-2.2%	-2.7%	-2.6%	-2.7%	-2.6%	-2.9%	-2.8%	-3.0%	-3.0%	-0.7%	-0.3%	-1.5%	-1.3%	-1.8%	-1.8%	-1.8%	-1.7%	-2.7%	-2.7%	-2.8%	-2.4%
	Democratic People's Republic of Kor..	-2.9%	-2.9%	-2.1%	-2.0%	-3.0%	-3.0%	1,194.0%	1,360.9%	-0.9%	-0.9%	-3.2%	-3.2%	-2.7%	-2.7%	27.8%	28.1%	-3.0%	-2.9%	-2.2%	-2.2%	-0.5%	-0.5%	-1.1%	-1.1%	-2.4%	-2.1%	-2.0%	-2.3%	-3.1%	-3.2%	-2.8%	-2.6%
	Djibouti	-2.2%	-2.4%	-2.8%	-2.9%	-2.0%	-2.1%	-1.6%	-1.8%	-1.1%	-1.1%	-2.3%	-2.3%	-2.0%	-1.9%	46.5%	46.2%	-2.4%	-2.3%	-2.9%	-2.9%	-0.3%	0.0%	-0.8%	-0.6%	-2.0%	-1.8%	-2.0%	-2.0%	-1.8%	-1.6%	-2.3%	-2.2%
	Dominican Republic	-2.4%	-2.3%	-3.0%	-3.0%	-2.8%	-2.7%	-3.1%	-3.2%	0.1%	-0.2%	-2.1%	-1.9%	-2.8%	-2.7%	-2.7%	-2.6%	-3.1%	-3.0%	-1.6%	-1.6%	-0.4%	-0.6%	0.0%	0.1%	0.1%	-0.6%	-1.9%	-2.0%	-3.0%	-2.8%	-2.9%	-2.7%
	El Salvador	-2.8%	-2.8%	-3.2%	-3.1%	-2.8%	-2.7%	-3.3%	-3.3%	-2.2%	-2.4%	-2.8%	-2.8%	-3.1%	-3.1%	15.9%	17.3%	-3.3%	-3.2%	-2.0%	-1.8%	-0.3%	-0.2%	-1.9%	-1.9%	-0.9%	-0.6%	-2.1%	-2.1%	-2.4%	-2.4%	-2.8%	-2.6%
	Eswatini	-1.3%	-1.5%	-1.2%	-1.1%	-1.2%	-1.5%	-2.8%	-2.8%	-0.2%	-0.8%	-2.9%	-3.0%	-1.7%	-1.9%	62.4%	63.6%	-1.7%	-1.8%	-1.9%	-1.9%	-0.0%	-0.1%	-2.2%	-2.3%	-0.8%	-1.0%	-0.8%	-1.2%	-1.8%	-2.8%	-2.4%	-2.3%
	Ghana	-2.3%	-2.2%	-2.9%	-3.0%	-2.2%	-1.9%	-1.3%	-0.7%	-0.2%	0.4%	-3.0%	-2.9%	-2.3%	-2.1%	-2.4%	-2.5%	-2.6%	-2.3%	-1.6%	-1.2%	-0.2%	0.0%	-0.6%	-0.1%	-0.0%	0.0%	-1.0%	-1.2%	-2.3%	-2.6%	-2.4%	-1.9%
	Guatemala	-2.6%	-2.5%	-2.9%	-2.9%	-2.3%	-2.2%	-3.2%	-3.2%	-1.7%	-1.7%	-3.0%	-3.0%	-2.4%	-2.4%	6.1%	5.4%	-3.2%	-3.2%	-2.9%	-2.9%	-0.3%	-0.5%	-3.1%	-3.2%	-2.5%	-2.2%	-0.6%	-0.2%	-2.0%	-1.7%	-2.5%	-2.3%
	Honduras	-2.7%	-2.5%	-2.9%	-2.8%	-2.8%	-2.7%	-3.2%	-3.2%	-1.4%	-1.2%	-2.7%	-2.5%	-2.5%	-2.2%	-2.4%	-2.4%	-3.1%	-3.0%	0.1%	-0.6%	-0.4%	-0.4%	-1.8%	-1.6%	-2.2%	-2.0%	-1.5%	-1.4%	-3.0%	-2.8%	-0.7%	-0.5%
	India	-2.6%	-2.7%	-2.8%	-2.8%	-2.4%	-2.6%	-2.9%	-3.0%	-1.3%	-1.5%	-3.0%	-3.1%	-2.8%	-2.6%	3.7%	4.7%	-3.0%	-2.9%	-2.4%	-2.5%	-1.0%	-0.7%	-1.8%	-1.9%	-2.0%	-1.6%	-1.6%	-1.7%	-2.4%	-2.2%	-2.7%	-2.7%
	Kenya	-2.3%	-2.3%	-2.0%	-1.8%	-2.3%	-2.5%	-2.2%	-2.3%	-0.8%	-0.9%	-2.8%	-2.9%	-2.5%	-2.6%	-2.5%	-2.5%	-2.6%	-2.7%	-2.1%	-2.1%	-0.2%	-0.1%	-1.9%	-2.1%	-1.6%	-1.8%	-0.8%	-0.9%	-2.2%	-2.2%	-2.4%	-2.4%
	Kiribati	-2.2%	-2.2%	-2.6%	-2.5%	-2.4%	-2.4%			-0.3%	-0.5%	-2.0%	-1.9%	-2.6%	-2.6%	0.7%	0.7%	-2.6%	-2.6%	-1.0%	-0.8%	-0.1%	-0.2%	-0.2%	-0.2%	-0.8%	-0.1%	-0.8%	-1.3%	-2.5%	-2.9%	-2.3%	-2.1%
	Kyrgyzstan	-2.8%	-2.8%	-2.9%	-2.9%	-2.9%	-2.9%	-3.3%	-3.3%	0.2%	0.1%	-1.8%	-1.8%	-2.8%	-2.8%	3.4%	7.2%	-2.6%	-2.8%	-0.3%	-0.6%	-0.2%	-0.2%	-3.3%	-3.2%	-2.4%	-1.6%	1.9%	1.3%	-3.1%	-3.0%	-2.4%	-2.3%
	Lao People's Democratic Republic	-2.8%	-2.8%	-2.9%	-3.0%	-2.8%	-2.8%	-3.0%	-3.0%	-0.9%	-1.0%	-3.0%	-3.0%	-2.8%	-2.7%	3,959.6%	3,998.5%	-3.0%	-3.0%	-2.1%	-2.1%	-0.2%	-0.2%	-1.2%	-1.0%	-1.5%	-2.5%	-1.7%	-1.7%	-3.2%	-3.2%	-2.7%	-2.6%
	Lesotho	-1.2%	-1.1%	-2.1%	-2.0%	-1.0%	-0.7%			-0.5%	-0.5%	-2.9%	-2.9%	-1.7%	-1.4%	9.9%	10.3%	-1.1%	-0.5%	-1.8%	-1.8%	0.0%	0.1%	-1.5%	-1.5%	-0.8%	-0.7%	-1.1%	-1.1%	-1.2%	-2.5%	-1.5%	-0.9%
	Maldives	-2.9%	-2.8%	-2.9%	-3.0%	-2.9%	-2.8%			-1.3%	-1.6%	-3.2%	-3.2%	-2.7%	-2.7%	6.8%	7.4%	-3.2%	-3.2%	-2.4%	-2.1%	-0.1%	-0.1%	-1.7%	-1.7%	-0.2%	-0.6%	-1.8%	-2.2%	-3.2%	-3.0%	-3.2%	-3.0%
	Marshall Islands	-1.5%	-1.6%	-2.5%	-2.6%	-1.5%	-1.6%			-0.5%	-0.6%	-1.5%	-1.6%	-1.9%	-2.1%	1.7%	1.7%	-2.2%	-2.2%	-0.6%	-0.8%	-0.0%	-0.1%	-0.1%	0.0%	-0.0%	-0.1%	-0.5%	-0.4%	-2.5%	-2.5%	-0.5%	-1.1%
	Mauritania	-2.3%	-2.3%	-2.5%	-2.3%	-2.4%	-2.5%	9.1%	7.9%	-1.4%	-1.5%	-3.1%	-3.1%	-2.6%	-2.5%	-3.1%	-3.0%	-2.8%	-2.8%	-1.8%	-1.8%	-0.7%	-0.5%	-1.6%	-1.6%	-0.4%	-0.4%	-1.7%	-1.9%	-2.3%	-2.7%	-2.9%	-2.8%
	Micronesia (Federated States of)	-2.3%	-2.2%	-2.8%	-2.7%	-2.6%	-2.5%			-1.0%	-1.1%	-2.5%	-2.4%	-2.7%	-2.7%	-1.6%	-1.6%	-2.9%	-2.9%	-1.6%	-1.6%	-0.1%	-0.1%	-1.0%	-1.0%	-0.0%	-0.1%	-1.3%	-1.2%	-2.8%	-2.7%	-2.4%	-2.0%
	Mongolia	-3.0%	-3.0%	-3.1%	-3.1%	-3.0%	-3.1%			-0.7%	-1.0%	-2.8%	-2.8%	-3.1%	-3.0%	294.7%	295.4%	-3.1%	-3.2%	-2.3%	-2.1%	-0.5%	-1.0%	1.5%	1.4%	-2.1%	-2.1%	-1.8%	-3.0%	-3.2%	-3.2%	-2.0%	-2.9%
	Morocco	-2.9%	-2.9%	-3.0%	-3.0%	-3.0%	-2.9%	-3.3%	-3.3%	-1.0%	-1.1%	-3.0%	-3.1%	-2.8%	-2.9%	3.8%	3.8%	-3.1%	-3.1%	-1.8%	-1.7%	-0.0%	-0.0%	-1.4%	-1.4%	0.1%	-0.0%	-1.5%	-1.7%	-3.0%	-3.1%	-2.9%	-2.9%
	Myanmar	-2.8%	-2.8%	-3.0%	-3.2%	-2.8%	-2.7%	-3.2%	-3.2%	-1.4%	-1.2%	-2.9%	-2.9%	-2.9%	-2.4%	17.3%	17.9%	-2.9%	-2.9%	-2.3%	-1.8%	-0.1%	-0.1%	0.2%	0.7%	-1.1%	-1.5%	-1.1%	-2.1%	-2.9%	-2.9%	-2.7%	-2.4%
	Nicaragua	-2.8%	-2.8%	-3.1%	-3.2%	-2.7%	-2.6%	-3.2%	-3.2%	-1.9%	-1.8%	-3.3%	-3.2%	-2.9%	-2.9%	15.3%	15.5%	-3.2%	-3.2%	-1.3%	-1.9%	-0.2%	-0.4%	-2.3%	-2.3%	-0.1%	-0.4%	-2.3%	-2.2%	-2.9%	-2.8%	-2.8%	-2.7%
	Nigeria	-2.0%	-2.1%	-2.1%	-2.4%	-1.8%	-1.9%	-1.9%	-1.8%	-0.8%	-0.8%	-2.8%	-2.8%	-1.7%	-1.6%	4.0%	3.9%	-2.4%	-2.3%	-1.8%	-1.8%	-0.2%	-0.1%	-0.0%	-0.1%	-1.0%	-0.5%	-0.7%	-0.7%	-2.2%	-2.7%	-2.4%	-2.2%
	Palestine	-2.7%	-2.6%	-2.0%	-1.9%	-2.9%	-2.8%			-1.4%	-1.0%	-3.2%	-3.2%	-3.0%	-2.9%	18.0%	18.0%	-3.1%	-3.0%	-2.3%	-2.6%	-0.0%	0.0%	-0.9%	-0.8%	-0.1%	-0.1%	-1.7%	-1.6%	-3.1%	-2.8%	-2.9%	-2.8%
	Sao Tome and Principe	-2.8%	-2.8%	-3.1%	-3.1%	-2.8%	-2.9%	-0.1%	-2.1%	-1.5%	-1.1%	-3.1%	-3.2%	-2.6%	-2.8%	-0.2%	0.8%	-3.0%	-3.1%	-1.9%	-2.1%	-0.9%	-0.7%	-1.4%	-1.4%	-0.6%	-0.6%	-1.4%	-1.9%	-2.8%	-3.1%	-3.0%	-2.9%
	Sudan	-2.9%	-2.9%	-2.7%	-2.8%	-2.9%	-2.9%	-2.1%	-2.6%	-1.2%	-1.3%	-3.2%	-3.2%	-3.0%	-2.9%	2.0%	2.0%	-3.1%	-3.1%	-2.7%	-2.6%	0.0%	0.0%	-0.8%	-0.5%	-0.4%	-0.4%	-1.4%	-1.5%	-2.9%	-3.1%	-3.0%	-2.7%
	Tajikistan	-2.7%	-2.6%	-2.8%	-2.9%	-2.7%	-2.5%	-3.3%	-3.3%	0.4%	-0.2%	-1.9%	-1.8%	-2.8%	-2.9%	2.2%	2.4%	-2.4%	-2.1%	-1.5%	-1.3%	0.0%	0.0%	-1.4%	-1.4%	-2.1%	-1.8%	-0.8%	-0.6%	-3.0%	-3.0%	-2.6%	-2.5%
	Timor-Leste	-2.8%	-2.8%	-2.8%	-2.8%	-2.8%	-2.9%	-3.3%	-3.3%	-1.2%	-1.1%	-3.0%	-3.0%	-2.6%	-2.6%	6.1%	6.2%	-3.0%	-3.0%	-2.1%	-1.9%	-0.1%	-0.2%	-1.3%	-1.1%	-1.4%	-1.7%	-1.1%	-1.3%	-3.1%	-3.1%	-2.6%	-2.1%
	Tuvalu	-2.8%	-2.7%	-2.5%	-2.5%	-3.0%	-3.0%			-1.3%	-1.3%	-2.7%	-2.6%	-3.0%	-3.0%	19.4%	20.6%	-3.2%	-3.2%	-1.7%	-1.3%	-0.2%	-0.5%	-1.2%	-1.2%	-0.2%	-0.3%	-1.8%	-2.0%	-3.1%	-3.1%	-3.0%	-2.9%
	Vanuatu	-1.5%	-1.4%	-2.0%	-2.0%	-1.3%	-1.3%	-3.3%	-3.3%	-0.7%	-0.7%	-2.0%	-2.0%	-1.4%	-1.5%	1.2%	1.4%	-2.0%	-1.8%	0.6%	0.8%	0.1%	0.1%	0.6%	0.7%	-0.1%	-0.1%	-0.2%	0.0%	-2.1%	-2.8%	-0.8%	-0.3%
	Venezuela (Bolivarian Republic of)	-2.1%	-2.1%	-2.8%	-2.8%	-2.0%	-1.8%	4.0%	2.0%	-0.7%	-0.9%	-2.0%	-1.8%	-2.6%	-2.7%	-2.7%	-2.8%	-2.8%	-2.9%	-1.5%	-1.2%	-0.1%	-0.2%	-0.8%	-1.2%	-0.5%	-0.6%	-0.4%	-0.3%	-3.0%	-3.0%	-2.7%	-2.7%
	Zambia	-2.6%	-2.6%	-2.6%	-2.5%	-2.8%	-2.8%	-2.5%	-2.6%	-1.0%	-1.0%	-3.1%	-3.1%	-3.0%	-3.0%	-1.7%	-1.7%	-3.0%	-3.0%	-3.1%	-3.1%	-0.5%	-0.3%	-1.2%	-1.2%	-2.6%	-2.5%	-1.7%	-2.0%	-2.7%	-2.9%	-3.0%	-2.8%
	Zimbabwe	-1.3%	-1.4%	-1.1%	-1.4%	-0.2%	-0.4%	-1.4%	-1.7%	-0.2%	-0.1%	-2.4%	-2.5%	-1.2%	-1.3%	-2.2%	-2.2%	0.3%	-0.1%	-1.9%	-1.9%	0.1%	0.0%	-0.5%	-0.4%	-0.3%	-0.4%	0.4%	0.2%	-0.1%	-2.3%	-0.0%	0.0%

<5 year

Middle SDI

Country	Total commuincable Female	Total commuincable Male	Enteric infections Female	Enteric infections Male	Lower respiratory infections Female	Lower respiratory infections Male	Malaria Female	Malaria Male	Neonatal sepsis and other neonatal infections Female	Neonatal sepsis and other neonatal infections Male
Albania	-2.9%	-2.9%	-2.8%	-2.6%	-3.0%	-3.0%			-1.8%	-1.2%
Algeria	-2.8%	-2.8%	-2.7%	-3.0%	-2.9%	-2.9%	-3.0%	-3.0%	-0.9%	-0.9%
Armenia	-2.7%	-2.6%	-3.1%	-3.1%	-2.8%	-2.6%	-3.3%	-3.3%	-1.5%	-1.9%
Azerbaijan	-2.6%	-2.7%	-3.0%	-3.1%	-2.6%	-2.7%	-3.3%	-3.3%	0.4%	-0.1%
Botswana	-1.8%	-1.8%	-2.1%	-2.1%	0.1%	-0.2%	1.8%	2.7%	-0.7%	-0.8%
Brazil	-2.8%	-2.7%	-3.1%	-3.1%	-2.8%	-2.8%	-3.3%	-3.3%	-1.6%	-1.5%
China	-3.1%	-3.1%	-3.2%	-3.1%	-3.2%	-3.1%	-3.3%	-3.3%	0.0%	-0.8%
Colombia	-2.3%	-2.3%	-2.9%	-3.0%	-2.4%	-2.5%	-3.2%	-3.3%	0.6%	0.9%
Costa Rica	-2.2%	-2.4%	-2.4%	-2.6%	-2.5%	-2.5%	-3.3%	-3.3%	-2.1%	-2.3%
Cuba	-2.2%	-2.3%	-2.3%	-2.5%	-2.2%	-2.2%			-0.9%	-1.5%
Ecuador	-2.6%	-2.6%	-3.1%	-3.1%	-2.6%	-2.5%	-3.3%	-3.3%	-1.7%	-2.0%
Egypt	-2.9%	-2.8%	-2.8%	-2.7%	-3.0%	-2.9%	-3.3%	-3.3%	-1.3%	-1.4%
Equatorial Guinea	-2.8%	-2.8%	-3.2%	-3.2%	-3.1%	-3.1%	-2.2%	-2.0%	-1.7%	-1.4%
Fiji	-1.0%	-1.0%	-0.4%	-0.5%	-0.6%	-0.7%			-1.1%	-1.2%
Gabon	-2.4%	-2.4%	-2.8%	-2.6%	-2.8%	-2.7%	-2.3%	-2.3%	-0.8%	-0.9%
Grenada	-1.3%	-1.6%	-1.7%	-2.5%	-1.5%	-1.8%			0.4%	0.1%
Guyana	-2.1%	-2.1%	-2.7%	-2.8%	-1.8%	-1.9%	-3.1%	-3.1%	-0.5%	-0.4%
Indonesia	-2.7%	-2.6%	-2.7%	-2.8%	-2.8%	-2.8%	-3.0%	-3.1%	-1.3%	-1.5%
Iran (Islamic Republic of)	-3.0%	-2.9%	-2.8%	-2.8%	-3.1%	-3.1%	-3.3%	-3.3%	-1.4%	-1.3%
Iraq	-2.6%	-2.7%	-2.8%	-2.8%	-2.8%	-3.0%	-3.3%	-3.3%	-1.5%	-0.3%
Jamaica	-1.7%	-2.0%	-2.9%	-3.0%	-2.5%	-2.5%			2.0%	0.8%
Mexico	-2.7%	-2.7%	-3.1%	-3.1%	-2.8%	-2.8%	-3.3%	-3.3%	-0.6%	-0.6%
Namibia	-1.8%	-1.9%	-1.9%	-1.7%	-1.8%	-2.0%	-2.2%	-2.2%	-0.7%	-0.8%
Nauru	-1.5%	-1.4%	-1.1%	-1.3%	-1.4%	-1.4%			-0.5%	-0.4%
Panama	-1.4%	-1.4%	-1.8%	-2.0%	-0.8%	-0.4%	-1.6%	-1.5%	-0.8%	-0.9%
Paraguay	-2.2%	-2.2%	-2.8%	-2.8%	-2.4%	-2.5%	-3.3%	-3.3%	-2.6%	-2.6%
Peru	-2.7%	-2.7%	-3.0%	-3.0%	-3.1%	-3.0%	-3.3%	-3.3%	-1.2%	-1.2%
Philippines	-2.3%	-2.3%	-2.8%	-2.8%	-2.4%	-2.3%	-3.2%	-3.3%	-0.8%	-1.1%
Saint Lucia	-1.4%	-1.8%	-2.2%	-2.5%	-1.6%	-1.8%			0.0%	-0.2%
Saint Vincent and the Grenadines	-1.4%	-1.8%	-2.5%	-2.6%	-1.8%	-1.9%			1.5%	0.5%
Samoa	-1.8%	-1.8%	-1.6%	-1.8%	-2.6%	-2.5%			-0.8%	-1.0%
South Africa	-2.1%	-2.2%	-2.5%	-2.5%	-2.4%	-2.4%	-1.3%	-1.8%	0.5%	-0.1%
Suriname	-2.0%	-2.1%	-2.6%	-2.8%	-1.8%	-2.0%	-3.3%	-3.3%	-0.4%	-0.4%
Syrian Arab Republic	-2.7%	-2.7%	-2.9%	-2.8%	-2.6%	-2.6%	-3.3%	-3.3%	-1.6%	-1.8%
Thailand	-2.4%	-2.5%	-2.5%	-2.8%	-2.8%	-2.8%	-3.3%	-3.3%	0.8%	0.3%
Tokelau	-2.2%	-2.2%	-2.1%	-2.3%	-2.6%	-2.7%			-1.2%	-1.5%
Tonga	-1.5%	-1.5%	-2.0%	-1.9%	-2.0%	-1.9%			-0.8%	-0.8%
Tunisia	-2.9%	-2.7%	-2.4%	-2.1%	-3.1%	-3.0%			-1.5%	-1.5%
Turkmenistan	-2.7%	-2.7%	-3.1%	-3.2%	-2.6%	-2.6%	-3.3%	-3.3%	1.7%	1.3%
Uzbekistan	-2.2%	-2.3%	-3.2%	-3.2%	-2.1%	-2.1%	-3.3%	-3.3%	2.6%	2.9%
Viet Nam	-2.6%	-2.6%	-2.8%	-3.0%	-2.7%	-2.7%	-3.2%	-3.2%	-1.5%	-1.6%

Country	Vaccine Preventable disease Female	Vaccine Preventable disease Male	Meningitis & Encephalitis Female	Meningitis & Encephalitis Male	HIV/AIDS Female	HIV/AIDS Male	Tuberculosis Female	Tuberculosis Male	Neglected Tropical diseases Female	Neglected Tropical diseases Male
Albania	-3.1%	-3.2%	-2.8%	-2.8%	0.5%	0.8%	-3.0%	-3.2%	-2.0%	-2.3%
Algeria	-3.0%	-3.0%	-2.8%	-2.8%	0.2%	0.3%	-3.1%	-3.0%	-1.8%	-1.9%
Armenia	-1.1%	-0.9%	-1.8%	-1.0%	15.9%	8.9%	-2.6%	-2.5%	0.3%	0.1%
Azerbaijan	-2.4%	-2.4%	-1.5%	-1.9%	0.2%	0.3%	-2.9%	-2.9%	-1.5%	-1.8%
Botswana	-2.6%	-2.8%	-0.5%	-0.5%	-3.0%	-3.0%	-0.9%	-0.8%	-2.0%	-1.9%
Brazil	-2.5%	-2.6%	-2.9%	-2.9%	-2.2%	-2.2%	-3.1%	-3.1%	-0.9%	-0.8%
China	-3.2%	-3.2%	-2.9%	-2.9%	2.5%	2.7%	-3.2%	-3.2%	-2.8%	-2.8%
Colombia	-2.5%	-2.7%	-2.6%	-2.7%	9.0%	3.3%	-2.8%	-2.9%	1.5%	0.3%
Costa Rica	-2.1%	-1.9%	-2.3%	-2.6%	-1.6%	-1.3%	-2.9%	-3.0%	-1.8%	-1.7%
Cuba	-1.5%	-1.6%	-3.0%	-3.0%	3.3%	9.4%	-2.3%	-2.3%	-1.5%	-1.3%
Ecuador	-2.3%	-2.3%	-2.6%	-2.5%	8.3%	9.9%	-3.2%	-3.1%	-2.5%	-2.4%
Egypt	-3.2%	-3.2%	-2.5%	-2.4%	-2.7%	-2.7%	-3.1%	-3.1%	-2.8%	-2.8%
Equatorial Guinea	-3.1%	-3.0%	-3.0%	-2.8%	27.2%	27.0%	-3.3%	-3.2%	-2.9%	-3.0%
Fiji	-2.1%	-2.3%	-1.4%	-1.5%	3.3%	3.5%	-1.5%	-0.9%	0.0%	-0.6%
Gabon	-2.8%	-2.7%	-2.8%	-2.7%	0.5%	0.4%	-3.0%	-2.9%	-2.7%	-2.7%
Grenada	-2.1%	-2.3%	-2.7%	-2.9%	-2.3%	-2.2%	-2.5%	-2.8%	-1.8%	-1.7%
Guyana	-2.5%	-2.8%	-2.3%	-2.4%	-0.7%	-1.2%	-2.7%	-2.8%	-0.6%	-0.8%
Indonesia	-2.9%	-2.9%	-2.7%	-2.6%	289.1%	322.8%	-3.0%	-3.0%	-2.5%	-1.6%
Iran (Islamic Republic of)	-3.2%	-3.2%	-3.0%	-2.9%	93.6%	96.7%	-3.2%	-3.2%	-2.4%	-2.3%
Iraq	-2.8%	-2.8%	-2.5%	-2.8%	17.2%	17.3%	-3.0%	-3.1%	-2.6%	-2.7%
Jamaica	-2.3%	-2.5%	-2.6%	-2.8%	0.8%	1.0%	-2.6%	-3.0%	-0.7%	-0.5%
Mexico	-2.9%	-2.9%	-2.6%	-2.6%	0.9%	-0.3%	-3.1%	-3.1%	-2.3%	-2.1%
Namibia	-3.1%	-3.1%	-1.9%	-2.1%	4.8%	4.9%	-2.2%	-2.4%	-1.4%	-1.6%
Nauru	-3.0%	-3.0%	-1.6%	-1.6%	24.8%	24.8%	-2.1%	-2.3%	-0.1%	0.0%
Panama	-2.8%	-2.9%	-2.5%	-2.7%	0.7%	0.9%	-2.4%	-2.5%	-2.4%	-2.4%
Paraguay	-2.8%	-2.8%	-2.6%	-2.7%	23.7%	24.0%	-2.8%	-2.7%	-1.3%	-1.2%
Peru	-2.4%	-2.4%	-2.7%	-2.6%	16.2%	16.1%	-3.2%	-3.2%	-2.5%	-2.2%
Philippines	-2.5%	-2.5%	-2.3%	-2.2%	17.8%	15.6%	-2.6%	-2.5%	-0.2%	-0.3%
Saint Lucia	-1.8%	-1.7%	-2.4%	-2.7%	-2.2%	-2.7%	-2.7%	-2.9%	-0.6%	-1.1%
Saint Vincent and the Grenadines	-1.0%	-1.8%	-2.5%	-2.7%	-2.1%	-2.4%	-2.8%	-2.8%	-1.3%	-1.2%
Samoa	-1.7%	-1.4%	-2.5%	-2.6%	2.0%	2.2%	-2.8%	-3.0%	-0.5%	-0.3%
South Africa	-2.5%	-2.6%	-2.4%	-2.5%	2.8%	3.2%	-2.6%	-2.7%	-2.6%	-2.7%
Suriname	-2.8%	-2.8%	-2.4%	-2.5%	-1.8%	-2.2%	-2.8%	-2.8%	1.2%	0.0%
Syrian Arab Republic	-2.9%	-3.0%	-2.6%	-2.7%	-0.2%	-0.0%	-3.0%	-3.1%	-0.5%	-0.4%
Thailand	-2.9%	-2.8%	-2.8%	-2.8%	-0.7%	-0.6%	-3.1%	-3.0%	-2.5%	-2.4%
Tokelau	-2.4%	-2.5%	-2.5%	-2.6%	25.2%	21.7%	-3.0%	-3.0%	-1.1%	-0.7%
Tonga	-1.3%	-1.4%	-1.7%	-1.7%	5.8%	5.8%	-2.4%	-2.3%	-1.4%	-1.3%
Tunisia	-3.0%	-2.9%	-3.0%	-2.7%	13.2%	12.8%	-3.2%	-3.1%	-2.3%	-2.3%
Turkmenistan	-2.6%	-2.7%	-2.2%	-2.3%	-2.1%	-1.7%	-2.8%	-2.9%	-1.4%	-1.8%
Uzbekistan	-2.5%	-2.6%	-2.5%	-2.6%	-2.0%	-0.5%	-2.4%	-2.5%	-0.5%	-0.8%
Viet Nam	-3.0%	-3.0%	-2.3%	-2.1%	-3.1%	-3.1%	-2.9%	-2.9%	-2.3%	-2.1%

Country	Infectious skin conditions Female	Infectious skin conditions Male	Sexually transmitted infections excluding HIV Female	Sexually transmitted infections excluding HIV Male	Upper respiratory infections Female	Upper respiratory infections Male	Other unspecified infectious diseases Female	Other unspecified infectious diseases Male	Hepatitis Female	Hepatitis Male	Rheumatic heart disease Female	Rheumatic heart disease Male
Albania	-1.1%	-1.0%	-1.6%	-1.3%	-0.9%	-1.1%	-2.0%	-2.0%	-3.1%	-3.0%	-1.4%	-2.7%
Algeria	-0.1%	-0.1%	-1.9%	-1.9%	-0.1%	-0.1%	-1.2%	-1.5%	-2.9%	-3.0%	-2.8%	-2.5%
Armenia	0.0%	0.0%	-2.8%	-3.0%	-2.5%	-0.8%	1.0%	1.5%	-3.1%	-2.4%	-0.3%	-1.1%
Azerbaijan	-0.1%	-0.0%	-0.8%	-1.0%	-1.8%	-1.8%	1.6%	1.1%	-3.0%	-3.0%	-1.9%	-2.0%
Botswana	-0.0%	-0.0%	-1.8%	-1.9%	-0.3%	-0.3%	-1.0%	-0.8%	-1.3%	-2.1%	-0.9%	-0.7%
Brazil	0.1%	-0.2%	-1.7%	-1.8%	-1.0%	-1.2%	-0.7%	-0.7%	-2.7%	-2.6%	-2.7%	-2.7%
China	-0.7%	-0.7%	-1.3%	-1.4%	-2.6%	-2.4%	-2.6%	-2.8%	-3.2%	-3.2%	-3.2%	-3.1%
Colombia	-0.1%	-0.3%	-2.6%	-2.5%	-1.0%	-1.2%	-0.9%	-0.7%	-2.5%	-2.3%	-2.8%	-2.9%
Costa Rica	-0.0%	-0.1%	-2.7%	-1.5%	-0.1%	-0.1%	-0.2%	0.4%	-2.9%	-3.0%	-1.8%	-1.7%
Cuba	-0.1%	-0.3%	-3.0%	-3.1%	-0.4%	-0.5%	-0.5%	-0.3%	-3.0%	-3.0%	-1.9%	-1.7%
Ecuador	0.0%	0.0%	-1.5%	-1.5%	-1.5%	-1.5%	-1.5%	-1.4%	-3.1%	-3.0%	-2.3%	-2.4%
Egypt	-0.2%	-0.2%	-1.7%	-1.6%	-1.0%	-0.9%	-2.0%	-2.1%	-3.2%	-3.1%	-3.2%	-3.0%
Equatorial Guinea	-1.1%	-0.5%	-1.0%	-0.2%	-2.8%	-2.7%	-2.4%	-2.3%	-3.2%	-3.2%	-3.1%	-3.0%
Fiji	0.0%	0.1%	-0.1%	-0.1%	0.0%	-0.1%	0.2%	0.9%	-1.5%	-1.8%	0.1%	0.3%
Gabon	-0.6%	-0.3%	-1.9%	-1.8%	-1.5%	-1.8%	-1.9%	-2.0%	-2.9%	-2.8%	-2.7%	-2.3%
Grenada	0.1%	-0.1%	-3.0%	-3.0%	-0.1%	-1.1%	-0.9%	-0.6%	-2.9%	-3.0%	-2.4%	-2.5%
Guyana	0.3%	0.1%	-2.9%	-2.9%	-0.1%	-0.4%	-0.9%	-0.5%	-2.9%	-2.9%	-2.6%	-2.4%
Indonesia	-0.1%	-0.1%	0.2%	0.7%	-0.3%	-0.5%	-1.4%	-1.7%	-3.2%	-2.8%	-2.9%	-2.8%
Iran (Islamic Republic of)	-0.1%	-0.1%	-1.2%	-1.2%	-0.2%	-0.1%	-1.2%	-1.5%	-3.2%	-3.1%	-3.1%	-3.0%
Iraq	-0.0%	-0.2%	-0.9%	-0.9%	-0.7%	-0.6%	-1.3%	-2.1%	-3.0%	-3.0%	-3.0%	-2.8%
Jamaica	-0.0%	-0.2%	-3.2%	-1.3%	-0.1%	-0.1%	0.2%	0.4%	-1.3%	-2.5%	-3.0%	-2.9%
Mexico	-0.1%	-0.1%	-3.1%	-3.1%	-2.4%	-2.4%	-1.5%	-1.2%	-2.3%	-2.1%	-2.9%	-2.8%
Namibia	-0.1%	-0.1%	-1.3%	-1.2%	-0.8%	-0.9%	-1.3%	-1.5%	-2.1%	-2.5%	-2.4%	-2.3%
Nauru	0.0%	0.0%	0.6%	0.6%	0.0%	-0.1%	-0.7%	-0.5%	-2.2%	-2.2%	-1.1%	-1.3%
Panama	-0.1%	-0.1%	2.6%	3.4%	0.7%	0.7%	2.1%	1.6%	-3.0%	-3.0%	-2.6%	-2.5%
Paraguay	0.1%	0.0%	0.2%	0.2%	-0.1%	-0.2%	0.6%	0.2%	-2.6%	-2.5%	-2.1%	-1.6%
Peru	-0.8%	-0.8%	-2.2%	-2.2%	-1.3%	-1.6%	-1.5%	-1.3%	-3.1%	-3.1%	-2.6%	-2.5%
Philippines	-0.3%	-0.2%	0.3%	0.2%	-0.9%	-0.8%	-0.4%	0.1%	-3.2%	-3.2%	-0.9%	-0.7%
Saint Lucia	0.0%	-0.1%	-2.6%	-2.6%	-0.1%	-1.2%	-0.8%	-0.6%	-2.6%	-2.8%	-2.3%	-2.3%
Saint Vincent and the Grenadines	0.5%	0.4%	-2.9%	-3.0%	0.2%	-1.0%	-1.2%	-0.9%	-2.9%	-2.7%	-2.5%	-2.6%
Samoa	-0.1%	-0.2%	0.0%	-0.0%	-0.0%	-0.1%	-1.1%	-1.2%	-2.3%	-2.7%	-2.5%	-2.2%
South Africa	-0.4%	-0.6%	-1.4%	-1.4%	-1.1%	-1.2%	-0.7%	-1.1%	-2.5%	-3.1%	-2.8%	-2.7%
Suriname	-0.2%	-0.5%	-1.3%	-1.3%	-0.2%	-0.4%	-0.7%	-0.7%	-2.9%	-3.0%	-2.3%	-2.4%
Syrian Arab Republic	0.0%	0.0%	-1.5%	-1.5%	-0.2%	-0.5%	-1.4%	-1.6%	-3.1%	-3.1%	-2.8%	-2.8%
Thailand	-0.1%	-0.1%	-2.5%	-2.5%	-0.3%	-0.4%	-1.1%	-2.2%	-2.6%	-2.4%	-1.1%	-2.8%
Tokelau	-0.1%	-0.2%	-0.7%	-0.9%	-0.1%	-0.2%	-1.2%	-1.3%	-2.7%	-2.6%	-2.5%	-2.5%
Tonga	-0.0%	-0.1%	-0.7%	-0.7%	-0.0%	-0.1%	-0.9%	-1.1%	-2.4%	-2.5%	-1.7%	-1.6%
Tunisia	-0.1%	-0.1%	-1.8%	-1.8%	-0.1%	-0.1%	-1.7%	-1.8%	-3.1%	-3.1%	-3.2%	-2.8%
Turkmenistan	0.0%	-0.1%	-3.1%	-2.5%	-2.3%	-2.0%	0.9%	1.3%	-3.2%	-3.2%	-2.8%	-2.8%
Uzbekistan	-0.1%	-0.1%	-2.4%	-3.0%	-2.3%	-2.3%	3.6%	3.9%	-3.2%	-3.2%	-2.4%	-2.4%
Viet Nam	-0.1%	-0.0%	-1.0%	-1.2%	-0.4%	-0.5%	-2.2%	-2.5%	-3.0%	-2.9%	-2.8%	-2.5%

<5 year

SDI	Country	Total communicable Female	Total communicable Male	Enteric infections Female	Enteric infections Male	Lower respiratory infections Female	Lower respiratory infections Male	Malaria Female	Malaria Male	Neonatal sepsis and other neonatal infections Female	Neonatal sepsis and other neonatal infections Male	Vaccine Preventable disease Female	Vaccine Preventable disease Male	Meningitis & Encephalitis Female	Meningitis & Encephalitis Male	HIV/AIDS Female	HIV/AIDS Male
High-middle SDI	American Samoa	-1.3%	-1.3%	-0.7%	-0.9%	-1.7%	-1.7%			-0.2%	-1.7%	-1.5%	-1.5%	-1.2%	-1.7%	8.1%	8.3%
	Antigua and Barbuda	-1.1%	-0.6%	-1.7%	-1.1%	-0.7%	0.1%			-0.8%	-0.6%	0.0%	-0.4%	-2.0%	-1.9%	-1.1%	-1.0%
	Argentina	-2.2%	-2.2%	-2.6%	-2.5%	-2.3%	-2.2%	-3.3%	-3.3%	-1.9%	-2.0%	-1.8%	-1.7%	-2.6%	-2.6%	2.7%	2.5%
	Bahamas	-1.6%	-1.6%	-2.1%	-2.1%	-2.1%	-1.8%			-0.8%	-1.1%	-0.6%	-0.8%	-2.5%	-2.6%	0.5%	-0.9%
	Bahrain	-2.3%	-2.3%	-1.3%	-1.2%	-2.6%	-2.7%			-0.1%	-1.9%	-3.0%	-3.1%	-2.7%	-2.7%	-2.6%	-2.6%
	Barbados	-0.7%	-1.1%	-1.4%	-1.7%	-1.2%	-1.5%			0.6%	0.0%	-1.7%	-1.5%	-2.3%	-2.6%	-1.8%	-2.1%
	Belarus	-2.3%	-2.4%	-2.1%	-2.2%	-3.0%	-3.0%			-0.9%	-1.2%	-2.5%	-2.1%	-2.0%	-2.1%	-0.3%	1.4%
	Bosnia and Herzegovina	-1.7%	-1.7%	-1.8%	-1.6%	-2.6%	-2.4%			0.0%	-0.3%	-1.6%	-1.6%	-2.1%	-2.3%	-1.5%	-1.6%
	Bulgaria	-2.3%	-2.3%	-1.3%	-1.6%	-2.6%	-2.6%			14.6%	14.4%	6.2%	9.4%	-2.2%	-2.2%	0.3%	-0.1%
	Chile	-2.6%	-2.7%	-2.2%	-2.4%	-3.0%	-3.1%			-1.7%	-1.9%	1.7%	1.6%	-2.8%	-2.8%	2.9%	1.1%
	Cook Islands	-2.6%	-2.6%	-0.9%	-1.0%	-3.1%	-3.1%			-2.0%	-2.2%	-2.6%	-2.4%	-2.7%	-2.8%	25.7%	29.5%
	Croatia	-1.4%	-1.5%	-0.4%	-1.1%	-2.8%	-2.9%			7.6%	7.3%	-1.0%	-0.5%	-2.7%	-2.7%	6.3%	-1.1%
	Dominica	0.3%	0.2%	-1.4%	-1.6%	1.0%	1.0%			1.0%	0.8%	5.6%	6.6%	-1.2%	-1.4%	1.2%	1.4%
	Georgia	-2.8%	-2.8%	-2.8%	-2.9%	-3.1%	-3.1%	-3.3%	-3.3%	3.0%	2.1%	0.9%	0.7%	-2.0%	-2.3%	11.0%	71.3%
	Greece	-1.1%	-1.5%	0.3%	0.2%	-1.0%	-1.4%			-3.0%	-3.0%	-2.9%	-2.7%	-2.0%	-2.0%	-0.2%	-2.1%
	Greenland	-2.3%	-2.4%	0.2%	0.3%	-2.8%	-2.9%			-0.2%	-1.4%	-2.3%	-2.4%	-2.9%	-2.7%	6.3%	6.2%
	Hungary	-2.2%	-2.4%	0.2%	0.3%	-2.8%	-2.9%			-1.8%	-0.9%	-2.0%	-1.8%	-2.8%	-2.9%	-2.7%	-2.8%
	Israel	-1.8%	-1.7%	0.1%	0.3%	-2.7%	-2.7%			-2.1%	-1.8%	-0.3%	-0.1%	-2.6%	-2.7%	-2.5%	-2.3%
	Italy	-1.6%	-1.8%	0.6%	0.4%	-2.9%	-2.9%			-1.3%	-1.1%	-2.9%	-2.7%	-2.5%	-2.6%	-2.4%	-2.7%
	Jordan	-2.2%	-2.1%	-2.2%	-2.2%	-2.2%	-2.3%			-1.5%	-1.1%	-2.7%	-2.8%	-2.1%	-2.1%	5.0%	5.0%
	Kazakhstan	-2.8%	-2.8%	-3.2%	-3.2%	-2.9%	-2.9%			0.4%	-0.2%	-2.0%	-1.9%	-2.2%	-2.3%	1.9%	0.0%
	Lebanon	-2.4%	-2.4%	-1.9%	-1.8%	-2.8%	-2.7%			-1.3%	-1.3%	-2.8%	-2.9%	-2.6%	-2.5%	0.2%	0.3%
	Libya	-2.6%	-2.7%	-2.6%	-2.6%	-2.7%	-2.9%			-1.6%	-1.8%	-2.9%	-3.0%	-2.7%	-2.7%	6.7%	6.1%
	Malaysia	-2.1%	-2.1%	-1.8%	-2.0%	-2.7%	-2.6%	-3.3%	-3.3%	-1.5%	-1.6%	-2.9%	-2.9%	-2.4%	-2.4%	78.8%	81.6%
	Malta	-1.2%	-1.5%	0.5%	0.3%	-1.9%	-2.0%			-0.5%	-1.0%	-2.3%	-2.2%	-2.1%	-2.3%	-1.8%	-2.4%
	Mauritius	-1.6%	-1.3%	-2.3%	-2.4%	-2.2%	-1.9%			-0.6%	0.6%	-1.4%	-1.6%	-2.0%	-2.0%	10.8%	12.0%
	Montenegro	-2.2%	-1.9%	-0.8%	-1.0%	-2.9%	-2.7%			-1.1%	-1.1%	-1.9%	-1.6%	-2.6%	-2.5%	6.5%	6.4%
	Niue	-0.8%	-0.8%	-1.1%	-1.1%	-1.0%	-1.0%			-0.4%	-0.7%	-1.3%	-1.1%	-2.0%	-1.9%	24.2%	26.2%
	North Macedonia	-2.9%	-2.8%	-3.2%	-3.2%	-3.1%	-3.0%			12.1%	16.3%	-1.4%	-1.2%	-2.9%	-2.9%	27.0%	27.3%
	Northern Mariana Islands	-0.3%	-0.5%	0.8%	0.4%	-0.9%	-1.1%			1.6%	-0.6%	-0.4%	0.3%	0.2%	0.1%	7.5%	7.5%
	Oman	-2.7%	-2.6%	-2.5%	-2.6%	-2.8%	-2.8%	-3.3%	-3.3%	-1.3%	-1.3%	-3.1%	-3.1%	-2.3%	-1.9%	3.6%	3.6%
	Palau	-1.8%	-1.9%	-1.3%	-1.4%	-2.1%	-2.3%			-0.9%	-1.6%	-1.9%	-1.9%	-2.5%	-2.4%	23.7%	23.3%
	Poland	-2.5%	-2.6%	-1.2%	-1.3%	-2.9%	-2.9%			-2.6%	-2.7%	-2.2%	-2.1%	-3.0%	-3.1%	6.0%	4.4%
	Portugal	-2.3%	-2.5%	-1.3%	-1.9%	-3.0%	-3.0%			-2.3%	-2.3%	-2.4%	-2.7%	-3.0%	-3.0%	1.0%	-0.6%
	Republic of Moldova	-2.2%	-2.2%	-2.7%	-2.9%	-2.5%	-2.4%			-0.6%	-0.6%	-1.3%	-1.2%	-2.8%	-2.9%	3.2%	-0.8%
	Romania	-2.8%	-2.7%	-2.7%	-2.7%	-2.8%	-2.7%			-1.5%	-1.1%	-1.2%	-2.2%	-2.9%	-3.0%	-3.2%	-3.2%
	Russian Federation	-2.0%	-2.2%	-2.0%	-2.3%	-2.6%	-2.7%			-0.3%	-0.7%	-2.4%	-2.1%	-2.3%	-2.5%	4.2%	1.8%
	Saint Kitts and Nevis	-1.8%	-1.7%	-2.5%	-2.6%	-1.9%	-1.8%			-0.0%	-0.0%	-2.2%	-1.6%	-2.6%	-2.7%	8.1%	8.0%
	Saudi Arabia	-3.0%	-3.0%	-3.0%	-3.0%	-3.1%	-3.1%	-3.3%	-3.3%	-2.5%	-2.5%	-3.3%	-3.3%	-2.8%	-2.8%	1.7%	1.5%
	Serbia	-2.7%	-2.8%	-0.8%	-0.6%	-3.1%	-3.2%			-2.7%	-2.9%	-2.4%	-2.4%	-2.9%	-3.1%	3.2%	1.6%
	Seychelles	-1.5%	-1.6%	-1.0%	-1.2%	-0.4%	-0.5%			-0.6%	-1.1%	-2.2%	-2.3%	-3.1%	-3.1%	0.7%	0.8%
	Spain	-1.6%	-1.8%	-0.4%	-0.5%	-2.7%	-2.8%			-1.3%	-1.3%	-0.8%	-1.0%	-2.8%	-2.8%	-2.7%	-2.8%
	Sri Lanka	-2.5%	-2.4%	-2.6%	-2.6%	-2.7%	-2.7%	-3.3%	-3.3%	-0.7%	-0.8%	-3.1%	-3.1%	-2.6%	-2.6%	-0.4%	-0.4%
	Trinidad and Tobago	-1.5%	-1.6%	-1.9%	-1.9%	-2.2%	-2.1%			-0.5%	-0.8%	-1.2%	-1.4%	-2.4%	-2.6%	2.7%	0.6%
	Turkey	-3.1%	-3.0%	-2.6%	-2.6%	-3.2%	-3.2%	-3.3%	-3.3%	-1.6%	-1.6%	-3.2%	-3.2%	-3.1%	-3.0%	45.7%	46.2%
	Ukraine	-1.1%	-1.6%	-1.4%	-1.8%	-2.0%	-2.5%			-0.2%	-0.4%	1.9%	0.9%	-1.0%	-1.8%	10.8%	1.6%
	United States Virgin Islands	-1.4%	-1.8%	-1.3%	-1.8%	-2.2%	-2.6%			-0.7%	-0.5%	-1.8%	-2.0%	-2.1%	-2.7%	-0.1%	-1.5%
	Uruguay	-2.1%	-2.1%	-2.3%	-2.6%	-2.3%	-2.2%			-1.6%	-1.6%	-1.6%	2.1%	-2.7%	-2.8%	0.7%	3.1%

SDI	Country	Tuberculosis Female	Tuberculosis Male	Neglected Tropical diseases Female	Neglected Tropical diseases Male	Infectious skin conditions Female	Infectious skin conditions Male	Sexually transmitted infections excluding HIV Female	Sexually transmitted infections excluding HIV Male	Upper respiratory infections Female	Upper respiratory infections Male	Other unspecified infectious diseases Female	Other unspecified infectious diseases Male	Hepatitis Female	Hepatitis Male	Rheumatic heart disease Female	Rheumatic heart disease Male
High-middle SDI	American Samoa	-2.3%	-2.6%	1.5%	-0.3%	0.0%	-0.0%	-1.2%	-1.1%	0.1%	-0.1%	-0.7%	-0.4%	-1.4%	-0.9%	-1.6%	-1.8%
	Antigua and Barbuda	-2.0%	-1.6%	-0.7%	0.0%	0.2%	0.6%	-2.7%	-2.5%	-0.1%	-0.0%	-0.7%	0.3%	-2.7%	-2.3%	-1.4%	-0.9%
	Argentina	-3.0%	-3.1%	-1.9%	-1.8%	0.0%	0.1%	-2.3%	-2.3%	-0.2%	-0.1%	-1.0%	-0.6%	-3.0%	-2.6%	-2.4%	-2.5%
	Bahamas	-2.7%	-2.6%	-0.2%	-0.5%	-0.1%	-0.1%	-2.7%	-2.4%	-0.8%	-0.0%	-0.3%	-0.0%	-2.8%	-2.6%	-1.8%	-1.4%
	Bahrain	-2.8%	-2.8%	-1.6%	-1.5%	-1.3%	-0.7%	-0.6%	-0.6%	-0.0%	-0.0%	-1.7%	-1.5%	-2.7%	-2.3%	-2.1%	-2.7%
	Barbados	-2.4%	-2.4%	3.7%	2.3%	0.0%	-0.3%	-2.5%	-2.6%	-0.3%	-0.1%	-0.2%	0.1%	-2.7%	-2.6%	-1.9%	-1.0%
	Belarus	-2.4%	-2.5%	-1.4%	-1.4%	-0.0%	-0.0%	-2.9%	-2.8%	-1.2%	-1.5%	2.8%	3.1%	-2.1%	-1.5%	-1.2%	-1.5%
	Bosnia and Herzegovina	-2.8%	-2.8%	-1.6%	-1.8%	0.0%	0.0%	-1.6%	-1.5%	-0.7%	-0.2%	-1.3%	-1.1%	-1.7%	-2.0%	-0.7%	-2.3%
	Bulgaria	-2.6%	-2.6%	-2.2%	-2.5%	-0.0%	-0.1%	-2.9%	-1.9%	-0.8%	-1.1%	-1.5%	-1.5%	-1.8%	-1.2%	-2.9%	-3.0%
	Chile	-2.8%	-3.0%	-2.4%	-2.3%	0.0%	-0.2%	-3.0%	-2.8%	-0.6%	-0.5%	-0.4%	-0.3%	-3.0%	-2.8%	-2.6%	-2.5%
	Cook Islands	-3.1%	-3.0%	-3.1%	-3.2%	-0.1%	-0.1%	-1.4%	-1.3%	0.0%	-0.1%	-1.1%	-1.3%	-1.4%	-1.9%	-3.1%	-3.0%
	Croatia	-2.6%	-3.0%	-1.3%	-1.3%	-0.0%	-0.0%	-3.0%	-3.0%	-0.1%	-0.0%	-1.1%	-1.0%	-0.9%	-1.6%	-1.8%	-1.8%
	Dominica	-1.6%	-1.2%	1.0%	-0.3%	0.2%	0.3%	-0.1%	0.0%	-0.5%	-0.2%	0.6%	2.1%	-2.6%	-2.4%	-0.9%	-0.7%
	Georgia	-2.5%	-2.7%	0.2%	0.6%	-0.0%	0.0%	-2.3%	2.0%	-0.6%	-0.9%	1.2%	2.0%	-2.3%	-1.0%	-0.7%	-0.5%
	Greece	-2.4%	-2.6%	-1.3%	-1.1%	-0.0%	-0.0%	-2.9%	-3.1%	-0.1%	-0.1%	-0.6%	-0.4%	1.5%	1.5%	-0.6%	-0.9%
	Greenland	-3.1%	-3.0%	-0.9%	-0.9%	0.0%	-0.1%	-0.9%	-1.2%	-0.0%	-0.0%	-1.9%	-2.2%	-3.0%	-2.1%	-3.0%	-2.5%
	Hungary	-2.4%	-2.7%	-1.2%	-1.3%	0.0%	0.0%	-3.2%	-3.2%	-2.4%	-2.5%	-1.3%	-1.3%	-2.3%	-2.5%	-0.4%	-1.9%
	Israel	-2.8%	-3.0%	-1.6%	-1.4%	-0.0%	-0.1%	-3.0%	-3.2%	-0.1%	-0.0%	-0.4%	-0.0%	-3.1%	-3.1%	-2.6%	-2.5%
	Italy	-2.8%	-3.0%	-1.7%	-1.3%	0.0%	0.0%	-3.1%	-3.1%	-0.5%	-0.6%	-0.7%	-0.6%	-0.3%	-0.6%	-2.4%	-2.5%
	Jordan	-2.9%	-2.8%	-2.0%	-2.1%	-0.1%	-0.0%	-0.9%	-0.8%	-0.1%	-0.1%	-1.7%	-1.8%	-2.6%	-2.9%	-2.5%	-2.2%
	Kazakhstan	-2.9%	-3.1%	-1.4%	-1.2%	0.0%	-0.0%	-3.0%	-2.1%	-1.7%	-1.1%	4.0%	5.7%	-3.1%	-3.1%	-2.7%	-2.8%
	Lebanon	-3.0%	-3.0%	-2.3%	-2.5%	-0.0%	-0.0%	-0.5%	-0.4%	-0.0%	-0.0%	-1.6%	-1.8%	-2.9%	-2.7%	-2.8%	-2.6%
	Libya	-2.9%	-2.7%	-1.5%	-1.7%	0.0%	0.0%	-0.4%	-0.6%	-0.0%	-0.1%	-1.2%	-1.6%	-3.0%	-2.9%	-2.6%	-2.5%
	Malaysia	-2.8%	-2.9%	-2.1%	-1.9%	-0.0%	-0.0%	-1.7%	-1.7%	-0.1%	-0.3%	-0.5%	-0.5%	-2.4%	-2.3%	-2.6%	-2.8%
	Malta	-2.1%	-2.5%	-1.8%	-1.9%	0.0%	0.0%	-3.0%	-3.0%	-0.4%	-0.7%	-0.9%	-0.8%	-1.9%	-2.1%	-0.8%	-1.8%
	Mauritius	-2.1%	-2.3%	-0.9%	-0.9%	0.1%	0.1%	-3.2%	-2.5%	-0.1%	-0.2%	-0.8%	-0.7%	-1.7%	-2.2%	-2.5%	-2.4%
	Montenegro	-3.0%	-2.9%	-0.9%	-1.1%	-0.0%	-0.0%	-0.6%	-0.6%	-0.8%	-0.1%	-1.5%	-1.7%	-2.2%	-1.9%	-2.6%	-2.4%
	Niue	-2.2%	-2.2%	0.1%	-0.2%	-0.0%	-0.1%	0.5%	0.5%	0.0%	-0.1%	-0.7%	-0.2%	-2.0%	-1.8%	-1.8%	-0.3%
	North Macedonia	-3.2%	-3.2%	-1.4%	-1.2%	-0.2%	-0.2%	-0.4%	-0.4%	-0.1%	-0.7%	-2.0%	-2.2%	-2.1%	-1.8%	-2.4%	-2.7%
	Northern Mariana Islands	-1.9%	-2.3%	0.7%	0.3%	0.2%	0.2%	-0.4%	-0.6%	0.1%	-0.0%	0.7%	0.4%	0.4%	-0.4%	-0.0%	0.7%
	Oman	-3.1%	-3.1%	-2.3%	-2.4%	-0.2%	-0.3%	-1.7%	-1.7%	-0.4%	-0.1%	-2.0%	-2.1%	-2.9%	-2.8%	-2.8%	-2.5%
	Palau	-2.6%	-2.6%	0.3%	-0.2%	0.0%	0.0%	0.3%	0.1%	0.1%	-0.1%	-0.9%	-1.1%	-2.2%	-2.6%	-2.3%	-2.0%
	Poland	-2.9%	-3.0%	-1.5%	-1.5%	-0.2%	-0.2%	-3.3%	-3.2%	-1.3%	-1.6%	-1.7%	-1.6%	-1.7%	-1.7%	-2.9%	-2.9%
	Portugal	-3.1%	-3.1%	-2.3%	-2.3%	-0.0%	-0.0%	-3.3%	-3.1%	-1.4%	-1.4%	-1.5%	-1.5%	-2.8%	-3.0%	-2.9%	-2.7%
	Republic of Moldova	-2.3%	-2.7%	-1.8%	-1.7%	-0.2%	-0.5%	-3.3%	-1.9%	-1.9%	-1.1%	3.9%	3.7%	-3.3%	-3.3%	-1.8%	-1.6%
	Romania	-2.6%	-2.8%	-1.8%	-2.1%	-0.1%	-0.2%	-2.7%	-1.4%	-2.1%	-2.1%	-2.6%	-2.5%	-3.3%	-3.3%	-1.2%	-1.8%
	Russian Federation	-2.3%	-2.7%	-1.2%	-1.2%	-0.2%	-0.2%	-1.8%	-1.3%	-1.9%	-2.0%	-0.9%	-0.8%	-2.5%	-2.1%	-2.0%	-2.5%
	Saint Kitts and Nevis	-2.9%	-2.8%	-1.0%	-1.1%	-0.0%	-0.4%	-2.9%	-2.7%	-0.4%	-1.2%	-1.0%	-0.6%	-3.1%	-2.6%	-2.9%	-2.7%
	Saudi Arabia	-3.2%	-3.2%	-2.6%	-2.7%	-0.0%	-0.0%	-2.0%	-2.0%	-0.1%	-0.1%	-1.6%	-1.8%	-3.1%	-3.1%	-3.2%	-3.2%
	Serbia	-3.2%	-3.2%	-2.2%	-2.5%	-0.1%	-0.0%	-1.4%	-1.8%	-0.1%	-0.4%	-2.0%	-2.4%	-2.7%	-3.0%	-2.9%	-3.0%
	Seychelles	-1.0%	-1.6%	-1.0%	-0.6%	0.0%	0.0%	-0.4%	-0.5%	-0.1%	-0.3%	0.2%	1.7%	-2.6%	-2.6%	-2.4%	-2.6%
	Spain	-2.8%	-3.1%	-2.1%	-1.9%	0.0%	0.0%	-3.2%	-3.2%	-0.2%	-0.2%	-0.7%	-0.8%	-1.8%	-2.3%	-2.1%	-2.5%
	Sri Lanka	-2.9%	-2.9%	-1.3%	-1.2%	-0.0%	-0.0%	-1.6%	-1.6%	-0.4%	-0.3%	-1.9%	-1.8%	-2.4%	-2.1%	-2.6%	-2.5%
	Trinidad and Tobago	-2.5%	-2.1%	-0.1%	0.3%	-0.0%	-0.0%	-2.8%	-1.2%	-0.1%	-0.1%	-0.1%	-0.1%	-2.8%	-2.6%	-2.0%	-2.0%
	Turkey	-3.2%	-3.3%	-2.5%	-2.6%	-0.2%	-0.3%	-1.8%	-1.7%	-0.6%	-0.4%	-2.0%	-1.9%	-3.1%	-3.1%	-2.8%	-2.0%
	Ukraine	0.7%	-1.1%	-0.6%	-0.9%	0.0%	0.0%	-2.2%	-2.0%	-0.0%	-0.1%	12.2%	8.6%	-2.4%	-2.7%	0.8%	1.8%
	United States Virgin Islands	-2.5%	-2.8%	-0.1%	-0.7%	0.1%	-0.3%	-1.1%	-1.2%	-0.7%	-0.1%	-0.6%	-1.2%	-2.4%	-1.9%	-2.4%	-2.5%
	Uruguay	-2.7%	-2.8%	-1.1%	-0.9%	0.0%	-0.3%	-3.2%	-3.3%	-0.3%	-0.1%	-1.1%	-1.2%	-3.0%	-2.7%	-2.3%	-2.2%

<5 year

High SDI

Country	Total communicable Female	Total communicable Male	Enteric infections Female	Enteric infections Male	Lower respiratory infections Female	Lower respiratory infections Male	Malaria Female	Malaria Male
Andorra	-2.0%	-1.9%	-0.0%	-0.3%	-2.7%	-2.7%		
Australia	-1.3%	-1.6%	0.6%	0.6%	-2.1%	-2.1%		
Austria	-1.2%	-1.6%	0.8%	0.7%	-2.8%	-2.8%		
Belgium	-1.0%	-1.4%	0.4%	0.3%	-2.2%	-2.2%		
Bermuda	-1.5%	-2.0%	-0.7%	-1.8%	-1.7%	-2.4%		
Brunei Darussalam	-0.4%	-0.4%	0.3%	0.0%	-0.4%	-0.4%		
Canada	-0.6%	-0.8%	1.6%	1.3%	-2.0%	-2.1%		
Cyprus	-2.3%	-2.3%	-0.0%	-0.1%	-3.0%	-3.0%		
Czechia	-1.7%	-1.8%	0.2%	0.3%	-2.8%	-2.7%		
Denmark	-1.6%	-1.7%	0.2%	0.1%	-2.6%	-2.5%		
Estonia	-2.1%	-2.6%	-0.2%	-1.3%	-2.6%	-2.8%		
Finland	-1.4%	-1.5%	-0.3%	-0.3%	-2.9%	-2.9%		
France	-1.4%	-1.7%	-1.0%	-1.2%	-2.3%	-2.5%		
Germany	-1.2%	-1.6%	0.3%	0.2%	-2.4%	-2.5%		
Guam	-0.4%	-0.2%	0.5%	0.0%	-0.5%	-0.2%		
Iceland	-1.5%	-1.5%	0.1%	0.0%	-2.4%	-2.4%		
Ireland	-1.2%	-1.5%	0.8%	0.7%	-2.6%	-2.7%		
Japan	-1.4%	-1.6%	-1.1%	-1.2%	-2.0%	-2.1%		
Kuwait	-2.1%	-1.9%	-1.8%	-1.5%	-2.4%	-2.3%		
Latvia	-2.2%	-2.3%	-1.3%	-1.5%	-2.4%	-2.6%		
Lithuania	-1.4%	-1.7%	-1.1%	-1.5%	-1.9%	-2.3%		
Luxembourg	-1.4%	-1.8%	-0.1%	-0.3%	-2.5%	-2.8%		
Monaco	-1.6%	-1.8%	0.1%	-0.0%	-2.6%	-2.6%		
Netherlands	-0.9%	-1.3%	0.7%	0.5%	-2.2%	-2.3%		
New Zealand	-1.4%	-1.8%	0.4%	0.4%	-2.6%	-2.8%		
Norway	-1.3%	-1.7%	0.4%	0.2%	-2.8%	-2.8%		
Puerto Rico	-1.0%	-1.0%	1.1%	1.2%	-2.0%	-2.3%		
Qatar	-2.7%	-2.6%	-1.7%	-1.4%	-2.8%	-2.7%		
Republic of Korea	-2.5%	-2.5%	-2.7%	-2.7%	-3.1%	-3.1%	-3.1%	-3.2%
San Marino	-1.2%	-1.3%	0.5%	0.3%	-2.3%	-2.5%		
Singapore	-2.3%	-2.5%	-2.8%	-2.8%	-2.8%	-2.8%		
Slovakia	-2.1%	-2.1%	-0.1%	-0.2%	-2.6%	-2.7%		
Slovenia	-1.6%	-1.9%	-0.5%	-0.3%	-2.9%	-3.0%		
Sweden	-1.1%	-1.3%	0.5%	0.2%	-2.6%	-2.5%		
Switzerland	-1.6%	-1.8%	-0.0%	-0.5%	-2.8%	-2.9%		
Taiwan (Province of China)	-1.4%	-1.5%	-1.2%	-1.3%	-2.4%	-2.3%		
United Arab Emirates	-2.6%	-2.5%	-1.1%	-1.6%	-3.0%	-3.0%	-3.3%	-3.3%
United Kingdom	-1.4%	-1.6%	0.5%	0.3%	-2.2%	-2.3%		
United States of America	-1.3%	-1.4%	-0.6%	-1.1%	-2.0%	-2.0%		

Country	Neonatal sepsis and other neonatal infections Female	Neonatal sepsis and other neonatal infections Male	Vaccine Preventable disease Female	Vaccine Preventable disease Male	Meningitis & Encephalitis Female	Meningitis & Encephalitis Male	HIV/AIDS Female	HIV/AIDS Male	Tuberculosis Female	Tuberculosis Male	Neglected Tropical diseases Female	Neglected Tropical diseases Male
Andorra	-1.6%	-1.7%	-3.1%	-3.1%	-2.4%	-2.7%	-2.2%	-2.2%	-2.9%	-2.9%	-1.5%	-1.3%
Australia	-2.3%	-2.6%	-0.5%	-0.1%	-2.4%	-2.6%	-0.7%	-2.8%	-2.1%	-2.5%	-1.3%	-1.1%
Austria	1.1%	-0.8%	1.9%	1.5%	-2.4%	-2.5%	-1.7%	-2.4%	-2.7%	-2.5%	-1.7%	-1.6%
Belgium	-1.2%	-2.1%	-2.0%	-2.0%	-2.1%	-2.1%	-2.4%	-2.6%	-2.7%	-2.8%	-1.8%	-1.3%
Bermuda	-1.3%	-0.8%	-0.6%	-0.5%	-2.5%	-2.9%	-2.1%	-2.3%	-2.3%	-2.3%	-1.1%	-0.9%
Brunei Darussalam	0.7%	0.6%	-1.8%	-2.1%	-1.8%	-1.2%	9.6%	10.0%	-2.4%	-2.5%	-1.1%	-0.9%
Canada	-0.2%	-0.9%	0.1%	-0.2%	-2.2%	-1.9%	0.3%	-2.5%	-2.1%	-2.3%	-1.1%	-0.7%
Cyprus	-2.3%	-2.4%	-3.2%	-3.2%	-3.0%	-3.0%	2.4%	2.0%	-2.8%	-3.1%	-2.3%	-2.4%
Czechia	3.1%	3.2%	2.5%	1.8%	-2.6%	-2.6%	6.9%	3.9%	-2.1%	-2.6%	-1.4%	-1.4%
Denmark	-0.5%	-1.5%	-1.5%	-1.9%	-3.0%	-3.0%	6.5%	5.8%	-2.8%	-2.8%	-1.6%	-1.2%
Estonia	-2.2%	-2.8%	-3.0%	-2.9%	-2.8%	-2.9%	21.9%	34.4%	-2.6%	-2.9%	-1.5%	-1.6%
Finland	-1.6%	-1.3%	-0.8%	-0.3%	-2.7%	-2.7%	-2.9%	-2.3%	-2.0%	-3.0%	-1.8%	-1.6%
France	-1.4%	-1.5%	-1.7%	-1.6%	-2.3%	-2.4%	-2.8%	-3.0%	-2.6%	-2.9%	-1.8%	-1.8%
Germany	-1.5%	-1.9%	-1.5%	-1.8%	-2.7%	-2.8%	-2.6%	-2.7%	-2.7%	-2.9%	-1.6%	-1.5%
Guam	-0.4%	0.2%	-0.8%	-0.7%	-0.4%	-0.4%	11.5%	11.6%	-1.5%	-1.6%	-0.2%	-0.3%
Iceland	-1.7%	-2.4%	2.4%	2.0%	-2.8%	-2.6%	-2.1%	-2.3%	-2.8%	-2.6%	-1.6%	-1.1%
Ireland	1.4%	0.3%	-2.5%	-2.4%	-2.7%	-2.8%	-1.0%	-2.4%	-2.6%	-2.9%	-1.9%	-1.5%
Japan	-1.9%	-1.8%	-2.6%	-2.7%	-2.2%	-2.4%	3.8%	4.3%	-2.3%	-2.8%	-1.5%	-1.4%
Kuwait	-1.7%	-1.7%	-2.8%	-2.8%	-2.3%	-2.2%	0.1%	-2.5%	-2.9%	-2.8%	-1.3%	-1.3%
Latvia	-1.7%	-1.5%	-2.3%	-1.9%	-2.7%	-3.0%	7.3%	1.1%	-2.7%	-2.8%	-1.1%	-0.2%
Lithuania	7.5%	3.9%	-2.6%	-2.3%	-2.6%	-2.8%	0.8%	15.7%	-2.4%	-2.5%	-0.8%	-0.9%
Luxembourg	-2.3%	-2.3%	-2.1%	-2.3%	-2.6%	-2.9%	-2.5%	-2.9%	-2.5%	-2.8%	-2.0%	-1.7%
Monaco	-1.6%	-1.4%	-1.5%	-1.6%	-2.4%	-2.8%	-2.9%	-2.9%	-2.9%	-3.0%	-1.7%	-1.4%
Netherlands	0.7%	-0.8%	2.3%	1.4%	-2.5%	-2.6%	-2.4%	-3.0%	-2.7%	-2.9%	-1.3%	-0.6%
New Zealand	0.7%	2.1%	-1.6%	-1.7%	-2.1%	-2.5%	-0.4%	-2.3%	-2.7%	-2.8%	-1.1%	-0.7%
Norway	-0.4%	-2.1%	-1.9%	-1.7%	-2.9%	-2.9%	0.2%	-2.8%	-2.3%	-2.9%	-1.6%	-1.6%
Puerto Rico	-0.3%	0.9%	-1.1%	-1.1%	-2.5%	-2.8%	-1.8%	-2.3%	-2.7%	-2.8%	-0.2%	-0.3%
Qatar	-1.9%	-1.7%	-3.1%	-3.2%	-2.8%	-2.5%	-2.8%	-2.8%	-3.0%	-3.0%	-1.8%	-1.9%
Republic of Korea	-2.0%	-1.8%	-3.0%	-3.0%	-2.8%	-2.8%	-1.3%	1.4%	-3.2%	-3.2%	-1.5%	-1.8%
San Marino	-1.4%	-1.4%	5.7%	5.5%	-2.3%	-2.2%	-2.5%	-2.5%	-2.8%	-2.8%	-1.5%	-1.5%
Singapore	0.5%	-1.5%	-1.6%	-1.6%	-2.8%	-3.0%	1.1%	-0.2%	-3.1%	-3.2%	-1.8%	-1.6%
Slovakia	-0.7%	-0.3%	6.0%	5.5%	-2.3%	-1.9%	5.0%	5.3%	-2.6%	-2.9%	-1.2%	-1.2%
Slovenia	0.2%	0.4%	5.9%	6.1%	-2.8%	-2.9%	2.7%	-2.5%	-2.6%	-2.9%	-1.9%	-2.1%
Sweden	-1.3%	-1.4%	-0.2%	-0.6%	-2.5%	-2.5%	-1.3%	-2.8%	-2.5%	-2.8%	-1.4%	-1.1%
Switzerland	-1.8%	-1.1%	-1.4%	-1.8%	-3.0%	-3.0%	4.6%	5.1%	-2.6%	-2.7%	-1.2%	-1.3%
Taiwan (Province of China)	14.8%	9.7%	-2.7%	-2.8%	-2.3%	-2.3%	8.1%	27.6%	-2.5%	-2.7%	-1.7%	-1.8%
United Arab Emirates	-2.0%	-1.9%	-3.0%	-3.0%	-2.9%	-2.9%	7.3%	7.4%	-3.0%	-3.0%	-1.2%	-1.4%
United Kingdom	-2.0%	-2.2%	-1.9%	-1.6%	-2.5%	-2.5%	-0.4%	-2.5%	-2.4%	-2.6%	-0.7%	-0.8%
United States of America	-0.8%	-0.9%	-0.5%	-0.7%	-2.3%	-2.4%	-3.1%	-3.1%	-2.6%	-2.6%	0.6%	0.9%

Country	Infectious skin conditions Female	Infectious skin conditions Male	Sexually transmitted infections excluding HIV Female	Sexually transmitted infections excluding HIV Male	Upper respiratory infections Female	Upper respiratory infections Male	Other unspecified infectious diseases Female	Other unspecified infectious diseases Male	Hepatitis Female	Hepatitis Male	Rheumatic heart disease Female	Rheumatic heart disease Male
Andorra	0.0%	0.0%	-1.2%	-1.0%	-0.3%	-0.2%	-1.8%	-2.0%	-2.8%	-2.9%	-2.9%	-2.3%
Australia	0.0%	0.0%	-3.2%	-2.9%	-0.3%	-0.3%	0.1%	-0.5%	-1.2%	-0.5%	-2.1%	-2.0%
Austria	0.0%	-0.0%	-3.0%	-3.1%	-0.3%	-0.6%	0.6%	0.0%	-1.7%	-1.1%	-2.4%	-2.2%
Belgium	0.0%	0.0%	-3.1%	-3.2%	-0.3%	-0.2%	0.1%	0.0%	-2.7%	-2.8%	-0.5%	-1.6%
Bermuda	0.1%	-0.2%	-2.9%	-2.8%	-0.1%	-0.1%	-1.3%	-1.4%	-2.4%	-0.9%	-2.9%	-2.7%
Brunei Darussalam	0.0%	0.0%	-0.0%	-0.1%	-0.4%	-0.0%	0.7%	1.3%	-2.0%	-1.4%	-2.6%	-2.2%
Canada	0.1%	0.1%	-2.9%	-2.8%	-0.2%	-0.2%	-0.3%	-0.2%	-1.7%	-1.7%	-1.6%	-1.4%
Cyprus	0.0%	-0.0%	-2.0%	-2.0%	-0.0%	-0.0%	-1.8%	-2.3%	-2.4%	-2.4%	-2.5%	-3.0%
Czechia	-0.0%	-0.0%	-3.0%	-3.2%	-1.6%	-1.4%	-1.6%	-1.5%	-1.2%	-2.6%	-2.7%	-2.6%
Denmark	0.0%	0.0%	-2.8%	-2.9%	-0.1%	-0.2%	-0.7%	-0.4%	-2.5%	-1.2%	-2.1%	-2.1%
Estonia	-0.0%	-0.2%	-2.9%	-2.5%	-0.5%	-0.6%	1.9%	0.6%	-3.0%	-3.1%	-1.7%	-1.8%
Finland	0.1%	-0.1%	-3.0%	-3.1%	-0.1%	-0.0%	-1.3%	-1.3%	-2.4%	-2.0%	-1.5%	-1.8%
France	0.0%	-0.0%	-3.0%	-3.0%	-0.9%	-1.2%	-1.1%	-1.1%	-2.1%	-2.6%	-1.5%	-1.7%
Germany	0.0%	0.0%	-2.9%	-3.0%	-0.6%	-0.8%	-1.5%	-1.2%	-2.6%	-2.9%	-2.0%	-2.2%
Guam	0.3%	0.2%	-0.4%	-0.6%	0.0%	-0.0%	0.0%	0.1%	0.2%	0.0%	-0.7%	0.0%
Iceland	0.1%	0.1%	-3.0%	-3.2%	-0.2%	-0.4%	-1.1%	-0.9%	-1.4%	-0.4%	-1.4%	-1.5%
Ireland	-0.0%	-0.0%	-3.1%	-3.1%	-0.1%	-0.1%	-0.6%	-1.0%	-1.4%	-1.7%	-1.6%	-2.1%
Japan	0.0%	0.0%	-3.0%	-3.0%	-0.4%	-0.4%	-0.5%	-0.5%	-1.8%	-2.1%	-1.3%	-1.6%
Kuwait	-0.0%	-0.1%	-2.8%	-3.0%	-0.0%	-0.0%	-0.9%	-0.8%	-3.0%	-2.8%	-3.0%	-2.6%
Latvia	-0.0%	-0.1%	-3.2%	-3.3%	-0.1%	-0.3%	0.9%	4.3%	-3.2%	-3.2%	-1.6%	-1.1%
Lithuania	0.0%	-0.0%	-2.9%	-3.3%	-0.8%	-1.2%	0.5%	1.1%	-3.1%	-3.1%	-1.5%	-1.4%
Luxembourg	0.1%	0.0%	-3.2%	-3.1%	-0.3%	-0.4%	-1.5%	-1.4%	-1.3%	-2.2%	-1.1%	-2.0%
Monaco	0.1%	0.1%	-1.6%	-1.5%	-0.0%	-0.0%	-1.7%	-1.6%	-1.8%	-2.3%	-2.1%	-1.7%
Netherlands	0.1%	-0.0%	-2.9%	-3.1%	-0.8%	-1.0%	0.2%	-0.0%	-1.4%	-1.8%	-1.2%	-1.3%
New Zealand	0.1%	0.0%	-2.9%	-3.1%	-0.2%	-0.5%	-0.3%	-0.8%	-1.7%	-1.6%	-2.0%	-1.5%
Norway	0.1%	0.0%	-3.0%	-1.0%	-0.3%	-0.1%	-0.3%	-0.3%	-1.0%	-1.1%	-2.3%	-2.6%
Puerto Rico	0.0%	-0.0%	-2.8%	-0.9%	-0.0%	-0.2%	-0.1%	0.2%	-2.2%	-2.4%	-2.7%	-2.5%
Qatar	-0.1%	-0.1%	-1.2%	-1.1%	-0.1%	-0.1%	-1.6%	-1.4%	-2.0%	-2.4%	-3.0%	-3.0%
Republic of Korea	0.0%	-0.0%	0.1%	0.4%	-0.3%	-0.3%	-1.2%	-1.3%	-3.0%	-2.9%	-2.8%	-2.7%
San Marino	-0.0%	-0.0%	-0.6%	-0.8%	-1.3%	-0.7%	-1.0%	-1.6%	-2.3%	-2.6%	-2.5%	-2.6%
Singapore	0.2%	-0.1%	-3.2%	-3.3%	-0.0%	-0.0%	-2.1%	-2.1%	-2.2%	-2.9%	-3.0%	-2.7%
Slovakia	-0.0%	-0.0%	-0.6%	-0.6%	-0.3%	-0.5%	-0.6%	-1.3%	-2.2%	-2.3%	-2.4%	-2.2%
Slovenia	-0.0%	-0.0%	-3.0%	-3.1%	-0.5%	-0.6%	-1.3%	-1.1%	-1.4%	-0.8%	-2.0%	-1.4%
Sweden	0.1%	0.1%	-3.0%	-3.1%	-0.5%	-0.2%	-0.3%	-0.5%	-1.1%	-1.2%	-1.5%	-2.0%
Switzerland	0.0%	0.0%	-2.7%	-2.9%	-0.2%	-0.2%	-0.6%	-0.9%	-0.9%	-2.9%	-1.5%	-1.8%
Taiwan (Province of China)	0.0%	0.0%	-2.9%	-2.2%	-1.1%	-1.2%	0.8%	0.7%	-1.0%	-1.7%	-1.7%	-1.1%
United Arab Emirates	0.0%	0.0%	-2.2%	-2.2%	-0.0%	-0.0%	-1.7%	-1.9%	-2.7%	-2.8%	-2.9%	-2.6%
United Kingdom	0.0%	0.0%	-0.2%	-0.2%	-0.5%	-0.7%	-0.6%	-0.8%	-0.3%	0.6%	-2.6%	-2.3%
United States of America	0.1%	0.1%	-2.6%	-2.6%	-0.3%	-0.3%	-0.4%	-0.4%	-1.8%	-1.9%	-1.9%	-1.8%

S13. (A) Communicable disease DALYs by cause for 5-14 year age group by sex in 2019, grouped by SDI. The shading ranges from green which indicates a low number of DALYs per 100,000 for that country with in the disease, while the highest rates are shaded in a dark orange colour and indicates a country has a large DALY burden.

5 to 14 years, DALYs/100,000, for males and females, 2019

Low SDI

	Total commuincable		Enteric infections		Lower respiratory infections		Malaria		Neonatal sepsis and other neonatal infections		Vaccine Preventable disease		Meningitis & Encephalitis	
	Female	Male	Female	Male	Female	Male	Female	Male	Female	Male	Female	Male	Female	Male
Afghanistan	2,996	2,881	536	624	645	638	32	26	8	13	408	323	228	218
Benin	5,754	6,617	1,195	1,490	573	573	1,456	1,820	42	26	742	660	386	446
Burkina Faso	6,439	6,786	1,873	2,046	735	710	1,494	1,537	36	35	383	293	575	589
Burundi	6,286	7,231	1,593	2,188	912	839	1,149	1,307	61	53	187	171	447	445
Central African Republic	9,255	11,659	1,739	2,560	793	1,041	1,666	2,608	14	12	701	668	756	930
Chad	7,487	8,290	1,940	2,322	976	925	606	791	21	24	1,125	968	729	789
Côte d'Ivoire	6,094	7,132	1,121	1,366	519	545	1,875	2,237	43	50	201	172	322	347
Dem. Rep. of the Congo	5,584	6,562	729	1,193	565	541	1,130	1,825	28	28	346	283	450	397
Eritrea	3,814	5,242	1,184	2,142	606	846	122	113	59	61	135	112	316	349
Ethiopia	4,009	4,876	984	1,164	352	435	181	312	79	82	277	231	337	403
Gambia	3,362	4,088	854	1,144	341	403	393	452	79	73	145	126	271	367
Guinea	7,400	8,330	1,839	2,360	1,007	1,003	1,039	1,170	29	28	689	582	708	763
Guinea-Bissau	5,785	6,729	1,139	1,379	519	663	376	432	42	38	1,383	1,408	419	567
Haiti	3,568	3,253	435	431	656	483	126	86	43	30	174	140	269	284
Liberia	6,735	7,493	1,328	1,671	543	504	1,866	2,114	49	49	366	287	429	393
Madagascar	4,773	6,021	1,153	2,559	764	622	606	706	53	53	511	384	322	275
Malawi	5,933	7,082	1,058	1,375	404	539	993	1,273	63	64	143	135	387	514
Mali	6,218	7,979	1,953	3,018	446	377	804	1,332	39	24	631	492	717	980
Mozambique	8,668	10,074	735	1,433	386	518	2,110	2,485	57	60	156	143	139	344
Nepal	2,638	2,902	1,014	1,163	197	175	2	2	118	76	202	162	185	220
Niger	7,060	7,915	1,652	2,185	685	624	1,196	1,590	30	28	1,652	1,404	533	553
Pakistan	5,416	5,887	2,269	2,537	362	555	279	298	45	30	216	167	431	682
Papua New Guinea	4,126	4,387	935	1,183	330	451	352	208	17	25	415	366	189	300
Rwanda	3,365	3,866	735	1,047	445	456	519	599	82	77	127	110	250	268
Senegal	4,034	4,699	1,028	1,396	416	433	611	699	58	63	306	258	415	456
Sierra Leone	7,470	8,843	1,580	2,345	909	852	2,438	2,873	32	27	184	136	623	671
Solomon Islands	2,868	3,148	596	828	364	543	88	69	12	23	133	142	215	179
Somalia	7,797	8,771	1,961	2,131	879	1,004	414	588	29	26	1,648	1,673	678	756
South Sudan	6,277	7,681	1,362	1,568	499	521	795	974	35	32	320	251	354	445
Togo	5,439	6,107	1,404	1,737	487	520	1,370	1,577	59	50	148	124	301	319
Uganda	5,041	6,117	735	1,090	447	505	1,086	1,373	68	58	458	417	233	288
United Republic of Tanzania	4,489	5,080	1,070	1,340	527	570	469	558	69	58	275	228	200	238
Yemen	1,885	1,865	523	577	305	256	48	70	6	10	188	152	92	90

	HIV/AIDS		Tuberculosis		Neglected Tropical diseases		Infectious skin conditions		Sexually transmitted infections excluding HIV		Upper respiratory infections		Other unspecified infectious diseases		Hepatitis		Rheumatic heart disease		Maternal sepsis and other maternal infections
	Female	Male	Female	Male	Female	Male	Female	Male	Female	Male	Female	Male	Female	Male	Female	Male	Female	Male	Female
Afghanistan	4	4	122	95	382	371	155	160	2	3	129	142	210	141	85	85	47	40	2
Benin	381	388	125	155	367	502	243	274	6	4	93	114	69	62	32	58	43	42	2
Burkina Faso	274	287	200	216	335	484	236	264	8	5	102	125	101	110	36	34	49	51	2
Burundi	478	549	431	328	382	638	332	362	5	6	129	137	54	64	42	39	79	106	5
Central African Republic	909	914	1,326	1,155	655	928	286	315	15	16	178	199	96	115	34	76	75	121	11
Chad	442	428	435	438	623	894	248	279	21	17	106	127	96	96	55	146	54	47	11
Côte d'Ivoire	1,018	1,055	171	193	322	592	250	272	11	9	103	121	75	87	23	49	41	39	1
Dem. Rep. of the Congo	228	240	594	349	914	1,041	265	290	10	8	142	156	95	91	23	45	56	74	10
Eritrea	217	235	248	274	273	392	337	368	7	7	133	144	76	70	28	33	67	95	7
Ethiopia	462	481	135	133	471	766	454	552	9	10	129	142	54	53	29	40	53	71	2
Gambia	301	327	111	152	359	452	238	268	10	9	72	95	121	117	26	66	39	37	3
Guinea	357	374	254	273	945	1,113	248	281	12	9	96	118	74	66	49	147	48	41	5
Guinea-Bissau	723	735	139	176	510	694	250	283	15	14	97	119	92	92	32	82	47	48	2
Haiti	661	742	180	85	220	228	276	300	13	12	161	177	85	101	46	29	218	124	5
Liberia	353	365	130	94	1,175	1,440	239	267	26	20	107	127	44	50	30	72	43	39	7
Madagascar	59	65	262	196	228	289	310	334	20	15	127	137	243	242	28	25	84	119	3
Malawi	1,775	2,009	224	200	229	260	327	357	14	16	141	154	88	62	27	45	58	80	2
Mali	245	237	238	152	438	593	340	383	23	17	89	116	151	151	43	62	50	44	10
Mozambique	3,943	3,747	279	244	259	406	323	348	15	17	125	138	59	73	25	46	56	70	1
Nepal	37	37	134	79	268	417	179	262	3	3	145	152	54	47	64	65	35	41	0
Niger	83	81	197	211	457	670	253	278	5	2	95	123	125	75	38	47	49	45	8
Pakistan	9	9	743	230	283	333	211	256	4	4	143	150	138	420	116	123	166	93	1
Papua New Guinea	242	232	326	126	329	547	344	369	21	17	129	137	60	81	66	63	370	283	1
Rwanda	148	185	123	130	320	337	338	376	8	8	132	141	57	25	27	38	53	70	2
Senegal	146	155	109	113	439	562	240	268	5	3	96	118	102	112	20	26	40	38	2
Sierra Leone	421	377	220	173	542	787	240	269	10	6	87	108	87	105	41	71	51	45	7
Solomon Islands	50	54	25	24	586	468	339	364	37	41	132	140	64	69	51	70	174	137	0
Somalia	253	258	664	539	485	853	332	361	15	22	187	210	87	77	64	133	92	140	9
South Sudan	455	470	244	286	1,553	2,407	339	372	20	20	127	135	74	70	35	52	62	78	2
Togo	589	628	154	152	411	440	241	270	6	4	105	123	99	74	25	49	40	40	1
Uganda	946	1,134	163	197	266	356	328	357	28	28	144	153	52	50	23	28	62	84	2
United Republic of Tanzania	655	720	184	164	363	452	365	401	15	14	135	145	77	82	25	32	58	79	2
Yemen	8	8	50	36	198	197	152	157	3	3	145	155	67	63	31	33	67	57	3

5 to 14 years, DALYs/100,000, for males and females, 2019

		Total communicable		Enteric infections		Lower respiratory infections		Malaria		Neonatal sepsis and other neonatal infections		Vaccine Preventable disease		Meningitis & Encephalitis		HIV/AIDS	
		Female	Male	Female	Male	Female	Male	Female	Male	Female	Male	Female	Male	Female	Male	Female	Male
Low-middle SDI	Angola	4,553	5,334	793	1,057	391	492	832	1,049	30	37	319	290	405	451	573	573
	Bangladesh	3,552	3,400	1,504	1,594	548	391	6	11	184	238	75	80	230	68	3	3
	Belize	1,173	1,150	170	189	131	131	1	1	148	83	4	7	52	54	83	83
	Bhutan	2,547	2,737	975	1,160	167	170	5	5	103	81	15	14	279	319	35	34
	Bolivia	1,632	1,746	282	279	346	381	2	2	94	91	51	42	87	99	53	54
	Cabo Verde	1,511	1,778	409	542	143	151	37	38	107	93	23	20	87	130	140	157
	Cambodia	2,088	2,454	393	527	379	465	22	24	90	116	99	98	64	91	118	125
	Cameroon	5,722	6,341	1,071	1,273	532	530	1,332	1,519	47	48	523	447	354	377	820	867
	Comoros	3,927	4,096	920	1,242	577	540	552	445	87	77	441	364	360	334	1	1
	Congo	4,366	5,186	436	833	282	311	1,116	1,293	39	43	277	250	256	279	869	1,064
	Dem. People's Rep. of Korea	960	1,009	155	173	120	117	2	1	14	16	25	21	80	83	15	15
	Djibouti	3,750	4,308	798	1,113	488	565	211	160	75	71	295	223	302	318	597	650
	Dominican Republic	1,575	1,704	212	266	170	220	0	0	307	261	65	55	99	137	33	34
	El Salvador	1,089	1,164	235	263	127	158	0	0	34	28	27	22	42	46	93	92
	Eswatini	5,718	6,676	719	1,170	482	729	47	37	37	34	119	106	214	352	2,934	2,784
	Ghana	4,192	5,012	911	1,033	296	378	1,006	1,362	104	120	157	139	434	422	473	478
	Guatemala	1,733	1,822	479	544	506	521	2	2	29	25	12	14	76	84	30	39
	Honduras	1,398	1,475	305	384	31	113	2	2	91	74	31	32	74	86	3	3
	India	3,960	3,553	1,686	1,531	337	237	238	239	47	44	80	63	355	280	27	30
	Kenya	4,270	5,192	1,136	1,570	284	364	367	530	92	76	192	176	274	270	1,007	1,115
	Kiribati	3,602	3,936	471	907	271	481	0	0	9	18	360	350	764	554	26	26
	Kyrgyzstan	946	1,010	175	180	207	245	0	0	12	14	1	1	83	109	35	27
	Lao PDR	2,445	2,789	484	678	348	355	10	9	76	105	289	254	155	203	43	44
	Lesotho	7,438	8,786	1,017	1,742	588	1,002	0	0	38	37	97	91	259	420	3,940	3,654
	Maldives	1,201	1,387	263	315	52	38	0	0	84	148	43	35	43	43	3	1
	Marshall Islands	2,108	2,276	280	358	280	452	0	0	15	24	237	223	163	145	42	45
	Mauritania	3,270	3,783	824	1,124	348	315	764	978	102	102	198	154	246	222	2	4
	Micronesia (Fed States of)	1,965	2,213	286	388	217	344	0	0	14	27	110	97	127	114	300	345
	Mongolia	1,017	1,131	189	195	227	282	0	0	13	17	28	23	100	138	2	2
	Morocco	1,180	1,083	270	295	148	121	0	0	9	15	119	94	52	50	28	27
	Myanmar	2,117	2,367	507	522	108	120	24	21	100	122	192	142	98	85	118	124
	Nicaragua	1,035	1,133	206	233	123	160	3	3	44	38	6	6	74	100	61	58
	Nigeria	5,693	6,866	1,836	2,008	267	396	1,483	1,825	45	49	295	304	383	542	183	198
	Palestine	859	935	267	288	132	179	0	0	17	20	19	17	42	39	2	2
	Sao Tome and Principe	1,927	2,274	523	637	356	358	63	67	70	92	101	80	171	102	1	2
	Sudan	1,819	2,167	477	693	275	249	49	71	7	9	171	145	79	92	70	65
	Tajikistan	1,484	1,714	396	390	520	710	1	1	18	22	43	35	74	93	1	1
	Timor-Leste	2,202	2,745	422	726	279	298	3	3	69	85	168	139	138	177	222	227
	Tuvalu	1,650	1,780	238	337	214	308	0	0	19	32	179	155	125	107	36	39
	Vanuatu	2,336	2,607	400	545	318	494	12	12	11	22	256	244	175	165	49	53
	Venezuela	1,320	1,371	206	245	114	142	88	77	263	210	7	11	63	72	29	29
	Zambia	5,594	6,719	965	1,221	382	500	946	1,159	78	76	106	99	341	442	1,551	1,780
	Zimbabwe	7,038	6,998	548	1,035	378	592	885	1,014	42	46	231	203	274	301	3,412	2,372

		Tuberculosis		Neglected Tropical diseases		Infectious skin conditions		Sexually transmitted infections excluding HIV		Upper respiratory infections		Other unspecified infectious diseases		Hepatitis		Rheumatic heart disease		Maternal sepsis and other maternal infections	
		Female	Male	Female	Male	Female	Male	Female	Male	Female	Male	Female	Male	Female	Male	Female	Male	Female	Male
Low-middle SDI	Angola	370	308	270	419	269	295	12	13	143	155	72	76	20	45	53	73	1	0
	Bangladesh	190	159	255	281	202	257	4	4	161	169	45	50	86	56	59	39	0	0
	Belize	19	23	82	83	260	267	1	1	138	146	38	37	11	12	37	34	0	0
	Bhutan	92	51	272	266	200	244	7	7	135	143	162	143	47	57	51	42	0	0
	Bolivia	106	122	93	128	284	291	6	5	136	146	42	57	12	15	37	33	1	0
	Cabo Verde	30	18	122	163	220	243	3	2	91	104	58	62	11	20	32	34	0	0
	Cambodia	183	220	149	179	296	314	1	2	124	138	59	61	66	53	44	39	0	0
	Cameroon	141	148	438	567	214	248	14	11	116	135	57	61	24	71	39	38	3	0
	Comoros	197	161	158	253	331	361	9	8	133	141	71	65	29	30	61	74	0	0
	Congo	221	175	292	330	272	300	7	7	133	144	97	68	17	27	51	63	1	0
	Dem. People's Rep. of Korea	41	39	46	39	283	317	1	2	116	128	28	24	9	13	25	23	0	0
	Djibouti	146	156	213	376	330	358	11	11	130	139	66	62	24	32	56	73	8	0
	Dominican Republic	59	58	130	161	258	264	7	8	129	135	23	21	15	27	67	57	0	0
	El Salvador	7	7	84	88	230	236	1	1	142	152	26	33	9	10	30	28	0	0
	Eswatini	544	703	145	211	210	221	10	9	117	135	56	75	17	29	68	82	1	0
	Ghana	95	145	300	467	204	220	8	9	92	110	54	66	26	29	32	33	0	0
	Guatemala	23	19	127	114	236	241	1	1	134	144	37	34	10	13	31	28	0	0
	Honduras	15	18	375	277	230	237	1	1	148	158	51	46	14	19	28	24	1	0
	India	148	93	292	325	260	260	2	2	143	154	127	107	152	148	64	41	0	0
	Kenya	96	110	222	335	325	336	5	6	168	184	27	30	29	33	45	58	1	0
	Kiribati	485	180	384	423	341	372	20	12	132	140	56	70	73	160	209	241	0	0
	Kyrgyzstan	30	21	74	65	146	151	1	1	93	110	34	27	21	28	34	30	0	0
	Lao PDR	187	237	250	285	298	318	2	3	139	152	43	37	18	22	103	88	0	0
	Lesotho	886	1,120	96	118	209	222	10	9	126	152	71	96	23	30	77	92	3	0
	Maldives	17	16	199	275	297	313	1	2	128	143	26	21	8	8	37	28	0	0
	Marshall Islands	121	58	221	218	337	363	9	8	127	134	33	47	21	37	223	164	0	0
	Mauritania	80	58	229	297	236	264	10	8	93	110	78	79	23	32	37	34	2	0
	Micronesia (Fed States of)	72	35	148	172	334	359	9	8	126	135	28	35	14	25	181	129	0	0
	Mongolia	69	45	43	46	147	161	7	8	87	97	31	30	40	60	35	28	0	0
	Morocco	113	30	75	74	151	155	2	2	129	144	32	29	14	16	37	30	0	0
	Myanmar	151	188	257	419	298	315	3	3	138	151	73	94	12	20	36	38	0	0
	Nicaragua	14	13	78	74	233	242	1	1	133	142	12	15	10	16	35	31	0	0
	Nigeria	104	161	523	697	295	354	8	7	103	126	100	107	27	52	39	38	2	0
	Palestine	8	6	40	36	152	156	1	1	126	137	24	24	12	12	17	16	0	0
	Sao Tome and Principe	34	29	185	364	232	258	5	4	94	105	33	96	14	36	44	42	0	0
	Sudan	34	39	211	356	153	157	6	6	125	137	54	54	51	46	56	50	2	0
	Tajikistan	52	53	60	54	144	149	1	6	79	95	32	29	29	45	34	32	0	0
	Timor-Leste	155	178	166	307	315	334	2	3	131	143	36	44	17	17	77	64	0	0
	Tuvalu	60	24	125	118	335	360	8	7	126	134	30	38	16	28	140	94	0	0
	Vanuatu	140	69	178	213	323	348	14	14	129	137	27	36	34	52	270	204	0	0
	Venezuela	15	12	136	153	227	234	1	1	141	150	15	20	9	11	5	5	1	0
	Zambia	167	159	378	520	330	359	16	15	136	149	120	120	24	41	54	77	1	0
	Zimbabwe	515	534	169	224	210	222	7	7	133	150	49	53	20	30	161	217	4	0

5 to 14 years, DALYs/100,000, for males and females, 2019

Middle SDI

	Total commuincable		Enteric infections		Lower respiratory infections		Malaria		Neonatal sepsis and other neonatal infections		Vaccine Preventable disease		Meningitis & Encephalitis	
	Female	Male	Female	Male	Female	Male	Female	Male	Female	Male	Female	Male	Female	Male
Albania	895	997	248	234	170	225	0	0	6	6	14	13	68	100
Algeria	922	899	261	269	155	118	0	0	14	19	39	32	47	47
Armenia	920	931	232	228	154	201	0	0	121	63	1	1	39	39
Azerbaijan	1,413	1,491	223	234	601	633	0	0	22	26	39	31	144	161
Botswana	2,922	3,442	387	575	607	707	32	28	46	65	97	78	213	263
Brazil	1,243	1,293	191	198	134	143	6	5	84	64	10	10	58	72
China	823	929	96	106	53	71	0	0	139	132	10	10	40	52
Colombia	1,128	1,213	177	199	107	120	7	6	154	158	7	9	67	79
Costa Rica	815	846	160	186	31	38	0	0	57	29	2	2	51	54
Cuba	843	901	132	173	37	42	0	0	88	63	4	6	50	53
Ecuador	1,228	1,294	233	225	218	266	1	1	57	54	37	32	63	73
Egypt	1,142	1,353	259	294	336	431	0	0	7	10	22	20	91	118
Equatorial Guinea	5,313	6,200	258	413	189	233	1,656	2,178	54	55	164	144	161	196
Fiji	1,959	2,393	269	395	313	476	0	0	43	58	77	62	151	203
Gabon	3,225	4,208	208	407	169	286	1,003	1,309	80	76	128	127	170	251
Grenada	1,160	1,170	203	222	153	125	0	0	153	138	6	5	42	39
Guyana	1,625	2,026	236	256	207	217	122	74	89	141	4	5	102	122
Indonesia	1,978	2,325	536	761	99	107	12	7	75	123	86	85	121	175
Iran (Islamic Republic of)	881	955	309	331	116	116	1	1	11	41	13	12	45	46
Iraq	1,105	1,121	267	288	157	149	0	0	23	30	73	61	134	127
Jamaica	1,200	1,172	138	158	75	72	0	0	245	191	1	2	59	61
Mexico	947	961	178	188	80	82	0	0	107	82	9	9	47	50
Namibia	4,032	5,056	423	820	358	560	321	300	47	48	51	43	138	209
Nauru	1,838	2,068	250	324	365	580	0	0	21	34	32	29	160	145
Panama	1,176	1,154	266	278	109	117	1	1	185	124	2	2	58	62
Paraguay	1,150	1,205	174	196	136	129	0	0	14	30	24	19	64	69
Peru	1,652	1,738	237	244	280	290	5	6	279	287	36	28	77	90
Philippines	2,965	3,237	500	593	471	501	3	1	164	158	189	183	233	276
Saint Lucia	1,191	1,109	210	232	96	81	0	0	177	96	4	12	81	74
Samoa	1,653	1,854	229	317	155	240	0	0	16	27	315	304	94	88
South Africa	4,635	5,231	376	401	293	329	29	23	54	60	153	133	98	114
St Vincent & the Grenadines	1,189	1,180	188	195	110	77	0	0	136	132	8	4	58	68
Suriname	1,497	1,507	207	234	113	107	5	5	191	175	18	17	90	108
Syrian Arab Republic	1,458	1,487	289	310	373	368	0	0	7	9	64	51	151	154
Thailand	1,283	1,382	323	385	118	122	1	1	107	97	37	34	68	78
Tokelau	1,370	1,423	202	284	132	148	0	0	20	40	151	130	86	66
Tonga	1,649	1,800	192	266	137	220	0	0	60	60	122	107	294	232
Tunisia	802	837	251	275	105	99	0	0	12	15	33	29	41	43
Turkmenistan	1,169	1,295	154	161	466	535	0	0	20	21	1	2	115	135
Uzbekistan	1,619	1,787	160	166	801	918	0	0	25	32	1	1	144	181
Viet Nam	1,148	1,371	288	345	49	82	2	1	129	134	46	42	80	139

	HIV/AIDS		Tuberculosis		Neglected Tropical diseases		Infectious skin conditions		Sexually transmitted infections excluding HIV		Upper respiratory infections		Other unspecified infectious diseases		Hepatitis		Rheumatic heart disease		Maternal sepsis and other maternal infections
	Female	Male	Female	Male	Female	Male	Female	Male	Female	Male	Female	Male	Female	Male	Female	Male	Female	Male	Female
Albania	1	1	1	2	33	33	164	169	1	1	108	119	40	43	10	14	32	38	0
Algeria	5	5	11	7	42	41	153	157	1	1	123	135	30	29	14	16	26	22	0
Armenia	5	2	10	6	37	49	143	148	1	1	115	122	23	32	9	13	30	25	0
Azerbaijan	2	2	41	36	34	47	143	148	1	2	86	96	21	22	17	24	37	30	0
Botswana	495	615	384	393	180	200	204	215	11	10	118	135	70	79	13	15	63	66	1
Brazil	44	42	12	10	125	164	312	309	2	1	177	191	36	36	10	12	42	36	1
China	10	11	11	13	34	40	280	327	1	1	115	128	9	10	9	12	16	17	0
Colombia	14	14	12	10	150	159	233	238	1	1	169	179	15	27	9	11	4	3	0
Costa Rica	35	35	3	3	47	50	227	234	1	1	141	149	17	21	9	12	34	31	0
Cuba	8	19	2	2	48	55	264	271	1	1	142	147	23	30	8	9	36	31	0
Ecuador	29	33	30	31	65	73	282	289	4	3	143	152	20	19	10	12	37	33	0
Egypt	1	1	9	9	46	70	167	169	1	1	104	118	28	40	16	26	54	47	0
Equatorial Guinea	1,875	2,000	113	74	322	336	257	279	16	13	130	137	63	62	12	26	43	53	1
Fiji	27	25	72	28	208	320	360	383	14	5	122	128	59	81	18	27	226	202	0
Gabon	423	460	111	123	389	546	263	289	6	6	135	145	83	98	13	28	45	57	1
Grenada	18	29	10	6	80	77	270	288	1	1	135	144	36	44	9	11	44	41	0
Guyana	143	96	43	49	160	522	262	269	2	2	140	145	48	64	13	16	53	49	0
Indonesia	24	20	131	152	339	321	311	327	3	6	159	172	31	25	32	36	19	8	0
Iran (Islamic Republic of)	4	4	9	7	20	22	155	159	1	1	133	146	22	23	13	16	28	28	0
Iraq	3	3	24	16	97	105	145	149	1	1	122	134	21	20	16	17	23	19	0
Jamaica	99	72	3	3	86	86	254	269	1	1	138	145	28	44	10	9	64	59	0
Mexico	18	22	9	7	56	59	239	246	1	1	153	165	21	24	12	14	14	12	0
Namibia	1,547	1,856	310	367	371	340	206	217	8	8	121	139	66	76	12	13	52	59	1
Nauru	35	38	80	34	156	163	337	362	10	8	121	128	28	38	18	31	224	152	0
Panama	25	35	53	39	42	45	231	237	1	1	139	151	22	22	10	11	34	29	0
Paraguay	104	104	18	17	66	87	302	298	10	10	163	177	27	26	8	9	38	34	0
Peru	113	114	51	43	65	100	285	292	3	2	150	158	30	42	11	14	30	27	0
Philippines	105	121	213	263	467	487	317	336	4	4	159	176	24	32	11	14	105	91	0
Saint Lucia	19	15	12	12	101	79	265	275	2	1	136	145	33	43	9	10	45	35	0
Samoa	46	50	44	21	126	160	330	357	6	6	124	132	29	33	12	24	127	95	0
South Africa	2,678	3,218	355	316	121	124	208	219	12	12	133	152	71	76	9	10	46	43	0
St Vincent & the Grenadines	88	87	13	13	79	74	266	277	1	2	140	146	40	52	9	10	51	42	0
Suriname	100	66	13	12	242	231	276	294	4	4	136	143	48	59	11	13	44	39	0
Syrian Arab Republic	1	1	13	11	193	187	144	148	2	2	122	135	35	34	13	21	53	55	0
Thailand	11	10	20	20	78	75	294	311	2	2	173	188	16	21	8	9	27	28	0
Tokelau	36	39	30	10	124	116	335	359	7	6	124	130	25	31	14	20	84	42	0
Tonga	13	18	32	17	151	147	334	360	4	4	125	132	22	37	71	126	91	75	0
Tunisia	4	5	7	5	33	35	152	156	1	1	124	135	18	17	12	15	8	8	0
Turkmenistan	3	4	40	32	42	40	144	149	1	1	87	98	28	28	28	56	39	33	0
Uzbekistan	8	18	41	32	81	70	143	149	1	1	89	97	52	45	26	38	46	39	0
Viet Nam	1	2	38	45	66	110	295	312	1	2	119	130	16	12	12	13	8	3	0

5 to 14 years, DALYs/100,000, for males and females, 2019

		Total commuincable		Enteric infections		Lower respiratory infections		Malaria		Neonatal sepsis and other neonatal infections		Vaccine Preventable disease		Meningitis & Encephalitis		HIV/AIDS		Tuberculosis		Neglected Tropical diseases		Infectious skin conditions		Sexually transmitted infections excluding HIV		Upper respiratory infections		Other unspecified infectious diseases		Hepatitis		Rheumatic heart disease		Maternal sepsis and other maternal infections
		Female	Male	Female	Male	Female	Male	Female	Male	Female	Male	Female	Male	Female	Male	Female	Male	Female	Male	Female	Male	Female	Male	Female	Male	Female	Male	Female	Male	Female	Male	Female	Male	Female
High-middle SDI	American Samoa	1,215	1,367	191	285	122	144	0	0	5	12	111	94	95	71	10	14	11	5	81	132	332	364	6	6	117	124	12	29	13	19	107	69	0
	Antigua and Barbuda	898	1,098	159	177	72	97	0	0	11	81	6	3	42	66	33	55	11	8	98	112	257	281	1	1	133	140	29	35	9	11	38	31	0
	Argentina	801	712	66	104	91	88	0	0	6	5	6	5	45	49	19	19	8	6	30	40	335	193	1	1	143	149	18	20	8	9	25	22	1
	Bahamas	1,083	1,017	151	168	83	85	0	0	143	67	2	6	49	57	119	95	11	10	53	50	258	267	1	1	135	137	32	33	9	10	36	32	0
	Bahrain	702	741	267	275	56	63	0	0	5	5	10	9	18	28	6	4	7	7	24	20	153	162	1	2	121	131	20	19	9	13	7	5	0
	Barbados	1,311	1,168	182	195	86	64	0	0	280	209	6	5	73	65	47	32	6	4	121	80	272	291	1	1	135	141	53	43	9	9	40	31	0
	Belarus	566	582	122	118	33	37	0	0	66	44	2	1	53	73	4	5	3	2	9	10	133	138	1	0	119	127	10	12	11	14	1	1	0
	Bosnia and Herzegovina	655	693	224	238	39	49	0	0	21	23	32	27	18	21	0	0	3	4	22	22	163	168	1	1	106	112	18	18	7	9	1	1	0
	Bulgaria	739	764	197	201	131	140	0	0	20	18	2	1	56	55	3	3	4	3	23	24	166	169	1	0	104	110	21	22	10	13	2	3	0
	Chile	658	542	72	92	31	28	0	0	4	10	4	5	31	33	5	4	5	3	4	4	335	191	1	0	144	149	12	12	8	8	2	2	0
	Cook Islands	956	1,126	167	237	81	127	0	0	27	53	29	25	16	17	36	40	6	3	101	97	327	352	5	5	116	122	14	19	8	11	23	17	0
	Croatia	541	600	165	190	17	17	0	0	49	71	1	1	15	13	1	4	2	1	8	9	163	168	1	0	104	109	8	10	6	6	1	1	0
	Dominica	1,307	1,260	165	180	127	122	0	0	247	165	27	23	89	110	31	67	27	27	72	49	276	290	4	6	143	144	30	29	11	11	55	38	2
	Georgia	856	907	214	224	141	133	0	0	31	43	3	2	45	69	5	4	19	15	54	57	145	149	1	1	87	98	33	30	11	18	68	64	0
	Greece	548	428	57	69	30	30	0	0	2	2	1	2	13	15	1	2	1	1	8	13	284	135	1	0	138	145	7	9	4	5	1	1	0
	Greenland	753	675	69	110	32	21	0	0	2	3	48	42	28	46	14	11	3	4	16	13	370	252	1	1	148	149	15	17	6	6	1	1	0
	Hungary	600	621	234	238	30	28	0	0	15	23	1	1	13	13	1	1	1	1	14	17	162	167	1	0	104	108	16	17	5	6	1	1	0
	Israel	506	419	63	74	24	23	0	0	2	3	3	3	14	15	3	6	1	0	7	12	234	117	1	0	139	145	11	15	3	4	2	3	0
	Italy	537	413	49	62	12	10	0	0	6	6	2	2	14	16	4	3	1	0	4	6	283	135	1	0	147	154	7	9	5	6	3	3	0
	Jordan	782	771	241	240	97	76	0	0	19	24	47	41	53	57	2	2	3	1	34	32	151	155	1	2	98	109	20	18	11	13	4	3	0
	Kazakhstan	782	814	155	156	138	138	0	0	21	24	2	3	97	109	4	2	19	12	53	56	144	149	1	1	99	109	34	37	12	14	4	3	0
	Lebanon	851	894	265	291	55	60	0	0	15	18	80	58	31	33	12	12	6	4	21	22	153	157	1	1	124	135	71	80	14	15	4	8	0
	Libya	936	901	293	296	97	80	0	0	5	7	26	21	35	33	5	5	8	7	121	92	148	152	1	1	122	132	28	25	13	14	35	35	0
	Malaysia	1,336	1,477	266	311	123	132	2	2	54	78	35	32	70	86	106	101	25	19	165	193	292	310	1	2	130	141	30	33	8	10	30	27	0
	Malta	571	440	45	54	38	28	0	0	4	4	2	3	22	22	3	3	0	0	14	24	290	137	1	0	138	146	8	13	4	4	1	1	0
	Mauritius	1,057	1,328	116	136	64	64	0	0	63	226	3	2	51	61	5	21	9	8	267	297	288	307	1	1	130	141	26	24	8	10	27	28	0
	Montenegro	583	607	166	163	36	46	0	0	35	26	14	12	14	22	4	5	1	1	21	30	164	168	1	1	105	110	13	15	7	8	1	1	0
	Niue	1,456	1,700	212	292	230	370	0	0	29	45	53	43	88	85	35	38	32	16	176	184	333	355	8	7	120	125	20	28	12	23	107	90	0
	North Macedonia	734	748	229	206	67	78	0	0	50	46	36	32	27	30	1	1	4	3	22	33	164	168	1	1	106	111	17	22	8	10	2	6	0
	Northern Mariana Islands	1,142	1,284	174	258	80	167	0	0	8	21	56	56	57	46	17	20	19	7	155	136	334	353	11	5	115	120	17	24	13	24	84	48	0
	Oman	930	954	299	307	120	98	0	0	10	14	19	14	82	106	7	8	7	5	50	56	156	160	1	1	120	130	39	40	19	13	3	2	0
	Palau	1,735	1,881	332	443	469	552	0	0	29	49	44	40	99	14	34	37	28	9	155	141	327	352	5	5	118	125	18	24	15	46	63	44	0
	Poland	523	548	129	138	44	46	0	0	5	6	1	1	18	19	4	4	2	1	18	19	166	170	1	0	112	118	16	18	6	7	1	1	0
	Portugal	548	429	49	58	33	29	0	0	3	6	2	2	17	22	4	6	1	1	8	13	281	134	1	0	133	140	11	13	4	5	1	1	0
	Republic of Moldova	953	956	133	134	134	139	0	0	313	300	1	1	42	56	5	4	12	7	23	18	134	138	1	0	124	130	19	17	8	10	3	3	0
	Romania	807	814	218	206	178	200	0	0	19	8	2	2	44	44	16	8	14	11	19	23	166	170	1	0	103	108	19	25	7	9	1	1	0
	Russian Federation	683	677	158	146	77	75	0	0	60	40	3	2	41	48	37	42	7	4	9	11	144	148	1	0	128	138	8	11	9	9	2	2	0
	Saint Kitts and Nevis	1,076	1,090	178	203	118	108	0	0	66	54	4	7	96	66	96	101	11	12	62	56	260	284	2	1	133	145	29	35	10	10	12	8	1
	Saudi Arabia	700	680	239	224	67	46	0	0	20	24	11	10	24	17	13	13	12	11	23	27	151	154	1	1	120	129	8	10	8	10	4	3	0
	Serbia	558	596	157	186	27	30	0	0	18	16	16	13	21	21	6	8	2	1	18	18	164	167	1	1	105	111	15	15	8	9	1	1	0
	Seychelles	1,300	1,679	267	407	154	216	0	0	156	224	49	39	39	83	24	20	13	10	115	135	294	310	2	2	130	142	26	55	8	10	24	27	0
	Spain	556	447	55	68	15	14	0	0	7	7	3	2	21	22	4	6	1	0	4	16	295	145	1	0	138	145	8	17	4	4	1	1	0
	Sri Lanka	1,269	1,343	332	356	107	103	0	0	105	127	25	21	76	79	1	1	18	15	148	153	285	302	1	2	134	145	22	25	7	9	8	7	0
	Trinidad and Tobago	993	1,025	137	144	84	90	0	0	88	101	3	2	54	58	83	53	5	5	70	77	256	270	1	1	133	139	35	40	9	9	37	34	0
	Turkey	831	867	261	284	85	90	0	0	50	37	25	24	36	39	2	2	10	9	25	23	148	152	1	1	145	152	30	40	10	12	2	2	0
	Ukraine	645	687	129	139	76	88	0	0	42	46	4	3	68	72	20	16	10	6	9	12	133	138	1	0	130	140	10	12	10	14	3	2	0
	United States Virgin Islands	835	874	173	184	23	34	0	0	67	57	34	33	21	28	23	20	5	3	61	57	257	275	2	4	131	135	25	30	9	9	5	6	0
	Uruguay	661	557	50	78	40	38	0	0	11	20	2	2	39	32	6	9	2	2	12	13	334	192	1	1	143	148	12	13	7	7	1	2	0

5 to 14 years, DALYs/100,000, for males and females, 2019

		Total communicable		Enteric infections		Lower respiratory infections		Malaria		Neonatal sepsis and other neonatal infections		Vaccine Preventable disease		Meningitis & Encephalitis	
		Female	Male	Female	Male	Female	Male	Female	Male	Female	Male	Female	Male	Female	Male
	Andorra	531	384	53	63	14	8	0	0	3	3	3	3	13	8
	Australia	586	462	41	57	13	9	0	0	2	2	3	3	12	14
	Austria	531	397	56	65	12	7	0	0	3	3	2	2	13	14
	Belgium	562	434	73	85	16	15	0	0	5	3	3	4	17	19
	Bermuda	701	714	145	164	28	24	0	0	21	3	3	3	39	20
	Brunei Darussalam	829	727	9	12	161	152	0	0	6	7	10	8	30	42
	Canada	824	581	61	124	18	14	0	0	9	7	4	3	12	12
	Cyprus	539	409	69	81	9	8	0	0	2	2	4	4	7	9
	Czechia	551	578	180	190	31	38	0	0	25	24	2	1	14	14
	Denmark	581	408	68	80	11	8	0	0	2	2	2	2	14	14
	Estonia	515	482	161	132	54	32	0	0	12	15	1	1	15	21
	Finland	515	411	54	65	8	7	0	0	3	3	2	2	10	12
	France	520	395	46	58	10	9	0	0	4	5	3	3	12	14
	Germany	609	438	65	77	15	15	0	0	3	3	2	3	12	14
	Guam	1,272	1,208	214	271	154	96	0	0	29	67	67	58	88	43
	Iceland	563	416	67	75	27	17	0	0	3	2	2	2	21	19
	Ireland	519	389	46	55	13	12	0	0	3	3	2	2	15	16
	Japan	581	447	5	6	32	36	0	0	3	3	2	2	12	12
High SDI	Kuwait	708	748	215	240	120	115	0	0	5	6	1	1	18	23
	Latvia	523	535	143	158	48	32	0	0	16	17	2	2	24	29
	Lithuania	545	548	152	143	65	58	0	0	25	21	1	1	21	24
	Luxembourg	551	418	69	80	15	12	0	0	2	2	2	3	14	13
	Monaco	528	390	53	63	23	15	0	0	3	3	3	3	7	11
	Netherlands	494	397	45	55	16	13	0	0	9	13	3	3	21	18
	New Zealand	625	505	70	77	10	10	0	0	6	5	2	2	16	17
	Norway	549	383	57	68	10	7	0	0	3	3	2	2	14	15
	Puerto Rico	876	864	147	150	33	22	0	0	157	141	2	2	21	16
	Qatar	648	646	192	206	84	58	0	0	3	4	14	11	32	24
	Republic of Korea	586	456	9	12	22	22	0	0	4	7	7	6	16	21
	San Marino	562	415	52	63	10	11	0	0	3	3	8	6	25	21
	Singapore	624	495	5	7	63	69	0	0	8	8	2	2	17	23
	Slovakia	608	660	180	235	82	70	0	0	10	10	9	7	21	21
	Slovenia	525	533	162	171	15	14	0	0	34	23	1	1	12	11
	Sweden	576	408	58	71	11	9	0	0	4	4	2	4	11	9
	Switzerland	525	388	53	61	9	7	0	0	4	7	4	2	11	9
	Taiwan (Province of China)	686	730	104	123	51	55	0	0	8	6	2	2	24	30
	United Arab Emirates	733	779	263	262	36	66	0	0	4	5	19	18	26	35
	United Kingdom	552	427	51	63	21	19	0	0	3	3	4	4	20	22
	United States of America	580	581	74	81	26	26	0	0	6	8	3	3	12	13

	HIV/AIDS		Tuberculosis		Neglected Tropical diseases		Infectious skin conditions		Sexually transmitted infections excluding HIV		Upper respiratory infections		Other unspecified infectious diseases		Hepatitis		Rheumatic heart disease		Maternal sepsis and other maternal infections
	Female	Male	Female	Male	Female	Male	Female	Male	Female	Male	Female	Male	Female	Male	Female	Male	Female	Male	Female
Andorra	2	2	0	0	4	5	285	134	1	1	136	143	9	8	7	5	1	1	0
Australia	2	1	1	0	8	9	345	201	1	0	140	144	11	14	4	5	2	2	0
Austria	2	3	0	0	5	8	288	136	1	0	134	141	10	12	3	4	1	1	0
Belgium	5	5	1	0	3	6	288	136	1	0	137	143	10	13	4	4	1	1	0
Bermuda	24	41	9	4	23	29	254	260	1	1	131	136	14	16	8	8	3	3	0
Brunei Darussalam	6	7	23	10	55	86	339	198	1	1	137	142	37	44	9	13	5	4	0
Canada	4	2	0	0	3	4	549	250	1	0	151	151	10	10	3	4	1	1	0
Cyprus	0	0	0	0	4	7	290	137	1	1	138	145	9	9	4	4	1	2	0
Czechia	2	1	1	1	12	13	164	168	1	0	102	108	13	14	5	6	1	1	0
Denmark	1	1	1	0	5	6	330	139	1	0	135	142	8	10	2	3	1	0	0
Estonia	5	1	2	1	6	7	129	134	1	0	114	122	8	9	7	7	1	1	0
Finland	0	3	0	0	4	3	288	160	1	0	135	143	7	9	2	3	1	1	0
France	3	3	1	0	3	6	286	135	1	0	138	145	8	12	4	4	2	1	0
Germany	3	4	0	0	5	6	355	158	1	0	135	144	9	11	3	3	1	1	0
Guam	25	35	29	7	38	46	340	358	8	6	114	119	19	24	13	20	136	58	0
Iceland	2	2	1	0	4	5	289	136	0	0	137	145	8	9	2	2	1	1	0
Ireland	1	1	0	0	2	5	289	136	1	0	137	143	8	11	3	3	1	1	0
Japan	1	2	1	0	14	9	336	197	1	0	151	158	18	14	5	6	1	1	0
Kuwait	4	2	8	4	31	31	152	156	1	1	120	131	21	23	8	9	3	7	0
Latvia	10	3	3	2	11	12	131	137	1	0	116	123	10	12	7	8	1	1	0
Lithuania	1	8	4	3	9	10	134	138	1	0	115	123	9	10	7	9	1	1	0
Luxembourg	1	2	0	0	4	6	289	136	1	0	135	143	15	18	3	3	1	1	0
Monaco	2	1	1	1	4	5	289	136	1	1	133	140	6	7	3	4	1	1	0
Netherlands	3	2	0	0	4	2	243	134	1	0	136	143	9	11	3	3	1	1	0
New Zealand	2	1	1	0	6	12	342	201	1	0	151	155	9	16	4	4	5	3	0
Norway	3	2	0	0	3	7	296	105	1	0	149	157	7	13	3	4	1	1	0
Puerto Rico	16	10	2	2	83	91	252	259	1	1	133	138	19	23	8	8	2	2	0
Qatar	2	3	6	4	13	12	155	159	1	1	120	130	14	17	9	11	2	5	0
Republic of Korea	1	2	5	4	20	19	339	197	2	1	139	144	15	12	7	10	1	1	0
San Marino	2	1	1	0	15	8	284	135	1	1	139	143	18	15	5	6	1	1	0
Singapore	1	2	2	1	21	18	344	197	1	0	139	146	14	12	5	7	2	2	0
Slovakia	0	0	1	1	15	16	165	169	1	1	102	107	14	14	7	7	2	1	0
Slovenia	2	1	1	1	11	13	166	170	1	0	102	107	11	13	6	7	1	1	0
Sweden	2	2	0	0	4	6	323	135	1	0	148	155	10	11	2	2	1	1	0
Switzerland	2	2	0	0	3	5	290	136	1	0	136	144	8	10	3	3	1	1	0
Taiwan (Province of China)	2	3	13	10	47	45	308	314	1	1	99	106	19	18	8	15	2	2	0
United Arab Emirates	6	6	10	9	30	24	153	157	1	1	119	130	30	22	10	11	25	32	0
United Kingdom	3	2	1	1	10	10	273	127	1	0	148	155	13	15	4	4	1	1	0
United States of America	3	3	0	0	10	8	262	256	1	0	162	161	16	15	4	4	1	1	0

S13 (B) Percentage change in DALYs/100,000 for each communicable condition for each location for 5-14 year age group by sex. The colours on this heat map are green for values less than 0, the darker the green the greater the reduction in DALYs/100,000 between 1990 and 2019, the orange tones represent positive numbers, an increase in DALYs/ 100,000, with darker shades indicating worse outcome.

5 to 14

Low SDI	Total commuincable		Enteric infections		Lower respiratory infections		Malaria		Neonatal sepsis and other neonatal infections		Vaccine Preventable disease		Meningitis & Encephalitis		HIV/AIDS	
	Female	Male	Female	Male	Female	Male	Female	Male	Female	Male	Female	Male	Female	Male	Female	Male
Afghanistan	-2.2%	-2.2%	-0.6%	0.2%	-1.9%	-1.8%	-1.5%	-1.5%	43.2%	54.1%	-3.0%	-3.0%	-1.6%	-1.4%	14.8%	14.6%
Benin	-1.4%	-1.3%	-1.2%	-1.1%	-1.2%	-1.4%	1.0%	1.4%	41.0%	35.4%	-2.5%	-2.6%	-1.6%	-1.6%	14,739.4%	16,318.1%
Burkina Faso	-1.6%	-1.6%	-1.1%	-1.1%	-0.1%	-0.4%	-1.3%	-1.4%	44.7%	57.1%	-2.9%	-3.0%	-1.3%	-1.2%	-0.5%	-0.2%
Burundi	-1.8%	-1.8%	-1.7%	-1.4%	-1.1%	-1.6%	-1.9%	-2.0%	54.8%	65.0%	-3.1%	-3.1%	-1.3%	-1.5%	3.8%	4.7%
Central African Republic	-1.0%	-1.1%	-0.8%	-0.3%	-0.6%	-0.6%	0.7%	0.9%	22.3%	24.4%	-2.2%	-2.3%	-1.2%	-1.1%	816.4%	940.0%
Chad	-1.0%	-1.1%	-0.3%	-0.5%	0.1%	-0.3%	-0.3%	-0.1%	38.0%	44.3%	-2.5%	-2.5%	-0.0%	-0.2%	102.2%	106.2%
Côte d'Ivoire	-1.3%	-1.3%	-1.0%	-0.9%	-1.1%	-1.4%	-0.6%	-0.4%	22.5%	39.7%	-3.0%	-3.1%	-1.4%	-1.6%	42.2%	47.0%
Dem Rep of the Congo	-1.6%	-1.6%	-1.2%	-0.6%	-1.5%	-1.6%	-0.9%	-0.8%	40.4%	38.3%	-2.7%	-2.7%	-1.6%	-1.7%	2.2%	2.8%
Eritrea	-2.1%	-2.0%	-2.3%	-1.7%	-1.1%	-1.2%	-2.5%	-2.6%	63.5%	86.4%	-3.1%	-3.2%	-1.1%	-1.3%	42.3%	45.9%
Ethiopia	-2.4%	-2.4%	-2.6%	-2.1%	-2.3%	-2.4%	-0.1%	1.9%	83.5%	95.7%	-3.1%	-3.1%	-2.3%	-2.5%	26.0%	24.3%
Gambia	-1.9%	-1.9%	-1.6%	-1.3%	-1.3%	-1.5%	-2.5%	-2.5%	14.5%	28.0%	-3.1%	-3.1%	-1.8%	-1.8%	432.7%	489.4%
Guinea	-1.4%	-1.3%	-0.9%	-0.8%	-0.7%	-0.6%	0.1%	-0.0%	64.0%	61.4%	-2.8%	-2.8%	-0.9%	-0.9%	195.4%	231.1%
Guinea-Bissau	-1.9%	-1.9%	-1.9%	-1.6%	-1.8%	-1.9%	-2.3%	-2.2%	63.0%	117.8%	-2.5%	-2.5%	-1.8%	-2.0%	186.5%	198.6%
Haiti	-1.2%	-1.2%	-1.9%	-0.9%	-1.5%	-1.6%	-1.6%	-1.2%	46.9%	66.3%	-2.3%	-2.5%	-1.9%	-2.0%	67.7%	68.1%
Liberia	-1.5%	-1.6%	-0.9%	-0.8%	-1.6%	-1.9%	0.6%	0.2%	93.9%	116.0%	-3.0%	-3.1%	-1.9%	-2.1%	258.6%	296.1%
Madagascar	-2.0%	-2.0%	-1.7%	-1.0%	-2.3%	-2.5%	-1.5%	-2.0%	37.8%	54.3%	-2.6%	-2.7%	-2.3%	-2.5%	155,892.5	169,917.5
Malawi	-1.8%	-1.7%	-1.9%	-1.6%	-2.2%	-2.0%	-2.1%	-2.2%	67.7%	74.9%	-3.1%	-3.1%	-2.2%	-2.1%	22.7%	28.4%
Mali	-1.9%	-1.7%	-1.2%	-0.2%	-1.3%	-1.3%	-2.0%	-1.8%	88.3%	70.6%	-2.9%	-2.9%	-1.7%	-1.6%	128.2%	138.3%
Mozambique	-1.0%	-1.1%	-2.0%	-0.9%	-1.8%	-1.5%	-1.3%	-1.5%	62.6%	65.5%	-3.2%	-3.2%	-2.1%	-1.9%	497.1%	497.6%
Nepal	-2.4%	-2.3%	-2.2%	-2.0%	-2.5%	-2.5%	-3.2%	-3.2%	118.8%	98.9%	-3.0%	-3.1%	-1.7%	-1.5%	8.1e+14%	7.0e+14%
Niger	-1.6%	-1.7%	-1.3%	-0.9%	-1.5%	-1.8%	-1.1%	-0.9%	78.7%	83.8%	-2.2%	-2.4%	-1.7%	-2.0%	77.8%	77.7%
Pakistan	-1.4%	-1.2%	-0.2%	-0.6%	0.7%	1.0%	-2.2%	-2.2%	21.1%	17.9%	-3.1%	-3.1%	-0.1%	0.2%	61.6%	59.8%
Papua New Guinea	-1.4%	-1.5%	-1.6%	-1.5%	-1.2%	-1.1%	-1.7%	-1.9%	9.3%	12.3%	-2.3%	-2.4%	-1.7%	-1.6%	50,880.9%	63,630.0%
Rwanda	-2.4%	-2.4%	-2.6%	-2.3%	-2.1%	-2.3%	-2.7%	-2.8%	74.4%	87.8%	-3.1%	-3.1%	-2.2%	-2.3%	10.1%	12.6%
Senegal	-1.8%	-1.7%	-1.7%	-1.5%	-1.6%	-1.8%	-1.6%	-1.8%	28.9%	37.6%	-2.8%	-2.8%	-1.6%	-1.7%	102.8%	116.1%
Sierra Leone	-1.1%	-1.3%	-1.0%	-0.4%	-0.5%	-1.2%	-0.7%	-0.7%	43.5%	46.7%	-2.9%	-3.0%	-1.2%	-1.6%	361.6%	339.3%
Solomon Islands	-1.5%	-1.6%	-1.5%	-1.4%	-1.4%	-1.7%	-2.3%	-2.1%	2.7%	5.7%	-2.9%	-2.9%	-1.6%	-1.6%	508.0%	545.3%
Somalia	-1.5%	-1.7%	-0.7%	0.1%	0.5%	1.0%	-1.5%	-0.9%	26.8%	28.0%	-2.0%	-1.7%	1.2%	1.9%	2,644.8%	2,627.3%
South Sudan	-1.4%	-1.5%	-0.0%	0.6%	-0.6%	-0.1%	-0.3%	0.3%	38.8%	41.4%	-2.5%	-2.4%	0.1%	0.6%	340.4%	379.0%
Togo	-1.3%	-1.2%	-0.9%	-0.9%	-1.6%	-1.5%	-0.6%	-0.6%	30.3%	34.5%	-3.0%	-3.0%	-1.8%	-1.7%	165.1%	194.3%
Uganda	-1.8%	-1.8%	-1.0%	-0.7%	-0.3%	-0.7%	-1.7%	-1.6%	45.2%	50.3%	-2.4%	-2.6%	-0.9%	-1.1%	-1.6%	-1.3%
United Republic of Tanzania	-1.7%	-1.8%	-1.6%	-1.5%	-1.6%	-1.8%	-2.2%	-2.2%	40.8%	41.7%	-2.8%	-2.9%	-1.2%	-1.3%	10.9%	13.0%
Yemen	-2.3%	-2.3%	-0.7%	-0.2%	-1.5%	-1.3%	-3.1%	-3.1%	22.6%	28.3%	-3.1%	-3.1%	-1.5%	-1.3%	14.0%	14.0%

Low SDI	Tuberculosis		Neglected Tropical diseases		Infectious skin conditions		Sexually transmitted infections excluding HIV		Upper respiratory infections		Other unspecified infectious diseases		Hepatitis		Rheumatic heart disease		Maternal sepsis and other maternal infections
	Female	Male	Female	Male	Female	Male	Female	Male	Female	Male	Female	Male	Female	Male	Female	Male	Female
Afghanistan	-2.5%	-2.6%	-1.4%	-1.9%	0.0%	0.0%	0.0%	-0.4%	-0.1%	-0.1%	-1.2%	-1.3%	-2.3%	-2.5%	-2.0%	-1.8%	-2.4%
Benin	-2.0%	-2.3%	-2.3%	-2.2%	-0.1%	-0.2%	-1.7%	-1.4%	-0.1%	-0.2%	0.5%	1.3%	-1.1%	-2.3%	-0.9%	-0.9%	-2.0%
Burkina Faso	-1.4%	-1.7%	-2.4%	-2.5%	-0.1%	-0.1%	-1.1%	-0.7%	-0.1%	-0.2%	2.9%	1.5%	-1.0%	-2.1%	-0.0%	-0.3%	-1.2%
Burundi	-1.8%	-2.2%	-2.1%	-2.0%	-0.1%	-0.2%	-1.1%	-2.2%	-0.5%	-0.6%	-1.1%	-1.1%	-1.0%	-1.5%	-1.3%	-1.5%	0.7%
Central African Republic	-0.4%	-0.5%	-2.6%	-2.7%	-0.1%	-0.0%	-0.7%	-1.3%	-0.4%	-0.3%	0.3%	0.3%	-0.8%	-1.7%	-0.5%	-0.6%	0.4%
Chad	-0.7%	-0.9%	-1.2%	-1.3%	-0.0%	-0.1%	-0.7%	-0.7%	-0.0%	-0.2%	0.2%	0.2%	0.3%	-1.0%	-0.5%	-0.6%	-1.2%
Côte d'Ivoire	-1.5%	-2.1%	-2.6%	-2.5%	-0.0%	-0.0%	-1.6%	-1.6%	-0.0%	-0.2%	0.1%	0.6%	-1.0%	-2.1%	-0.9%	-0.8%	-1.7%
Dem Rep of the Congo	-1.7%	-2.3%	-2.2%	-2.4%	-0.2%	-0.2%	-1.8%	-2.1%	-0.3%	-0.3%	-0.2%	-0.3%	-1.0%	-2.0%	-1.0%	-0.9%	1.5%
Eritrea	-2.0%	-2.1%	-2.5%	-2.8%	-0.2%	-0.2%	-0.8%	-2.3%	-0.4%	-0.5%	-1.3%	-1.6%	-1.1%	-1.6%	-1.0%	-1.2%	-1.5%
Ethiopia	-2.7%	-2.8%	-2.6%	-2.8%	-0.3%	-0.3%	-2.2%	-2.5%	-1.0%	-1.1%	-0.8%	-0.7%	-1.9%	-1.9%	-1.6%	-1.9%	-2.4%
Gambia	-1.9%	-2.0%	-1.7%	-1.8%	-0.1%	-0.1%	-1.4%	-1.2%	-0.0%	-0.2%	-0.2%	-0.1%	-0.9%	-1.0%	-0.8%	-0.7%	-1.9%
Guinea	-1.7%	-1.8%	-1.1%	-1.4%	-0.1%	-0.1%	-1.7%	-1.5%	-0.0%	-0.2%	0.9%	1.1%	-0.7%	-1.3%	-1.0%	-0.7%	-1.7%
Guinea-Bissau	-2.3%	-2.5%	-1.5%	-1.9%	-0.1%	-0.1%	-2.1%	-2.1%	-0.0%	-0.4%	0.7%	0.6%	-1.5%	-2.4%	-1.4%	-1.5%	-2.4%
Haiti	-2.0%	-2.8%	-1.0%	-2.3%	-0.0%	0.0%	-0.6%	-1.5%	-0.4%	-0.4%	0.3%	0.1%	-2.0%	-1.8%	-1.6%	-1.6%	-0.6%
Liberia	-2.3%	-2.7%	-1.4%	-1.8%	-0.2%	-0.3%	-0.7%	-0.6%	0.0%	-0.1%	-0.8%	-0.6%	-1.2%	-2.2%	-1.2%	-0.9%	-1.8%
Madagascar	-2.5%	-2.9%	-1.7%	-2.3%	-0.1%	-0.1%	-1.4%	-2.0%	-0.7%	-0.8%	-1.5%	-1.9%	-1.9%	-2.5%	-2.0%	-2.0%	-1.9%
Malawi	-2.3%	-2.5%	-2.6%	-2.7%	-0.1%	-0.1%	-0.9%	-1.4%	-0.7%	-0.6%	-0.5%	-0.4%	-1.9%	-2.4%	-1.5%	-1.3%	-1.6%
Mali	-1.0%	-1.6%	-1.7%	-2.3%	-0.1%	-0.1%	-1.1%	-0.8%	-0.1%	-0.3%	0.5%	1.1%	-1.6%	-2.6%	-1.2%	-0.9%	-2.1%
Mozambique	-2.3%	-2.7%	-1.9%	-2.4%	-0.1%	-0.1%	-2.4%	-2.5%	-0.9%	-0.8%	-0.2%	-0.2%	-1.8%	-2.3%	-1.2%	-0.9%	-2.6%
Nepal	-2.9%	-3.0%	-2.4%	-2.7%	-0.1%	-0.1%	-1.5%	-1.8%	-0.5%	-0.4%	0.4%	0.0%	-2.6%	-2.2%	-2.1%	-1.5%	-3.2%
Niger	-2.2%	-2.5%	-1.3%	-2.0%	-0.2%	-0.3%	-2.1%	-1.9%	0.2%	-0.1%	-0.2%	-0.4%	-1.3%	-2.1%	-1.2%	-1.2%	-1.2%
Pakistan	-1.3%	-0.9%	-1.7%	-1.8%	-0.0%	-0.0%	0.6%	0.4%	-0.2%	-0.1%	0.2%	0.1%	-1.4%	-0.9%	0.5%	1.5%	-2.2%
Papua New Guinea	-1.9%	-1.8%	-1.3%	-1.8%	0.0%	0.0%	-0.4%	-0.3%	-0.1%	-0.1%	-0.2%	0.0%	-1.9%	-2.3%	-0.2%	-0.2%	-2.2%
Rwanda	-2.8%	-2.8%	-2.2%	-2.3%	-0.4%	-0.4%	-1.6%	-2.2%	-0.5%	-0.6%	-1.2%	-2.2%	-2.0%	-2.5%	-1.9%	-2.1%	-2.5%
Senegal	-2.2%	-2.5%	-1.6%	-1.7%	-0.1%	-0.2%	-2.0%	-1.9%	0.0%	-0.2%	-0.5%	-0.3%	-1.7%	-2.1%	-1.0%	-0.9%	-2.1%
Sierra Leone	-1.6%	-2.4%	-2.0%	-2.3%	-0.2%	-0.2%	-1.5%	-1.6%	-0.0%	-0.2%	0.4%	0.2%	-0.8%	-2.3%	-0.8%	-1.0%	-0.9%
Solomon Islands	-1.6%	-1.7%	-0.5%	-0.7%	-0.0%	-0.0%	0.8%	1.2%	-0.0%	-0.0%	-0.5%	-0.3%	-1.7%	-2.5%	-1.0%	-1.0%	-0.8%
Somalia	0.2%	0.0%	-3.0%	-3.0%	-0.0%	-0.1%	1.2%	0.1%	0.1%	0.4%	-0.9%	-1.0%	0.4%	1.0%	-0.1%	0.2%	2.3%
South Sudan	-0.9%	-0.9%	-2.3%	-2.4%	-0.0%	-0.0%	0.9%	0.3%	-0.2%	-0.1%	-0.7%	-0.9%	0.4%	0.9%	-0.4%	-0.1%	0.3%
Togo	-1.9%	-2.1%	-1.7%	-2.0%	-0.1%	-0.1%	-2.1%	-1.9%	-0.0%	-0.2%	0.6%	0.5%	-1.4%	-2.3%	-1.2%	-0.9%	-2.1%
Uganda	-1.5%	-2.2%	-3.0%	-3.0%	-0.0%	-0.1%	2.9%	1.2%	-0.3%	-0.4%	-0.8%	-1.4%	-0.5%	-0.9%	-0.1%	-0.5%	-1.0%
United Republic of Tanzania	-1.9%	-2.4%	-2.3%	-2.4%	-0.3%	-0.3%	-1.9%	-2.1%	-0.3%	-0.3%	-0.3%	-0.6%	-1.4%	-1.8%	-1.0%	-1.1%	-1.5%
Yemen	-2.5%	-2.6%	-2.0%	-2.4%	-0.1%	-0.1%	-0.2%	-0.2%	-0.2%	-0.3%	1.9%	1.6%	-2.5%	-2.7%	-1.6%	-1.4%	-2.1%

5 to 14

		Total communicable		Enteric infections		Lower respiratory infections		Malaria		Neonatal sepsis and other neonatal infections		Vaccine Preventable disease		Meningitis & Encephalitis		HIV/AIDS		Tuberculosis		Neglected Tropical diseases		Infectious skin conditions		Sexually transmitted infections excluding HIV		Upper respiratory infections		Other unspecified infectious diseases		Hepatitis		Rheumatic heart disease		Maternal sepsis and other maternal infections
		Female	Male	Female	Male	Female	Male	Female	Male	Female	Male	Female	Male	Female	Male	Female	Male	Female	Male	Female	Male	Female	Male	Female	Male	Female	Male	Female	Male	Female	Male	Female	Male	Female
Low-middle SDI	Angola	-2.0%	-2.1%	-2.3%	-1.7%	-1.9%	-1.9%	-0.1%	0.1%	74.8%	102.3%	-2.8%	-2.9%	-2.0%	-2.1%	520.6%	554.9%	-2.5%	-2.7%	-2.9%	-3.0%	-0.3%	-0.3%	-1.5%	-1.9%	-0.5%	-0.6%	-0.2%	-0.8%	-1.6%	-2.6%	-1.3%	-1.4%	-1.8%
	Bangladesh	-2.5%	-2.5%	-2.0%	-1.9%	-2.1%	-2.1%	-3.3%	-3.3%	124.4%	207.6%	-3.1%	-3.0%	-1.2%	-0.4%			-2.8%	-2.9%	-2.3%	-2.5%	-0.1%	-0.1%	-1.4%	-2.1%	-0.5%	-0.3%	-1.5%	-1.2%	-2.6%	-2.7%	-0.9%	-1.1%	-3.1%
	Belize	-0.9%	-1.2%	-0.4%	-0.8%	-1.5%	-1.5%	-3.3%	-3.3%	5.8%	7.2%	-2.4%	-2.6%	-2.2%	-2.4%	-1.7%	-2.2%	-2.7%	-2.7%	-0.9%	-0.7%	-0.0%	-0.1%	0.2%	-0.2%	-0.2%	-0.3%	-0.7%	-0.5%	-2.5%	-2.0%	-1.4%	-1.2%	-2.0%
	Bhutan	-2.5%	-2.5%	-1.6%	-1.4%	-1.5%	-1.0%	-3.3%	-3.3%	128.9%	103.7%	-3.3%	-3.3%	-1.4%	-1.1%	226.6%	209.6%	-2.7%	-2.5%	-1.8%	-2.0%	-0.1%	-0.1%	1.1%	1.8%	-0.3%	-0.2%	-0.0%	0.2%	-2.0%	-2.1%	-1.2%	-0.3%	-2.5%
	Bolivia (Plurinational State of)	-2.2%	-2.0%	-2.2%	-1.7%	-2.3%	-2.2%	-3.3%	-3.3%	22.5%	18.3%	-2.9%	-2.9%	-2.3%	-2.2%	41.3%	38.2%	-3.0%	-3.0%	-2.2%	-2.1%	-0.1%	-0.0%	-1.8%	-2.1%	-0.2%	-0.2%	-1.4%	-1.0%	-2.2%	-2.2%	-1.7%	-1.6%	-2.9%
	Cabo Verde	-2.1%	-1.8%	-2.2%	-2.1%	-2.1%	-1.2%	0.4%	0.2%	16.9%	15.0%	-3.3%	-3.3%	-2.0%	-1.3%	21.7%	30.5%	-2.8%	-2.6%	-1.6%	-1.2%	-0.2%	-0.3%	-1.1%	-0.9%	-0.2%	-0.2%	-0.7%	-0.9%	-1.9%	-2.2%	-1.7%	-1.1%	-2.9%
	Cambodia	-2.5%	-2.4%	-2.5%	-2.2%	-2.5%	-2.4%	-3.1%	-3.1%	48.1%	47.4%	-3.2%	-3.2%	-1.9%	-1.7%	24,979.2%	26,274.2%	-2.8%	-2.7%	-2.5%	-2.6%	-0.1%	-0.1%	-1.3%	-1.8%	-0.5%	-0.4%	-1.1%	-0.8%	-2.1%	-1.8%	-2.2%	-2.0%	-3.0%
	Cameroon	-1.4%	-1.4%	-1.0%	-0.9%	-0.6%	-0.7%	0.7%	0.5%	29.0%	43.6%	-2.9%	-2.9%	-1.4%	-1.4%	311.9%	361.1%	-1.6%	-1.9%	-2.4%	-2.4%	-0.2%	-0.2%	-1.9%	-1.9%	0.0%	-0.1%	-1.0%	-1.0%	-0.8%	-2.2%	-0.7%	-0.5%	-1.8%
	Comoros	-1.9%	-1.9%	-1.7%	-1.3%	-1.0%	-1.4%	-2.7%	-2.8%	34.7%	43.1%	-2.3%	-2.5%	-0.8%	-1.1%	423.6%	502.4%	-1.9%	-2.5%	-1.3%	-1.8%	-0.2%	-0.2%	-1.5%	-2.1%	-0.3%	-0.4%	-1.0%	-1.3%	-0.9%	-1.2%	-1.2%	-1.3%	-1.5%
	Congo	-1.5%	-1.7%	-1.8%	-1.5%	-1.4%	-1.6%	-0.3%	-1.0%	18.1%	30.8%	-2.0%	-2.2%	-1.6%	-1.9%	2.0%	3.3%	-2.0%	-2.3%	-2.9%	-3.0%	-0.2%	-0.2%	-1.3%	-1.7%	-0.2%	-0.3%	-0.0%	-0.5%	-1.3%	-2.0%	-0.7%	-1.1%	-1.7%
	Democratic People's Republic of Kor..	-1.9%	-1.7%	0.3%	0.4%	-2.5%	-2.4%	194.2%	207.0%	9.2%	12.7%	-3.2%	-3.2%	-1.9%	-1.7%	688.4%	638.9%	-2.0%	-1.9%	-1.7%	-1.8%	-0.1%	-0.1%	-1.0%	-0.9%	-0.2%	-0.2%	-0.9%	-1.0%	-1.9%	-2.0%	-1.3%	-0.9%	-2.1%
	Djibouti	-1.5%	-1.6%	-1.5%	-1.2%	-1.0%	-0.7%	-0.9%	-0.8%	19.6%	28.9%	-2.6%	-2.6%	-0.8%	-0.6%	72,533.5%	85,676.9%	-1.9%	-2.1%	-2.8%	-2.9%	-0.1%	-0.1%	-0.2%	-1.3%	-0.3%	-0.2%	-1.1%	-1.2%	-1.0%	-0.9%	-1.0%	-0.9%	-0.4%
	Dominican Republic	-0.8%	-1.0%	-0.4%	-1.0%	-1.5%	-1.3%	-2.9%	-2.9%	12.4%	16.2%	-2.0%	-2.2%	-1.9%	-1.9%	347.3%	347.0%	-2.6%	-2.5%	-0.9%	-1.5%	-0.0%	-0.1%	-0.8%	-1.0%	0.0%	-0.1%	-1.4%	-1.3%	-1.8%	-1.7%	-1.4%	-1.4%	-1.9%
	El Salvador	-1.6%	-1.5%	-1.9%	-2.0%	-2.1%	-1.8%	-3.2%	-3.2%	-0.1%	-0.2%	-2.8%	-2.8%	-2.7%	-2.6%	75.3%	66.6%	-3.1%	-3.1%	-1.4%	-1.5%	-0.0%	-0.0%	-1.2%	-1.1%	-0.2%	-0.1%	-1.2%	-0.9%	-0.7%	-1.1%	-1.2%	-0.8%	-3.2%
	Eswatini	0.0%	-0.0%	-1.1%	-1.1%	-0.9%	-0.1%	-2.8%	-2.9%	8.2%	9.0%	-3.1%	-3.1%	-1.2%	-0.7%	195,694.5	206,193.7	-1.2%	-0.8%	-2.3%	-2.0%	-0.0%	-0.0%	-1.9%	-2.0%	-0.2%	-0.3%	-0.3%	-0.3%	-1.2%	-1.4%	-1.1%	-0.9%	-2.0%
	Ghana	-1.7%	-1.6%	-1.7%	-1.8%	-1.6%	-1.6%	-0.8%	-0.7%	34.2%	57.6%	-3.1%	-3.1%	-1.9%	-1.6%	40.7%	43.6%	-2.4%	-2.4%	-2.1%	-2.0%	-0.2%	-0.2%	-1.1%	-1.6%	-0.0%	-0.1%	0.9%	2.6%	-1.9%	-2.1%	-0.6%	-0.4%	-2.4%
	Guatemala	-2.6%	-2.6%	-2.9%	-2.8%	-2.7%	-2.7%	-3.1%	-3.2%	4.9%	6.8%	-3.2%	-3.2%	-2.1%	-2.2%	-1.0%	1.6%	-3.2%	-3.2%	-2.7%	-2.7%	-0.1%	-0.1%	-1.0%	-2.6%	-0.5%	-0.3%	-0.8%	-0.8%	-1.6%	-1.5%	-1.1%	-0.9%	-3.1%
	Honduras	-2.1%	-1.8%	-2.6%	-2.3%	-3.2%	-2.5%	-3.2%	-3.2%	9.4%	8.7%	-2.9%	-2.8%	-2.4%	-2.2%	-1.6%	-1.6%	-3.0%	-2.8%	0.2%	-0.4%	-0.1%	-0.1%	-2.3%	-1.2%	-0.6%	-0.4%	-1.9%	-2.1%	-2.6%	-2.2%	-0.1%	0.0%	-3.2%
	India	-2.4%	-2.2%	-2.3%	-2.2%	-2.4%	-2.2%	-2.9%	-2.8%	31.6%	30.5%	-3.1%	-3.1%	-2.3%	-2.2%	6,257.7%	7,160.1%	-2.8%	-2.7%	-2.0%	-2.3%	-0.2%	-0.2%	-1.8%	-1.7%	-0.4%	-0.3%	-1.0%	-1.0%	-2.3%	-1.9%	-1.9%	-1.6%	-3.1%
	Kenya	-1.1%	-1.1%	-1.4%	-1.3%	-1.2%	-0.8%	-1.7%	-1.7%	9.5%	9.0%	-2.8%	-2.8%	-1.5%	-1.3%	32.9%	36.7%	-1.8%	-1.6%	-1.6%	-2.0%	-0.1%	-0.1%	-1.7%	-1.7%	-0.1%	-0.2%	-0.4%	-0.3%	-1.2%	-0.9%	-0.3%	-0.2%	-2.3%
	Kiribati	-1.4%	-1.7%	-1.8%	-1.5%	-1.4%	-1.3%			8.1%	7.6%	-2.0%	-2.1%	-1.6%	-1.7%	362.9%	376.9%	-1.7%	-1.9%	-0.4%	-2.1%	-0.1%	-0.0%	-0.9%	-0.3%	0.0%	-0.0%	-0.5%	-0.5%	-1.7%	-2.6%	-1.0%	-1.2%	-1.2%
	Kyrgyzstan	-1.8%	-1.8%	-0.3%	-0.5%	-2.6%	-2.5%	-3.3%	-3.3%	2.0%	3.9%	-0.9%	-1.0%	-2.2%	-2.1%	7.2%	5.5%	-2.0%	-2.1%	-0.8%	-1.0%	0.0%	-0.0%	-0.1%	-1.2%	-0.4%	-0.3%	-0.8%	-0.9%	-2.8%	-2.7%	-1.6%	-1.5%	-0.4%
	Lao People's Democratic Republic	-2.5%	-2.5%	-2.6%	-2.1%	-2.6%	-2.6%	-2.7%	-2.7%	66.1%	76.1%	-3.0%	-3.1%	-2.4%	-2.3%	5.8e+14%	4.1e+14%	-2.8%	-2.8%	-1.9%	-2.2%	-0.1%	-0.1%	-1.1%	-1.7%	-0.5%	-0.4%	-1.3%	-1.5%	-3.0%	-3.1%	-1.5%	-1.4%	-3.2%
	Lesotho	0.8%	0.6%	-0.9%	-1.0%	-0.7%	0.4%			8.7%	10.1%	-3.0%	-3.0%	-1.2%	-0.5%	639.2%	686.8%	-1.0%	-0.3%	-2.6%	-2.5%	-0.0%	0.0%	-1.3%	-1.1%	-0.3%	-0.3%	-0.0%	0.2%	-1.4%	-2.1%	-0.9%	-0.7%	-2.3%
	Maldives	-2.4%	-2.1%	-2.3%	-2.0%	-2.6%	-2.1%			16.6%	12.7%	-3.2%	-3.2%	-2.2%	-2.0%	23.9%	21.7%	-3.1%	-3.1%	-2.1%	-1.7%	-0.1%	-0.1%	-1.4%	-1.8%	-0.2%	-0.3%	-2.2%	-2.1%	-3.0%	-1.8%	-2.8%	-2.3%	-3.1%
	Marshall Islands	-1.1%	-1.0%	-1.5%	-1.4%	-0.9%	-0.6%			1.3%	1.8%	-2.0%	-2.1%	-1.4%	-1.3%	415.0%	450.1%	-1.8%	-1.7%	-1.0%	-1.2%	-0.0%	-0.0%	-0.0%	0.3%	-0.1%	-0.1%	-1.3%	-0.5%	-2.1%	-2.1%	0.2%	0.0%	-1.4%
	Mauritania	-1.8%	-1.7%	-1.9%	-1.5%	-1.6%	-1.9%	13.2%	16.9%	36.7%	50.8%	-3.1%	-3.1%	-1.9%	-2.1%	-0.8%	-0.1%	-2.3%	-2.7%	-1.4%	-1.7%	-0.3%	-0.3%	-1.9%	-1.9%	-0.1%	-0.2%	-0.8%	-0.6%	-1.5%	-2.3%	-1.2%	-0.9%	-2.8%
	Micronesia (Federated States of)	-1.4%	-1.6%	-1.9%	-1.9%	-1.8%	-1.9%			2.3%	3.8%	-2.7%	-2.8%	-2.0%	-2.1%	2,334.7%	2,731.1%	-2.5%	-2.4%	-1.8%	-2.4%	-0.1%	-0.1%	-1.1%	-1.1%	-0.1%	-0.1%	-1.4%	-1.0%	-2.3%	-2.4%	-0.9%	-1.4%	-2.0%
	Mongolia	-2.3%	-2.0%	0.1%	0.1%	-2.6%	-2.3%			9.4%	10.2%	-2.7%	-2.6%	-2.9%	-2.6%	24,393.6%	24,499.7%	-2.7%	-2.4%	-1.8%	-1.7%	0.1%	0.0%	1.8%	1.7%	-0.2%	-0.2%	-1.5%	-1.8%	-3.0%	-2.8%	-1.0%	-1.1%	-2.7%
	Morocco	-2.2%	-2.0%	-1.2%	-0.6%	-2.0%	-1.9%	-3.3%	-3.3%	15.5%	23.8%	-3.0%	-3.0%	-2.0%	-2.0%	82.7%	79.4%	-2.7%	-2.8%	-1.6%	-1.6%	-0.0%	-0.1%	-1.1%	-1.1%	-0.1%	-0.1%	-1.3%	-1.1%	-2.2%	-2.3%	-2.1%	-2.0%	-3.0%
	Myanmar	-2.6%	-2.5%	-2.2%	-2.2%	-2.9%	-3.0%	-3.2%	-3.2%	43.3%	55.7%	-3.1%	-3.1%	-2.8%	-2.3%	30,946.9%	34,349.5%	-2.8%	-2.7%	-2.7%	-2.5%	-0.1%	-0.1%	-0.5%	-1.1%	-0.5%	-0.5%	-1.4%	-1.4%	-2.3%	-2.7%	-1.9%	-2.0%	-2.5%
	Nicaragua	-1.6%	-1.6%	-1.4%	-1.6%	-2.1%	-1.9%	-3.1%	-3.0%	-1.3%	-0.8%	-3.2%	-3.2%	-2.4%	-2.4%	81.5%	81.8%	-3.0%	-3.1%	-1.5%	-1.7%	-0.0%	-0.0%	-1.6%	-1.3%	-0.1%	-0.1%	-1.9%	-1.5%	-1.5%	-1.4%	-1.6%	-1.2%	-3.0%
	Nigeria	-1.6%	-1.6%	-1.1%	-0.9%	-2.4%	-2.2%	-0.8%	-1.0%	22.9%	35.3%	-2.9%	-2.9%	-1.4%	-1.3%	167.1%	179.7%	-2.3%	-2.2%	-1.8%	-2.2%	0.1%	0.2%	-1.3%	-0.6%	-0.1%	-0.1%	-0.2%	-0.2%	-1.8%	-2.2%	-1.2%	-1.1%	-1.7%
	Palestine	-2.0%	-1.9%	-0.9%	-0.8%	-1.8%	-1.8%			3.5%	6.9%	-3.3%	-3.3%	-2.3%	-2.2%	125.5%	102.5%	-2.7%	-2.7%	-1.9%	-2.4%	-0.1%	-0.1%	-0.7%	-0.9%	-0.1%	-0.2%	-1.0%	-0.8%	-2.4%	-1.6%	-1.1%	-1.0%	-3.1%
	Sao Tome and Principe	-2.2%	-2.6%	-2.0%	-2.7%	-1.7%	-2.6%	-2.9%	-3.0%	14.2%	24.8%	-3.2%	-3.2%	-1.5%	-2.4%	6.0%	11.4%	-2.5%	-2.6%	-1.7%	-2.1%	-0.2%	-0.3%	-1.7%	-1.6%	-0.1%	-0.4%	-0.8%	-1.4%	-1.9%	-3.0%	-1.0%	-1.7%	-2.8%
	Sudan	-2.4%	-2.4%	-0.3%	0.7%	-1.5%	-1.3%	-1.2%	-1.4%	34.8%	41.4%	-3.2%	-3.2%	-2.2%	-2.2%	186.6%	180.0%	-2.6%	-2.7%	-2.4%	-2.5%	-0.0%	-0.0%	0.5%	0.1%	-0.2%	-0.2%	-0.6%	-0.5%	-1.9%	-2.6%	-1.8%	-1.5%	-2.4%
	Tajikistan	-1.7%	-1.6%	-0.4%	-0.5%	-2.2%	-1.8%	-3.2%	-3.2%	5.7%	8.0%	-2.1%	-2.1%	-2.5%	-2.6%	26.4%	52.4%	-1.4%	-1.0%	-0.9%	-1.0%	-0.0%	-0.0%	-1.3%	-0.0%	-0.1%	-0.2%	-0.5%	-0.5%	-2.5%	-2.5%	-1.3%	-1.3%	-1.3%
	Timor-Leste	-2.4%	-2.3%	-2.1%	-1.5%	-2.2%	-1.8%	-3.3%	-3.3%	39.2%	24.4%	-3.1%	-3.1%	-1.8%	-1.5%	4,003.8%	4,187.5%	-2.5%	-2.3%	-1.6%	-2.3%	-0.1%	-0.1%	-0.8%	-1.1%	-0.4%	-0.3%	-0.8%	-0.6%	-2.7%	-2.5%	-0.7%	-0.2%	-2.8%
	Tuvalu	-2.0%	-1.9%	-2.0%	-1.6%	-2.5%	-2.4%			13.5%	13.7%	-2.6%	-2.7%	-2.5%	-2.5%	486.7%	517.3%	-2.9%	-2.8%	-0.3%	-0.5%	-0.1%	-0.1%	-1.7%	-1.6%	-0.1%	-0.2%	-1.2%	-1.0%	-2.6%	-2.8%	-1.9%	-2.1%	-2.7%
	Vanuatu	-1.3%	-1.4%	-0.9%	-1.2%	-0.4%	-0.5%	-3.3%	-3.3%	2.0%	2.6%	-2.2%	-2.3%	-0.8%	-1.1%	466.7%	501.1%	-1.3%	-1.2%	1.3%	-2.0%	-0.0%	-0.0%	0.8%	0.8%	-0.0%	-0.1%	0.7%	0.7%	-1.6%	-2.5%	0.8%	0.2%	-0.4%
	Venezuela (Bolivarian Republic of)	-0.5%	-0.6%	-0.5%	-0.4%	-1.6%	-1.4%	6.4%	4.2%	2.6%	3.0%	-2.4%	-2.0%	-2.0%	-2.0%	2.1%	-0.4%	-2.5%	-2.7%	-1.4%	-1.3%	-0.1%	-0.1%	0.1%	-0.9%	-0.1%	-0.1%	-0.8%	-0.4%	-2.1%	-2.2%	-2.6%	-2.5%	-2.2%
	Zambia	-1.7%	-1.7%	-1.9%	-0.9%	-2.0%	-1.7%	-1.9%	-1.8%	49.6%	68.7%	-3.1%	-3.1%	-2.5%	-2.3%	22.9%	25.6%	-2.4%	-2.7%	-2.7%	-2.9%	-0.1%	-0.2%	-1.3%	-1.3%	-0.6%	-0.5%	-0.5%	-0.5%	-1.8%	-1.8%	-1.5%	-1.3%	-2.3%
	Zimbabwe	0.9%	0.3%	1.6%	2.1%	0.4%	1.0%	-1.6%	-1.7%	6.6%	7.1%	-2.6%	-2.7%	0.1%	0.4%	119.9%	88.0%	0.6%	0.6%	-2.5%	-2.2%	0.0%	0.0%	0.1%	0.4%	-0.0%	0.0%	1.6%	1.7%	-0.3%	-1.8%	-0.2%	0.4%	0.4%

5 to 14

Middle SDI

Country	Total communicable		Enteric infections		Lower respiratory infections		Malaria		Neonatal sepsis and other neonatal infections		Vaccine Preventable disease		Meningitis & Encephalitis		HIV/AIDS	
	Female	Male	Female	Male	Female	Male	Female	Male	Female	Male	Female	Male	Female	Male	Female	Male
Albania	-1.9%	-1.6%	-0.6%	-0.5%	-2.6%	-2.1%			3.7%	7.5%	-3.2%	-3.2%	-2.3%	-1.8%	16.4%	17.4%
Algeria	-2.1%	-2.0%	-1.0%	-0.8%	-2.4%	-2.5%	-2.1%	-2.1%	14.6%	18.8%	-3.2%	-3.2%	-2.3%	-2.4%	11.1%	10.9%
Armenia	-0.7%	-0.6%	-0.0%	-0.0%	-1.6%	-1.3%	-3.3%	-3.3%	-0.6%	-0.4%	-1.9%	-2.3%	-1.6%	-1.4%	48.6%	14.2%
Azerbaijan	-1.3%	-1.4%	0.6%	0.1%	-1.7%	-1.8%	-3.3%	-3.3%	5.3%	10.4%	-2.4%	-2.4%	-1.1%	-1.2%	15.9%	13.1%
Botswana	-1.3%	-1.4%	-1.8%	-2.3%	0.2%	0.0%	-0.6%	-0.8%	8.2%	11.8%	-3.0%	-3.1%	-0.7%	-0.7%	99.6%	121.1%
Brazil	-1.1%	-1.2%	-0.7%	-0.7%	-1.9%	-1.9%	-3.2%	-3.2%	7.3%	12.4%	-2.1%	-2.7%	-2.3%	-2.4%	0.3%	-0.8%
China	-1.8%	-1.8%	-1.4%	-1.7%	-2.8%	-2.8%	-3.3%	-3.3%	23.2%	20.0%	-3.2%	-3.2%	-2.6%	-2.5%	58.7%	51.6%
Colombia	-1.2%	-1.3%	-1.3%	-1.4%	-2.1%	-2.2%	-3.3%	-3.3%	4.1%	8.9%	-2.4%	-2.6%	-1.9%	-2.0%	7.4%	1.9%
Costa Rica	-0.9%	-0.8%	-0.5%	-0.4%	-1.9%	-1.1%	-3.3%	-3.3%	-1.6%	-2.2%	-2.0%	-1.7%	-2.0%	-2.0%	-0.3%	0.1%
Cuba	-1.0%	-0.8%	0.9%	1.6%	-1.3%	-1.3%			-0.4%	-0.7%	-1.0%	-0.9%	-2.8%	-2.7%	4.3%	9.7%
Ecuador	-2.0%	-2.0%	-1.5%	-1.9%	-1.9%	-1.8%	-3.3%	-3.3%	-0.5%	-0.4%	-2.4%	-2.5%	-1.9%	-2.0%	11.4%	11.3%
Egypt	-2.5%	-2.3%	-1.4%	-1.7%	-2.7%	-2.5%	-3.3%	-3.3%	11.6%	12.1%	-3.2%	-3.2%	-2.2%	-2.1%	11.7%	16.3%
Equatorial Guinea	-2.0%	-2.1%	-3.0%	-2.9%	-2.6%	-2.6%	0.0%	-0.8%	88.7%	114.7%	-3.1%	-3.2%	-2.7%	-2.8%	516.5%	570.7%
Fiji	-1.1%	-1.1%	-1.4%	-1.2%	-0.8%	-0.4%			0.2%	1.8%	-2.6%	-2.7%	-1.8%	-1.3%	166.9%	166.6%
Gabon	-1.7%	-1.8%	-2.3%	-2.2%	-2.2%	-1.8%	-1.0%	-1.5%	20.6%	23.4%	-2.7%	-2.7%	-2.3%	-2.1%	153.0%	184.4%
Grenada	-1.0%	-0.4%	0.6%	1.0%	-1.7%	-0.8%			4.0%	5.1%	-2.6%	-2.6%	-2.7%	-2.5%	-1.8%	-1.4%
Guyana	-1.5%	-1.5%	-1.4%	-1.8%	-1.0%	-0.8%	-2.8%	-2.9%	2.2%	7.3%	-2.8%	-2.8%	-1.8%	-2.0%	0.2%	-1.3%
Indonesia	-2.3%	-2.2%	-2.4%	-2.3%	-2.5%	-2.5%	-2.8%	-2.9%	13.6%	10.8%	-3.0%	-3.0%	-2.2%	-2.3%	8.1e+14%	6.2e+14%
Iran (Islamic Republic of)	-1.6%	-1.5%	-0.2%	-0.2%	-2.0%	-2.0%	-3.3%	-3.3%	7.3%	19.3%	-3.2%	-3.2%	-1.9%	-2.0%	73.2%	74.3%
Iraq	-2.2%	-2.2%	-1.2%	-1.1%	-2.5%	-2.6%	-3.3%	-3.3%	5.1%	13.7%	-3.0%	-3.0%	-2.1%	-2.3%	48.2%	56.6%
Jamaica	-0.6%	-0.8%	-0.8%	-1.3%	-2.3%	-2.0%			9.6%	8.7%	-2.2%	-2.9%	-2.0%	-2.2%	2.0%	-0.6%
Mexico	-1.6%	-1.6%	-2.3%	-2.3%	-2.5%	-2.5%	-3.3%	-3.3%	2.4%	2.5%	-2.6%	-2.7%	-1.9%	-1.9%	1.5%	-0.5%
Namibia	-1.1%	-1.0%	-1.8%	-1.7%	-1.3%	-0.7%	-2.5%	-2.6%	13.0%	14.8%	-3.2%	-3.2%	-1.6%	-1.2%	458.9%	586.6%
Nauru	-0.9%	-0.9%	-1.2%	-1.2%	-0.9%	-0.8%			2.8%	2.7%	-3.0%	-3.0%	-1.3%	-1.4%	575.5%	623.9%
Panama	-0.9%	-0.9%	-0.9%	-0.9%	-0.7%	-0.4%	-2.0%	-1.8%	1.6%	1.2%	-2.6%	-2.7%	-1.9%	-2.1%	1.5%	1.0%
Paraguay	-1.2%	-1.2%	-1.0%	-1.3%	-2.1%	-2.2%	-3.3%	-3.3%	-2.6%	-1.8%	-2.7%	-2.8%	-2.2%	-2.2%	111.6%	107.9%
Peru	-1.7%	-1.5%	-1.4%	-1.0%	-2.3%	-2.2%	-3.2%	-3.1%	12.5%	16.3%	-2.4%	-2.5%	-1.9%	-1.7%	55.0%	51.4%
Philippines	-1.5%	-1.6%	-1.0%	-1.3%	-2.0%	-2.0%	-3.2%	-3.2%	5.2%	7.8%	-2.7%	-2.7%	-1.9%	-1.9%	12.7%	9.9%
Saint Lucia	-0.1%	-0.6%	1.1%	0.9%	-0.2%	-0.8%			4.6%	5.9%	-2.0%	-1.4%	-1.5%	-2.1%	-0.9%	-1.7%
Saint Vincent and the Grenadines	-0.6%	-0.8%	-0.3%	-0.3%	-1.2%	-1.6%			1.8%	5.1%	-1.4%	-1.6%	-2.4%	-2.6%	1.6%	-0.7%
Samoa	-1.1%	-1.1%	-1.4%	-1.2%	-2.0%	-2.0%			1.5%	1.6%	-0.7%	-0.6%	-2.0%	-2.0%	497.5%	548.9%
South Africa	0.2%	0.5%	-1.9%	-2.0%	-2.0%	-2.0%	-2.1%	-2.1%	15.7%	16.2%	-2.6%	-2.6%	-2.1%	-2.2%	2,215.5%	2,694.5%
Suriname	-1.4%	-1.2%	-0.4%	-0.6%	-1.4%	-1.2%	-3.3%	-3.3%	5.7%	8.4%	-2.8%	-2.7%	-2.1%	-2.1%	-1.7%	-2.0%
Syrian Arab Republic	-1.6%	-1.7%	-0.7%	-0.9%	-0.7%	-0.9%	-3.3%	-3.3%	1.6%	2.9%	-3.1%	-3.1%	-2.1%	-2.1%	4.8%	5.5%
Thailand	-1.8%	-1.9%	-1.9%	-1.8%	-1.8%	-2.0%	-3.3%	-3.3%	13.5%	7.0%	-3.0%	-3.0%	-2.3%	-2.5%	4.7e+14%	3.1e+14%
Tokelau	-1.4%	-1.4%	-1.5%	-1.2%	-2.0%	-2.1%			2.1%	2.9%	-2.3%	-2.4%	-2.0%	-2.1%	533.5%	539.8%
Tonga	-0.9%	-1.1%	-0.2%	-0.2%	-1.0%	-1.0%			1.6%	1.6%	-1.6%	-1.8%	-1.4%	-1.3%	215.7%	236.9%
Tunisia	-1.7%	-1.5%	-1.2%	-0.9%	-2.4%	-2.2%			6.2%	6.1%	-2.9%	-2.8%	-2.2%	-2.1%	69.2%	76.2%
Turkmenistan	-1.4%	-1.4%	-1.0%	-1.3%	-1.7%	-1.7%	-3.3%	-3.3%	11.6%	16.8%	-2.4%	-2.2%	-1.2%	-1.1%	-1.1%	-0.6%
Uzbekistan	-0.7%	-0.7%	-0.5%	-0.6%	-0.1%	-0.0%	-3.3%	-3.3%	14.4%	18.1%	-2.2%	-2.4%	-1.7%	-1.7%	-0.7%	3.3%
Viet Nam	-2.0%	-2.0%	-1.7%	-1.8%	-1.7%	-1.6%	-3.1%	-3.0%	3.5%	5.5%	-3.1%	-3.1%	-1.0%	-0.9%	9.1e+13%	8.4e+13%

Country	Tuberculosis		Neglected Tropical diseases		Infectious skin conditions		Sexually transmitted infections excluding HIV		Upper respiratory infections		Other unspecified infectious diseases		Hepatitis		Rheumatic heart disease		Maternal sepsis and other maternal infections
	Female	Male	Female	Male	Female	Male	Female	Male	Female	Male	Female	Male	Female	Male	Female	Male	Female
Albania	-2.7%	-2.9%	-1.8%	-2.2%	-0.0%	-0.1%	-0.7%	-0.2%	-0.2%	-0.2%	-1.2%	-1.1%	-0.9%	-0.7%	-1.6%	-1.3%	-0.2%
Algeria	-2.9%	-2.9%	-2.0%	-2.2%	-0.0%	-0.0%	-1.1%	-1.1%	-0.1%	-0.1%	-1.3%	-1.4%	-2.3%	-2.4%	-2.5%	-2.4%	-3.2%
Armenia	-1.9%	-2.1%	-0.9%	-0.6%	0.0%	0.0%	-0.3%	-2.1%	-0.4%	-0.1%	-0.8%	-0.4%	-1.7%	-0.7%	-0.8%	-0.8%	-2.2%
Azerbaijan	-2.1%	-2.1%	-1.7%	-1.8%	-0.0%	-0.0%	-1.8%	-1.5%	-0.1%	-0.2%	-1.4%	-1.1%	-1.9%	-2.0%	-0.9%	-1.1%	-2.7%
Botswana	-1.5%	-1.4%	-2.4%	-2.4%	-0.0%	-0.1%	-1.3%	-1.4%	-0.2%	-0.3%	-0.7%	-0.6%	-1.5%	-1.9%	-0.9%	-1.0%	-2.5%
Brazil	-2.5%	-2.7%	-0.6%	-0.8%	0.0%	-0.0%	-0.9%	-1.4%	-0.2%	-0.1%	-1.0%	-0.8%	-1.4%	-1.6%	-1.6%	-1.7%	-1.5%
China	-3.0%	-3.0%	-2.6%	-2.6%	-0.1%	-0.1%	-1.3%	-1.3%	-0.6%	-0.7%	-2.4%	-2.5%	-2.5%	-2.5%	-2.3%	-2.2%	-3.3%
Colombia	-2.6%	-2.8%	1.0%	0.1%	-0.0%	-0.1%	-0.6%	-1.3%	-0.2%	-0.2%	-1.5%	-1.3%	-1.2%	-1.2%	-2.8%	-2.9%	-2.3%
Costa Rica	-2.6%	-2.7%	-1.2%	-1.0%	-0.0%	-0.0%	-0.3%	-0.8%	-0.1%	-0.1%	-0.8%	-0.3%	-2.0%	-1.7%	-0.9%	-0.8%	-2.3%
Cuba	-2.3%	-2.3%	-1.0%	-0.9%	-0.0%	-0.0%	-0.1%	-0.8%	-0.1%	-0.0%	-0.8%	-0.5%	-2.1%	-2.1%	-1.2%	-1.2%	-1.3%
Ecuador	-3.0%	-3.0%	-2.5%	-2.5%	-0.0%	-0.0%	-1.0%	-1.5%	-0.1%	-0.2%	-2.0%	-2.1%	-2.0%	-2.1%	-1.3%	-1.2%	-2.6%
Egypt	-2.9%	-2.9%	-2.9%	-2.8%	-0.2%	-0.3%	-0.9%	-0.8%	-0.4%	-0.4%	-1.7%	-1.3%	-2.9%	-2.6%	-2.8%	-2.6%	-2.3%
Equatorial Guinea	-3.1%	-3.2%	-2.9%	-3.0%	-0.4%	-0.5%	-1.5%	-2.1%	-0.8%	-1.0%	-1.5%	-1.7%	-2.3%	-2.8%	-1.5%	-2.0%	-3.2%
Fiji	-1.7%	-1.1%	-0.3%	-1.9%	-0.0%	-0.0%	-0.7%	0.1%	-0.1%	-0.1%	0.1%	0.5%	-1.7%	-1.8%	-0.8%	-0.3%	-0.9%
Gabon	-2.4%	-2.3%	-2.6%	-2.6%	-0.2%	-0.2%	-1.8%	-1.8%	-0.3%	-0.3%	-0.5%	-0.5%	-1.7%	-2.0%	-0.7%	-1.1%	-2.7%
Grenada	-2.3%	-2.1%	-1.6%	-1.3%	-0.0%	-0.0%	0.2%	-1.3%	-0.2%	-0.3%	-1.0%	-0.6%	-2.4%	-2.1%	-1.9%	-1.0%	-1.9%
Guyana	-2.5%	-2.5%	-0.9%	-1.5%	0.1%	0.1%	0.2%	-2.1%	-0.1%	-0.2%	-0.7%	-0.3%	-2.4%	-2.3%	-1.7%	-1.7%	-2.1%
Indonesia	-2.8%	-2.7%	-2.0%	-1.8%	-0.0%	-0.1%	-0.4%	-1.6%	0.0%	-0.1%	-1.5%	-2.1%	-3.0%	-2.4%	-2.3%	-2.1%	-2.9%
Iran (Islamic Republic of)	-2.7%	-2.9%	-2.3%	-2.4%	-0.0%	-0.0%	0.4%	0.3%	-0.1%	-0.1%	-1.1%	-1.0%	-2.3%	-1.9%	-2.2%	-2.1%	-3.0%
Iraq	-2.9%	-3.0%	-2.0%	-2.4%	-0.1%	-0.1%	-1.2%	-1.1%	-0.2%	-0.2%	-1.4%	-1.6%	-2.6%	-2.5%	-2.4%	-2.3%	-2.2%
Jamaica	-1.9%	-2.5%	-0.1%	0.1%	-0.0%	-0.1%	-0.5%	-0.3%	-0.1%	-0.1%	-0.2%	1.2%	-1.4%	-1.4%	-2.3%	-2.1%	-2.4%
Mexico	-3.0%	-3.0%	-1.9%	-1.9%	0.2%	0.1%	-0.9%	-2.3%	-0.4%	-0.4%	-1.5%	-1.4%	-0.7%	-0.7%	-1.9%	-1.9%	-2.2%
Namibia	-2.1%	-1.8%	-1.2%	-1.3%	-0.1%	-0.1%	-0.8%	-0.4%	-0.3%	-0.3%	-0.7%	-0.7%	-1.3%	-1.3%	-1.3%	-1.2%	-2.5%
Nauru	-1.7%	-1.6%	2.7%	2.7%	-0.0%	-0.1%	-0.2%	-0.2%	0.0%	0.0%	-0.8%	-0.5%	-1.6%	-1.8%	0.4%	-0.1%	-1.1%
Panama	-2.2%	-2.0%	-2.4%	-2.4%	-0.0%	-0.0%	1.2%	0.4%	-0.1%	-0.1%	-0.2%	-0.0%	-2.1%	-1.7%	-1.8%	-1.1%	-1.7%
Paraguay	-2.6%	-2.5%	-1.5%	-1.7%	-0.0%	-0.0%	-0.2%	-0.3%	-0.1%	-0.1%	-0.7%	-0.6%	-1.8%	-1.6%	-1.0%	-1.0%	-2.9%
Peru	-3.1%	-3.1%	-2.2%	-2.0%	-0.1%	-0.1%	-1.7%	-2.1%	-0.1%	-0.2%	-1.6%	-1.0%	-2.4%	-2.4%	-1.0%	-1.0%	-3.1%
Philippines	-2.1%	-2.2%	-1.2%	-1.7%	-0.0%	-0.1%	0.8%	-0.0%	-0.3%	-0.2%	-1.0%	-0.2%	-2.7%	-2.7%	2.4%	2.2%	-2.6%
Saint Lucia	-2.2%	-2.4%	-1.2%	-1.5%	0.0%	-0.0%	1.3%	-1.6%	-0.1%	-0.2%	-0.9%	-0.6%	-1.2%	-1.6%	-0.7%	-1.0%	-1.6%
Saint Vincent and the Grenadines	-2.4%	-2.4%	-1.1%	-1.1%	0.1%	0.1%	-0.0%	-2.5%	-0.1%	-0.3%	-0.8%	-0.5%	-1.9%	-1.4%	-1.4%	-1.3%	-0.9%
Samoa	-2.4%	-2.3%	0.3%	-0.6%	-0.1%	-0.0%	-0.8%	-0.2%	-0.1%	-0.1%	-0.8%	-0.7%	-1.6%	-2.3%	-1.1%	-1.3%	-2.7%
South Africa	-1.7%	-1.8%	-2.3%	-2.3%	-0.0%	-0.1%	-1.6%	-1.5%	-0.4%	-0.3%	0.3%	0.3%	-1.6%	-2.4%	-1.7%	-1.7%	-3.1%
Suriname	-2.5%	-2.3%	0.4%	-0.2%	0.0%	0.0%	-1.0%	-1.2%	-0.1%	-0.1%	-0.6%	-0.1%	-2.2%	-2.2%	-1.5%	-1.1%	-1.1%
Syrian Arab Republic	-2.6%	-2.7%	2.3%	2.1%	-0.2%	-0.2%	-0.5%	-0.2%	-0.2%	-0.2%	-1.4%	-1.3%	-2.3%	-2.2%	-2.6%	-2.6%	-2.9%
Thailand	-2.9%	-3.0%	-2.4%	-2.6%	-0.1%	-0.2%	-0.7%	-1.4%	-0.2%	-0.2%	-1.5%	-1.5%	-2.3%	-2.4%	-1.8%	-1.8%	-1.5%
Tokelau	-2.6%	-2.8%	0.3%	0.0%	-0.0%	-0.1%	-1.1%	-0.9%	-0.1%	-0.2%	-1.3%	-1.0%	-2.0%	-2.1%	-1.5%	-1.8%	-2.5%
Tonga	-1.8%	-1.6%	-1.1%	-1.9%	-0.0%	-0.0%	-0.3%	-0.2%	-0.1%	-0.1%	-0.6%	-0.4%	-2.2%	-2.3%	-0.6%	-0.7%	-1.7%
Tunisia	-2.9%	-2.9%	-1.9%	-2.1%	-0.0%	-0.0%	-1.0%	-0.9%	-0.1%	-0.1%	-1.2%	-1.1%	-2.2%	-2.2%	-2.9%	-2.7%	-3.0%
Turkmenistan	-2.0%	-1.9%	-1.7%	-1.8%	0.0%	0.0%	-1.4%	-1.7%	-0.2%	-0.3%	-0.8%	-0.7%	-2.7%	-2.2%	-1.2%	-1.5%	-1.0%
Uzbekistan	-1.0%	-1.2%	-0.9%	-0.8%	-0.0%	-0.0%	-1.9%	-2.3%	-0.3%	-0.5%	-0.6%	-0.5%	-2.8%	-2.8%	-1.3%	-1.3%	-2.2%
Viet Nam	-2.3%	-2.6%	-3.0%	-2.8%	-0.0%	-0.0%	-0.1%	-0.7%	-0.3%	-0.3%	-2.4%	-2.4%	-2.4%	-2.2%	-2.1%	-2.1%	-2.8%

5 to 14

High-middle SDI

Country	Total communicable		Enteric infections		Lower respiratory infections		Malaria		Neonatal sepsis and other neonatal infections		Vaccine Preventable disease		Meningitis & Encephalitis		HIV/AIDS	
	Female	Male	Female	Male	Female	Male	Female	Male	Female	Male	Female	Male	Female	Male	Female	Male
American Samoa	-0.7%	-0.8%	-1.0%	-0.6%	-1.3%	-1.4%			0.0%	-2.0%	-1.5%	-1.5%	-0.6%	-1.4%	523.2%	526.3%
Antigua and Barbuda	-0.5%	-0.2%	0.8%	1.3%	-1.0%	0.9%			-1.3%	0.3%	-0.9%	-1.8%	-2.1%	-1.7%	-0.5%	-1.0%
Argentina	-0.6%	-0.8%	0.0%	0.3%	0.0%	-0.3%	-3.3%	-3.3%	-2.2%	-2.6%	-1.4%	-2.0%	-1.4%	-1.5%	4.4%	1.0%
Bahamas	-0.0%	-0.6%	1.3%	1.0%	-0.9%	-1.4%			1.3%	-0.4%	-1.2%	-0.8%	-1.6%	-2.1%	2.1%	-0.3%
Bahrain	-0.8%	-1.0%	-0.4%	-0.3%	-0.6%	-1.4%			3.9%	2.3%	-3.0%	-3.0%	-1.6%	-2.0%	21.8%	14.4%
Barbados	-0.3%	-0.3%	1.2%	1.4%	-1.4%	-1.2%			3.2%	3.4%	-2.2%	-2.0%	-2.2%	-2.3%	-1.4%	-2.0%
Belarus	-0.8%	-0.6%	-0.1%	-0.2%	-2.5%	-2.1%			-1.0%	-0.4%	-1.5%	-1.2%	-1.1%	-1.2%	2.8%	2.5%
Bosnia and Herzegovina	-1.0%	-1.0%	-1.0%	-0.8%	-2.2%	-2.3%			-0.2%	-0.4%	-1.5%	-1.4%	-2.0%	-2.0%	5.6%	7.1%
Bulgaria	-1.3%	-1.3%	-0.6%	-0.6%	-2.5%	-2.4%			11.7%	21.1%	1.0%	-0.8%	-1.1%	-1.3%	1.6%	0.8%
Chile	-0.9%	-1.2%	-0.3%	-0.6%	-2.7%	-2.8%			-0.3%	0.3%	-0.7%	-1.0%	-2.0%	-2.2%	5.9%	3.5%
Cook Islands	-1.4%	-1.5%	-1.1%	-0.8%	-2.7%	-2.7%			0.4%	1.2%	-2.4%	-2.4%	-1.9%	-1.8%	618.3%	677.6%
Croatia	-0.5%	-0.5%	-0.6%	-0.6%	-2.4%	-2.4%			1.7%	2.3%	-0.9%	-0.7%	-1.8%	-1.8%	5.8%	-0.5%
Dominica	0.0%	0.0%	0.7%	0.8%	0.3%	0.5%			2.5%	2.2%	0.8%	0.7%	-1.4%	-1.3%	0.1%	0.9%
Georgia	-1.6%	-1.7%	0.7%	0.7%	-2.8%	-2.9%	-3.3%	-3.3%	0.9%	1.7%	2.0%	0.8%	-1.7%	-1.7%	45.5%	35.8%
Greece	-0.3%	-0.4%	-0.4%	-0.4%	-1.0%	-1.2%			-1.3%	-1.6%	-1.5%	-1.1%	-1.5%	-1.2%	0.4%	-2.1%
Greenland	-1.0%	-1.0%	-0.2%	0.0%	-2.3%	-2.3%			2.6%	3.2%	-2.4%	-2.4%	-2.7%	-1.9%	4.5%	2.8%
Hungary	-0.8%	-0.7%	-0.6%	-0.5%	-1.8%	-2.1%			-2.3%	2.0%	-2.3%	-2.0%	-2.3%	-2.4%	-2.7%	-2.7%
Israel	-0.5%	-0.5%	-0.2%	-0.1%	-2.0%	-1.6%			-1.1%	-0.2%	-0.9%	0.0%	-2.2%	-1.8%	-2.0%	-1.7%
Italy	-0.3%	-0.4%	0.0%	0.3%	-2.3%	-2.4%			0.0%	1.2%	-2.2%	-1.6%	-1.8%	-1.7%	-2.0%	-2.4%
Jordan	-1.6%	-1.4%	-0.7%	-0.6%	-1.9%	-1.5%			3.6%	5.0%	-2.9%	-2.9%	-2.1%	-1.8%	29.0%	29.3%
Kazakhstan	-1.3%	-1.4%	-0.0%	-0.1%	-2.2%	-2.4%			1.2%	2.1%	-1.7%	-2.1%	-1.7%	-1.9%	7.5%	1.1%
Lebanon	-1.7%	-1.6%	-0.4%	-0.4%	-1.8%	-1.9%			4.5%	4.7%	-2.9%	-3.0%	-1.8%	-1.8%	6.1%	6.0%
Libya	-1.5%	-1.5%	0.0%	-0.2%	-1.8%	-2.0%			1.0%	2.2%	-3.1%	-3.1%	-1.7%	-2.0%	19.3%	20.2%
Malaysia	-1.5%	-1.4%	-1.5%	-1.4%	-1.7%	-1.7%	-3.3%	-3.2%	-0.4%	-0.6%	-3.0%	-3.0%	-1.7%	-1.8%	1.3e+09%	1.2e+09%
Malta	-0.4%	-0.4%	-0.5%	-0.4%	-1.1%	-0.8%			0.1%	0.2%	-1.9%	-1.2%	-1.5%	-1.3%	-0.8%	-1.4%
Mauritius	-0.9%	-0.7%	-1.2%	-1.4%	-2.4%	-2.3%			0.1%	2.6%	-1.4%	-1.8%	-1.1%	-1.1%	10.1%	10.5%
Montenegro	-0.8%	-0.8%	-0.7%	-0.8%	-2.1%	-1.9%			-1.5%	-1.2%	-1.5%	-1.5%	-1.8%	-1.6%	51.0%	58.5%
Niue	-0.7%	-0.8%	-1.3%	-1.1%	-1.0%	-1.1%			2.0%	2.6%	-1.3%	-1.4%	-1.8%	-1.8%	544.4%	604.6%
North Macedonia	-0.8%	-0.9%	-0.5%	-0.7%	-2.2%	-2.1%			31.3%	47.1%	-1.0%	-0.8%	-1.8%	-2.0%	40.3%	46.5%
Northern Mariana Islands	-0.1%	-0.2%	-0.5%	-0.4%	-1.1%	-0.9%			3.1%	0.3%	-0.5%	-0.2%	0.1%	-0.5%	334.8%	398.8%
Oman	-1.8%	-1.6%	-1.0%	-0.5%	-2.0%	-2.0%	-3.3%	-3.3%	4.7%	6.8%	-3.2%	-3.2%	-1.6%	-1.5%	40.6%	33.0%
Palau	-1.0%	-1.1%	-1.6%	-1.6%	-1.3%	-1.5%			2.1%	3.2%	-1.9%	-2.0%	-1.7%	-0.9%	613.6%	679.7%
Poland	-0.8%	-0.9%	-0.7%	-0.7%	-1.3%	-1.4%			-2.6%	-2.7%	-2.4%	-2.2%	-2.2%	-2.5%	9.7%	6.8%
Portugal	-0.9%	-1.2%	-0.4%	-0.6%	-2.5%	-2.6%			-1.8%	-1.3%	-1.9%	-2.7%	-2.6%	-2.5%	2.9%	-0.1%
Republic of Moldova	-0.3%	-0.4%	-0.0%	-0.0%	-1.6%	-1.4%			2.4%	4.4%	-2.3%	-1.0%	-1.8%	-1.9%	19.5%	1.3%
Romania	-1.4%	-1.5%	-0.6%	-0.6%	-2.1%	-2.1%			-0.4%	2.4%	-2.0%	-2.4%	-2.4%	-2.6%	-1.3%	-0.1%
Russian Federation	-0.5%	-0.7%	-0.4%	-0.5%	-1.1%	-1.3%			-0.1%	0.3%	-2.3%	-2.5%	-1.6%	-1.8%	1.1%	1.2%
Saint Kitts and Nevis	-0.8%	-0.7%	-0.6%	-0.8%	-1.4%	-1.3%			3.3%	3.5%	-2.2%	-1.6%	-2.3%	-2.4%	95.8%	91.5%
Saudi Arabia	-2.3%	-2.2%	-1.7%	-1.4%	-2.5%	-2.4%	-2.3%	-2.7%	0.1%	1.0%	-3.3%	-3.3%	-2.4%	-2.6%	14.9%	15.9%
Serbia	-1.1%	-1.2%	-0.7%	-0.7%	-2.6%	-2.6%			-2.1%	-1.8%	-2.3%	-2.2%	-2.0%	-2.2%	8.2%	12.6%
Seychelles	-1.1%	-0.6%	-1.5%	-0.5%	-1.0%	0.7%			1.6%	1.2%	-2.6%	-2.6%	-2.9%	-2.5%	93.9%	78.0%
Spain	-0.6%	-0.9%	-0.4%	-0.4%	-2.3%	-2.5%			0.2%	0.9%	-0.9%	-1.0%	-2.4%	-2.3%	-2.3%	-2.8%
Sri Lanka	-1.9%	-2.0%	-2.1%	-2.2%	-2.4%	-2.3%	-3.3%	-3.3%	3.8%	3.4%	-3.2%	-3.2%	-2.3%	-2.3%	11.4%	10.8%
Trinidad and Tobago	-1.0%	-1.1%	-0.8%	-0.3%	-2.3%	-2.3%			0.7%	2.0%	-0.3%	-1.2%	-2.5%	-2.7%	0.8%	-0.4%
Turkey	-1.9%	-2.0%	-0.9%	-0.9%	-2.5%	-2.6%	-3.3%	-3.3%	8.2%	12.3%	-3.2%	-3.2%	-2.5%	-2.5%	1.9e+14%	9.0e+13%
Ukraine	-0.4%	-0.4%	0.4%	0.4%	-1.0%	-0.6%			-2.0%	-1.3%	1.0%	0.2%	-0.5%	-1.1%	6.6%	0.4%
United States Virgin Islands	-0.3%	-0.6%	1.4%	1.1%	-1.8%	-2.2%			0.2%	2.1%	-1.7%	-1.5%	-1.5%	-2.3%	0.3%	-1.4%
Uruguay	-0.4%	-0.4%	0.3%	0.5%	-0.7%	-1.0%			-1.4%	-0.4%	-1.4%	-0.2%	-1.6%	-1.8%	2.8%	4.4%

Country	Tuberculosis		Neglected Tropical diseases		Infectious skin conditions		Sexually transmitted infections excluding HIV		Upper respiratory infections		Other unspecified infectious diseases		Hepatitis		Rheumatic heart disease		Maternal sepsis and other maternal infections
	Female	Male	Female	Male	Female	Male	Female	Male	Female	Male	Female	Male	Female	Male	Female	Male	Female
American Samoa	-1.9%	-2.3%	0.4%	-1.1%	-0.1%	-0.1%	-1.6%	-1.5%	-0.0%	-0.0%	-0.7%	-0.1%	-0.7%	-0.3%	-0.5%	-1.0%	-1.2%
Antigua and Barbuda	-2.0%	-2.0%	-0.7%	-0.2%	-0.0%	0.1%	-0.1%	-1.0%	-0.1%	-0.1%	-1.0%	-0.4%	-1.8%	-1.6%	-1.0%	-0.4%	-1.3%
Argentina	-2.6%	-2.7%	-2.4%	-2.4%	0.0%	0.0%	-0.3%	-1.7%	-0.0%	-0.0%	-1.0%	-0.8%	-1.7%	-1.6%	-0.7%	-0.9%	-2.1%
Bahamas	-2.1%	-2.3%	-0.5%	-0.5%	-0.0%	-0.0%	0.8%	-0.5%	-0.1%	-0.1%	-0.6%	-0.3%	-1.6%	-1.5%	-0.7%	-0.7%	-1.6%
Bahrain	-2.4%	-2.6%	-1.8%	-1.8%	-0.3%	-0.4%	-0.2%	-0.2%	-0.1%	-0.1%	-1.6%	-1.4%	-0.8%	-1.1%	-2.0%	-2.7%	-3.0%
Barbados	-2.2%	-2.2%	-0.3%	0.1%	0.0%	-0.1%	0.1%	-1.4%	-0.1%	-0.0%	-1.1%	-0.6%	-2.0%	-1.7%	-1.3%	-0.9%	-1.9%
Belarus	-2.3%	-2.4%	-1.4%	-1.5%	0.0%	0.0%	-0.6%	-2.1%	-0.2%	-0.2%	-0.0%	-0.2%	-1.2%	-0.7%	-2.1%	-2.7%	-2.4%
Bosnia and Herzegovina	-2.8%	-2.6%	-1.5%	-1.8%	-0.0%	-0.0%	-0.6%	-0.5%	-0.2%	-0.2%	-1.1%	-1.1%	-1.4%	-1.2%	-2.4%	-2.6%	-3.1%
Bulgaria	-1.6%	-1.7%	-1.7%	-2.2%	0.1%	0.0%	-0.4%	-0.5%	-0.1%	-0.1%	-0.8%	-0.8%	-0.7%	-0.9%	-2.8%	-2.9%	-1.8%
Chile	-2.5%	-2.9%	-2.4%	-2.4%	-0.0%	-0.0%	-0.1%	-2.1%	-0.2%	-0.2%	-0.4%	-0.4%	-2.0%	-1.6%	-2.6%	-2.8%	-2.9%
Cook Islands	-2.7%	-2.5%	-1.9%	-2.2%	-0.0%	-0.0%	-0.8%	-0.5%	-0.1%	-0.2%	-1.2%	-0.9%	-0.9%	-1.6%	-2.4%	-2.6%	-1.0%
Croatia	-2.5%	-2.7%	-1.6%	-1.5%	-0.0%	-0.0%	-0.3%	-0.6%	-0.1%	-0.0%	-1.1%	-1.1%	-0.5%	-0.6%	-2.8%	-2.5%	-2.3%
Dominica	-1.8%	-1.4%	-0.5%	-0.9%	0.0%	0.0%	-0.4%	-0.4%	-0.2%	-0.1%	-0.3%	0.4%	-2.1%	-1.6%	-0.6%	-0.4%	0.5%
Georgia	-2.1%	-2.4%	-0.3%	-0.2%	0.1%	0.0%	-0.3%	-0.8%	0.1%	-0.0%	-0.2%	0.0%	-1.5%	-0.7%	-0.9%	-1.1%	-0.7%
Greece	-2.2%	-2.6%	-1.2%	-1.1%	-0.0%	-0.0%	-0.2%	-1.1%	-0.1%	-0.0%	-0.3%	-0.2%	-0.4%	-0.5%	-1.4%	-1.5%	-1.1%
Greenland	-2.6%	-2.9%	-1.3%	-1.2%	-0.0%	-0.0%	-0.5%	-0.2%	-0.1%	-0.1%	-1.2%	-1.2%	-1.3%	-0.5%	-2.2%	-1.9%	-1.3%
Hungary	-2.2%	-2.3%	-1.3%	-1.3%	0.1%	0.1%	-0.7%	-1.0%	-0.1%	-0.1%	-0.9%	-1.0%	-0.7%	-0.8%	-2.6%	-2.9%	-1.3%
Israel	-2.7%	-2.8%	-1.7%	-1.3%	0.0%	-0.0%	-0.5%	-1.2%	0.0%	0.0%	-0.7%	-0.5%	-2.1%	-1.8%	-2.4%	-2.0%	-3.2%
Italy	-2.6%	-2.8%	-1.5%	-0.8%	0.1%	0.0%	-0.5%	-2.5%	-0.0%	-0.0%	-0.3%	-0.0%	0.1%	-0.4%	-2.0%	-2.0%	-2.6%
Jordan	-2.8%	-2.7%	-2.0%	-2.2%	-0.1%	-0.1%	-0.8%	-0.7%	-0.1%	-0.1%	-1.6%	-1.5%	-2.0%	-2.2%	-2.5%	-2.3%	-2.6%
Kazakhstan	-2.4%	-2.7%	-1.2%	-1.0%	0.0%	0.0%	0.4%	-1.8%	-0.2%	-0.1%	-0.4%	-0.0%	-2.5%	-2.4%	-2.6%	-2.7%	-0.0%
Lebanon	-2.7%	-2.7%	-2.4%	-2.6%	-0.0%	-0.0%	-0.2%	-0.4%	-0.1%	-0.1%	-1.3%	-1.4%	-2.2%	-1.9%	-2.8%	-2.6%	-2.8%
Libya	-2.6%	-2.6%	-1.1%	-1.4%	-0.1%	-0.1%	-0.1%	-0.4%	-0.0%	-0.1%	-1.0%	-1.1%	-2.3%	-2.0%	-2.3%	-2.4%	-2.7%
Malaysia	-1.9%	-2.4%	-1.9%	-1.8%	-0.0%	-0.0%	-0.6%	-0.6%	-0.2%	-0.2%	-1.0%	-0.9%	-1.4%	-1.2%	-2.2%	-2.2%	-2.7%
Malta	-1.9%	-1.9%	-1.8%	-1.6%	0.0%	0.0%	-0.5%	-1.0%	-0.1%	-0.1%	-0.8%	-0.2%	-0.5%	-0.2%	-1.5%	-0.9%	-0.5%
Mauritius	-0.6%	-2.2%	-0.5%	-0.4%	-0.0%	-0.0%	-0.0%	-0.7%	-0.2%	-0.2%	-1.4%	-1.1%	-1.8%	-1.9%	-2.2%	-2.3%	-1.4%
Montenegro	-2.4%	-2.4%	-0.6%	-0.9%	-0.0%	-0.0%	-0.5%	-0.3%	-0.0%	-0.0%	-0.7%	-1.0%	-0.6%	-0.6%	-2.3%	-2.1%	-1.3%
Niue	-2.1%	-2.0%	1.4%	-0.1%	-0.1%	-0.1%	0.1%	0.1%	-0.1%	-0.2%	-1.2%	-0.8%	-1.6%	-1.7%	-0.4%	-0.6%	-1.3%
North Macedonia	-2.9%	-2.9%	-1.5%	-1.3%	-0.0%	-0.0%	-0.3%	-0.1%	-0.0%	-0.0%	-1.5%	-1.4%	-0.9%	-1.0%	-2.4%	-2.6%	-2.0%
Northern Mariana Islands	-1.9%	-2.1%	2.6%	1.8%	0.0%	-0.1%	-1.2%	-1.1%	0.0%	-0.1%	-0.7%	-0.4%	-0.0%	-0.3%	-0.3%	0.2%	-0.7%
Oman	-2.8%	-2.8%	-2.2%	-2.2%	-0.0%	-0.0%	-0.3%	-0.6%	-0.1%	-0.1%	-1.8%	-1.7%	-2.5%	-1.7%	-2.7%	-2.2%	-2.9%
Palau	-1.9%	-2.1%	1.6%	1.4%	-0.0%	-0.0%	0.1%	-0.1%	-0.0%	-0.1%	-1.0%	-0.6%	-1.5%	-2.2%	-1.1%	-1.4%	-0.9%
Poland	-2.5%	-2.7%	-1.5%	-1.5%	-0.0%	-0.0%	-0.0%	-1.6%	-0.2%	-0.3%	-1.4%	-1.4%	-0.8%	-1.2%	-2.9%	-3.0%	-3.1%
Portugal	-2.9%	-3.0%	-2.2%	-1.9%	0.0%	-0.0%	-2.0%	-2.8%	-0.2%	-0.1%	-1.5%	-1.4%	-1.6%	-1.3%	-3.0%	-3.0%	-2.6%
Republic of Moldova	-1.5%	-1.9%	-1.3%	-1.4%	-0.1%	-0.1%	-1.8%	-1.4%	-0.1%	-0.1%	-0.8%	-0.7%	-2.3%	-2.9%	-2.6%	-2.7%	-2.5%
Romania	-1.7%	-2.2%	-2.0%	-2.2%	0.2%	0.2%	-0.3%	-0.8%	-0.2%	-0.2%	-1.9%	-2.1%	-2.5%	-2.7%	-2.6%	-2.7%	-1.1%
Russian Federation	-2.1%	-2.4%	-1.4%	-1.4%	0.0%	0.0%	-0.6%	-1.7%	-0.2%	-0.2%	-0.8%	-0.8%	-1.1%	-1.2%	-2.4%	-2.7%	-2.8%
Saint Kitts and Nevis	-2.5%	-2.4%	-0.6%	-0.5%	0.0%	-0.1%	0.2%	-1.9%	-0.2%	-0.4%	-1.1%	-0.6%	-2.6%	-1.8%	-2.5%	-2.4%	-1.6%
Saudi Arabia	-3.0%	-2.8%	-2.3%	-2.5%	-0.1%	-0.1%	-0.8%	-0.7%	-0.1%	-0.1%	-1.3%	-1.3%	-2.0%	-2.1%	-2.9%	-2.9%	-3.0%
Serbia	-2.9%	-3.0%	-1.9%	-2.3%	0.0%	0.0%	-1.0%	-1.0%	-0.1%	-0.0%	-1.2%	-1.4%	-1.2%	-1.2%	-2.7%	-2.9%	-1.7%
Seychelles	0.0%	-1.8%	0.9%	1.3%	-0.0%	-0.0%	-1.4%	-0.2%	-0.2%	-0.2%	-0.9%	1.0%	-1.7%	-1.6%	-1.7%	-1.7%	-0.9%
Spain	-2.8%	-3.0%	-2.2%	-1.7%	0.1%	0.0%	-1.0%	-3.0%	-0.0%	-0.0%	-0.7%	-0.9%	-0.6%	-0.8%	-2.4%	-2.5%	-1.4%
Sri Lanka	-2.2%	-2.5%	-1.6%	-2.1%	-0.0%	-0.0%	-0.3%	-0.6%	-0.3%	-0.3%	-1.9%	-1.9%	-1.8%	-1.8%	-2.3%	-2.4%	-2.8%
Trinidad and Tobago	-2.3%	-2.3%	-0.6%	-0.1%	-0.0%	-0.1%	0.0%	-0.3%	-0.1%	-0.2%	-0.8%	-0.6%	-2.0%	-2.3%	-1.7%	-1.7%	-2.3%
Turkey	-2.6%	-3.0%	-2.4%	-2.7%	-0.1%	-0.1%	-1.0%	-1.0%	-0.1%	-0.2%	-2.0%	-1.8%	-2.8%	-2.9%	-2.5%	-1.9%	-2.7%
Ukraine	-1.0%	-0.8%	-0.8%	-0.9%	0.0%	0.0%	0.1%	-2.1%	-0.0%	0.0%	0.7%	0.2%	-1.0%	-1.0%	-0.0%	-0.4%	1.0%
United States Virgin Islands	-2.4%	-2.3%	-0.4%	-0.4%	0.0%	-0.0%	-0.9%	-1.6%	-0.1%	-0.1%	-0.8%	-0.9%	-1.5%	-1.7%	-2.5%	-2.7%	-1.7%
Uruguay	-1.9%	-2.5%	-1.6%	-1.6%	-0.0%	-0.0%	-2.2%	-2.8%	-0.1%	-0.1%	-1.3%	-1.1%	-1.2%	-1.1%	-1.5%	-2.0%	-1.9%

5 to 14

		Total communicable		Enteric infections		Lower respiratory infections		Malaria		Neonatal sepsis and other neonatal infections		Vaccine Preventable disease		Meningitis & Encephalitis		HIV/AIDS		Tuberculosis		Neglected Tropical diseases		Infectious skin conditions		Sexually transmitted infections excluding HIV		Upper respiratory infections		Other unspecified infectious diseases		Hepatitis		Rheumatic heart disease		Maternal sepsis and other maternal infections
		Female	Male	Female	Male	Female	Male	Female	Male	Female	Male	Female	Male	Female	Male	Female	Male	Female	Male	Female	Male	Female	Male	Female	Male	Female	Male	Female	Male	Female	Male	Female	Male	Female
High SDI	Andorra	-0.4%	-0.5%	-0.2%	-0.2%	-2.0%	-2.2%			-0.7%	-0.3%	-2.8%	-2.7%	-1.5%	-1.9%	-1.9%	-2.1%	-2.5%	-2.6%	-1.5%	-1.0%	0.0%	-0.0%	-0.6%	-0.8%	-0.0%	-0.0%	-1.0%	-1.0%	-2.3%	-2.0%	-1.9%	-1.6%	-1.4%
High SDI	Australia	-0.2%	-0.3%	0.1%	0.3%	-1.4%	-1.8%			-0.5%	-0.5%	-0.3%	-0.4%	-1.8%	-1.9%	-0.8%	-3.0%	-1.9%	-2.3%	-1.0%	-1.0%	0.0%	0.0%	-0.7%	-1.9%	-0.1%	-0.1%	-0.1%	-0.5%	-0.2%	-0.3%	-2.1%	-2.3%	-1.5%
High SDI	Austria	-0.3%	-0.5%	-0.3%	-0.3%	-2.1%	-2.4%			1.1%	0.9%	-0.3%	0.0%	-1.4%	-2.1%	-0.3%	-2.1%	-2.4%	-2.8%	-1.7%	-1.4%	-0.0%	-0.1%	-0.1%	-0.8%	-0.1%	-0.1%	0.5%	-0.0%	-0.5%	-0.4%	-1.4%	-2.2%	-1.9%
High SDI	Belgium	-0.3%	-0.4%	-0.3%	-0.3%	-1.5%	-1.7%			-0.5%	-1.0%	-1.3%	-1.2%	-1.3%	-1.4%	-2.1%	-2.5%	-2.5%	-2.5%	-1.7%	-1.3%	0.0%	-0.0%	-0.3%	-2.2%	-0.1%	-0.1%	-0.1%	-0.2%	-1.1%	-0.7%	-0.8%	-1.0%	-2.0%
High SDI	Bermuda	-0.7%	-0.6%	1.4%	1.5%	-1.5%	-0.9%			-2.1%	0.6%	-0.9%	-0.4%	-2.2%	-2.0%	-1.6%	-2.4%	-2.3%	-2.2%	-1.5%	-1.0%	-0.0%	-0.0%	-0.1%	-0.8%	-0.1%	-0.1%	-1.6%	-1.2%	-1.0%	-0.6%	-2.7%	-2.4%	-2.5%
High SDI	Brunei Darussalam	-0.4%	-1.1%	-0.9%	-0.8%	0.4%	-1.4%			4.7%	3.9%	-1.6%	-1.9%	-1.5%	-1.8%	14.3%	19.2%	-2.2%	-2.8%	-0.8%	-1.5%	-0.0%	-0.0%	-0.1%	-0.5%	-0.0%	-0.1%	-0.3%	-0.8%	-1.5%	-1.9%	-1.9%	-2.6%	-1.6%
High SDI	Canada	-0.1%	-0.2%	0.6%	0.6%	-1.8%	-2.0%			3.3%	-1.0%	-1.0%	-1.3%	-1.7%	-1.7%	-0.3%	-2.4%	-1.6%	-2.1%	-1.4%	-1.2%	-0.0%	-0.0%	-0.2%	-0.6%	-0.1%	-0.1%	-0.5%	-0.5%	-0.5%	-0.4%	-1.9%	-1.6%	-1.6%
High SDI	Cyprus	-0.4%	-0.8%	-0.6%	-0.6%	-1.5%	-2.2%			0.0%	0.1%	-2.8%	-2.8%	-1.9%	-2.6%	14.8%	9.7%	-2.6%	-3.0%	-2.4%	-2.2%	-0.0%	-0.0%	-0.5%	-0.7%	-0.0%	-0.0%	-1.2%	-1.5%	-0.7%	-1.0%	-1.7%	-2.4%	-2.7%
High SDI	Czechia	-0.5%	-0.6%	-0.7%	-0.7%	-1.8%	-1.8%			2.7%	6.4%	-0.7%	-0.8%	-1.6%	-1.7%	11.2%	3.7%	-2.2%	-2.4%	-1.3%	-1.3%	0.1%	0.1%	-0.9%	-1.4%	-0.2%	-0.1%	-1.1%	-1.1%	-0.7%	-1.0%	-2.7%	-2.9%	-3.0%
High SDI	Denmark	-0.4%	-0.6%	-0.3%	-0.2%	-1.8%	-2.1%			0.3%	0.6%	-0.6%	-2.3%	-2.5%	-2.6%	5.5%	4.9%	-2.6%	-2.7%	-1.7%	-1.3%	0.0%	0.0%	-0.5%	-0.6%	-0.0%	-0.0%	-0.7%	-0.6%	-1.5%	-0.6%	-1.8%	-2.4%	-0.5%
High SDI	Estonia	-0.4%	-1.1%	0.7%	-0.2%	-1.6%	-2.1%			-1.3%	-2.8%	-1.9%	-1.7%	-2.2%	-2.1%	36.9%	4.6%	-2.4%	-2.4%	-1.7%	-1.7%	-0.0%	-0.0%	-0.4%	-3.0%	-0.1%	-0.1%	-0.9%	-1.0%	-1.0%	-1.4%	-2.2%	-2.8%	-2.6%
High SDI	Finland	-0.4%	-0.6%	-0.5%	-0.4%	-2.6%	-2.8%			-0.6%	0.3%	-1.3%	-1.4%	-1.5%	-2.2%	-2.0%	-2.4%	-1.8%	-2.3%	-1.8%	-1.3%	-0.0%	-0.1%	-0.3%	-0.6%	-0.1%	-0.0%	-0.7%	-0.8%	0.4%	-0.2%	-0.9%	-1.9%	-2.8%
High SDI	France	-0.4%	-0.6%	-0.4%	-0.3%	-2.0%	-2.2%			-1.3%	-1.6%	-1.8%	-1.8%	-1.9%	-1.9%	-2.1%	-3.0%	-2.5%	-2.7%	-1.9%	-1.6%	-0.0%	-0.0%	-0.0%	-1.6%	-0.1%	-0.1%	-0.7%	-0.8%	-0.8%	-1.1%	-1.7%	-1.8%	-0.6%
High SDI	Germany	-0.3%	-0.6%	-0.2%	-0.2%	-1.9%	-2.0%			-0.4%	-0.4%	-0.9%	-1.4%	-2.1%	-1.9%	-1.4%	-2.9%	-2.8%	-2.9%	-1.8%	-1.5%	-0.0%	-0.0%	-0.4%	-1.4%	-0.1%	-0.1%	-1.3%	-1.0%	-0.6%	-1.1%	-2.0%	-2.1%	-2.6%
High SDI	Guam	-0.3%	-0.9%	-0.5%	-0.9%	-0.4%	-2.3%			0.3%	0.8%	-0.9%	-1.3%	-0.4%	-2.1%	782.0%	849.2%	-1.2%	-2.1%	-0.5%	-0.4%	0.1%	-0.0%	-0.0%	-1.1%	-0.0%	-0.1%	-0.6%	-0.5%	0.3%	-1.0%	-0.3%	-1.6%	-1.1%
High SDI	Iceland	-0.5%	-0.5%	-0.4%	-0.3%	-2.0%	-2.1%			0.2%	-0.7%	-0.5%	-0.5%	-2.1%	-2.0%	-2.2%	-2.5%	-2.7%	-2.5%	-1.4%	-1.0%	-0.0%	-0.0%	-0.6%	-2.2%	-0.0%	-0.1%	-0.7%	-0.6%	-0.4%	-0.1%	-1.3%	-1.6%	-1.9%
High SDI	Ireland	-0.5%	-0.7%	-0.5%	-0.4%	-2.4%	-2.5%			0.5%	1.5%	-1.3%	-1.7%	-2.5%	-2.4%	0.0%	-2.2%	-2.1%	-2.7%	-1.9%	-1.4%	0.0%	-0.0%	-0.4%	-1.4%	-0.0%	-0.0%	-0.5%	-0.8%	-0.6%	-0.7%	-1.5%	-1.2%	-0.4%
High SDI	Japan	-0.4%	-0.5%	-1.3%	-1.1%	-1.6%	-1.7%			-0.7%	-0.6%	-2.4%	-2.3%	-1.7%	-1.8%	2.6%	4.6%	-2.6%	-2.8%	-1.3%	-1.2%	0.0%	0.0%	0.3%	-1.0%	-0.0%	-0.1%	-0.7%	-0.7%	-0.2%	-0.7%	-1.5%	-1.7%	-2.2%
High SDI	Kuwait	-0.7%	-0.7%	-0.2%	-0.1%	-1.1%	-1.5%			-2.0%	-0.7%	-2.4%	-2.4%	-1.7%	-1.5%	0.7%	-2.3%	-2.7%	-2.6%	-1.2%	-1.1%	-0.0%	-0.0%	-0.3%	-0.8%	-0.1%	-0.1%	-1.1%	-0.8%	-1.8%	-1.2%	-2.9%	-2.7%	-2.4%
High SDI	Latvia	-0.9%	-0.9%	-0.5%	-0.4%	-1.3%	-1.9%			-2.1%	0.6%	-0.9%	-0.7%	-2.3%	-2.3%	7.9%	0.4%	-2.4%	-2.7%	-1.3%	-1.1%	-0.1%	-0.1%	0.0%	-0.6%	-0.1%	-0.1%	-0.4%	-0.3%	-2.7%	-2.7%	-2.4%	-2.8%	-2.4%
High SDI	Lithuania	-0.2%	-0.4%	0.1%	-0.3%	-0.4%	-0.7%			7.7%	5.3%	-1.6%	-0.9%	-1.9%	-2.1%	2.5%	14.6%	-2.1%	-2.1%	-0.8%	-1.1%	0.0%	0.0%	-0.2%	-1.1%	-0.2%	-0.2%	-0.2%	-0.5%	-0.9%	-0.0%	-2.3%	-1.3%	-2.8%
High SDI	Luxembourg	-0.6%	-0.5%	-0.3%	-0.2%	-2.4%	-2.0%			-0.3%	-0.2%	-2.4%	-2.0%	-2.3%	-2.1%	-2.2%	-2.4%	-2.7%	-2.3%	-2.0%	-1.4%	-0.0%	-0.0%	-0.8%	-2.5%	-0.1%	-0.1%	-1.3%	-1.0%	-0.7%	-0.6%	-2.3%	-1.7%	-2.5%
High SDI	Monaco	-0.2%	-0.4%	-0.3%	-0.2%	-1.5%	-1.7%			-0.5%	0.2%	-0.6%	-0.8%	-1.9%	-2.2%	-2.0%	-2.2%	-2.1%	-2.6%	-1.4%	-0.9%	-0.0%	-0.0%	-0.8%	-1.0%	-0.0%	-0.0%	-0.4%	-0.3%	-0.4%	-0.3%	-0.5%	-1.3%	-1.8%
High SDI	Netherlands	-0.4%	-0.6%	-0.3%	-0.3%	-1.6%	-1.9%			-0.5%	-0.9%	0.2%	-0.1%	-2.1%	-2.1%	-2.0%	-2.9%	-2.5%	-2.7%	-1.6%	-0.8%	0.0%	-0.0%	-0.1%	-1.0%	-0.1%	-0.1%	-0.2%	-0.0%	-0.2%	-0.3%	-0.8%	-1.3%	-1.9%
High SDI	New Zealand	-0.3%	-0.5%	-0.2%	-0.4%	-2.3%	-2.6%			1.8%	1.7%	-1.6%	-1.7%	-1.4%	-1.8%	-2.3%	-2.9%	-2.3%	-2.6%	-1.3%	-0.7%	0.0%	-0.0%	0.1%	-1.3%	-0.0%	-0.1%	-0.5%	-0.6%	-0.5%	-0.7%	-2.1%	-2.5%	-1.0%
High SDI	Norway	-0.5%	-0.6%	-0.3%	-0.3%	-2.5%	-2.5%			-0.3%	-0.2%	-0.8%	-1.2%	-2.5%	-2.4%	2.9%	-2.7%	-2.1%	-2.4%	-1.2%	-1.3%	-0.0%	-0.0%	-0.8%	1.0%	-0.2%	-0.1%	0.0%	-0.4%	-0.2%	-0.3%	-1.8%	-2.7%	-2.4%
High SDI	Puerto Rico	-0.5%	-0.3%	1.2%	1.3%	-2.1%	-2.2%			0.1%	2.6%	-1.2%	-1.3%	-2.4%	-2.7%	-2.1%	-2.7%	-2.4%	-2.3%	-0.6%	-0.5%	-0.0%	-0.0%	-0.1%	-0.6%	-0.1%	-0.2%	-1.1%	-0.7%	-0.7%	-0.9%	-2.6%	-1.9%	-2.2%
High SDI	Qatar	-1.7%	-1.6%	-1.2%	-0.9%	-1.9%	-2.1%			1.6%	3.2%	-3.2%	-3.2%	-2.1%	-1.6%	1.0%	2.0%	-2.8%	-2.9%	-2.2%	-2.1%	-0.0%	-0.0%	-0.8%	-0.8%	-0.1%	-0.1%	-1.8%	-1.6%	-0.9%	-1.4%	-2.8%	-3.0%	-2.9%
High SDI	Republic of Korea	-1.6%	-1.8%	-1.8%	-2.1%	-3.0%	-3.0%	-3.2%	-3.3%	-0.2%	1.7%	-2.9%	-2.9%	-2.7%	-2.6%	-0.5%	-1.2%	-3.2%	-3.2%	-1.9%	-1.8%	0.0%	-0.0%	-0.8%	-0.2%	0.0%	-0.0%	-1.8%	-1.8%	-2.3%	-2.2%	-2.9%	-2.8%	-3.2%
High SDI	San Marino	-0.3%	-0.4%	-0.3%	-0.2%	-1.5%	-1.9%			-0.3%	0.1%	2.3%	1.8%	-1.6%	-1.3%	-1.7%	-1.9%	-2.5%	-2.7%	-1.3%	-1.2%	0.0%	-0.0%	-0.4%	-0.5%	-0.1%	-0.0%	-0.5%	-0.8%	-1.3%	-1.4%	-1.7%	-1.9%	-1.5%
High SDI	Singapore	-0.8%	-1.0%	-1.2%	-1.8%	-2.2%	-2.1%			5.5%	3.8%	-1.8%	-1.8%	-2.1%	-2.0%	3.8%	2.6%	-2.8%	-3.0%	-1.4%	-1.3%	0.0%	-0.0%	-0.7%	-1.8%	-0.0%	0.0%	-1.7%	-1.5%	-0.7%	-1.2%	-3.0%	-2.9%	-1.8%
High SDI	Slovakia	-1.1%	-1.0%	-0.8%	-0.8%	-2.3%	-2.4%			-0.2%	1.6%	1.6%	1.0%	-1.8%	-1.4%	8.2e+13%	6.1e+13%	-2.6%	-2.7%	-1.1%	-1.2%	0.0%	0.0%	-0.6%	-0.5%	-0.1%	-0.1%	-0.7%	-1.1%	-0.5%	-0.8%	-2.1%	-2.2%	-2.3%
High SDI	Slovenia	-0.5%	-0.6%	-0.7%	-0.7%	-1.6%	-2.1%			-0.6%	1.0%	-0.0%	0.3%	-1.7%	-2.1%	-2.4%	-1.8%	-2.3%	-2.6%	-1.7%	-1.9%	0.1%	0.1%	-0.4%	-1.1%	-0.1%	-0.1%	-0.7%	-0.9%	-0.3%	-0.4%	-2.1%	-1.2%	-2.2%
High SDI	Sweden	-0.2%	-0.4%	-0.3%	-0.3%	-2.0%	-2.2%			-0.6%	-0.9%	0.1%	-0.6%	-1.7%	-1.8%	-0.6%	-2.5%	-2.0%	-2.3%	-1.4%	-0.8%	0.0%	-0.0%	-0.4%	-1.0%	-0.0%	-0.0%	0.2%	-0.1%	-0.4%	-0.4%	-1.8%	-2.1%	-2.8%
High SDI	Switzerland	-0.3%	-0.5%	-0.2%	-0.2%	-2.4%	-2.6%			-2.2%	0.1%	-0.9%	-2.1%	-2.1%	-2.6%	12.4%	6.8%	-2.1%	-2.7%	-1.5%	-1.0%	0.0%	-0.0%	0.0%	-0.5%	-0.1%	-0.0%	-0.4%	-0.6%	-0.7%	0.6%	-2.2%	-1.7%	-1.2%
High SDI	Taiwan (Province of China)	-0.7%	-0.7%	-0.3%	-0.1%	-1.7%	-1.7%			9.1%	7.2%	-2.4%	-2.3%	-1.6%	-1.6%	21.8%	17.9%	-2.1%	-2.2%	-1.6%	-1.6%	0.1%	0.0%	-0.3%	-1.2%	-0.2%	-0.3%	-0.4%	-0.2%	-0.8%	-1.2%	-2.8%	-2.8%	-1.5%
High SDI	United Arab Emirates	-1.5%	-1.6%	-0.9%	-0.7%	-2.2%	-2.4%	-3.3%	-3.3%	1.3%	5.2%	-3.1%	-3.0%	-2.3%	-2.4%	44.9%	44.9%	-2.7%	-2.7%	-1.4%	-1.5%	-0.1%	-0.1%	-0.8%	-0.7%	-0.1%	-0.1%	-1.2%	-1.4%	-1.5%	-1.7%	-2.2%	-2.2%	-2.6%
High SDI	United Kingdom	-0.4%	-0.6%	-0.4%	-0.3%	-1.7%	-1.9%			-0.1%	0.0%	-1.7%	-1.6%	-2.0%	-2.0%	0.7%	-2.4%	-2.1%	-2.4%	-1.4%	-0.9%	-0.0%	-0.0%	-0.0%	-0.9%	-0.1%	-0.1%	-0.9%	-0.7%	-0.0%	0.1%	-2.4%	-2.4%	-1.5%
High SDI	United States of America	-0.3%	-0.4%	0.1%	-0.2%	-1.1%	-1.2%			-0.9%	-1.1%	-1.5%	-1.5%	-1.9%	-1.8%	-2.9%	-2.9%	-2.5%	-2.6%	0.2%	0.2%	-0.0%	-0.0%	-0.2%	-1.3%	-0.1%	-0.1%	-0.5%	-0.5%	-0.2%	-0.2%	-1.9%	-1.8%	-1.2%

S14. (A) Communicable disease DALYs by cause for 15-24 year age group by sex in 2019, grouped by SDI. The shading ranges from green which indicates a low number of DALYs per 100,000 for that country with in the disease, while the highest rates are shaded in a dark orange colour and indicates a country has a large DALY burden.

15 to 24 years, DALYs/100,000, for males and females, 2019

		Total commuincable		Enteric infections		Lower respiratory infections		Malaria		Neonatal sepsis and other neonatal infections		Vaccine Preventable disease		Meningitis & Encephalitis	
		Female	Male	Female	Male	Female	Male	Female	Male	Female	Male	Female	Male	Female	Male
Low SDI	Afghanistan	3,087	2,195	316	321	269	271	240	147	7	11	77	49	238	153
	Benin	5,038	6,084	647	800	402	436	1,730	2,401	37	23	79	86	329	422
	Burkina Faso	5,423	6,176	988	1,090	418	432	1,727	2,027	31	30	49	52	393	464
	Burundi	6,852	8,496	1,052	1,570	607	652	783	656	53	47	49	102	380	498
	Central African Republic	11,669	13,878	1,127	2,189	572	955	1,051	2,378	11	9	95	118	486	700
	Chad	5,871	6,914	912	1,339	462	602	610	1,038	18	21	101	190	442	671
	Côte d'Ivoire	6,333	8,128	544	655	389	479	2,021	3,027	38	43	24	38	284	376
	Dem. Rep. of the Congo	6,068	6,381	413	864	410	537	817	1,002	25	24	105	119	295	323
	Eritrea	5,124	8,212	784	2,136	494	1,041	406	526	53	54	28	63	299	552
	Ethiopia	4,644	5,289	784	960	272	352	135	108	74	76	51	72	323	480
	Gambia	4,449	5,675	464	791	312	602	595	1,088	74	67	20	37	272	637
	Guinea	6,640	6,975	846	1,168	603	672	1,094	1,340	25	24	66	94	514	654
	Guinea-Bissau	6,024	7,153	886	910	550	792	444	696	37	32	189	222	479	774
	Haiti	4,487	2,749	177	212	377	205	377	323	37	27	22	22	116	100
	Liberia	7,710	8,085	730	835	382	365	2,188	2,713	43	44	42	47	355	403
	Madagascar	5,270	6,238	902	1,598	643	664	468	579	48	47	72	76	321	359
	Malawi	6,819	8,019	819	1,334	317	560	766	937	58	56	42	101	360	731
	Mali	4,812	5,383	953	1,429	177	204	748	1,087	34	21	69	97	430	893
	Mozambique	17,208	14,820	467	1,046	345	604	1,422	1,994	48	50	33	64	225	480
	Nepal	2,141	2,528	584	589	106	125	2	1	110	71	39	30	112	117
	Niger	4,785	5,860	875	1,149	411	427	1,048	1,875	27	25	164	188	402	479
	Pakistan	3,638	4,394	1,081	1,466	73	217	76	55	40	28	56	35	192	349
	Papua New Guinea	4,824	5,477	548	857	269	337	724	919	15	22	47	48	108	197
	Rwanda	4,155	4,747	607	847	366	406	368	438	76	69	27	33	255	337
	Senegal	3,934	5,146	572	931	294	426	828	1,428	53	58	33	54	334	545
	Sierra Leone	7,219	8,007	720	1,205	500	525	2,340	3,189	27	23	21	39	407	545
	Solomon Islands	5,928	5,397	840	775	1,291	1,402	386	457	11	20	27	20	193	198
	Somalia	8,259	9,971	1,347	1,514	605	780	333	367	26	22	340	670	581	842
	South Sudan	7,134	7,682	1,087	1,397	325	347	603	608	31	28	72	93	307	438
	Togo	5,900	6,711	726	913	406	518	1,732	2,240	53	43	20	31	293	385
	Uganda	6,474	7,170	580	935	360	473	790	773	62	50	63	72	231	378
	United Republic of Tanzania	5,334	5,946	745	984	334	413	415	313	62	52	41	58	154	255
	Yemen	3,422	3,259	258	269	151	159	1,876	2,103	6	9	16	18	42	50

		HIV/AIDS		Tuberculosis		Neglected Tropical diseases		Infectious skin conditions		Sexually transmitted infections excluding HIV		Upper respiratory infections		Other unspecified infectious diseases		Hepatitis		Rheumatic heart disease		Maternal sepsis and other maternal infections
		Female	Male	Female	Male	Female	Male	Female	Male	Female	Male	Female	Male	Female	Male	Female	Male	Female	Male	Female
Low SDI	Afghanistan	35	36	329	153	587	490	96	100	11	10	122	133	266	93	167	154	134	75	193
	Benin	543	527	334	525	343	341	129	149	33	11	95	134	21	11	69	125	100	92	149
	Burkina Faso	497	619	489	701	175	250	130	142	47	9	103	149	21	20	76	100	102	90	178
	Burundi	760	864	1,589	2,661	483	714	191	229	66	20	112	131	17	15	96	158	174	181	439
	Central African Republic	3,696	2,277	2,556	3,283	732	1,041	127	157	41	16	155	188	41	81	68	259	167	228	743
	Chad	1,062	664	711	1,188	547	542	129	155	41	9	106	145	24	25	94	217	114	108	498
	Côte d'Ivoire	1,735	1,560	443	746	307	675	130	148	37	9	103	138	22	22	54	123	97	89	105
	Dem. Rep. of the Congo	493	302	1,133	1,151	1,333	1,354	122	139	33	14	127	157	39	57	52	184	128	152	544
	Eritrea	764	557	905	2,239	316	246	191	229	60	16	122	143	26	27	69	195	164	188	441
	Ethiopia	1,172	883	507	962	489	613	269	307	50	13	107	126	15	14	83	194	129	130	185
	Gambia	1,335	646	365	937	399	267	127	151	36	8	75	119	35	22	61	206	92	97	187
	Guinea	1,278	537	533	819	831	999	131	155	44	9	98	140	25	19	102	256	108	90	342
	Guinea-Bissau	1,692	1,411	544	1,163	565	473	131	155	45	9	99	145	30	25	93	215	129	129	110
	Haiti	1,501	956	390	179	322	118	194	205	34	15	133	149	41	48	62	33	326	157	377
	Liberia	1,012	551	424	572	1,660	1,968	128	146	43	9	112	159	22	18	79	166	101	91	389
	Madagascar	305	85	1,036	1,588	316	266	187	217	68	17	118	138	200	231	82	129	227	245	278
	Malawi	2,788	1,600	712	1,654	219	242	188	226	51	18	134	157	21	20	64	227	147	156	132
	Mali	704	360	489	429	291	250	164	195	52	9	89	136	32	32	91	138	139	104	350
	Mozambique	12,503	6,535	1,153	2,861	301	393	189	231	77	20	111	134	17	22	56	220	136	168	126
	Nepal	73	102	228	394	343	339	137	194	14	6	122	139	20	12	104	177	112	232	32
	Niger	196	152	413	678	223	318	141	158	40	9	95	148	24	23	90	126	118	104	516
	Pakistan	40	56	927	769	145	137	153	184	8	4	118	135	45	303	251	243	345	414	86
	Papua New Guinea	1,030	390	495	503	359	791	322	345	25	8	106	117	69	273	48	83	475	587	186
	Rwanda	819	554	525	966	369	414	167	198	57	17	123	139	23	17	78	174	137	136	159
	Senegal	346	200	329	657	549	327	129	150	36	6	97	138	37	36	51	100	92	90	155
	Sierra Leone	1,508	668	501	611	413	671	127	147	41	9	90	134	18	21	75	121	113	97	320
	Solomon Islands	259	136	219	183	949	634	325	349	21	8	111	124	358	352	133	191	573	547	230
	Somalia	642	424	2,040	3,624	671	608	191	230	84	28	165	202	40	35	135	394	200	232	859
	South Sudan	1,655	642	737	1,343	1,561	2,105	194	226	61	18	109	121	24	27	78	164	134	125	157
	Togo	1,412	1,149	433	643	256	244	129	148	33	9	108	150	23	18	62	126	98	96	116
	Uganda	2,711	1,962	626	1,454	299	361	190	223	58	23	138	156	17	17	61	128	151	166	138
	United Republic of Tanzania	1,790	1,648	620	1,166	439	355	218	256	57	19	124	144	21	32	53	116	133	134	128
	Yemen	41	44	63	50	358	157	94	98	10	10	135	148	50	19	40	54	80	72	202

15 to 24 years, DALYs/100,000, for males and females, 2019

Low-middle SDI

Country	Total communicable Female	Total communicable Male	Enteric infections Female	Enteric infections Male	Lower respiratory infections Female	Lower respiratory infections Male	Malaria Female	Malaria Male	Neonatal sepsis and other neonatal infections Female	Neonatal sepsis and other neonatal infections Male	Vaccine Preventable disease Female	Vaccine Preventable disease Male	Meningitis & Encephalitis Female	Meningitis & Encephalitis Male
Angola	5,926	5,299	565	815	301	485	700	874	27	32	43	50	280	354
Bangladesh	2,008	2,050	625	595	111	119	5	4	176	220	20	13	95	68
Belize	1,531	1,646	99	139	161	270	1	0	142	76	3	5	44	63
Bhutan	1,636	2,107	474	589	62	96	4	2	100	78	6	6	130	201
Bolivia	1,574	1,797	138	169	213	259	2	1	87	84	7	9	50	65
Cabo Verde	1,529	2,091	250	328	168	342	81	90	100	88	5	7	99	247
Cambodia	2,370	2,963	289	360	331	615	38	43	84	106	18	32	46	69
Cameroon	6,930	7,069	544	690	378	475	1,437	2,180	42	41	52	60	303	413
Comoros	4,778	4,738	698	776	431	341	1,682	1,434	81	72	58	56	330	307
Congo	7,002	6,481	324	658	301	348	1,102	1,125	35	38	40	40	227	247
Dem. People's Rep. of Korea	1,099	1,341	107	109	110	184	2	1	13	15	6	7	61	82
Djibouti	4,791	5,157	424	790	254	456	526	573	68	64	31	58	204	372
Dominican Republic	1,763	1,807	108	150	121	163	6	5	302	253	9	12	56	80
El Salvador	876	1,145	79	127	117	238	0	1	33	25	5	5	30	50
Eswatini	14,409	10,470	629	1,072	554	915	35	35	32	28	17	21	200	378
Ghana	5,462	6,506	555	638	251	720	1,484	1,804	93	107	27	32	452	612
Guatemala	1,539	1,780	246	343	392	521	3	0	27	23	5	6	70	87
Honduras	1,145	1,039	121	135	32	81	2	1	85	69	7	6	57	53
India	2,965	2,781	954	861	168	121	71	56	44	42	19	16	200	178
Kenya	6,938	7,728	842	1,316	307	462	299	345	87	69	144	229	347	436
Kiribati	5,102	6,069	551	755	533	740	0	0	8	17	56	49	511	648
Kyrgyzstan	1,012	993	182	171	96	119	0	0	12	13	1	1	69	82
Lao PDR	2,382	2,553	309	387	261	307	42	36	71	95	40	56	95	125
Lesotho	20,783	14,120	873	1,631	749	1,165	0	0	32	29	14	20	254	429
Maldives	947	1,167	184	272	37	38	0	0	81	143	7	7	28	35
Marshall Islands	3,167	3,009	290	279	666	694	0	0	14	22	36	30	118	142
Mauritania	2,863	3,279	436	601	242	247	952	1,316	95	96	19	26	200	212
Micronesia (Fed States of)	2,948	3,191	277	314	646	807	0	0	13	25	18	17	108	151
Mongolia	1,178	1,364	204	188	140	150	0	0	12	16	5	4	65	97
Morocco	1,130	843	172	190	122	111	0	0	8	14	16	15	38	41
Myanmar	2,312	3,041	331	411	150	346	45	47	93	110	28	41	63	63
Nicaragua	966	1,167	64	116	84	150	9	5	43	36	3	3	44	75
Nigeria	5,235	5,687	870	901	204	257	1,567	2,042	47	50	36	60	256	383
Palestine	664	756	172	172	99	182	0	0	17	19	4	4	26	33
Sao Tome and Principe	2,111	2,717	297	351	434	699	356	263	66	86	34	48	186	155
Sudan	1,727	2,186	254	315	166	180	165	753	6	9	19	20	42	57
Tajikistan	1,455	1,563	272	242	345	420	2	2	18	21	6	6	74	75
Timor-Leste	2,302	2,789	279	419	212	291	4	4	65	79	25	39	88	117
Tuvalu	2,267	2,456	226	270	512	580	0	0	18	31	27	23	92	127
Vanuatu	3,294	3,434	418	468	581	737	28	24	11	20	36	34	107	158
Venezuela	1,643	1,522	87	133	172	158	284	204	249	188	6	6	72	66
Zambia	9,041	9,335	798	985	399	545	619	717	71	65	33	63	417	672
Zimbabwe	8,762	9,818	463	1,211	431	1,119	191	227	37	40	33	41	341	577

Country	HIV/AIDS Female	HIV/AIDS Male	Tuberculosis Female	Tuberculosis Male	Neglected Tropical diseases Female	Neglected Tropical diseases Male	Infectious skin conditions Female	Infectious skin conditions Male	Sexually transmitted infections excluding HIV Female	Sexually transmitted infections excluding HIV Male	Upper respiratory infections Female	Upper respiratory infections Male	Other unspecified infectious diseases Female	Other unspecified infectious diseases Male	Hepatitis Female	Hepatitis Male	Rheumatic heart disease Female	Rheumatic heart disease Male	Maternal sepsis and other maternal infections Female
Angola	2,265	565	796	997	348	419	123	140	33	14	121	141	46	65	56	208	121	140	101
Bangladesh	6	5	258	292	169	159	149	184	10	7	136	152	20	23	99	98	120	109	11
Belize	439	422	71	141	86	59	188	198	17	12	112	123	24	19	19	34	91	86	33
Bhutan	76	123	175	215	101	81	150	180	13	6	107	122	44	34	61	159	121	215	12
Bolivia	135	177	276	377	138	171	211	219	20	7	110	119	18	39	16	32	82	69	71
Cabo Verde	256	283	117	132	82	80	120	140	29	10	91	121	22	10	31	134	70	81	8
Cambodia	191	189	547	581	98	61	248	259	11	15	100	119	51	83	165	326	117	106	35
Cameroon	2,674	1,486	389	610	508	576	125	140	45	13	116	155	20	14	53	128	88	86	154
Comoros	7	10	716	885	114	244	192	218	61	15	125	141	34	21	73	99	143	121	34
Congo	3,455	2,395	633	688	314	352	124	140	30	13	115	137	38	48	47	126	118	126	100
Dem. People's Rep. of Korea	38	63	177	311	51	13	260	264	8	11	104	120	22	12	28	74	74	76	37
Djibouti	1,948	1,003	398	1,061	73	138	187	219	54	16	115	132	24	20	44	132	121	123	318
Dominican Republic	214	191	195	272	248	189	184	189	27	24	103	108	16	19	30	69	110	84	33
El Salvador	108	186	16	32	99	68	155	167	18	9	112	124	8	17	13	36	66	58	18
Eswatini	10,992	5,383	1,208	1,825	228	186	128	140	43	20	103	125	32	54	33	134	137	155	40
Ghana	1,503	950	293	836	343	340	93	110	40	15	94	127	23	15	82	126	90	76	41
Guatemala	99	138	62	84	195	121	161	178	27	8	105	117	15	19	25	78	69	56	41
Honduras	41	42	82	60	271	165	154	166	20	9	118	132	25	28	27	43	64	51	40
India	116	75	443	407	185	242	178	180	16	10	112	129	68	51	148	218	195	194	46
Kenya	3,396	2,576	516	1,223	283	352	201	215	43	14	147	170	19	18	94	181	117	124	94
Kiribati	49	26	1,714	1,611	308	318	325	356	24	8	111	124	187	344	147	279	487	794	92
Kyrgyzstan	30	16	150	158	98	41	78	84	13	14	89	103	24	8	49	89	97	94	25
Lao PDR	72	155	488	479	318	221	251	260	12	15	111	128	27	38	46	90	201	160	39
Lesotho	15,826	7,283	2,280	2,857	69	48	128	140	45	21	114	150	32	55	41	119	156	172	169
Maldives	14	2	29	27	111	181	247	250	10	16	101	118	24	12	12	22	57	45	6
Marshall Islands	244	135	425	299	230	151	323	344	19	8	105	116	55	100	61	126	533	563	48
Mauritania	8	13	188	205	196	106	128	143	35	9	95	129	24	16	50	90	79	71	118
Micronesia (Fed States of)	360	262	315	234	175	142	321	345	20	8	106	118	56	110	53	125	436	532	44
Mongolia	4	8	259	410	62	38	79	86	15	15	84	95	18	17	108	163	87	78	35
Morocco	59	41	296	53	77	42	94	97	12	12	115	129	22	11	24	36	57	50	17
Myanmar	334	202	353	423	194	288	252	260	12	16	111	130	170	428	45	182	78	94	53
Nicaragua	183	242	39	56	100	66	158	170	18	9	106	118	7	13	14	41	77	66	19
Nigeria	975	408	251	486	471	526	172	203	30	8	93	123	33	25	62	131	87	84	82
Palestine	12	13	14	10	21	13	95	96	12	10	115	125	10	13	19	31	36	34	11
Sao Tome and Principe	3	5	126	233	139	238	127	145	34	9	96	125	29	69	43	176	117	115	25
Sudan	361	110	56	68	158	265	95	100	16	10	115	127	22	20	71	90	70	64	110
Tajikistan	17	3	256	298	73	51	76	80	18	26	74	92	16	10	81	135	112	103	16
Timor-Leste	480	483	344	373	117	341	270	279	11	15	105	122	22	32	38	68	144	126	98
Tuvalu	147	159	220	133	133	94	320	341	19	8	105	117	59	94	50	117	311	364	29
Vanuatu	265	134	376	356	343	173	302	325	19	8	107	119	66	104	67	141	501	634	66
Venezuela	102	213	69	43	212	170	152	163	23	8	110	123	16	16	17	19	17	10	56
Zambia	4,901	3,435	715	1,563	356	419	191	225	71	16	132	151	21	61	85	266	146	152	84
Zimbabwe	4,675	3,162	1,370	2,306	235	200	128	139	45	19	124	154	20	41	40	118	309	465	320

15 to 24 years, DALYs/100,000, for males and females, 2019

		Total commuincable		Enteric infections		Lower respiratory infections		Malaria		Neonatal sepsis and other neonatal infections		Vaccine Preventable disease		Meningitis & Encephalitis	
		Female	Male	Female	Male	Female	Male	Female	Male	Female	Male	Female	Male	Female	Male
Middle SDI	Albania	708	762	271	217	54	108	0	0	6	6	3	3	34	55
	Algeria	725	684	160	156	118	110	1	1	13	18	6	6	32	36
	Armenia	837	857	250	219	53	120	0	0	117	62	1	1	29	43
	Azerbaijan	1,041	1,309	239	215	175	265	0	0	21	24	6	6	68	98
	Botswana	9,278	7,381	349	554	660	813	32	28	43	57	12	13	186	260
	Brazil	1,240	1,281	112	103	130	199	8	7	80	58	3	5	35	54
	China	771	894	58	58	28	47	0	0	133	126	3	3	24	32
	Colombia	887	1,101	48	92	86	129	10	9	147	148	4	5	43	62
	Costa Rica	712	819	43	84	35	77	0	0	56	28	2	2	43	63
	Cuba	753	861	69	105	51	66	0	0	85	61	3	4	36	43
	Ecuador	1,167	1,577	130	140	165	302	3	1	53	49	6	8	40	58
	Egypt	822	1,061	159	163	161	295	0	0	7	9	8	7	52	75
	Equatorial Guinea	9,921	5,883	187	326	202	307	1,425	1,582	48	50	27	31	148	199
	Fiji	2,122	2,712	225	286	367	382	0	0	40	52	11	10	81	140
	Gabon	5,004	4,701	166	368	168	414	1,238	1,286	74	68	18	21	150	279
	Grenada	1,170	1,149	113	150	186	191	0	0	145	130	5	4	41	39
	Guyana	2,239	2,860	133	185	220	301	214	186	82	128	3	4	72	104
	Indonesia	1,967	2,751	394	505	69	113	29	22	71	113	17	30	77	130
	Iran (Islamic Republic of)	710	793	197	195	96	134	1	1	11	38	4	4	35	38
	Iraq	878	805	160	163	101	92	0	0	21	27	11	10	67	65
	Jamaica	1,305	1,140	72	96	70	65	0	0	238	185	2	2	47	43
	Mexico	812	1,003	50	79	88	148	0	0	102	76	4	4	39	50
	Namibia	7,790	7,528	365	735	396	734	269	243	44	43	8	10	126	231
	Nauru	2,465	2,964	204	240	635	906	0	0	20	30	6	6	90	141
	Panama	1,350	1,903	102	149	103	138	2	0	176	117	2	2	38	59
	Paraguay	1,059	1,163	118	130	103	163	0	0	14	28	5	5	41	65
	Peru	1,516	1,814	124	157	208	306	11	6	268	272	6	6	47	67
	Philippines	2,310	2,915	292	338	294	380	2	3	152	144	27	37	107	135
	Saint Lucia	1,084	1,154	116	156	74	135	0	0	170	90	3	8	50	80
	Samoa	2,082	2,173	203	252	410	438	0	0	15	26	57	43	80	101
	South Africa	12,815	7,137	359	347	325	418	33	18	49	55	19	17	103	138
	St Vincent & the Grenadines	1,332	1,422	105	136	110	136	0	0	128	123	4	3	42	78
	Suriname	1,884	1,582	115	158	122	144	12	8	178	163	5	7	66	94
	Syrian Arab Republic	1,003	997	175	173	179	230	0	0	6	8	9	9	82	83
	Thailand	1,485	1,792	190	220	144	225	2	1	102	88	7	9	63	90
	Tokelau	2,001	1,830	197	238	435	319	0	0	20	38	24	19	83	85
	Tonga	1,831	2,351	168	248	225	354	0	0	57	57	18	18	170	263
	Tunisia	641	662	152	151	73	86	0	0	12	15	6	5	28	32
	Turkmenistan	1,428	1,603	169	156	269	357	0	0	19	20	1	2	123	131
	Uzbekistan	1,455	1,483	164	151	383	449	0	0	23	30	1	1	115	137
	Viet Nam	1,220	1,933	205	242	49	166	7	7	124	125	10	11	57	120

		HIV/AIDS		Tuberculosis		Neglected Tropical diseases		Infectious skin conditions		Sexually transmitted infections excluding HIV		Upper respiratory infections		Other unspecified infectious diseases		Hepatitis		Rheumatic heart disease		Maternal sepsis and other maternal infections
		Female	Male	Female	Male	Female	Male	Female	Male	Female	Male	Female	Male	Female	Male	Female	Male	Female	Male	Female
Middle SDI	Albania	2	4	2	3	8	10	112	116	10	6	105	109	20	30	16	34	58	64	9
	Algeria	15	13	21	13	38	16	94	97	10	10	108	118	21	14	26	40	39	36	22
	Armenia	4	8	29	45	36	32	76	80	13	14	116	124	9	10	15	31	78	68	12
	Azerbaijan	7	6	134	295	56	32	76	80	15	15	80	92	19	8	38	79	94	95	13
	Botswana	6,285	4,085	921	924	282	155	124	135	40	17	102	123	34	43	26	54	124	119	59
	Brazil	129	184	39	51	167	118	215	213	21	9	134	146	35	32	15	28	86	74	30
	China	26	45	36	54	49	57	249	265	7	9	95	106	7	7	18	48	35	35	3
	Colombia	74	175	31	32	76	77	158	174	22	9	133	147	12	21	11	16	6	5	24
	Costa Rica	65	97	8	12	72	42	151	167	18	9	108	120	11	18	14	29	78	71	8
	Cuba	21	120	4	4	40	18	185	200	25	12	113	121	18	22	10	14	82	71	11
	Ecuador	112	272	86	184	97	102	211	221	18	7	114	123	13	16	13	27	73	67	32
	Egypt	4	4	24	30	51	56	105	108	12	11	95	107	15	14	45	115	68	67	15
	Equatorial Guinea	6,754	2,126	312	370	359	378	120	133	29	13	112	127	32	44	31	93	96	102	40
	Fiji	63	28	145	78	217	511	348	362	20	8	99	109	140	322	40	59	305	365	21
	Gabon	1,909	553	300	547	497	556	122	139	27	13	116	136	44	56	35	150	99	115	41
	Grenada	102	51	18	16	71	41	201	221	18	15	109	117	23	45	14	23	118	106	7
	Guyana	676	355	153	233	144	825	185	193	32	16	113	119	27	49	27	51	127	109	32
	Indonesia	108	153	454	710	185	293	260	270	7	18	127	143	23	21	101	213	29	19	17
	Iran (Islamic Republic of)	27	27	17	15	12	9	94	97	9	10	114	125	15	17	23	35	47	49	8
	Iraq	12	5	60	37	151	109	85	89	12	10	107	117	12	8	26	33	44	40	9
	Jamaica	280	203	10	13	92	54	184	190	18	13	112	120	30	47	16	16	127	93	8
	Mexico	55	165	36	43	72	50	160	175	18	7	118	131	12	15	13	34	30	26	15
	Namibia	4,772	3,580	834	1,140	501	317	125	135	39	20	107	129	28	42	23	52	109	118	45
	Nauru	143	155	229	197	170	143	319	342	19	8	99	108	51	104	51	127	398	457	33
	Panama	260	895	130	109	117	41	151	166	20	8	107	117	22	15	13	23	78	64	29
	Paraguay	160	142	50	93	79	66	209	213	20	10	126	136	23	19	13	25	76	69	24
	Peru	119	184	152	221	102	116	218	228	19	7	122	130	15	20	15	36	66	57	23
	Philippines	179	368	397	528	229	302	256	266	12	14	127	147	18	47	25	56	174	149	18
	Saint Lucia	94	77	30	59	81	49	191	206	22	15	109	119	26	53	12	22	99	84	8
	Samoa	239	140	176	115	112	154	317	334	18	8	103	112	44	75	38	87	259	287	12
	South Africa	10,253	4,688	957	815	236	128	126	136	35	25	110	131	60	81	16	39	111	100	25
	St Vincent & the Grenadines	360	306	26	56	80	40	195	206	17	20	112	121	29	68	13	21	103	109	9
	Suriname	533	243	37	45	314	191	202	219	20	14	109	117	34	70	19	29	95	80	23
	Syrian Arab Republic	4	7	18	14	193	134	94	96	11	9	113	124	26	14	26	44	57	52	8
	Thailand	385	473	43	43	59	59	251	260	11	14	143	162	20	57	17	53	42	37	6
	Tokelau	158	164	143	56	136	95	319	336	19	8	100	111	48	61	53	99	238	201	28
	Tonga	50	49	86	73	278	281	318	340	19	8	103	114	95	180	98	219	125	148	23
	Tunisia	51	60	14	10	38	27	94	97	12	10	110	119	16	12	19	30	9	10	7
	Turkmenistan	10	12	288	293	49	30	78	80	16	13	81	93	20	15	119	276	171	124	12
	Uzbekistan	20	20	171	141	95	48	77	81	14	14	81	88	37	11	81	167	167	145	24
	Viet Nam	63	414	147	200	135	169	250	261	8	15	95	109	18	14	27	68	20	12	5

15 to 24 years, DALYs/100,000, for males and females, 2019

High-middle SDI

	Total communicable		Enteric infections		Lower respiratory infections		Malaria		Neonatal sepsis and other neonatal infections		Vaccine Preventable disease		Meningitis & Encephalitis	
	Female	Male	Female	Male	Female	Male	Female	Male	Female	Male	Female	Male	Female	Male
American Samoa	1,442	1,866	165	238	259	297	0	0	5	11	18	14	81	101
Antigua and Barbuda	952	1,152	84	115	76	133	0	0	11	78	5	3	32	54
Argentina	861	882	73	94	174	224	0	0	6	5	3	3	38	48
Bahamas	1,459	1,395	81	110	121	182	0	0	136	62	2	5	47	68
Bahrain	555	579	160	154	58	75	0	0	5	5	3	3	15	31
Barbados	1,333	1,228	97	125	94	116	0	0	273	205	5	5	61	76
Belarus	591	606	180	152	31	65	0	0	63	43	1	1	45	52
Bosnia and Herzegovina	592	637	239	225	36	58	0	0	20	22	5	5	19	26
Bulgaria	700	796	198	185	118	192	0	0	19	18	1	1	46	55
Chile	539	542	81	80	32	37	0	0	4	9	3	3	28	32
Cook Islands	1,187	1,556	130	186	221	446	0	0	27	51	6	5	18	25
Croatia	485	517	154	147	12	24	0	0	48	69	1	1	14	14
Dominica	1,477	1,373	92	120	127	156	0	0	232	149	7	8	67	98
Georgia	859	1,040	234	214	97	165	0	0	30	41	1	1	38	57
Greece	367	355	40	43	34	52	0	0	2	2	2	2	15	18
Greenland	553	508	82	105	30	23	0	0	2	3	6	5	21	35
Hungary	588	570	256	218	38	49	0	0	15	22	1	1	14	16
Israel	340	343	42	43	21	36	0	0	2	3	2	2	13	15
Italy	355	323	25	27	13	18	0	0	6	6	2	2	15	17
Jordan	578	539	148	141	89	80	0	0	18	23	8	6	34	36
Kazakhstan	826	822	161	144	111	141	0	0	20	23	1	1	60	62
Lebanon	716	817	168	173	72	119	0	0	14	17	28	18	30	37
Libya	878	807	189	185	106	116	0	0	5	7	6	5	29	33
Malaysia	1,286	1,546	166	183	178	283	4	2	52	75	7	8	61	85
Malta	381	361	31	33	42	49	0	0	4	4	2	2	22	24
Mauritius	1,241	1,603	70	75	69	150	0	0	61	217	2	2	32	69
Montenegro	556	551	172	148	51	61	0	0	34	25	3	3	17	21
Niue	1,641	2,096	164	234	322	492	0	0	27	41	8	8	50	79
North Macedonia	661	624	248	190	50	65	0	0	49	44	5	5	22	24
Northern Mariana Islands	1,325	1,489	140	194	169	285	0	0	8	20	10	8	44	60
Oman	758	747	182	166	123	132	0	0	10	13	4	4	64	70
Palau	2,251	2,814	262	360	918	1,262	0	0	27	44	7	7	70	21
Poland	494	535	135	125	63	100	0	0	5	6	1	1	18	22
Portugal	433	436	34	37	45	65	0	0	3	5	2	2	19	25
Republic of Moldova	997	1,090	198	176	71	164	0	0	303	289	1	1	40	53
Romania	849	925	234	190	185	278	0	0	18	8	1	1	33	36
Russian Federation	926	933	232	196	99	153	0	0	56	37	2	1	36	46
Saint Kitts and Nevis	1,324	1,612	86	128	89	164	0	0	63	50	3	4	56	55
Saudi Arabia	811	654	146	129	180	111	1	1	19	22	4	4	36	27
Serbia	503	549	163	173	32	55	0	0	18	16	3	3	20	22
Seychelles	1,393	1,868	203	222	257	417	0	0	149	213	10	10	47	98
Spain	371	338	39	42	17	22	0	0	7	7	3	2	20	19
Sri Lanka	1,107	1,284	222	237	111	169	0	0	100	120	6	6	64	82
Trinidad and Tobago	1,206	1,272	68	97	90	153	0	0	84	95	2	2	45	73
Turkey	630	629	158	157	64	84	0	0	48	35	5	5	20	22
Ukraine	890	1,190	165	161	113	255	0	0	40	42	2	1	65	91
United States Virgin Islands	747	793	89	113	23	52	0	0	64	51	6	6	18	26
Uruguay	611	625	54	68	78	82	0	0	11	19	2	2	34	26

	HIV/AIDS		Tuberculosis		Neglected Tropical diseases		Infectious skin conditions		Sexually transmitted infections excluding HIV		Upper respiratory infections		Other unspecified infectious diseases		Hepatitis		Rheumatic heart disease		Maternal sepsis and other maternal infections
	Female	Male	Female	Male	Female	Male	Female	Male	Female	Male	Female	Male	Female	Male	Female	Male	Female	Male	Female
American Samoa	24	22	38	23	117	185	317	392	18	8	96	105	42	173	43	89	199	208	19
Antigua and Barbuda	145	197	17	14	138	95	185	205	16	15	106	113	20	37	11	22	86	70	21
Argentina	68	92	41	39	35	35	173	136	17	6	122	114	13	15	14	23	54	49	29
Bahamas	528	431	35	69	51	23	190	194	20	13	107	111	28	25	17	28	87	75	10
Bahrain	8	13	22	15	18	2	97	105	10	10	102	110	19	10	16	35	16	12	5
Barbados	253	144	13	11	58	49	206	229	20	16	108	115	17	49	13	18	92	69	23
Belarus	23	21	19	30	8	6	61	65	10	5	110	119	10	8	16	29	6	9	7
Bosnia and Herzegovina	2	2	7	15	10	9	111	115	10	6	104	109	11	13	12	27	2	5	4
Bulgaria	22	27	6	7	8	9	115	118	10	5	101	104	13	15	22	44	13	16	7
Chile	18	67	18	16	11	8	170	130	13	4	122	114	11	15	11	18	7	8	8
Cook Islands	143	151	26	20	110	90	313	324	19	7	93	101	16	12	16	40	43	98	5
Croatia	2	6	4	3	8	6	111	115	10	5	100	104	8	8	8	13	2	3	3
Dominica	200	179	74	115	78	43	209	216	17	21	115	119	32	42	20	21	127	87	82
Georgia	6	13	74	126	34	24	77	80	14	14	84	96	15	14	18	56	119	139	18
Greece	4	8	3	4	5	6	127	93	5	1	114	108	6	6	8	11	2	1	2
Greenland	27	25	8	8	9	1	183	161	14	3	133	115	15	9	13	13	5	2	5
Hungary	2	3	2	2	8	5	112	115	10	5	101	104	11	11	10	15	3	3	4
Israel	6	12	2	3	3	3	107	94	5	1	113	106	9	10	6	9	5	6	5
Italy	11	12	2	2	4	1	127	94	7	2	121	115	8	8	8	13	5	7	1
Jordan	7	6	4	3	33	13	94	98	9	8	86	95	14	6	13	21	6	4	15
Kazakhstan	14	12	107	98	74	50	77	80	16	14	97	108	25	14	30	55	20	19	12
Lebanon	44	31	15	15	14	13	94	97	9	10	107	116	70	102	30	49	10	20	10
Libya	58	28	20	18	146	88	94	97	9	10	108	118	26	15	27	39	53	49	3
Malaysia	90	93	83	76	175	194	246	254	10	15	102	116	32	71	18	43	55	48	6
Malta	6	8	2	3	6	12	127	95	5	1	113	106	8	11	6	8	4	4	3
Mauritius	68	119	17	11	462	466	243	249	10	13	104	118	15	12	18	47	67	56	7
Montenegro	10	13	5	5	10	15	111	114	10	6	102	106	9	9	14	18	5	6	4
Niue	139	145	76	56	185	179	315	337	18	7	97	107	39	73	32	85	156	252	12
North Macedonia	3	2	13	13	10	9	111	115	10	6	103	107	11	9	16	22	5	12	4
Northern Mariana Islands	62	34	60	26	162	129	329	342	20	7	93	101	50	80	46	109	122	92	9
Oman	25	19	16	13	45	27	99	118	10	10	102	110	36	27	31	37	4	2	5
Palau	127	145	83	52	163	127	310	321	18	8	97	105	36	40	28	142	100	178	6
Poland	6	10	4	4	8	7	109	112	9	5	106	109	11	12	10	17	4	6	4
Portugal	44	50	6	7	12	18	124	91	5	2	111	105	11	11	8	12	5	4	2
Republic of Moldova	61	28	54	98	16	6	73	80	12	5	116	126	18	9	14	32	14	22	7
Romania	57	74	43	46	10	9	114	118	10	5	100	104	12	14	17	31	8	11	8
Russian Federation	176	144	68	84	11	10	80	88	10	6	115	125	8	8	17	29	7	8	8
Saint Kitts and Nevis	538	709	23	41	48	29	186	215	21	15	106	118	23	40	13	21	26	24	43
Saudi Arabia	41	30	99	49	26	22	94	97	11	10	101	109	8	6	22	29	19	9	4
Serbia	4	11	6	4	9	8	111	116	10	6	103	107	9	10	12	16	1	2	3
Seychelles	101	49	32	30	125	132	244	251	13	16	102	115	31	210	19	51	51	53	8
Spain	12	16	3	2	5	3	127	92	4	2	112	106	10	9	7	11	3	3	2
Sri Lanka	7	12	48	40	122	127	241	245	14	14	106	122	28	55	15	40	17	15	5
Trinidad and Tobago	390	292	9	14	65	61	183	203	16	12	105	111	38	54	15	21	90	83	7
Turkey	9	11	20	20	20	5	94	97	8	9	129	135	27	26	13	22	4	3	9
Ukraine	110	63	131	250	11	13	64	71	11	5	119	132	7	9	32	76	12	21	8
United States Virgin Islands	142	108	12	15	40	34	183	203	17	17	103	108	20	25	11	17	13	18	6
Uruguay	51	116	19	21	9	8	171	133	15	5	122	114	9	9	11	15	9	9	16

15 to 24 years, DALYs/100,000, for males and females, 2019

SDI	Country	Total commuincable		Enteric infections		Lower respiratory infections		Malaria		Neonatal sepsis and other neonatal infections		Vaccine Preventable disease		Meningitis & Encephalitis	
		Female	Male	Female	Male	Female	Male	Female	Male	Female	Male	Female	Male	Female	Male
High SDI	Andorra	411	388	37	40	22	19	0	0	3	3	3	3	18	13
	Australia	426	366	37	43	14	13	0	0	2	2	2	2	14	15
	Austria	347	318	40	41	12	14	0	0	3	3	2	2	15	17
	Belgium	378	338	51	52	22	23	0	0	5	3	2	2	22	23
	Bermuda	666	734	71	101	28	45	0	0	20	3	2	2	31	23
	Brunei Darussalam	741	680	11	10	149	127	0	0	6	7	5	4	37	35
	Canada	521	480	72	118	20	23	0	0	9	7	2	2	14	13
	Cyprus	343	307	48	50	7	15	0	0	2	2	3	3	6	9
	Czechia	535	559	193	170	51	80	0	0	24	24	1	1	15	23
	Denmark	368	317	48	52	13	15	0	0	2	2	2	2	18	20
	Estonia	633	638	245	172	54	77	0	0	12	14	1	1	20	23
	Finland	332	310	37	40	9	11	0	0	3	3	2	2	18	16
	France	341	309	31	36	13	15	0	0	4	5	3	3	15	16
	Germany	392	342	47	48	19	26	0	0	3	3	2	2	14	15
	Guam	1,277	1,432	164	208	210	206	0	0	28	64	9	8	46	62
	Iceland	372	328	48	48	35	31	0	0	3	2	2	2	22	23
	Ireland	336	299	32	34	16	18	0	0	3	3	2	2	18	17
	Japan	398	343	4	5	23	31	0	0	3	3	2	2	13	14
	Kuwait	503	531	122	125	73	112	0	0	5	6	2	2	17	20
	Latvia	604	630	212	208	63	91	0	0	16	16	1	2	23	30
	Lithuania	607	584	234	188	65	75	0	0	25	20	1	1	24	30
	Luxembourg	375	342	50	51	21	27	0	0	2	2	2	3	17	17
	Monaco	377	353	36	39	38	47	0	0	3	3	3	3	10	21
	Netherlands	334	314	31	35	18	16	0	0	9	13	2	2	19	19
	New Zealand	485	419	68	60	11	13	0	0	6	5	1	1	24	23
	Norway	361	308	35	42	11	11	0	0	3	3	2	2	18	25
	Puerto Rico	817	870	74	93	49	76	0	0	150	132	2	2	22	22
	Qatar	511	446	115	100	84	43	0	0	3	3	4	3	27	19
	Republic of Korea	428	386	9	9	16	19	0	0	4	7	4	3	17	17
	San Marino	410	349	37	39	20	21	0	0	3	3	3	3	28	25
	Singapore	451	438	7	7	60	98	0	0	8	8	3	2	19	23
	Slovakia	571	631	189	225	89	109	0	0	10	10	3	2	20	19
	Slovenia	487	472	170	157	20	22	0	0	33	22	1	1	13	16
	Sweden	358	311	39	42	10	15	0	0	4	4	2	2	12	13
	Switzerland	329	296	36	38	9	12	0	0	4	7	2	2	10	12
	Taiwan (Province of China)	610	651	61	63	36	61	0	0	8	6	2	2	14	18
	United Arab Emirates	652	663	170	158	68	106	0	0	4	5	7	7	28	41
	United Kingdom	388	351	34	37	31	38	0	0	3	3	3	3	27	29
	United States of America	532	501	88	76	34	38	0	0	6	7	2	2	13	15

SDI	Country	HIV/AIDS		Tuberculosis		Neglected Tropical diseases		Infectious skin conditions		Sexually transmitted infections excluding HIV		Upper respiratory infections		Other unspecified infectious diseases		Hepatitis		Rheumatic heart disease		Maternal sepsis and other maternal infections
		Female	Male	Female	Male	Female	Male	Female	Male	Female	Male	Female	Male	Female	Male	Female	Male	Female	Male	Female
High SDI	Andorra	47	81	2	1	2	1	127	93	5	2	110	104	12	8	20	17	3	1	1
	Australia	4	4	2	2	5	3	186	148	14	3	116	105	14	10	8	11	6	6	2
	Austria	6	14	2	3	4	3	128	95	5	1	109	104	9	10	6	8	3	3	3
	Belgium	10	11	2	3	1	1	126	93	4	1	111	105	11	12	7	8	2	2	2
	Bermuda	142	186	13	4	28	20	181	190	15	12	103	108	14	12	9	14	9	15	2
	Brunei Darussalam	26	53	70	33	49	86	170	131	17	3	118	109	38	39	28	34	15	9	2
	Canada	14	11	2	2	3	0	214	161	13	2	135	118	13	10	7	9	3	3	1
	Cyprus	15	8	1	2	1	2	126	93	5	2	113	106	8	6	6	7	1	3	1
	Czechia	4	4	2	2	3	4	111	116	9	5	98	103	9	11	9	14	2	2	3
	Denmark	9	6	1	2	2	1	138	95	5	1	111	105	9	9	6	7	1	1	2
	Estonia	81	116	14	15	8	4	59	65	10	5	105	113	9	9	10	19	2	4	4
	Finland	1	2	1	2	2	1	128	109	5	1	110	104	8	9	6	8	1	1	2
	France	7	8	2	2	1	1	126	93	5	1	112	106	9	11	6	9	5	3	3
	Germany	6	9	2	3	3	1	161	109	5	2	110	104	10	9	5	8	3	3	3
	Guam	50	135	71	33	46	18	324	336	19	7	90	98	32	28	38	105	143	124	9
	Iceland	2	8	2	2	1	1	126	92	5	1	111	104	9	7	4	5	2	2	2
	Ireland	3	5	2	2	1	1	126	93	5	1	110	103	9	11	6	8	2	1	2
	Japan	2	3	3	2	8	2	169	131	13	2	129	121	17	11	9	14	2	3	1
	Kuwait	1	1	17	8	23	7	93	98	9	9	102	109	21	7	10	18	5	10	3
	Latvia	52	32	17	21	7	7	64	72	10	5	107	114	10	9	12	17	5	7	5
	Lithuania	14	18	27	27	9	5	66	71	10	5	104	112	10	8	10	18	4	5	4
	Luxembourg	8	10	2	2	2	1	126	93	5	2	109	102	20	21	7	9	2	3	2
	Monaco	23	19	4	5	4	2	127	93	5	2	104	98	7	6	8	13	2	2	2
	Netherlands	7	5	2	2	1	1	111	94	5	2	111	105	10	12	6	8	1	1	2
	New Zealand	6	5	3	2	3	1	186	151	13	2	125	115	11	10	8	13	14	17	6
	Norway	12	15	1	1	2	1	130	74	7	2	118	113	14	11	6	8	1	1	2
	Puerto Rico	60	79	4	3	117	96	182	193	14	13	105	109	20	28	10	16	4	7	5
	Qatar	3	6	21	8	9	2	94	97	10	11	99	106	12	10	22	29	3	9	5
	Republic of Korea	1	6	14	12	27	33	167	129	15	3	120	111	16	11	15	24	2	2	0
	San Marino	22	18	2	1	13	5	126	93	5	2	112	105	22	15	12	16	4	3	1
	Singapore	2	8	7	6	19	14	168	131	14	2	118	109	14	12	10	15	3	4	0
	Slovakia	2	2	3	2	7	7	112	116	10	6	98	101	8	10	13	18	4	3	4
	Slovenia	0	2	2	1	4	4	111	115	9	5	98	101	9	11	9	13	3	2	3
	Sweden	2	4	2	2	1	1	144	99	6	1	119	113	9	10	4	5	1	1	2
	Switzerland	7	5	2	2	1	0	126	94	5	2	110	103	9	10	5	9	1	1	2
	Taiwan (Province of China)	3	20	31	26	52	45	283	265	8	10	82	89	13	9	13	33	4	3	1
	United Arab Emirates	32	21	28	25	24	5	95	99	10	10	100	108	31	11	18	24	35	44	3
	United Kingdom	9	9	4	3	2	1	122	91	8	1	119	112	13	12	8	10	2	2	4
	United States of America	21	30	2	2	6	3	172	171	12	3	141	126	16	14	8	12	3	3	6

S14 (B) Percentage change in DALYs/100,000 for each communicable condition for each location for 15-24 year age group by sex. The colours on this heat map are green for values less than 0, the darker the green the greater the reduction in DALYs/100,000 between 1990 and 2019, the orange tones represent positive numbers, an increase in DALYs/ 100,000, with darker shades indicating worse outcome.

		15 to 24																																
		Total commuincable		Enteric infections		Lower respiratory infections		Malaria		Neonatal sepsis and other neonatal infections		Vaccine Preventable disease		Meningitis & Encephalitis		HIV/AIDS		Tuberculosis		Neglected Tropical diseases		Infectious skin conditions		Sexually transmitted infections excluding HIV		Upper respiratory infections		Other unspecified infectious diseases		Hepatitis		Rheumatic heart disease		Maternal sepsis and other maternal infections
		Female	Male	Female	Male	Female	Male	Female	Male	Female	Male	Female	Male	Female	Male	Female	Male	Female	Male	Female	Male	Female	Male	Female	Male	Female	Male	Female	Male	Female	Male	Female	Male	Female
Low SDI	Afghanistan	-1.7%	-1.5%	-0.5%	-0.6%	-1.6%	-1.4%	1.3%	3.1%	45.4%	57.4%	-3.1%	-3.1%	-1.2%	-1.2%	22.3%	14.7%	-2.4%	-2.3%	-0.9%	-1.3%	0.0%	0.0%	-0.1%	0.0%	0.1%	0.1%	-1.4%	-1.4%	-2.1%	-2.1%	-1.9%	-1.6%	-2.2%
	Benin	-1.0%	-1.1%	-1.5%	-1.6%	-0.8%	-1.0%	1.5%	1.2%	45.4%	37.6%	-2.6%	-2.7%	-1.3%	-1.5%	25.0%	112.0%	-1.8%	-2.0%	-2.5%	-2.7%	-0.0%	-0.1%	-1.7%	0.0%	-0.1%	-0.2%	-0.3%	-0.5%	-1.4%	-2.1%	-1.2%	-1.1%	-2.3%
	Burkina Faso	-2.2%	-1.5%	-1.6%	-1.3%	-0.7%	-0.0%	-0.9%	-0.9%	49.1%	59.7%	-3.0%	-2.9%	-1.6%	-1.1%	-3.1%	-1.8%	-1.2%	-1.3%	-2.9%	-3.0%	0.0%	-0.0%	-1.7%	-0.1%	-0.1%	-0.3%	0.4%	0.0%	-1.2%	-1.7%	-0.5%	-0.5%	-2.1%
	Burundi	-2.1%	-1.7%	-1.9%	-1.4%	-1.4%	-1.4%	-1.8%	-2.2%	60.8%	68.4%	-3.0%	-2.8%	-1.4%	-1.3%	-3.0%	-0.1%	-1.8%	-1.9%	-2.0%	-2.0%	-0.0%	-0.1%	-1.2%	-1.7%	-0.1%	-0.2%	-1.9%	-2.3%	-1.2%	-1.3%	-1.3%	-1.4%	-1.1%
	Central African Republic	-1.2%	-0.8%	-0.7%	-0.5%	-0.5%	-0.7%	1.1%	1.6%	23.3%	23.5%	-2.2%	-2.3%	-0.8%	-1.0%	-1.6%	5.5%	-0.3%	-0.6%	-2.6%	-2.7%	0.0%	-0.1%	-0.7%	-1.4%	-0.0%	-0.1%	0.3%	-0.4%	-0.8%	-1.6%	-0.7%	-0.9%	0.0%
	Chad	-1.1%	-0.9%	-1.2%	-1.1%	-0.6%	-0.3%	-1.0%	-0.7%	38.1%	45.4%	-2.8%	-2.6%	-0.7%	-0.3%	0.6%	12.9%	-1.2%	-0.9%	-1.6%	-2.1%	0.0%	0.1%	-1.6%	-0.0%	-0.1%	-0.3%	-0.1%	-0.6%	-0.7%	-1.1%	-1.1%	-0.8%	-1.5%
	Côte d'Ivoire	-1.8%	-1.0%	-1.4%	-1.5%	-0.8%	-1.1%	-0.5%	-0.2%	26.4%	46.7%	-3.1%	-3.0%	-1.3%	-1.4%	-2.3%	4.5%	-1.4%	-1.7%	-2.7%	-2.5%	0.0%	0.1%	-1.7%	0.0%	0.0%	-0.1%	-0.3%	-0.6%	-1.4%	-2.0%	-1.2%	-1.1%	-1.8%
	Dem Rep of the Congo	-1.7%	-1.4%	-1.3%	-0.9%	-1.2%	-0.9%	-1.1%	-1.0%	46.3%	44.5%	-1.9%	-1.6%	-1.2%	-0.9%	-2.7%	-0.2%	-1.3%	-1.4%	-1.9%	-2.2%	-0.0%	-0.1%	-1.0%	-0.8%	0.0%	0.1%	-0.5%	-0.5%	-1.1%	-1.5%	-1.0%	-0.7%	-0.1%
	Eritrea	-1.9%	-1.7%	-2.5%	-1.8%	-1.1%	-0.8%	-2.3%	-2.5%	81.8%	103.8%	-3.2%	-3.1%	-1.0%	-0.8%	-1.2%	11.9%	-1.9%	-1.7%	-2.2%	-2.7%	-0.0%	-0.1%	-0.8%	-1.9%	-0.3%	-0.4%	-1.7%	-2.2%	-1.1%	-0.7%	-1.0%	-1.2%	-2.0%
	Ethiopia	-2.3%	-2.2%	-2.6%	-2.2%	-2.4%	-2.2%	-0.7%	2.5%	98.9%	105.6%	-3.1%	-3.1%	-2.4%	-2.3%	-1.4%	10.4%	-2.8%	-2.6%	-2.2%	-2.7%	-0.1%	-0.2%	-2.0%	-2.2%	-0.6%	-0.7%	-1.0%	-1.2%	-2.0%	-1.4%	-1.6%	-1.6%	-2.7%
	Gambia	-1.1%	-1.3%	-1.5%	-1.3%	-0.6%	0.1%	-2.5%	-2.4%	18.1%	34.2%	-3.0%	-2.9%	-1.4%	-0.8%	23.0%	84.1%	-1.4%	-0.9%	-1.6%	-2.0%	0.0%	0.2%	-1.3%	0.1%	0.0%	-0.1%	1.2%	-0.3%	-1.2%	-0.5%	-1.0%	-0.6%	-1.7%
	Guinea	-0.8%	-0.7%	-1.6%	-1.3%	-0.5%	0.3%	0.4%	0.8%	63.5%	60.0%	-2.9%	-2.8%	-0.9%	-0.3%	10.5%	44.0%	-1.6%	-1.2%	-1.5%	-1.6%	-0.0%	0.0%	-1.7%	0.0%	-0.0%	-0.1%	-0.2%	-0.7%	-1.0%	-0.9%	-1.3%	-0.7%	-1.7%
	Guinea-Bissau	-1.3%	-1.4%	-1.9%	-1.7%	-1.2%	-1.0%	-2.2%	-1.8%	70.6%	107.3%	-2.5%	-2.6%	-1.5%	-1.5%	11.2%	84.9%	-2.0%	-1.8%	-1.5%	-2.4%	-0.1%	-0.1%	-1.9%	-0.1%	-0.1%	-0.2%	0.6%	-0.6%	-1.4%	-1.8%	-1.6%	-1.5%	-2.4%
	Haiti	-1.6%	-1.3%	-1.7%	-1.3%	-1.6%	-1.4%	-1.2%	-1.4%	53.8%	74.1%	-2.5%	-2.6%	-1.9%	-1.8%	-1.7%	2.2%	-2.0%	-2.5%	-1.3%	-2.9%	0.0%	0.0%	-0.5%	-1.2%	-0.1%	-0.0%	-0.3%	-0.4%	-2.0%	-1.4%	-1.8%	-1.4%	-1.0%
	Liberia	-0.9%	-1.2%	-1.3%	-1.3%	-1.1%	-1.0%	1.0%	0.3%	101.4%	120.6%	-3.0%	-3.0%	-1.7%	-1.5%	3.8%	22.9%	-2.0%	-2.0%	-1.5%	-1.9%	-0.1%	-0.2%	-1.5%	0.1%	0.1%	0.1%	-0.7%	-0.7%	-1.1%	-1.5%	-1.3%	-0.8%	-1.5%
	Madagascar	-1.6%	-1.6%	-1.7%	-0.8%	-1.7%	-1.7%	-1.7%	-1.2%	44.7%	59.5%	-2.5%	-2.6%	-1.7%	-1.5%	1,193.1%	2,481.2%	-2.1%	-2.1%	-1.7%	-2.6%	-0.1%	-0.1%	-1.4%	-1.6%	-0.2%	-0.2%	-0.8%	-1.3%	-1.7%	-1.8%	-1.5%	-1.2%	-1.8%
	Malawi	-2.3%	-1.5%	-1.8%	-1.2%	-1.9%	-0.7%	-2.1%	-1.9%	74.5%	74.7%	-2.9%	-2.4%	-2.1%	-1.0%	-2.5%	-0.7%	-2.1%	-1.5%	-2.8%	-2.9%	0.0%	0.0%	-1.4%	-1.3%	-0.2%	-0.2%	-0.2%	-0.5%	-2.0%	-1.7%	-1.2%	-0.7%	-1.8%
	Mali	-1.8%	-1.6%	-1.9%	-0.9%	-1.5%	-1.1%	-2.0%	-1.8%	90.3%	69.9%	-3.0%	-2.9%	-1.9%	-1.4%	1.3%	13.7%	-1.1%	-1.8%	-2.4%	-2.8%	-0.1%	-0.0%	-2.0%	-0.0%	-0.2%	-0.3%	-0.5%	-0.3%	-2.0%	-2.2%	-1.8%	-1.1%	-2.4%
	Mozambique	1.5%	0.3%	-2.2%	-1.2%	-0.9%	1.0%	-1.8%	-1.6%	60.8%	62.4%	-3.1%	-3.0%	-1.6%	-0.4%	24.0%	55.1%	-1.7%	-0.7%	-2.2%	-2.4%	-0.0%	0.0%	-1.1%	-1.3%	-0.6%	-0.5%	-0.5%	-0.7%	-1.4%	-1.1%	-0.6%	-0.3%	-2.6%
	Nepal	-2.3%	-2.1%	-2.2%	-2.1%	-2.2%	-1.7%	-3.1%	-3.0%	140.5%	117.3%	-3.1%	-3.0%	-1.6%	-1.3%	66,683.1%	52,995.2%	-2.9%	-2.6%	-2.3%	-2.7%	-0.0%	-0.0%	-1.7%	-0.2%	-0.3%	-0.3%	-0.8%	-0.7%	-2.3%	-1.5%	-2.1%	-1.4%	-3.2%
	Niger	-1.6%	-1.5%	-1.8%	-1.4%	-1.2%	-1.0%	-1.2%	-0.8%	77.6%	81.0%	-2.4%	-2.4%	-1.5%	-1.5%	-0.7%	6.6%	-2.0%	-1.9%	-2.1%	-2.6%	-0.1%	-0.1%	-1.7%	-0.1%	0.0%	-0.1%	-1.1%	-1.1%	-1.3%	-1.5%	-1.3%	-1.1%	-1.4%
	Pakistan	-1.6%	-1.2%	-1.3%	-1.5%	-0.0%	0.1%	-2.7%	-2.7%	24.8%	19.4%	-3.1%	-3.1%	-1.0%	-0.7%	36.4%	24.4%	-1.6%	-1.2%	-2.5%	-2.2%	-0.0%	-0.0%	-0.3%	0.2%	0.1%	0.0%	0.2%	-0.7%	-1.2%	-0.7%	-0.5%	0.0%	-2.4%
	Papua New Guinea	-0.8%	-1.0%	-1.6%	-1.5%	-0.7%	-0.6%	-1.7%	-1.6%	11.2%	14.8%	-2.3%	-2.4%	-1.5%	-1.3%	963.3%	2,508.6%	-1.6%	-1.6%	-1.6%	-1.1%	-0.0%	-0.0%	-0.2%	0.0%	-0.1%	-0.1%	-0.3%	0.4%	-1.7%	-1.6%	-0.0%	-0.0%	-1.9%
	Rwanda	-2.4%	-2.2%	-2.5%	-2.0%	-2.1%	-1.9%	-2.6%	-2.7%	88.5%	99.3%	-3.1%	-3.0%	-2.2%	-1.9%	-2.0%	3.0%	-2.7%	-2.6%	-2.4%	-2.0%	-0.2%	-0.3%	-1.7%	-1.8%	-0.3%	-0.4%	-1.6%	-2.2%	-1.9%	-2.1%	-1.8%	-1.8%	-2.7%
	Senegal	-1.6%	-1.5%	-1.9%	-1.6%	-1.3%	-0.9%	-1.6%	-1.5%	34.9%	46.6%	-2.9%	-2.8%	-1.6%	-1.2%	1.4%	20.2%	-2.1%	-1.9%	-1.5%	-2.2%	-0.1%	0.0%	-1.7%	0.1%	-0.1%	-0.2%	0.1%	-0.9%	-1.7%	-1.6%	-1.2%	-0.9%	-2.1%
	Sierra Leone	-0.4%	-1.2%	-1.1%	-0.8%	0.3%	-0.6%	-0.3%	-1.1%	41.6%	44.5%	-2.9%	-2.8%	-0.9%	-1.2%	13.4%	32.7%	-1.2%	-1.8%	-2.3%	-2.5%	-0.0%	0.0%	-1.3%	-0.0%	-0.0%	-0.1%	-0.5%	-0.9%	-0.9%	-2.0%	-0.9%	-1.0%	-0.7%
	Solomon Islands	-1.0%	-1.3%	-1.5%	-1.6%	-1.2%	-1.4%	-2.0%	-1.9%	3.8%	7.4%	-2.9%	-2.9%	-1.3%	-1.5%	22.2%	20.3%	-1.3%	-1.6%	-0.4%	-0.4%	-0.0%	-0.0%	-0.1%	-0.0%	-0.1%	-0.1%	-0.3%	-0.6%	-1.5%	-1.9%	-0.6%	-0.7%	-1.1%
	Somalia	-1.3%	-1.3%	-1.6%	-1.2%	-0.8%	-0.1%	-1.8%	-1.7%	29.1%	27.2%	-2.3%	-1.8%	-0.0%	0.9%	78.9%	413.0%	-0.7%	0.1%	-2.6%	-3.0%	-0.1%	-0.1%	-0.6%	-1.2%	0.1%	0.2%	-1.6%	-1.7%	-0.7%	0.1%	-0.9%	-0.7%	0.2%
	South Sudan	-0.8%	-1.2%	-0.9%	-0.2%	-1.2%	-0.6%	-1.1%	-0.1%	41.5%	42.6%	-2.6%	-2.3%	-0.4%	0.3%	15.6%	48.7%	-1.3%	-1.0%	-1.8%	-2.2%	0.0%	0.1%	-0.8%	-1.3%	-0.1%	-0.1%	-1.8%	-1.6%	-0.5%	0.2%	-0.9%	-0.6%	-1.0%
	Togo	-0.8%	-0.5%	-1.3%	-1.2%	-1.1%	-0.3%	-0.2%	0.1%	36.2%	39.3%	-3.0%	-2.8%	-1.6%	-1.0%	3.3%	42.1%	-1.8%	-1.4%	-2.4%	-2.5%	-0.0%	0.0%	-1.8%	-0.0%	-0.0%	-0.1%	-0.2%	-0.8%	-1.6%	-1.8%	-1.4%	-0.7%	-2.1%
	Uganda	-2.5%	-1.7%	-1.2%	-0.9%	0.0%	0.0%	-1.6%	-1.7%	52.9%	54.7%	-2.6%	-2.6%	-0.6%	-0.4%	-2.9%	-0.8%	-1.1%	-1.4%	-3.0%	-3.1%	0.0%	0.0%	-0.4%	-0.9%	-0.3%	-0.3%	-1.5%	-1.8%	-0.3%	-0.3%	-0.2%	-0.4%	-1.5%
	United Republic of Tanzania	-2.3%	-1.8%	-1.7%	-1.8%	-1.3%	-1.2%	-2.0%	-2.2%	46.4%	45.4%	-2.8%	-2.8%	-0.8%	-0.7%	-2.7%	-1.6%	-1.7%	-1.8%	-2.4%	-2.7%	-0.3%	-0.3%	-1.0%	-1.1%	-0.3%	-0.3%	-0.6%	-0.6%	-1.3%	-1.3%	-0.7%	-1.0%	-2.1%
	Yemen	-1.9%	-2.1%	0.7%	0.2%	-0.4%	0.2%	-2.2%	-2.3%	23.5%	29.7%	-3.0%	-3.0%	-0.6%	-0.5%	12.6%	8.7%	-2.1%	-1.9%	-1.9%	-2.4%	0.0%	-0.0%	-0.1%	0.0%	0.0%	0.1%	2.3%	0.1%	-1.8%	-2.2%	-1.0%	-0.7%	-1.7%

15 to 24

Low-middle SDI

	Total communicable		Enteric infections		Lower respiratory infections		Malaria		Neonatal sepsis and other neonatal infections		Vaccine Preventable disease		Meningitis & Encephalitis		HIV/AIDS		Tuberculosis		Neglected Tropical diseases		Infectious skin conditions		Sexually transmitted infections excluding HIV		Upper respiratory infections		Other unspecified infectious diseases		Hepatitis		Rheumatic heart disease		Maternal sepsis and other maternal infections
	Female	Male	Female	Male	Female	Male	Female	Male	Female	Male	Female	Male	Female	Male	Female	Male	Female	Male	Female	Male	Female	Male	Female	Male	Female	Male	Female	Male	Female	Male	Female	Male	Female
Angola	-1.3%	-1.9%	-2.4%	-2.0%	-1.6%	-1.3%	-0.6%	0.0%	79.0%	105.2%	-2.9%	-2.9%	-1.7%	-1.5%	40.7%	99.6%	-2.2%	-2.2%	-2.8%	-3.0%	-0.1%	-0.2%	-1.2%	-1.4%	-0.3%	-0.4%	-0.2%	-1.0%	-1.3%	-2.1%	-1.3%	-1.2%	-2.2%
Bangladesh	-2.4%	-2.3%	-2.0%	-2.0%	-2.0%	-2.3%	-3.3%	-3.3%	156.5%	239.0%	-2.9%	-3.1%	-1.5%	-1.7%			-2.8%	-2.8%	-2.3%	-2.5%	-0.0%	-0.1%	-1.7%	-1.9%	-0.3%	-0.4%	-1.1%	-1.3%	-2.8%	-2.8%	-1.6%	-1.8%	-3.1%
Belize	-0.5%	-0.4%	0.4%	0.4%	0.1%	1.3%	-3.3%	-3.3%	6.4%	7.7%	-1.5%	-2.1%	-1.3%	-1.5%	-0.3%	-0.5%	-2.2%	-1.6%	-0.9%	0.2%	0.0%	0.1%	0.2%	-0.0%	-0.1%	-0.2%	0.3%	0.3%	-2.3%	-1.1%	-0.8%	-0.6%	-1.6%
Bhutan	-2.5%	-2.2%	-2.2%	-2.1%	-2.0%	-1.3%	-3.3%	-3.3%	153.8%	123.2%	-3.3%	-3.3%	-2.0%	-1.7%	15.2%	13.0%	-2.9%	-2.6%	-2.6%	-2.6%	-0.1%	-0.1%	-1.5%	-0.0%	-0.4%	-0.5%	0.3%	-0.7%	-2.3%	-2.0%	-2.1%	-1.4%	-3.0%
Bolivia (Plurinational State of)	-2.2%	-1.9%	-1.7%	-0.8%	-2.0%	-1.5%	-3.3%	-3.3%	26.1%	21.6%	-3.0%	-2.9%	-2.1%	-1.6%	5.6%	1.9%	-2.9%	-2.8%	-2.1%	-1.9%	-0.1%	-0.1%	-0.9%	-0.7%	-0.2%	-0.2%	-1.4%	-1.0%	-2.1%	-1.6%	-1.7%	-1.2%	-2.7%
Cabo Verde	-2.0%	-1.0%	-2.3%	-2.2%	-2.0%	0.1%	3.8%	4.3%	17.9%	16.6%	-3.2%	-3.2%	-2.0%	-0.5%	-1.3%	8.6%	-2.7%	-2.0%	-2.4%	-1.5%	-0.2%	-0.2%	-0.3%	0.2%	-0.2%	-0.4%	0.5%	-2.4%	-2.3%	-0.1%	-2.3%	-1.5%	-3.1%
Cambodia	-2.2%	-1.9%	-2.1%	-2.0%	-2.1%	-1.5%	-3.1%	-3.1%	56.5%	56.7%	-3.2%	-3.1%	-1.4%	-1.1%	173.4%	59.0%	-2.6%	-2.2%	-2.7%	-2.9%	-0.1%	-0.2%	-0.4%	-0.2%	-0.4%	-0.4%	-1.2%	-0.6%	-1.8%	-1.3%	-2.1%	-1.7%	-3.0%
Cameroon	-0.7%	-0.9%	-1.7%	-1.5%	-0.8%	-0.2%	0.2%	0.3%	33.9%	48.2%	-3.0%	-2.9%	-1.5%	-1.1%	5.2%	22.5%	-1.7%	-1.5%	-2.5%	-2.6%	-0.0%	0.0%	-1.6%	0.0%	-0.0%	-0.0%	-1.6%	-2.5%	-1.5%	-2.0%	-1.2%	-0.6%	-2.0%
Comoros	-1.8%	-1.8%	-1.8%	-1.4%	-1.0%	-1.0%	-2.2%	-2.3%	41.4%	50.8%	-2.4%	-2.4%	-0.6%	-0.5%	65.0%	59.2%	-1.7%	-1.9%	-2.2%	-2.1%	-0.1%	-0.1%	-0.8%	-0.9%	-0.1%	-0.1%	-0.9%	-1.6%	-0.8%	-0.4%	-1.1%	-0.8%	-2.2%
Congo	-1.9%	-1.8%	-1.8%	-1.8%	-1.3%	-1.8%	0.4%	-1.3%	21.2%	36.5%	-2.0%	-2.3%	-1.4%	-1.9%	-1.9%	7.2%	-1.8%	-2.2%	-3.0%	-3.1%	-0.1%	-0.3%	-0.9%	-1.0%	-0.1%	-0.2%	0.0%	-1.1%	-1.1%	-2.0%	-1.0%	-1.3%	-2.1%
Democratic People's Republic of Kor...	-0.8%	-0.6%	0.3%	0.3%	-1.6%	-1.2%	133.3%	100.7%	10.3%	14.6%	-3.1%	-3.1%	-1.1%	-0.9%	54.8%	52.9%	-1.1%	-0.4%	-0.6%	-2.1%	-0.0%	-0.1%	-0.2%	-0.0%	-0.1%	-0.1%	-0.3%	-0.2%	-1.6%	-1.5%	-1.2%	-0.5%	-1.3%
Djibouti	-0.5%	-1.1%	-1.9%	-1.6%	-1.2%	-0.6%	-1.7%	-1.5%	22.7%	32.9%	-2.7%	-2.5%	-1.0%	-0.2%	407.4%	1,486.0%	-2.0%	-1.6%	-2.9%	-3.0%	-0.1%	-0.0%	-0.7%	-1.3%	-0.1%	-0.1%	-1.4%	-1.8%	-1.3%	-0.6%	-0.9%	-0.8%	-1.5%
Dominican Republic	-0.7%	-1.0%	0.0%	-0.5%	-0.4%	0.3%	-1.6%	-1.6%	14.3%	18.9%	-1.9%	-2.2%	-1.2%	-1.1%	-1.1%	-0.5%	-2.1%	-2.0%	-1.1%	-2.0%	-0.0%	-0.0%	-0.3%	-1.2%	-0.2%	-0.2%	-0.4%	-0.5%	-1.4%	-1.1%	-1.2%	-1.0%	-1.6%
El Salvador	-1.4%	-1.0%	-1.8%	-1.9%	-0.9%	-0.4%	-3.2%	-2.9%	0.4%	0.4%	-2.6%	-2.8%	-2.2%	-2.2%	11.8%	5.5%	-2.8%	-2.8%	-1.6%	-1.2%	-0.1%	-0.1%	-0.2%	0.1%	-0.2%	-0.2%	-1.0%	-0.7%	-0.9%	-1.5%	-0.7%	-0.5%	-3.2%
Eswatini	4.7%	2.4%	-1.2%	-0.5%	-0.7%	0.8%	-2.9%	-2.9%	8.6%	9.8%	-3.1%	-3.0%	-0.9%	0.2%	57.1%	155.1%	-0.9%	-0.4%	-2.1%	-1.9%	-0.0%	0.0%	-0.7%	-0.2%	-0.3%	-0.2%	-0.3%	0.1%	-1.1%	1.0%	-1.0%	-0.6%	-2.2%
Ghana	-1.3%	-0.8%	-1.9%	-1.9%	-1.6%	1.2%	-0.5%	0.8%	40.0%	66.7%	-3.1%	-3.0%	-2.0%	-1.2%	1.1%	26.0%	-2.3%	-1.8%	-2.3%	-2.5%	-0.1%	-0.1%	-0.8%	0.1%	-0.2%	-0.3%	-0.0%	0.4%	-2.0%	-1.7%	-1.3%	-0.6%	-2.7%
Guatemala	-2.5%	-2.3%	-2.9%	-2.8%	-2.5%	-2.2%	-3.2%	-3.3%	6.8%	9.7%	-3.2%	-3.1%	-1.7%	-1.5%	0.4%	-1.1%	-3.1%	-3.0%	-2.5%	-2.5%	0.0%	0.0%	-0.3%	-1.0%	-0.4%	-0.3%	-1.0%	-0.2%	-1.6%	-1.1%	-0.8%	-0.4%	-2.8%
Honduras	-2.0%	-1.4%	-2.4%	-2.1%	-3.0%	-2.0%	-3.2%	-3.2%	11.1%	11.1%	-2.4%	-2.6%	-2.0%	-2.0%	-0.4%	-1.4%	-2.6%	-2.5%	-1.2%	-1.0%	-0.1%	-0.1%	-0.4%	-0.0%	-0.3%	-0.2%	-1.8%	-1.8%	-2.1%	-1.8%	-0.2%	-0.1%	-3.1%
India	-2.2%	-2.0%	-2.2%	-2.2%	-1.7%	-1.4%	-2.8%	-2.7%	37.1%	37.0%	-3.1%	-3.1%	-1.9%	-1.9%	187.9%	89.6%	-2.6%	-2.3%	-2.3%	-2.3%	-0.2%	-0.2%	-1.6%	-0.1%	-0.4%	-0.4%	-0.5%	-1.2%	-1.8%	-1.3%	-1.8%	-1.7%	-3.1%
Kenya	-1.4%	-0.2%	-1.5%	-1.4%	-0.8%	0.4%	-1.3%	-1.4%	11.3%	9.9%	-2.1%	-1.9%	-1.3%	-0.4%	-1.4%	6.7%	-1.3%	-0.1%	-1.7%	-2.2%	-0.0%	-0.0%	-0.6%	-0.5%	0.0%	0.0%	0.8%	0.1%	-1.0%	-0.2%	-0.3%	-0.0%	-2.1%
Kiribati	-1.3%	-1.4%	-1.7%	-1.9%	-1.0%	-0.8%			9.8%	9.7%	-1.9%	-2.0%	-1.3%	-1.4%	1.6%	4.6%	-1.4%	-1.1%	-1.6%	-2.4%	-0.0%	-0.1%	-0.4%	-0.1%	0.0%	0.1%	0.0%	-0.8%	-1.4%	-2.1%	-1.1%	-1.0%	-1.2%
Kyrgyzstan	-1.4%	-1.3%	0.9%	0.9%	-2.3%	-2.2%	-3.3%	-3.3%	2.4%	4.8%	-0.2%	-1.1%	-2.2%	-1.9%	10.1%	5.1%	-1.0%	-1.0%	-0.4%	0.2%	-0.1%	-0.1%	-0.1%	-0.1%	0.0%	0.1%	0.2%	-0.8%	-2.6%	-1.8%	-2.3%	-2.2%	-1.0%
Lao People's Democratic Republic	-2.2%	-2.1%	-2.1%	-2.1%	-2.1%	-2.0%	-2.7%	-2.9%	75.9%	81.8%	-3.1%	-3.1%	-2.0%	-1.8%	29,223.0%	39,068.1%	-2.6%	-2.5%	-1.6%	-2.0%	-0.1%	-0.2%	-0.2%	-0.1%	-0.4%	-0.4%	-0.8%	-1.2%	-2.5%	-2.5%	-1.1%	-1.2%	-3.1%
Lesotho	5.2%	2.8%	-0.8%	-0.6%	0.6%	1.1%			8.8%	10.8%	-3.0%	-2.9%	-0.2%	0.2%	17.9%	43.9%	0.6%	0.2%	-2.9%	-2.9%	0.0%	0.0%	-0.5%	-0.1%	-0.4%	-0.4%	-0.2%	0.1%	-0.8%	-0.3%	-0.5%	-0.5%	-1.9%
Maldives	-2.1%	-1.4%	-2.1%	-1.8%	-2.6%	-1.6%			19.4%	14.7%	-3.2%	-3.2%	-1.9%	-1.5%	17.5%	14.2%	-3.1%	-2.9%	-2.1%	-1.4%	-0.1%	-0.1%	-0.7%	0.0%	-0.2%	-0.3%	-1.5%	-1.4%	-2.9%	-1.5%	-2.6%	-1.7%	-3.0%
Marshall Islands	-0.6%	-1.4%	-1.7%	-2.0%	-0.1%	-1.5%			1.8%	2.5%	-2.1%	-2.1%	-1.1%	-2.0%	24.6%	21.0%	-1.3%	-1.7%	-1.4%	-1.0%	-0.0%	-0.1%	-0.0%	-0.1%	-0.1%	-0.1%	-0.1%	-1.2%	-1.7%	-2.2%	0.2%	-1.3%	-1.7%
Mauritania	-1.5%	-1.2%	-2.0%	-1.9%	-1.7%	-1.8%	12.8%	12.6%	45.1%	64.6%	-3.1%	-3.1%	-2.0%	-2.0%	-1.7%	-1.6%	-2.5%	-2.6%	-1.6%	-2.4%	-0.1%	-0.2%	-1.9%	-0.1%	-0.1%	-0.2%	-0.8%	-1.4%	-1.8%	-2.1%	-1.7%	-1.3%	-2.9%
Micronesia (Federated States of)	-1.1%	-1.3%	-1.9%	-2.0%	-0.7%	-0.8%			3.1%	5.0%	-2.6%	-2.6%	-1.4%	-1.5%	41.0%	39.0%	-2.0%	-2.0%	-2.4%	-2.8%	-0.0%	-0.1%	-0.0%	0.1%	-0.1%	-0.1%	-0.1%	-0.5%	-1.7%	-1.4%	-0.7%	-0.8%	-2.3%
Mongolia	-2.0%	-1.4%	0.3%	0.2%	-2.1%	-1.8%			10.6%	11.3%	-2.3%	-2.3%	-2.8%	-2.4%	1,639.7%	1,581.8%	-1.8%	-1.3%	-2.5%	-1.3%	-0.0%	-0.0%	0.0%	0.1%	-0.2%	-0.3%	-0.8%	-2.4%	-2.6%	-2.1%	-1.3%	-1.0%	-2.7%
Morocco	-1.9%	-1.3%	-1.1%	-1.2%	-1.3%	-0.9%	-3.3%	-3.3%	17.6%	27.3%	-3.0%	-3.0%	-1.6%	-1.5%	16.4%	13.9%	-2.5%	-2.5%	-1.5%	-0.8%	-0.0%	-0.0%	-0.6%	-0.2%	-0.2%	-0.1%	-0.6%	-1.0%	-2.0%	-2.0%	-1.9%	-1.6%	-3.1%
Myanmar	-2.2%	-1.9%	-2.0%	-1.8%	-2.3%	-1.8%	-3.2%	-3.2%	52.2%	63.3%	-3.1%	-3.0%	-2.7%	-1.7%	169.7%	60.1%	-2.8%	-2.3%	-2.1%	-2.4%	-0.1%	-0.1%	-0.5%	-0.1%	-0.5%	-0.6%	0.2%	0.7%	-1.8%	-1.5%	-1.9%	-1.5%	-2.7%
Nicaragua	-1.3%	-0.5%	-1.6%	-1.0%	-1.3%	0.6%	-2.7%	-2.5%	-1.1%	-0.5%	-2.7%	-2.7%	-2.1%	-1.3%	51.0%	47.7%	-2.8%	-2.8%	-1.8%	-1.4%	-0.0%	0.0%	-0.5%	0.2%	-0.1%	-0.1%	-1.7%	-0.2%	-1.3%	0.4%	-1.3%	-0.7%	-2.9%
Nigeria	-1.3%	-1.5%	-1.8%	-1.7%	-2.1%	-1.9%	-0.9%	-0.7%	28.5%	43.7%	-3.0%	-2.8%	-1.6%	-1.3%	2.7%	11.9%	-2.1%	-2.0%	-1.7%	-2.5%	0.0%	0.1%	-1.6%	-0.1%	-0.2%	-0.4%	2.7%	-0.3%	-1.8%	-1.7%	-1.5%	-1.1%	-2.0%
Palestine	-1.3%	-1.4%	-1.1%	-1.0%	-1.5%	-1.7%			4.0%	8.3%	-3.2%	-3.2%	-1.9%	-2.1%	104.0%	28.5%	-2.5%	-2.7%	-1.8%	-2.7%	-0.0%	-0.0%	-0.1%	0.0%	-0.2%	-0.1%	-0.9%	-0.7%	-1.9%	-1.4%	-1.0%	-0.9%	-2.5%
Sao Tome and Principe	-2.3%	-1.6%	-1.5%	-1.1%	0.0%	3.0%	-3.0%	-3.0%	16.3%	28.4%	-2.9%	-2.7%	-0.3%	1.0%	-1.9%	-2.4%	-1.8%	-1.1%	-1.9%	-1.7%	-0.0%	0.1%	-1.0%	0.1%	-0.1%	-0.1%	0.2%	1.3%	-1.4%	-0.2%	-0.6%	0.6%	-2.5%
Sudan	-1.9%	-2.1%	-0.6%	-0.5%	-1.7%	-1.0%	-1.9%	-2.2%	37.6%	44.7%	-3.2%	-3.2%	-2.4%	-2.2%	15.6%	54.7%	-2.7%	-2.4%	-2.5%	-2.7%	0.0%	0.0%	0.5%	-0.0%	-0.2%	-0.2%	-0.6%	-0.8%	-2.0%	-2.4%	-2.1%	-1.5%	-2.8%
Tajikistan	-1.4%	-0.6%	0.6%	0.5%	-1.7%	-0.8%	-3.3%	-3.3%	6.2%	8.4%	-1.8%	-1.7%	-2.5%	-2.2%	7.4%	0.8%	-0.2%	2.1%	-1.3%	-0.4%	-0.0%	-0.0%	-0.9%	-0.2%	0.2%	0.1%	-0.0%	-0.5%	-2.4%	-0.8%	-2.2%	-1.6%	-1.4%
Timor-Leste	-2.1%	-1.9%	-1.6%	-1.4%	-1.6%	0.0%	-3.3%	-3.3%	46.1%	28.1%	-3.1%	-3.0%	-1.3%	-0.5%	27.0%	23.3%	-2.3%	-1.4%	-1.9%	-2.2%	-0.0%	-0.0%	-0.2%	-0.2%	-0.3%	-0.3%	0.2%	0.1%	-2.3%	-1.3%	-0.5%	0.4%	-2.7%
Tuvalu	-1.5%	-1.2%	-1.8%	-1.6%	-1.7%	-1.4%			16.2%	17.4%	-2.5%	-2.5%	-1.8%	-1.7%	95.6%	45.0%	-2.4%	-2.5%	-0.8%	0.8%	-0.0%	-0.1%	-0.3%	-0.0%	-0.2%	-0.2%	-0.8%	-0.6%	-2.0%	-1.7%	-1.5%	-1.2%	-2.8%
Vanuatu	-1.5%	-1.9%	-0.8%	-1.1%	-0.0%	0.1%	-3.3%	-3.3%	2.5%	3.4%	-2.1%	-2.2%	-0.5%	-0.8%	25.0%	19.3%	-1.0%	-1.0%	0.2%	-2.7%	0.0%	0.0%	-0.1%	0.1%	-0.1%	-0.0%	1.0%	0.6%	-1.2%	-1.9%	0.6%	0.3%	-1.0%
Venezuela (Bolivarian Republic of)	-0.0%	-0.1%	-0.1%	0.0%	-0.3%	-1.2%	12.1%	11.1%	2.9%	3.5%	-2.1%	-1.9%	-1.0%	-1.6%	3.3%	1.7%	-1.3%	-1.9%	-1.5%	-1.3%	-0.0%	-0.0%	0.5%	0.0%	-0.0%	-0.0%	-0.1%	0.2%	-1.8%	-1.9%	-2.3%	-2.5%	-1.7%
Zambia	-2.1%	-1.4%	-2.0%	-1.2%	-1.6%	-0.4%	-2.0%	-1.7%	58.8%	72.0%	-3.0%	-2.8%	-2.2%	-1.4%	-2.1%	-0.4%	-2.2%	-1.8%	-2.3%	-2.8%	-0.1%	-0.0%	-1.2%	-0.8%	-0.2%	-0.2%	-1.5%	-0.8%	-1.4%	-0.6%	-1.3%	-0.7%	-2.4%
Zimbabwe	-1.4%	0.4%	1.0%	1.2%	0.6%	1.5%	-2.7%	-2.7%	7.4%	7.9%	-2.7%	-2.6%	0.1%	0.8%	-1.8%	1.3%	0.8%	1.7%	-2.4%	-2.4%	0.0%	0.1%	-0.2%	0.0%	-0.0%	0.1%	0.8%	1.3%	-0.4%	-1.2%	0.2%	0.4%	0.8%

15 to 24

		Total communicable		Enteric infections		Lower respiratory infections		Malaria		Neonatal sepsis and other neonatal infections		Vaccine Preventable disease		Meningitis & Encephalitis		HIV/AIDS		Tuberculosis		Neglected Tropical diseases		Infectious skin conditions		Sexually transmitted infections excluding HIV		Upper respiratory infections		Other unspecified infectious diseases		Hepatitis		Rheumatic heart disease		Maternal sepsis and other maternal infections
		Female	Male	Female	Male	Female	Male	Female	Male	Female	Male	Female	Male	Female	Male	Female	Male	Female	Male	Female	Male	Female	Male	Female	Male	Female	Male	Female	Male	Female	Male	Female	Male	Female
Middle SDI	Albania	-1.3%	-1.3%	-0.7%	-0.6%	-2.5%	-2.1%			4.0%	8.3%	-3.1%	-3.0%	-2.2%	-1.9%	6.5%	13.6%	-2.6%	-2.6%	-2.3%	-2.6%	-0.0%	-0.1%	-0.3%	0.1%	-0.2%	-0.2%	-1.4%	-1.2%	-1.9%	-1.2%	-1.3%	-1.1%	-1.2%
	Algeria	-1.8%	-1.4%	-0.7%	-0.9%	-1.9%	-1.9%	-3.0%	-3.0%	16.6%	22.3%	-3.1%	-3.1%	-1.9%	-2.0%	10.8%	14.3%	-2.8%	-2.7%	-1.6%	-2.1%	-0.0%	-0.0%	-0.1%	-0.1%	-0.0%	-0.0%	-1.0%	-1.0%	-1.9%	-1.9%	-2.1%	-1.7%	-3.1%
	Armenia	-0.7%	-0.1%	0.5%	0.7%	-1.3%	0.4%	-3.3%	-3.3%	-0.5%	-0.2%	-1.8%	0.1%	-1.7%	-0.9%	15.8%	27.9%	-1.9%	-1.2%	-0.8%	-0.2%	-0.0%	0.0%	-0.5%	-0.6%	-0.1%	-0.1%	0.5%	-0.2%	-1.5%	0.5%	-1.5%	-1.1%	-2.0%
	Azerbaijan	-0.9%	-0.3%	1.1%	1.0%	-1.0%	-0.5%	-3.3%	-3.3%	5.8%	11.1%	-2.1%	-2.3%	-1.2%	-1.1%	4.8%	-0.2%	-1.5%	-0.2%	-1.9%	-1.0%	-0.0%	-0.0%	-0.8%	-0.8%	-0.1%	-0.2%	-0.3%	-0.4%	-1.6%	-0.3%	-1.4%	-1.0%	-1.8%
	Botswana	-1.1%	-0.9%	-1.9%	-2.4%	-0.3%	-1.1%	-0.8%	-1.5%	9.7%	14.2%	-3.1%	-3.1%	-0.8%	-1.4%	-0.9%	1.8%	-1.2%	-1.9%	-2.2%	-2.4%	-0.1%	-0.1%	-0.9%	-0.2%	-0.3%	-0.5%	-0.4%	-1.2%	-1.4%	-1.8%	-1.1%	-1.5%	-2.6%
	Brazil	-1.0%	-1.3%	-0.6%	-1.0%	-1.4%	-1.4%	-3.1%	-3.2%	7.8%	13.7%	-2.0%	-2.8%	-2.1%	-2.0%	0.2%	-1.6%	-2.2%	-2.0%	-1.0%	-1.1%	0.0%	0.0%	-0.6%	-0.1%	-0.2%	-0.4%	-0.6%	-0.5%	-1.5%	-1.9%	-1.3%	-1.5%	-2.0%
	China	-1.5%	-1.4%	-1.3%	-1.7%	-2.5%	-2.5%	-3.3%	-3.3%	24.5%	22.7%	-3.0%	-3.0%	-2.3%	-2.3%	15.4%	23.8%	-2.7%	-2.5%	-2.5%	-2.2%	-0.1%	-0.1%	-0.7%	-0.4%	-0.5%	-0.7%	-2.0%	-2.2%	-2.4%	-2.3%	-2.4%	-2.2%	-3.1%
	Colombia	-1.0%	-0.9%	-1.6%	-1.5%	-1.4%	-1.3%	-3.1%	-3.2%	4.7%	11.8%	-2.0%	-2.5%	-1.5%	-1.7%	11.9%	4.7%	-2.3%	-2.5%	-1.2%	-0.7%	0.1%	0.1%	-0.6%	-0.4%	-0.2%	-0.3%	-0.8%	-0.2%	-1.0%	-1.3%	-2.8%	-2.9%	-2.2%
	Costa Rica	-0.9%	-0.5%	-0.7%	-0.4%	-1.3%	0.3%	-3.3%	-3.3%	-1.5%	-2.1%	-1.6%	-0.9%	-1.6%	-1.3%	0.5%	0.9%	-2.4%	-2.3%	-1.2%	-0.9%	-0.0%	0.0%	-0.2%	0.1%	-0.2%	-0.2%	-0.1%	1.7%	-1.6%	-0.5%	-0.8%	-0.7%	-2.4%
	Cuba	-0.8%	-0.6%	1.3%	1.1%	-1.5%	-1.5%			-0.2%	-0.4%	-0.6%	-0.8%	-2.5%	-2.6%	1.2%	8.5%	-2.5%	-2.5%	-0.8%	-0.9%	-0.0%	-0.1%	2.0%	-0.0%	-0.0%	-0.1%	-0.2%	0.2%	-2.4%	-2.1%	-1.4%	-1.4%	-2.0%
	Ecuador	-2.0%	-1.5%	-0.8%	-1.3%	-1.3%	-0.1%	-3.3%	-3.3%	-0.2%	-0.0%	-2.7%	-2.6%	-1.7%	-1.5%	11.5%	16.1%	-3.0%	-2.6%	-2.1%	-1.9%	-0.0%	0.0%	-0.0%	-0.3%	-0.1%	-0.1%	-1.0%	0.4%	-2.3%	-1.7%	-1.3%	-1.2%	-2.5%
	Egypt	-2.1%	-1.6%	-0.7%	-0.8%	-1.9%	-0.8%	-3.3%	-3.3%	12.6%	13.9%	-3.1%	-3.0%	-1.9%	-1.5%	2.7%	4.9%	-2.7%	-2.4%	-3.0%	-3.0%	-0.3%	-0.3%	-0.2%	-0.3%	-0.2%	-0.2%	-1.1%	-1.3%	-2.4%	-1.6%	-2.3%	-1.8%	-3.0%
	Equatorial Guinea	-0.4%	-2.1%	-3.0%	-2.9%	-2.1%	-2.2%	0.6%	-1.2%	100.9%	128.0%	-3.1%	-3.1%	-2.4%	-2.4%	49.6%	188.6%	-2.9%	-3.0%	-2.7%	-3.0%	-0.2%	-0.6%	-1.4%	-1.3%	-0.6%	-0.9%	-1.3%	-2.0%	-2.0%	-2.6%	-1.7%	-2.0%	-3.2%
	Fiji	-1.2%	-1.2%	-1.7%	-1.5%	-1.4%	-1.2%			0.5%	2.3%	-2.6%	-2.5%	-1.9%	-1.6%	12.1%	12.6%	-1.9%	-1.7%	-1.1%	-1.7%	-0.0%	-0.0%	-0.5%	-0.0%	-0.1%	-0.1%	-0.7%	0.3%	-1.4%	-1.3%	-1.4%	-1.2%	-1.7%
	Gabon	-1.0%	-1.8%	-2.0%	-2.0%	-1.7%	-1.2%	0.2%	-1.1%	24.4%	28.8%	-2.6%	-2.7%	-1.7%	-1.5%	1.6%	12.6%	-2.0%	-2.2%	-2.5%	-2.8%	-0.1%	-0.2%	-0.8%	-0.8%	-0.1%	-0.1%	0.1%	-0.6%	-1.4%	-1.6%	-0.8%	-1.0%	-2.7%
	Grenada	-0.9%	-0.7%	0.9%	1.0%	-0.9%	-0.5%			4.5%	5.7%	-2.5%	-2.6%	-2.3%	-2.4%	-1.7%	-2.2%	-2.2%	-2.2%	-1.7%	-1.5%	0.1%	0.2%	0.1%	-0.8%	-0.1%	-0.3%	0.0%	0.1%	-2.5%	-2.1%	-1.6%	-1.2%	-2.3%
	Guyana	-1.3%	-1.5%	-1.3%	-1.7%	-0.0%	-0.2%	-2.8%	-2.9%	2.8%	9.2%	-2.5%	-2.4%	-1.4%	-1.8%	2.1%	-1.1%	-1.7%	-1.7%	-1.9%	-0.7%	0.1%	0.1%	1.0%	-1.3%	-0.2%	-0.2%	-0.8%	-0.5%	-2.3%	-1.9%	-1.1%	-1.5%	-1.9%
	Indonesia	-2.1%	-1.6%	-2.1%	-2.1%	-2.1%	-1.8%	-2.6%	-2.7%	15.6%	12.2%	-2.9%	-2.9%	-1.7%	-1.6%	5,901.6%	7,410.9%	-2.6%	-1.8%	-1.6%	-1.7%	-0.0%	-0.1%	-0.6%	-0.1%	-0.3%	-0.4%	-0.9%	-1.6%	-2.7%	-1.3%	-1.9%	-1.8%	-2.9%
	Iran (Islamic Republic of)	-1.5%	-1.5%	0.2%	-0.3%	-1.1%	-0.7%	-3.3%	-3.3%	7.9%	21.1%	-3.0%	-3.0%	-1.2%	-1.4%	97.9%	35.4%	-2.3%	-2.4%	-1.7%	-2.1%	-0.0%	-0.0%	0.0%	0.2%	-0.1%	-0.1%	0.3%	0.6%	-1.9%	-1.3%	-1.6%	-1.2%	-2.9%
	Iraq	-1.5%	-1.4%	-1.0%	-1.0%	-1.8%	-1.6%	-3.3%	-3.3%	5.7%	15.9%	-2.9%	-2.9%	-1.6%	-1.7%	29.4%	25.2%	-2.7%	-2.5%	-1.1%	-1.6%	-0.0%	-0.1%	-0.6%	-0.1%	-0.0%	-0.1%	-0.5%	-0.6%	-2.0%	-1.6%	-1.8%	-1.6%	-2.1%
	Jamaica	0.3%	0.2%	0.3%	0.6%	-0.4%	-0.2%			10.2%	9.3%	-1.5%	-2.5%	-0.9%	-1.1%	1.5%	0.3%	-1.6%	-1.8%	-0.3%	0.3%	-0.0%	0.0%	-0.2%	0.1%	-0.1%	-0.1%	2.5%	6.1%	-0.7%	-0.9%	-1.6%	-1.5%	-2.5%
	Mexico	-1.2%	-1.0%	-2.5%	-2.3%	-1.6%	-1.0%	-3.2%	-3.2%	2.6%	3.0%	-2.4%	-2.5%	-1.3%	-1.1%	1.8%	0.7%	-2.6%	-2.5%	-1.4%	-1.6%	0.2%	0.3%	-0.3%	-0.8%	-0.3%	-0.3%	-1.3%	-1.1%	-1.0%	-0.8%	-1.9%	-1.7%	-2.1%
	Namibia	0.0%	0.1%	-2.0%	-1.6%	-1.4%	-0.5%	-2.6%	-2.7%	15.0%	17.4%	-3.2%	-3.2%	-1.5%	-0.9%	8.4%	35.5%	-1.6%	-1.3%	-1.4%	-1.1%	-0.1%	-0.1%	-0.8%	-0.1%	-0.2%	-0.3%	-0.9%	-0.7%	-1.2%	-0.5%	-1.3%	-1.0%	-2.6%
	Nauru	-0.5%	-0.6%	-1.3%	-1.2%	-0.6%	-0.7%			3.4%	3.6%	-2.8%	-2.8%	-1.2%	-1.4%	108.3%	48.1%	-1.5%	-1.7%	1.5%	5.9%	-0.0%	-0.0%	-0.1%	-0.1%	0.1%	0.1%	-0.4%	-0.2%	-1.3%	-1.3%	-0.3%	-0.4%	-1.6%
	Panama	-0.7%	0.6%	-1.0%	-0.9%	-0.0%	0.1%	-3.0%	-3.3%	1.8%	1.6%	-1.6%	-2.2%	-1.6%	-2.0%	2.3%	12.7%	-1.9%	-1.8%	-1.9%	-2.2%	-0.0%	0.0%	0.4%	0.2%	-0.2%	-0.2%	-0.4%	1.5%	-1.4%	-1.2%	-1.6%	-1.4%	-1.7%
	Paraguay	-0.9%	-0.3%	0.3%	-0.3%	-1.0%	0.2%	-3.3%	-3.3%	-2.5%	-1.7%	-2.5%	-2.6%	-1.7%	-1.3%	26.8%	16.9%	-2.2%	-1.4%	-2.1%	-1.5%	-0.0%	0.1%	-0.3%	-0.1%	-0.1%	-0.1%	-0.1%	1.4%	-1.2%	-0.8%	-0.8%	-0.5%	-2.9%
	Peru	-1.8%	-1.9%	-0.3%	-0.2%	-1.7%	-1.6%	-3.1%	-3.2%	14.8%	20.0%	-2.2%	-2.5%	-1.5%	-1.6%	5.3%	1.4%	-3.0%	-3.0%	-1.7%	-1.7%	-0.1%	-0.1%	-0.6%	-0.5%	-0.2%	-0.2%	-1.4%	-0.2%	-2.2%	-2.4%	-1.0%	-0.8%	-3.0%
	Philippines	-1.0%	-0.9%	-0.5%	-0.9%	-0.9%	-0.6%	-3.2%	-3.2%	5.7%	9.2%	-2.7%	-2.4%	-1.4%	-1.4%	11.0%	8.9%	-1.8%	-1.6%	-1.7%	-2.1%	-0.0%	-0.1%	1.2%	0.2%	-0.1%	-0.2%	-0.8%	3.4%	-2.2%	-1.8%	3.2%	2.4%	-2.1%
	Saint Lucia	-0.9%	-0.8%	1.1%	0.9%	-1.1%	-0.3%			5.0%	6.4%	-2.2%	-1.4%	-1.9%	-1.8%	-1.5%	-1.3%	-2.5%	-2.3%	-2.2%	-2.1%	0.1%	0.2%	0.1%	-1.0%	-0.1%	-0.2%	0.5%	0.4%	-1.8%	-1.6%	-1.5%	-1.1%	-2.4%
	Saint Vincent and the Grenadines	-0.5%	-0.5%	0.1%	0.5%	-0.8%	0.0%			2.1%	5.6%	-0.3%	-1.2%	-2.1%	-2.1%	0.6%	-0.5%	-2.4%	-1.9%	-1.6%	-1.5%	0.2%	0.3%	-0.1%	-2.0%	-0.1%	-0.2%	-0.4%	-0.2%	-2.1%	-1.2%	-1.3%	-1.0%	-1.4%
	Samoa	-0.3%	-0.7%	-1.1%	-1.2%	-0.5%	-0.9%			1.9%	2.2%	0.1%	-0.4%	-0.9%	-1.3%	24.4%	21.2%	-1.4%	-1.6%	0.2%	-1.4%	-0.0%	-0.0%	-0.1%	-0.1%	-0.1%	-0.1%	-0.3%	-0.4%	-0.8%	-1.3%	-0.4%	-0.8%	-2.6%
	South Africa	3.8%	4.6%	-1.9%	-1.2%	-2.3%	-0.5%	-1.1%	-0.3%	17.0%	18.0%	-2.6%	-2.4%	-1.8%	-0.3%	48.8%	150.4%	-1.5%	0.1%	-2.2%	-1.9%	-0.1%	-0.1%	-1.4%	-0.2%	-0.4%	-0.2%	0.8%	3.7%	-2.1%	-1.2%	-1.7%	-0.6%	-2.7%
	Suriname	-1.3%	-1.5%	-0.1%	-0.3%	-0.7%	-0.7%	-3.3%	-3.3%	6.9%	9.7%	-2.5%	-2.5%	-1.7%	-1.8%	-0.8%	-1.2%	-2.3%	-2.2%	-0.2%	0.0%	0.1%	0.3%	-0.2%	-0.7%	-0.1%	-0.1%	0.2%	0.9%	-2.2%	-2.1%	-1.2%	-1.1%	-1.5%
	Syrian Arab Republic	-1.2%	-1.0%	-0.8%	-1.1%	-0.5%	-0.2%	-3.3%	-3.3%	1.8%	3.0%	-3.0%	-3.0%	-1.8%	-1.6%	5.4%	18.9%	-2.6%	-2.6%	2.3%	4.9%	-0.0%	-0.0%	-0.8%	-0.2%	-0.1%	-0.1%	-0.9%	-0.9%	-1.8%	-1.8%	-2.5%	-2.5%	-2.9%
	Thailand	-1.0%	-1.2%	-1.9%	-1.7%	0.6%	0.2%	-3.3%	-3.3%	14.7%	8.6%	-2.9%	-2.9%	-1.7%	-1.7%	19.5%	14.3%	-2.8%	-2.9%	-2.4%	-2.9%	-0.3%	-0.5%	-0.9%	-0.2%	-0.2%	-0.2%	-1.6%	-1.3%	-2.2%	-1.8%	-1.9%	-2.3%	-1.9%
	Tokelau	-1.1%	-0.9%	-1.6%	-1.3%	-1.2%	-1.3%			2.7%	3.6%	-2.3%	-2.4%	-1.5%	-1.5%	109.9%	52.8%	-2.3%	-2.6%	-0.8%	1.6%	-0.0%	-0.0%	0.0%	0.0%	-0.2%	-0.2%	-0.7%	-0.5%	-1.3%	-1.0%	-1.3%	-1.3%	-2.5%
	Tonga	-0.7%	-0.4%	-0.3%	0.1%	-0.5%	0.9%			1.7%	2.0%	-1.7%	-1.4%	-1.1%	-0.0%	11.6%	12.6%	-1.3%	-0.8%	-1.2%	-1.7%	-0.0%	0.0%	-0.0%	0.1%	-0.1%	-0.1%	-0.3%	0.7%	-1.8%	-1.2%	-0.6%	-0.2%	-1.4%
	Tunisia	-0.9%	-0.5%	-0.8%	-0.9%	-1.2%	-0.7%			6.7%	7.0%	-2.5%	-2.5%	-1.2%	-1.2%	87.1%	87.1%	-2.4%	-2.3%	-1.3%	-1.3%	-0.0%	-0.0%	-0.1%	-0.1%	-0.1%	-0.2%	-0.1%	-0.0%	-1.6%	-1.4%	-2.5%	-2.2%	-3.0%
	Turkmenistan	-0.9%	-0.4%	0.1%	-0.5%	-1.0%	-0.2%	-3.3%	-3.3%	12.4%	18.2%	-1.5%	-1.0%	-0.6%	-0.6%	-1.0%	-0.5%	-0.6%	-0.1%	-1.9%	-1.3%	0.0%	-0.0%	-0.9%	-0.6%	-0.1%	-0.2%	-0.4%	0.4%	-1.8%	-0.1%	-1.4%	-1.6%	-1.0%
	Uzbekistan	-1.1%	-0.3%	0.4%	0.4%	0.2%	1.2%	-3.3%	-3.3%	14.8%	19.5%	-1.2%	-1.7%	-2.0%	-1.6%	2.7%	5.1%	-0.6%	-0.3%	-1.4%	-0.2%	-0.0%	-0.0%	-0.9%	-1.0%	-0.2%	-0.2%	-0.5%	-0.2%	-2.4%	-0.7%	-1.9%	-1.6%	-1.3%
	Viet Nam	-1.8%	-1.3%	-1.8%	-2.0%	-1.5%	-0.4%	-3.1%	-3.1%	4.0%	6.5%	-3.1%	-3.1%	-1.3%	-1.0%	16.6%	38.1%	-2.5%	-2.2%	-2.6%	-2.6%	-0.1%	-0.1%	0.0%	0.0%	-0.4%	-0.5%	-1.7%	-1.9%	-2.4%	-1.9%	-2.2%	-2.0%	-2.7%

15 to 24

High-middle SDI

Country	Total commuincable Female	Total commuincable Male	Enteric infections Female	Enteric infections Male	Lower respiratory infections Female	Lower respiratory infections Male	Malaria Female	Malaria Male	Neonatal sepsis and other neonatal infections Female	Neonatal sepsis and other neonatal infections Male	Vaccine Preventable disease Female	Vaccine Preventable disease Male	Meningitis & Encephalitis Female	Meningitis & Encephalitis Male
American Samoa	-0.3%	-0.6%	-1.0%	-0.3%	-0.2%	-0.8%			0.2%	-1.9%	-1.2%	-1.2%	0.7%	-0.7%
Antigua and Barbuda	-0.9%	-0.5%	0.8%	1.0%	-1.0%	-0.3%			-1.3%	0.6%	-1.3%	-2.4%	-2.0%	-2.2%
Argentina	-0.7%	-0.4%	0.1%	0.5%	1.9%	2.9%	-3.3%	-3.3%	-2.1%	-2.5%	-1.4%	-1.2%	-1.0%	-0.9%
Bahamas	0.0%	-0.5%	1.5%	1.3%	-0.2%	-0.5%			1.5%	-0.2%	-1.2%	-0.5%	-1.2%	-1.7%
Bahrain	-1.2%	-0.6%	-0.7%	-0.5%	-1.7%	-0.1%			4.2%	2.8%	-2.7%	-2.5%	-1.8%	-1.4%
Barbados	-0.1%	-0.7%	1.5%	1.1%	-0.6%	-0.9%			3.6%	4.1%	-1.9%	-2.1%	-1.7%	-2.1%
Belarus	-0.7%	-0.6%	0.2%	-0.1%	-2.2%	-1.2%			-0.9%	-0.0%	-0.5%	-0.3%	-1.0%	-1.0%
Bosnia and Herzegovina	-1.0%	-0.8%	-1.0%	-0.8%	-2.0%	-1.4%			-0.2%	-0.2%	-1.1%	-0.8%	-1.8%	-1.5%
Bulgaria	-1.0%	-1.2%	-0.7%	-0.7%	-2.1%	-2.0%			12.3%	23.1%	0.2%	0.2%	-0.8%	-0.7%
Chile	-1.3%	-1.3%	-0.5%	-0.9%	-2.6%	-2.7%			-0.2%	0.7%	-0.2%	-0.5%	-1.5%	-1.6%
Cook Islands	-1.3%	-1.3%	-0.9%	-0.7%	-2.0%	-1.5%			0.7%	1.7%	-1.8%	-1.7%	-1.1%	-0.9%
Croatia	-0.7%	-0.7%	-0.8%	-1.1%	-2.5%	-2.2%			1.9%	2.7%	-0.1%	-0.1%	-1.5%	-1.5%
Dominica	-0.5%	-0.6%	0.7%	0.5%	-0.4%	-0.5%			2.8%	2.4%	-1.8%	-1.6%	-1.6%	-1.8%
Georgia	-1.0%	-0.9%	0.9%	1.0%	-2.4%	-2.1%	-3.3%	-3.3%	1.0%	2.0%	0.2%	-0.1%	-1.4%	-0.8%
Greece	-0.3%	-0.5%	-0.6%	-0.5%	-0.6%	-1.0%			-1.2%	-1.5%	-0.3%	-0.2%	-1.2%	-0.9%
Greenland	-0.7%	-0.8%	0.1%	0.1%	-1.8%	-1.8%			3.5%	5.8%	-1.9%	-1.8%	-2.1%	-1.3%
Hungary	-1.0%	-0.9%	-0.6%	-0.7%	-2.1%	-2.1%			-2.3%	2.4%	-1.4%	-1.0%	-2.2%	-2.3%
Israel	-0.6%	-0.6%	-0.3%	-0.4%	-1.5%	-1.2%			-1.1%	-0.1%	-0.3%	0.0%	-1.6%	-1.9%
Italy	-0.7%	-1.2%	-0.6%	-0.6%	-2.0%	-2.2%			0.2%	1.5%	-1.0%	-0.6%	-1.4%	-1.5%
Jordan	-1.4%	-1.0%	-0.9%	-0.9%	-1.8%	-1.4%			4.0%	5.6%	-2.9%	-2.8%	-2.0%	-1.6%
Kazakhstan	-1.4%	-1.4%	0.7%	0.6%	-1.8%	-1.8%			1.3%	2.8%	-1.2%	-2.5%	-1.8%	-1.8%
Lebanon	-1.2%	-1.0%	-0.7%	-0.7%	-1.2%	-0.8%			5.2%	6.3%	-2.6%	-2.8%	-1.5%	-1.3%
Libya	-0.8%	-0.7%	-0.0%	-0.3%	-0.9%	-0.9%			1.3%	2.6%	-3.0%	-3.0%	-1.2%	-1.4%
Malaysia	-1.2%	-1.1%	-1.6%	-1.5%	-0.7%	-0.3%	-3.1%	-3.2%	-0.2%	-0.4%	-2.9%	-2.8%	-1.5%	-1.4%
Malta	-0.4%	-0.6%	-0.5%	-0.6%	-0.8%	-1.0%			0.2%	0.3%	-0.9%	-0.9%	-1.3%	-1.4%
Mauritius	-0.8%	0.0%	-1.0%	-0.8%	-2.1%	-1.2%			0.3%	2.9%	-0.6%	-0.4%	-1.5%	0.4%
Montenegro	-0.5%	-0.7%	-0.6%	-0.8%	-0.6%	-1.5%			-1.5%	-1.1%	-0.6%	-0.8%	-0.8%	-1.3%
Niue	-0.6%	-0.8%	-1.4%	-1.3%	-0.9%	-1.0%			2.2%	2.9%	-1.1%	-1.1%	-1.6%	-1.8%
North Macedonia	-0.8%	-0.8%	-0.6%	-0.8%	-1.9%	-1.6%			31.9%	48.9%	-0.7%	-0.7%	-1.2%	-1.5%
Northern Mariana Islands	-0.6%	-0.4%	-0.6%	-0.3%	-1.6%	-1.0%			3.5%	0.5%	-0.1%	-0.1%	-0.2%	-0.4%
Oman	-1.5%	-1.3%	-0.8%	-1.0%	-1.2%	-1.5%	-3.3%	-3.3%	5.3%	8.3%	-3.1%	-3.0%	-1.3%	-1.4%
Palau	-0.7%	-0.7%	-1.5%	-1.6%	-0.8%	-0.7%			2.5%	3.9%	-1.6%	-1.6%	-1.3%	-0.8%
Poland	-0.8%	-0.9%	-0.5%	-0.4%	-0.7%	-0.4%			-2.5%	-2.7%	-1.6%	-1.0%	-2.0%	-2.3%
Portugal	-0.9%	-1.5%	-0.5%	-0.5%	-1.8%	-2.2%			-1.7%	-1.1%	-0.4%	-1.5%	-2.1%	-2.1%
Republic of Moldova	-0.4%	-0.7%	0.4%	0.2%	-1.6%	-1.1%			2.6%	5.2%	-0.6%	-0.3%	-1.6%	-1.6%
Romania	-1.0%	-1.0%	-0.6%	-0.7%	-1.3%	-1.2%			-0.3%	2.9%	-1.4%	-1.1%	-2.0%	-2.2%
Russian Federation	0.3%	-0.1%	0.3%	0.2%	0.6%	0.2%			-0.0%	0.7%	-1.4%	-2.0%	-1.1%	-1.5%
Saint Kitts and Nevis	0.2%	1.0%	-0.4%	-0.3%	-1.4%	0.3%			3.8%	4.0%	-2.2%	-1.5%	-2.2%	-2.0%
Saudi Arabia	-1.8%	-1.3%	-1.7%	-1.3%	-1.2%	-0.5%	-3.3%	-3.2%	0.4%	1.5%	-3.2%	-3.2%	-1.9%	-1.6%
Serbia	-1.0%	-1.0%	-0.7%	-0.7%	-2.1%	-1.9%			-2.0%	-1.6%	-1.7%	-1.5%	-1.5%	-1.5%
Seychelles	-0.8%	-0.5%	-1.5%	-1.5%	-1.0%	-0.0%			1.9%	1.5%	-2.6%	-2.6%	-2.5%	-2.3%
Spain	-1.2%	-2.0%	-0.5%	-0.6%	-2.3%	-2.6%			0.3%	1.2%	-0.7%	-0.8%	-2.0%	-2.2%
Sri Lanka	-1.7%	-1.6%	-1.9%	-2.2%	-2.0%	-1.6%	-3.3%	-3.3%	4.9%	5.0%	-3.0%	-3.1%	-1.9%	-2.1%
Trinidad and Tobago	-0.3%	-0.6%	0.5%	0.3%	-1.8%	-1.4%			0.9%	2.6%	0.4%	-2.1%	-2.2%	-2.2%
Turkey	-1.6%	-1.4%	-1.1%	-1.3%	-1.4%	-1.5%	-3.3%	-3.3%	8.6%	13.1%	-3.1%	-3.1%	-2.3%	-2.3%
Ukraine	0.7%	1.4%	0.5%	0.4%	1.1%	2.9%			-1.9%	-1.2%	0.4%	-0.6%	-0.1%	-0.2%
United States Virgin Islands	-0.2%	-0.6%	1.5%	1.1%	-1.6%	-1.8%			0.4%	2.5%	-1.2%	-1.2%	-1.4%	-2.2%
Uruguay	-0.5%	0.1%	-0.1%	0.5%	-0.5%	0.7%			-1.3%	-0.2%	-0.1%	-0.9%	-1.5%	-1.7%

Country	HIV/AIDS Female	HIV/AIDS Male	Tuberculosis Female	Tuberculosis Male	Neglected Tropical diseases Female	Neglected Tropical diseases Male	Infectious skin conditions Female	Infectious skin conditions Male	Sexually transmitted infections excluding HIV Female	Sexually transmitted infections excluding HIV Male	Upper respiratory infections Female	Upper respiratory infections Male	Other unspecified infectious diseases Female	Other unspecified infectious diseases Male	Hepatitis Female	Hepatitis Male	Rheumatic heart disease Female	Rheumatic heart disease Male	Maternal sepsis and other maternal infections Female
American Samoa	14.6%	5.3%	-1.3%	-2.4%	1.4%	-1.6%	-0.0%	0.2%	-0.3%	-0.0%	0.0%	0.1%	-0.3%	1.4%	-0.0%	-0.0%	-0.4%	-1.0%	-2.0%
Antigua and Barbuda	-1.1%	-0.3%	-2.1%	-2.3%	-1.2%	0.1%	0.0%	0.2%	-0.0%	-0.8%	-0.1%	-0.1%	0.6%	0.6%	-2.4%	-2.0%	-1.2%	-0.9%	-1.8%
Argentina	0.6%	-0.7%	-2.2%	-2.1%	-2.6%	-2.4%	0.0%	0.1%	-0.2%	-0.6%	-0.1%	-0.1%	-0.2%	-0.1%	-1.5%	-0.8%	-0.9%	-0.9%	-2.1%
Bahamas	0.5%	-0.1%	-2.0%	-1.8%	-0.1%	-0.1%	0.1%	0.1%	0.3%	-0.2%	-0.0%	-0.0%	0.8%	0.7%	-1.8%	-1.5%	-0.7%	-0.8%	-1.8%
Bahrain	2.5%	0.3%	-2.6%	-2.2%	-1.7%	-1.9%	-0.4%	-0.3%	-0.4%	-0.3%	-0.0%	-0.0%	-1.3%	-0.2%	-1.9%	-1.0%	-2.2%	-2.1%	-2.7%
Barbados	-0.1%	-1.9%	-2.2%	-2.3%	0.3%	2.2%	0.1%	0.1%	0.1%	-0.6%	-0.1%	-0.0%	0.1%	0.2%	-2.1%	-1.9%	-1.0%	-0.9%	-1.8%
Belarus	4.8%	3.5%	-2.1%	-1.6%	-1.2%	-1.3%	-0.0%	-0.0%	-0.3%	-1.0%	-0.2%	-0.2%	0.3%	0.4%	-0.8%	0.3%	-2.8%	-2.7%	-1.9%
Bosnia and Herzegovina	5.2%	-0.0%	-2.6%	-2.1%	-1.6%	-1.8%	-0.0%	-0.0%	-0.3%	0.1%	-0.3%	-0.3%	-1.1%	-0.3%	-1.3%	-0.8%	-2.5%	-2.1%	-2.8%
Bulgaria	5.0%	2.6%	-1.7%	-1.7%	-1.7%	-2.4%	0.1%	0.0%	-0.4%	0.0%	-0.1%	-0.1%	-0.3%	-0.7%	-1.1%	-0.6%	-2.5%	-2.5%	-2.3%
Chile	4.7%	2.8%	-2.5%	-2.6%	-2.5%	-2.5%	-0.0%	-0.0%	-0.3%	-0.7%	-0.2%	-0.3%	0.3%	0.5%	-1.6%	-1.4%	-2.6%	-2.7%	-2.8%
Cook Islands	110.9%	49.5%	-2.2%	-2.0%	-2.5%	-2.9%	-0.0%	-0.0%	0.0%	0.0%	-0.2%	-0.2%	-0.6%	-1.1%	-1.5%	-1.2%	-2.0%	-1.7%	-1.4%
Croatia	4.6%	0.9%	-2.7%	-2.9%	-1.0%	-1.4%	0.0%	-0.0%	-0.1%	0.1%	-0.1%	-0.1%	0.2%	-0.1%	-1.4%	-1.4%	-2.8%	-2.7%	-2.2%
Dominica	0.1%	-0.7%	-2.0%	-1.7%	-1.2%	-1.0%	0.0%	-0.0%	0.2%	-0.9%	-0.2%	-0.2%	0.9%	1.1%	-2.3%	-1.9%	-1.1%	-1.0%	-1.0%
Georgia	20.8%	30.7%	-1.5%	-1.5%	-0.7%	0.1%	0.0%	-0.0%	-0.0%	-0.0%	0.0%	-0.0%	-0.2%	1.2%	-1.3%	0.0%	-1.0%	-0.8%	0.1%
Greece	1.9%	-1.5%	-2.1%	-2.2%	-0.7%	-1.0%	0.0%	-0.0%	-0.1%	0.0%	-0.1%	-0.1%	0.9%	0.6%	-0.6%	-0.7%	-2.0%	-2.4%	-2.2%
Greenland	-1.6%	-2.7%	-1.7%	-0.8%	-1.0%	-1.2%	0.1%	0.1%	-0.4%	0.3%	-0.0%	-0.1%	-0.6%	0.2%	-1.0%	0.7%	-2.5%	-2.0%	-1.7%
Hungary	-2.6%	-2.6%	-2.6%	-2.5%	-1.1%	-1.3%	0.0%	0.0%	-0.2%	0.0%	-0.2%	-0.2%	-0.4%	-0.1%	-1.9%	-1.9%	-2.6%	-2.8%	-2.2%
Israel	-1.1%	-1.2%	-2.4%	-2.7%	-1.3%	-1.3%	0.0%	0.1%	-0.2%	-0.7%	-0.1%	-0.2%	0.4%	0.8%	-1.8%	-1.2%	-2.1%	-1.8%	-2.1%
Italy	-2.6%	-3.0%	-2.2%	-2.3%	1.9%	-2.0%	0.0%	0.0%	0.8%	-2.1%	-0.1%	-0.1%	0.8%	0.5%	-1.1%	-1.6%	-1.9%	-2.1%	-1.5%
Jordan	20.0%	27.9%	-2.9%	-2.8%	-1.6%	-2.1%	-0.0%	-0.0%	-0.2%	-0.2%	-0.1%	-0.1%	-1.2%	-1.4%	-1.9%	-1.8%	-2.7%	-2.3%	-2.6%
Kazakhstan	7.0%	1.8%	-2.1%	-2.3%	-1.8%	-0.9%	0.0%	-0.0%	0.1%	-0.5%	-0.2%	-0.1%	0.1%	0.3%	-2.1%	-1.2%	-2.9%	-2.8%	-0.7%
Lebanon	4.8%	0.6%	-2.6%	-2.4%	-2.1%	-2.5%	-0.0%	-0.0%	-0.1%	0.0%	-0.0%	-0.1%	-0.9%	-0.5%	-1.9%	-1.3%	-2.6%	-2.3%	-2.5%
Libya	33.5%	22.8%	-2.2%	-2.1%	-1.4%	-0.8%	-0.0%	-0.0%	-0.1%	-0.1%	0.1%	0.1%	0.9%	-0.3%	-1.9%	-1.5%	-1.6%	-1.3%	-2.9%
Malaysia	74.4%	21.3%	-1.8%	-1.9%	-1.7%	-1.9%	0.0%	0.0%	-0.4%	0.0%	-0.2%	-0.2%	-0.6%	3.7%	-1.1%	-0.7%	-2.1%	-2.1%	-2.9%
Malta	-0.3%	-1.9%	-1.2%	-1.0%	-1.6%	-1.8%	0.0%	0.1%	0.0%	-0.4%	-0.1%	-0.2%	0.5%	0.1%	-0.4%	-0.3%	-1.0%	-1.6%	-1.6%
Mauritius	7.2%	32.0%	-2.1%	-1.5%	-0.4%	-0.0%	0.0%	0.0%	-0.5%	-0.2%	-0.2%	-0.2%	-0.2%	0.6%	-1.9%	-1.2%	-2.2%	-1.6%	-2.2%
Montenegro	9.0%	8.8%	-1.7%	-2.2%	-0.4%	-0.6%	-0.0%	-0.0%	-0.2%	-0.0%	-0.0%	0.0%	-0.2%	-0.5%	-0.4%	-1.0%	-1.5%	-2.1%	-1.8%
Niue	101.9%	48.5%	-1.9%	-2.1%	0.5%	-0.7%	-0.0%	-0.0%	-0.0%	-0.0%	-0.1%	-0.1%	-0.7%	-0.3%	-1.5%	-1.5%	-1.0%	-0.9%	-1.9%
North Macedonia	22.7%	12.3%	-2.9%	-2.7%	-0.8%	-1.3%	0.0%	-0.0%	-0.2%	0.1%	-0.0%	-0.1%	-0.4%	-1.0%	-1.0%	-0.8%	-2.7%	-2.3%	-2.5%
Northern Mariana Islands	19.1%	11.8%	-1.9%	-2.5%	1.4%	3.7%	0.0%	0.0%	-0.3%	-0.2%	0.1%	0.1%	0.4%	1.2%	-1.3%	-1.2%	-1.2%	-0.4%	-2.0%
Oman	32.0%	24.3%	-2.6%	-2.6%	-2.4%	-2.0%	-0.1%	-0.0%	-0.1%	0.1%	-0.0%	-0.1%	-1.5%	-1.6%	-2.1%	-1.8%	-2.4%	-2.5%	-2.8%
Palau	97.8%	45.2%	-1.6%	-1.9%	0.8%	2.9%	-0.0%	-0.0%	-0.1%	0.1%	-0.1%	-0.1%	-0.3%	-0.2%	-1.3%	-1.5%	-1.3%	-1.0%	-1.5%
Poland	17.9%	15.8%	-2.5%	-2.5%	-1.3%	-1.4%	-0.0%	-0.1%	0.1%	-0.1%	-0.2%	-0.3%	-1.1%	-1.1%	-1.5%	-1.5%	-2.8%	-2.9%	-2.4%
Portugal	1.3%	-1.9%	-2.5%	-2.8%	-1.7%	-1.7%	-0.0%	-0.0%	-0.2%	-2.2%	-0.1%	-0.3%	-0.5%	-0.7%	-1.9%	-2.1%	-2.7%	-2.8%	-2.1%
Republic of Moldova	22.7%	2.7%	-1.4%	-1.4%	-1.1%	-1.7%	-0.0%	-0.2%	-1.5%	-0.3%	0.0%	0.0%	0.4%	0.8%	-2.9%	-2.7%	-2.8%	-2.9%	-2.6%
Romania	4.2%	11.8%	-1.6%	-1.6%	-1.7%	-2.3%	0.0%	0.0%	-1.0%	-0.0%	-0.2%	-0.2%	-1.5%	-1.7%	-2.6%	-2.0%	-2.8%	-2.6%	-2.5%
Russian Federation	10.4%	6.1%	-0.5%	-1.4%	-0.6%	-1.1%	-0.0%	0.0%	-0.6%	-0.5%	-0.1%	-0.1%	0.3%	0.1%	-1.2%	-0.8%	-2.7%	-2.8%	-2.3%
Saint Kitts and Nevis	20.3%	9.6%	-2.5%	-2.1%	0.0%	-0.5%	0.0%	0.2%	-0.1%	-0.7%	-0.2%	-0.2%	-0.3%	0.4%	-2.7%	-1.5%	-2.3%	-1.5%	-1.8%
Saudi Arabia	9.7%	10.9%	-2.6%	-2.3%	-2.0%	-2.1%	-0.1%	-0.0%	-0.2%	0.0%	-0.0%	-0.0%	0.3%	-0.5%	-1.9%	-1.5%	-2.7%	-2.5%	-2.9%
Serbia	-1.6%	0.1%	-2.6%	-2.8%	-1.8%	-2.3%	-0.0%	-0.0%	-0.3%	0.0%	-0.1%	-0.1%	-0.9%	-0.8%	-1.9%	-1.3%	-2.7%	-2.6%	-2.4%
Seychelles	34.9%	15.6%	-1.7%	-1.8%	1.5%	2.7%	0.0%	0.0%	-1.4%	0.1%	-0.2%	-0.2%	-0.3%	4.5%	-2.0%	-1.4%	-1.9%	-2.1%	-1.9%
Spain	-3.0%	-3.2%	-2.7%	-3.0%	-1.5%	-2.2%	0.0%	-0.0%	-0.6%	-2.8%	-0.1%	-0.2%	-0.3%	-0.2%	-1.6%	-2.3%	-2.3%	-2.6%	-1.7%
Sri Lanka	6.1%	12.0%	-2.5%	-2.3%	-1.7%	-1.3%	-0.0%	-0.1%	-0.3%	-0.1%	-0.3%	-0.3%	-1.5%	-0.3%	-2.0%	-1.8%	-2.6%	-2.5%	-2.7%
Trinidad and Tobago	3.7%	-0.0%	-2.2%	-1.3%	-0.7%	1.9%	-0.0%	0.0%	0.2%	0.1%	-0.2%	-0.2%	0.7%	0.8%	-2.3%	-2.0%	-1.4%	-1.4%	-2.4%
Turkey	83.5%	98.4%	-2.8%	-2.7%	-2.5%	-2.9%	-0.1%	-0.1%	-0.8%	-0.3%	-0.1%	-0.2%	-1.9%	-1.5%	-2.7%	-2.6%	-2.6%	-2.2%	-3.0%
Ukraine	18.9%	3.9%	3.8%	7.0%	0.5%	0.5%	0.1%	0.2%	0.1%	-1.2%	0.0%	0.1%	2.2%	1.6%	0.4%	3.3%	0.3%	0.8%	-1.9%
United States Virgin Islands	1.1%	-0.2%	-2.4%	-1.6%	0.3%	0.1%	0.0%	0.1%	-0.1%	-1.2%	-0.1%	-0.2%	0.2%	-0.9%	-1.9%	-2.0%	-2.4%	-2.3%	-2.1%
Uruguay	5.1%	9.8%	-1.9%	-1.7%	-1.8%	-1.8%	-0.0%	-0.0%	-0.6%	-2.3%	-0.1%	-0.1%	-1.1%	-0.7%	-1.7%	-0.6%	-1.8%	-1.3%	-1.6%

15 to 24

SDI	Country	Total communicable Female	Total communicable Male	Enteric infections Female	Enteric infections Male	Lower respiratory infections Female	Lower respiratory infections Male	Malaria Female	Malaria Male	Neonatal sepsis and other neonatal infections Female	Neonatal sepsis and other neonatal infections Male	Vaccine Preventable disease Female	Vaccine Preventable disease Male	Meningitis & Encephalitis Female	Meningitis & Encephalitis Male	HIV/AIDS Female	HIV/AIDS Male	Tuberculosis Female	Tuberculosis Male	Neglected Tropical diseases Female	Neglected Tropical diseases Male	Infectious skin conditions Female	Infectious skin conditions Male	Sexually transmitted infections excluding HIV Female	Sexually transmitted infections excluding HIV Male	Upper respiratory infections Female	Upper respiratory infections Male	Other unspecified infectious diseases Female	Other unspecified infectious diseases Male	Hepatitis Female	Hepatitis Male	Rheumatic heart disease Female	Rheumatic heart disease Male	Maternal sepsis and other maternal infections Female
High SDI	Andorra	-0.3%	-0.3%	-0.3%	-0.3%	-1.1%	-1.5%			-0.6%	-0.2%	-1.3%	-1.3%	-0.9%	-1.3%	2.8%	0.6%	-2.0%	-1.8%	-1.0%	-0.6%	0.0%	-0.0%	-0.2%	0.1%	-0.0%	-0.1%	-0.3%	-0.5%	-1.9%	-1.6%	-1.8%	-1.6%	-1.8%
	Australia	-0.3%	-0.6%	0.1%	0.3%	-1.3%	-1.8%			-0.4%	-0.4%	-0.7%	0.8%	-1.7%	-1.9%	0.3%	-2.9%	-1.4%	-1.4%	-0.3%	-0.5%	-0.0%	-0.0%	-0.5%	-0.8%	-0.0%	-0.1%	0.5%	0.1%	-0.7%	-0.8%	-1.9%	-2.0%	-0.9%
	Austria	-0.4%	-0.7%	-0.3%	-0.4%	-2.0%	-2.2%			1.2%	1.1%	-0.1%	0.1%	-1.3%	-1.6%	-0.6%	-1.8%	-2.2%	-2.0%	-0.7%	-1.3%	0.0%	0.0%	-0.2%	-0.2%	-0.1%	-0.1%	2.0%	1.5%	-0.6%	-1.1%	-2.0%	-2.0%	-1.9%
	Belgium	-0.4%	-0.6%	-0.4%	-0.5%	-1.1%	-1.6%			-0.4%	-0.8%	-0.3%	-0.5%	-1.2%	-1.2%	-1.6%	-1.9%	-1.8%	-1.6%	-0.2%	-1.5%	0.0%	0.0%	-0.3%	-0.8%	-0.1%	-0.1%	1.3%	0.6%	-1.0%	-0.9%	-0.8%	-1.5%	-1.9%
	Bermuda	-0.8%	-1.2%	1.6%	1.4%	-1.7%	-1.3%			-2.0%	0.8%	-0.4%	-0.4%	-2.0%	-2.2%	-0.4%	-2.1%	-2.3%	-2.3%	-1.2%	-0.1%	0.0%	0.0%	-0.4%	-0.4%	-0.0%	-0.1%	-0.6%	-0.1%	-1.7%	-1.6%	-2.6%	-2.4%	-2.7%
	Brunei Darussalam	-0.8%	-1.6%	-0.8%	-1.0%	0.3%	-1.8%			5.0%	5.0%	-0.8%	-1.8%	-1.7%	-2.3%	12.0%	21.2%	-2.4%	-2.8%	-0.7%	-2.2%	-0.0%	-0.0%	-0.4%	-0.1%	-0.0%	-0.0%	0.2%	-1.1%	-1.6%	-2.2%	-1.8%	-2.6%	-2.3%
	Canada	-0.1%	-0.4%	0.8%	0.9%	-1.3%	-1.8%			3.4%	-0.8%	-0.2%	-0.3%	-1.5%	-1.4%	0.8%	-2.7%	-1.2%	-1.1%	-0.6%	-1.2%	-0.0%	-0.0%	-0.2%	-0.0%	-0.1%	-0.1%	0.0%	-0.1%	-0.3%	-0.5%	-1.4%	-1.5%	-1.8%
	Cyprus	-0.3%	-0.5%	-0.7%	-0.8%	-1.7%	-1.3%			0.1%	0.2%	-1.5%	-1.4%	-2.1%	-1.8%	80.0%	10.6%	-1.9%	-2.4%	-1.5%	-2.3%	-0.0%	-0.0%	-0.1%	0.0%	-0.1%	-0.1%	-0.4%	-0.4%	-0.9%	-1.0%	-2.2%	-1.9%	-3.0%
	Czechia	-0.5%	-0.5%	-0.6%	-0.7%	-1.2%	-0.9%			2.8%	7.0%	0.1%	-0.1%	-1.6%	-1.3%	13.8%	9.0%	-2.5%	-2.4%	-1.2%	-1.2%	0.0%	0.0%	-0.3%	-0.1%	-0.1%	-0.2%	-0.5%	-0.4%	-1.1%	-1.2%	-2.5%	-2.7%	-2.4%
	Denmark	-0.5%	-0.6%	-0.4%	-0.4%	-1.2%	-1.4%			0.4%	0.7%	-0.1%	-0.9%	-2.3%	-2.4%	12.6%	2.4%	-1.9%	-1.7%	-1.0%	-0.3%	0.0%	-0.0%	-0.3%	-0.0%	-0.1%	-0.1%	0.3%	0.4%	-1.0%	-0.1%	-2.2%	-2.6%	-1.8%
	Estonia	0.2%	-0.7%	1.2%	-0.1%	-0.7%	-1.4%			-1.1%	-2.8%	-0.4%	-0.5%	-1.9%	-2.1%	407.4%	247.7%	-2.2%	-2.5%	-0.4%	-1.4%	-0.2%	-0.2%	-1.1%	-2.1%	-0.1%	-0.1%	0.5%	0.5%	-1.7%	-1.2%	-2.3%	-3.0%	-2.5%
	Finland	-0.6%	-0.8%	-0.5%	-0.6%	-2.6%	-2.8%			-0.5%	0.6%	0.1%	-0.4%	-1.6%	-2.1%	-2.0%	-1.8%	-1.7%	-1.7%	-0.9%	0.0%	-0.0%	-0.0%	-0.6%	-0.2%	-0.1%	-0.1%	-0.1%	-0.4%	-0.9%	-0.2%	-2.2%	-2.4%	-2.2%
	France	-0.6%	-1.2%	-0.5%	-0.5%	-1.9%	-2.1%			-1.3%	-1.5%	-0.9%	-1.4%	-1.7%	-1.9%	-2.5%	-3.1%	-2.1%	-2.2%	-1.1%	-2.1%	0.0%	-0.0%	-0.2%	-0.8%	-0.1%	-0.2%	-0.1%	-0.4%	-0.6%	-1.1%	-1.6%	-1.9%	-1.6%
	Germany	-0.6%	-0.9%	-0.2%	-0.3%	-1.4%	-1.6%			-0.3%	-0.2%	-0.1%	-0.4%	-1.7%	-1.7%	-2.8%	-2.8%	-2.3%	-2.1%	-0.0%	-1.5%	0.0%	-0.0%	-0.6%	-0.8%	-0.1%	-0.1%	-0.3%	-0.5%	-1.3%	-1.4%	-1.7%	-2.1%	-2.6%
	Guam	-0.3%	-0.4%	-0.4%	-0.3%	-0.5%	-1.1%			0.4%	1.2%	-0.9%	-0.8%	-0.2%	-1.2%	15.8%	7.4%	-1.0%	-1.9%	-0.9%	-0.1%	0.1%	0.0%	-0.1%	-0.1%	-0.1%	-0.0%	0.4%	1.0%	-0.3%	-0.7%	-0.9%	-1.1%	-1.3%
	Iceland	-0.5%	-0.9%	-0.5%	-0.5%	-1.2%	-2.1%			0.3%	-0.6%	0.4%	0.2%	-1.7%	-2.0%	-2.4%	-2.3%	-2.3%	-2.2%	-0.2%	-0.6%	-0.0%	0.0%	-0.2%	-1.6%	-0.1%	-0.2%	0.4%	-0.5%	0.2%	-0.2%	-1.4%	-2.2%	-2.0%
	Ireland	-0.8%	-0.7%	-0.6%	-0.6%	-2.3%	-2.2%			0.6%	1.7%	-0.3%	-0.5%	-2.4%	-2.1%	0.2%	-1.3%	-1.8%	-2.1%	-1.7%	0.5%	-0.0%	-0.0%	-0.2%	-0.2%	-0.1%	-0.2%	-0.1%	-0.2%	-0.4%	0.0%	-2.4%	-2.1%	-1.3%
	Japan	-0.5%	-0.7%	-0.8%	-0.8%	-1.9%	-2.0%			-0.7%	-0.5%	-1.3%	-1.3%	-1.6%	-1.7%	6.8%	5.8%	-2.1%	-2.2%	-1.2%	-0.7%	-0.0%	-0.0%	0.6%	-0.0%	-0.1%	-0.1%	-0.6%	-0.2%	-1.1%	-1.5%	-1.8%	-2.1%	-1.2%
	Kuwait	-1.0%	-0.9%	-0.3%	-0.5%	-1.2%	-1.1%			-1.9%	-0.1%	-0.8%	-0.6%	-1.5%	-1.5%	0.0%	-1.3%	-2.7%	-2.6%	-0.8%	-0.8%	-0.0%	0.0%	-0.2%	-0.3%	-0.0%	-0.0%	-0.7%	-0.2%	-1.6%	-1.8%	-2.9%	-2.6%	-1.9%
	Latvia	-0.7%	-0.9%	-0.3%	-0.3%	-0.7%	-1.1%			-2.0%	1.3%	0.7%	0.8%	-1.9%	-2.1%	9.2%	2.1%	-2.3%	-2.2%	-0.7%	-0.5%	-0.0%	0.0%	-0.4%	-0.6%	-0.1%	-0.1%	0.8%	0.4%	-2.3%	-1.9%	-2.8%	-3.0%	-2.5%
	Lithuania	-0.2%	-0.5%	0.5%	-0.2%	0.3%	0.2%			8.1%	6.1%	-0.1%	-0.2%	-1.7%	-1.8%	0.8%	19.7%	-1.8%	-2.0%	-0.4%	-1.1%	0.1%	0.0%	-0.3%	-0.6%	-0.1%	-0.1%	0.5%	0.1%	-1.2%	-0.5%	-2.8%	-2.9%	-2.5%
	Luxembourg	-0.4%	-1.1%	-0.3%	-0.4%	-1.4%	-2.2%			-0.2%	0.1%	-1.0%	-1.8%	-1.3%	-2.1%	-1.4%	-2.7%	-1.8%	-2.2%	-1.0%	-1.3%	0.0%	-0.0%	-0.4%	-2.5%	-0.1%	-0.2%	-0.1%	-0.6%	-0.6%	-1.6%	-1.6%	-2.4%	-2.4%
	Monaco	-0.3%	-0.6%	-0.3%	-0.3%	-0.8%	-0.9%			-0.5%	0.3%	-0.3%	-0.1%	-1.3%	-1.5%	0.2%	-1.6%	-2.0%	-2.2%	-0.4%	-0.6%	0.0%	-0.0%	-0.4%	-0.3%	-0.1%	-0.1%	1.4%	0.6%	0.1%	-0.1%	-1.0%	-1.4%	-2.0%
	Netherlands	-0.4%	-0.6%	-0.3%	-0.3%	-1.1%	-1.2%			-0.4%	-0.8%	-0.1%	0.1%	-1.8%	-1.8%	-1.3%	-2.8%	-1.9%	-1.9%	-0.8%	1.5%	0.0%	-0.0%	-0.4%	-0.2%	-0.1%	-0.2%	0.8%	0.6%	-0.1%	0.1%	-1.6%	-1.8%	-1.6%
	New Zealand	-0.5%	-0.8%	0.1%	-0.2%	-2.4%	-2.5%			2.0%	2.0%	-0.5%	-0.9%	-1.5%	-1.5%	-1.9%	-2.7%	-1.8%	-1.8%	0.3%	-1.3%	-0.0%	-0.0%	0.5%	-0.1%	-0.1%	-0.1%	0.4%	-0.2%	-0.9%	-0.6%	-2.2%	-2.3%	-1.2%
	Norway	-0.6%	-1.0%	-0.5%	-0.3%	-2.4%	-2.3%			-0.1%	-0.0%	-0.0%	-0.1%	-2.3%	-2.2%	6.8%	-2.4%	-1.8%	-2.2%	-0.5%	0.1%	-0.0%	0.0%	-0.3%	0.7%	-0.1%	-0.2%	0.7%	0.9%	-0.4%	-0.3%	-2.3%	-2.4%	-2.2%
	Puerto Rico	-1.1%	-1.1%	1.6%	1.7%	-2.1%	-1.8%			0.1%	2.9%	-1.8%	-0.7%	-2.3%	-2.4%	-2.5%	-2.7%	-2.5%	-2.4%	-1.2%	-0.3%	-0.0%	0.0%	-0.2%	0.1%	-0.1%	-0.1%	0.5%	1.0%	-1.8%	-1.5%	-2.6%	-1.3%	-2.6%
	Qatar	-1.4%	-1.2%	-1.3%	-1.4%	-1.5%	-1.5%			1.8%	4.0%	-3.0%	-2.9%	-1.8%	-1.4%	-1.8%	-0.0%	-2.7%	-2.5%	-1.5%	-2.0%	-0.0%	-0.1%	-0.1%	0.1%	-0.1%	-0.1%	-1.0%	-1.0%	-1.1%	-1.5%	-2.7%	-2.8%	-2.9%
	Republic of Korea	-1.9%	-1.9%	-1.6%	-2.0%	-2.8%	-2.7%	-3.2%	-3.2%	0.1%	2.3%	-1.8%	-1.9%	-2.5%	-2.6%	0.7%	1.2%	-3.1%	-3.2%	-1.9%	-1.1%	-0.1%	-0.1%	-0.8%	-0.1%	-0.2%	-0.3%	-1.9%	-1.9%	-2.7%	-2.6%	-2.8%	-2.7%	-3.2%
	San Marino	-0.1%	-0.4%	-0.4%	-0.4%	1.1%	-0.9%			-0.2%	0.2%	0.4%	0.4%	-0.6%	-0.6%	0.4%	-1.7%	-1.7%	-1.9%	-0.3%	-0.5%	0.0%	-0.0%	-0.1%	0.0%	-0.0%	-0.1%	0.7%	0.1%	-0.5%	-1.3%	-0.7%	-1.0%	-1.7%
	Singapore	-1.0%	-1.2%	-1.9%	-1.8%	-1.9%	-1.9%			5.7%	4.1%	-0.7%	-1.1%	-2.1%	-1.9%	2.3%	2.0%	-2.6%	-2.6%	-0.8%	-0.4%	0.0%	0.0%	-0.6%	-0.3%	-0.1%	-0.2%	-1.4%	-1.3%	-1.4%	-1.6%	-2.9%	-2.7%	-2.7%
	Slovakia	-1.0%	-1.0%	-0.8%	-0.8%	-2.0%	-2.1%			-0.1%	1.9%	0.2%	0.1%	-1.6%	-1.2%	18.5%	13.9%	-2.5%	-2.5%	-1.0%	-1.1%	-0.0%	-0.0%	-0.2%	0.2%	-0.1%	-0.2%	-0.2%	-0.5%	-0.8%	-1.2%	-1.7%	-2.1%	-2.1%
	Slovenia	-0.6%	-0.5%	-0.6%	-0.6%	-1.7%	-1.8%			-0.5%	1.3%	0.1%	-0.2%	-1.5%	-1.4%	-1.6%	-1.5%	-2.4%	-2.3%	-1.3%	-2.0%	0.0%	0.0%	-0.3%	-0.2%	-0.1%	-0.1%	-0.0%	0.2%	-1.1%	-1.0%	-1.8%	-2.2%	-2.3%
	Sweden	-0.4%	-0.6%	-0.3%	-0.3%	-2.0%	-2.2%			-0.5%	-0.8%	-0.0%	-0.1%	-1.7%	-1.7%	0.2%	-2.3%	-1.6%	-1.7%	0.3%	-0.6%	0.0%	0.0%	-0.4%	-0.6%	-0.1%	-0.1%	1.0%	0.3%	-1.0%	-0.4%	-2.2%	-2.2%	-1.9%
	Switzerland	-0.7%	-1.0%	-0.3%	-0.3%	-2.5%	-2.6%			-2.2%	0.4%	-2.3%	-1.8%	-2.5%	-2.7%	-0.3%	-1.7%	-2.1%	-1.5%	1.1%	-1.2%	0.0%	0.0%	-0.1%	-0.2%	-0.0%	-0.1%	0.4%	-0.5%	-1.4%	-1.3%	-2.2%	-2.7%	-2.0%
	Taiwan (Province of China)	-0.7%	-0.8%	-0.5%	-0.4%	-1.6%	-1.5%			9.6%	8.1%	-0.9%	-1.0%	-1.9%	-2.1%	9.6%	35.2%	-1.7%	-1.7%	-1.4%	-1.3%	0.0%	0.0%	-0.6%	0.0%	-0.3%	-0.4%	-0.1%	1.0%	-1.6%	-2.0%	-2.8%	-2.8%	-2.5%
	United Arab Emirates	-0.9%	-0.9%	-0.7%	-0.7%	-1.3%	-1.2%	-3.3%	-3.3%	1.7%	6.3%	-2.8%	-2.7%	-1.6%	-1.6%	34.3%	16.8%	-2.4%	-2.4%	-0.2%	-1.2%	0.0%	0.0%	-0.3%	-0.2%	0.1%	0.1%	-0.0%	-0.5%	-1.4%	-1.4%	-0.7%	-1.3%	-2.9%
	United Kingdom	-0.4%	-0.7%	-0.4%	-0.4%	-1.1%	-1.5%			-0.0%	0.2%	-1.0%	-0.9%	-1.7%	-1.8%	2.6%	-2.1%	-1.7%	-1.8%	-1.0%	-1.3%	0.1%	0.0%	0.6%	-0.6%	-0.1%	-0.1%	-0.2%	-0.6%	0.5%	0.7%	-2.2%	-2.2%	-1.6%
	United States of America	-0.5%	-1.0%	1.2%	1.0%	-1.1%	-1.1%			-0.8%	-1.0%	-0.5%	-0.7%	-1.5%	-1.7%	-2.3%	-2.8%	-2.3%	-2.0%	0.0%	-0.1%	-0.1%	-0.1%	-0.5%	-0.7%	-0.1%	-0.1%	-0.6%	-1.0%	-0.5%	-0.6%	-2.1%	-2.0%	-1.8%

S15. (A) Percentage change in DALYs/100,000 for each communicable condition for each location for 0-24 year old age group by sex. The colours on this heat map are green for values less than 0, the darker the green the greater the reduction in DALYs/100,000 between 1990 and 2019, the orange tones represent positive numbers, an increase in DALYs/ 100,000, with darker shades indicating worse outcome.

0 to 24 years, percentage change in DALYs/100,000, for males and females, 1990 to 2019

		Total communicable		Enteric infections		Lower respiratory infections		Malaria		Neonatal sepsis and other neonatal infections	
		Female	Male	Female	Male	Female	Male	Female	Male	Female	Male
Low SDI	Afghanistan	-2.6%	-2.6%	-2.3%	-1.8%	-2.6%	-2.7%	0.1%	0.4%	-0.3%	-0.5%
	Benin	-2.0%	-2.0%	-2.3%	-2.4%	-2.3%	-2.3%	-0.8%	-0.7%	-1.4%	-1.4%
	Burkina Faso	-2.0%	-2.0%	-2.3%	-2.3%	-1.6%	-1.6%	-1.4%	-1.5%	-1.0%	-1.0%
	Burundi	-2.4%	-2.4%	-2.0%	-1.3%	-2.4%	-2.6%	-2.5%	-2.6%	-1.0%	-1.2%
	Central African Republic	-1.6%	-1.5%	-1.6%	-1.4%	-1.7%	-1.6%	-0.7%	-0.6%	-0.4%	-0.3%
	Chad	-1.7%	-1.6%	-1.1%	-1.1%	-1.6%	-1.6%	-1.7%	-1.8%	-1.4%	-1.2%
	Côte d'Ivoire	-2.1%	-2.1%	-2.3%	-2.4%	-2.1%	-2.1%	-1.5%	-1.7%	-1.4%	-1.3%
	Dem Rep of the Congo	-2.5%	-2.4%	-2.4%	-1.9%	-2.7%	-2.8%	-2.3%	-2.4%	-1.3%	-1.3%
	Eritrea	-2.6%	-2.6%	-2.5%	-2.5%	-2.6%	-2.7%	-2.7%	-2.7%	-1.3%	-1.3%
	Ethiopia	-2.7%	-2.7%	-2.7%	-2.6%	-2.9%	-2.9%	-0.4%	-0.2%	-1.3%	-1.5%
	Gambia	-2.6%	-2.6%	-2.4%	-2.4%	-2.6%	-2.7%	-3.0%	-2.9%	-1.7%	-1.7%
	Guinea	-2.3%	-2.2%	-2.4%	-2.3%	-2.5%	-2.4%	-1.4%	-1.2%	-1.6%	-1.5%
	Guinea-Bissau	-2.5%	-2.5%	-2.3%	-2.4%	-2.6%	-2.8%	-2.7%	-2.7%	-1.7%	-1.6%
	Haiti	-2.3%	-2.3%	-2.6%	-2.6%	-2.4%	-2.3%	-1.9%	-1.9%	-0.1%	-0.5%
	Liberia	-2.7%	-2.7%	-2.7%	-2.6%	-3.0%	-3.1%	-2.2%	-2.3%	-2.1%	-2.1%
	Madagascar	-2.3%	-2.3%	-2.3%	-2.1%	-2.5%	-2.6%	-2.0%	-2.2%	-1.2%	-1.4%
	Malawi	-2.8%	-2.7%	-3.0%	-2.9%	-2.7%	-2.6%	-2.8%	-2.9%	-1.7%	-1.8%
	Mali	-2.3%	-2.2%	-2.7%	-2.3%	-1.3%	-1.8%	-2.3%	-2.2%	-1.1%	-1.2%
	Mozambique	-2.4%	-2.4%	-2.9%	-3.0%	-2.6%	-2.7%	-2.6%	-2.4%	-1.2%	-1.1%
	Nepal	-3.0%	-2.9%	-3.0%	-3.0%	-3.0%	-3.0%	-3.3%	-3.3%	-1.4%	-1.6%
	Niger	-2.3%	-2.3%	-2.4%	-2.4%	-2.4%	-2.5%	-1.9%	-1.7%	-1.1%	-1.0%
	Pakistan	-2.3%	-2.3%	-2.1%	-2.2%	-1.9%	-2.1%	-2.6%	-2.6%	-1.1%	-1.4%
	Papua New Guinea	-1.4%	-1.5%	-1.6%	-1.5%	-1.2%	-1.4%	-1.8%	-1.8%	0.4%	0.3%
	Rwanda	-2.8%	-2.8%	-3.0%	-3.0%	-2.7%	-2.9%	-2.7%	-2.8%	-1.5%	-1.6%
	Senegal	-2.5%	-2.6%	-2.6%	-2.7%	-2.5%	-2.6%	-2.4%	-2.4%	-1.5%	-1.7%
	Sierra Leone	-2.3%	-2.3%	-2.7%	-2.7%	-2.4%	-2.4%	-1.8%	-1.6%	-1.5%	-1.5%
	Solomon Islands	-1.8%	-1.9%	-2.2%	-2.4%	-2.1%	-2.0%	-2.1%	-1.9%	-1.0%	-1.1%
	Somalia	-2.0%	-2.0%	-1.9%	-1.3%	-1.8%	-1.9%	-2.5%	-2.6%	-0.4%	-0.5%
	South Sudan	-1.8%	-1.8%	-1.4%	-1.2%	-2.0%	-2.0%	-1.2%	-1.0%	-0.8%	-0.8%
	Togo	-2.1%	-2.1%	-2.1%	-2.1%	-2.3%	-2.3%	-1.8%	-1.9%	-1.6%	-1.6%
	Uganda	-2.6%	-2.6%	-2.8%	-2.8%	-2.3%	-2.5%	-2.6%	-2.6%	-1.2%	-1.5%
	United Republic of Tanzania	-2.5%	-2.5%	-2.9%	-2.8%	-2.4%	-2.6%	-2.5%	-2.6%	-0.9%	-0.9%
	Yemen	-2.7%	-2.8%	-2.7%	-2.7%	-2.8%	-2.8%	-2.5%	-2.5%	-1.2%	-1.2%

		Vaccine Preventable disease		Meningitis & Encephalitis		HIV/AIDS		Tuberculosis		Neglected Tropical diseases	
		Female	Male	Female	Male	Female	Male	Female	Male	Female	Male
Low SDI	Afghanistan	-3.0%	-3.0%	-2.5%	-2.6%	8.3%	7.5%	-2.7%	-2.8%	-1.7%	-2.1%
	Benin	-2.6%	-2.6%	-2.3%	-2.1%	15.8%	19.0%	-2.5%	-2.4%	-2.2%	-2.2%
	Burkina Faso	-2.9%	-2.9%	-1.9%	-1.8%	-3.1%	-2.9%	-1.6%	-1.6%	-2.0%	-2.2%
	Burundi	-3.1%	-3.1%	-2.4%	-2.4%	-3.0%	-2.8%	-2.3%	-2.3%	-2.1%	-2.0%
	Central African Republic	-2.3%	-2.2%	-2.0%	-1.9%	-2.1%	-1.8%	-1.2%	-1.0%	-2.6%	-2.7%
	Chad	-2.6%	-2.6%	-1.3%	-1.1%	0.7%	1.5%	-1.7%	-1.5%	-1.4%	-1.6%
	Côte d'Ivoire	-3.0%	-3.0%	-2.3%	-2.3%	-2.2%	-1.7%	-2.0%	-2.0%	-2.4%	-2.3%
	Dem Rep of the Congo	-2.8%	-2.9%	-2.6%	-2.6%	-2.7%	-2.5%	-2.6%	-2.7%	-2.1%	-2.4%
	Eritrea	-3.2%	-3.2%	-2.4%	-2.3%	-1.3%	-0.6%	-2.5%	-2.2%	-2.6%	-2.8%
	Ethiopia	-3.1%	-3.1%	-2.8%	-2.8%	-1.6%	-1.2%	-2.9%	-2.9%	-2.8%	-2.9%
	Gambia	-3.1%	-3.1%	-2.5%	-2.5%	16.8%	17.6%	-2.3%	-2.0%	-1.7%	-1.9%
	Guinea	-2.9%	-2.8%	-2.0%	-1.7%	7.4%	8.0%	-2.5%	-2.3%	-1.5%	-1.6%
	Guinea-Bissau	-2.7%	-2.7%	-2.6%	-2.7%	7.3%	10.4%	-2.7%	-2.6%	-1.7%	-2.2%
	Haiti	-2.6%	-2.4%	-2.4%	-2.3%	-1.8%	-1.6%	-2.5%	-2.8%	-1.2%	-2.4%
	Liberia	-3.2%	-3.2%	-2.9%	-3.0%	3.2%	4.0%	-2.9%	-3.0%	-1.7%	-2.0%
	Madagascar	-2.7%	-2.7%	-2.6%	-2.6%	733.7%	633.6%	-2.5%	-2.5%	-1.7%	-2.4%
	Malawi	-3.1%	-3.1%	-2.8%	-2.7%	-2.3%	-2.0%	-2.7%	-2.5%	-2.6%	-2.7%
	Mali	-2.9%	-2.8%	-2.5%	-1.6%	2.2%	3.6%	-2.0%	-2.1%	-1.9%	-2.1%
	Mozambique	-3.2%	-3.1%	-2.7%	-2.6%	21.8%	24.1%	-2.6%	-2.3%	-2.2%	-2.4%
	Nepal	-3.2%	-3.2%	-2.9%	-2.8%	7,661.3%	8,453.9%	-3.1%	-2.9%	-2.4%	-2.8%
	Niger	-2.4%	-2.3%	-2.6%	-2.5%	-1.0%	-0.5%	-2.7%	-2.6%	-1.8%	-2.0%
	Pakistan	-3.1%	-3.1%	-2.1%	-2.1%	19.1%	20.9%	-2.3%	-1.7%	-2.1%	-2.0%
	Papua New Guinea	-2.1%	-2.2%	-1.9%	-2.0%	737.7%	760.6%	-1.8%	-1.8%	-1.4%	-1.5%
	Rwanda	-3.1%	-3.1%	-2.7%	-2.7%	-2.2%	-1.9%	-2.9%	-2.8%	-2.2%	-2.2%
	Senegal	-2.9%	-2.9%	-2.6%	-2.6%	0.7%	1.2%	-2.6%	-2.5%	-1.7%	-2.0%
	Sierra Leone	-2.8%	-2.9%	-2.4%	-2.4%	9.6%	8.4%	-2.4%	-2.4%	-2.1%	-2.2%
	Solomon Islands	-2.9%	-2.9%	-2.1%	-2.1%	4.8%	3.6%	-1.5%	-1.8%	-1.1%	-0.6%
	Somalia	-2.3%	-2.3%	-1.2%	-1.0%	51.5%	59.4%	-1.4%	-1.2%	-3.0%	-3.1%
	South Sudan	-2.7%	-2.7%	-1.7%	-1.6%	9.6%	10.0%	-1.9%	-1.8%	-2.6%	-2.6%
	Togo	-3.0%	-3.0%	-2.4%	-2.3%	1.9%	3.3%	-2.3%	-2.1%	-2.0%	-2.1%
	Uganda	-2.7%	-2.7%	-2.5%	-2.6%	-3.0%	-3.0%	-2.2%	-2.3%	-3.0%	-3.0%
	United Republic of Tanzania	-2.7%	-2.8%	-2.3%	-2.3%	-2.8%	-2.6%	-2.2%	-2.2%	-2.3%	-2.5%
	Yemen	-3.1%	-3.1%	-2.6%	-2.6%	3.7%	3.8%	-2.8%	-2.8%	-1.9%	-2.3%

		Infectious skin conditions		Sexually transmitted infections excluding HIV		Upper respiratory infections		Other unspecified infectious diseases		Hepatitis		Rheumatic heart disease		Maternal sepsis and other maternal infections
		Female	Male	Female	Male	Female	Male	Female	Male	Female	Male	Female	Male	Female
Low SDI	Afghanistan	0.0%	-0.0%	-0.5%	-0.4%	-0.2%	-0.2%	-1.6%	-1.8%	-2.6%	-2.8%	-2.1%	-1.9%	-2.2%
	Benin	-0.4%	-0.3%	-1.8%	-1.7%	-0.5%	-0.5%	-1.0%	-1.0%	-1.5%	-2.4%	-1.6%	-1.5%	-2.1%
	Burkina Faso	0.1%	-0.1%	-1.0%	-1.0%	-0.5%	-0.6%	-0.3%	-0.4%	-1.3%	-2.2%	-0.6%	-0.8%	-2.0%
	Burundi	-0.3%	-0.3%	-1.8%	-1.9%	-2.0%	-2.0%	-2.2%	-2.4%	-1.5%	-1.5%	-1.6%	-1.6%	-0.9%
	Central African Republic	-0.2%	-0.1%	-1.2%	-0.9%	-1.5%	-1.1%	-1.2%	-1.0%	-1.3%	-1.8%	-0.8%	-0.7%	0.3%
	Chad	-0.2%	-0.1%	-1.5%	-1.5%	-0.4%	-0.5%	-1.3%	-1.5%	-0.7%	-1.6%	-1.3%	-1.4%	-1.5%
	Côte d'Ivoire	-0.3%	-0.2%	-1.9%	-1.9%	-0.3%	-0.4%	-1.2%	-1.5%	-1.5%	-2.3%	-1.2%	-1.3%	-1.7%
	Dem Rep of the Congo	-0.5%	-0.3%	-2.1%	-2.1%	-1.8%	-2.0%	-1.9%	-2.2%	-1.9%	-2.4%	-1.3%	-1.0%	0.3%
	Eritrea	-0.3%	-0.2%	-1.9%	-1.9%	-2.1%	-2.0%	-2.4%	-2.5%	-1.6%	-1.2%	-1.4%	-1.4%	-1.8%
	Ethiopia	-0.6%	-0.4%	-2.7%	-2.7%	-2.6%	-2.5%	-2.1%	-2.2%	-2.2%	-1.8%	-1.9%	-2.0%	-2.6%
	Gambia	-0.3%	-0.2%	-1.7%	-1.8%	-0.4%	-0.4%	-1.0%	-1.4%	-1.3%	-0.9%	-1.3%	-1.3%	-1.4%
	Guinea	-0.5%	-0.3%	-1.9%	-1.8%	-0.6%	-0.5%	-1.5%	-1.7%	-1.5%	-1.8%	-1.7%	-1.7%	-1.5%
	Guinea-Bissau	-0.4%	-0.3%	-2.3%	-2.3%	-0.6%	-0.6%	-1.2%	-1.8%	-1.8%	-2.5%	-1.9%	-2.0%	-2.3%
	Haiti	-0.3%	-0.3%	-0.8%	-1.0%	-1.2%	-1.4%	-0.5%	-0.8%	-2.4%	-2.3%	-1.9%	-1.8%	-0.6%
	Liberia	-0.8%	-0.6%	-1.2%	-1.2%	-0.5%	-0.5%	-2.2%	-2.5%	-2.0%	-2.7%	-1.8%	-2.0%	-1.0%
	Madagascar	-0.3%	-0.2%	-1.2%	-1.2%	-1.9%	-2.1%	-1.3%	-1.7%	-1.9%	-2.2%	-1.8%	-1.7%	-1.6%
	Malawi	-0.4%	-0.2%	-1.3%	-1.2%	-2.4%	-2.4%	-1.6%	-1.9%	-2.3%	-2.4%	-1.6%	-1.3%	-1.6%
	Mali	-0.3%	-0.2%	-1.0%	-1.1%	-0.8%	-0.6%	-1.3%	-0.9%	-2.0%	-2.7%	-1.8%	-1.6%	-2.3%
	Mozambique	-0.2%	-0.1%	-2.5%	-2.5%	-2.5%	-2.3%	-0.8%	-1.3%	-2.2%	-2.4%	-1.3%	-0.8%	-2.5%
	Nepal	-0.1%	-0.1%	-1.8%	-1.7%	-1.6%	-1.3%	-0.8%	-1.5%	-2.8%	-2.5%	-2.1%	-1.2%	-3.1%
	Niger	-0.5%	-0.4%	-2.1%	-2.0%	-0.8%	-0.5%	-1.6%	-1.7%	-1.9%	-2.3%	-2.0%	-2.0%	-1.4%
	Pakistan	-0.0%	-0.0%	-1.6%	-1.5%	-1.1%	-0.9%	-1.0%	-1.1%	-2.1%	-1.8%	-0.7%	0.3%	-2.3%
	Papua New Guinea	0.0%	-0.0%	-0.6%	-0.6%	-0.1%	-0.2%	0.1%	0.3%	-1.8%	-2.1%	-0.3%	-0.2%	-1.9%
	Rwanda	-0.5%	-0.5%	-1.8%	-1.7%	-1.8%	-1.9%	-2.3%	-2.6%	-2.1%	-2.4%	-1.9%	-2.0%	-2.5%
	Senegal	-0.4%	-0.3%	-1.9%	-2.0%	-0.5%	-0.4%	-1.3%	-1.5%	-2.1%	-2.1%	-1.5%	-1.8%	-1.9%
	Sierra Leone	-0.4%	-0.3%	-1.8%	-1.7%	-0.6%	-0.6%	-1.2%	-1.5%	-1.4%	-2.5%	-1.6%	-1.8%	-0.3%
	Solomon Islands	-0.0%	-0.0%	-0.2%	-0.1%	-0.1%	-0.1%	-0.8%	-0.9%	-1.7%	-2.2%	-0.8%	-0.8%	-1.0%
	Somalia	-0.2%	-0.2%	-0.7%	-0.6%	-1.8%	-1.3%	-2.1%	-2.3%	-0.8%	-0.5%	-1.1%	-0.7%	0.6%
	South Sudan	-0.2%	-0.1%	-0.7%	-0.6%	-1.8%	-1.6%	-2.3%	-2.4%	-1.1%	-0.6%	-1.3%	-1.0%	-1.0%
	Togo	-0.3%	-0.2%	-2.2%	-2.2%	-0.2%	-0.2%	-1.1%	-1.5%	-1.7%	-2.4%	-1.5%	-1.4%	-1.9%
	Uganda	-0.2%	-0.1%	1.2%	1.3%	-1.9%	-2.1%	-2.2%	-2.4%	-1.2%	-1.1%	-0.5%	-0.7%	-1.3%
	United Republic of Tanzania	-0.3%	-0.2%	-2.2%	-2.2%	-1.7%	-1.8%	-1.0%	-1.3%	-1.7%	-1.9%	-1.1%	-1.2%	-2.0%
	Yemen	-0.1%	-0.1%	-1.1%	-1.0%	-0.4%	-0.5%	-0.1%	-0.7%	-2.9%	-3.1%	-2.1%	-1.7%	-1.3%

0 to 24 years, percentage change in DALYs/100,000, for males and females, 1990 to 2019

SDI	Country	Total communicable Female	Total communicable Male	Enteric infections Female	Enteric infections Male	Lower respiratory infections Female	Lower respiratory infections Male	Malaria Female	Malaria Male	Neonatal sepsis and other neonatal infections Female	Neonatal sepsis and other neonatal infections Male	Vaccine Preventable disease Female	Vaccine Preventable disease Male	Meningitis & Encephalitis Female	Meningitis & Encephalitis Male	HIV/AIDS Female	HIV/AIDS Male	Tuberculosis Female	Tuberculosis Male	Neglected Tropical diseases Female	Neglected Tropical diseases Male	Infectious skin conditions Female	Infectious skin conditions Male	Sexually transmitted infections excluding HIV Female	Sexually transmitted infections excluding HIV Male	Upper respiratory infections Female	Upper respiratory infections Male	Other unspecified infectious diseases Female	Other unspecified infectious diseases Male	Hepatitis Female	Hepatitis Male	Rheumatic heart disease Female	Rheumatic heart disease Male	Maternal sepsis and other maternal infections Female
Low-middle SDI	Angola	-2.7%	-2.7%	-2.9%	-3.0%	-2.9%	-2.9%	-1.7%	-1.2%	-1.7%	-1.1%	-2.8%	-2.7%	-2.7%	-2.6%	28.4%	25.8%	-2.9%	-2.9%	-2.9%	-3.0%	-0.4%	-0.2%	-1.7%	-1.1%	-2.2%	-2.1%	-1.9%	-1.9%	-2.3%	-2.7%	-1.8%	-1.5%	-2.2%
	Bangladesh	-2.9%	-2.9%	-2.9%	-2.9%	-2.8%	-2.9%	-3.3%	-3.3%	-1.6%	-1.4%	-3.2%	-3.2%	-1.5%	-1.5%			-3.0%	-3.0%	-2.5%	-2.6%	-0.2%	-0.4%	-2.2%	-2.2%	-1.4%	-1.1%	-1.9%	-1.7%	-2.8%	-2.8%	-1.2%	-1.3%	-3.0%
	Belize	-2.2%	-2.1%	-2.7%	-2.6%	-2.6%	-2.3%	-3.3%	-3.3%	-0.9%	-0.7%	-2.7%	-2.7%	-2.8%	-2.7%	-1.6%	-1.8%	-2.7%	-2.5%	-1.0%	-0.6%	-0.1%	-0.1%	-1.4%	0.5%	-0.3%	-0.7%	-0.9%	-0.7%	-2.7%	-1.9%	-0.9%	-0.6%	-1.2%
	Bhutan	-3.0%	-2.9%	-2.6%	-2.4%	-2.8%	-2.9%	-3.3%	-3.3%	-1.6%	-1.7%	-3.3%	-3.3%	-2.5%	-2.4%	10.0%	11.0%	-3.0%	-2.9%	-2.2%	-2.3%	-0.3%	-0.2%	-1.2%	-1.0%	-1.5%	-1.1%	-0.8%	-0.9%	-2.7%	-2.6%	-2.0%	-1.1%	-2.9%
	Bolivia (Plurinational State of)	-2.7%	-2.7%	-2.9%	-2.9%	-2.8%	-2.9%	-3.3%	-3.3%	-0.8%	-0.6%	-2.9%	-2.9%	-2.7%	-2.7%	4.7%	3.8%	-3.1%	-3.0%	-2.1%	-2.1%	-0.2%	-0.2%	-2.1%	-2.1%	-1.7%	-1.6%	-1.3%	-1.2%	-2.6%	-2.5%	-1.9%	-1.6%	-2.7%
	Cabo Verde	-2.8%	-2.8%	-3.1%	-3.1%	-2.8%	-2.6%	22.5%	23.7%	-0.6%	-1.3%	-3.3%	-3.3%	-2.8%	-2.8%	-1.1%	-0.3%	-3.0%	-2.7%	-1.8%	-1.6%	-0.5%	-0.4%	-2.1%	-2.2%	-0.3%	-0.3%	-1.0%	-2.5%	-2.4%	-1.4%	-2.1%	-1.5%	-3.0%
	Cambodia	-2.8%	-2.8%	-3.0%	-3.0%	-2.9%	-2.8%	-3.1%	-3.1%	-1.2%	-1.4%	-3.2%	-3.2%	-2.9%	-2.8%	105.4%	79.1%	-2.9%	-2.8%	-2.5%	-2.6%	-0.2%	-0.2%	-2.2%	-2.1%	-0.8%	-1.2%	-1.7%	-1.4%	-1.9%	-1.1%	-2.1%	-2.1%	-2.9%
	Cameroon	-2.0%	-2.1%	-2.3%	-2.2%	-1.9%	-1.9%	-0.9%	-1.0%	-1.4%	-1.5%	-2.9%	-2.9%	-2.1%	-2.0%	4.3%	5.7%	-1.9%	-1.8%	-2.3%	-2.3%	-0.3%	-0.2%	-2.3%	-2.3%	-0.2%	-0.2%	-1.8%	-2.1%	-1.3%	-2.2%	-1.2%	-0.9%	-1.9%
	Comoros	-2.5%	-2.6%	-2.6%	-2.6%	-2.5%	-2.7%	-2.7%	-2.9%	-1.5%	-1.7%	-2.6%	-2.7%	-2.0%	-2.2%	61.2%	65.7%	-2.3%	-2.4%	-1.7%	-1.9%	-0.4%	-0.3%	-2.3%	-2.3%	-1.9%	-1.9%	-2.1%	-2.4%	-1.1%	-0.9%	-1.2%	-1.0%	-1.9%
	Congo	-2.4%	-2.4%	-2.7%	-2.6%	-2.7%	-2.6%	-2.1%	-2.0%	-1.1%	-0.8%	-2.4%	-2.4%	-2.5%	-2.4%	-2.3%	-2.0%	-2.6%	-2.7%	-3.0%	-3.0%	-0.4%	-0.3%	-1.7%	-1.5%	-1.1%	-1.1%	-1.5%	-1.6%	-1.9%	-2.1%	-1.1%	-1.3%	-2.1%
	Democratic People's Republic of Kor..	-2.9%	-2.9%	-2.2%	-2.2%	-3.1%	-3.1%	196.0%	199.4%	-1.3%	-1.5%	-3.3%	-3.3%	-2.7%	-2.7%	27.1%	27.5%	-2.5%	-2.2%	-1.5%	-2.2%	-0.2%	-0.2%	-1.6%	-1.7%	-1.5%	-1.3%	-1.9%	-2.4%	-2.6%	-2.7%	-1.4%	-0.7%	-1.1%
	Djibouti	-2.2%	-2.3%	-2.7%	-2.8%	-2.1%	-2.1%	-1.6%	-1.6%	-1.2%	-1.2%	-2.4%	-2.4%	-2.0%	-1.7%	198.6%	211.7%	-2.3%	-2.1%	-2.9%	-2.9%	-0.2%	-0.0%	-1.0%	-0.9%	-1.6%	-1.3%	-2.0%	-2.0%	-1.5%	-0.9%	-1.2%	-1.0%	-1.4%
	Dominican Republic	-2.3%	-2.2%	-3.0%	-3.0%	-2.8%	-2.7%	-2.1%	-2.0%	0.2%	-0.3%	-2.2%	-2.1%	-2.7%	-2.6%	-1.7%	-1.7%	-2.8%	-2.7%	-1.1%	-1.8%	-0.1%	-0.2%	-0.3%	-0.3%	-0.0%	-0.4%	-1.6%	-1.7%	-2.1%	-1.9%	-1.5%	-1.4%	-1.5%
	El Salvador	-2.7%	-2.7%	-3.1%	-3.1%	-2.8%	-2.7%	-3.2%	-3.1%	-2.3%	-2.5%	-2.9%	-2.9%	-3.0%	-3.0%	15.2%	13.1%	-3.1%	-3.1%	-1.7%	-1.5%	-0.2%	-0.2%	-1.8%	-2.0%	-0.5%	-0.3%	-1.8%	-1.8%	-1.2%	-1.4%	-0.9%	-0.6%	-3.1%
	Eswatini	-0.9%	-1.4%	-1.5%	-1.4%	-1.5%	-1.7%	-2.8%	-2.9%	-0.7%	-1.2%	-3.0%	-3.0%	-1.6%	-1.7%	74.9%	102.0%	-1.5%	-1.5%	-2.1%	-1.9%	-0.1%	-0.1%	-2.4%	-2.5%	-0.6%	-0.7%	-1.1%	-1.3%	-1.4%	-1.5%	-1.0%	-0.6%	-1.9%
	Ghana	-2.3%	-2.3%	-2.9%	-3.0%	-2.3%	-2.0%	-1.5%	-1.0%	-0.7%	-0.2%	-3.0%	-3.0%	-2.3%	-2.0%	-0.4%	-0.1%	-2.6%	-2.1%	-2.0%	-2.0%	-0.3%	-0.2%	-1.0%	-0.7%	-0.1%	-0.1%	-0.9%	-0.8%	-2.0%	-1.8%	-1.1%	-0.7%	-2.5%
	Guatemala	-2.7%	-2.7%	-3.0%	-3.0%	-2.6%	-2.5%	-3.2%	-3.2%	-2.0%	-2.1%	-3.1%	-3.1%	-2.4%	-2.4%	1.4%	0.8%	-3.2%	-3.1%	-2.8%	-2.8%	-0.2%	-0.3%	-0.9%	-2.3%	-1.8%	-1.5%	-1.1%	-0.9%	-1.6%	-0.8%	-0.7%	-0.5%	-2.7%
	Honduras	-2.7%	-2.6%	-3.0%	-2.9%	-3.0%	-2.8%	-3.2%	-3.2%	-1.6%	-1.6%	-2.8%	-2.7%	-2.6%	-2.3%	-1.9%	-2.0%	-2.9%	-2.8%	-0.4%	-0.8%	-0.3%	-0.3%	-2.0%	-1.9%	-1.5%	-1.4%	-1.8%	-1.9%	-2.7%	-2.4%	0.3%	0.4%	-3.1%
	India	-2.7%	-2.7%	-2.7%	-2.7%	-2.6%	-2.7%	-3.0%	-3.0%	-1.7%	-1.8%	-3.1%	-3.1%	-2.7%	-2.6%	13.1%	12.2%	-2.8%	-2.7%	-2.3%	-2.4%	-0.7%	-0.5%	-2.2%	-2.2%	-1.2%	-0.9%	-1.5%	-1.6%	-2.4%	-2.1%	-1.8%	-1.5%	-3.0%
	Kenya	-2.3%	-2.2%	-2.1%	-2.0%	-2.5%	-2.6%	-2.4%	-2.4%	-1.2%	-1.4%	-2.9%	-2.9%	-2.4%	-2.4%	-1.9%	-1.6%	-2.1%	-1.5%	-1.7%	-2.1%	-0.2%	-0.1%	-2.2%	-2.4%	-1.0%	-1.1%	-1.1%	-1.2%	-1.4%	-0.7%	-0.3%	-0.0%	-1.9%
	Kiribati	-2.1%	-2.1%	-2.6%	-2.4%	-2.4%	-2.3%			-0.7%	-0.7%	-2.1%	-2.1%	-2.3%	-2.3%	0.9%	1.1%	-1.8%	-1.8%	-1.0%	-2.1%	-0.1%	-0.1%	-0.6%	-0.6%	-0.3%	-0.0%	-0.6%	-1.0%	-1.8%	-2.4%	-1.3%	-1.1%	-1.2%
	Kyrgyzstan	-2.7%	-2.8%	-2.7%	-2.8%	-2.9%	-2.9%	-3.3%	-3.3%	0.0%	-0.1%	-1.8%	-1.8%	-2.7%	-2.7%	4.8%	6.2%	-2.2%	-2.4%	-0.5%	-0.7%	-0.1%	-0.1%	-2.8%	-2.8%	-2.0%	-1.2%	0.4%	0.1%	-2.9%	-2.7%	-2.1%	-2.0%	-0.8%
	Lao People's Democratic Republic	-2.8%	-2.8%	-2.9%	-3.0%	-2.9%	-2.9%	-2.9%	-2.9%	-1.1%	-1.3%	-3.0%	-3.1%	-2.8%	-2.7%	4,834.4%	5,756.5%	-2.9%	-2.9%	-1.8%	-2.1%	-0.2%	-0.2%	-1.5%	-1.4%	-1.0%	-1.8%	-1.7%	-1.7%	-2.9%	-3.0%	-1.3%	-1.3%	-3.1%
	Lesotho	-0.6%	-0.9%	-2.1%	-2.0%	-1.2%	-1.0%			-0.9%	-0.9%	-3.0%	-3.0%	-1.5%	-1.1%	18.0%	20.3%	-0.7%	-0.3%	-2.6%	-2.5%	-0.1%	-0.1%	-1.8%	-1.8%	-0.6%	-0.5%	-1.2%	-1.2%	-1.1%	-1.6%	-0.5%	-0.3%	-1.6%
	Maldives	-2.8%	-2.8%	-2.8%	-2.9%	-2.9%	-2.9%			-1.2%	-1.7%	-3.2%	-3.2%	-2.7%	-2.7%	12.9%	7.7%	-3.2%	-3.1%	-2.3%	-2.1%	-0.1%	-0.2%	-1.8%	-1.9%	-0.3%	-0.5%	-2.0%	-2.3%	-3.0%	-1.8%	-2.8%	-2.2%	-2.9%
	Marshall Islands	-1.5%	-1.6%	-2.3%	-2.4%	-1.5%	-1.7%			-0.8%	-0.9%	-1.8%	-1.9%	-1.7%	-1.9%	6.6%	4.7%	-1.4%	-1.6%	-1.0%	-1.1%	-0.0%	-0.1%	-0.5%	-0.4%	-0.1%	-0.2%	-0.8%	-0.7%	-1.8%	-2.0%	0.5%	-0.7%	-1.3%
	Mauritania	-2.5%	-2.4%	-2.7%	-2.5%	-2.6%	-2.6%	7.4%	7.3%	-1.8%	-1.8%	-3.1%	-3.1%	-2.6%	-2.5%	-2.8%	-2.6%	-2.7%	-2.8%	-1.7%	-2.0%	-0.5%	-0.4%	-2.0%	-2.0%	-0.2%	-0.3%	-1.8%	-2.0%	-1.8%	-2.3%	-1.6%	-1.5%	-2.8%
	Micronesia (Federated States of)	-2.2%	-2.2%	-2.6%	-2.6%	-2.5%	-2.5%			-1.4%	-1.5%	-2.7%	-2.7%	-2.4%	-2.4%	-0.7%	-0.9%	-2.2%	-2.2%	-2.0%	-2.5%	-0.1%	-0.1%	-1.6%	-1.6%	-0.1%	-0.2%	-1.1%	-0.8%	-1.9%	-1.5%	-0.7%	-0.6%	-1.9%
	Mongolia	-2.9%	-2.9%	-3.0%	-3.0%	-3.0%	-3.0%			-0.5%	-0.8%	-2.7%	-2.8%	-3.0%	-2.9%	484.9%	578.4%	-2.9%	-2.8%	-2.3%	-1.8%	-0.1%	-0.4%	1.8%	1.6%	-1.2%	-1.3%	-1.6%	-2.7%	-3.0%	-2.9%	-1.3%	-1.3%	-2.7%
	Morocco	-2.9%	-2.9%	-3.0%	-2.9%	-3.0%	-3.0%	-3.3%	-3.3%	-1.3%	-1.4%	-3.1%	-3.1%	-2.8%	-2.9%	6.0%	5.1%	-2.8%	-2.9%	-1.6%	-1.6%	-0.1%	-0.1%	-1.7%	-1.7%	-0.1%	-0.1%	-1.4%	-1.6%	-2.6%	-2.8%	-2.2%	-2.0%	-3.1%
	Myanmar	-2.8%	-2.8%	-3.0%	-3.1%	-2.9%	-2.8%	-3.2%	-3.2%	-1.5%	-1.4%	-3.0%	-3.0%	-2.9%	-2.5%	73.4%	47.9%	-2.9%	-2.8%	-2.5%	-2.4%	-0.1%	-0.1%	-0.4%	0.1%	-0.8%	-1.0%	-1.1%	-1.6%	-2.1%	-2.1%	-1.9%	-2.0%	-2.6%
	Nicaragua	-2.8%	-2.8%	-3.1%	-3.2%	-2.8%	-2.7%	-3.1%	-3.1%	-2.1%	-2.1%	-3.3%	-3.3%	-2.9%	-2.9%	21.2%	22.3%	-3.0%	-3.1%	-1.6%	-1.8%	-0.2%	-0.3%	-2.3%	-2.4%	-0.2%	-0.2%	-2.3%	-2.0%	-2.4%	-2.0%	-1.3%	-0.9%	-2.8%
	Nigeria	-2.1%	-2.2%	-2.2%	-2.5%	-2.0%	-2.1%	-2.0%	-1.9%	-1.1%	-1.1%	-2.9%	-2.9%	-1.9%	-1.8%	3.7%	5.0%	-2.5%	-2.4%	-1.8%	-2.2%	-0.1%	0.1%	-0.5%	-0.5%	-0.6%	-0.4%	-0.8%	-0.9%	-2.1%	-2.4%	-1.8%	-1.7%	-1.9%
	Palestine	-2.7%	-2.6%	-1.8%	-1.7%	-2.9%	-2.7%			-1.9%	-1.5%	-3.3%	-3.3%	-3.0%	-2.9%	22.5%	19.2%	-2.9%	-2.8%	-2.1%	-2.5%	-0.1%	-0.1%	-1.4%	-1.4%	-0.2%	-0.2%	-1.6%	-1.5%	-2.8%	-2.0%	-1.4%	-1.1%	-2.3%
	Sao Tome and Principe	-2.8%	-2.8%	-3.1%	-3.1%	-2.8%	-2.9%	-2.2%	-2.5%	-1.8%	-1.5%	-3.2%	-3.2%	-2.5%	-2.6%	-1.1%	-1.3%	-2.8%	-2.6%	-2.1%	-2.2%	-0.5%	-0.4%	-1.8%	-1.8%	-0.3%	-0.4%	-1.6%	-2.0%	-2.1%	-2.6%	-1.2%	-1.6%	-2.3%
	Sudan	-2.9%	-2.9%	-2.7%	-2.8%	-3.0%	-2.9%	-2.3%	-2.3%	-1.5%	-1.7%	-3.2%	-3.2%	-3.0%	-2.9%	9.2%	6.6%	-3.0%	-3.0%	-2.6%	-2.6%	-0.0%	-0.1%	-1.2%	-1.1%	-0.3%	-0.3%	-1.4%	-1.6%	-2.7%	-3.1%	-2.7%	-2.2%	-2.7%
	Tajikistan	-2.7%	-2.7%	-2.8%	-2.9%	-2.7%	-2.6%	-3.3%	-3.3%	0.0%	-0.5%	-2.1%	-2.0%	-2.8%	-2.9%	5.2%	1.9%	-2.1%	-1.7%	-1.2%	-1.0%	-0.1%	-0.1%	-1.5%	-1.4%	-1.7%	-1.4%	-0.7%	-0.7%	-2.8%	-2.7%	-1.9%	-1.4%	-1.2%
	Timor-Leste	-2.9%	-2.8%	-2.8%	-2.8%	-2.9%	-3.0%	-3.3%	-3.3%	-1.7%	-1.5%	-3.1%	-3.1%	-2.7%	-2.7%	6.6%	7.1%	-2.9%	-2.9%	-2.0%	-2.1%	-0.1%	-0.1%	-1.8%	-1.5%	-0.9%	-1.1%	-1.3%	-1.2%	-2.9%	-2.6%	-0.9%	-0.2%	-2.5%
	Tuvalu	-2.7%	-2.7%	-2.5%	-2.4%	-3.0%	-3.0%			-1.7%	-1.8%	-2.8%	-2.8%	-2.9%	-2.9%	31.9%	29.1%	-2.8%	-2.9%	-1.1%	-0.6%	-0.1%	-0.2%	-1.8%	-1.9%	-0.2%	-0.3%	-1.6%	-1.5%	-2.4%	-2.1%	-2.0%	-1.4%	-2.7%
	Vanuatu	-1.6%	-1.7%	-2.0%	-2.0%	-1.5%	-1.4%	-3.3%	-3.3%	-1.1%	-1.1%	-2.2%	-2.2%	-1.3%	-1.4%	4.9%	3.4%	-1.3%	-1.3%	0.7%	-2.1%	0.0%	0.0%	-0.0%	0.1%	-0.1%	-0.1%	0.1%	0.3%	-1.5%	-2.2%	0.7%	0.4%	-0.8%
	Venezuela (Bolivarian Republic of)	-1.8%	-1.9%	-2.7%	-2.7%	-2.0%	-1.9%	6.6%	5.2%	-0.6%	-0.9%	-2.2%	-2.0%	-2.4%	-2.6%	-1.5%	-1.5%	-2.2%	-2.6%	-1.4%	-1.3%	-0.1%	-0.1%	-0.4%	-1.1%	-0.2%	-0.2%	-0.7%	-0.4%	-2.3%	-2.4%	-2.4%	-2.5%	-1.6%
	Zambia	-2.5%	-2.5%	-2.6%	-2.5%	-2.8%	-2.8%	-2.5%	-2.5%	-1.2%	-1.3%	-3.1%	-3.1%	-2.9%	-2.9%	-1.8%	-1.2%	-2.7%	-2.6%	-2.8%	-3.0%	-0.3%	-0.2%	-1.4%	-1.5%	-2.0%	-2.0%	-1.6%	-1.8%	-2.0%	-1.8%	-1.7%	-1.3%	-2.3%
	Zimbabwe	-1.2%	-1.1%	-0.9%	-1.0%	-0.4%	-0.5%	-1.7%	-1.9%	-0.4%	-0.3%	-2.5%	-2.6%	-0.8%	-0.9%	-1.4%	-1.1%	0.5%	0.6%	-2.4%	-2.2%	-0.0%	-0.0%	-0.6%	-0.6%	-0.2%	-0.1%	0.6%	0.6%	-0.3%	-1.8%	0.2%	0.6%	1.2%

0 to 24 years, percentage change in DALYs/100,000, for males and females, 1990 to 2019

		Total communicable		Enteric infections		Lower respiratory infections		Malaria		Neonatal sepsis and other neonatal infections		Vaccine Preventable disease		Meningitis & Encephalitis		HIV/AIDS		Tuberculosis		Neglected Tropical diseases		Infectious skin conditions		Sexually transmitted infections excluding HIV		Upper respiratory infections		Other unspecified infectious diseases		Hepatitis		Rheumatic heart disease		Maternal sepsis and other maternal infections
		Female	Male	Female	Male	Female	Male	Female	Male	Female	Male	Female	Male	Female	Male	Female	Male	Female	Male	Female	Male	Female	Male	Female	Male	Female	Male	Female	Male	Female	Male	Female	Male	Female
Middle SDI	Albania	-2.8%	-2.8%	-1.9%	-1.9%	-3.0%	-3.0%			-1.5%	-1.1%	-3.2%	-3.2%	-2.7%	-2.7%	1.7%	3.9%	-3.0%	-3.0%	-2.1%	-2.4%	-0.5%	-0.5%	-1.7%	-1.6%	-0.6%	-0.8%	-1.9%	-1.9%	-2.2%	-1.6%	-1.3%	-1.4%	-0.6%
	Algeria	-2.6%	-2.7%	-2.4%	-2.8%	-2.8%	-2.8%	-2.9%	-2.9%	-0.8%	-0.7%	-3.0%	-3.1%	-2.7%	-2.7%	2.1%	2.1%	-2.9%	-2.9%	-1.8%	-2.1%	-0.1%	-0.1%	-1.8%	-1.8%	-0.1%	-0.1%	-1.2%	-1.4%	-2.5%	-2.6%	-2.4%	-2.1%	-3.1%
	Armenia	-2.6%	-2.5%	-2.8%	-2.9%	-2.8%	-2.6%	-3.3%	-3.3%	-1.5%	-2.0%	-1.4%	-1.4%	-1.8%	-1.2%	19.9%	16.6%	-2.4%	-2.2%	-0.5%	-0.4%	-0.0%	-0.0%	-0.4%	-0.8%	-1.5%	-0.3%	0.0%	0.3%	-2.2%	-0.9%	-1.2%	-1.0%	-2.0%
	Azerbaijan	-2.6%	-2.7%	-2.9%	-3.0%	-2.7%	-2.8%	-3.3%	-3.3%	0.0%	-0.4%	-2.5%	-2.5%	-1.7%	-1.8%	1.3%	0.1%	-2.6%	-2.4%	-1.8%	-1.7%	-0.1%	-0.0%	-1.1%	-1.3%	-1.3%	-1.3%	-0.2%	-0.3%	-2.5%	-2.3%	-1.2%	-0.9%	-1.7%
	Botswana	-1.6%	-1.7%	-2.1%	-2.3%	-0.2%	-0.6%	0.4%	0.3%	-0.8%	-1.0%	-2.8%	-2.9%	-0.7%	-0.9%	-1.6%	-1.5%	-1.1%	-1.4%	-2.3%	-2.3%	-0.1%	-0.1%	-1.9%	-2.0%	-0.3%	-0.4%	-1.0%	-1.0%	-1.4%	-1.8%	-0.9%	-1.1%	-2.5%
	Brazil	-2.6%	-2.6%	-3.0%	-3.0%	-2.8%	-2.8%	-3.2%	-3.3%	-1.5%	-1.6%	-2.5%	-2.7%	-2.8%	-2.8%	-1.1%	-1.7%	-2.8%	-2.8%	-0.9%	-1.0%	-0.0%	-0.1%	-1.5%	-1.8%	-0.5%	-0.6%	-0.8%	-0.8%	-1.9%	-2.0%	-1.4%	-1.6%	-1.8%
	China	-2.9%	-2.9%	-3.0%	-3.0%	-3.1%	-3.1%	-3.3%	-3.3%	4.4%	2.1%	-3.2%	-3.2%	-2.8%	-2.8%	6.6%	9.0%	-3.1%	-3.1%	-2.6%	-2.5%	-0.2%	-0.2%	-1.1%	-1.2%	-1.6%	-1.5%	-2.4%	-2.7%	-3.0%	-2.9%	-2.6%	-2.4%	-3.1%
	Colombia	-2.1%	-2.2%	-2.9%	-2.9%	-2.4%	-2.6%	-3.2%	-3.2%	0.6%	0.8%	-2.6%	-2.7%	-2.5%	-2.6%	10.7%	4.8%	-2.6%	-2.8%	0.3%	-0.2%	-0.1%	-0.1%	-2.1%	-2.3%	-0.5%	-0.6%	-1.2%	-1.0%	-1.5%	-1.5%	-2.8%	-2.9%	-2.1%
	Costa Rica	-1.9%	-2.0%	-2.2%	-2.3%	-2.6%	-2.6%	-3.3%	-3.3%	-2.1%	-2.5%	-2.2%	-2.0%	-2.3%	-2.4%	-0.6%	-0.2%	-2.7%	-2.7%	-1.3%	-1.3%	-0.1%	-0.1%	-1.4%	-1.1%	-0.2%	-0.2%	-0.7%	-0.1%	-2.0%	-1.7%	-0.6%	-0.5%	-2.2%
	Cuba	-1.7%	-1.7%	-1.6%	-1.7%	-2.1%	-2.2%			-0.7%	-1.4%	-1.3%	-1.4%	-2.9%	-2.9%	1.7%	8.0%	-2.4%	-2.4%	-1.0%	-0.9%	0.0%	-0.1%	1.4%	-0.4%	-0.1%	-0.2%	-0.5%	-0.3%	-2.4%	-2.3%	-1.5%	-1.4%	-2.1%
	Ecuador	-2.6%	-2.5%	-3.0%	-3.1%	-2.6%	-2.5%	-3.3%	-3.3%	-1.8%	-2.1%	-2.4%	-2.5%	-2.4%	-2.4%	10.5%	15.1%	-3.0%	-2.8%	-2.4%	-2.3%	-0.0%	-0.0%	-1.6%	-1.7%	-0.8%	-0.8%	-1.7%	-1.6%	-2.5%	-2.2%	-1.2%	-1.1%	-2.4%
	Egypt	-2.9%	-2.8%	-2.8%	-2.8%	-3.0%	-3.0%	-3.3%	-3.3%	-1.6%	-1.7%	-3.2%	-3.2%	-2.5%	-2.4%	-1.8%	-1.7%	-2.8%	-2.8%	-2.9%	-2.9%	-0.3%	-0.3%	-1.8%	-1.8%	-0.6%	-0.5%	-1.9%	-1.9%	-3.0%	-2.7%	-2.9%	-2.5%	-3.0%
	Equatorial Guinea	-2.8%	-2.9%	-3.2%	-3.3%	-3.2%	-3.2%	-2.3%	-2.3%	-2.1%	-2.1%	-3.2%	-3.2%	-3.0%	-2.9%	42.5%	38.8%	-3.2%	-3.2%	-2.8%	-3.0%	-0.8%	-0.7%	-1.6%	-1.5%	-2.3%	-2.4%	-2.4%	-2.5%	-2.7%	-2.8%	-1.8%	-1.8%	-3.2%
	Fiji	-1.1%	-1.1%	-0.9%	-0.9%	-0.8%	-0.8%			-1.1%	-1.1%	-2.2%	-2.4%	-1.7%	-1.5%	7.3%	6.5%	-1.8%	-1.4%	-0.6%	-1.7%	-0.0%	-0.0%	-0.2%	-0.2%	-0.1%	-0.1%	-0.2%	0.5%	-1.5%	-1.5%	-1.0%	-0.8%	-1.7%
	Gabon	-2.4%	-2.4%	-2.9%	-2.7%	-2.8%	-2.7%	-2.3%	-2.3%	-1.2%	-1.3%	-2.9%	-2.8%	-2.7%	-2.5%	1.7%	1.9%	-2.7%	-2.7%	-2.5%	-2.6%	-0.5%	-0.3%	-2.2%	-2.1%	-1.0%	-1.2%	-1.7%	-1.8%	-1.9%	-1.8%	-0.7%	-0.9%	-2.6%
	Grenada	-1.4%	-1.6%	-1.3%	-1.9%	-1.8%	-1.9%			0.2%	-0.2%	-2.4%	-2.6%	-2.8%	-2.9%	-1.6%	-2.0%	-2.2%	-2.3%	-1.7%	-1.7%	-0.1%	-0.2%	0.1%	-1.8%	-0.3%	-0.6%	-1.0%	-0.7%	-2.5%	-2.2%	-1.5%	-0.8%	-2.0%
	Guyana	-2.0%	-2.1%	-2.6%	-2.8%	-1.8%	-1.9%	-2.9%	-3.0%	-0.7%	-0.7%	-2.6%	-2.8%	-2.2%	-2.3%	1.0%	-1.1%	-2.2%	-2.2%	-1.3%	-1.0%	0.1%	0.0%	-1.9%	-2.6%	-0.2%	-0.3%	-0.9%	-0.5%	-2.4%	-2.1%	-1.3%	-1.4%	-1.8%
	Indonesia	-2.6%	-2.6%	-2.7%	-2.7%	-2.9%	-2.9%	-2.8%	-2.9%	-1.4%	-1.6%	-3.0%	-3.0%	-2.6%	-2.7%	420.0%	487.6%	-2.8%	-2.6%	-2.4%	-1.8%	-0.1%	-0.1%	-0.3%	-0.0%	-0.2%	-0.3%	-1.5%	-1.9%	-2.9%	-1.8%	-2.2%	-2.0%	-2.8%
	Iran (Islamic Republic of)	-2.8%	-2.7%	-2.4%	-2.4%	-3.1%	-3.0%	-3.3%	-3.3%	-1.5%	-1.1%	-3.2%	-3.2%	-2.8%	-2.7%	88.7%	72.4%	-3.0%	-3.0%	-2.3%	-2.4%	-0.1%	-0.1%	-1.2%	-1.2%	-0.2%	-0.2%	-1.2%	-1.4%	-2.8%	-2.4%	-2.4%	-2.0%	-2.8%
	Iraq	-2.6%	-2.7%	-2.7%	-2.7%	-2.9%	-3.1%	-3.3%	-3.3%	-1.9%	-1.0%	-2.9%	-3.0%	-2.5%	-2.7%	17.7%	15.3%	-2.9%	-3.0%	-1.9%	-2.3%	-0.2%	-0.2%	-1.4%	-1.4%	-0.4%	-0.4%	-1.6%	-2.1%	-2.7%	-2.7%	-2.2%	-2.0%	-1.8%
	Jamaica	-1.5%	-1.8%	-2.8%	-2.9%	-2.5%	-2.5%			2.0%	0.4%	-2.3%	-2.7%	-2.5%	-2.7%	1.5%	0.3%	-1.8%	-2.4%	-0.3%	-0.2%	-0.1%	-0.2%	-1.1%	-0.1%	-0.1%	-0.1%	0.2%	1.3%	-0.8%	-1.2%	-1.9%	-1.8%	-2.3%
	Mexico	-2.6%	-2.6%	-3.1%	-3.1%	-2.8%	-2.8%	-3.2%	-3.2%	-0.7%	-0.9%	-2.9%	-2.9%	-2.4%	-2.5%	1.5%	0.5%	-2.9%	-2.8%	-1.9%	-1.9%	0.1%	0.1%	-1.4%	-2.4%	-1.5%	-1.6%	-1.6%	-1.4%	-1.8%	-1.5%	-2.0%	-1.9%	-1.9%
	Namibia	-1.6%	-1.7%	-2.0%	-1.8%	-1.9%	-2.0%	-2.4%	-2.5%	-0.8%	-1.0%	-3.1%	-3.2%	-1.9%	-1.8%	8.0%	11.6%	-2.0%	-2.0%	-1.3%	-1.2%	-0.1%	-0.1%	-1.4%	-1.4%	-0.5%	-0.6%	-1.3%	-1.4%	-1.6%	-1.4%	-1.3%	-1.1%	-2.5%
	Nauru	-1.6%	-1.5%	-1.4%	-1.5%	-1.6%	-1.6%			-0.9%	-0.9%	-3.1%	-3.1%	-1.8%	-1.7%	37.4%	32.6%	-1.7%	-1.9%	1.2%	2.0%	-0.0%	-0.0%	-0.2%	-0.2%	-0.0%	-0.1%	-0.8%	-0.5%	-1.4%	-1.3%	-0.2%	-0.2%	-1.2%
	Panama	-1.3%	-1.1%	-1.7%	-1.9%	-0.9%	-0.5%	-2.8%	-3.0%	-0.5%	-0.8%	-2.8%	-2.9%	-2.3%	-2.5%	1.8%	8.5%	-2.2%	-2.3%	-2.1%	-2.3%	-0.1%	-0.0%	0.8%	1.0%	0.1%	0.1%	0.8%	1.1%	-2.3%	-2.1%	-1.7%	-1.4%	-1.7%
	Paraguay	-2.2%	-2.2%	-2.8%	-2.8%	-2.6%	-2.6%	-3.3%	-3.3%	-2.8%	-2.8%	-2.9%	-2.9%	-2.6%	-2.6%	22.1%	20.6%	-2.6%	-2.2%	-1.7%	-1.6%	-0.1%	-0.1%	-0.6%	-0.6%	-0.2%	-0.3%	-0.3%	-0.3%	-1.6%	-1.0%	-0.6%	-0.4%	-2.8%
	Peru	-2.6%	-2.6%	-2.9%	-3.0%	-3.0%	-3.0%	-3.2%	-3.2%	-0.9%	-1.1%	-2.5%	-2.5%	-2.5%	-2.4%	14.2%	10.0%	-3.1%	-3.1%	-2.1%	-2.0%	-0.3%	-0.3%	-2.3%	-2.4%	-0.6%	-0.8%	-1.6%	-1.2%	-2.6%	-2.5%	-1.0%	-0.9%	-2.9%
	Philippines	-2.2%	-2.2%	-2.6%	-2.7%	-2.4%	-2.4%	-3.2%	-3.2%	-0.8%	-1.2%	-2.6%	-2.6%	-2.2%	-2.1%	13.9%	11.2%	-2.3%	-2.2%	-1.1%	-1.6%	-0.1%	-0.2%	-0.0%	-0.2%	-0.5%	-0.4%	-0.7%	0.5%	-2.7%	-2.6%	2.6%	2.1%	-2.0%
	Saint Lucia	-1.4%	-1.8%	-1.9%	-2.2%	-2.0%	-2.1%			-0.4%	-0.7%	-2.2%	-1.9%	-2.4%	-2.6%	-1.4%	-1.7%	-2.5%	-2.4%	-1.6%	-1.7%	-0.1%	-0.1%	-2.0%	-2.2%	-0.2%	-0.7%	-0.8%	-0.6%	-1.6%	-1.6%	-1.0%	-0.7%	-2.1%
	Saint Vincent and the Grenadines	-1.3%	-1.6%	-2.3%	-2.4%	-1.8%	-2.0%			1.1%	0.4%	-1.2%	-1.8%	-2.5%	-2.6%	-0.4%	-1.3%	-2.5%	-2.2%	-1.4%	-1.3%	0.2%	0.1%	-0.5%	-2.8%	-0.1%	-0.6%	-1.0%	-0.7%	-2.2%	-1.4%	-1.3%	-1.0%	-1.1%
	Samoa	-1.6%	-1.5%	-1.5%	-1.5%	-2.4%	-2.4%			-0.9%	-1.0%	-1.7%	-1.4%	-2.2%	-2.2%	8.7%	7.3%	-1.9%	-2.1%	0.1%	-0.9%	-0.1%	-0.1%	-0.4%	-0.2%	-0.1%	-0.1%	-0.8%	-0.7%	-1.1%	-1.7%	-0.8%	-1.0%	-2.6%
	South Africa	-1.3%	-1.7%	-2.4%	-2.5%	-2.4%	-2.4%	-1.6%	-1.8%	0.6%	-0.1%	-2.5%	-2.6%	-2.3%	-2.3%	28.2%	25.0%	-2.1%	-2.2%	-2.2%	-2.2%	-0.2%	-0.2%	-1.4%	-1.5%	-0.6%	-0.6%	-0.4%	-0.9%	-2.1%	-2.4%	-1.7%	-1.2%	-2.7%
	Suriname	-1.9%	-2.0%	-2.6%	-2.7%	-1.8%	-2.0%	-3.3%	-3.3%	-0.3%	-0.4%	-2.9%	-2.8%	-2.4%	-2.4%	-1.3%	-1.8%	-2.5%	-2.5%	0.3%	-0.1%	-0.1%	-0.1%	-1.4%	-1.4%	-0.2%	-0.2%	-0.6%	-0.3%	-2.3%	-2.4%	-1.3%	-1.1%	-1.4%
	Syrian Arab Republic	-2.7%	-2.7%	-2.7%	-2.6%	-2.7%	-2.7%	-3.3%	-3.3%	-2.3%	-2.3%	-3.1%	-3.1%	-2.6%	-2.7%	-0.1%	1.1%	-2.8%	-2.9%	1.9%	1.8%	-0.3%	-0.2%	-2.1%	-2.1%	-0.3%	-0.4%	-1.5%	-1.7%	-2.4%	-2.5%	-2.6%	-2.7%	-2.7%
	Thailand	-2.1%	-2.2%	-2.2%	-2.5%	-2.6%	-2.7%	-3.3%	-3.3%	1.3%	0.4%	-2.9%	-2.9%	-2.6%	-2.6%	3.0%	3.3%	-2.9%	-3.0%	-2.4%	-2.7%	-0.2%	-0.3%	-2.3%	-2.3%	-0.3%	-0.3%	-1.5%	-1.7%	-2.2%	-1.8%	-1.8%	-2.1%	-1.7%
	Tokelau	-2.2%	-2.1%	-2.1%	-2.0%	-2.6%	-2.7%			-1.5%	-1.5%	-2.6%	-2.5%	-2.4%	-2.4%	37.1%	31.6%	-2.5%	-2.8%	-0.6%	0.0%	-0.1%	-0.1%	-1.2%	-1.2%	-0.2%	-0.2%	-1.3%	-1.1%	-1.5%	-1.4%	-1.6%	-1.4%	-2.4%
	Tonga	-1.3%	-1.3%	-1.4%	-1.1%	-1.8%	-1.7%			-0.7%	-0.7%	-1.4%	-1.5%	-1.6%	-1.5%	9.3%	10.0%	-1.8%	-1.6%	-1.2%	-1.7%	-0.0%	-0.0%	-0.7%	-0.7%	-0.1%	-0.1%	-0.7%	-0.3%	-2.1%	-2.0%	-0.7%	-0.5%	-1.3%
	Tunisia	-2.7%	-2.5%	-2.0%	-1.7%	-3.1%	-3.0%			-1.6%	-1.6%	-3.0%	-2.9%	-2.8%	-2.6%	25.7%	26.4%	-3.0%	-2.9%	-1.9%	-2.0%	-0.1%	-0.1%	-1.8%	-1.9%	-0.1%	-0.2%	-1.5%	-1.6%	-2.6%	-2.7%	-2.9%	-2.6%	-2.9%
	Turkmenistan	-2.7%	-2.7%	-3.1%	-3.1%	-2.7%	-2.7%	-3.3%	-3.3%	1.5%	0.9%	-2.6%	-2.7%	-2.0%	-2.1%	-1.5%	-1.1%	-2.3%	-2.4%	-1.7%	-1.7%	-0.0%	-0.1%	-0.9%	-0.6%	-1.8%	-1.3%	-0.0%	0.3%	-2.9%	-2.7%	-1.4%	-1.5%	-0.9%
	Uzbekistan	-2.3%	-2.3%	-3.1%	-3.1%	-2.2%	-2.2%	-3.3%	-3.3%	1.9%	2.2%	-2.6%	-2.7%	-2.4%	-2.4%	-0.2%	1.7%	-2.0%	-2.2%	-1.0%	-0.8%	-0.1%	-0.1%	-0.8%	-1.0%	-2.1%	-2.1%	0.7%	1.2%	-3.0%	-2.9%	-1.6%	-1.4%	-1.0%
	Viet Nam	-2.5%	-2.5%	-2.5%	-2.8%	-2.7%	-2.7%	-3.1%	-3.1%	-1.5%	-1.6%	-3.0%	-3.1%	-2.2%	-2.0%	0.3%	13.2%	-2.6%	-2.5%	-2.7%	-2.6%	-0.1%	-0.1%	-1.2%	-1.4%	-0.4%	-0.4%	-2.2%	-2.5%	-2.5%	-1.9%	-2.2%	-2.1%	-2.6%

0 to 24 years, percentage change in DALYs/100,000, for males and females, 1990 to 2019

High-middle SDI

	Total communicable		Enteric infections		Lower respiratory infections		Malaria		Neonatal sepsis and other neonatal infections		Vaccine Preventable disease		Meningitis & Encephalitis		HIV/AIDS	
	Female	Male	Female	Male	Female	Male	Female	Male	Female	Male	Female	Male	Female	Male	Female	Male
American Samoa	-1.4%	-1.4%	-1.2%	-1.0%	-1.9%	-1.9%			-1.0%	-2.1%	-2.0%	-1.9%	-1.1%	-1.6%	10.2%	8.3%
Antigua and Barbuda	-1.2%	-0.8%	-1.5%	-0.7%	-1.2%	-0.5%			-1.4%	-1.0%	-0.9%	-1.7%	-2.2%	-2.1%	-1.0%	-0.5%
Argentina	-1.8%	-1.9%	-2.3%	-2.2%	-2.1%	-2.0%	-3.3%	-3.3%	-2.1%	-2.2%	-1.9%	-1.9%	-2.4%	-2.4%	1.7%	0.1%
Bahamas	-1.2%	-1.4%	-1.5%	-1.6%	-2.1%	-1.8%			-0.9%	-1.4%	-1.1%	-1.0%	-2.3%	-2.4%	0.7%	-0.3%
Bahrain	-2.0%	-1.9%	-1.0%	-1.0%	-2.6%	-2.5%			-0.9%	-2.2%	-3.1%	-3.1%	-2.6%	-2.4%	-1.4%	-1.4%
Barbados	-0.6%	-1.0%	-0.7%	-0.8%	-1.3%	-1.5%			0.8%	0.3%	-2.0%	-1.9%	-2.2%	-2.5%	-0.6%	-2.0%
Belarus	-1.7%	-1.9%	-1.1%	-1.3%	-2.9%	-2.9%			-0.8%	-1.0%	-2.2%	-1.9%	-1.7%	-1.8%	1.8%	2.3%
Bosnia and Herzegovina	-1.4%	-1.4%	-1.2%	-1.1%	-2.5%	-2.3%			-0.3%	-0.5%	-1.8%	-1.7%	-2.1%	-2.2%	1.2%	-0.3%
Bulgaria	-1.9%	-2.0%	-0.9%	-1.0%	-2.5%	-2.5%			14.6%	16.4%	4.3%	3.7%	-1.8%	-1.9%	2.1%	1.7%
Chile	-2.2%	-2.4%	-1.8%	-2.0%	-3.0%	-3.1%			-1.9%	-2.1%	0.6%	0.3%	-2.6%	-2.7%	4.2%	2.9%
Cook Islands	-2.3%	-2.3%	-1.1%	-0.9%	-3.0%	-2.9%			-1.9%	-1.9%	-2.6%	-2.5%	-2.2%	-2.1%	47.9%	41.1%
Croatia	-0.9%	-1.0%	-0.7%	-1.0%	-2.7%	-2.7%			4.0%	4.4%	-1.0%	-0.6%	-2.5%	-2.5%	5.6%	-0.1%
Dominica	-0.3%	-0.4%	-1.3%	-1.4%	0.1%	0.1%			0.4%	0.1%	2.9%	3.4%	-1.6%	-1.7%	0.5%	0.1%
Georgia	-2.5%	-2.6%	-2.1%	-2.3%	-3.1%	-3.1%	-3.3%	-3.3%	3.1%	2.1%	1.2%	0.7%	-1.8%	-2.0%	20.3%	44.3%
Greece	-0.5%	-0.8%	0.1%	0.1%	-0.7%	-1.1%			-2.9%	-2.9%	-2.4%	-2.1%	-1.6%	-1.5%	0.7%	-1.8%
Greenland	-2.0%	-2.1%	-0.1%	0.1%	-2.8%	-2.9%			-0.4%	-1.4%	-2.4%	-2.4%	-2.9%	-2.6%	2.8%	0.7%
Hungary	-1.5%	-1.7%	-0.5%	-0.4%	-2.6%	-2.7%			-1.9%	-0.2%	-2.0%	-1.7%	-2.6%	-2.7%	-2.7%	-2.7%
Israel	-1.1%	-1.2%	0.1%	0.2%	-2.5%	-2.4%			-1.9%	-1.6%	-0.3%	0.1%	-2.4%	-2.5%	-2.2%	-1.8%
Italy	-0.9%	-1.2%	0.5%	0.4%	-2.7%	-2.7%			-0.9%	-0.7%	-2.5%	-2.0%	-2.1%	-2.2%	-2.5%	-2.9%
Jordan	-2.2%	-2.2%	-1.9%	-1.8%	-2.4%	-2.5%			-1.8%	-1.4%	-2.9%	-2.9%	-2.2%	-2.1%	5.4%	5.4%
Kazakhstan	-2.6%	-2.6%	-2.9%	-3.0%	-2.9%	-2.9%			0.6%	0.1%	-1.9%	-2.0%	-2.0%	-2.1%	4.1%	0.8%
Lebanon	-2.3%	-2.2%	-1.6%	-1.4%	-2.8%	-2.6%			-1.4%	-1.3%	-2.9%	-2.9%	-2.5%	-2.3%	0.5%	0.3%
Libya	-2.5%	-2.6%	-2.4%	-2.3%	-2.8%	-2.9%			-2.2%	-2.3%	-3.1%	-3.1%	-2.6%	-2.7%	10.1%	6.2%
Malaysia	-2.0%	-1.9%	-1.8%	-1.9%	-2.5%	-2.4%	-3.2%	-3.2%	-1.7%	-1.8%	-3.0%	-3.0%	-2.2%	-2.2%	77.6%	62.7%
Malta	-0.8%	-1.0%	-0.0%	-0.1%	-1.7%	-1.7%			-0.5%	-0.8%	-2.1%	-1.9%	-1.9%	-2.0%	-1.0%	-1.9%
Mauritius	-1.4%	-1.1%	-2.2%	-2.3%	-2.3%	-2.0%			-0.9%	0.5%	-1.6%	-1.8%	-1.9%	-1.7%	9.1%	22.7%
Montenegro	-1.7%	-1.5%	-0.7%	-0.9%	-2.8%	-2.6%			-1.3%	-1.3%	-2.0%	-1.7%	-2.3%	-2.2%	7.3%	7.5%
Niue	-0.9%	-1.0%	-1.3%	-1.3%	-1.2%	-1.3%			-0.6%	-0.8%	-1.6%	-1.4%	-2.0%	-2.0%	44.2%	38.4%
North Macedonia	-2.6%	-2.6%	-2.9%	-2.9%	-3.0%	-3.0%			14.2%	18.3%	-1.5%	-1.3%	-2.8%	-2.8%	25.6%	23.7%
Northern Mariana Islands	-0.8%	-1.0%	-0.4%	-0.5%	-1.6%	-1.7%			0.6%	-1.3%	-1.2%	-1.1%	-0.4%	-0.6%	12.2%	8.3%
Oman	-2.6%	-2.5%	-2.1%	-2.2%	-2.8%	-2.7%	-3.3%	-3.3%	-1.5%	-1.6%	-3.1%	-3.2%	-2.2%	-1.9%	9.1%	8.2%
Palau	-1.8%	-1.8%	-1.5%	-1.6%	-2.1%	-2.2%			-1.0%	-1.7%	-2.1%	-2.1%	-2.2%	-1.8%	41.3%	34.4%
Poland	-1.8%	-2.0%	-0.8%	-0.9%	-2.6%	-2.6%			-2.6%	-2.7%	-2.2%	-2.1%	-2.8%	-2.9%	10.1%	8.2%
Portugal	-1.7%	-2.0%	-0.9%	-1.4%	-2.7%	-2.8%			-2.2%	-2.2%	-2.0%	-2.6%	-2.8%	-2.8%	1.4%	-1.7%
Republic of Moldova	-2.1%	-2.2%	-2.2%	-2.4%	-2.6%	-2.6%			-0.5%	-0.6%	-1.8%	-1.5%	-2.7%	-2.8%	21.7%	2.5%
Romania	-2.5%	-2.5%	-1.9%	-2.0%	-2.7%	-2.6%			-1.1%	-0.7%	-1.4%	-2.2%	-2.7%	-2.9%	-2.5%	-2.5%
Russian Federation	-1.4%	-1.7%	-1.1%	-1.5%	-2.4%	-2.5%			-0.2%	-0.5%	-2.3%	-2.1%	-2.1%	-2.3%	5.2%	2.7%
Saint Kitts and Nevis	-1.6%	-1.3%	-2.4%	-2.4%	-2.0%	-1.8%			-0.0%	-0.1%	-2.3%	-1.7%	-2.6%	-2.6%	11.9%	9.5%
Saudi Arabia	-2.9%	-2.9%	-2.9%	-2.9%	-2.8%	-2.8%	-3.3%	-3.2%	-2.7%	-2.7%	-3.3%	-3.3%	-2.6%	-2.7%	3.1%	2.6%
Serbia	-2.2%	-2.4%	-0.8%	-0.7%	-3.1%	-3.1%			-2.6%	-2.8%	-2.5%	-2.4%	-2.7%	-2.9%	1.9%	1.7%
Seychelles	-1.3%	-1.3%	-1.3%	-1.1%	-0.6%	-0.4%			-0.4%	-0.9%	-2.3%	-2.3%	-3.0%	-2.9%	10.3%	5.8%
Spain	-1.0%	-1.5%	-0.1%	-0.2%	-2.4%	-2.7%			-0.7%	-0.8%	-0.5%	-0.6%	-2.5%	-2.5%	-2.9%	-3.1%
Sri Lanka	-2.3%	-2.2%	-2.3%	-2.4%	-2.6%	-2.5%	-3.3%	-3.3%	-0.3%	-0.5%	-3.1%	-3.1%	-2.5%	-2.5%	0.6%	1.5%
Trinidad and Tobago	-1.3%	-1.4%	-1.7%	-1.6%	-2.3%	-2.2%			-0.7%	-0.8%	-1.3%	-1.6%	-2.4%	-2.6%	3.0%	0.2%
Turkey	-3.0%	-2.9%	-2.4%	-2.4%	-3.2%	-3.2%	-3.3%	-3.3%	-1.9%	-1.9%	-3.2%	-3.2%	-3.0%	-3.0%	50.6%	54.4%
Ukraine	-0.6%	-0.9%	-0.4%	-0.8%	-1.7%	-2.0%			-0.8%	-0.7%	1.5%	0.6%	-0.9%	-1.5%	13.4%	2.1%
United States Virgin Islands	-1.1%	-1.5%	-0.5%	-0.9%	-2.2%	-2.6%			-0.6%	-0.4%	-1.9%	-2.1%	-1.9%	-2.6%	0.8%	-0.7%
Uruguay	-1.7%	-1.7%	-2.0%	-2.2%	-2.2%	-2.1%			-1.7%	-1.7%	-1.6%	1.1%	-2.4%	-2.6%	4.7%	9.3%

	Tuberculosis		Neglected Tropical diseases		Infectious skin conditions		Sexually transmitted infections excluding HIV		Upper respiratory infections		Other unspecified infectious diseases		Hepatitis		Rheumatic heart disease		Maternal sepsis and other maternal infections
	Female	Male	Female	Male	Female	Male	Female	Male	Female	Male	Female	Male	Female	Male	Female	Male	Female
American Samoa	-1.5%	-2.3%	1.0%	-1.2%	-0.0%	0.1%	-1.8%	-1.8%	-0.1%	-0.1%	-0.7%	0.5%	0.0%	0.4%	-0.3%	-0.7%	-1.7%
Antigua and Barbuda	-2.1%	-2.1%	-0.9%	-0.1%	-0.0%	0.2%	0.0%	-1.8%	-0.2%	-0.2%	-0.8%	-0.1%	-2.2%	-1.8%	-0.9%	-0.5%	-1.5%
Argentina	-2.6%	-2.7%	-2.4%	-2.3%	-0.1%	0.0%	-1.9%	-2.3%	-0.1%	-0.1%	-1.0%	-0.8%	-2.0%	-1.5%	-0.8%	-0.9%	-1.9%
Bahamas	-2.2%	-2.0%	-0.4%	-0.6%	-0.0%	-0.1%	-0.1%	-0.6%	-0.4%	-0.1%	-0.3%	-0.1%	-1.9%	-1.6%	-0.6%	-0.6%	-1.7%
Bahrain	-2.5%	-2.5%	-1.8%	-1.9%	-0.8%	-0.5%	-1.3%	-1.3%	-0.2%	-0.2%	-1.7%	-1.6%	-2.0%	-1.1%	-2.0%	-2.3%	-2.6%
Barbados	-2.2%	-2.3%	0.3%	0.9%	0.0%	-0.1%	-1.5%	-1.6%	-0.2%	-0.1%	-0.8%	-0.2%	-2.1%	-1.9%	-1.0%	-0.9%	-1.7%
Belarus	-2.2%	-2.0%	-1.3%	-1.4%	0.1%	0.0%	-1.1%	-1.9%	-0.5%	-0.7%	1.7%	1.9%	-1.5%	-0.8%	-2.8%	-2.8%	-2.0%
Bosnia and Herzegovina	-2.6%	-2.3%	-1.6%	-1.8%	-0.1%	-0.0%	-1.5%	-1.5%	-0.3%	-0.2%	-1.3%	-1.0%	-1.2%	-0.9%	-2.4%	-2.2%	-2.8%
Bulgaria	-2.1%	-2.2%	-1.8%	-2.3%	0.1%	0.0%	-0.6%	-0.4%	-0.3%	-0.4%	-1.1%	-1.2%	-1.2%	-0.9%	-2.6%	-2.6%	-2.4%
Chile	-2.6%	-2.8%	-2.5%	-2.4%	-0.0%	-0.0%	-1.2%	-2.2%	-0.4%	-0.4%	-0.5%	-0.4%	-2.2%	-1.8%	-2.6%	-2.7%	-2.8%
Cook Islands	-2.4%	-2.4%	-2.8%	-3.0%	-0.0%	-0.0%	-1.7%	-1.5%	-0.2%	-0.2%	-1.1%	-1.2%	-1.2%	-1.2%	-2.3%	-2.0%	-1.1%
Croatia	-2.6%	-2.9%	-1.3%	-1.4%	-0.0%	-0.0%	-0.1%	0.0%	-0.1%	-0.1%	-0.9%	-0.9%	-1.1%	-1.2%	-2.7%	-2.6%	-2.2%
Dominica	-1.8%	-1.4%	-0.6%	-0.9%	-0.0%	-0.0%	-0.7%	-0.7%	-0.4%	-0.2%	0.1%	1.0%	-2.3%	-1.8%	-0.7%	-0.6%	-0.6%
Georgia	-2.2%	-2.3%	-0.2%	0.2%	0.1%	0.1%	-0.3%	-0.2%	-0.1%	-0.3%	0.5%	1.3%	-1.7%	-0.5%	-1.0%	-0.9%	-0.2%
Greece	-2.2%	-2.3%	-1.0%	-1.0%	0.0%	-0.0%	-0.3%	-0.6%	-0.0%	-0.0%	-0.0%	0.0%	-0.5%	-0.6%	-1.8%	-2.1%	-2.3%
Greenland	-2.9%	-2.9%	-1.1%	-1.0%	0.1%	0.1%	-1.1%	-1.2%	-0.1%	-0.0%	-1.5%	-1.9%	-1.8%	-0.2%	-2.6%	-2.1%	-1.7%
Hungary	-2.5%	-2.5%	-1.2%	-1.3%	0.0%	0.0%	-0.2%	-0.2%	-1.1%	-1.3%	-1.0%	-1.0%	-1.6%	-1.8%	-2.5%	-2.8%	-2.1%
Israel	-2.7%	-2.8%	-1.5%	-1.3%	0.0%	-0.0%	-0.4%	-1.5%	-0.0%	-0.0%	-0.3%	0.1%	-2.4%	-2.1%	-2.3%	-2.0%	-2.2%
Italy	-2.5%	-2.6%	-0.8%	-1.0%	0.2%	0.1%	-0.3%	-2.4%	-0.1%	-0.1%	-0.0%	-0.0%	-0.9%	-1.5%	-2.1%	-2.2%	-1.8%
Jordan	-2.9%	-2.8%	-1.9%	-2.2%	-0.1%	-0.1%	-1.3%	-1.2%	-0.2%	-0.2%	-1.7%	-1.8%	-2.2%	-2.4%	-2.6%	-2.2%	-2.5%
Kazakhstan	-2.6%	-2.8%	-1.5%	-1.0%	0.1%	0.1%	-0.9%	-1.2%	-0.8%	-0.4%	1.9%	3.5%	-2.8%	-2.6%	-2.8%	-2.9%	-0.8%
Lebanon	-2.8%	-2.7%	-2.4%	-2.6%	-0.0%	-0.0%	-0.7%	-0.6%	-0.1%	-0.1%	-1.5%	-1.6%	-2.5%	-2.0%	-2.7%	-2.4%	-2.5%
Libya	-2.5%	-2.5%	-1.1%	-1.3%	-0.2%	-0.2%	-1.3%	-1.4%	-0.1%	-0.1%	-1.2%	-1.7%	-2.5%	-2.2%	-2.3%	-2.3%	-2.7%
Malaysia	-2.0%	-2.2%	-1.8%	-1.9%	-0.1%	-0.1%	-1.7%	-1.6%	-0.3%	-0.3%	-0.8%	0.3%	-1.2%	-0.6%	-2.0%	-2.1%	-2.8%
Malta	-1.4%	-1.3%	-1.8%	-1.8%	-0.1%	-0.0%	0.2%	-0.5%	-0.2%	-0.4%	-0.7%	-0.6%	-0.5%	-0.4%	-1.0%	-1.4%	-1.4%
Mauritius	-1.7%	-2.0%	-0.3%	-0.1%	-0.0%	-0.0%	-0.0%	0.1%	-0.3%	-0.3%	-1.2%	-1.0%	-1.7%	-1.2%	-2.1%	-1.8%	-1.9%
Montenegro	-2.5%	-2.5%	-0.8%	-1.0%	-0.1%	-0.1%	-0.7%	-0.7%	-0.2%	-0.0%	-1.2%	-1.3%	-0.6%	-0.8%	-1.7%	-2.0%	-1.7%
Niue	-2.0%	-2.1%	0.8%	-0.3%	-0.1%	-0.1%	0.0%	-0.1%	-0.1%	-0.2%	-0.9%	-0.4%	-1.4%	-1.4%	-0.7%	-0.6%	-1.6%
North Macedonia	-3.1%	-3.0%	-1.5%	-1.4%	-0.1%	-0.1%	-0.5%	-0.5%	-0.0%	-0.2%	-1.7%	-1.9%	-1.1%	-0.9%	-2.6%	-2.4%	-2.4%
Northern Mariana Islands	-1.8%	-2.4%	1.7%	2.0%	0.1%	0.1%	-1.3%	-1.6%	-0.0%	-0.1%	0.1%	0.6%	-0.9%	-0.6%	-0.7%	0.2%	-1.8%
Oman	-2.8%	-2.8%	-2.3%	-2.4%	-0.2%	-0.2%	-1.6%	-1.6%	-0.3%	-0.2%	-2.0%	-2.1%	-2.6%	-1.7%	-2.6%	-2.4%	-2.6%
Palau	-1.9%	-2.1%	1.0%	1.5%	-0.0%	-0.0%	-0.3%	-0.5%	-0.0%	-0.1%	-0.9%	-0.7%	-1.5%	-1.7%	-1.3%	-1.1%	-1.5%
Poland	-2.6%	-2.7%	-1.5%	-1.5%	-0.1%	-0.1%	0.2%	-0.4%	-0.5%	-0.7%	-1.5%	-1.5%	-1.3%	-1.4%	-2.8%	-2.9%	-2.3%
Portugal	-2.7%	-2.9%	-2.0%	-1.9%	-0.0%	-0.1%	-1.6%	-2.6%	-0.5%	-0.6%	-1.2%	-1.2%	-1.9%	-2.0%	-2.8%	-2.8%	-2.1%
Republic of Moldova	-2.0%	-2.0%	-1.5%	-1.7%	-0.2%	-0.3%	-1.8%	-0.2%	-0.9%	-0.4%	1.1%	1.2%	-3.0%	-2.9%	-2.7%	-2.7%	-2.5%
Romania	-2.3%	-2.5%	-1.9%	-2.2%	0.1%	0.0%	-1.3%	-0.5%	-0.9%	-0.9%	-2.4%	-2.3%	-2.9%	-2.8%	-2.7%	-2.6%	-2.5%
Russian Federation	-1.5%	-2.0%	-1.1%	-1.2%	-0.0%	0.0%	-1.0%	-1.0%	-0.9%	-1.1%	-0.7%	-0.6%	-1.7%	-1.3%	-2.6%	-2.8%	-2.4%
Saint Kitts and Nevis	-2.6%	-2.3%	-0.7%	-0.8%	-0.1%	-0.1%	-2.0%	-2.2%	-0.3%	-0.7%	-1.0%	-0.5%	-2.7%	-1.6%	-2.3%	-1.8%	-1.6%
Saudi Arabia	-2.7%	-2.6%	-2.4%	-2.5%	-0.2%	-0.2%	-2.0%	-2.1%	-0.2%	-0.2%	-1.5%	-1.8%	-2.3%	-1.9%	-2.6%	-2.7%	-2.7%
Serbia	-2.9%	-3.0%	-2.1%	-2.4%	-0.1%	-0.0%	-1.4%	-1.7%	-0.1%	-0.1%	-1.7%	-1.8%	-1.7%	-1.8%	-2.7%	-2.7%	-2.2%
Seychelles	-1.3%	-1.7%	0.6%	1.1%	-0.0%	-0.0%	-0.7%	-0.5%	-0.2%	-0.2%	-0.2%	2.7%	-2.0%	-1.5%	-1.8%	-2.0%	-1.9%
Spain	-2.7%	-3.0%	-1.9%	-1.7%	0.2%	0.1%	-1.3%	-2.9%	-0.0%	-0.0%	-0.4%	-0.5%	-1.5%	-2.2%	-2.4%	-2.6%	-1.9%
Sri Lanka	-2.5%	-2.5%	-1.6%	-1.8%	-0.1%	-0.0%	-1.4%	-1.4%	-0.3%	-0.3%	-1.8%	-1.4%	-1.9%	-1.7%	-2.5%	-2.4%	-2.7%
Trinidad and Tobago	-2.3%	-1.9%	-0.6%	0.3%	-0.1%	-0.1%	0.6%	0.4%	-0.2%	-0.2%	-0.3%	-0.1%	-2.2%	-2.1%	-1.4%	-1.4%	-2.3%
Turkey	-3.0%	-3.2%	-2.5%	-2.8%	-0.2%	-0.3%	-2.0%	-1.9%	-0.4%	-0.3%	-2.1%	-2.0%	-3.0%	-2.9%	-2.6%	-2.0%	-2.9%
Ukraine	2.3%	4.2%	-0.4%	-0.6%	0.0%	0.1%	0.0%	-1.4%	0.0%	0.0%	6.9%	4.7%	-0.9%	0.1%	0.3%	0.7%	-2.0%
United States Virgin Islands	-2.4%	-1.9%	-0.2%	-0.5%	0.0%	-0.1%	-1.3%	-1.4%	-0.3%	-0.2%	-0.6%	-1.1%	-1.8%	-1.8%	-2.4%	-2.4%	-2.0%
Uruguay	-2.2%	-2.1%	-1.5%	-1.4%	-0.1%	-0.1%	-2.8%	-3.1%	-0.2%	-0.1%	-1.2%	-1.2%	-1.9%	-1.2%	-1.7%	-1.5%	-1.4%

0 to 24 years, percentage change in DALYs/100,000, for males and females, 1990 to 2019

		Total communicable		Enteric infections		Lower respiratory infections		Malaria		Neonatal sepsis and other neonatal infections		Vaccine Preventable disease		Meningitis & Encephalitis		HIV/AIDS		Tuberculosis	
		Female	Male	Female	Male	Female	Male	Female	Male	Female	Male	Female	Male	Female	Male	Female	Male	Female	Male
High SDI	Andorra	-1.2%	-1.2%	-0.2%	-0.4%	-2.5%	-2.5%			-1.6%	-1.7%	-3.1%	-3.1%	-2.0%	-2.3%	-0.6%	-0.6%	-2.4%	-2.2%
	Australia	-0.7%	-1.0%	0.3%	0.5%	-1.9%	-2.0%			-2.2%	-2.5%	-0.4%	-0.0%	-2.2%	-2.4%	-0.3%	-2.9%	-1.7%	-2.1%
	Austria	-0.6%	-1.0%	0.3%	0.3%	-2.6%	-2.6%			1.5%	-0.5%	1.2%	1.1%	-2.0%	-2.3%	-1.1%	-2.0%	-2.4%	-2.2%
	Belgium	-0.6%	-0.9%	0.1%	0.0%	-1.9%	-2.1%			-1.0%	-2.0%	-1.7%	-1.6%	-1.8%	-1.8%	-2.1%	-2.3%	-2.3%	-2.2%
	Bermuda	-1.3%	-1.5%	-0.1%	-0.9%	-1.9%	-2.3%			-1.7%	-0.9%	-0.9%	-0.8%	-2.5%	-2.7%	-1.3%	-2.2%	-2.3%	-2.3%
	Brunei Darussalam	-0.8%	-1.2%	-0.6%	-0.8%	-0.6%	-1.3%			-0.2%	-0.4%	-2.1%	-2.4%	-1.9%	-1.9%	9.4%	14.2%	-2.3%	-2.7%
	Canada	-0.3%	-0.6%	1.0%	0.8%	-1.9%	-2.1%			-0.1%	-1.0%	-0.3%	-0.6%	-2.0%	-1.8%	0.5%	-2.6%	-1.5%	-1.6%
	Cyprus	-1.5%	-1.7%	-0.3%	-0.4%	-2.8%	-2.8%			-2.2%	-2.3%	-3.1%	-3.1%	-2.8%	-2.9%	65.6%	10.7%	-2.4%	-3.0%
	Czechia	-0.9%	-1.0%	-0.5%	-0.4%	-2.3%	-2.3%			3.8%	5.0%	1.8%	1.3%	-2.2%	-2.2%	10.6%	5.9%	-2.4%	-2.5%
	Denmark	-0.9%	-1.1%	0.0%	-0.0%	-2.4%	-2.4%			-0.3%	-1.2%	-1.1%	-1.8%	-2.8%	-2.8%	9.5%	3.0%	-2.4%	-2.3%
	Estonia	-1.4%	-2.0%	0.5%	-0.7%	-2.4%	-2.7%			-2.0%	-2.8%	-2.7%	-2.6%	-2.6%	-2.8%	136.5%	140.6%	-2.3%	-2.6%
	Finland	-0.9%	-1.1%	-0.5%	-0.5%	-2.8%	-2.9%			-1.7%	-1.4%	-0.9%	-0.8%	-2.2%	-2.5%	-2.8%	-2.2%	-1.8%	-2.5%
	France	-0.9%	-1.3%	-0.8%	-1.0%	-2.2%	-2.4%			-1.5%	-1.6%	-1.6%	-1.7%	-2.1%	-2.3%	-2.6%	-3.1%	-2.4%	-2.5%
	Germany	-0.7%	-1.1%	0.1%	-0.0%	-2.1%	-2.2%			-1.4%	-1.8%	-1.1%	-1.5%	-2.4%	-2.5%	-2.6%	-2.8%	-2.5%	-2.3%
	Guam	-0.5%	-0.5%	-0.1%	-0.4%	-0.7%	-0.8%			-0.6%	0.1%	-1.0%	-0.8%	-0.5%	-1.1%	12.9%	9.9%	-1.2%	-2.0%
	Iceland	-1.0%	-1.1%	-0.2%	-0.3%	-2.2%	-2.3%			-1.7%	-2.3%	1.1%	0.8%	-2.5%	-2.4%	-2.3%	-2.3%	-2.5%	-2.3%
	Ireland	-0.8%	-1.0%	0.2%	0.2%	-2.4%	-2.6%			1.6%	0.7%	-2.1%	-2.1%	-2.6%	-2.6%	-0.3%	-1.9%	-2.1%	-2.4%
	Japan	-0.7%	-0.9%	-0.9%	-1.0%	-1.9%	-1.9%			-1.7%	-1.6%	-2.3%	-2.4%	-1.9%	-2.0%	4.8%	5.1%	-2.3%	-2.5%
	Kuwait	-1.7%	-1.6%	-1.3%	-1.0%	-2.3%	-2.2%			-1.9%	-1.8%	-2.7%	-2.7%	-2.2%	-2.1%	0.0%	-2.3%	-2.7%	-2.7%
	Latvia	-1.6%	-1.8%	-0.8%	-0.8%	-2.2%	-2.5%			-1.8%	-1.2%	-1.9%	-1.6%	-2.6%	-2.8%	8.1%	1.4%	-2.4%	-2.5%
	Lithuania	-0.9%	-1.2%	-0.3%	-0.8%	-1.7%	-2.0%			7.3%	3.9%	-2.3%	-2.0%	-2.5%	-2.6%	0.9%	17.4%	-2.0%	-2.1%
	Luxembourg	-0.9%	-1.3%	-0.2%	-0.3%	-2.4%	-2.6%			-2.2%	-2.3%	-2.0%	-2.2%	-2.3%	-2.6%	-1.9%	-2.7%	-2.2%	-2.3%
	Monaco	-0.8%	-1.1%	0.1%	-0.0%	-2.2%	-2.2%			-1.4%	-1.2%	-1.1%	-1.1%	-2.0%	-2.5%	-2.2%	-2.4%	-2.4%	-2.5%
	Netherlands	-0.6%	-1.0%	0.2%	0.1%	-1.9%	-2.1%			0.5%	-0.8%	1.3%	0.8%	-2.3%	-2.4%	-1.9%	-2.9%	-2.3%	-2.3%
	New Zealand	-0.9%	-1.3%	0.1%	-0.0%	-2.6%	-2.8%			0.7%	2.0%	-1.6%	-1.7%	-1.9%	-2.3%	-1.9%	-2.7%	-2.3%	-2.3%
	Norway	-0.8%	-1.3%	-0.0%	-0.1%	-2.7%	-2.7%			-0.5%	-2.0%	-1.5%	-1.4%	-2.7%	-2.7%	3.6%	-2.6%	-2.1%	-2.5%
	Puerto Rico	-1.2%	-1.2%	0.6%	0.7%	-2.3%	-2.4%			-0.8%	0.2%	-1.7%	-1.6%	-2.6%	-2.8%	-2.3%	-2.6%	-2.5%	-2.5%
	Qatar	-2.5%	-2.5%	-1.6%	-1.7%	-2.6%	-2.7%			-2.0%	-2.1%	-3.2%	-3.2%	-2.7%	-2.4%	-2.6%	-2.2%	-2.8%	-2.8%
	Republic of Korea	-2.1%	-2.2%	-2.4%	-2.5%	-3.0%	-3.0%	-3.2%	-3.2%	-1.9%	-1.7%	-3.0%	-3.0%	-2.7%	-2.7%	-0.5%	0.7%	-3.2%	-3.2%
	San Marino	-0.7%	-0.8%	0.1%	0.1%	-1.8%	-2.2%			-1.3%	-1.3%	5.1%	4.9%	-1.9%	-1.7%	-1.5%	-2.1%	-2.3%	-2.3%
	Singapore	-1.4%	-1.7%	-2.4%	-2.4%	-2.4%	-2.5%			2.3%	-0.4%	-1.2%	-1.3%	-2.5%	-2.5%	2.3%	1.3%	-2.8%	-2.9%
	Slovakia	-1.6%	-1.6%	-0.6%	-0.6%	-2.5%	-2.5%			-0.4%	0.3%	5.4%	4.7%	-2.1%	-1.7%	7.8%	7.4%	-2.5%	-2.7%
	Slovenia	-0.9%	-1.0%	-0.6%	-0.5%	-2.6%	-2.8%			0.1%	0.9%	3.8%	3.7%	-2.3%	-2.4%	-1.4%	-2.3%	-2.4%	-2.6%
	Sweden	-0.6%	-0.9%	0.1%	-0.0%	-2.4%	-2.5%			-1.3%	-1.4%	-0.1%	-0.6%	-2.2%	-2.3%	-0.9%	-2.6%	-2.0%	-2.3%
	Switzerland	-1.0%	-1.3%	-0.0%	-0.3%	-2.7%	-2.8%			-1.7%	-0.9%	-1.6%	-1.9%	-2.8%	-2.9%	0.7%	-0.9%	-2.3%	-2.0%
	Taiwan (Province of China)	-1.0%	-1.1%	-0.9%	-0.9%	-2.2%	-2.1%			13.6%	9.0%	-2.5%	-2.5%	-2.1%	-2.2%	10.9%	34.3%	-2.0%	-2.1%
	United Arab Emirates	-2.3%	-2.2%	-1.2%	-1.2%	-2.9%	-2.7%	-3.3%	-3.3%	-2.4%	-2.2%	-3.1%	-3.1%	-2.8%	-2.7%	9.7%	8.1%	-2.6%	-2.7%
	United Kingdom	-0.9%	-1.2%	0.1%	0.0%	-2.0%	-2.2%			-1.8%	-2.1%	-1.7%	-1.5%	-2.3%	-2.3%	0.7%	-2.3%	-2.0%	-2.2%
	United States of America	-0.9%	-1.1%	0.1%	-0.5%	-1.9%	-1.9%			-1.1%	-1.2%	-0.9%	-1.1%	-2.2%	-2.3%	-2.8%	-2.9%	-2.4%	-2.3%

		Neglected Tropical diseases		Infectious skin conditions		Sexually transmitted infections excluding HIV		Upper respiratory infections		Other unspecified infectious diseases		Hepatitis		Rheumatic heart disease		Maternal sepsis and other maternal infections
		Female	Male	Female	Male	Female	Male	Female	Male	Female	Male	Female	Male	Female	Male	Female
High SDI	Andorra	-1.4%	-1.0%	0.1%	0.0%	-1.2%	-1.1%	-0.1%	-0.1%	-1.4%	-1.4%	-2.3%	-2.1%	-2.1%	-1.7%	-1.9%
	Australia	-1.0%	-1.0%	0.0%	0.0%	-1.0%	-1.9%	-0.1%	-0.1%	0.2%	-0.4%	-0.7%	-0.7%	-2.0%	-2.1%	-1.0%
	Austria	-1.4%	-1.4%	0.1%	-0.0%	-0.5%	-0.6%	-0.1%	-0.2%	1.1%	0.5%	-0.8%	-1.1%	-2.0%	-2.1%	-2.0%
	Belgium	-1.6%	-1.2%	0.1%	0.0%	-0.7%	-1.6%	-0.1%	-0.1%	0.3%	0.1%	-1.4%	-1.3%	-0.8%	-1.5%	-2.0%
	Bermuda	-1.3%	-0.8%	0.0%	-0.0%	-0.9%	-0.8%	-0.1%	-0.1%	-1.3%	-1.1%	-1.6%	-1.3%	-2.6%	-2.4%	-2.7%
	Brunei Darussalam	-0.9%	-1.8%	-0.1%	-0.0%	-0.6%	-0.9%	-0.3%	-0.2%	-0.1%	-0.3%	-1.7%	-1.9%	-1.7%	-2.5%	-2.0%
	Canada	-1.1%	-1.1%	0.0%	0.0%	-0.2%	-0.4%	-0.1%	-0.1%	-0.4%	-0.4%	-0.5%	-0.5%	-1.6%	-1.5%	-1.8%
	Cyprus	-2.3%	-2.3%	-0.1%	-0.1%	-1.8%	-1.9%	-0.1%	-0.1%	-1.5%	-1.8%	-1.1%	-1.1%	-2.1%	-2.3%	-3.0%
	Czechia	-1.1%	-1.2%	0.1%	0.1%	-0.7%	-0.8%	-0.5%	-0.5%	-1.1%	-1.0%	-1.1%	-1.4%	-2.6%	-2.8%	-2.5%
	Denmark	-1.5%	-1.1%	0.1%	0.0%	-0.6%	-0.4%	-0.0%	-0.1%	-0.4%	-0.2%	-1.4%	-0.4%	-2.1%	-2.5%	-1.9%
	Estonia	-1.3%	-1.6%	-0.0%	-0.1%	-1.2%	-2.4%	-0.2%	-0.2%	0.9%	0.1%	-2.0%	-1.9%	-2.3%	-3.0%	-2.5%
	Finland	-1.8%	-1.5%	0.0%	-0.1%	-0.6%	-0.4%	-0.1%	-0.1%	-1.0%	-1.1%	-0.9%	-0.4%	-1.8%	-2.2%	-2.2%
	France	-1.8%	-1.7%	0.1%	0.0%	-0.4%	-1.3%	-0.3%	-0.5%	-0.9%	-0.9%	-0.9%	-1.4%	-1.7%	-1.9%	-1.7%
	Germany	-1.4%	-1.5%	0.1%	-0.0%	-0.8%	-1.0%	-0.2%	-0.3%	-1.1%	-1.0%	-1.4%	-1.7%	-1.8%	-2.1%	-2.6%
	Guam	-0.6%	-0.3%	0.1%	0.1%	-0.7%	-0.7%	-0.0%	-0.0%	-0.1%	0.1%	-0.1%	-0.9%	-0.6%	-1.3%	-1.3%
	Iceland	-1.4%	-1.0%	0.0%	0.0%	-0.3%	-1.8%	-0.1%	-0.2%	-0.8%	-0.8%	-0.2%	-0.2%	-1.4%	-1.9%	-2.0%
	Ireland	-1.8%	-1.3%	0.0%	-0.0%	-0.4%	-0.7%	-0.1%	-0.1%	-0.4%	-0.7%	-0.6%	-0.4%	-2.2%	-1.9%	-1.4%
	Japan	-1.3%	-1.2%	0.0%	0.0%	0.4%	-0.4%	-0.1%	-0.1%	-0.5%	-0.4%	-1.0%	-1.5%	-1.7%	-2.0%	-1.3%
	Kuwait	-1.2%	-1.2%	-0.1%	-0.1%	-0.0%	-0.2%	-0.1%	-0.1%	-1.0%	-0.9%	-2.1%	-1.9%	-2.9%	-2.6%	-1.8%
	Latvia	-1.1%	-0.5%	0.0%	0.0%	-1.2%	-1.5%	-0.1%	-0.1%	0.5%	2.7%	-3.0%	-3.0%	-2.7%	-3.0%	-2.5%
	Lithuania	-0.7%	-1.1%	0.0%	-0.0%	-0.2%	-1.4%	-0.3%	-0.5%	0.3%	0.5%	-1.9%	-1.6%	-2.7%	-2.8%	-2.5%
	Luxembourg	-1.9%	-1.5%	0.1%	0.0%	-0.8%	-2.6%	-0.1%	-0.2%	-1.2%	-1.2%	-0.7%	-1.5%	-1.8%	-2.3%	-2.4%
	Monaco	-1.2%	-0.9%	0.2%	0.0%	-1.4%	-1.3%	0.0%	-0.0%	-0.8%	-0.7%	-0.4%	-0.5%	-1.2%	-1.5%	-2.2%
	Netherlands	-1.3%	-0.4%	0.1%	0.0%	-0.6%	-0.6%	-0.3%	-0.4%	0.3%	0.1%	-0.3%	-0.3%	-1.4%	-1.6%	-1.7%
	New Zealand	-1.0%	-0.7%	0.1%	0.0%	0.2%	-0.5%	-0.1%	-0.2%	-0.3%	-0.7%	-0.9%	-0.8%	-2.2%	-2.4%	-1.3%
	Norway	-1.2%	-1.2%	0.1%	0.0%	-0.6%	0.3%	-0.2%	-0.1%	-0.0%	-0.2%	-0.5%	-0.4%	-2.2%	-2.6%	-2.3%
	Puerto Rico	-0.8%	-0.3%	-0.1%	-0.1%	0.1%	0.4%	-0.2%	-0.2%	-0.6%	-0.3%	-1.4%	-1.3%	-2.5%	-1.5%	-2.5%
	Qatar	-2.0%	-2.3%	-0.1%	-0.3%	-1.4%	-1.8%	-0.1%	-0.3%	-1.7%	-1.9%	-1.1%	-1.3%	-2.8%	-2.8%	-2.8%
	Republic of Korea	-1.8%	-1.4%	-0.1%	-0.1%	0.0%	0.3%	-0.1%	-0.2%	-1.6%	-1.7%	-2.6%	-2.6%	-2.8%	-2.7%	-3.2%
	San Marino	-1.1%	-1.2%	0.1%	0.0%	-0.4%	-0.6%	-0.6%	-0.2%	-0.6%	-1.1%	-1.4%	-2.0%	-1.5%	-1.5%	-1.8%
	Singapore	-1.3%	-1.1%	0.1%	-0.0%	-1.0%	-2.0%	0.0%	0.1%	-1.6%	-1.6%	-1.5%	-1.8%	-2.9%	-2.8%	-2.8%
	Slovakia	-1.1%	-1.2%	-0.0%	-0.0%	-0.4%	-0.4%	-0.2%	-0.2%	-0.5%	-1.1%	-0.9%	-1.2%	-1.9%	-2.1%	-2.1%
	Slovenia	-1.6%	-1.9%	0.1%	0.1%	-0.6%	-0.5%	-0.2%	-0.2%	-0.7%	-0.7%	-1.0%	-0.9%	-2.0%	-2.0%	-2.4%
	Sweden	-1.2%	-0.7%	0.2%	0.1%	-0.7%	-1.1%	-0.1%	-0.1%	0.1%	-0.3%	-0.9%	-0.6%	-2.0%	-2.1%	-2.0%
	Switzerland	-1.0%	-1.0%	0.1%	0.0%	-0.3%	-0.6%	-0.0%	-0.1%	-0.3%	-0.6%	-1.3%	-1.6%	-2.2%	-2.6%	-2.1%
	Taiwan (Province of China)	-1.5%	-1.5%	0.0%	-0.0%	-0.2%	0.2%	-0.5%	-0.6%	0.1%	0.3%	-1.3%	-1.7%	-2.7%	-2.7%	-2.4%
	United Arab Emirates	-1.2%	-1.5%	-0.1%	-0.1%	-2.2%	-2.2%	-0.1%	-0.1%	-1.5%	-1.7%	-1.7%	-1.9%	-2.2%	-1.8%	-2.8%
	United Kingdom	-0.9%	-0.8%	0.1%	0.0%	-0.1%	-0.2%	-0.2%	-0.3%	-0.6%	-0.7%	0.2%	0.4%	-2.4%	-2.3%	-1.8%
	United States of America	0.2%	0.2%	0.0%	0.0%	-0.7%	-1.4%	-0.2%	-0.2%	-0.7%	-0.7%	-0.6%	-0.6%	-2.0%	-1.9%	-1.7%

S16: Relationship between enteric and lower respiratory tract disease burden for 0-24 year olds by country, 2019.

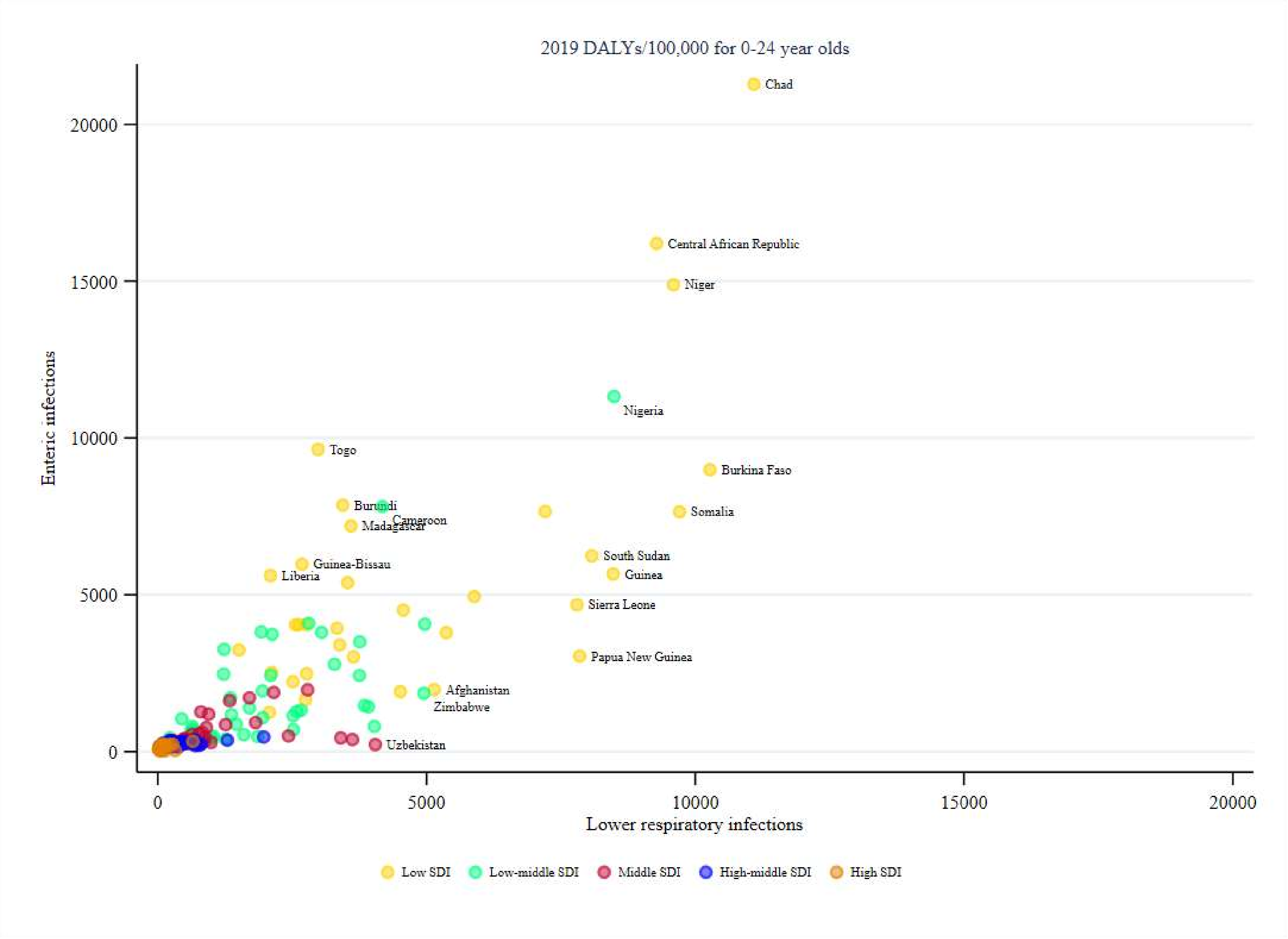

S17_1 Part A: Enteric Infections Incidence, YLD, death and DALY per 100 000 in 2019 for each age group

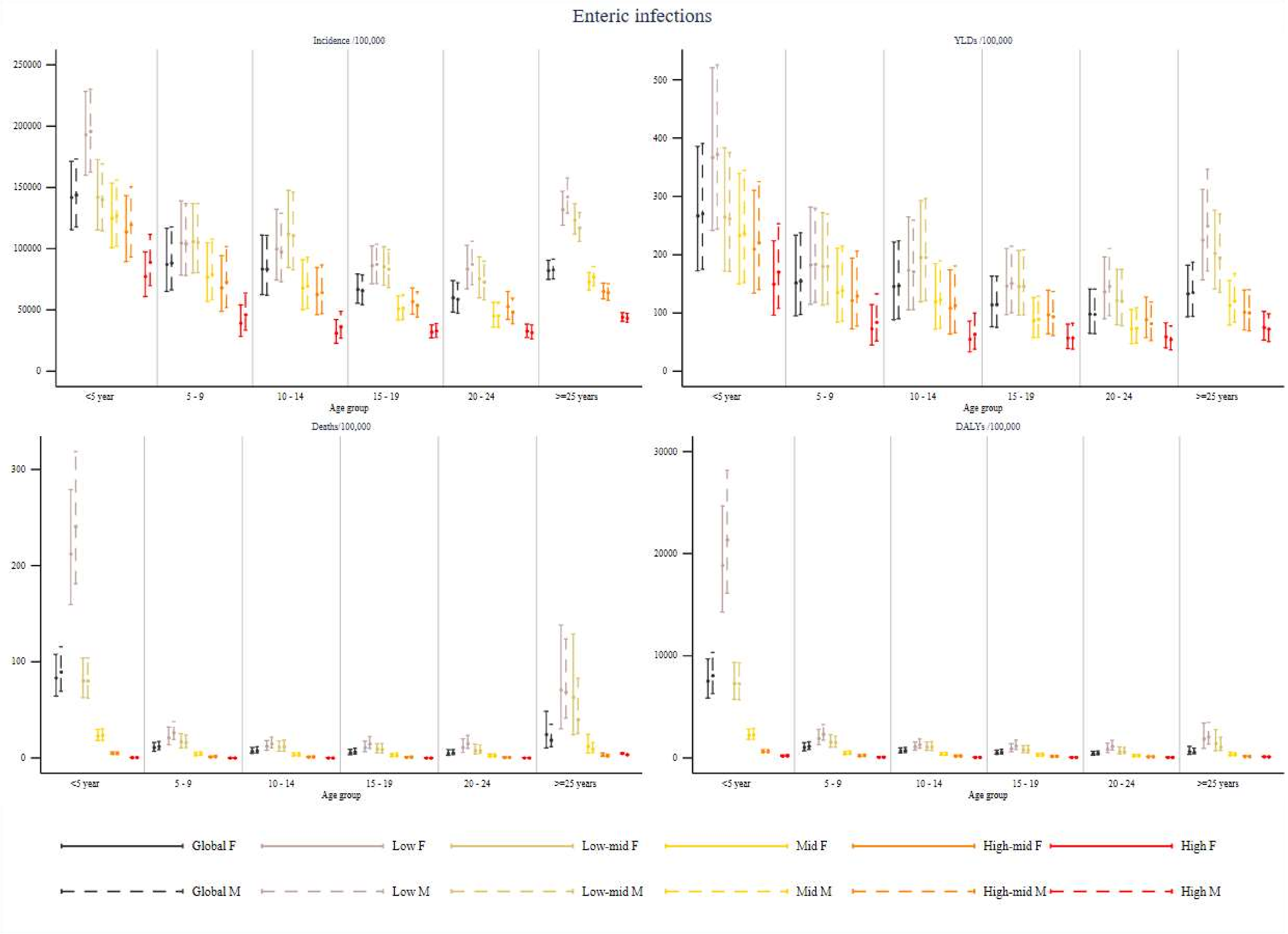

S17_1 Part B: Contribution of individual causes for death/100,000 and DALYs/ 100,00 for Enteric Infections in 2019

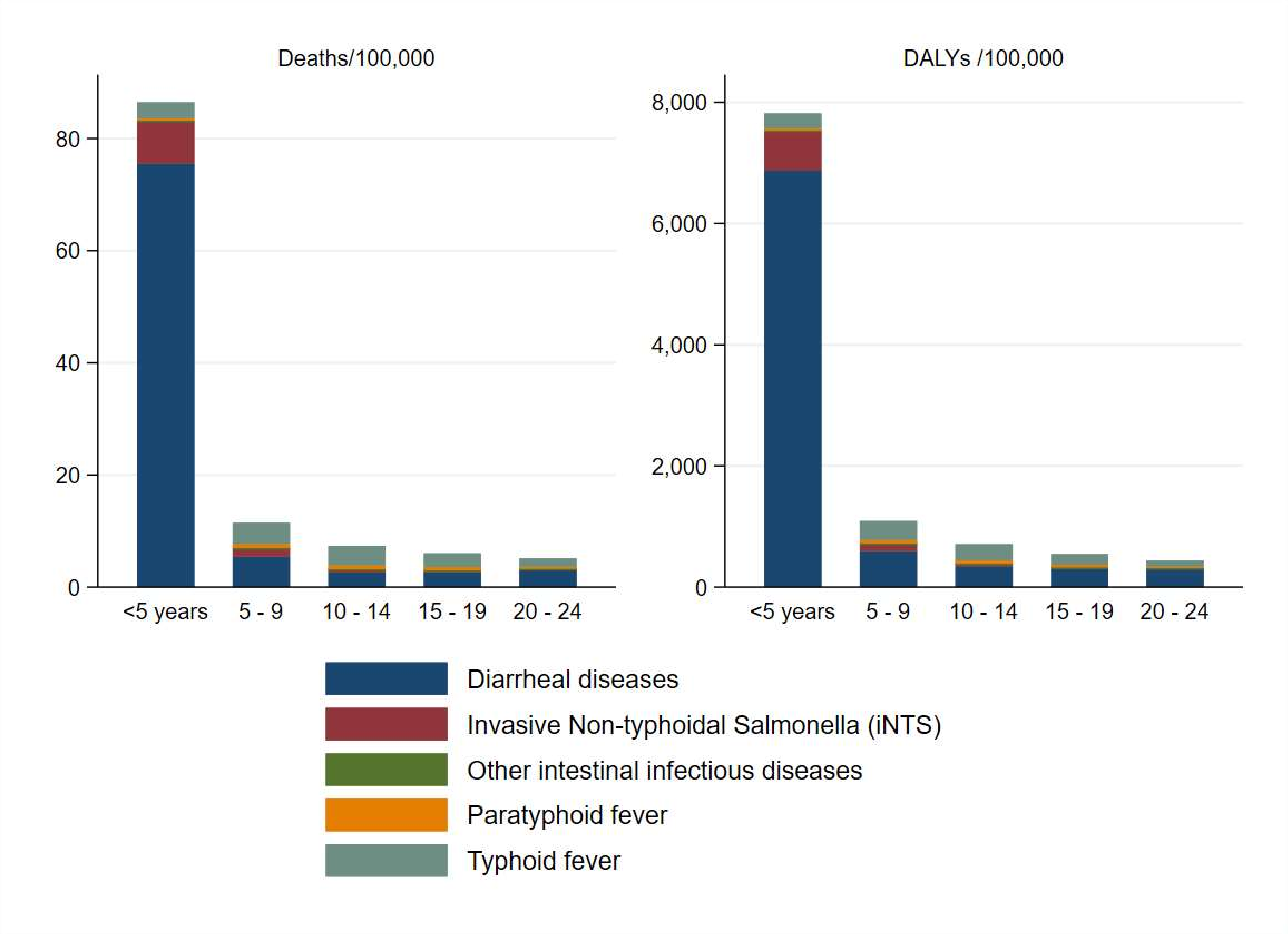

S17_2 Part A: Hepatitis Incidence, YLD, death and DALY per 100 000 in 2019

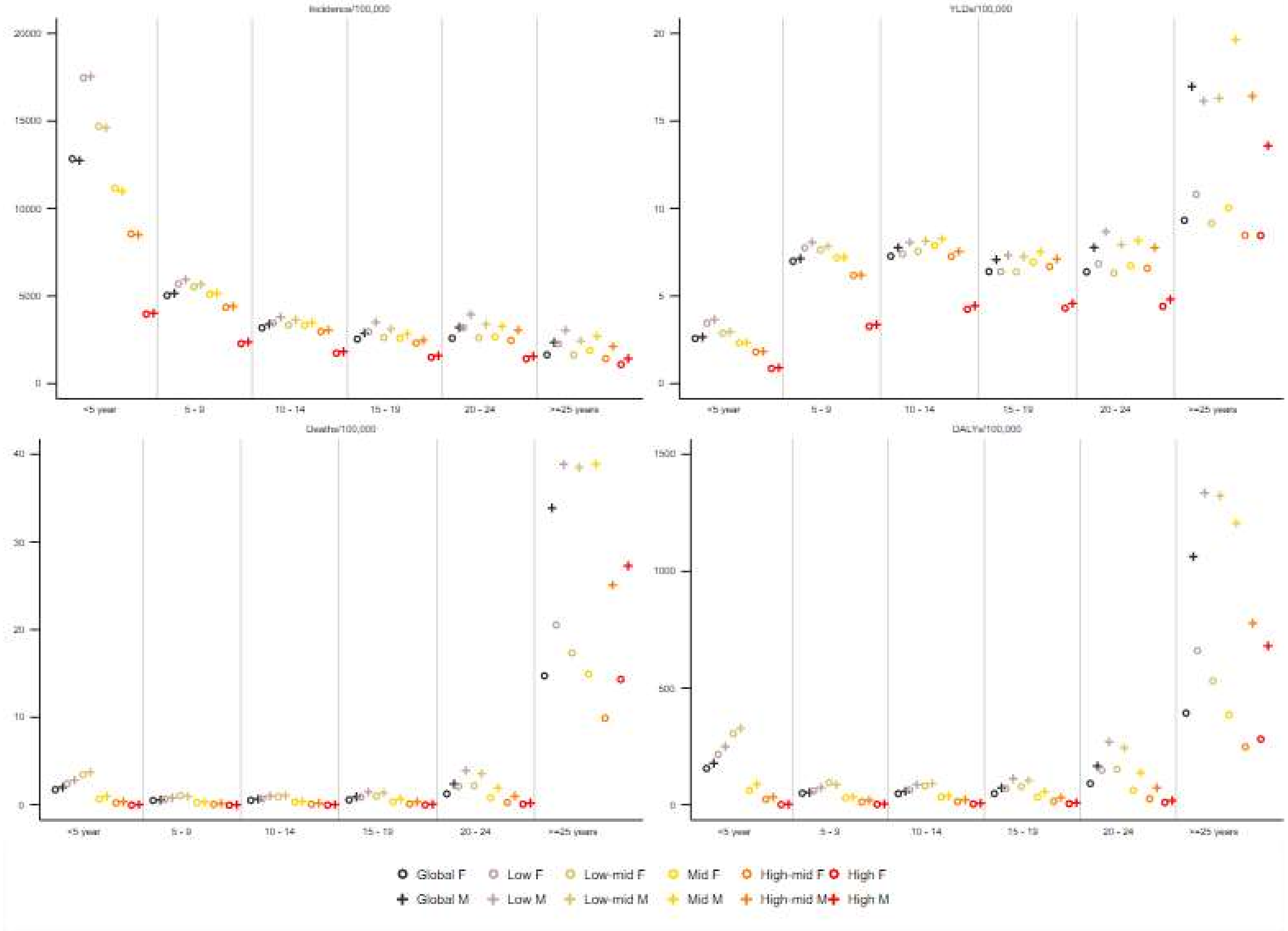

S17_2 Part B: Contribution of individual causes for death/100,000 and DALYs/ 100,00 for Hepatitis in 2019

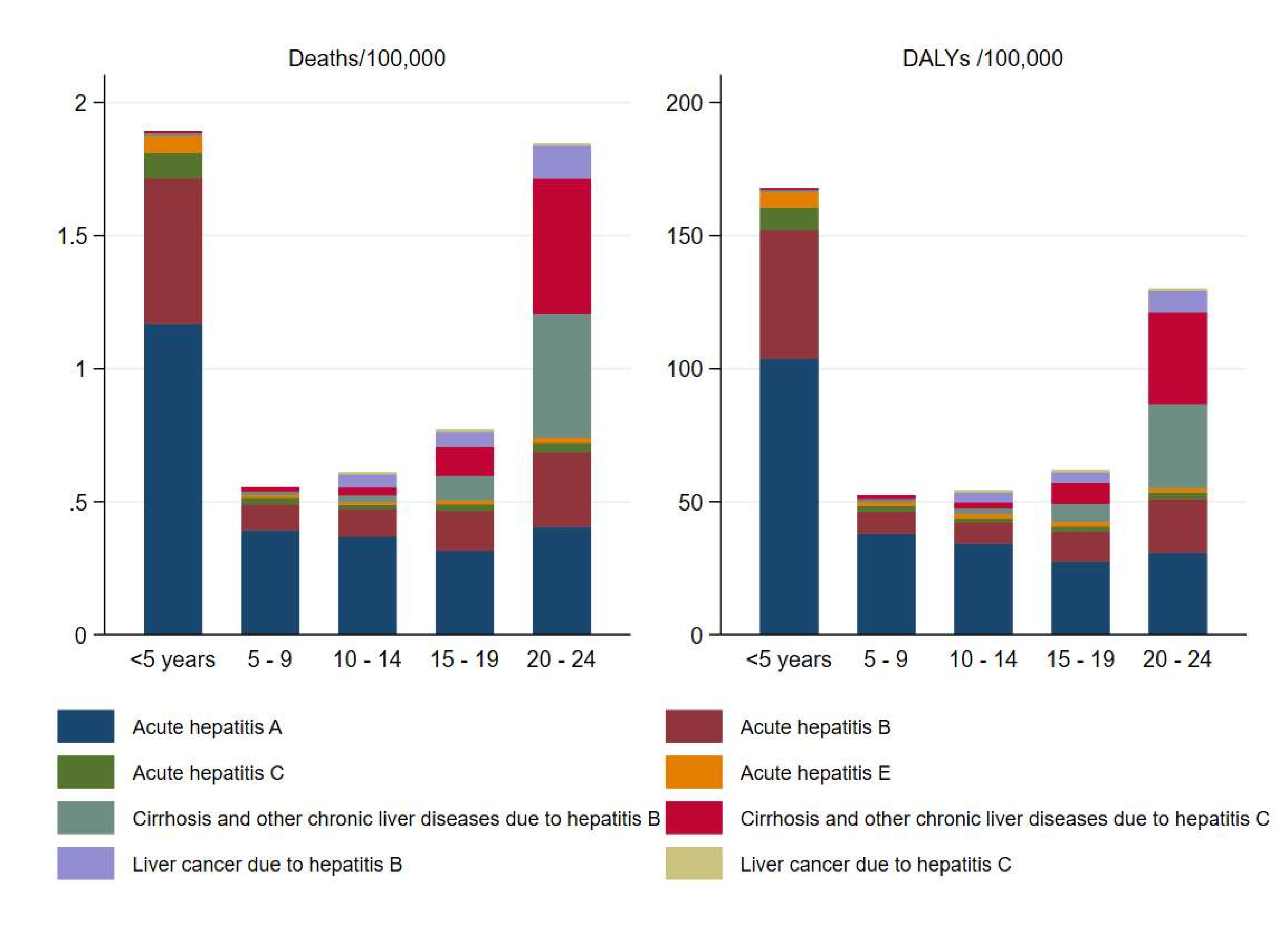

S17_3 Part A: HIV/AIDS Incidence, YLD, death and DALY per 100 000 in 2019 for each age group

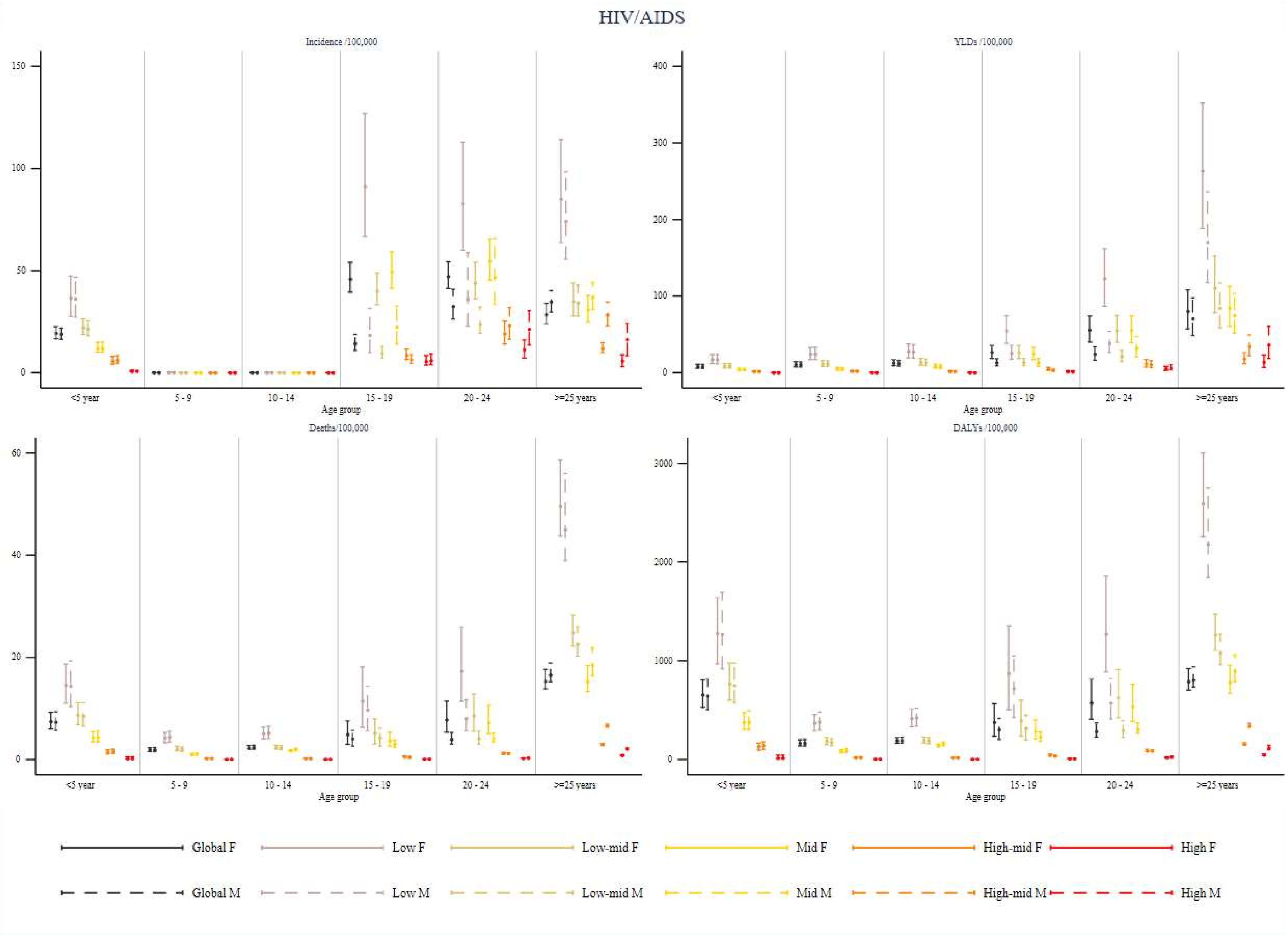

S17_3 Part B: Contribution of individual causes for death/100,000 and DALYs/ 100,00 for HIV/AIDS in 2019

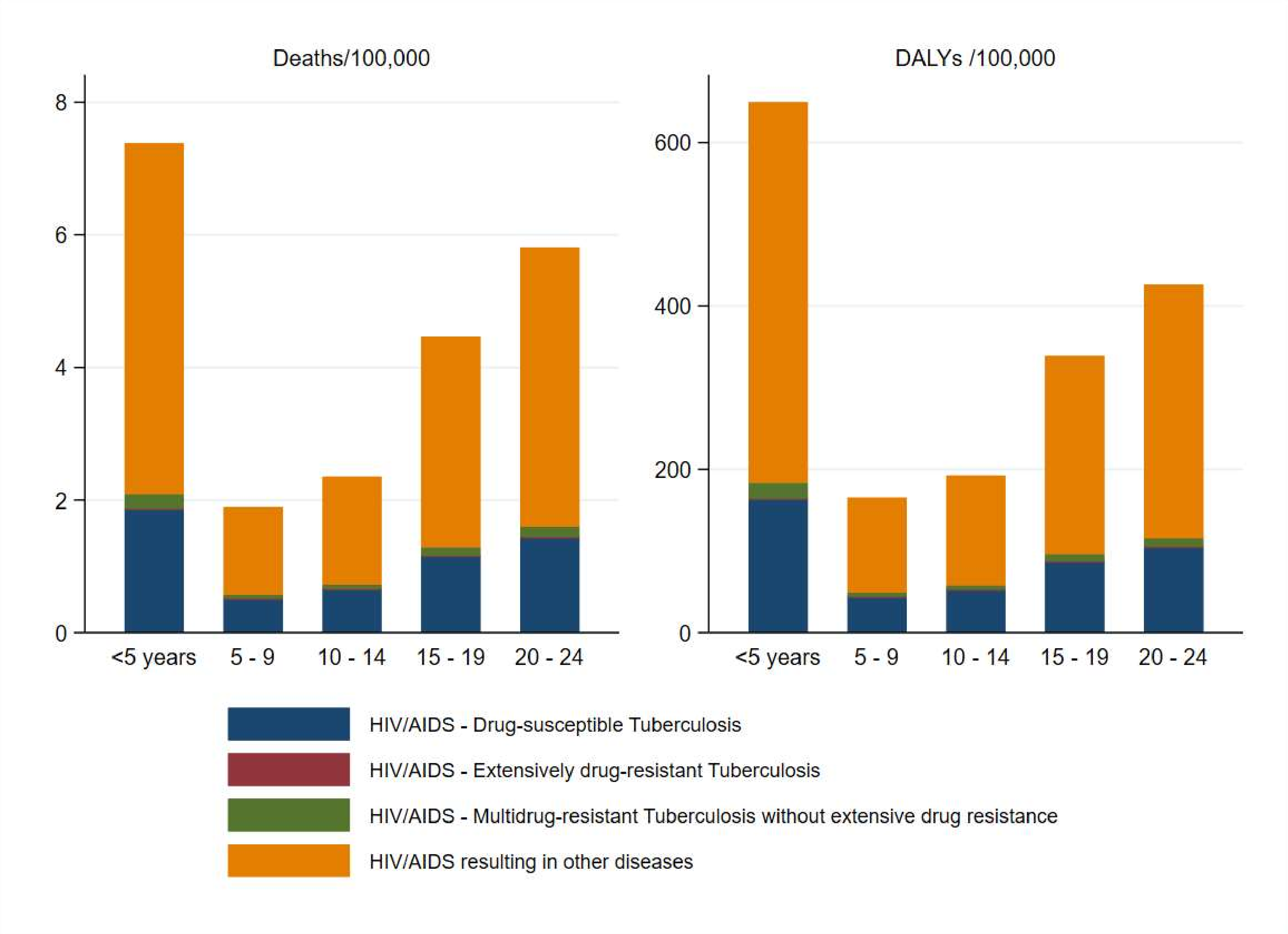

S17_4 Part A: Infectious skin conditions Incidence, YLD, death and DALY per 100 000 in 2019 for each age group

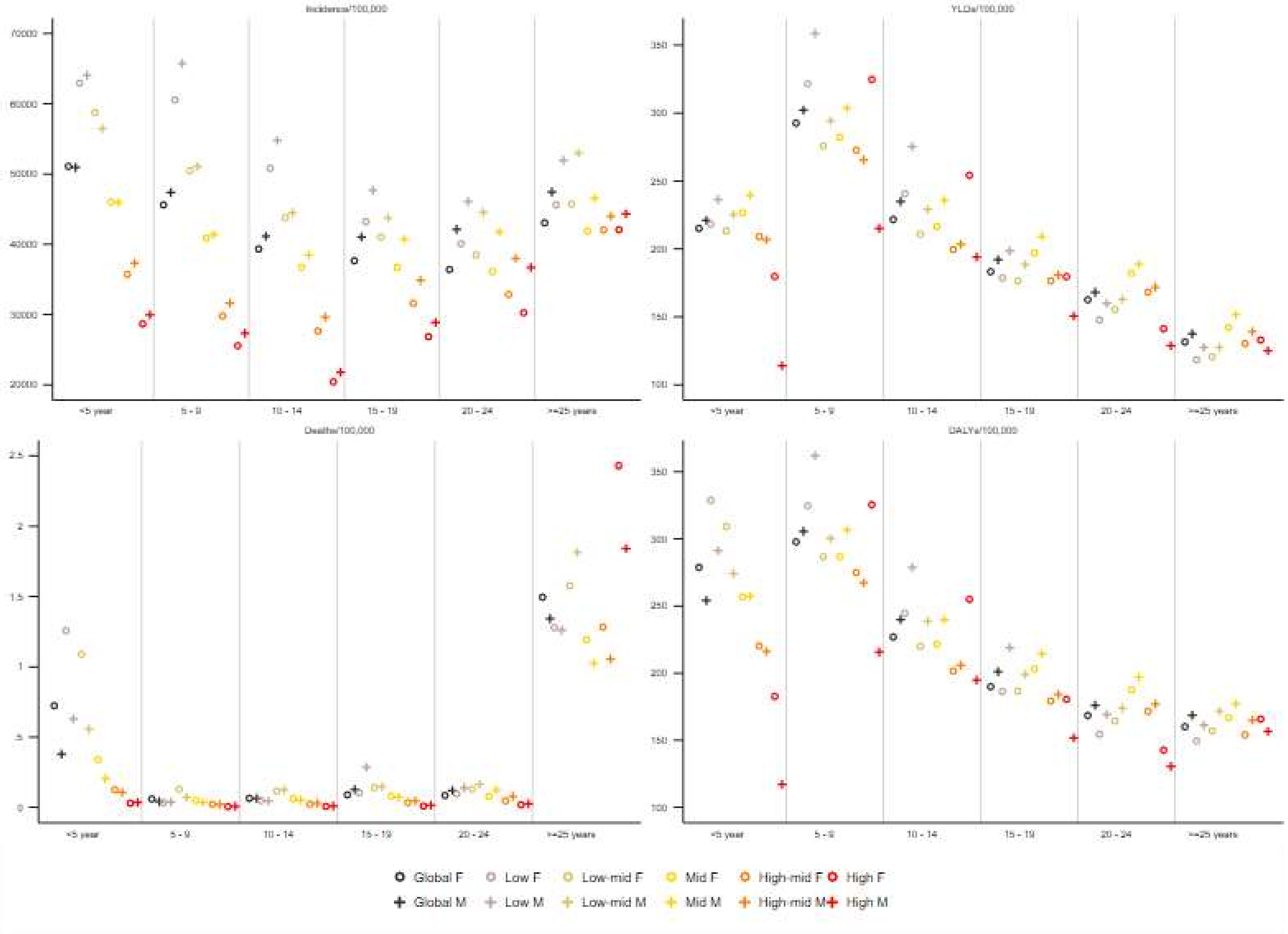

S17_4 Part B: Contribution of individual causes for death/100,000 and DALYs/ 100,00 for Infectious skin conditions in 2019

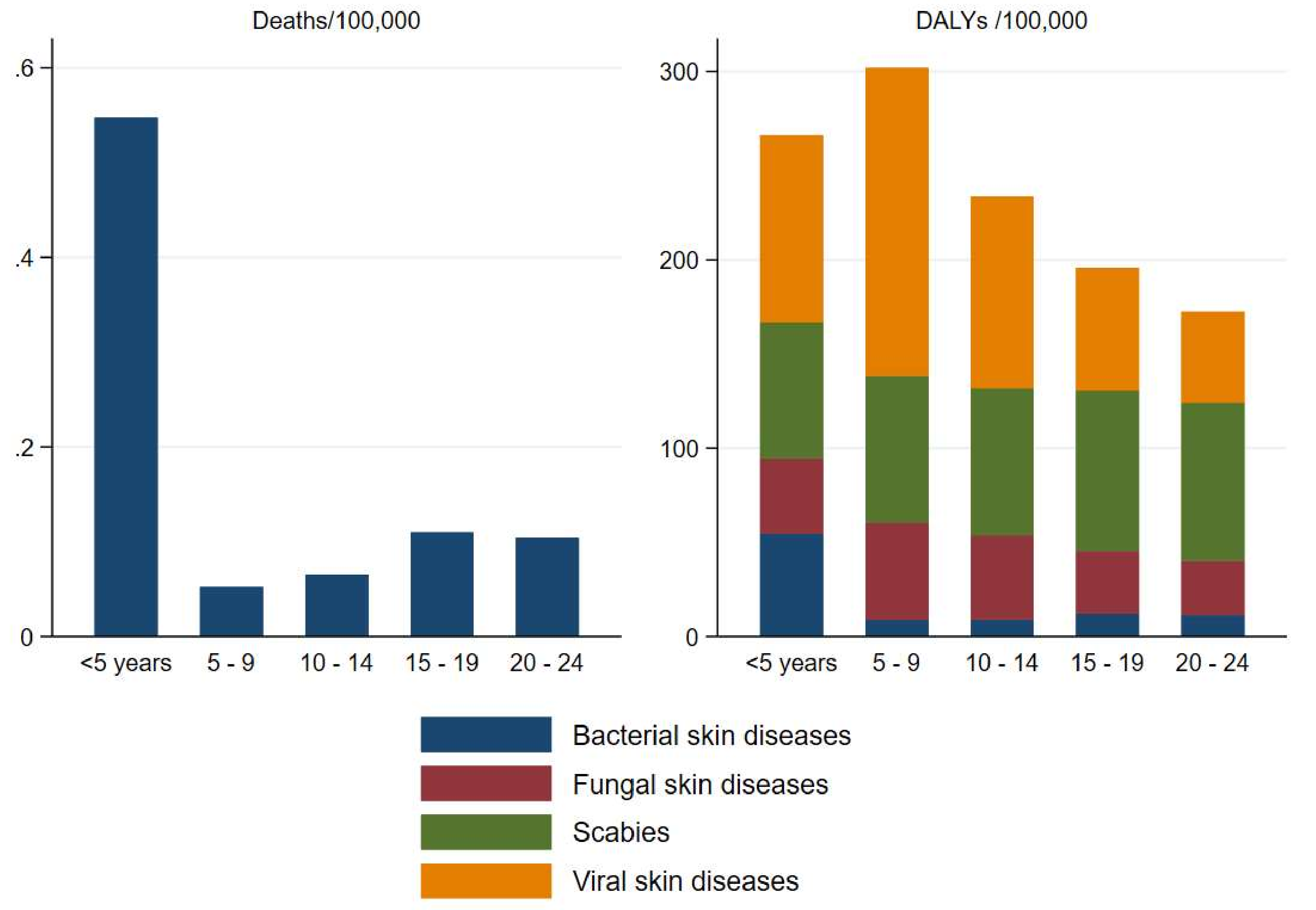

S17_5 Part A: Lower respiratory infections Incidence, YLD, death and DALY per 100 000 in 2019 for each age group

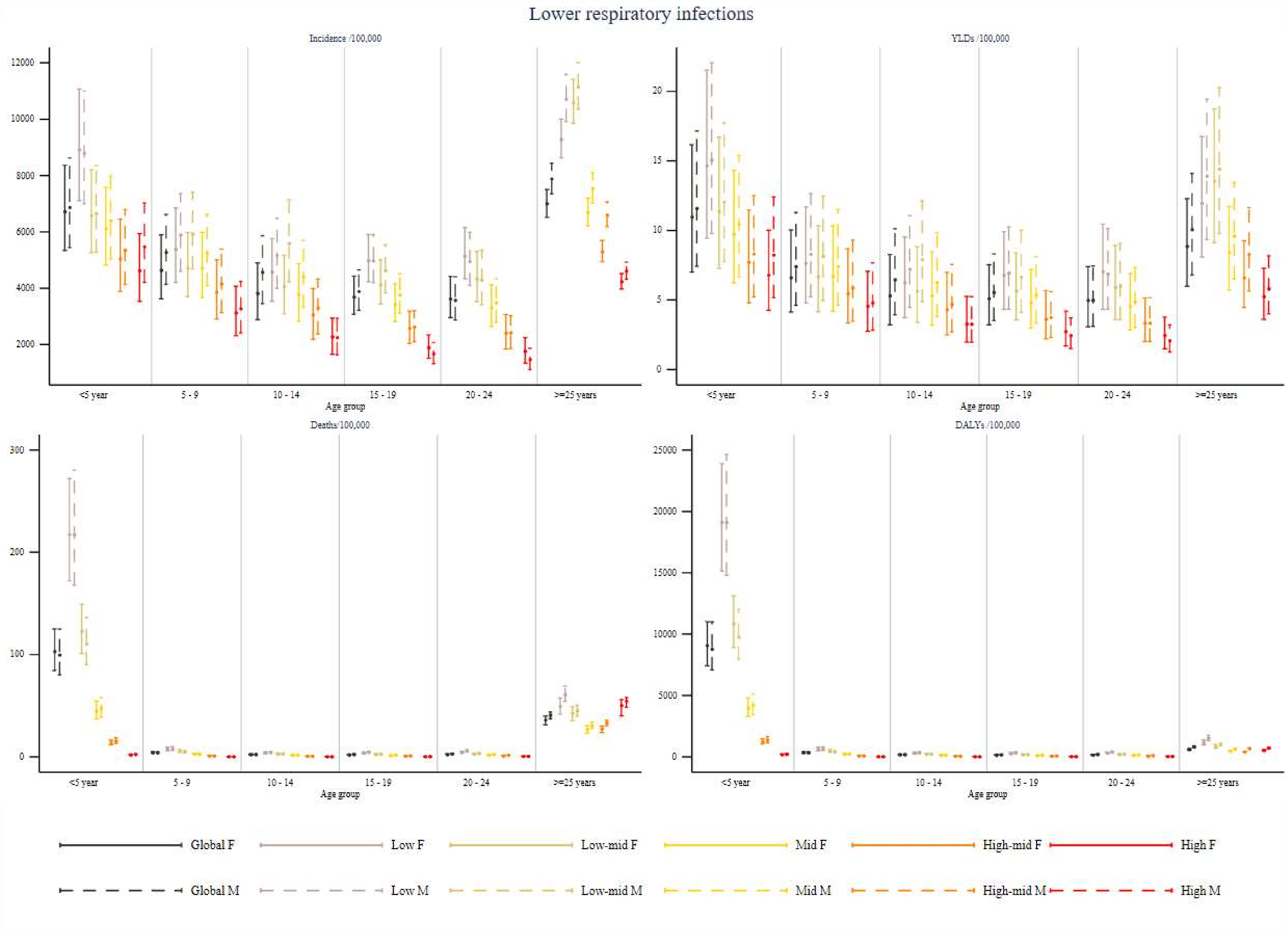

S17_6 Part A: Malaria Incidence, YLD, death and DALY per 100 000 in 2019 for each age group

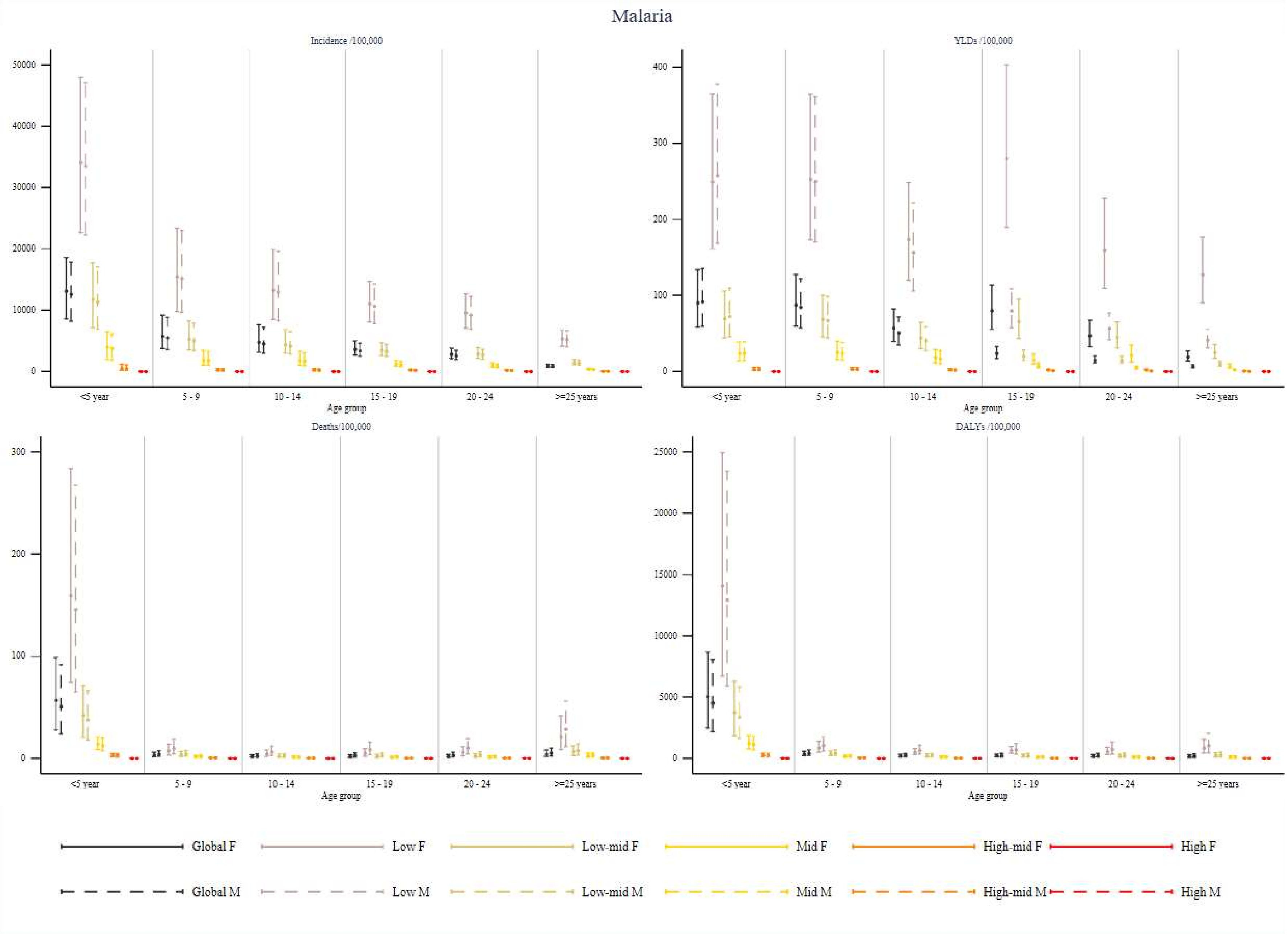

S17_7 Part A: Maternal sepsis and other maternal infections Incidence, YLD, death and DALY per 100 000 in 2019 for each age group

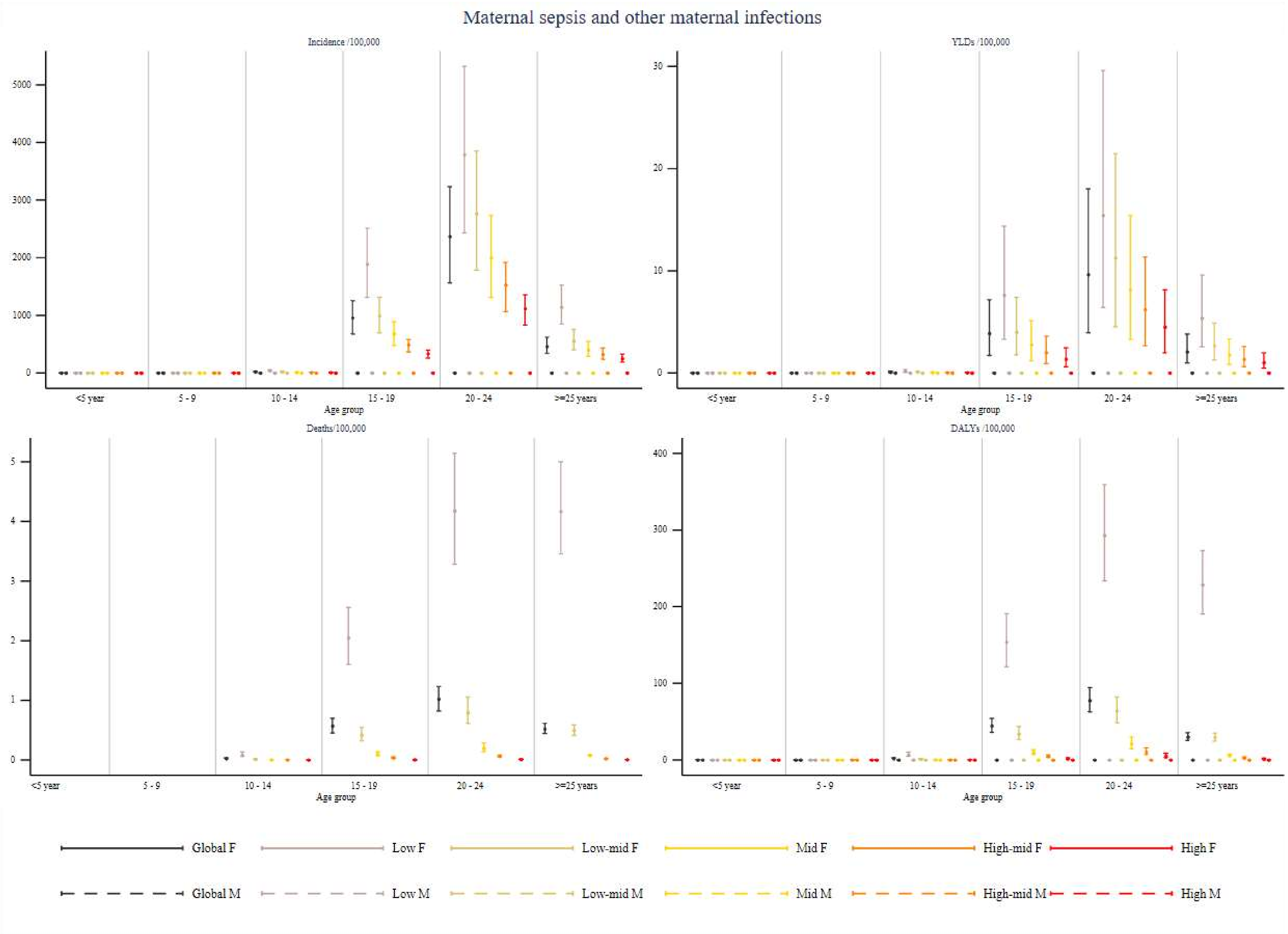

S17_8 Part A: Meningitis & Encephalitis Incidence, YLD, death and DALY per 100 000 in 2019 for each age group

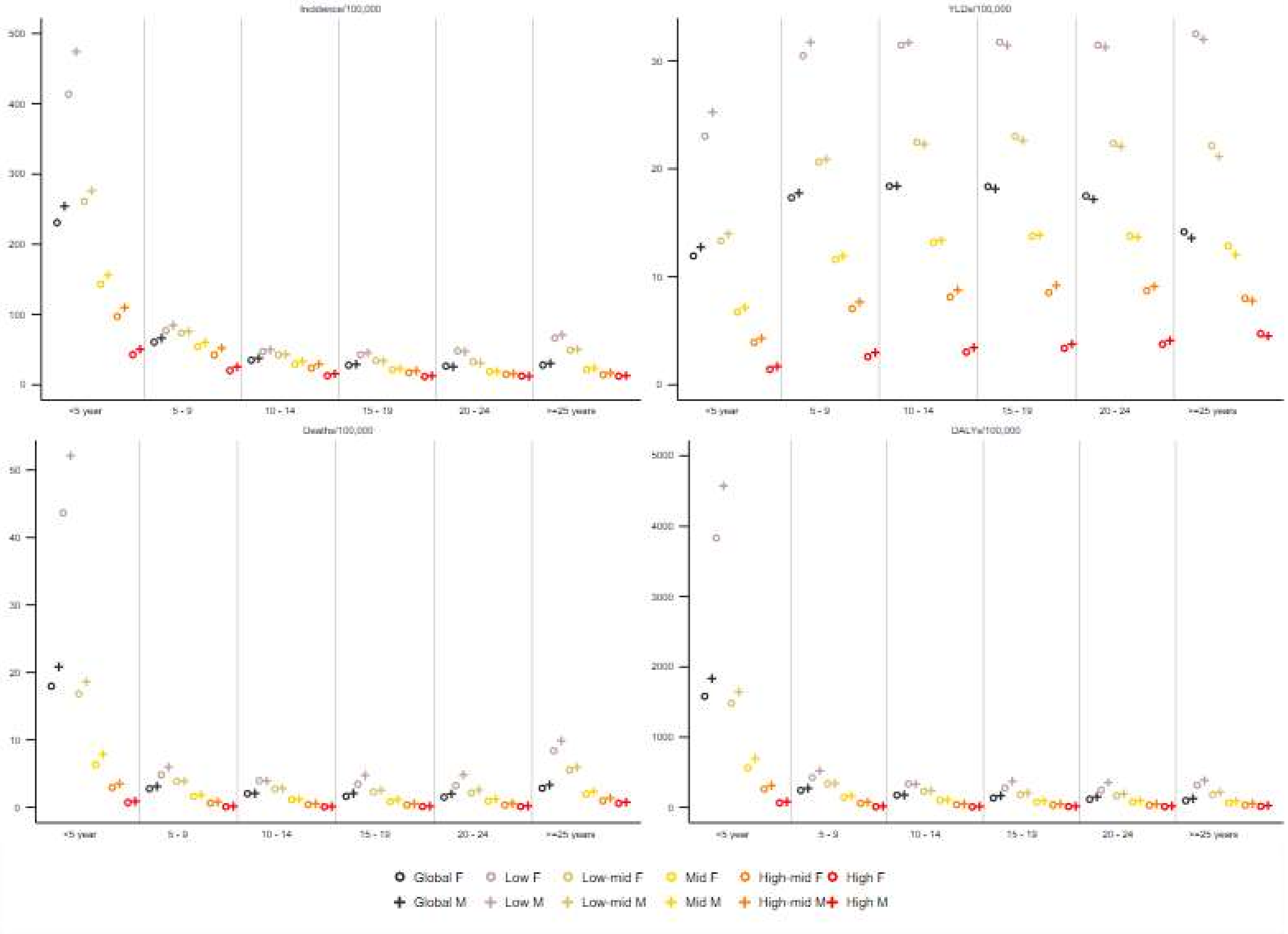

S17_8 Part B: Contribution of individual causes for death/100,000 and DALYs/ 100,00 for Meningitis & Encephalitis in 2019

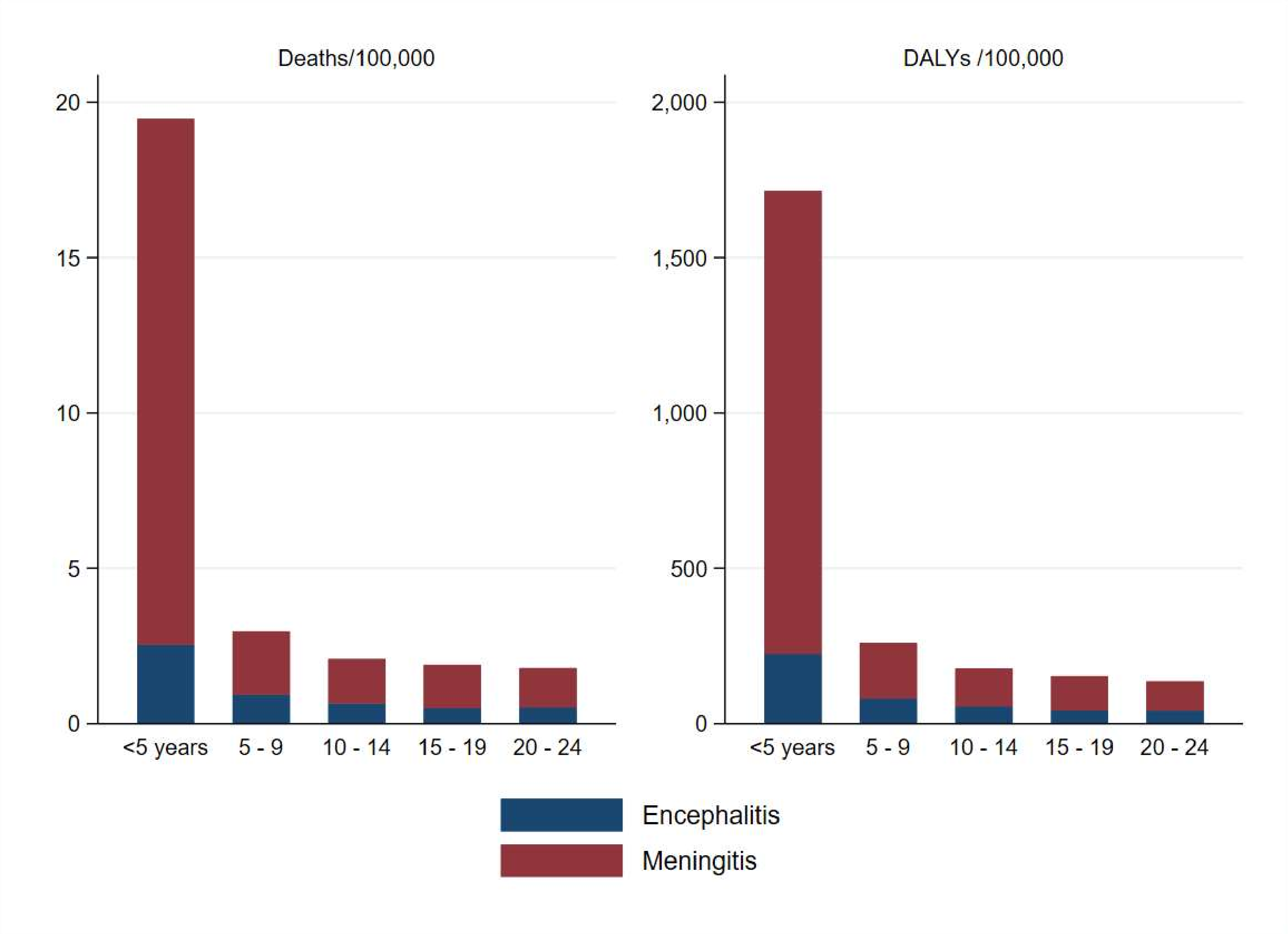

S17_9 Part A: Neglected Tropical Diseases Incidence, YLD, death and DALY per 100 000 in 2019 for each age group

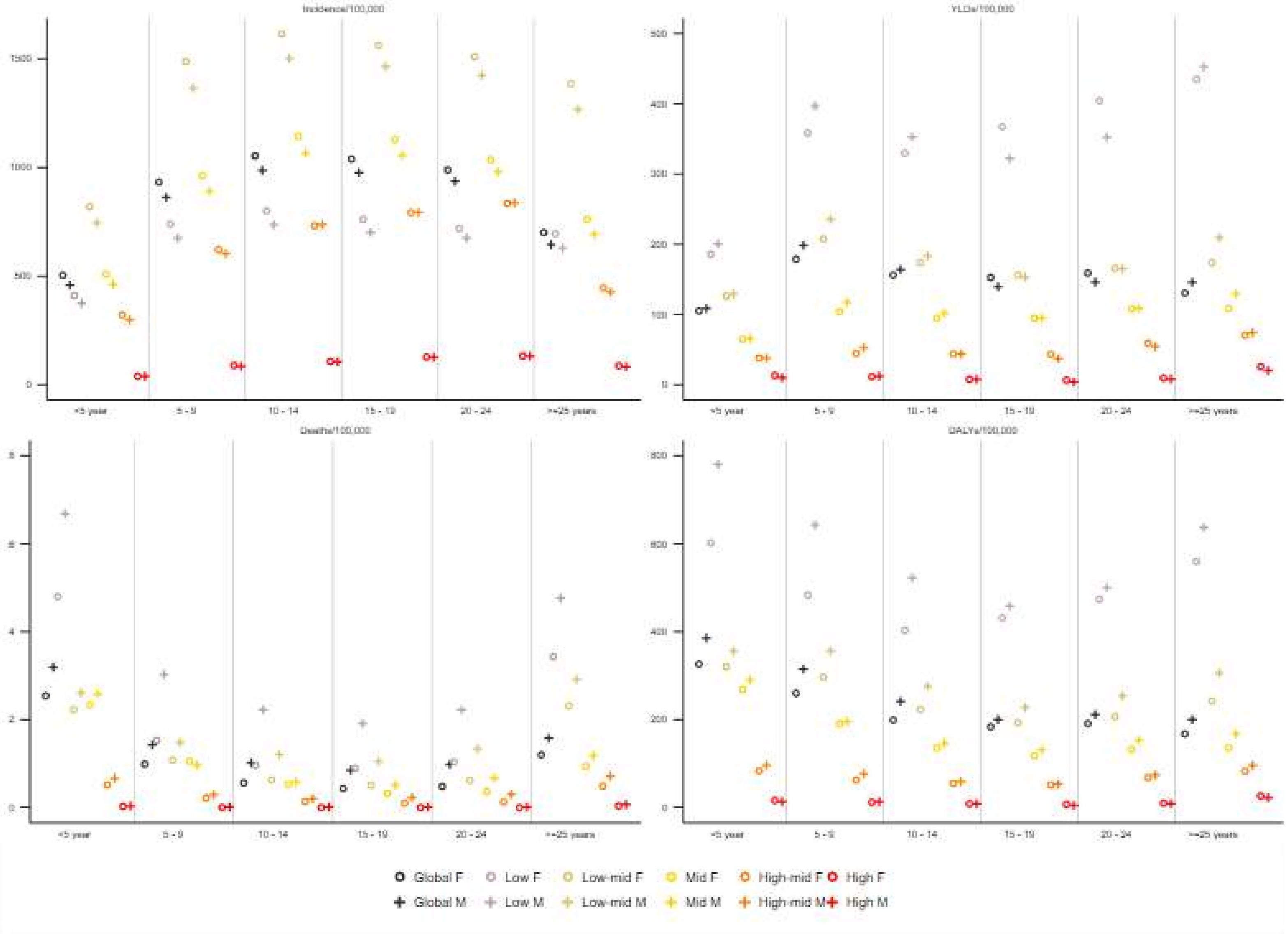

S17_9 Part B: Contribution of individual causes for death/100,000 and DALYs/ 100,00 for Neglected Tropical Diseases in 2019

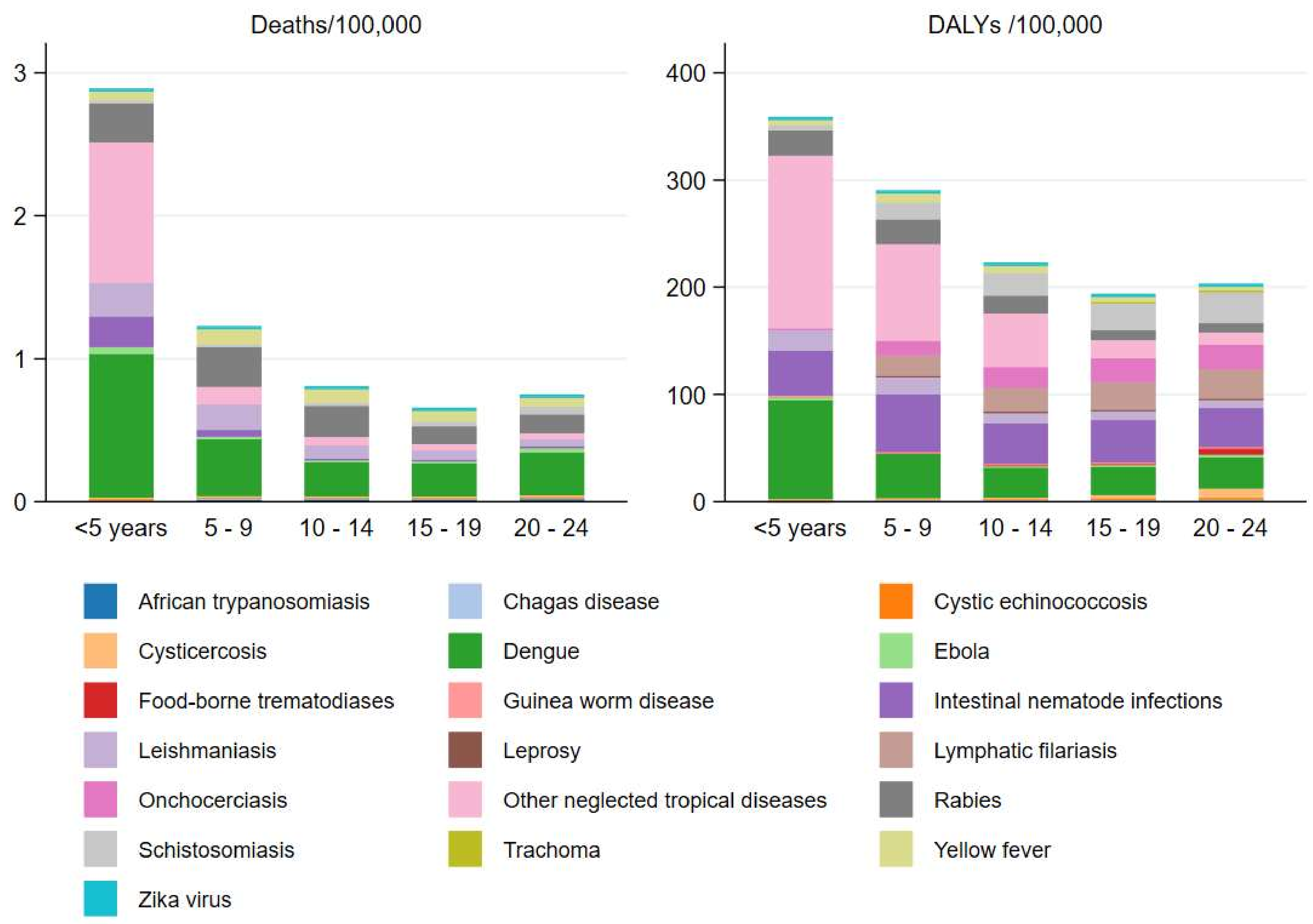

S17_10 Part A: Neonatal sepsis and other neonatal infections Incidence, YLD, death and DALY per 100 000 in 2019 for each age group

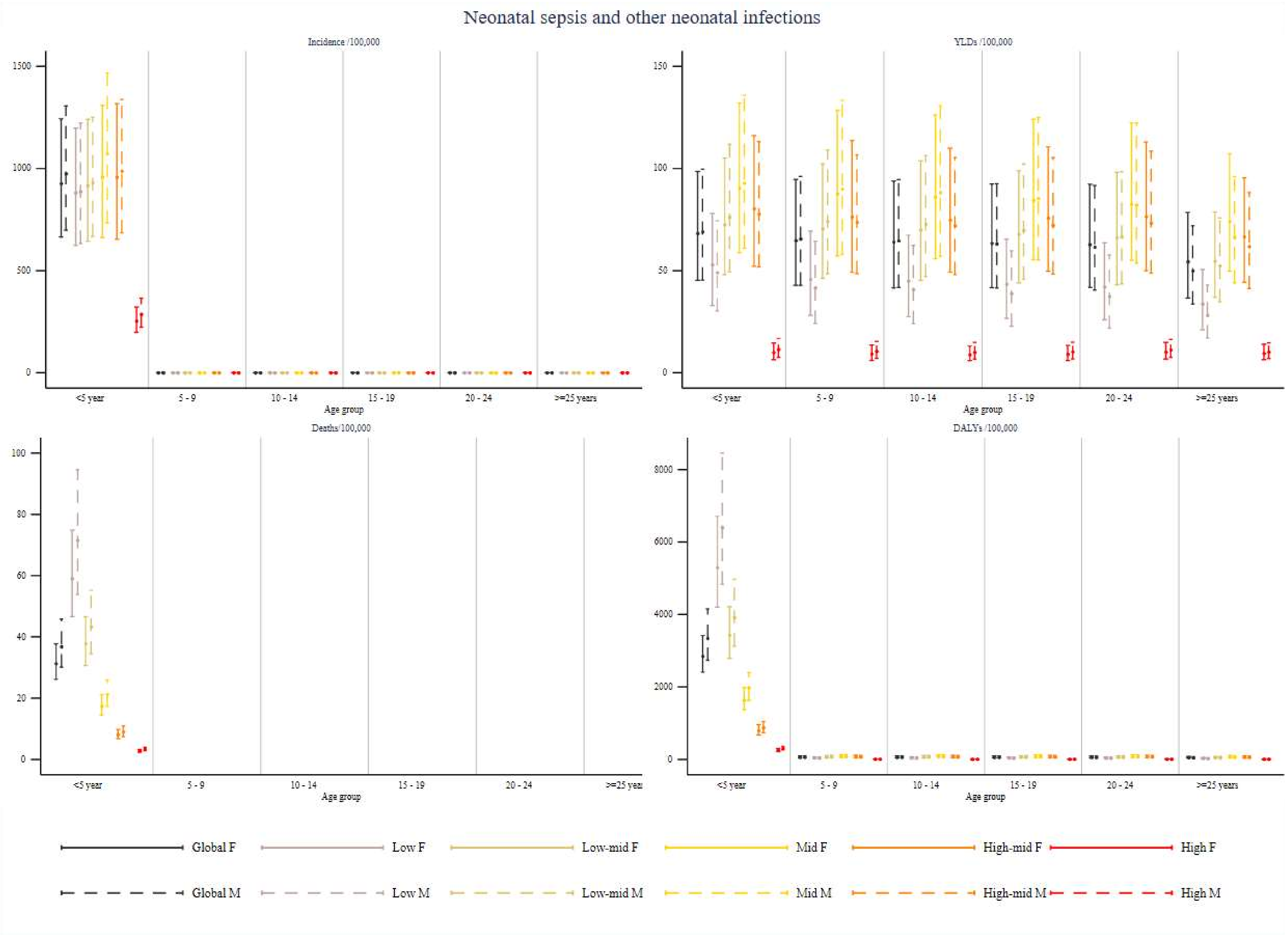

S17_11 Part A: Other unspecified infectious diseases Incidence, YLD, death and DALY per 100 000 in 2019 for each age group

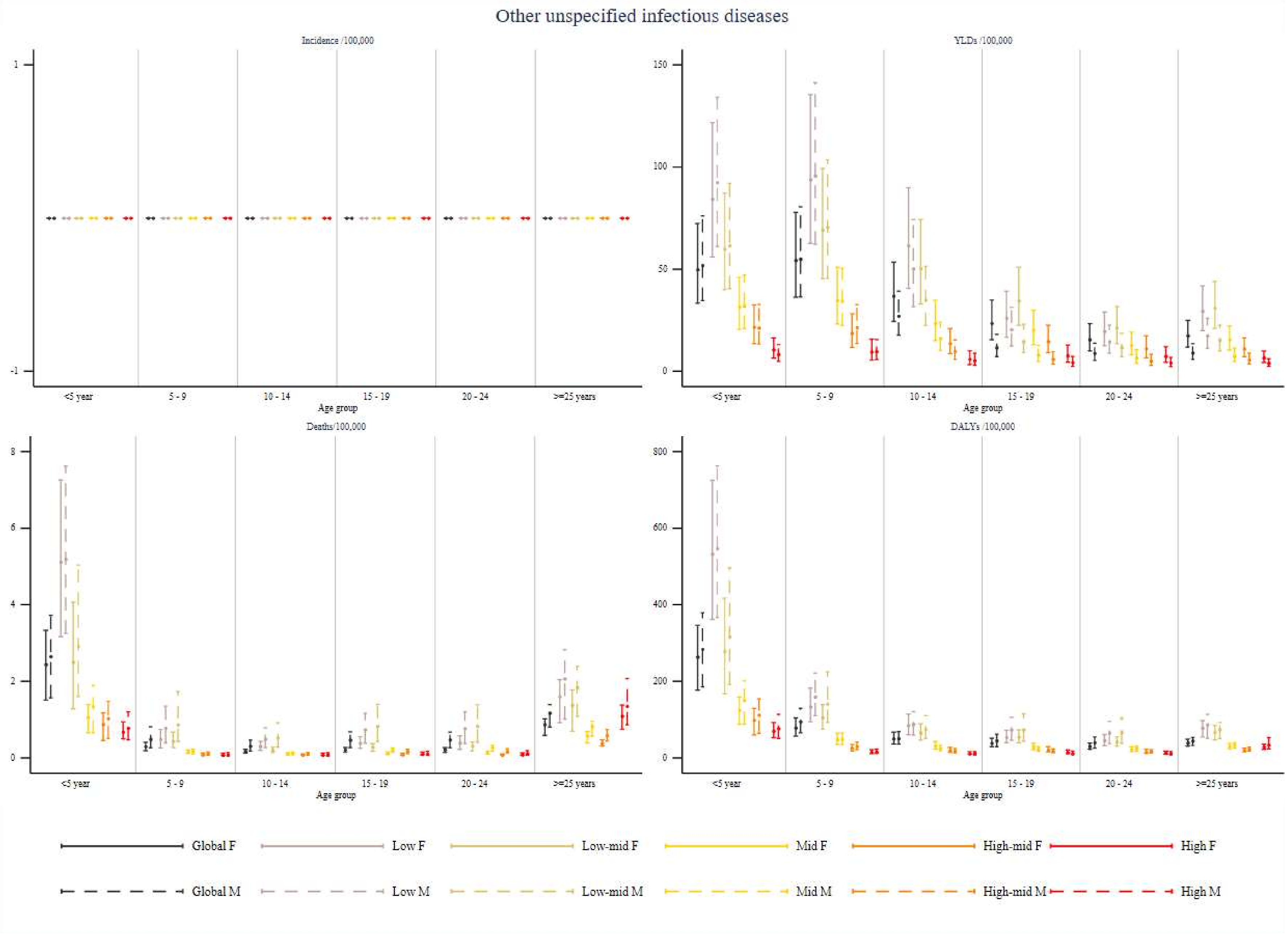

S17_12 Part A: Rheumatic heart disease Incidence, YLD, death and DALY per 100 000 in 2019 for each age group

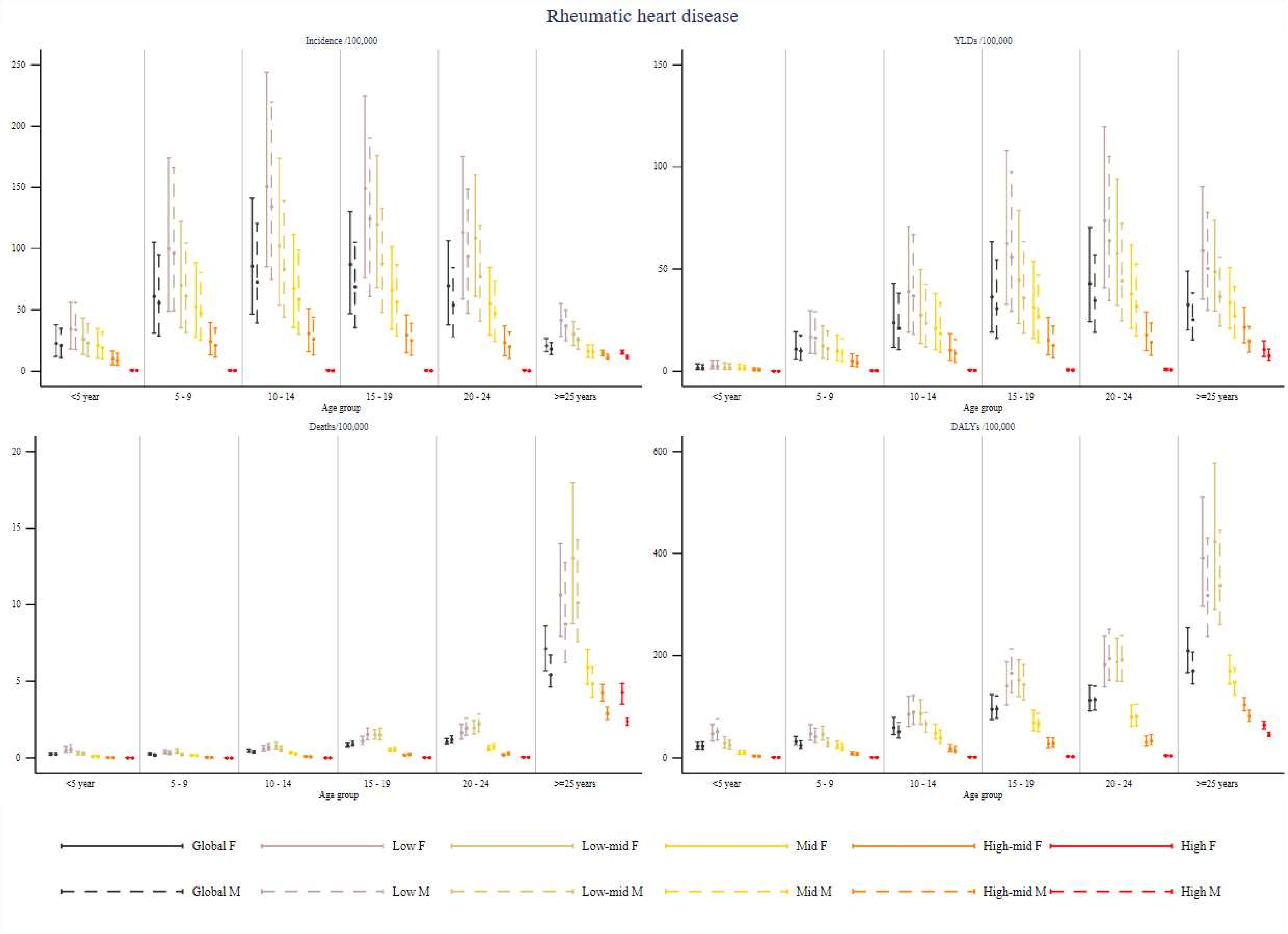

S17_13 Part A: Sexually transmitted infections excluding HIV Incidence, YLD, death and DALY per 100 000 in 2019 for each age group

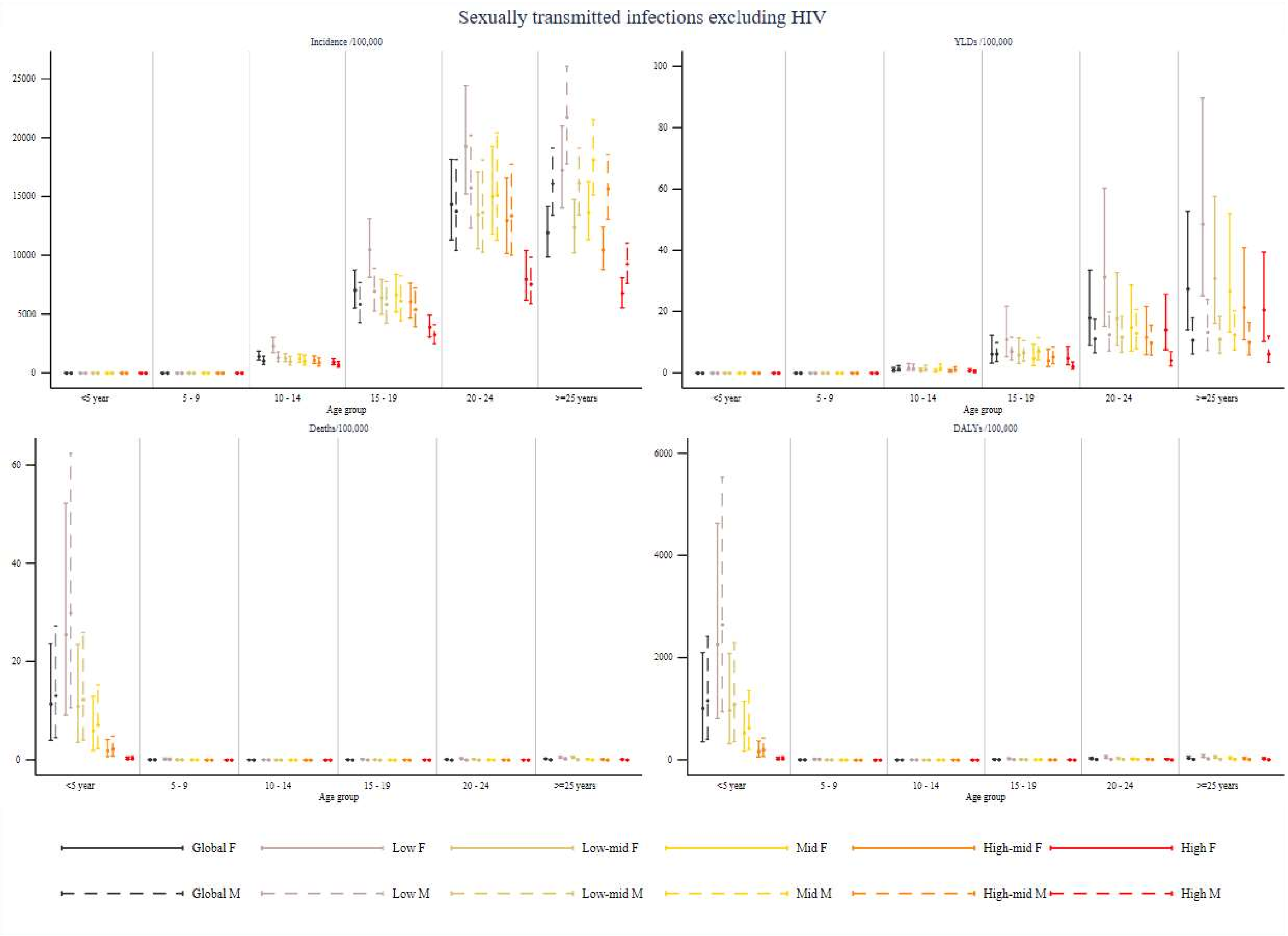

S17_13 Part B: Contribution of individual causes for death/100,000 and DALYs/ 100,00 for Sexually transmitted infections excluding HIV in 2019

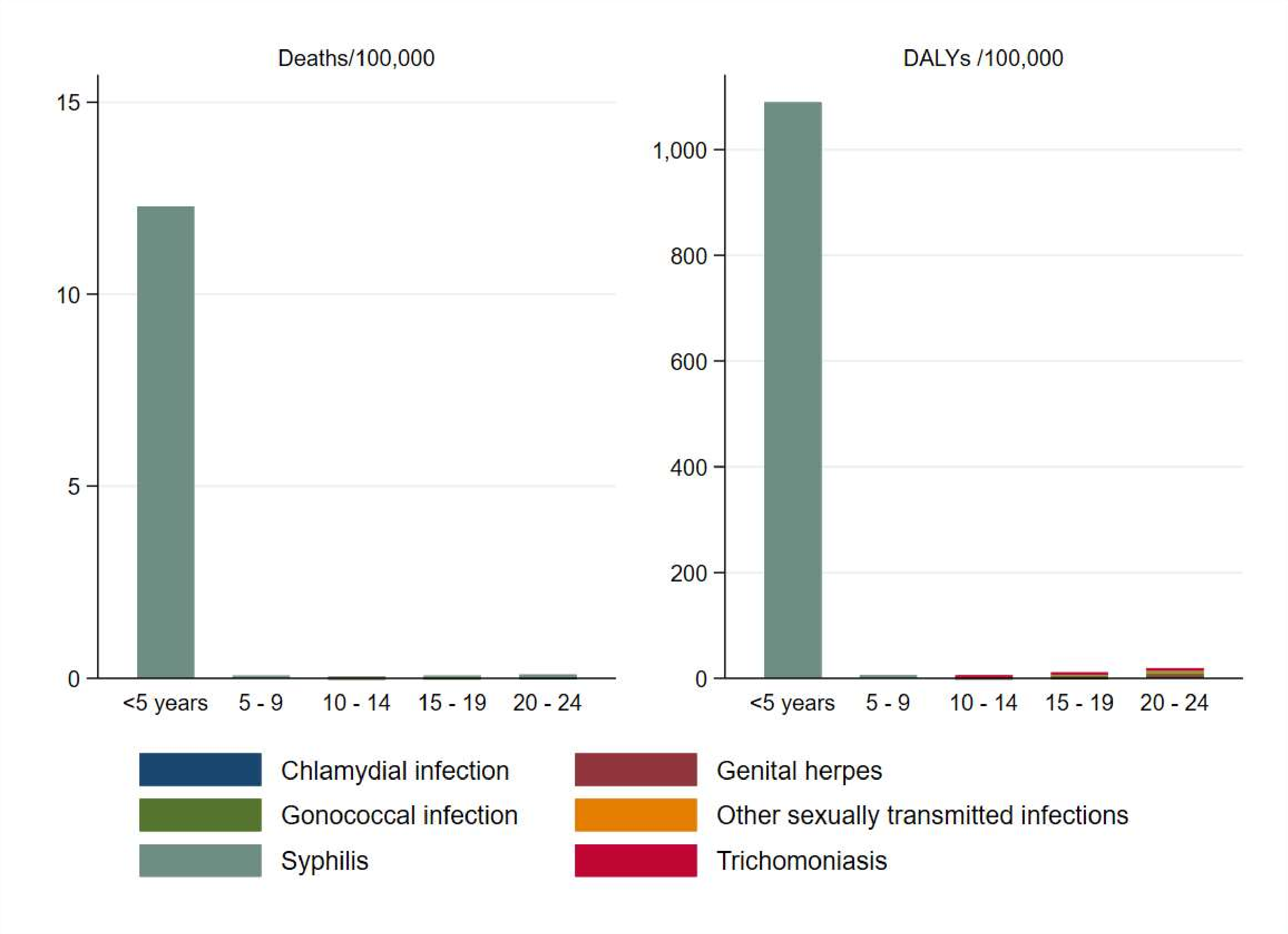

S17_14 Part A: Tuberculosis Incidence, YLD, death and DALY per 100 000 in 2019 for each age group

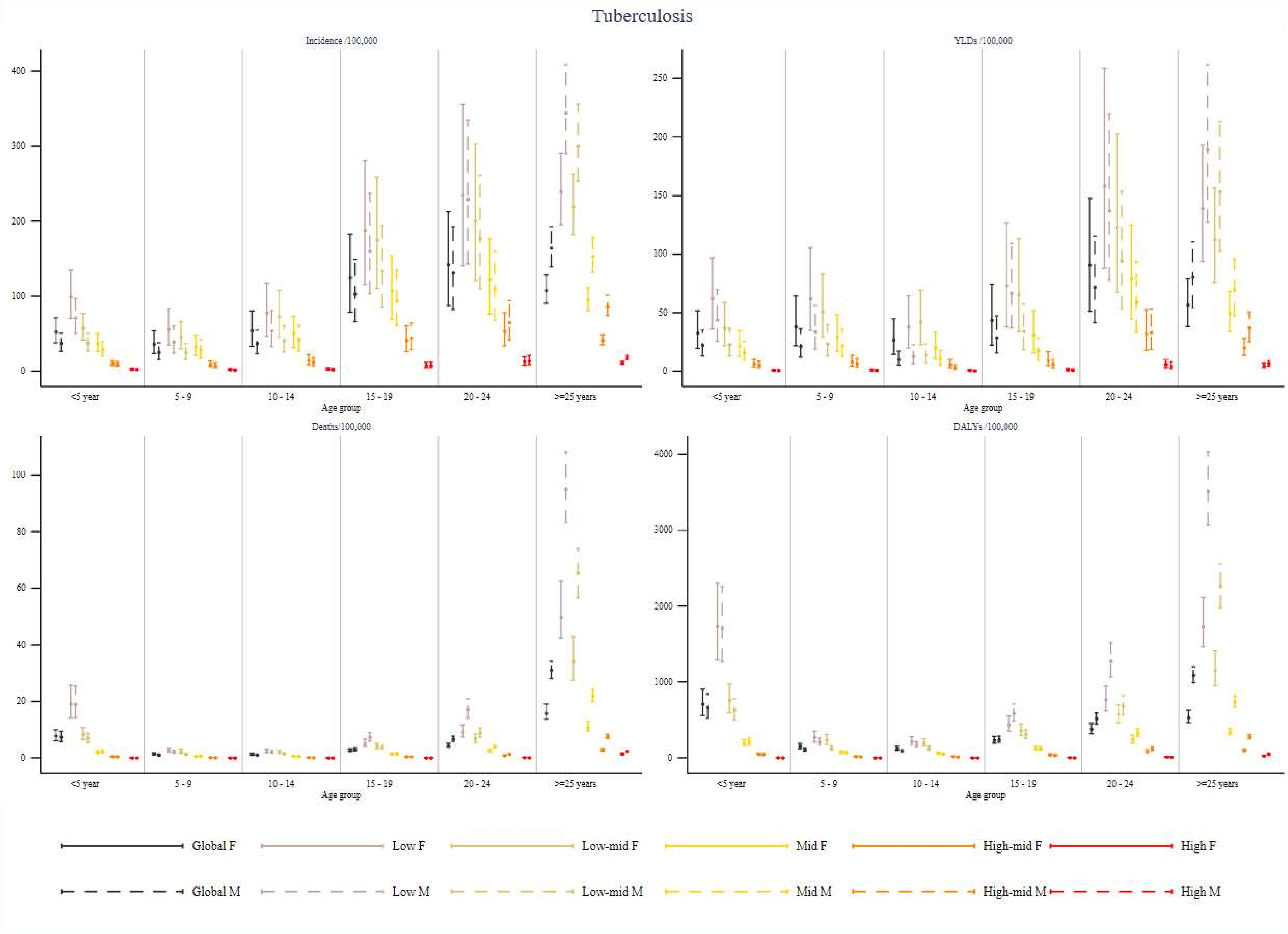

S17_14 Part B: Contribution of individual causes for death/100,000 and DALYs/ 100,00 for Tuberculosis in 2019

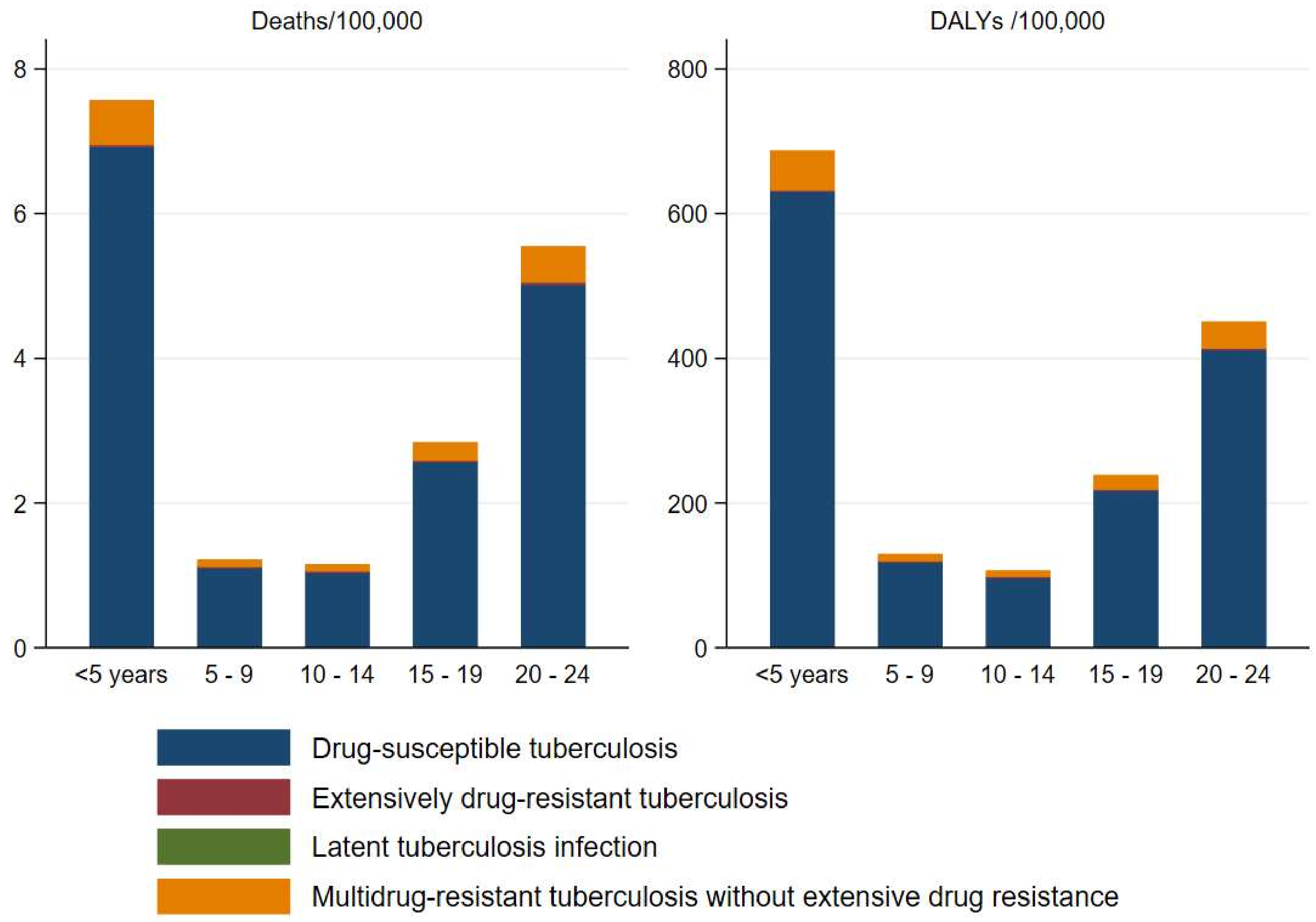

S17_15 Part A: Upper respiratory infections Incidence, YLD, death and DALY per 100 000 in 2019 for each age group

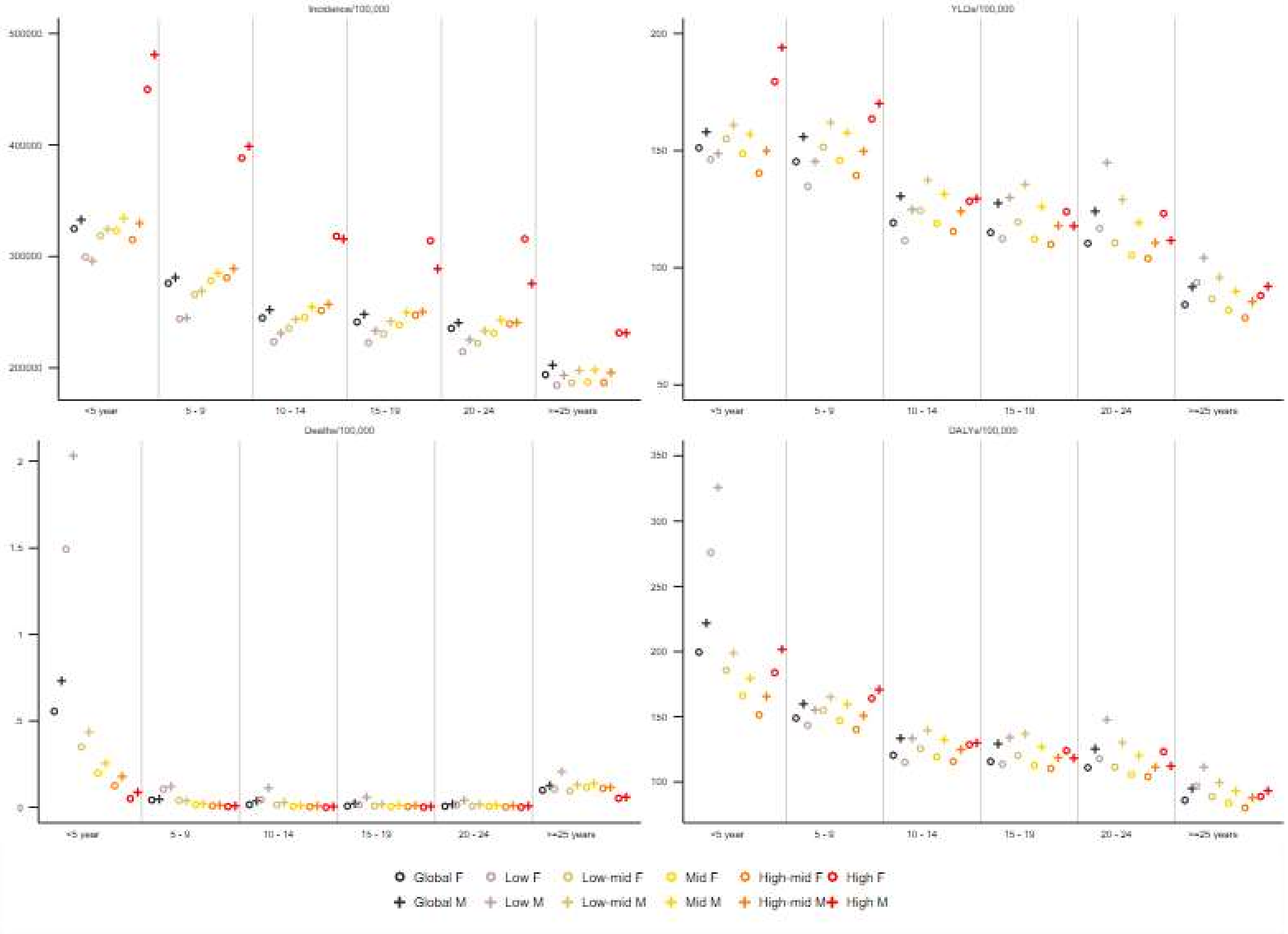

S17_15 Part B: Contribution of individual causes for death/100,000 and DALYs/ 100,00 for Upper respiratory infections in 2019

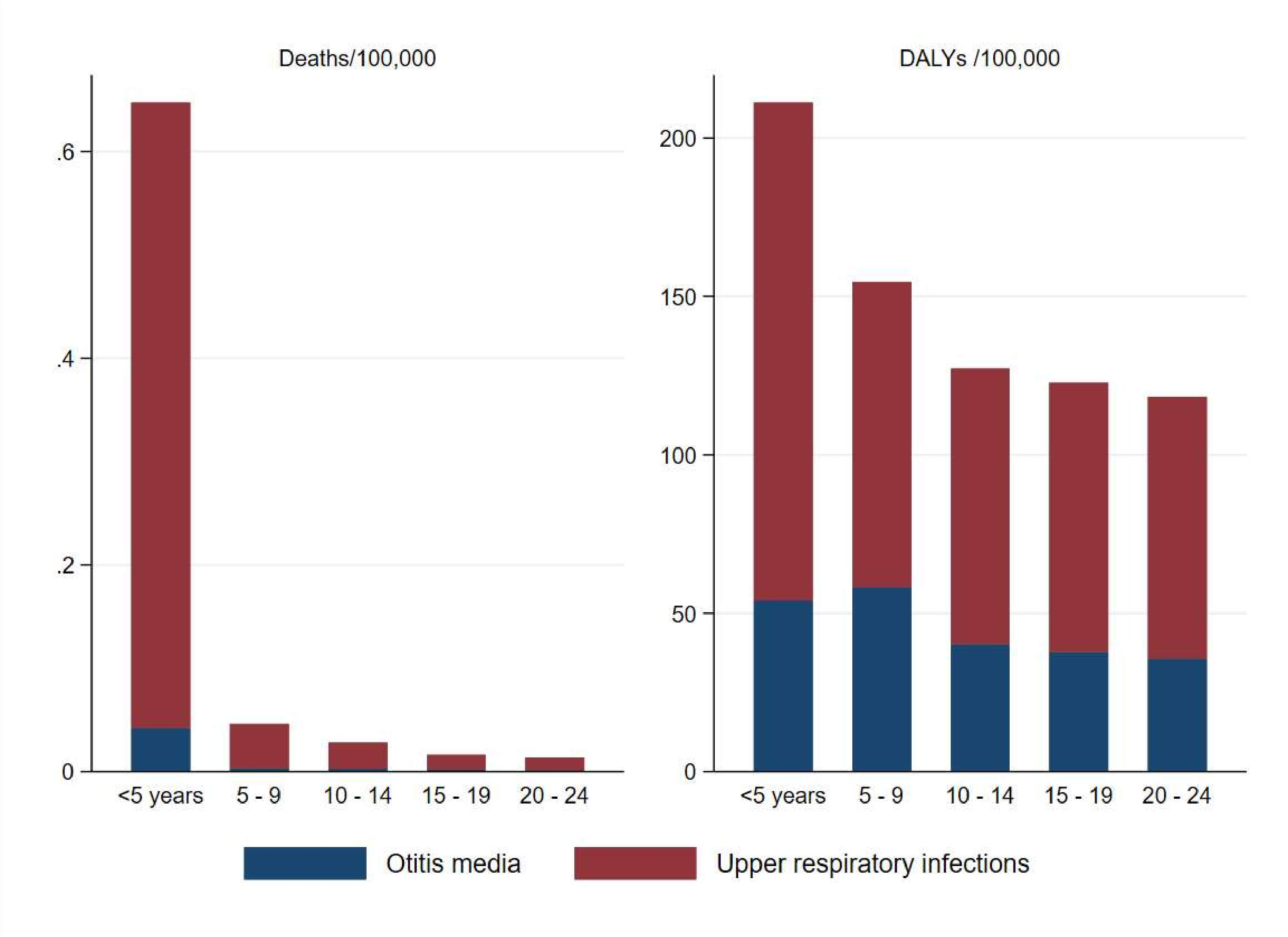

S17_16 Part A: Vaccine preventable diseases Incidence, YLD, death and DALY per 100 000 in 2019 for each age group

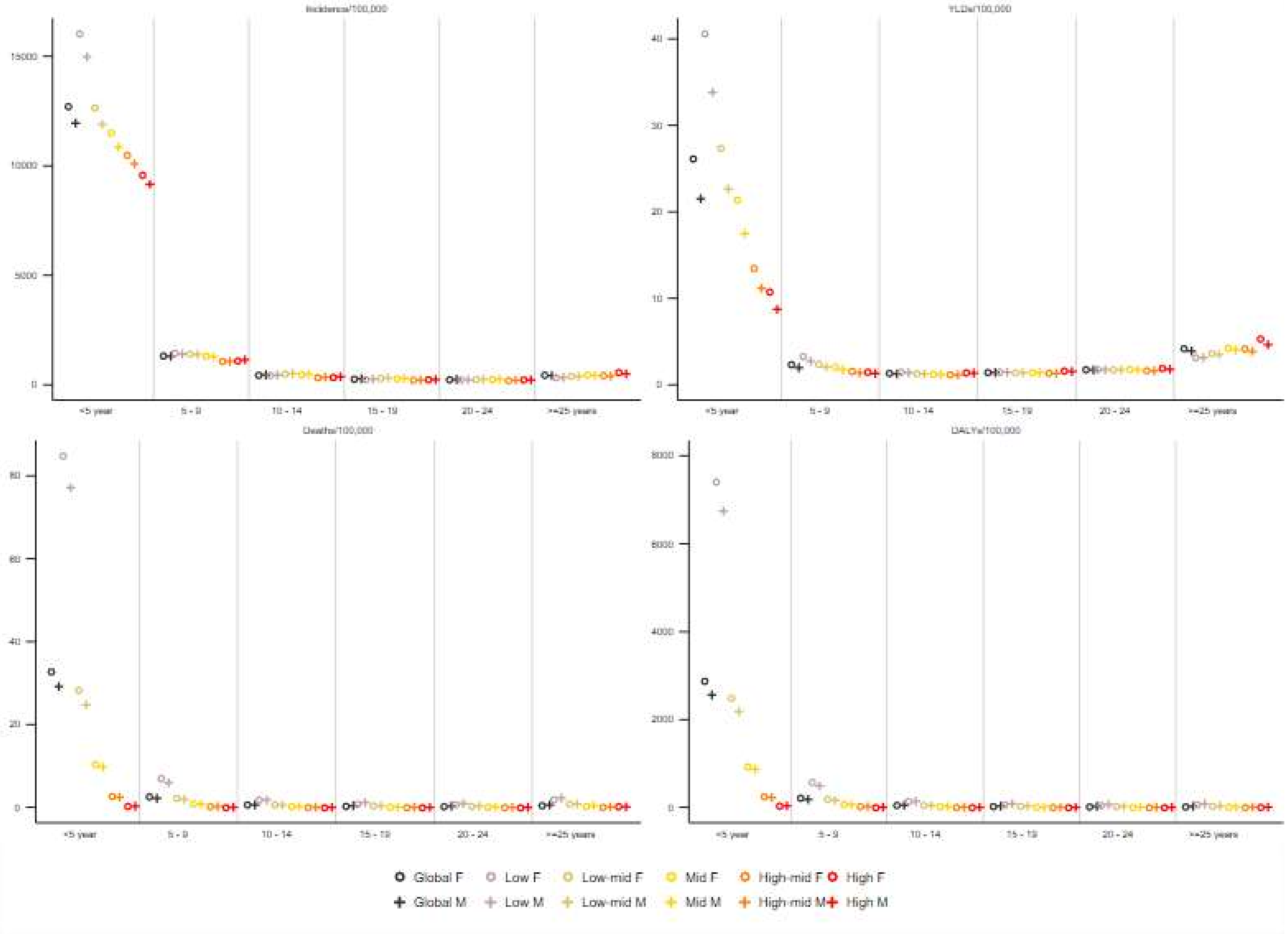

S17_16 Part B: Contribution of individual causes for death/100,000 and DALYs/ 100,00 for Vaccine preventable diseases in 2019

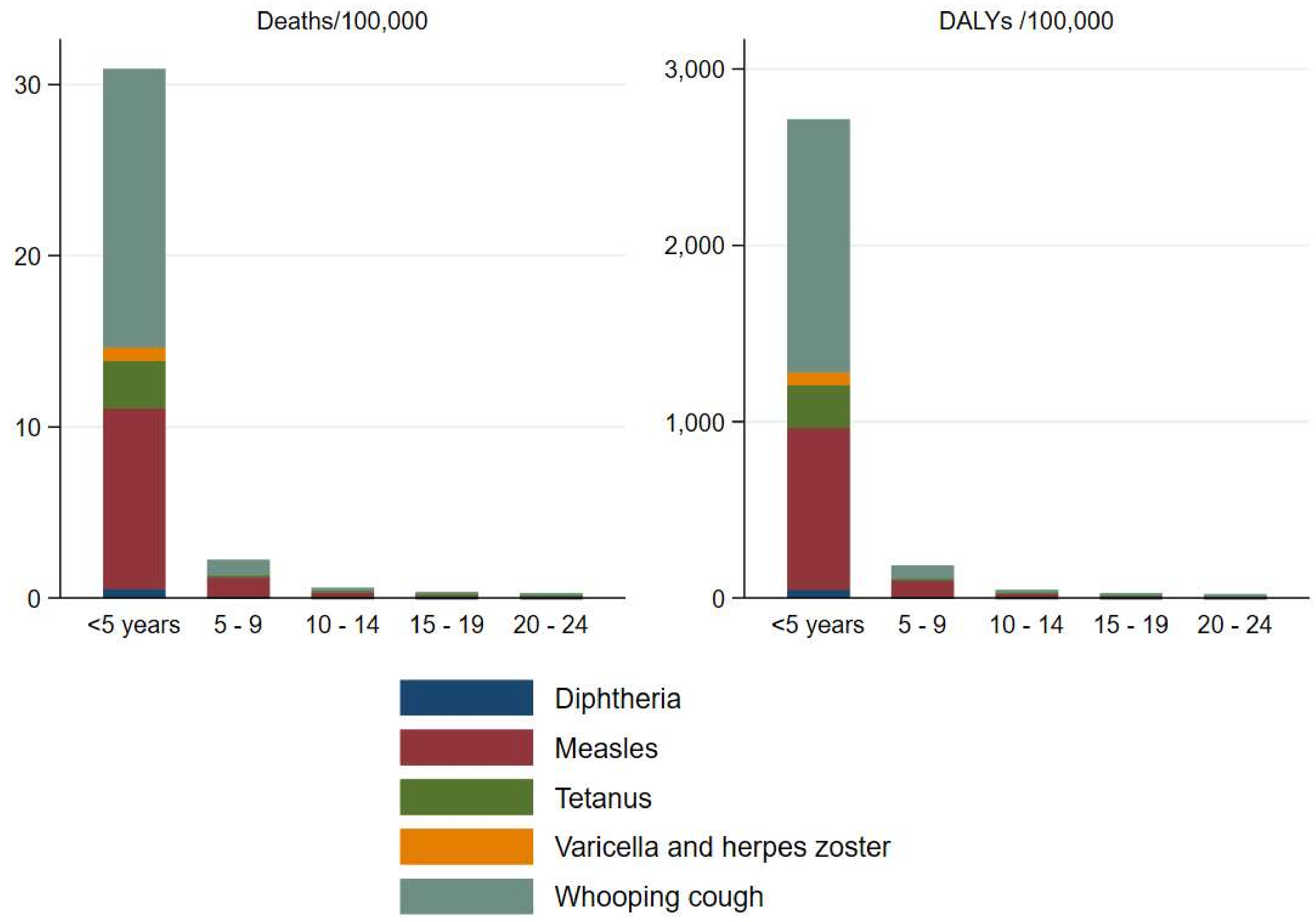

S18. HIV mortality to incidence ratio for males and females, aged <5 years, 15-19 and 20-24, in 2019.

Note that the MIR for 15-19 year old females in Syria is not shown (32).

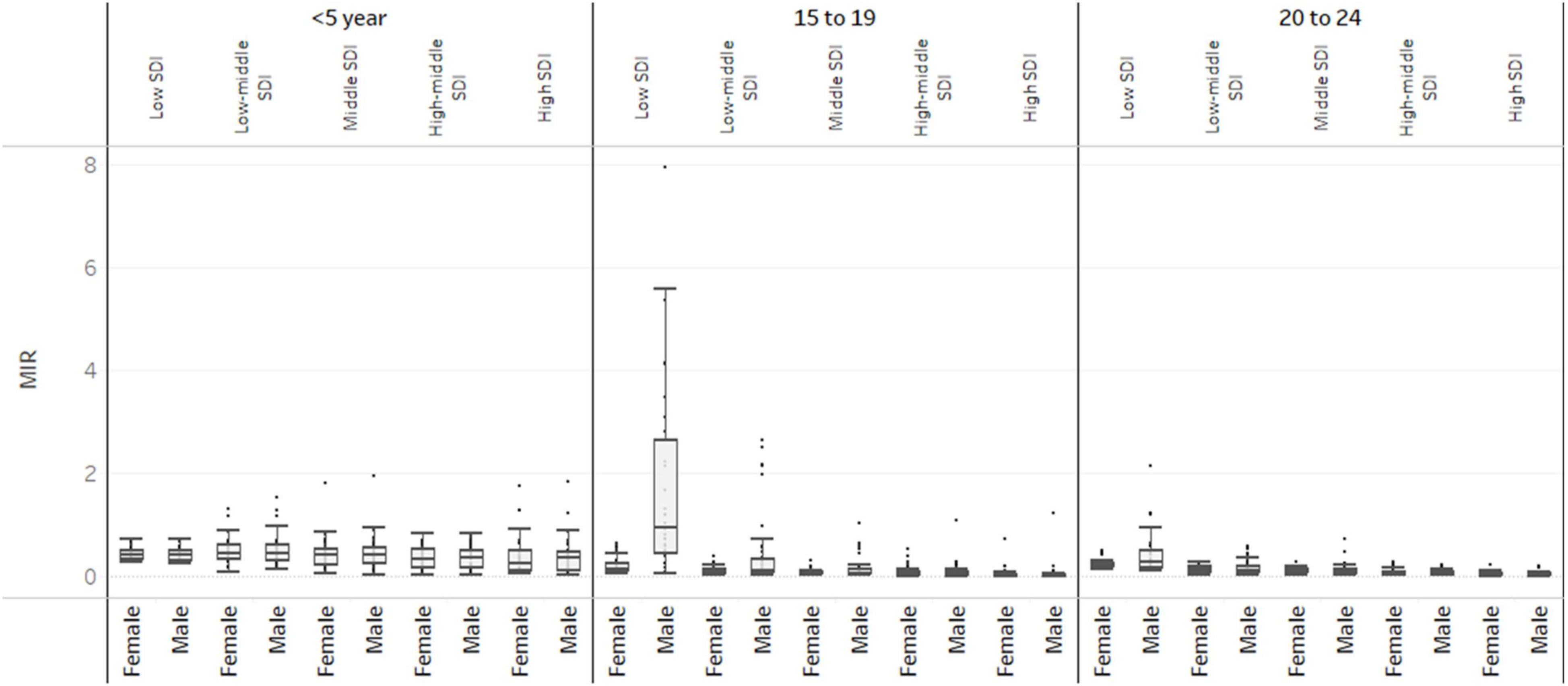

S19. Mortality to Incidence ratio by location for HIV, for females and males aged less than 5 years, 15 to 19 years and 20 to 24 years. The colour on this figure is green shades for those locations with a value less than 1.6 (the global average) and yellow to dark orange spectrum for MIR increasing from 1.6.

	<5 year		15 to 19		20 to 24	
	Female	Male	Female	Male	Female	Male
Afghanistan	0.649	0.653	0.054	0.058	0.218	0.221
Albania	0.451	0.405	0.112	0.061	0.163	0.461
Algeria	0.335	0.367	0.053	0.071	0.144	0.163
American Samoa	0.692	0.377	0.046	0.048	0.075	0.109
Andorra	0.147	0.349	0.013	0.022	0.042	0.078
Angola	0.609	0.613	0.072	0.353	0.160	0.114
Antigua and Barbuda	0.127	0.125	0.060	0.074	0.038	0.037
Argentina	0.012	0.012	0.012	0.016	0.023	0.027
Armenia	0.053	0.031	0.041	0.044	0.030	0.062
Australia	0.120	0.046	0.006	0.002	0.015	0.005
Austria	0.092	0.100	0.017	0.011	0.010	0.014
Azerbaijan	0.196	0.313	0.034	0.057	0.047	0.034
Bahamas	0.531	0.508	0.031	0.036	0.089	0.068
Bahrain	0.338	0.496	0.261	0.120	0.079	0.096
Bangladesh	0.666	0.658	0.096	0.082	0.133	0.102
Barbados	0.363	0.383	0.036	0.053	0.055	0.043
Belarus	0.171	0.218	0.226	1.083	0.031	0.040
Belgium	0.120	0.118	0.006	0.007	0.011	0.010
Belize	0.169	0.173	0.065	0.096	0.070	0.057
Benin	0.547	0.527	0.192	2.620	0.200	0.457
Bermuda	0.125	0.123	0.067	0.078	0.040	0.039
Bhutan	0.567	0.566	0.079	0.080	0.104	0.098
Bolivia	0.690	0.685	0.092	0.084	0.114	0.083
Bosnia and Herzegovina	0.214	0.397	0.045	0.049	0.182	0.045
Botswana	0.460	0.480	0.062	0.205	0.148	0.207
Brazil	0.193	0.211	0.040	0.043	0.064	0.050
Brunei Darussalam	0.487	0.482	0.018	0.017	0.035	0.030
Bulgaria	0.662	0.318	0.046	0.037	0.085	0.050
Burkina Faso	0.318	0.326	0.447	3.452	0.427	1.217
Burundi	0.311	0.311	0.645	7.924	0.506	2.137
Cabo Verde	0.277	0.244	0.125	2.167	0.120	0.425
Cambodia	0.316	0.446	0.392	0.958	0.279	0.422
Cameroon	0.357	0.350	0.132	0.724	0.279	0.271
Canada	0.483	0.430	0.008	0.011	0.016	0.012
Central African Republic	0.307	0.290	0.202	2.120	0.254	0.484
Chad	0.348	0.330	0.111	0.908	0.191	0.200
Chile	0.102	0.100	0.011	0.011	0.024	0.019
China	0.853	0.633	0.092	0.068	0.125	0.086
Colombia	1.791	1.923	0.051	0.044	0.066	0.051
Comoros	0.842	0.212	0.030	0.037	0.052	0.045
Congo	0.375	0.383	0.250	2.485	0.243	0.577
Cook Islands	0.420	0.425	0.042	0.042	0.081	0.071
Costa Rica	0.664	0.707	0.038	0.038	0.122	0.075
Côte d'Ivoire	0.492	0.512	0.290	4.128	0.282	0.930
Croatia	0.243	0.183	0.036	0.033	0.026	0.039
Cuba	0.046	0.069	0.044	0.038	0.052	0.060
Cyprus	0.142	0.429	0.022	0.013	0.070	0.037
Czechia	0.908	0.865	0.022	0.008	0.089	0.047
Dem Rep of the Congo	0.300	0.299	0.268	2.219	0.307	0.582
Denmark	0.071	0.074	0.006	0.010	0.013	0.016
DPR Korea	0.633	0.638	0.082	0.067	0.154	0.123

	<5 year		15 to 19		20 to 24	
	Female	Male	Female	Male	Female	Male
Djibouti	0.652	0.662	0.259	2.136	0.269	0.317
Dominica	0.137	0.135	0.066	0.094	0.039	0.042
Dominican Republic	0.455	0.480	0.049	0.099	0.121	0.141
Ecuador	0.526	0.577	0.048	0.045	0.072	0.063
Egypt	0.312	0.260	0.030	0.040	0.122	0.085
El Salvador	0.874	0.971	0.098	0.075	0.080	0.066
Equatorial Guinea	0.280	0.276	0.067	0.446	0.118	0.099
Eritrea	0.596	0.587	0.485	5.337	0.444	1.214
Estonia	0.235	0.250	0.026	0.021	0.039	0.050
Eswatini	0.288	0.298	0.065	0.314	0.147	0.136
Ethiopia	0.406	0.414	0.573	5.563	0.464	1.185
Fiji	0.461	0.672	0.063	0.159	0.076	0.096
Finland	0.306	0.298	0.004	0.007	0.009	0.014
France	0.354	0.372	0.010	0.011	0.016	0.017
Gabon	0.447	0.410	0.071	0.587	0.140	0.150
Gambia	0.320	0.307	0.108	0.708	0.162	0.162
Georgia	0.021	0.060	0.018	0.009	0.021	0.041
Germany	0.388	0.370	0.010	0.011	0.017	0.018
Ghana	0.588	0.553	0.232	2.630	0.254	0.532
Greece	0.315	0.216	0.008	0.013	0.015	0.025
Greenland	0.281	0.818	0.011	0.020	0.021	0.015
Grenada	0.133	0.129	0.074	0.149	0.043	0.031
Guam	0.918	0.373	0.053	0.050	0.098	0.194
Guatemala	0.279	0.263	0.056	0.057	0.092	0.076
Guinea	0.356	0.347	0.080	0.584	0.171	0.142
Guinea-Bissau	0.334	0.462	0.162	2.796	0.188	0.423
Guyana	0.121	0.119	0.059	0.133	0.065	0.065
Haiti	0.675	0.585	0.200	1.197	0.224	0.306
Honduras	0.187	0.245	0.061	0.115	0.056	0.040
Hungary	0.598	0.656	0.026	0.028	0.106	0.066
Iceland	1.748	1.839	0.093	0.023	0.111	0.018
India	0.320	0.301	0.235	0.477	0.192	0.120
Indonesia	0.586	0.939	0.048	0.044	0.150	0.091
Iran	0.518	0.585	0.047	0.045	0.158	0.125
Iraq	0.675	0.604	0.047	0.055	0.128	0.122
Ireland	0.347	0.321	0.008	0.006	0.011	0.011
Israel	0.204	0.179	0.006	0.008	0.016	0.016
Italy	0.149	0.152	0.008	0.012	0.016	0.016
Jamaica	0.798	0.756	0.048	0.074	0.097	0.095
Japan	0.502	0.615	0.003	0.006	0.010	0.012
Jordan	0.685	0.714	0.163	0.217	0.221	0.131
Kazakhstan	0.123	0.129	0.047	0.056	0.030	0.033
Kenya	0.377	0.410	0.214	1.954	0.243	0.551
Kiribati	0.419	0.597	0.080	0.102	0.096	0.117
Kuwait	1.283	0.882	0.009	0.010	0.025	0.026
Kyrgyzstan	0.161	0.183	0.023	0.018	0.021	0.028
Lao PDR	0.614	0.598	0.102	0.081	0.108	0.110
Latvia	0.313	0.358	0.053	0.043	0.045	0.023
Lebanon	0.672	0.696	0.076	0.065	0.279	0.194
Lesotho	0.362	0.362	0.089	0.289	0.157	0.162
Liberia	0.409	0.385	0.129	1.020	0.220	0.227

	<5 year		15 to 19		20 to 24	
	Female	Male	Female	Male	Female	Male
Libya	0.510	0.544	0.054	0.072	0.233	0.215
Lithuania	0.067	0.060	0.195	0.111	0.089	0.040
Luxembourg	0.644	0.636	0.014	0.015	0.015	0.016
Madagascar	0.364	0.374	0.094	0.492	0.180	0.120
Malawi	0.496	0.507	0.074	0.448	0.164	0.162
Malaysia	0.612	0.551	0.529	0.285	0.063	0.041
Maldives	0.285	1.515	0.069	0.240	0.142	0.089
Mali	0.417	0.397	0.137	0.787	0.247	0.263
Malta	0.041	0.041	0.006	0.010	0.008	0.015
Marshall Islands	0.440	0.421	0.053	0.065	0.115	0.091
Mauritania	0.443	0.126	0.054	0.084	0.063	0.060
Mauritius	0.118	0.124	0.025	0.042	0.058	0.081
Mexico	0.258	0.246	0.039	0.036	0.070	0.055
Micronesia (Federated States of)	0.545	0.564	0.079	0.092	0.137	0.125
Monaco	0.253	0.325	0.030	0.032	0.054	0.046
Mongolia	0.648	0.664	0.068	0.050	0.148	0.118
Montenegro	0.464	0.470	0.043	0.048	0.090	0.079
Morocco	0.497	0.511	0.077	0.143	0.207	0.276
Mozambique	0.450	0.460	0.082	0.231	0.216	0.147
Myanmar	0.319	0.322	0.225	0.306	0.144	0.098
Namibia	0.417	0.411	0.180	1.028	0.181	0.239
Nauru	0.448	0.422	0.046	0.047	0.082	0.074
Nepal	0.715	0.713	0.236	0.166	0.136	0.124
Netherlands	0.071	0.068	0.008	0.008	0.014	0.011
New Zealand	0.079	0.111	0.006	0.006	0.016	0.011
Nicaragua	0.547	0.451	0.067	0.062	0.087	0.065
Niger	0.464	0.452	0.213	1.194	0.235	0.316
Nigeria	0.437	0.424	0.118	0.564	0.190	0.165
Niue	0.442	0.416	0.046	0.049	0.083	0.074
North Macedonia	0.456	0.466	0.069	0.062	0.074	0.102
Northern Mariana Islands	0.599	0.458	0.097	0.051	0.154	0.113
Norway	0.077	0.062	0.008	0.006	0.015	0.010
Oman	0.399	0.325	0.126	0.225	0.050	0.068
Pakistan	0.309	0.310	0.051	0.046	0.120	0.093
Palau	0.484	0.457	0.043	0.049	0.079	0.075
Palestine	0.609	0.586	0.071	0.068	0.259	0.223
Panama	0.321	0.315	0.067	0.055	0.063	0.051
Papua New Guinea	0.536	0.566	0.114	0.707	0.177	0.144
Paraguay	0.656	0.881	0.060	0.075	0.128	0.087
Peru	0.704	0.536	0.173	0.124	0.071	0.046
Philippines	0.118	0.115	0.025	0.020	0.076	0.045
Poland	0.157	0.177	0.016	0.013	0.046	0.041
Portugal	0.633	0.599	0.017	0.021	0.031	0.032
Puerto Rico	0.113	0.113	0.192	0.194	0.043	0.036
Qatar	0.676	0.500	0.716	1.207	0.075	0.171
Republic of Korea	0.058	0.088	0.002	0.010	0.008	0.021
Republic of Moldova	0.042	0.059	0.017	0.027	0.036	0.034
Romania	0.171	0.167	0.045	0.073	0.052	0.047
Russian Federation	0.241	0.240	0.049	0.073	0.036	0.035
Rwanda	0.284	0.275	0.162	1.308	0.211	0.445
Saint Kitts and Nevis	0.447	0.417	0.074	0.076	0.088	0.074

	<5 year		15 to 19		20 to 24	
	Female	Male	Female	Male	Female	Male
Saint Lucia	0.15	0.15	0.08	0.15	0.04	0.04
Saint Vincent and the Grenadines	0.21	0.21	0.11	0.20	0.06	0.05
Samoa	0.40	0.40	0.05	0.06	0.12	0.09
San Marino	0.28	0.35	0.03	0.03	0.05	0.04
Sao Tome and Principe	0.60	0.17	0.03	0.05	0.05	0.05
Saudi Arabia	0.54	0.78	0.15	0.27	0.15	0.20
Senegal	0.38	0.37	0.19	1.67	0.22	0.32
Serbia	0.83	0.41	0.38	0.08	0.08	0.07
Seychelles	0.25	0.48	0.05	0.08	0.11	0.06
Sierra Leone	0.35	0.32	0.07	0.23	0.23	0.18
Singapore	0.09	0.02	0.04	0.01	0.02	0.01
Slovakia	0.23	0.35	0.04	0.05	0.10	0.16
Slovenia	1.27	1.21	0.01	0.02	0.02	0.03
Solomon Islands	0.52	0.50	0.06	0.08	0.12	0.10
Somalia	0.48	0.47	0.55	4.11	0.42	0.63
South Africa	0.38	0.46	0.07	0.51	0.14	0.14
South Sudan	0.42	0.43	0.10	0.61	0.18	0.15
Spain	0.38	0.35	0.01	0.01	0.02	0.02
Sri Lanka	0.53	0.63	0.05	0.04	0.12	0.10
Sudan	1.29	1.28	0.07	0.30	0.14	0.09
Suriname	0.27	0.28	0.05	0.11	0.07	0.05
Sweden	0.44	0.48	0.00	0.01	0.01	0.01
Switzerland	0.48	0.51	0.01	0.01	0.01	0.01
Syrian Arab Republic	0.35	0.33	32.01	0.04	0.15	0.25
Taiwan (Province of China)	0.11	0.11	0.01	0.01	0.02	0.01
Tajikistan	0.08	0.13	0.05	0.32	0.04	0.03
Thailand	0.43	0.44	0.04	0.04	0.09	0.07
Timor-Leste	0.48	0.46	0.18	0.18	0.24	0.18
Togo	0.28	0.26	0.29	3.06	0.24	0.58
Tokelau	0.44	0.42	0.05	0.06	0.09	0.08
Tonga	0.63	0.39	0.04	0.05	0.11	0.14
Trinidad and Tobago	0.33	0.34	0.05	0.09	0.06	0.06
Tunisia	0.50	0.53	0.04	0.04	0.28	0.70
Turkey	0.69	0.59	0.04	0.04	0.18	0.17
Turkmenistan	0.25	0.25	0.31	0.62	0.03	0.04
Tuvalu	0.43	0.41	0.05	0.05	0.09	0.08
Uganda	0.31	0.30	0.12	0.95	0.13	0.19
Ukraine	0.10	0.11	0.12	0.20	0.04	0.04
United Arab Emirates	0.64	0.68	0.06	0.08	0.21	0.18
United Kingdom	0.17	0.18	0.01	0.00	0.01	0.01
United Republic of Tanzania	0.27	0.26	0.12	0.39	0.16	0.28
United States of America	0.16	0.18	0.01	0.01	0.01	0.01
United States Virgin Islands	0.22	0.22	0.04	0.04	0.05	0.05
Uruguay	0.10	0.08	0.01	0.02	0.02	0.02
Uzbekistan	0.06	0.10	0.07	0.10	0.03	0.04
Vanuatu	0.46	0.44	0.06	0.07	0.12	0.09
Venezuela	1.17	1.15	0.04	0.04	0.07	0.05
Viet Nam	0.15	0.22	0.03	0.04	0.06	0.10
Yemen	0.66	0.68	0.05	0.06	0.20	0.21
Zambia	0.44	0.44	0.09	0.29	0.20	0.25
Zimbabwe	0.26	0.26	0.13	0.54	0.23	0.36

S20_1 Part A: Enteric Infections Death/ 100 000 Uncertainty interval for each age group (full data at: https://vizhub.healthdata.org/gbd-results/)

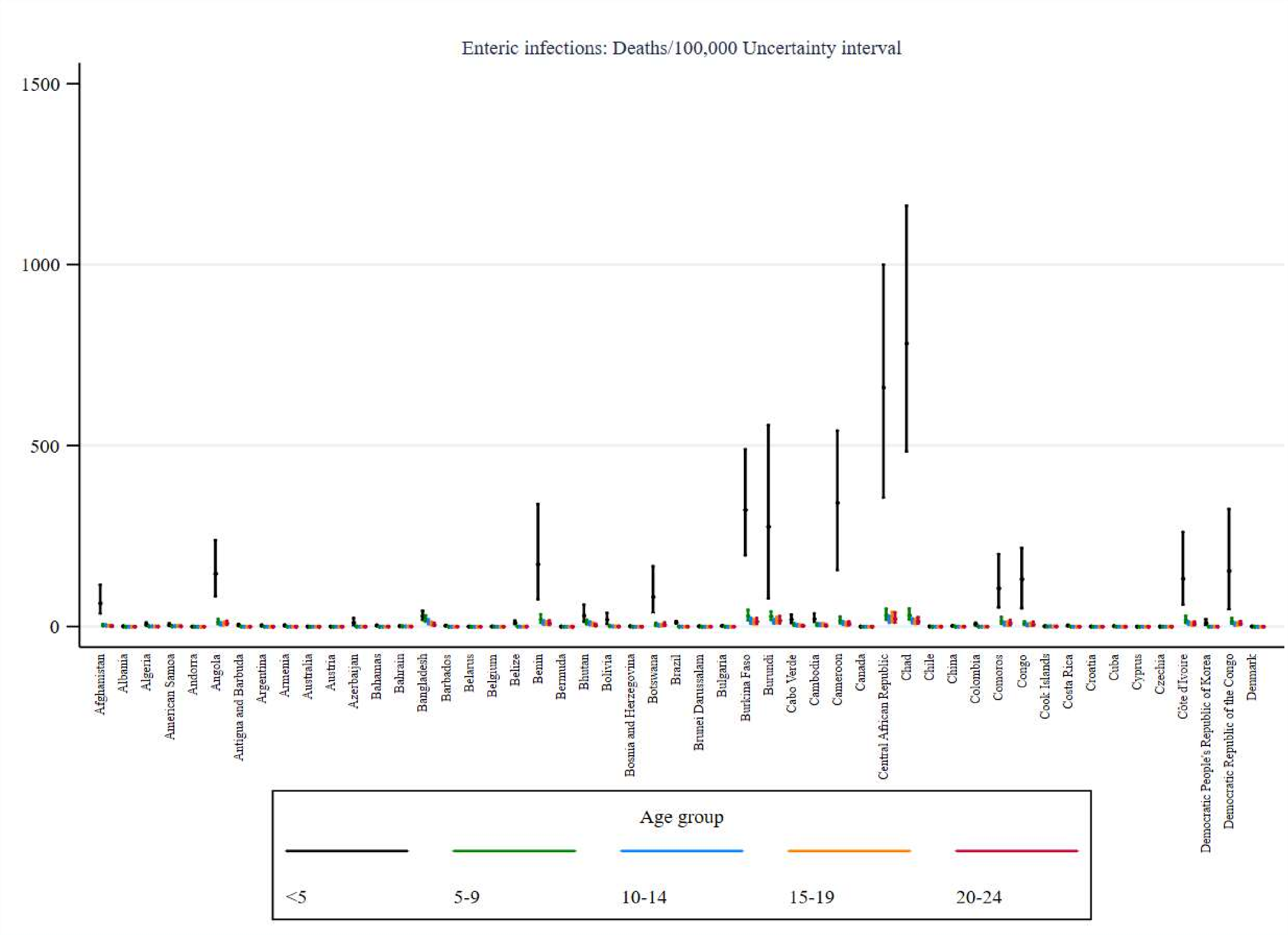

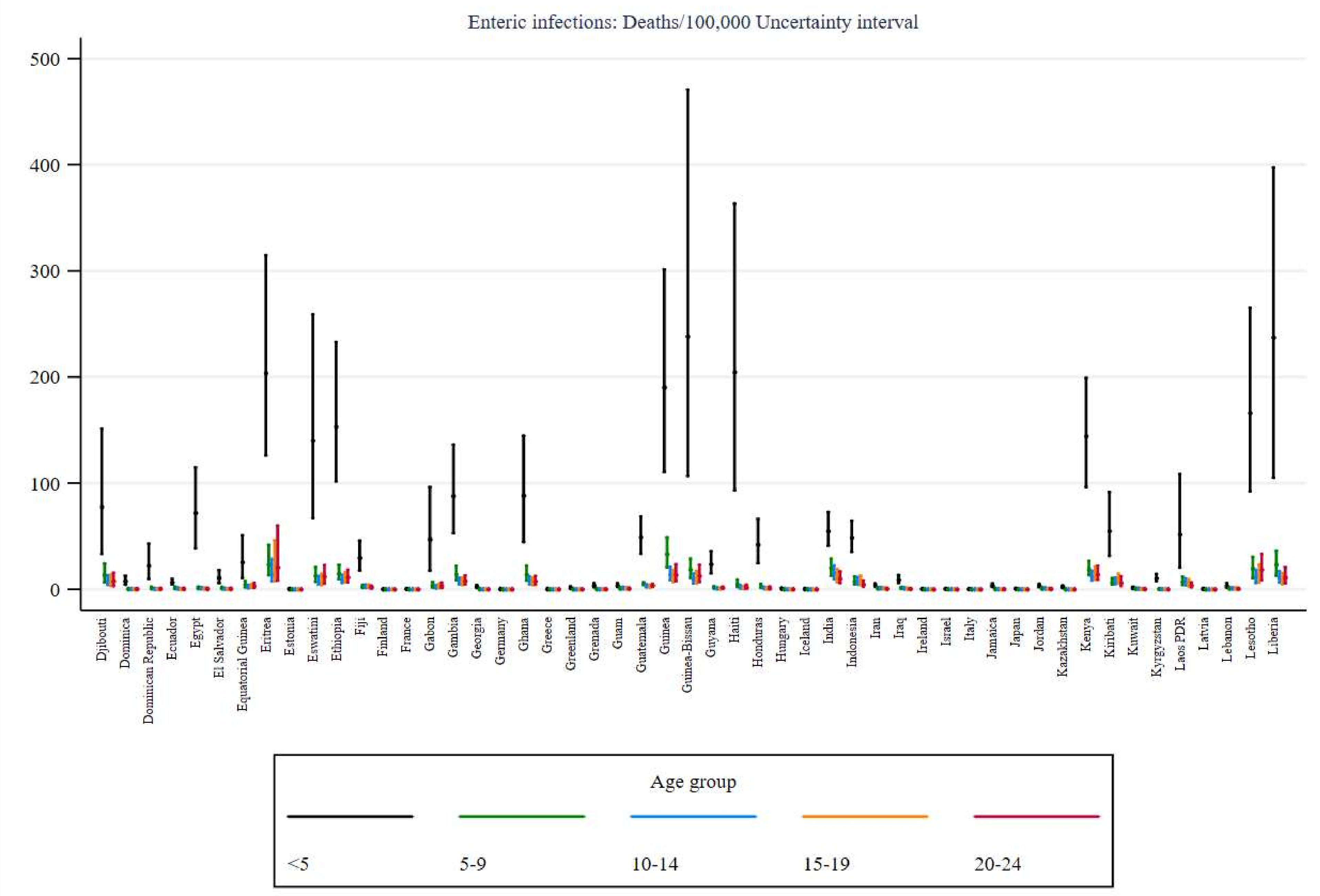
Enteric infections: Deaths/100,000 Uncertainty interval
500
400
300
200
100
0
Djibouti
Dominica
Dominican Republic
Ecuador
Egypt
El Salvador
Equatorial Guinea
Eritrea
Estonia
Eswatini
Ethiopia
Fiji
Finland
France
Gabon
Gambia
Georgia
Germany
Ghana
Greece
Greenland
Grenada
Guam
Guatemala
Guinea
Guinea-Bissau
Guyana
Haiti
Honduras
Hungary
Iceland
India
Indonesia
Iran
Iraq
Ireland
Israel
Italy
Jamaica
Japan
Jordan
Kazakhstan
Kenya
Kiribati
Kuwait
Kyrgyzstan
Laos PDR
Latvia
Lebanon
Lesotho
Liberia
Age group
<5
5-9
10-14
15-19
20-24

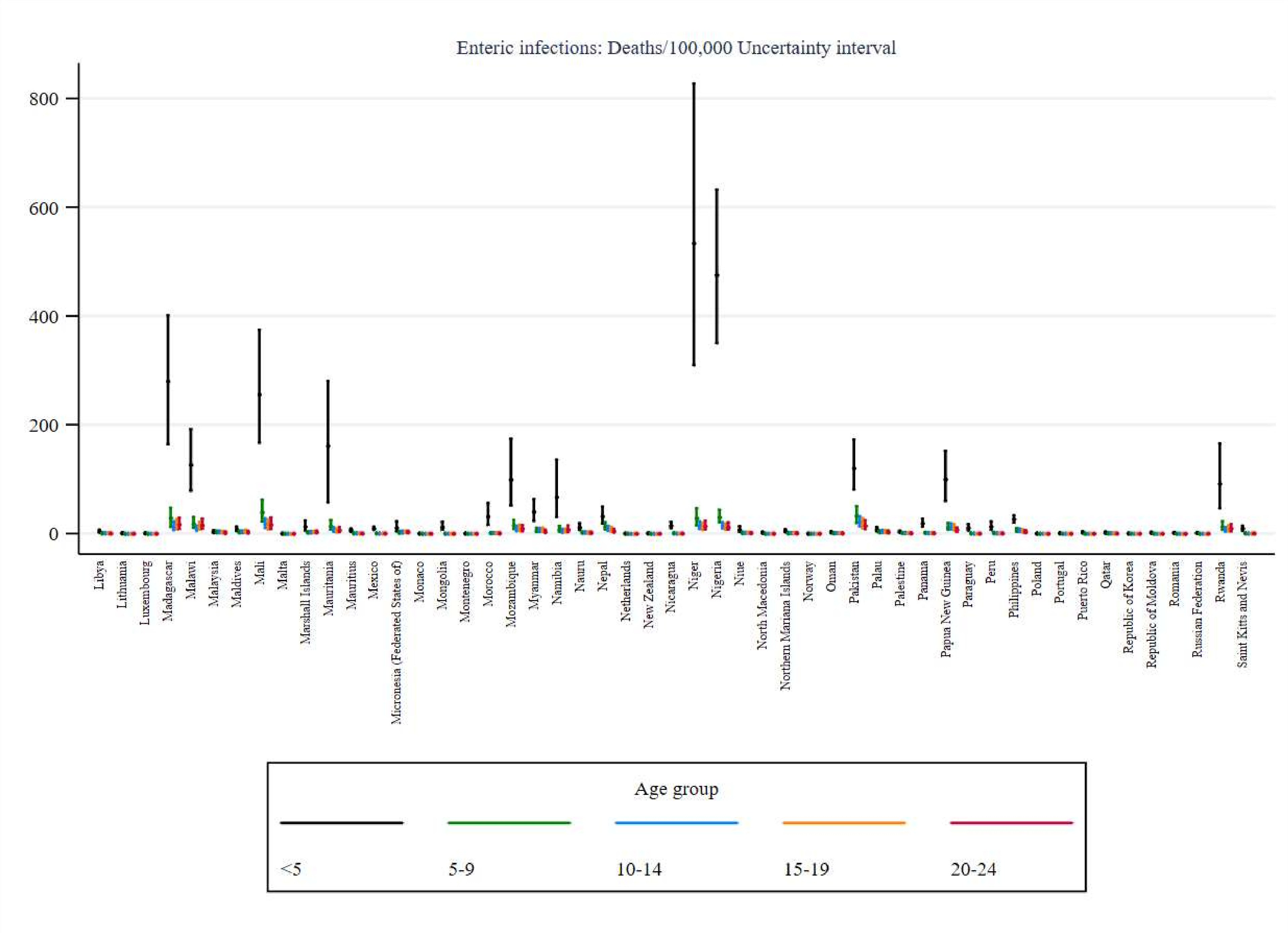
Enteric infections: Deaths/100,000 Uncertainty interval
800
600
400
200
0
Libya
Lithuania
Luxembourg
Madagascar
Malawi
Malaysia
Maldives
Mali
Malta
Marshall Islands
Mauritania
Mauritius
Mexico
Micronesia (Federated States of)
Monaco
Mongolia
Montenegro
Morocco
Mozambique
Myanmar
Namibia
Nauru
Nepal
Netherlands
New Zealand
Nicaragua
Niger
Nigeria
Niue
North Macedonia
Northern Mariana Islands
Norway
Oman
Pakistan
Palau
Palestine
Panama
Papua New Guinea
Paraguay
Peru
Philippines
Poland
Portugal
Puerto Rico
Qatar
Republic of Korea
Republic of Moldova
Romania
Russian Federation
Rwanda
Saint Kitts and Nevis
Age group
<5
5-9
10-14
15-19
20-24

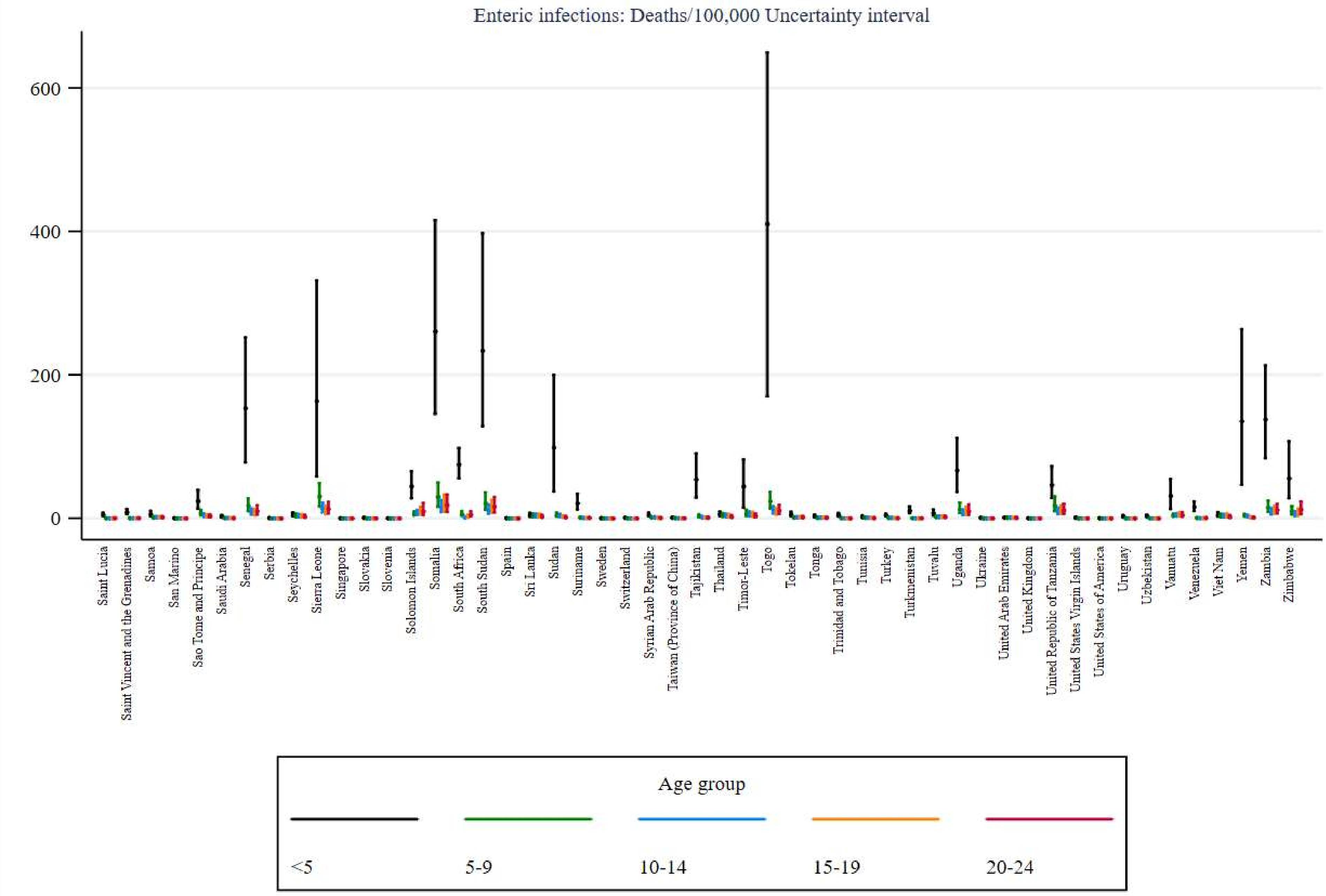

Enteric infections: Deaths/100,000 Uncertainty interval
600
400
200
0
Saint Lucia
Saint Vincent and the Grenadines
Samoa
San Marino
Sao Tome and Principe
Saudi Arabia
Senegal
Serbia
Seychelles
Sierra Leone
Singapore
Slovakia
Slovenia
Solomon Islands
Somalia
South Africa
South Sudan
Spain
Sri Lanka
Sudan
Suriname
Sweden
Switzerland
Syrian Arab Republic
Taiwan (Province of China)
Tajikistan
Thailand
Timor-Leste
Togo
Tokelau
Tonga
Trinidad and Tobago
Tunisia
Turkey
Turkmenistan
Tuvalu
Uganda
Ukraine
United Arab Emirates
United Kingdom
United Republic of Tanzania
United States Virgin Islands
United States of America
Uruguay
Uzbekistan
Vanuatu
Venezuela
Viet Nam
Yemen
Zambia
Zimbabwe
Age group
<5
5-9
10-14
15-19
20-24

S20_1 Part B: Enteric Infections DALYs/ 100 000 Uncertainty interval for each age group

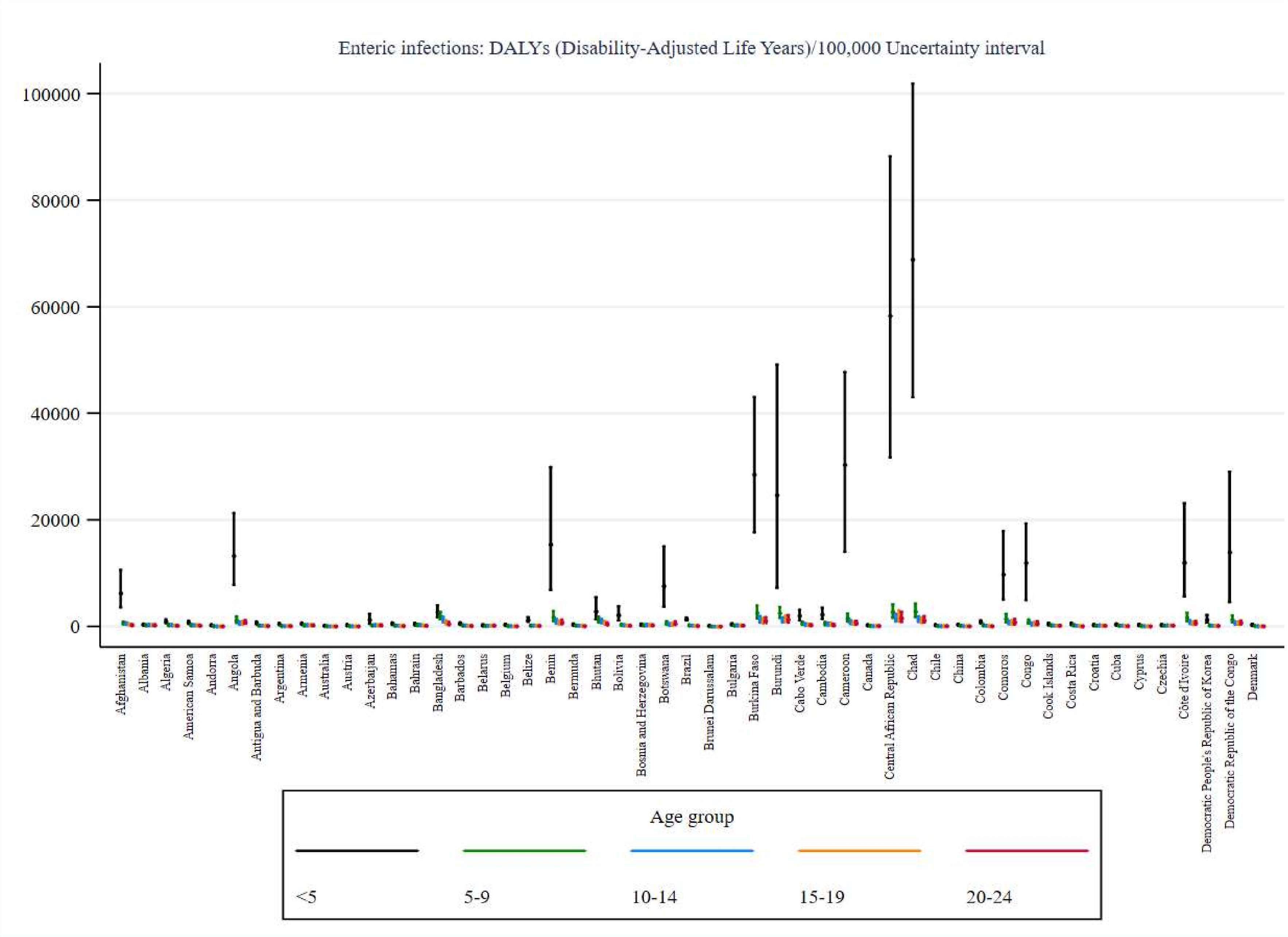

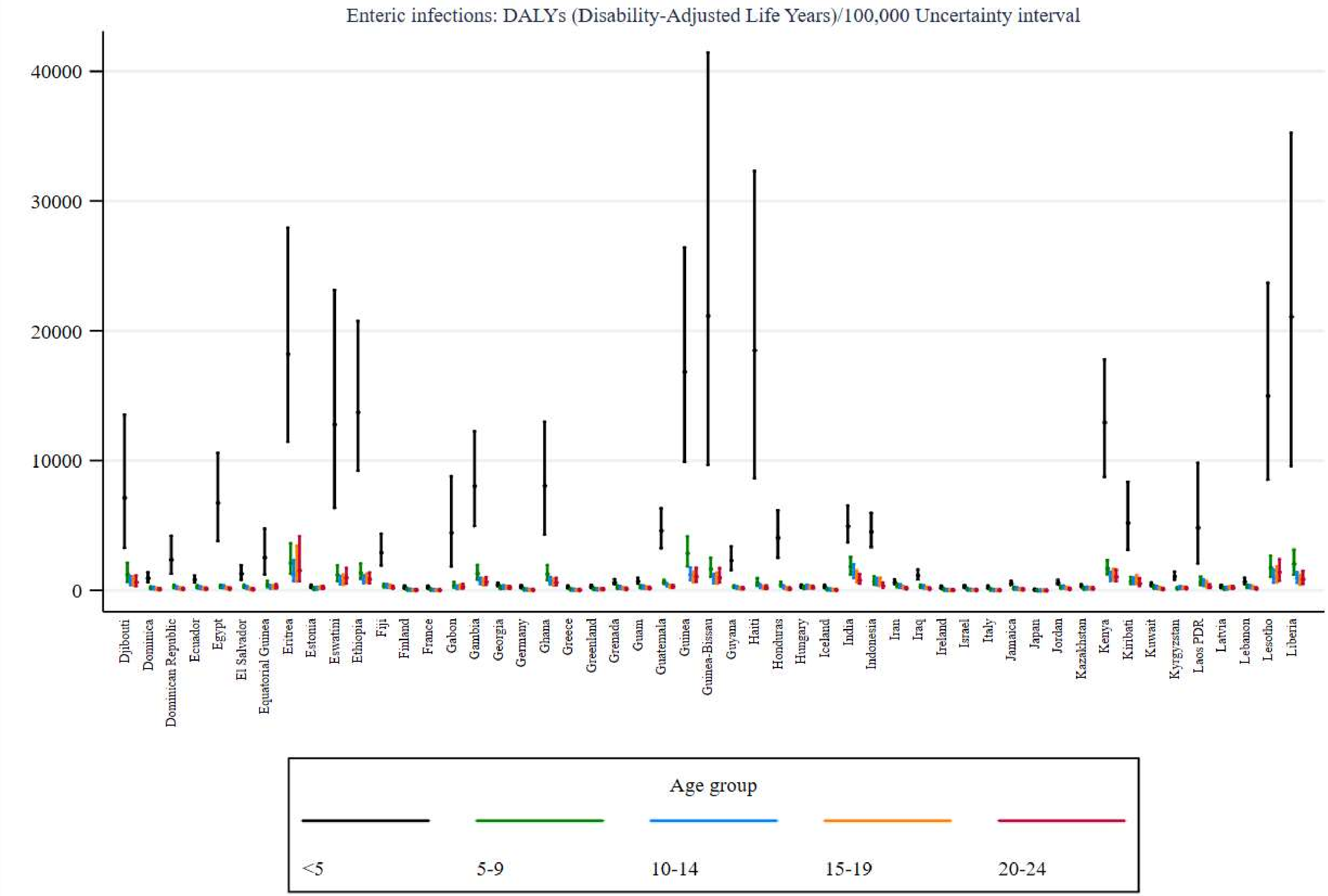

Enteric infections: DALYs (Disability-Adjusted Life Years)/100,000 Uncertainty interval
40000
30000
20000
10000
0
Djibouti
Dominica
Dominican Republic
Ecuador
Egypt
El Salvador
Equatorial Guinea
Eritrea
Estonia
Eswatini
Ethiopia
Fiji
Finland
France
Gabon
Gambia
Georgia
Germany
Ghana
Greece
Greenland
Grenada
Guam
Guatemala
Guinea
Guinea-Bissau
Guyana
Haiti
Honduras
Hungary
Iceland
India
Indonesia
Iran
Iraq
Ireland
Israel
Italy
Jamaica
Japan
Jordan
Kazakhstan
Kenya
Kiribati
Kuwait
Kyrgyzstan
Laos PDR
Latvia
Lebanon
Lesotho
Liberia
Age group
<5
5-9
10-14
15-19
20-24

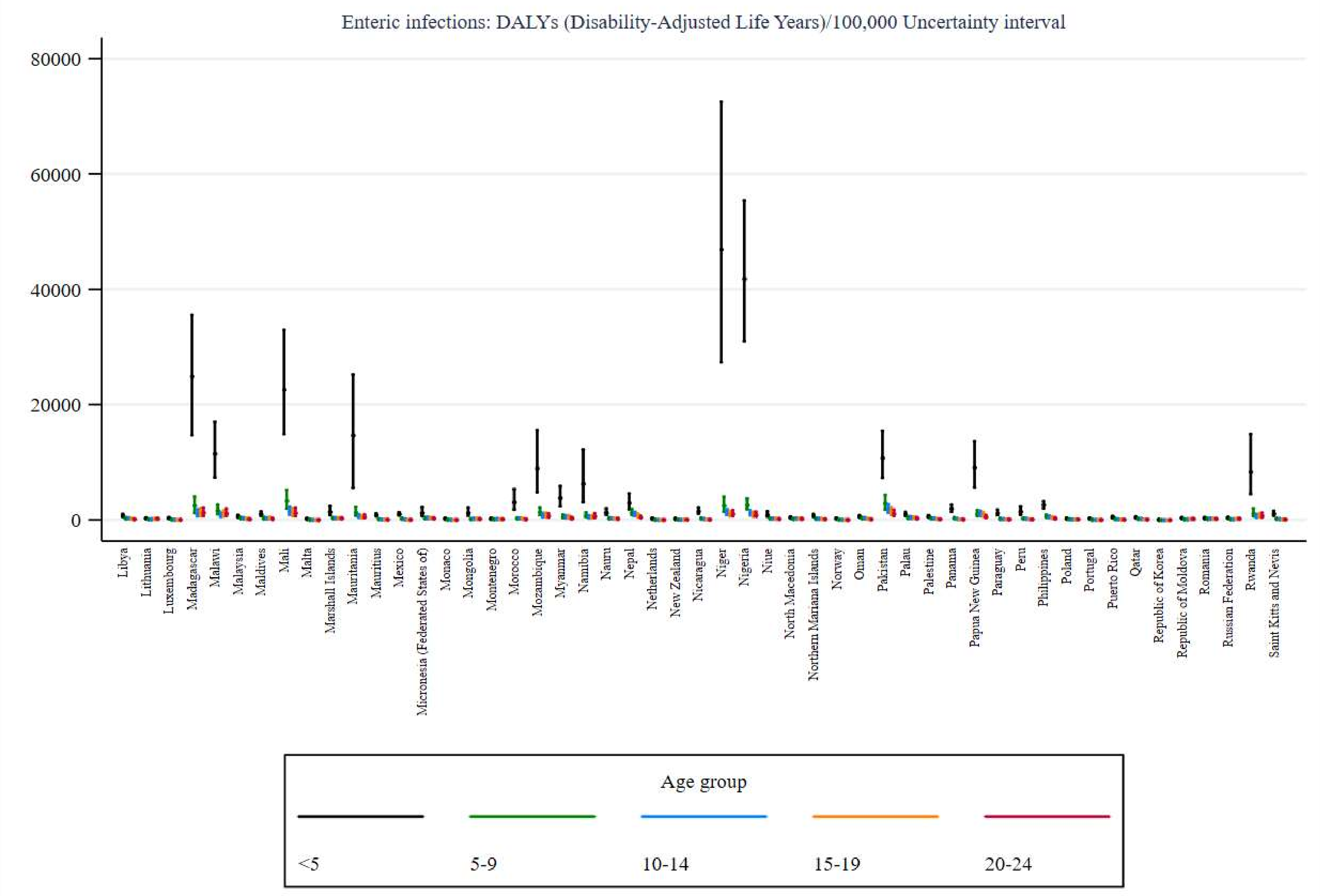
Enteric infections: DALYs (Disability-Adjusted Life Years)/100,000 Uncertainty interval
80000
60000
40000
20000
0
Libya
Lithuania
Luxembourg
Madagascar
Malawi
Malaysia
Maldives
Mali
Malta
Marshall Islands
Mauritania
Mauritius
Mexico
Micronesia (Federated States of)
Monaco
Mongolia
Montenegro
Morocco
Mozambique
Myanmar
Namibia
Nauru
Nepal
Netherlands
New Zealand
Nicaragua
Niger
Nigeria
Niue
North Macedonia
Northern Mariana Islands
Norway
Oman
Pakistan
Palau
Palestine
Panama
Papua New Guinea
Paraguay
Peru
Philippines
Poland
Portugal
Puerto Rico
Qatar
Republic of Korea
Republic of Moldova
Romania
Russian Federation
Rwanda
Saint Kitts and Nevis
Age group
<5
5-9
10-14
15-19
20-24

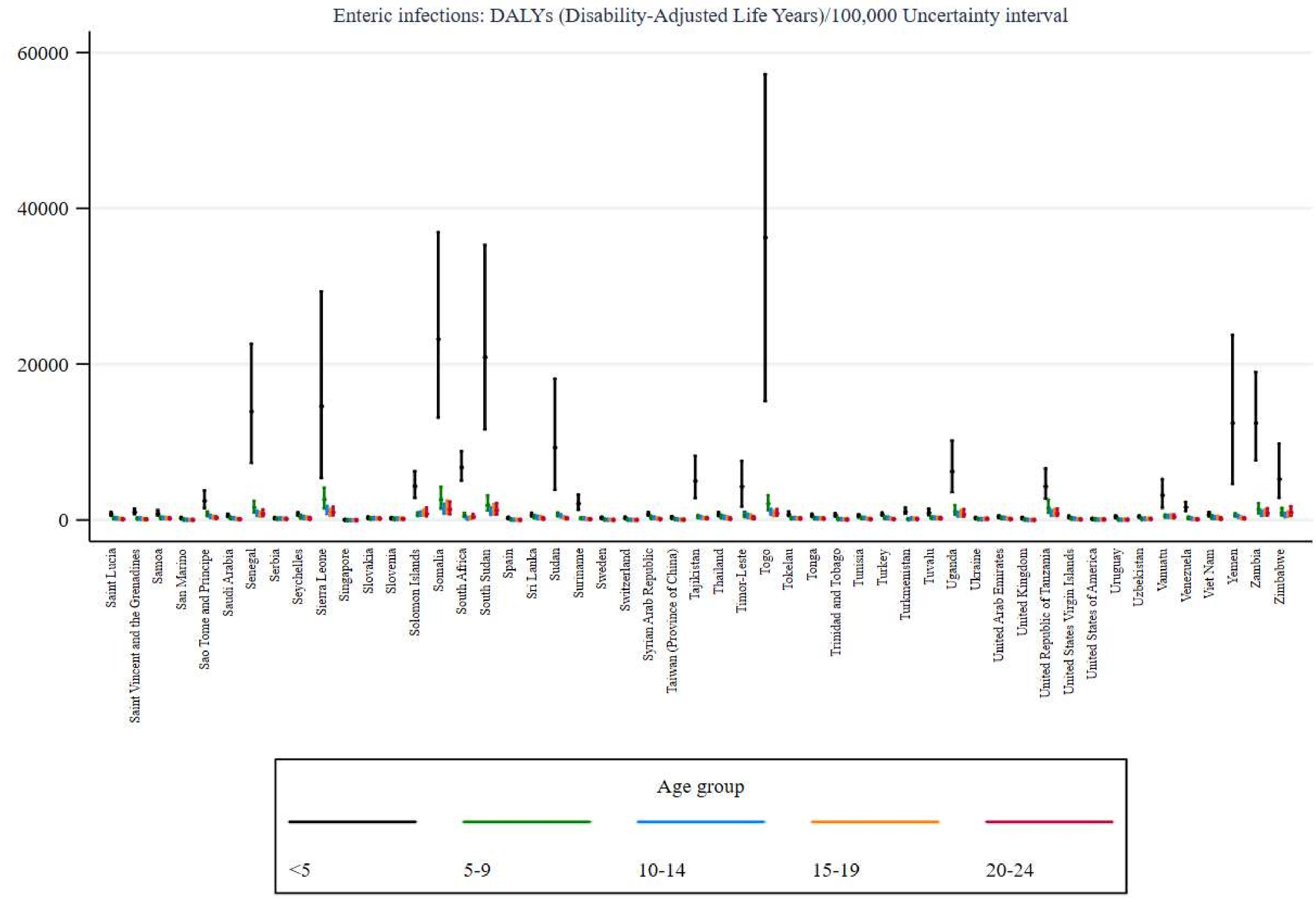

Enteric infections: DALYs (Disability-Adjusted Life Years)/100,000 Uncertainty interval
60000
40000
20000
0
Saint Lucia
Saint Vincent and the Grenadines
Samoa
San Marino
Sao Tome and Principe
Saudi Arabia
Senegal
Serbia
Seychelles
Sierra Leone
Singapore
Slovakia
Slovenia
Solomon Islands
Somalia
South Africa
South Sudan
Spain
Sri Lanka
Sudan
Suriname
Sweden
Switzerland
Syrian Arab Republic
Taiwan (Province of China)
Tajikistan
Thailand
Timor-Leste
Togo
Tokelau
Tonga
Trinidad and Tobago
Tunisia
Turkey
Turkmenistan
Tuvalu
Uganda
Ukraine
United Arab Emirates
United Kingdom
United Republic of Tanzania
United States Virgin Islands
United States of America
Uruguay
Uzbekistan
Vanuatu
Venezuela
Viet Nam
Yemen
Zambia
Zimbabwe
Age group
<5
5-9
10-14
15-19
20-24

S20_2 Part A: HIV Deaths/ 100 000 Uncertainty interval for each age group

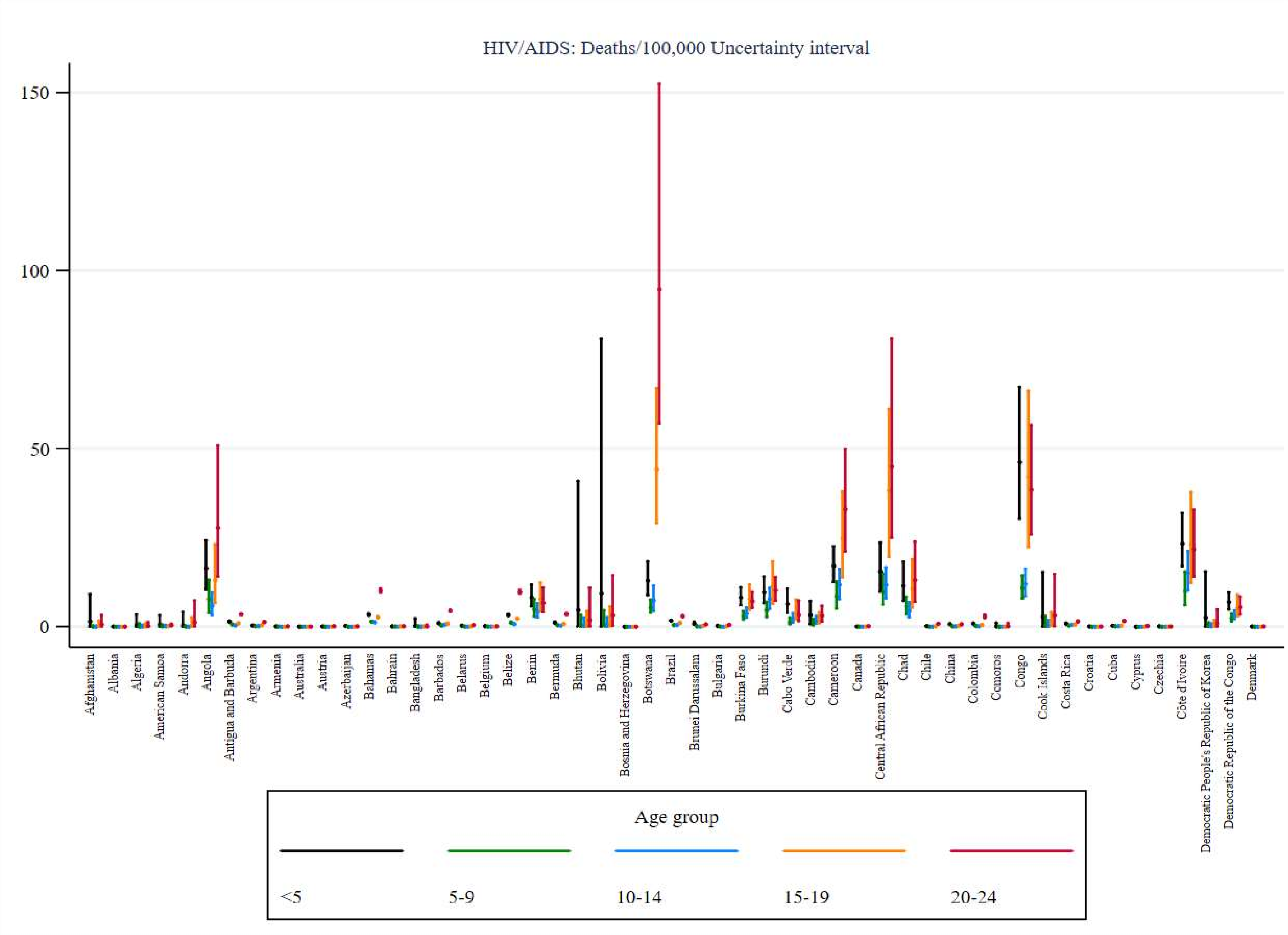

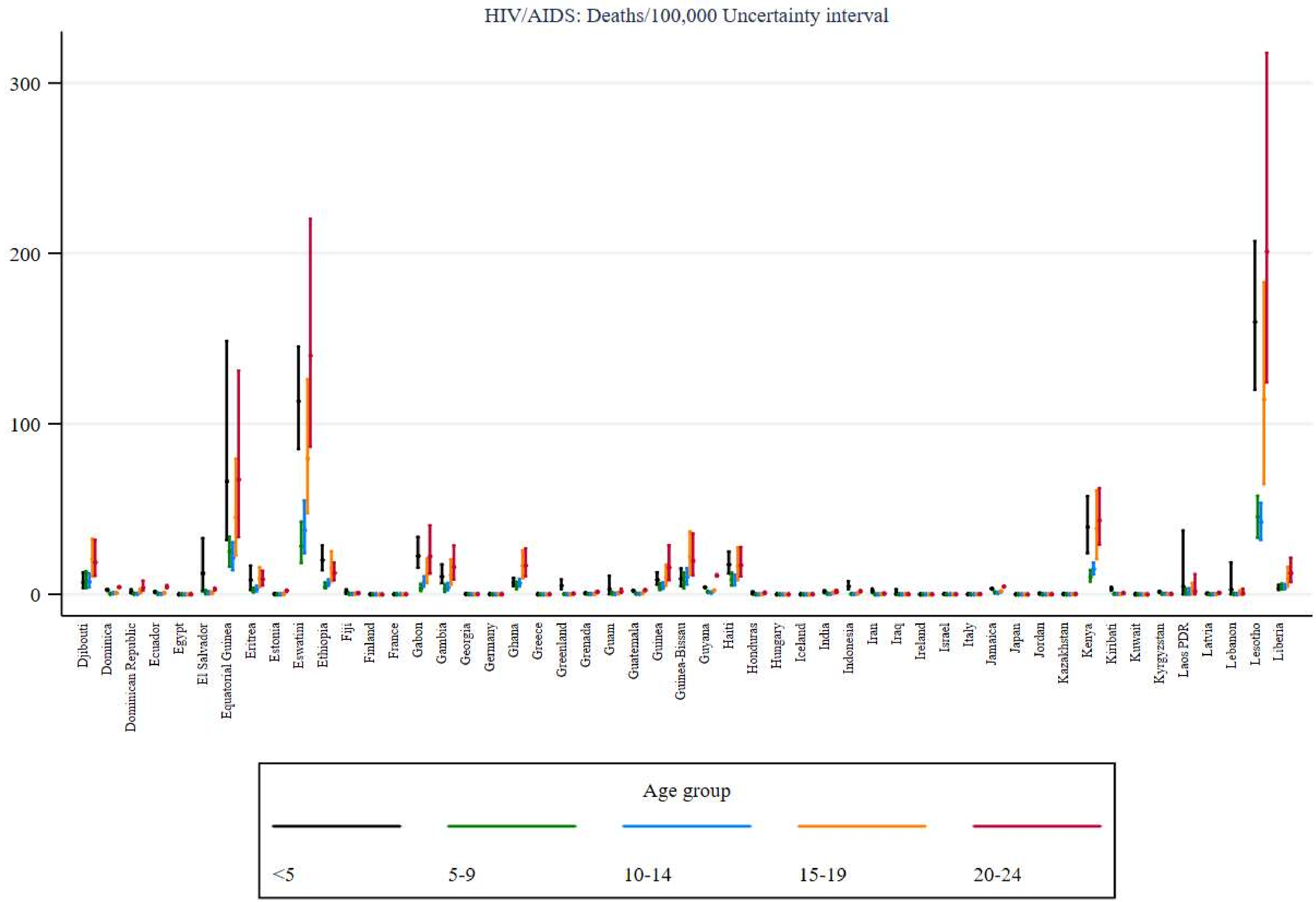
HIV/AIDS: Deaths/100,000 Uncertainty interval
300
200
100
0
Djibouti
Dominica
Dominican Republic
Ecuador
Egypt
El Salvador
Equatorial Guinea
Eritrea
Estonia
Eswatini
Ethiopia
Fiji
Finland
France
Gabon
Gambia
Georgia
Germany
Ghana
Greece
Greenland
Grenada
Guam
Guatemala
Guinea
Guinea-Bissau
Guyana
Haiti
Honduras
Hungary
Iceland
India
Indonesia
Iran
Iraq
Ireland
Israel
Italy
Jamaica
Japan
Jordan
Kazakhstan
Kenya
Kiribati
Kuwait
Kyrgyzstan
Laos PDR
Latvia
Lebanon
Lesotho
Liberia
Age group
<5
5-9
10-14
15-19
20-24

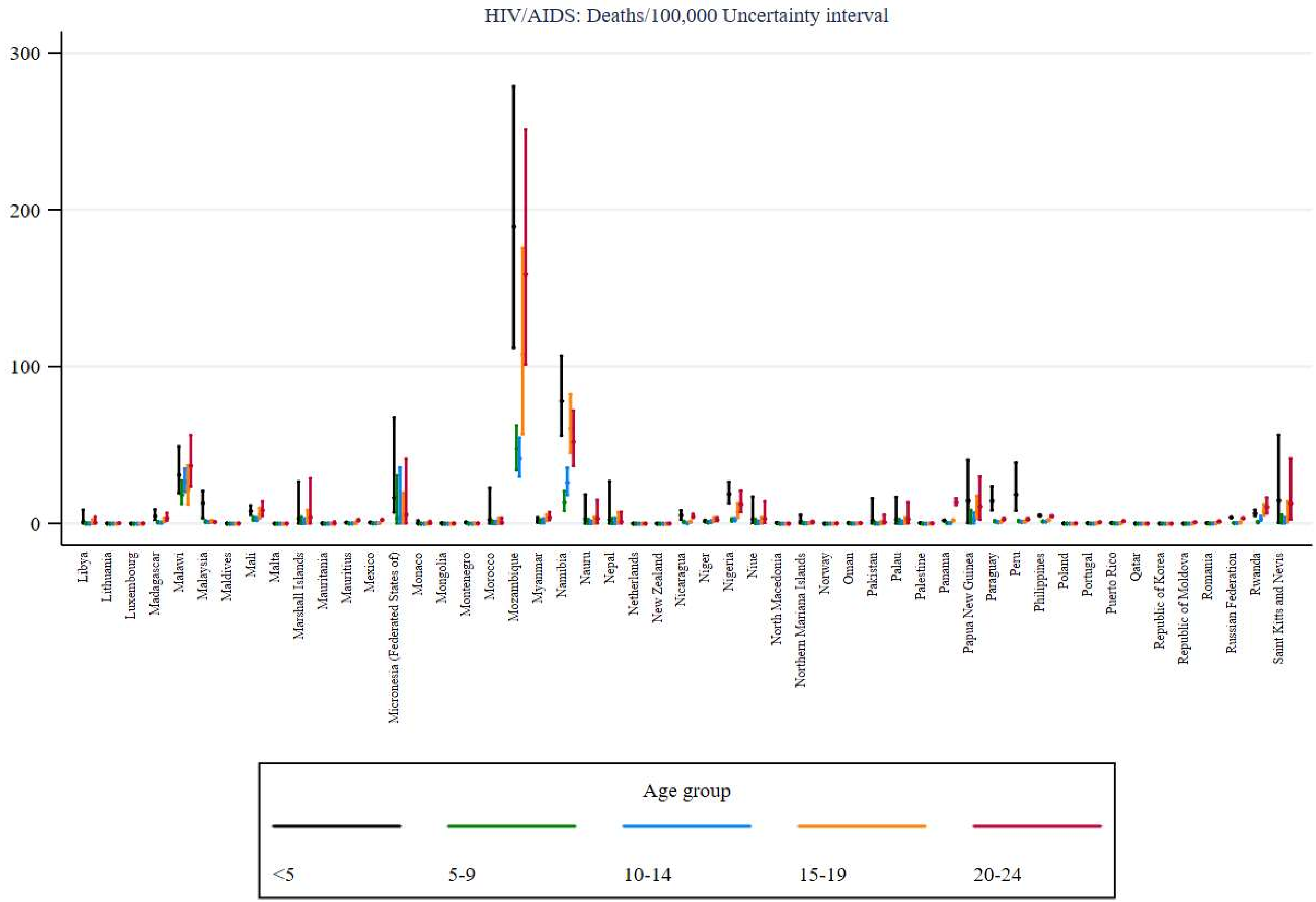

HIV/AIDS: Deaths/100,000 Uncertainty interval
300
200
100
0
Libya
Lithuania
Luxembourg
Madagascar
Malawi
Malaysia
Maldives
Mali
Malta
Marshall Islands
Mauritania
Mauritius
Mexico
Micronesia (Federated States of)
Monaco
Mongolia
Montenegro
Morocco
Mozambique
Myanmar
Namibia
Nauru
Nepal
Netherlands
New Zealand
Nicaragua
Niger
Nigeria
Niue
North Macedonia
Northern Mariana Islands
Norway
Oman
Pakistan
Palau
Palestine
Panama
Papua New Guinea
Paraguay
Peru
Philippines
Poland
Portugal
Puerto Rico
Qatar
Republic of Korea
Republic of Moldova
Romania
Russian Federation
Rwanda
Saint Kitts and Nevis
Age group
<5
5-9
10-14
15-19
20-24

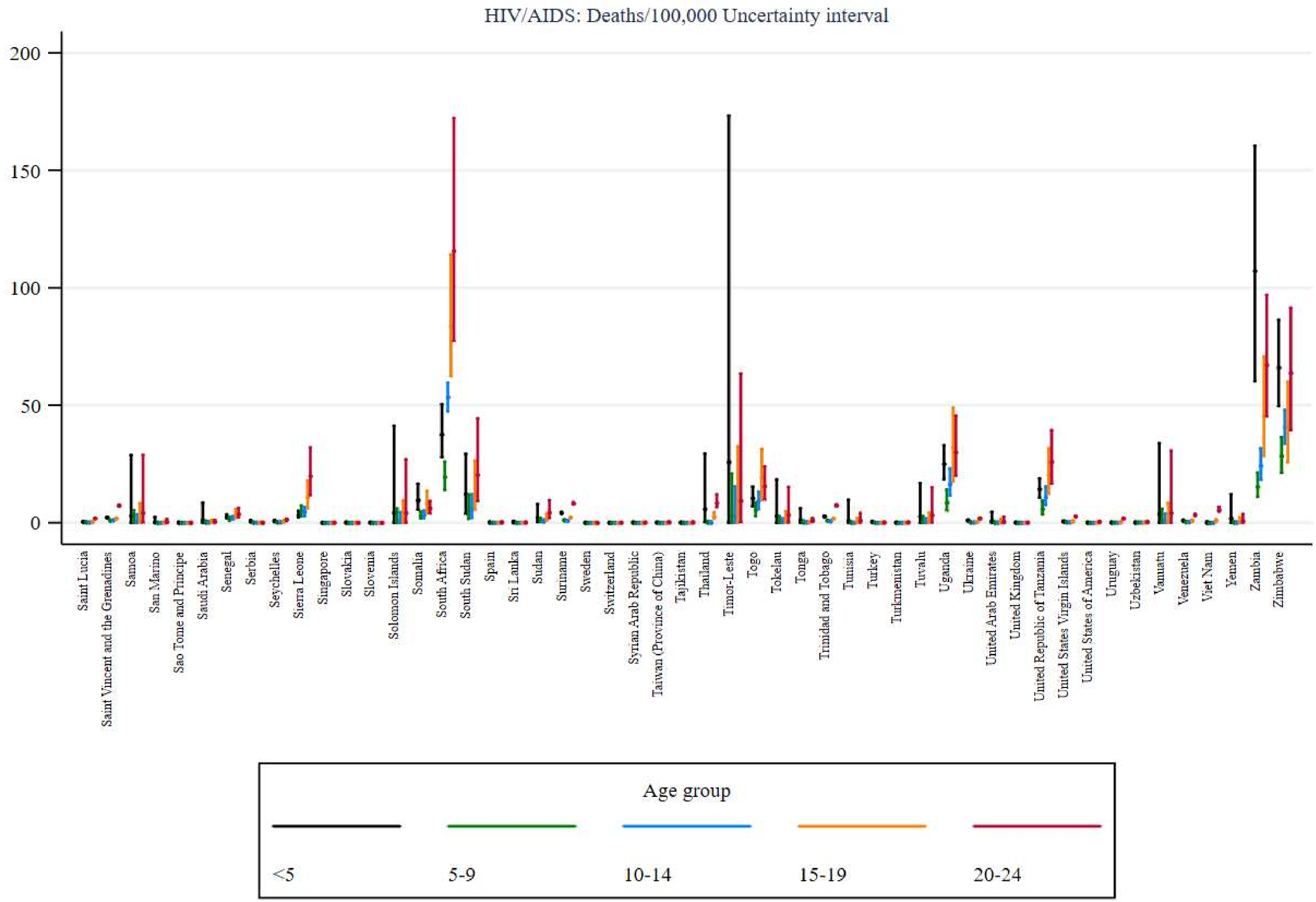
HIV/AIDS: Deaths/100,000 Uncertainty interval
200
150
100
50
0
Saint Lucia
Saint Vincent and the Grenadines
Samoa
San Marino
Sao Tome and Principe
Saudi Arabia
Senegal
Serbia
Seychelles
Sierra Leone
Singapore
Slovakia
Slovenia
Solomon Islands
Somalia
South Africa
South Sudan
Spain
Sri Lanka
Sudan
Suriname
Sweden
Switzerland
Syrian Arab Republic
Taiwan (Province of China)
Tajikistan
Thailand
Timor-Leste
Togo
Tokelau
Tonga
Trinidad and Tobago
Tunisia
Turkey
Turkmenistan
Tuvalu
Uganda
Ukraine
United Arab Emirates
United Kingdom
United Republic of Tanzania
United States Virgin Islands
United States of America
Uruguay
Uzbekistan
Vanuatu
Venezuela
Viet Nam
Yemen
Zambia
Zimbabwe
Age group
<5
5-9
10-14
15-19
20-24

S20_2 Part B: HIV DALYs/ 100 000 Uncertainty interval for each age group

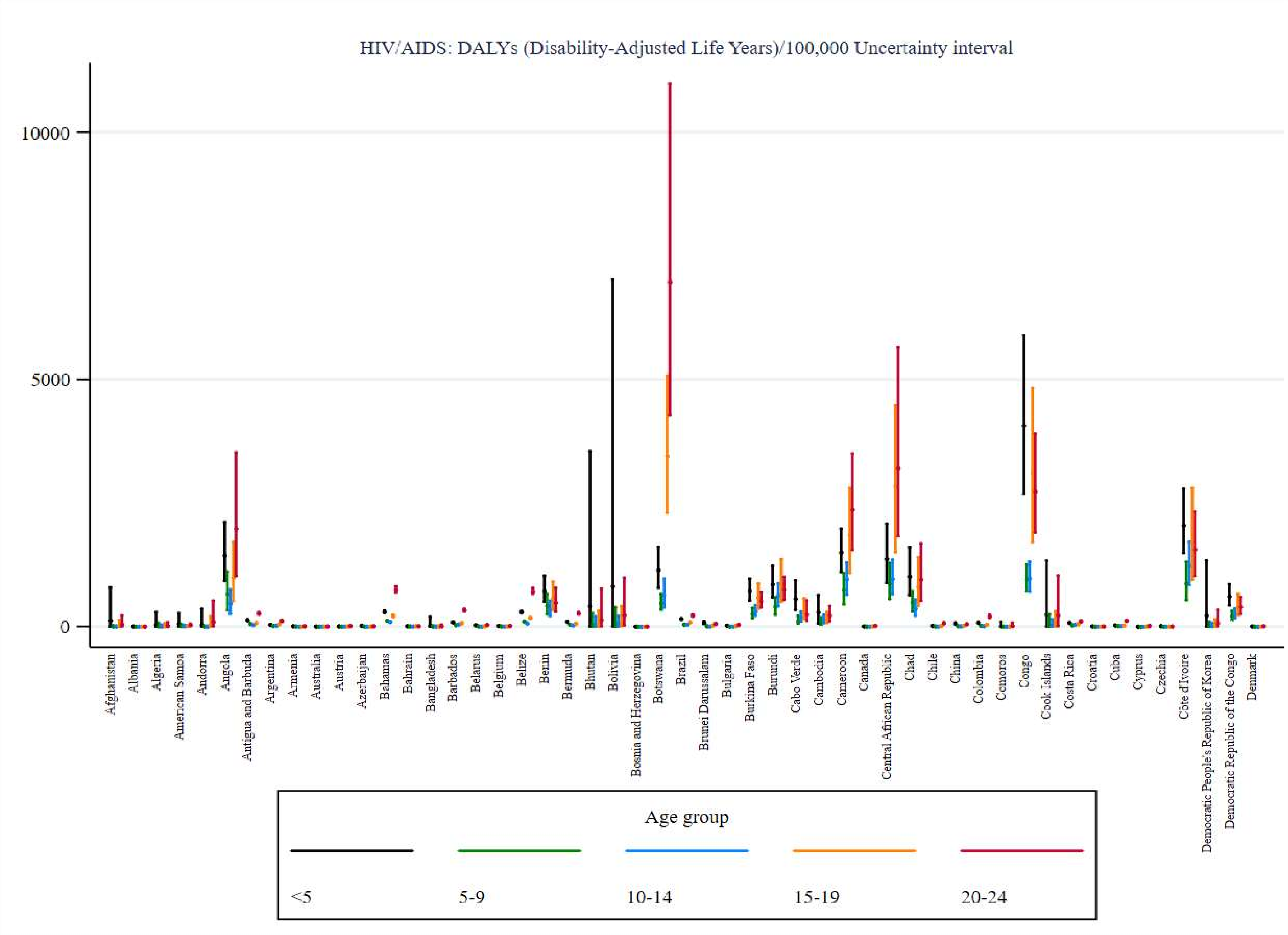

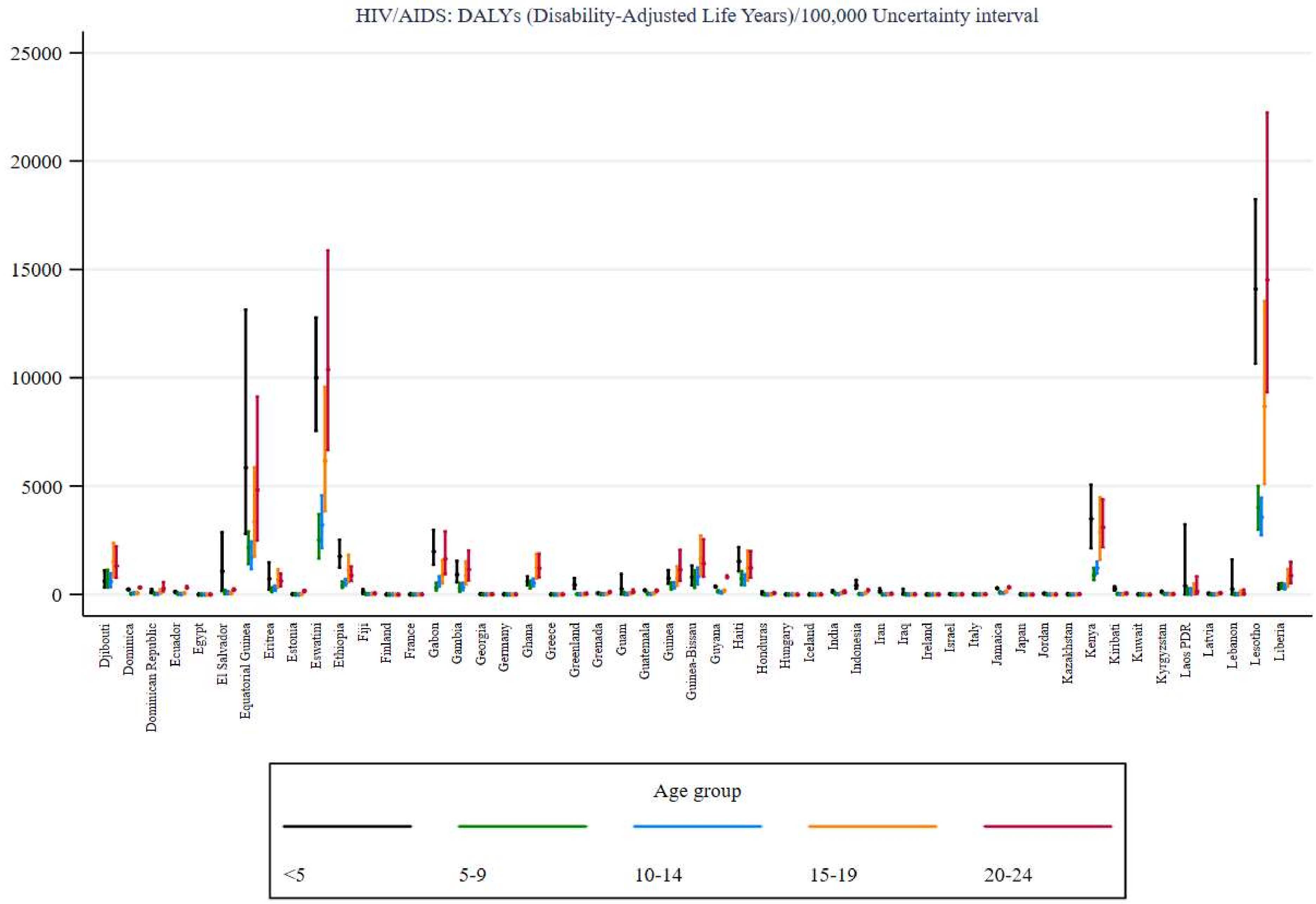

HIV/AIDS: DALYs (Disability-Adjusted Life Years)/100,000 Uncertainty interval
25000
20000
15000
10000
5000
0
Djibouti
Dominica
Dominican Republic
Ecuador
Egypt
El Salvador
Equatorial Guinea
Eritrea
Estonia
Eswatini
Ethiopia
Fiji
Finland
France
Gabon
Gambia
Georgia
Germany
Ghana
Greece
Greenland
Grenada
Guam
Guatemala
Guinea
Guinea-Bissau
Guyana
Haiti
Honduras
Hungary
Iceland
India
Indonesia
Iran
Iraq
Ireland
Israel
Italy
Jamaica
Japan
Jordan
Kazakhstan
Kenya
Kiribati
Kuwait
Kyrgyzstan
Laos PDR
Latvia
Lebanon
Lesotho
Liberia
Age group
<5
5-9
10-14
15-19
20-24

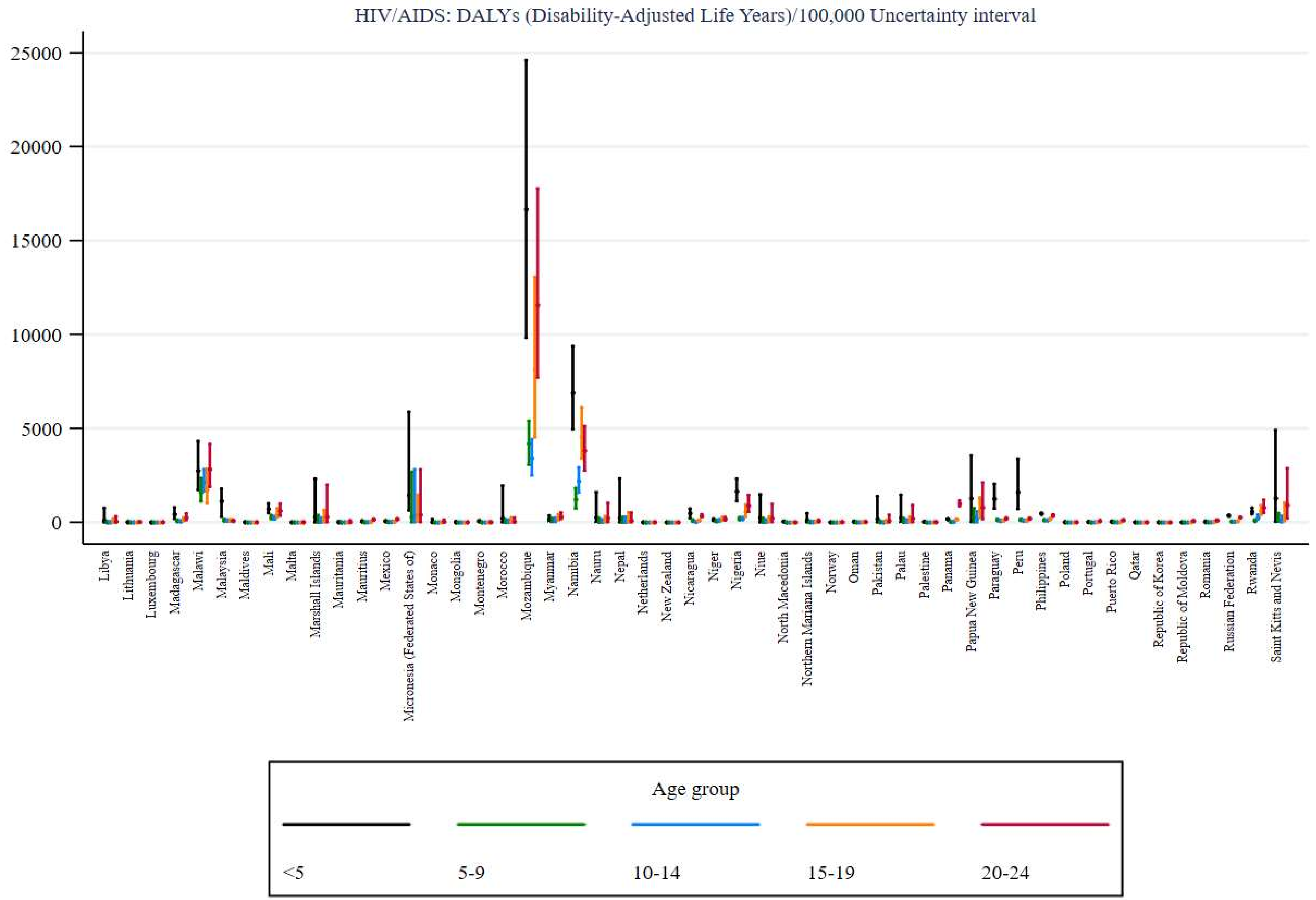

HIV/AIDS: DALYs (Disability-Adjusted Life Years)/100,000 Uncertainty interval
25000
20000
15000
10000
5000
0
Libya
Lithuania
Luxembourg
Madagascar
Malawi
Malaysia
Maldives
Mali
Malta
Marshall Islands
Mauritania
Mauritius
Mexico
Micronesia (Federated States of)
Monaco
Mongolia
Montenegro
Morocco
Mozambique
Myanmar
Namibia
Nauru
Nepal
Netherlands
New Zealand
Nicaragua
Niger
Nigeria
Niue
North Macedonia
Northern Mariana Islands
Norway
Oman
Pakistan
Palau
Palestine
Panama
Papua New Guinea
Paraguay
Peru
Philippines
Poland
Portugal
Puerto Rico
Qatar
Republic of Korea
Republic of Moldova
Romania
Russian Federation
Rwanda
Saint Kitts and Nevis
Age group
<5
5-9
10-14
15-19
20-24

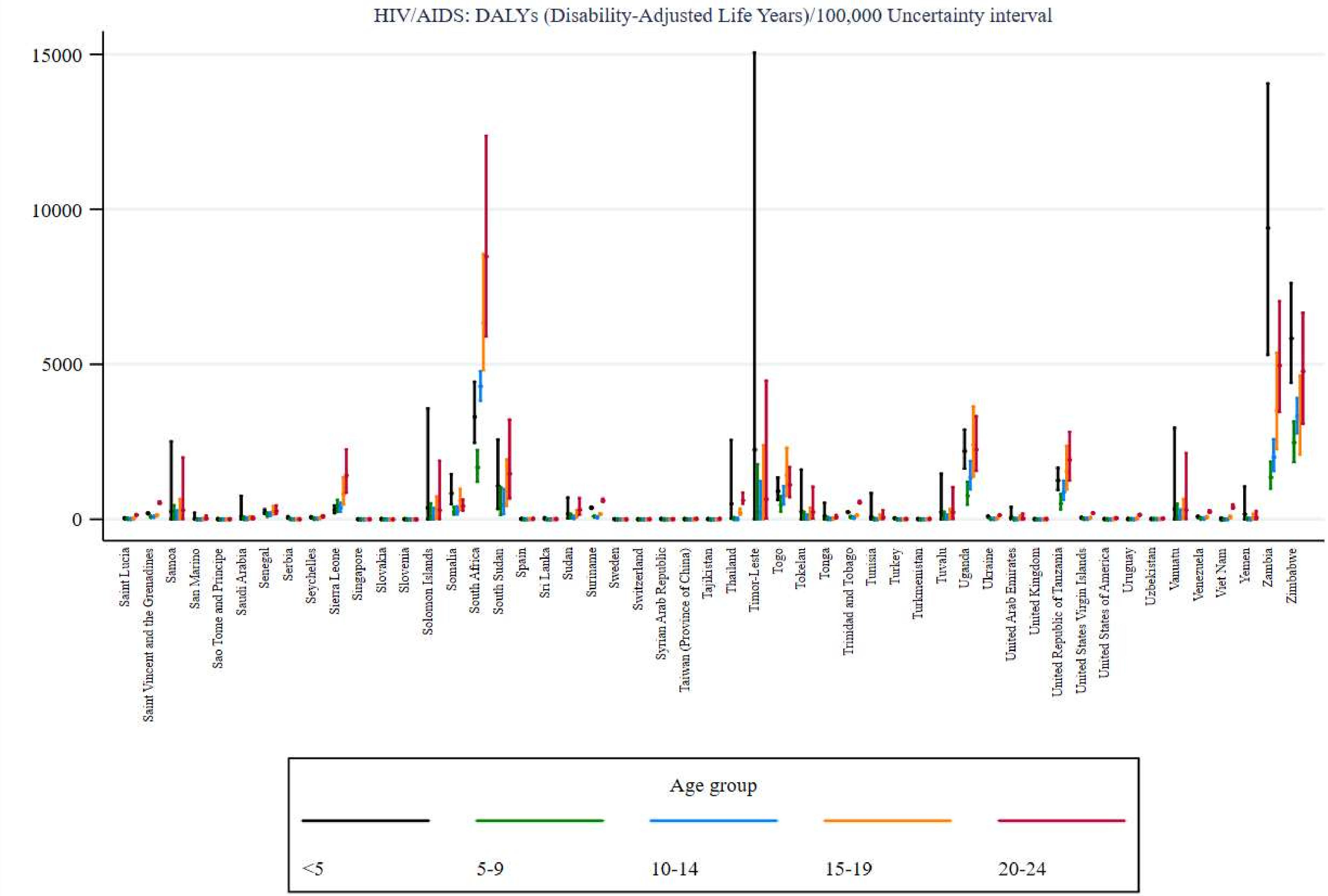

HIV/AIDS: DALYs (Disability-Adjusted Life Years)/100,000 Uncertainty interval
15000
10000
5000
0
Saint Lucia
Saint Vincent and the Grenadines
Samoa
San Marino
Sao Tome and Principe
Saudi Arabia
Senegal
Serbia
Seychelles
Sierra Leone
Singapore
Slovakia
Slovenia
Solomon Islands
Somalia
South Africa
South Sudan
Spain
Sri Lanka
Sudan
Suriname
Sweden
Switzerland
Syrian Arab Republic
Taiwan (Province of China)
Tajikistan
Thailand
Timor-Leste
Togo
Tokelau
Tonga
Trinidad and Tobago
Tunisia
Turkey
Turkmenistan
Tuvalu
Uganda
Ukraine
United Arab Emirates
United Kingdom
United Republic of Tanzania
United States Virgin Islands
United States of America
Uruguay
Uzbekistan
Vanuatu
Venezuela
Viet Nam
Yemen
Zambia
Zimbabwe
Age group
<5
5-9
10-14
15-19
20-24

S20_3 Part A: Lower respiratory infections Death/ 100 000 Uncertainty interval for each age group

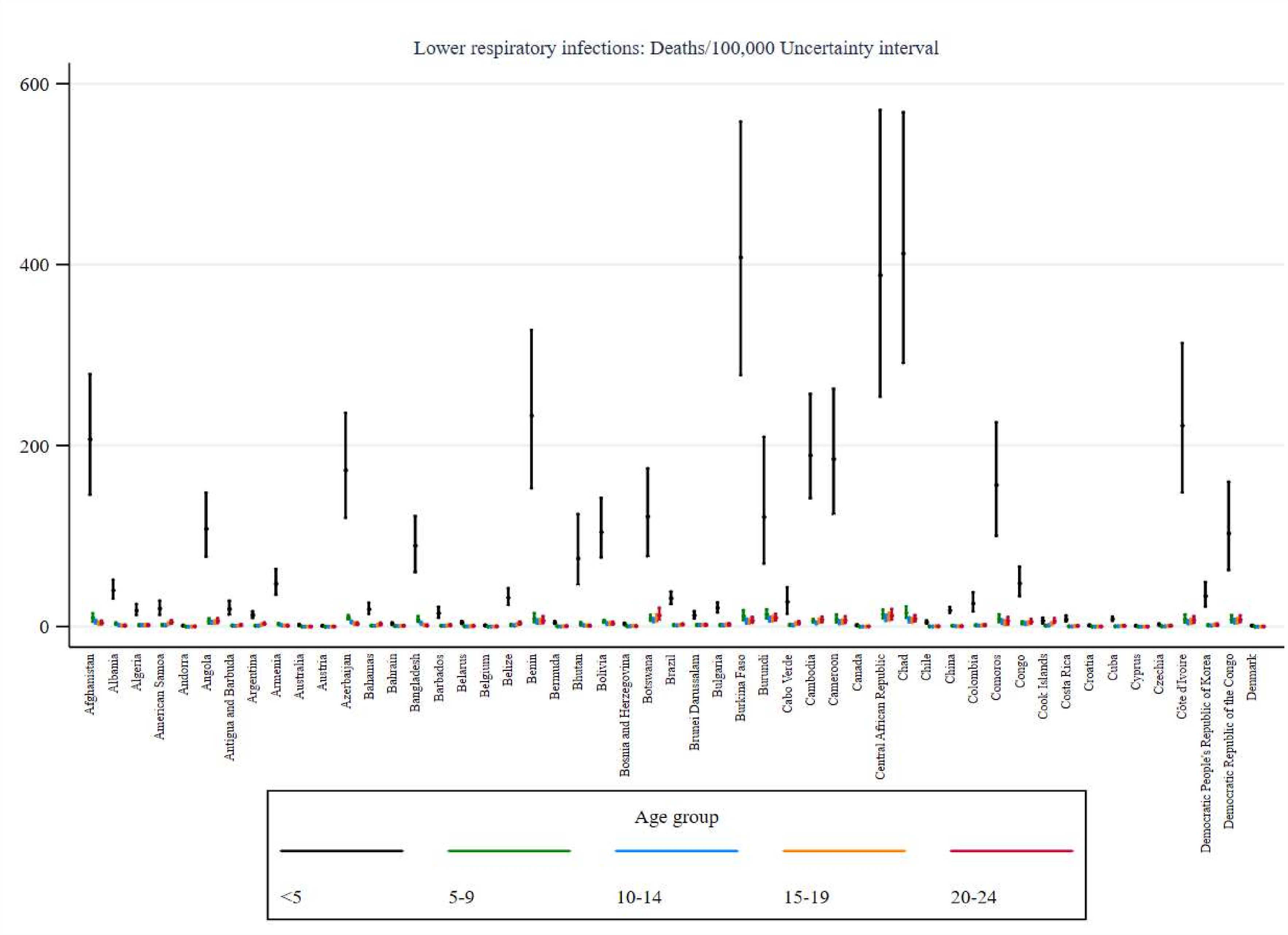

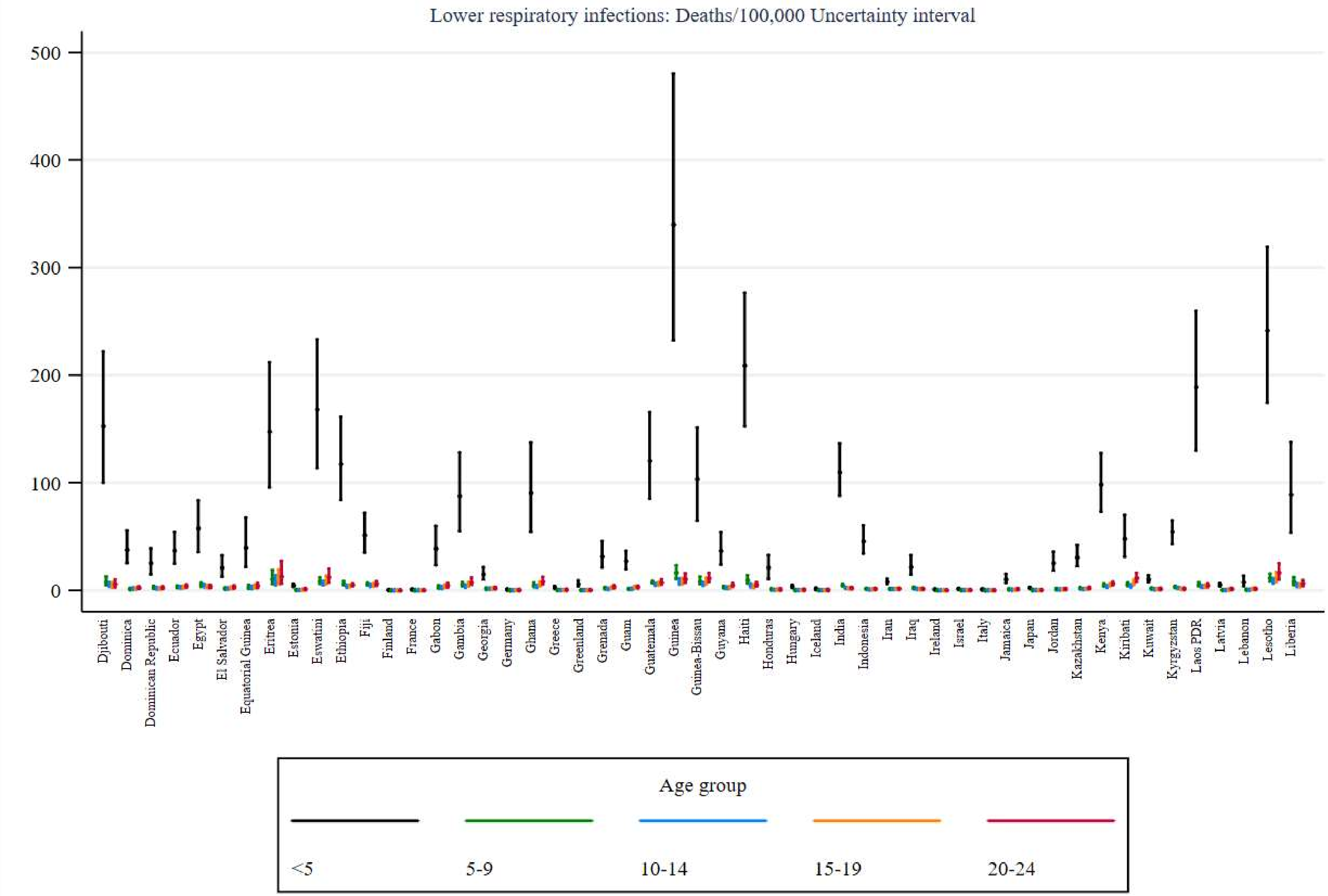
Lower respiratory infections: Deaths/100,000 Uncertainty interval
500
400
300
200
100
0
Djibouti
Dominica
Dominican Republic
Ecuador
Egypt
El Salvador
Equatorial Guinea
Eritrea
Estonia
Eswatini
Ethiopia
Fiji
Finland
France
Gabon
Gambia
Georgia
Germany
Ghana
Greece
Greenland
Grenada
Guam
Guatemala
Guinea
Guinea-Bissau
Guyana
Haiti
Honduras
Hungary
Iceland
India
Indonesia
Iran
Iraq
Ireland
Israel
Italy
Jamaica
Japan
Jordan
Kazakhstan
Kenya
Kiribati
Kuwait
Kyrgyzstan
Laos PDR
Latvia
Lebanon
Lesotho
Liberia
Age group
<5
5-9
10-14
15-19
20-24

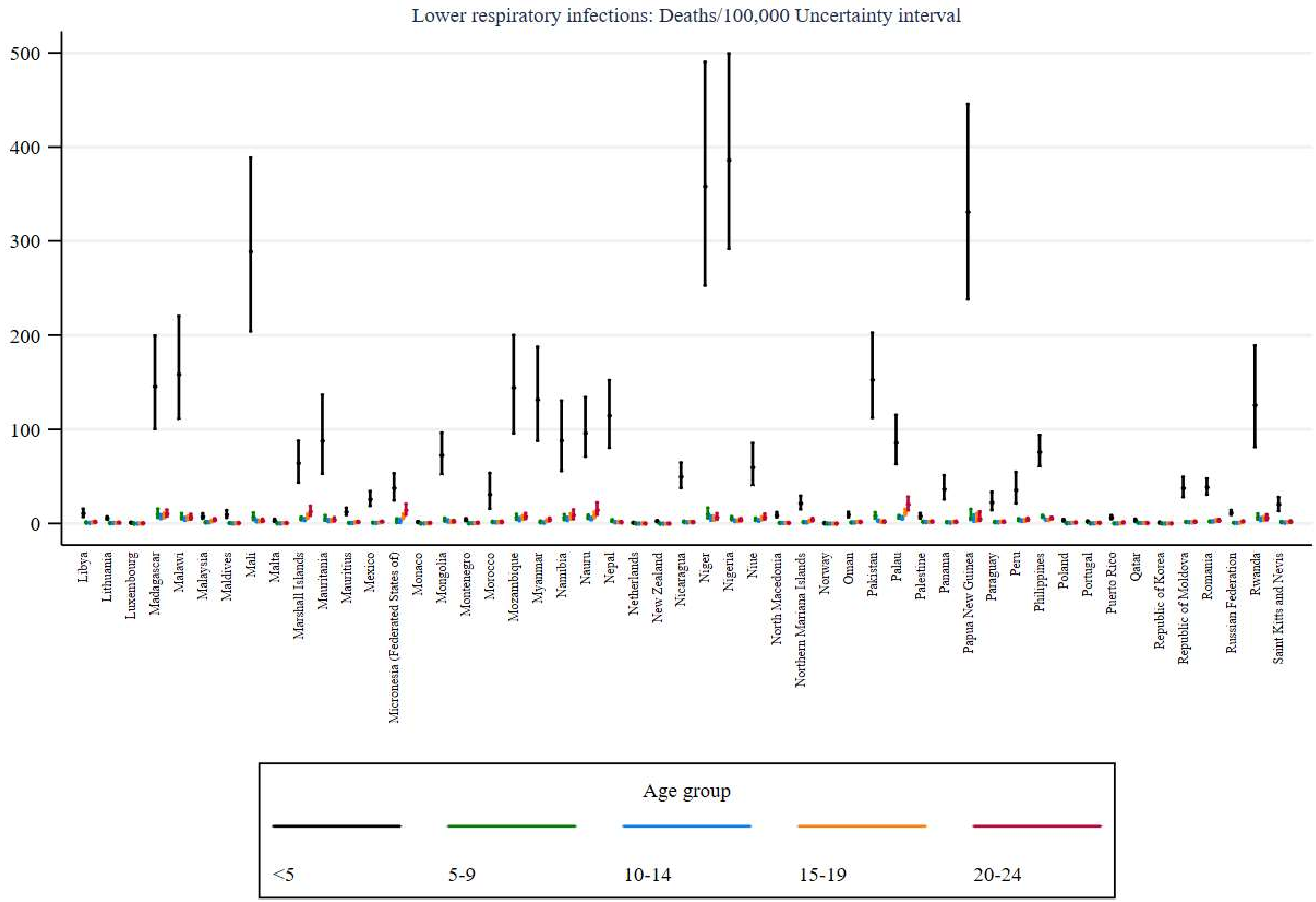

Lower respiratory infections: Deaths/100,000 Uncertainty interval
500
400
300
200
100
0
Libya
Lithuania
Luxembourg
Madagascar
Malawi
Malaysia
Maldives
Mali
Malta
Marshall Islands
Mauritania
Mauritius
Mexico
Micronesia (Federated States of)
Monaco
Mongolia
Montenegro
Morocco
Mozambique
Myanmar
Namibia
Nauru
Nepal
Netherlands
New Zealand
Nicaragua
Niger
Nigeria
Niue
North Macedonia
Northern Mariana Islands
Norway
Oman
Pakistan
Palau
Palestine
Panama
Papua New Guinea
Paraguay
Peru
Philippines
Poland
Portugal
Puerto Rico
Qatar
Republic of Korea
Republic of Moldova
Romania
Russian Federation
Rwanda
Saint Kitts and Nevis
Age group
<5
5-9
10-14
15-19
20-24

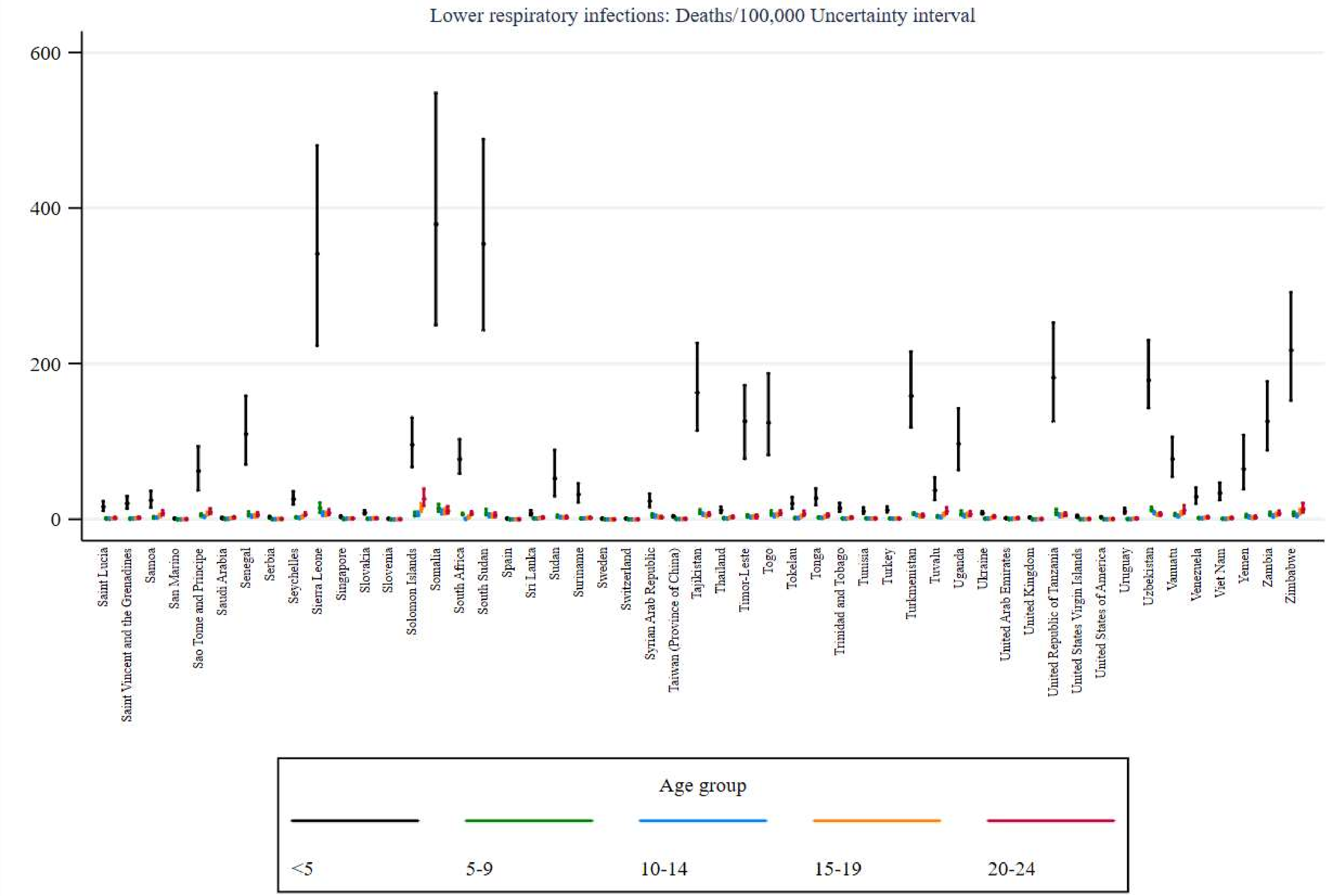
Lower respiratory infections: Deaths/100,000 Uncertainty interval
600
400
200
0
Saint Lucia
Saint Vincent and the Grenadines
Samoa
San Marino
Sao Tome and Principe
Saudi Arabia
Senegal
Serbia
Seychelles
Sierra Leone
Singapore
Slovakia
Slovenia
Solomon Islands
Somalia
South Africa
South Sudan
Spain
Sri Lanka
Sudan
Suriname
Sweden
Switzerland
Syrian Arab Republic
Taiwan (Province of China)
Tajikistan
Thailand
Timor-Leste
Togo
Tokelau
Tonga
Trinidad and Tobago
Tunisia
Turkey
Turkmenistan
Tuvalu
Uganda
Ukraine
United Arab Emirates
United Kingdom
United Republic of Tanzania
United States Virgin Islands
United States of America
Uruguay
Uzbekistan
Vanuatu
Venezuela
Viet Nam
Yemen
Zambia
Zimbabwe
Age group
<5
5-9
10-14
15-19
20-24

S20_4 Part B: Lower respiratory infections DALYs/ 100 000 Uncertainty interval for each age group

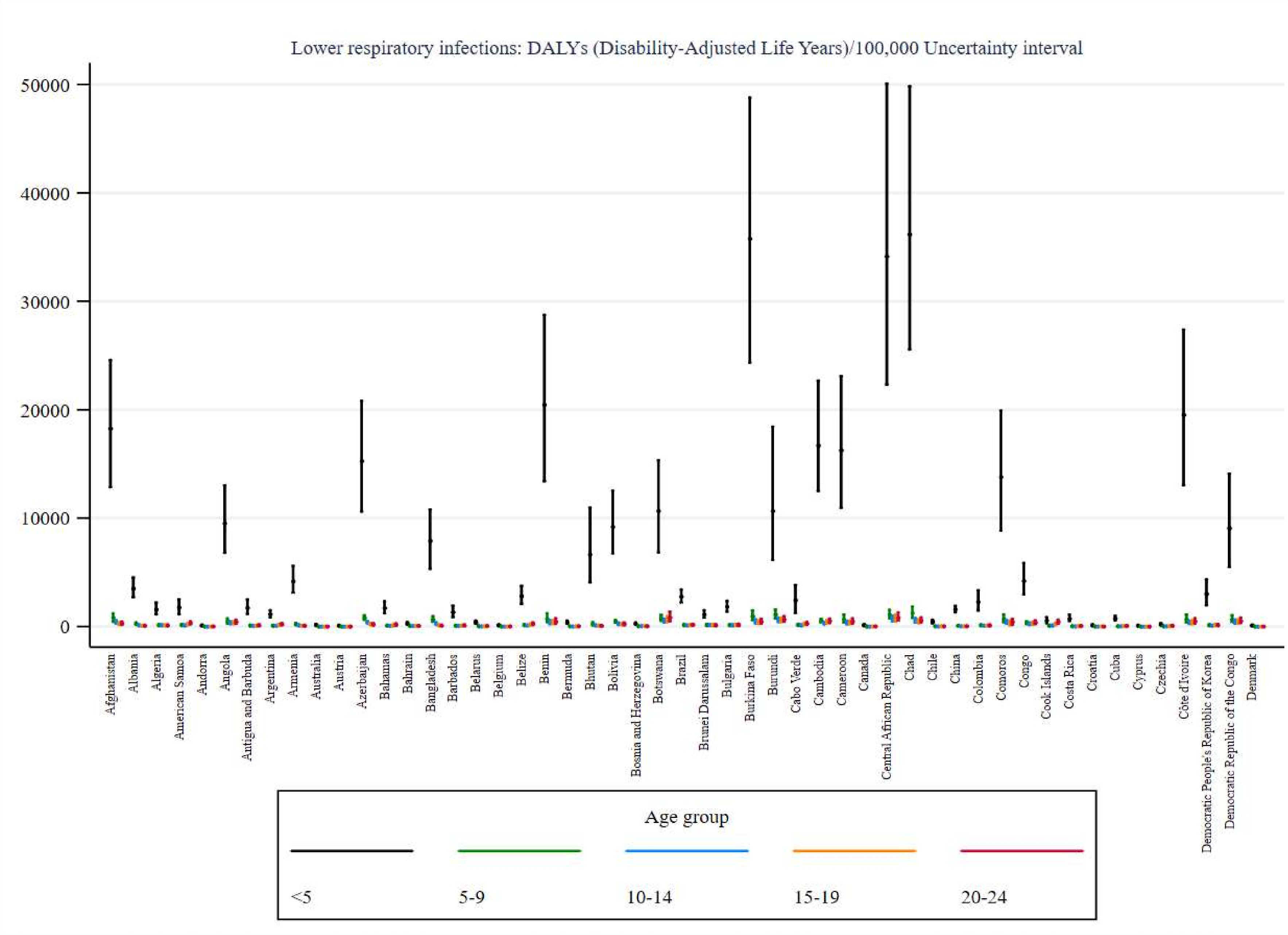

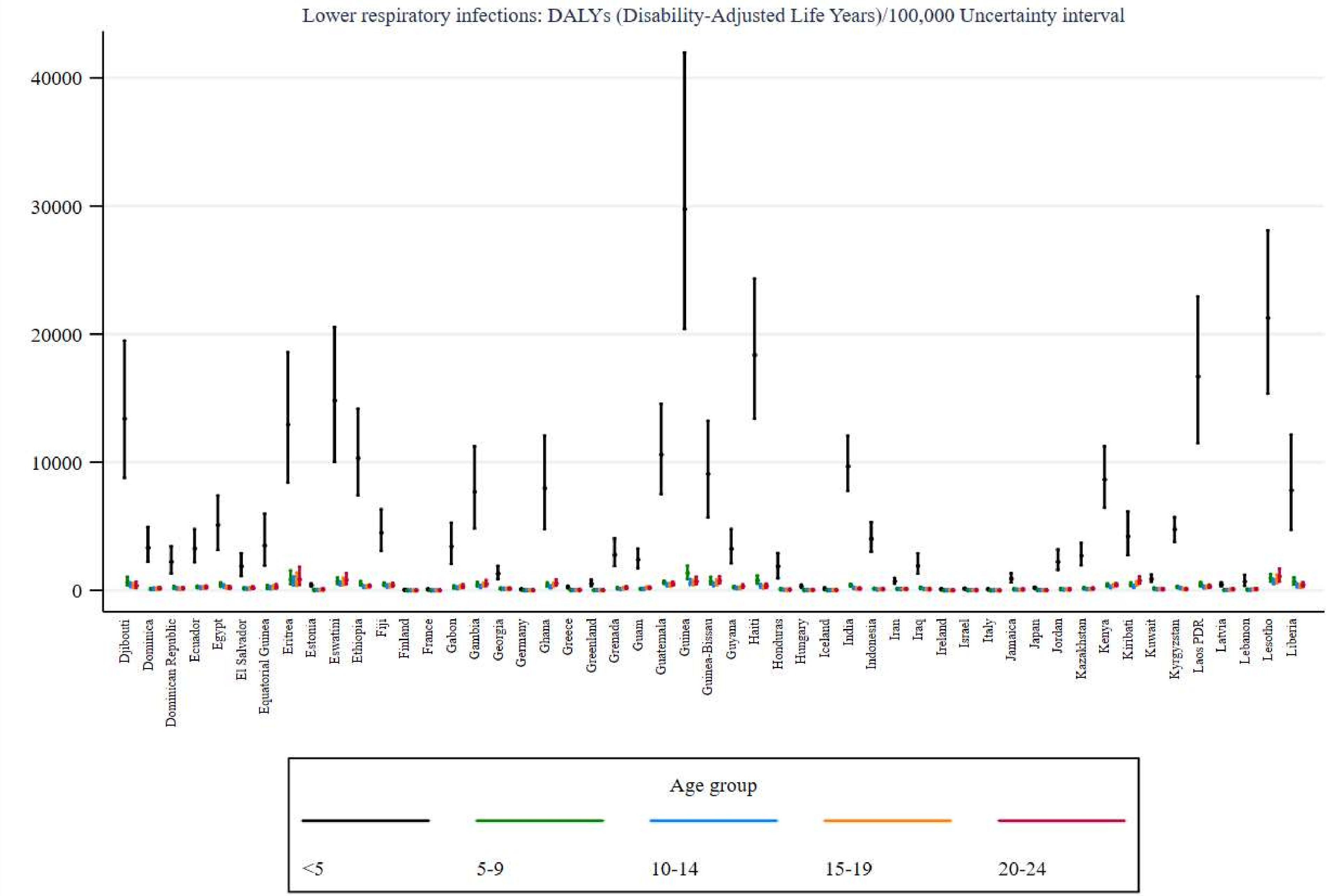

Lower respiratory infections: DALYs (Disability-Adjusted Life Years)/100,000 Uncertainty interval
40000
30000
20000
10000
0
Djibouti
Dominica
Dominican Republic
Ecuador
Egypt
El Salvador
Equatorial Guinea
Eritrea
Estonia
Eswatini
Ethiopia
Fiji
Finland
France
Gabon
Gambia
Georgia
Germany
Ghana
Greece
Greenland
Grenada
Guam
Guatemala
Guinea
Guinea-Bissau
Guyana
Haiti
Honduras
Hungary
Iceland
India
Indonesia
Iran
Iraq
Ireland
Israel
Italy
Jamaica
Japan
Jordan
Kazakhstan
Kenya
Kiribati
Kuwait
Kyrgyzstan
Laos PDR
Latvia
Lebanon
Lesotho
Liberia
Age group
<5
5-9
10-14
15-19
20-24

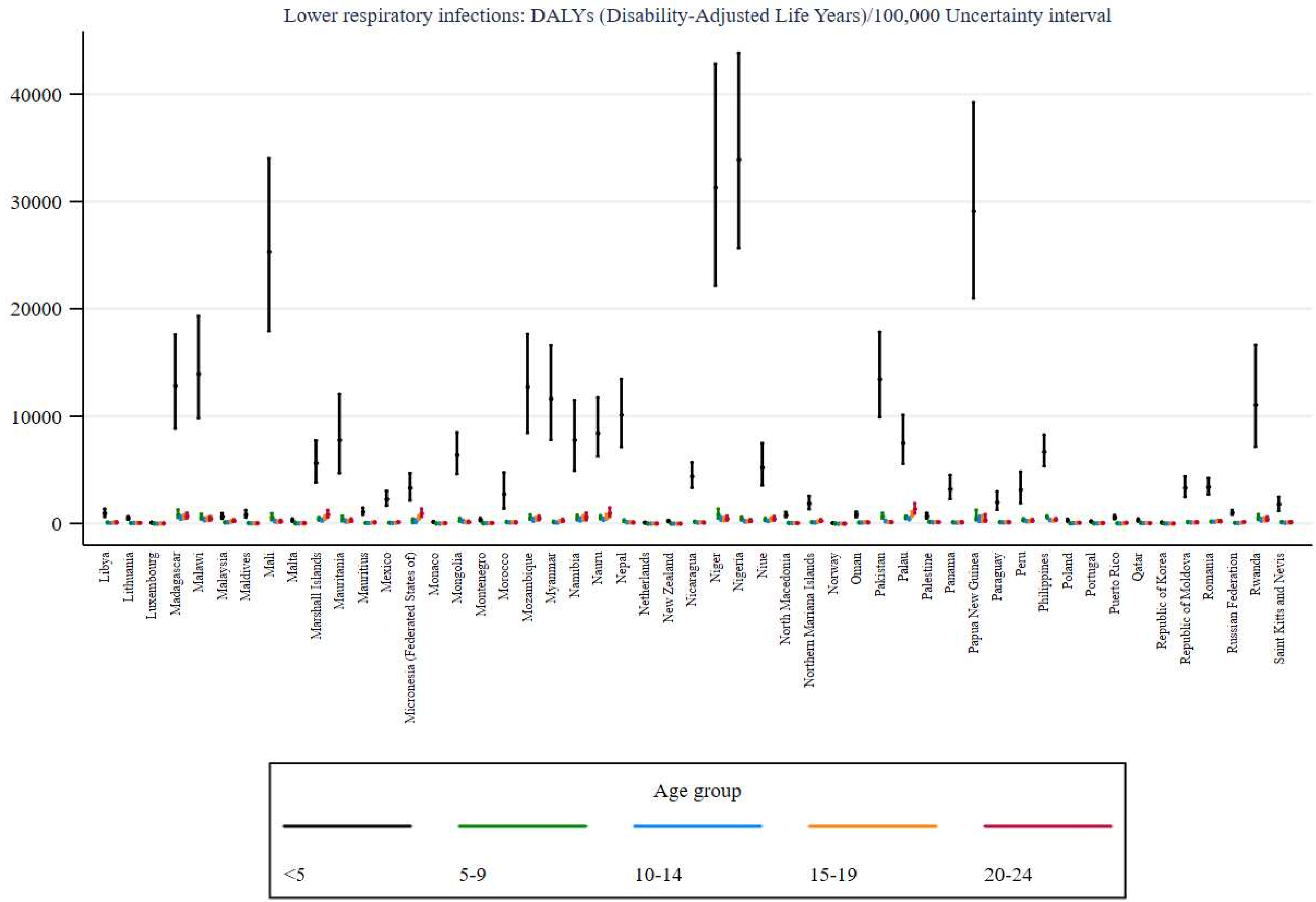

Lower respiratory infections: DALYs (Disability-Adjusted Life Years)/100,000 Uncertainty interval
40000
30000
20000
10000
0
Libya
Lithuania
Luxembourg
Madagascar
Malawi
Malaysia
Maldives
Mali
Malta
Marshall Islands
Mauritania
Mauritius
Mexico
Micronesia (Federated States of)
Monaco
Mongolia
Montenegro
Morocco
Mozambique
Myanmar
Namibia
Nauru
Nepal
Netherlands
New Zealand
Nicaragua
Niger
Nigeria
Niue
North Macedonia
Northern Mariana Islands
Norway
Oman
Pakistan
Palau
Palestine
Panama
Papua New Guinea
Paraguay
Peru
Philippines
Poland
Portugal
Puerto Rico
Qatar
Republic of Korea
Republic of Moldova
Romania
Russian Federation
Rwanda
Saint Kitts and Nevis
Age group
<5
5-9
10-14
15-19
20-24

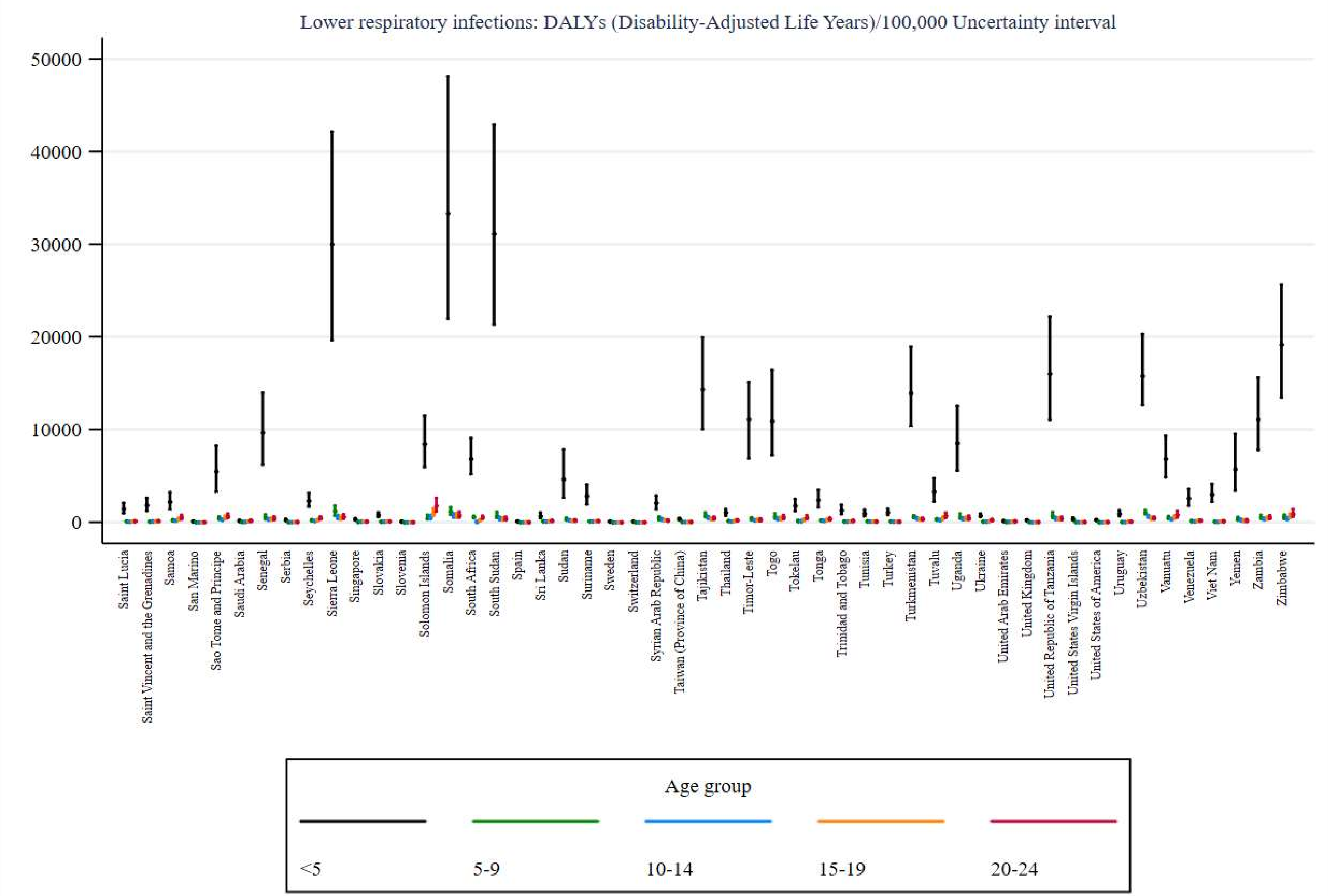
Lower respiratory infections: DALYs (Disability-Adjusted Life Years)/100,000 Uncertainty interval
50000
40000
30000
20000
10000
0
Saint Lucia
Saint Vincent and the Grenadines
Samoa
San Marino
Sao Tome and Principe
Saudi Arabia
Senegal
Serbia
Seychelles
Sierra Leone
Singapore
Slovakia
Slovenia
Solomon Islands
Somalia
South Africa
South Sudan
Spain
Sri Lanka
Sudan
Suriname
Sweden
Switzerland
Syrian Arab Republic
Taiwan (Province of China)
Tajikistan
Thailand
Timor-Leste
Togo
Tokelau
Tonga
Trinidad and Tobago
Tunisia
Turkey
Turkmenistan
Tuvalu
Uganda
Ukraine
United Arab Emirates
United Kingdom
United Republic of Tanzania
United States Virgin Islands
United States of America
Uruguay
Uzbekistan
Vanuatu
Venezuela
Viet Nam
Yemen
Zambia
Zimbabwe
Age group
<5
5-9
10-14
15-19
20-24

S20_4 Part A: Malaria Deaths/ 100 000 Uncertainty interval for each age group

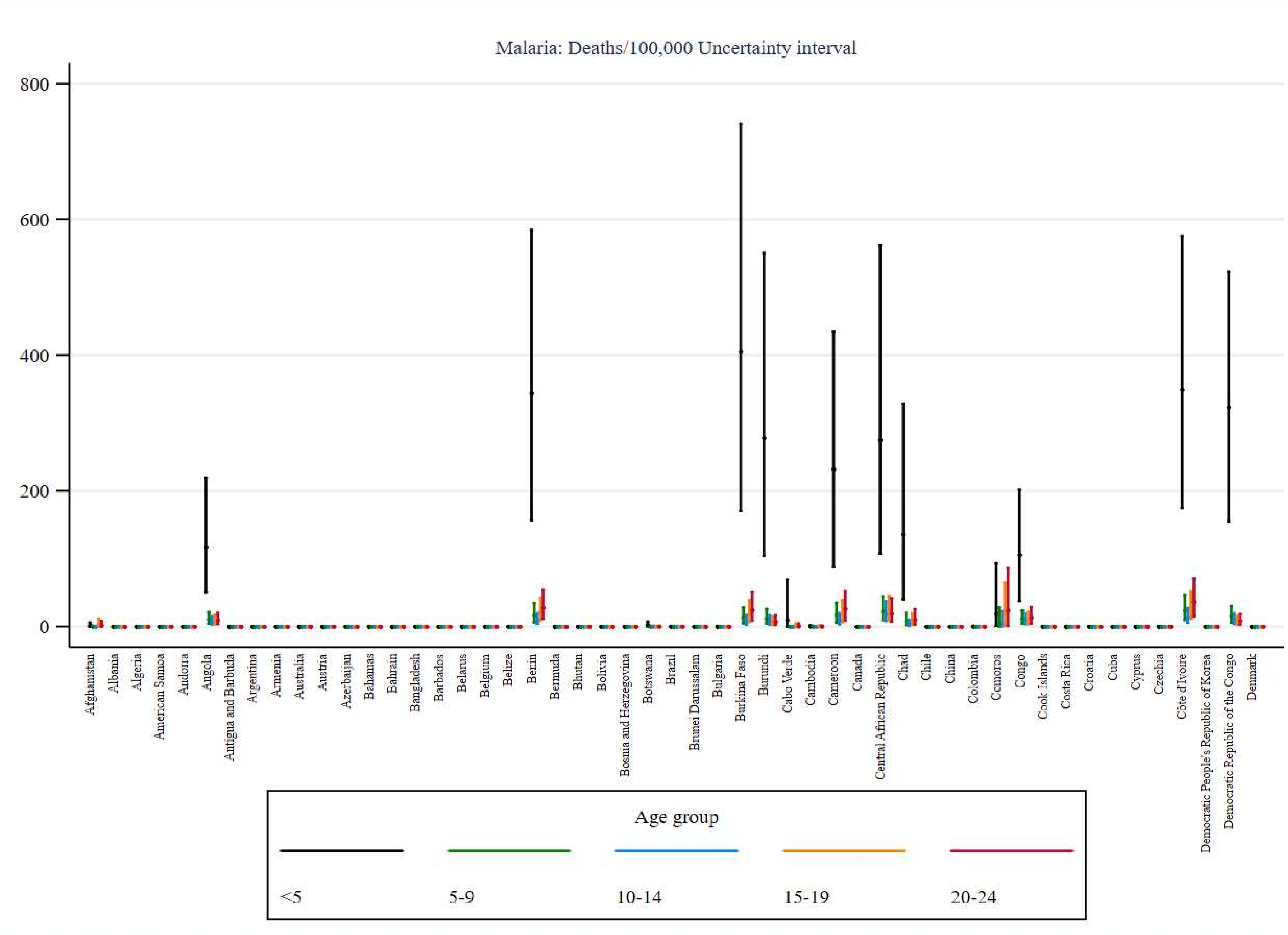

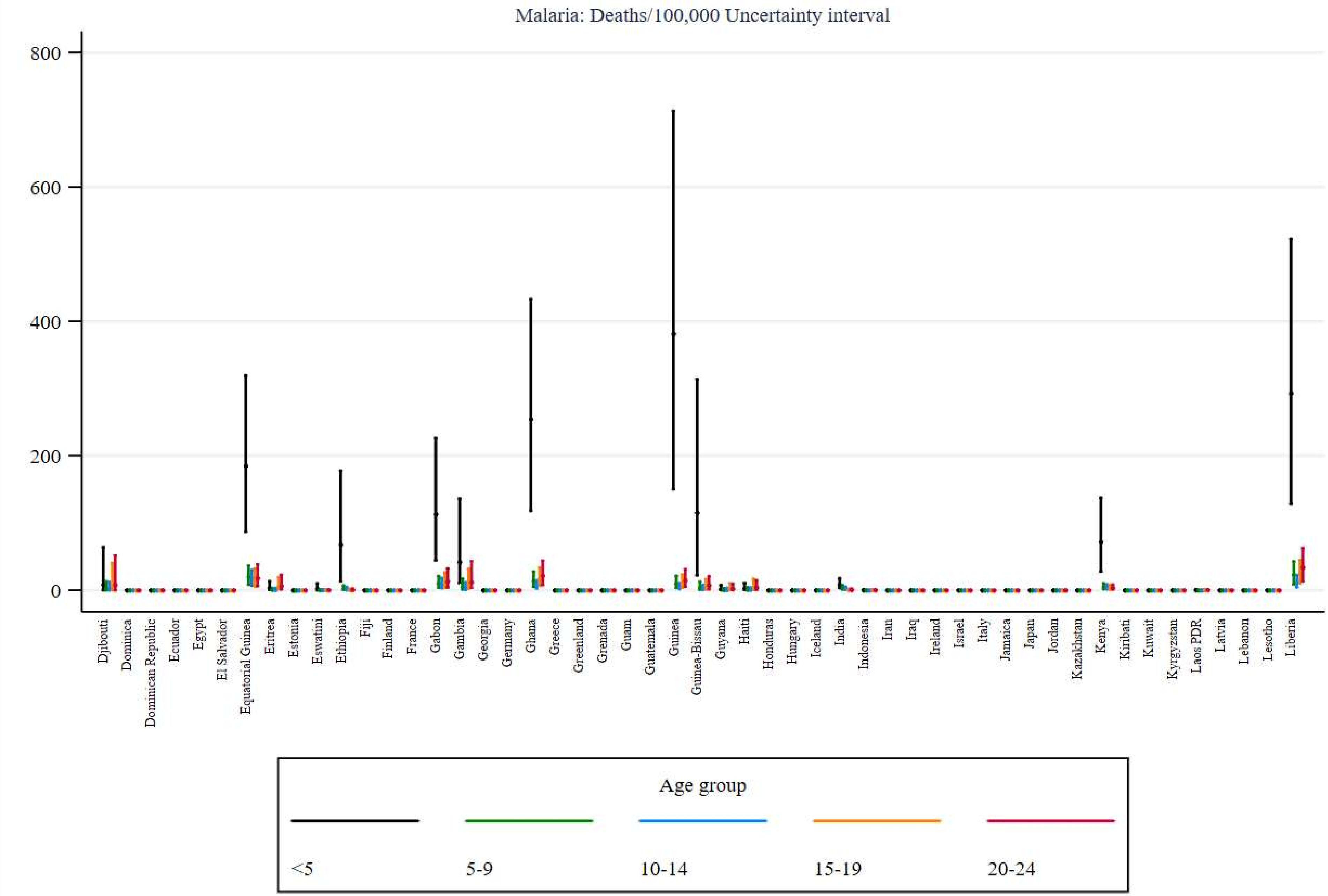

Malaria: Deaths/100,000 Uncertainty interval
800
600
400
200
0
Djibouti
Dominica
Dominican Republic
Ecuador
Egypt
El Salvador
Equatorial Guinea
Eritrea
Estonia
Eswatini
Ethiopia
Fiji
Finland
France
Gabon
Gambia
Georgia
Germany
Ghana
Greece
Greenland
Grenada
Guam
Guatemala
Guinea
Guinea-Bissau
Guyana
Haiti
Honduras
Hungary
Iceland
India
Indonesia
Iran
Iraq
Ireland
Israel
Italy
Jamaica
Japan
Jordan
Kazakhstan
Kenya
Kiribati
Kuwait
Kyrgyzstan
Laos PDR
Latvia
Lebanon
Lesotho
Liberia
Age group
<5
5-9
10-14
15-19
20-24

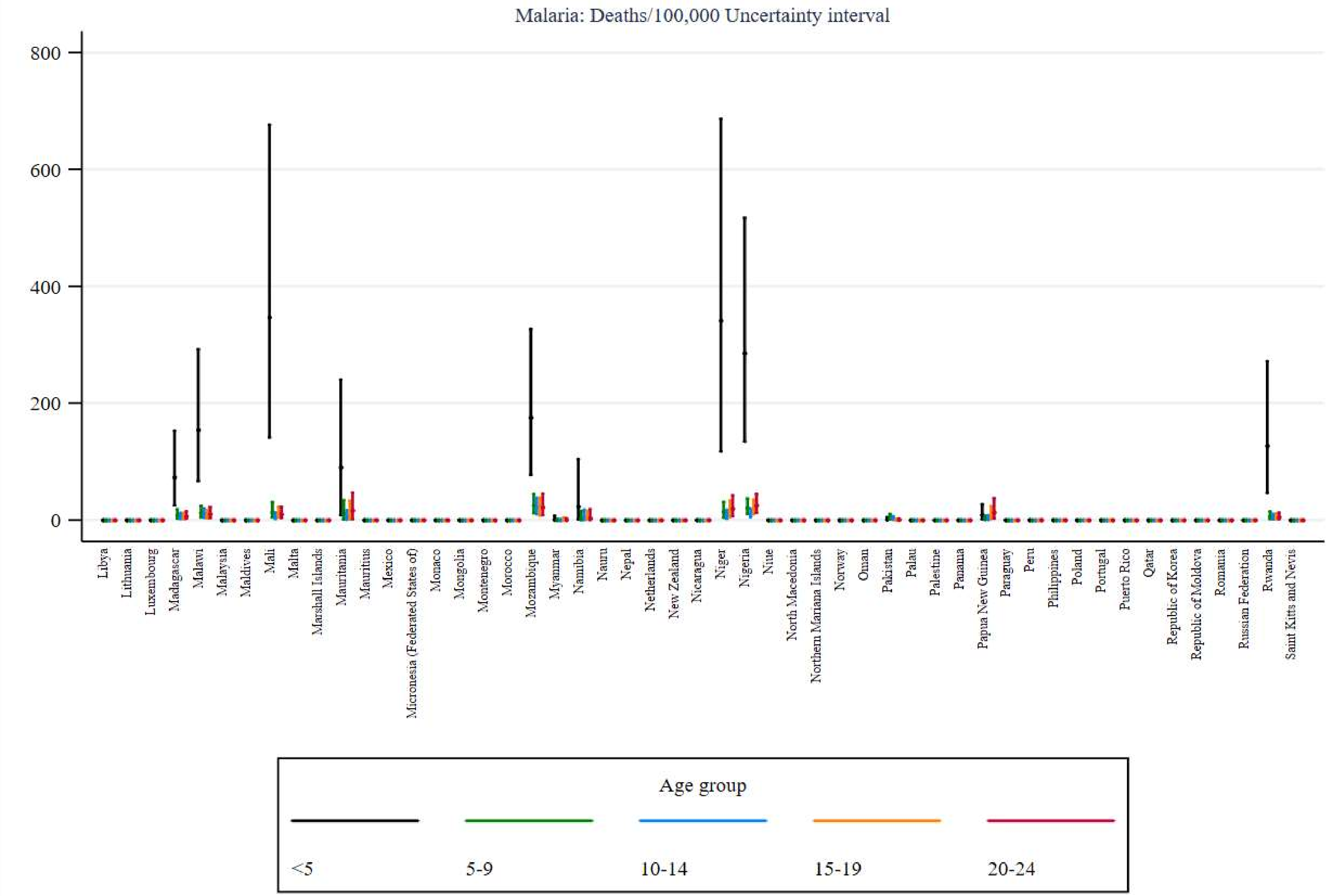
Malaria: Deaths/100,000 Uncertainty interval
800
600
400
200
0
Libya
Lithuania
Luxembourg
Madagascar
Malawi
Malaysia
Maldives
Mali
Malta
Marshall Islands
Mauritania
Mauritius
Mexico
Micronesia (Federated States of)
Monaco
Mongolia
Montenegro
Morocco
Mozambique
Myanmar
Namibia
Nauru
Nepal
Netherlands
New Zealand
Nicaragua
Niger
Nigeria
Niue
North Macedonia
Northern Mariana Islands
Norway
Oman
Pakistan
Palau
Palestine
Panama
Papua New Guinea
Paraguay
Peru
Philippines
Poland
Portugal
Puerto Rico
Qatar
Republic of Korea
Republic of Moldova
Romania
Russian Federation
Rwanda
Saint Kitts and Nevis
Age group
<5
5-9
10-14
15-19
20-24

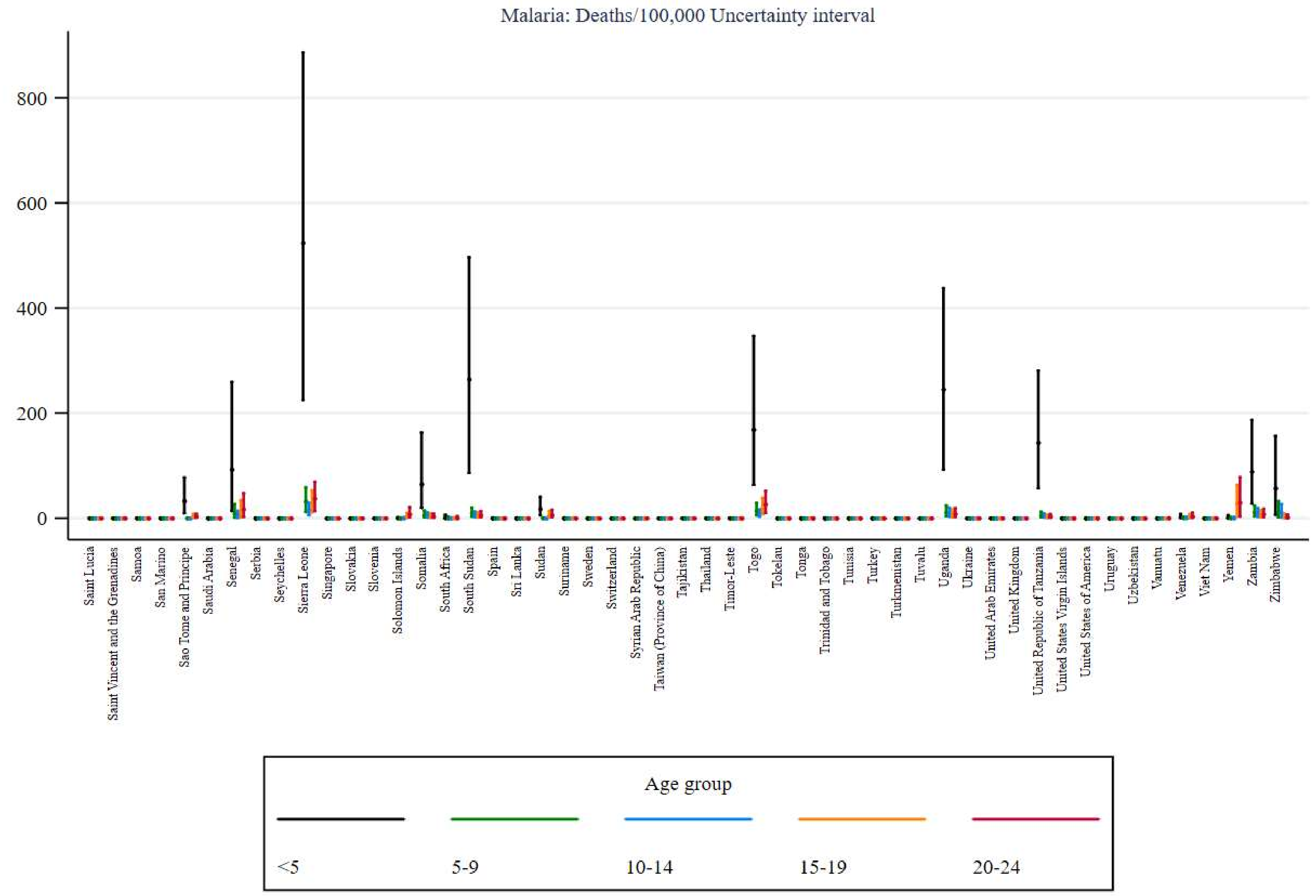

Malaria: Deaths/100,000 Uncertainty interval
800
600
400
200
0
Saint Lucia
Saint Vincent and the Grenadines
Samoa
San Marino
Sao Tome and Principe
Saudi Arabia
Senegal
Serbia
Seychelles
Sierra Leone
Singapore
Slovakia
Slovenia
Solomon Islands
Somalia
South Africa
South Sudan
Spain
Sri Lanka
Sudan
Suriname
Sweden
Switzerland
Syrian Arab Republic
Taiwan (Province of China)
Tajikistan
Thailand
Timor-Leste
Togo
Tokelau
Tonga
Trinidad and Tobago
Tunisia
Turkey
Turkmenistan
Tuvalu
Uganda
Ukraine
United Arab Emirates
United Kingdom
United Republic of Tanzania
United States Virgin Islands
United States of America
Uruguay
Uzbekistan
Vanuatu
Venezuela
Viet Nam
Yemen
Zambia
Zimbabwe
Age group
<5
5-9
10-14
15-19
20-24

S20_4 Part B: Malaria DALYs/ 100 000 Uncertainty interval for each age group

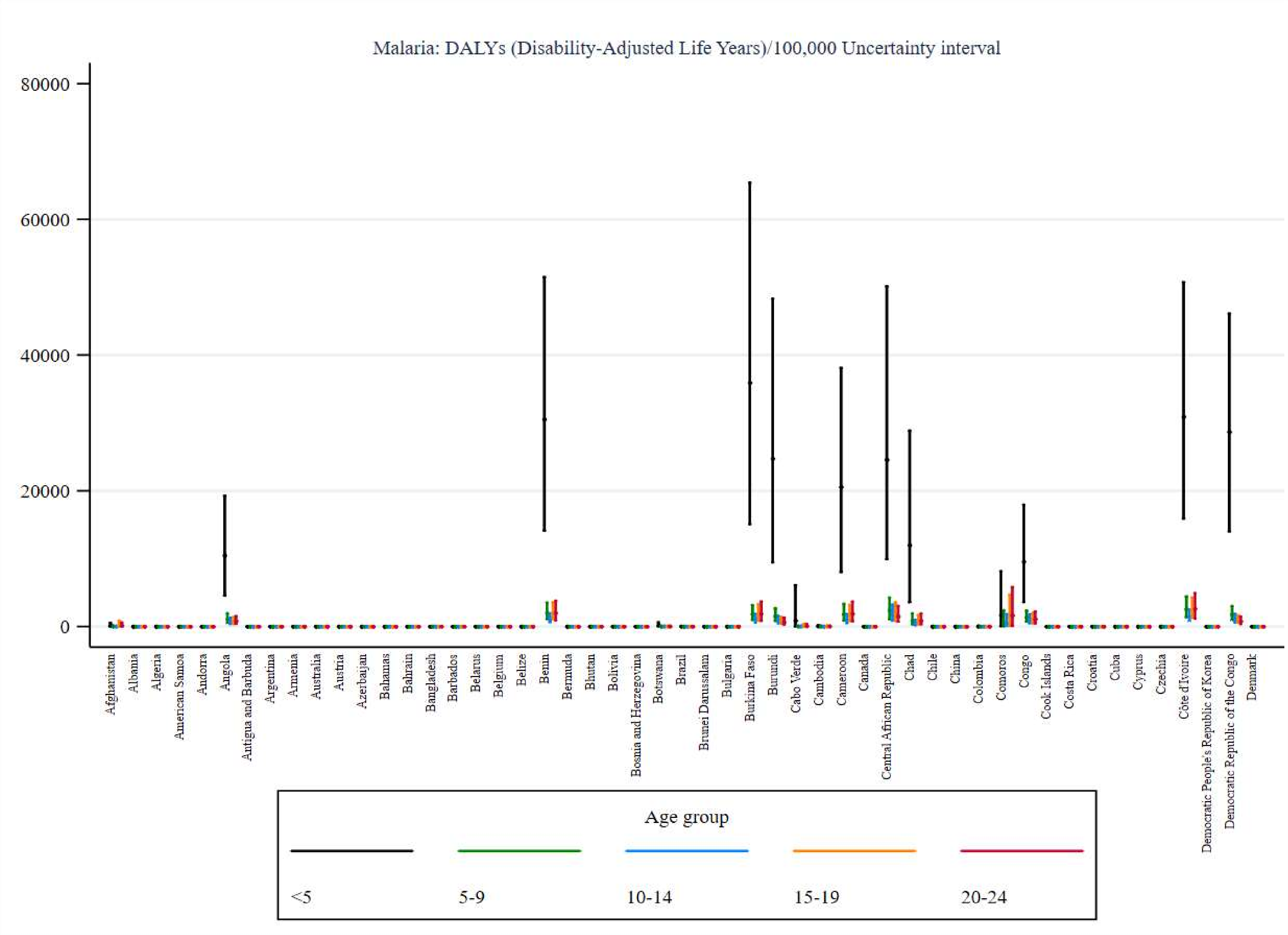

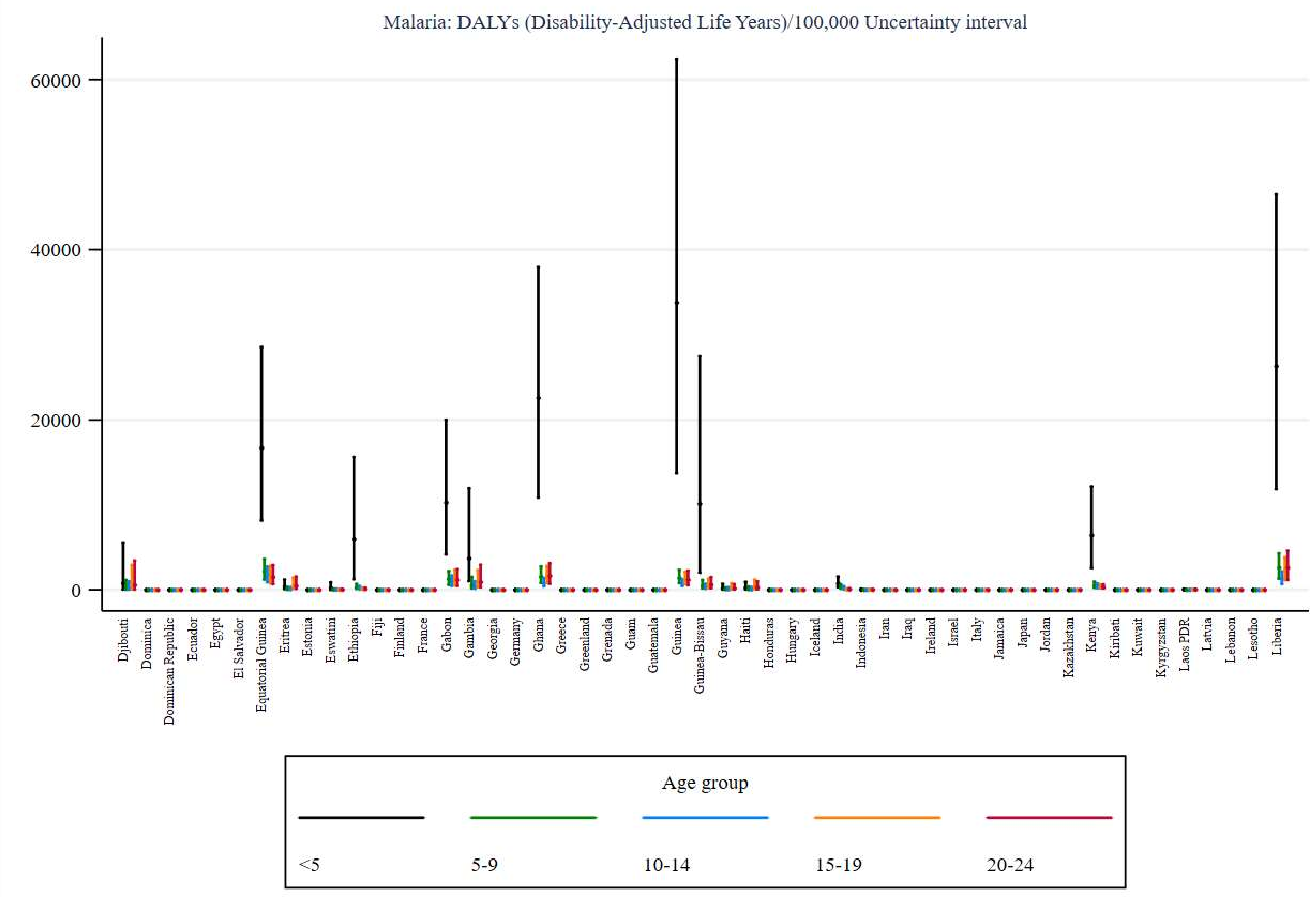

Malaria: DALYs (Disability-Adjusted Life Years)/100,000 Uncertainty interval
60000
40000
20000
0
Djibouti
Dominica
Dominican Republic
Ecuador
Egypt
El Salvador
Equatorial Guinea
Eritrea
Estonia
Eswatini
Ethiopia
Fiji
Finland
France
Gabon
Gambia
Georgia
Germany
Ghana
Greece
Greenland
Grenada
Guam
Guatemala
Guinea
Guinea-Bissau
Guyana
Haiti
Honduras
Hungary
Iceland
India
Indonesia
Iran
Iraq
Ireland
Israel
Italy
Jamaica
Japan
Jordan
Kazakhstan
Kenya
Kiribati
Kuwait
Kyrgyzstan
Laos PDR
Latvia
Lebanon
Lesotho
Liberia
Age group
<5
5-9
10-14
15-19
20-24

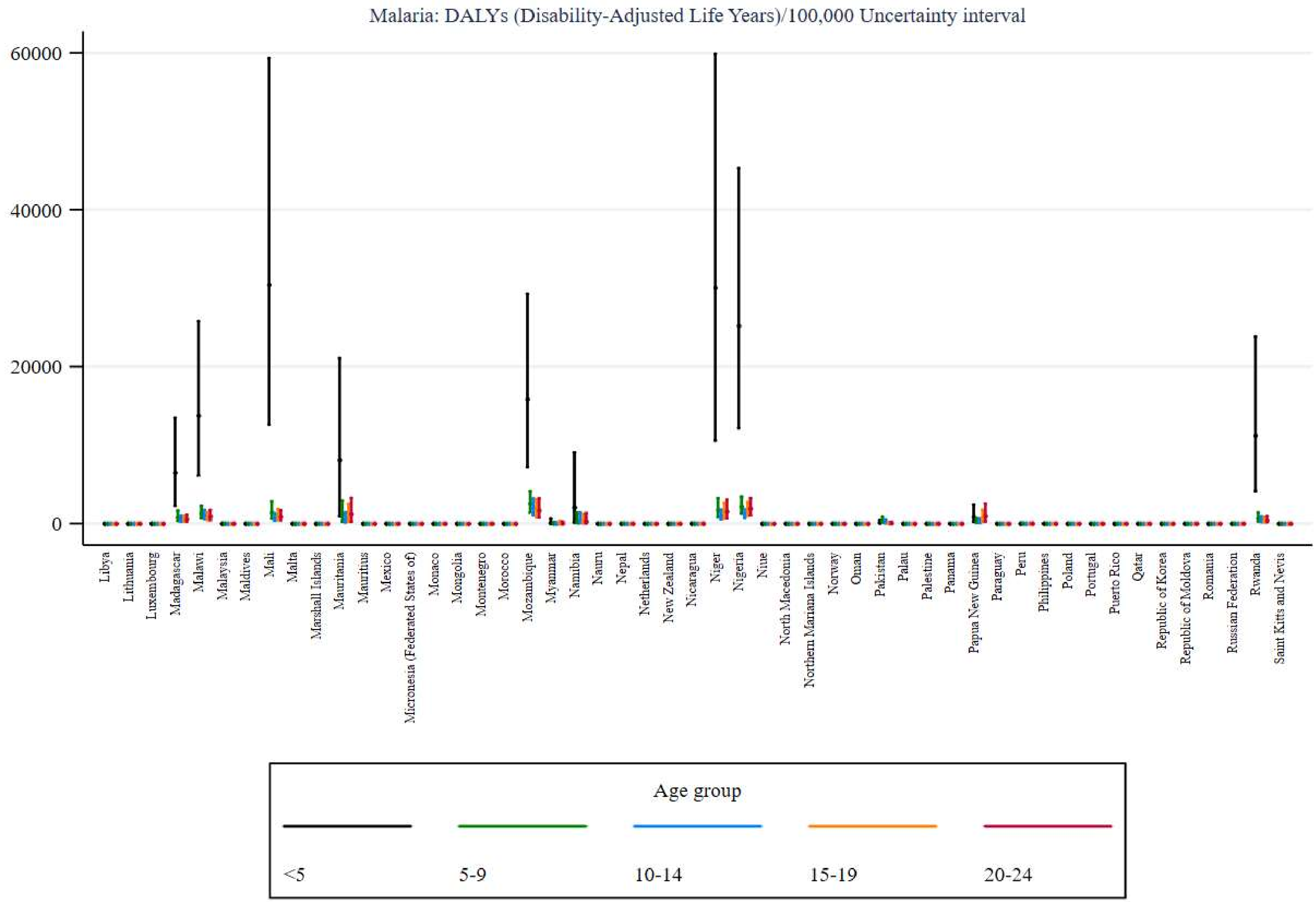

Malaria: DALYs (Disability-Adjusted Life Years)/100,000 Uncertainty interval
60000
40000
20000
0
Libya
Lithuania
Luxembourg
Madagascar
Malawi
Malaysia
Maldives
Mali
Malta
Marshall Islands
Mauritania
Mauritius
Mexico
Micronesia (Federated States of)
Monaco
Mongolia
Montenegro
Morocco
Mozambique
Myanmar
Namibia
Nauru
Nepal
Netherlands
New Zealand
Nicaragua
Niger
Nigeria
Niue
North Macedonia
Northern Mariana Islands
Norway
Oman
Pakistan
Palau
Palestine
Panama
Papua New Guinea
Paraguay
Peru
Philippines
Poland
Portugal
Puerto Rico
Qatar
Republic of Korea
Republic of Moldova
Romania
Russian Federation
Rwanda
Saint Kitts and Nevis
Age group
<5
5-9
10-14
15-19
20-24

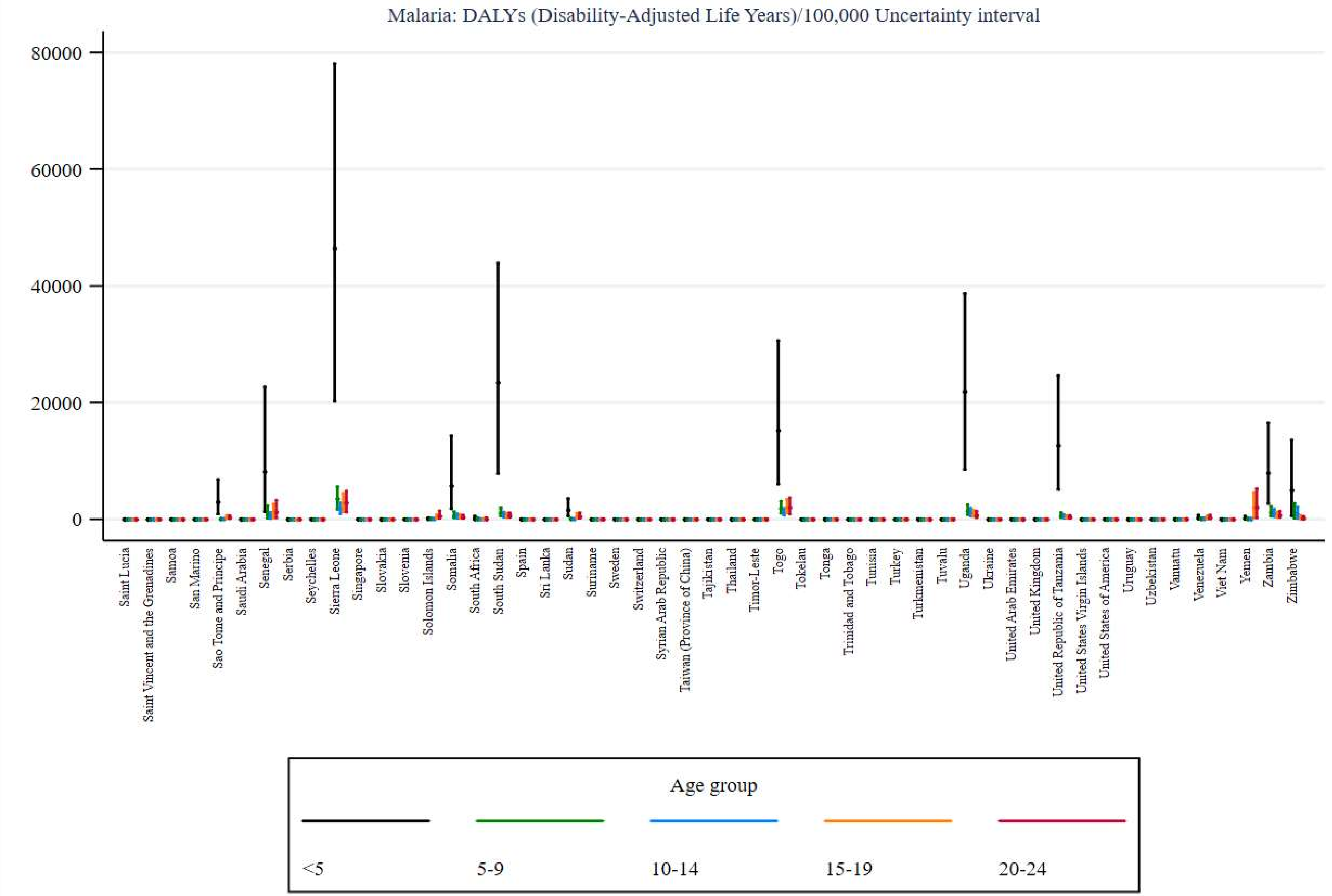

Malaria: DALYs (Disability-Adjusted Life Years)/100,000 Uncertainty interval
80000
60000
40000
20000
0
Saint Lucia
Saint Vincent and the Grenadines
Samoa
San Marino
Sao Tome and Principe
Saudi Arabia
Senegal
Serbia
Seychelles
Sierra Leone
Singapore
Slovakia
Slovenia
Solomon Islands
Somalia
South Africa
South Sudan
Spain
Sri Lanka
Sudan
Suriname
Sweden
Switzerland
Syrian Arab Republic
Taiwan (Province of China)
Tajikistan
Thailand
Timor-Leste
Togo
Tokelau
Tonga
Trinidad and Tobago
Tunisia
Turkey
Turkmenistan
Tuvalu
Uganda
Ukraine
United Arab Emirates
United Kingdom
United Republic of Tanzania
United States Virgin Islands
United States of America
Uruguay
Uzbekistan
Vanuatu
Venezuela
Viet Nam
Yemen
Zambia
Zimbabwe
Age group
<5
5-9
10-14
15-19
20-24

S20_5 Part A: Maternal sepsis and other maternal infections Deaths/ 100 000 Uncertainty interval for each age group

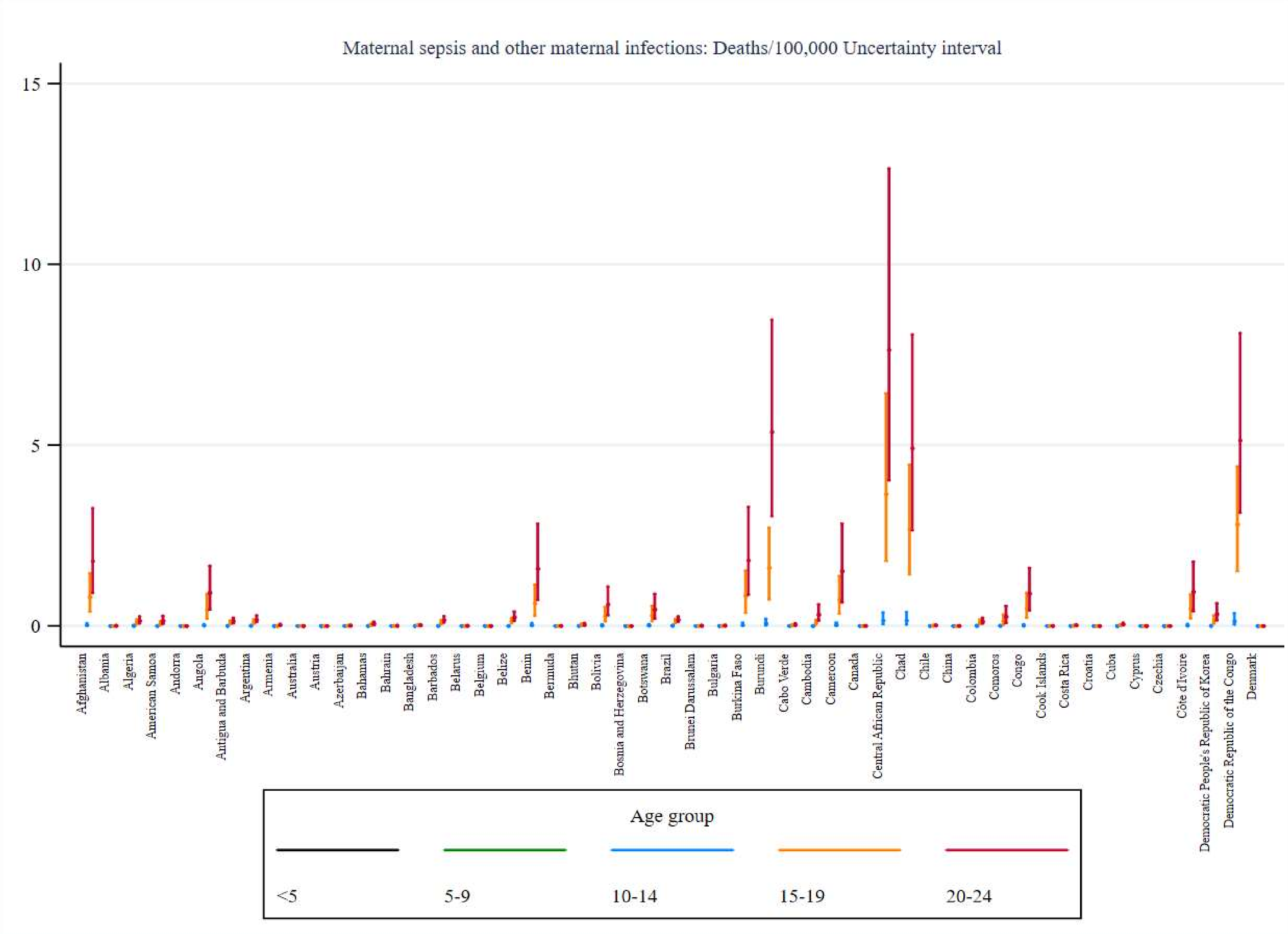

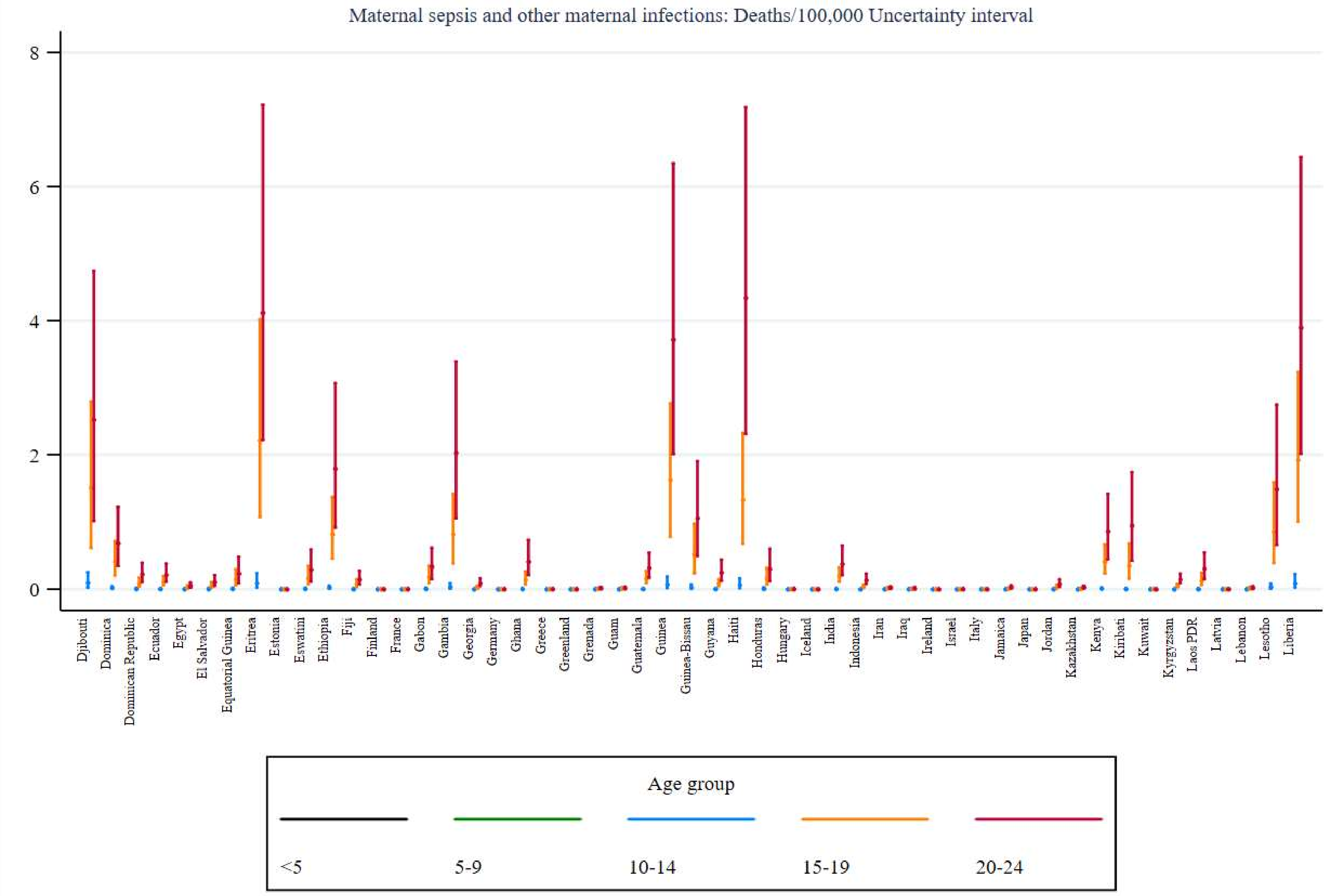
Maternal sepsis and other maternal infections: Deaths/100,000 Uncertainty interval
8
6
4
2
0
Djibouti
Dominica
Dominican Republic
Ecuador
Egypt
El Salvador
Equatorial Guinea
Eritrea
Estonia
Eswatini
Ethiopia
Fiji
Finland
France
Gabon
Gambia
Georgia
Germany
Ghana
Greece
Greenland
Grenada
Guam
Guatemala
Guinea
Guinea-Bissau
Guyana
Haiti
Honduras
Hungary
Iceland
India
Indonesia
Iran
Iraq
Ireland
Israel
Italy
Jamaica
Japan
Jordan
Kazakhstan
Kenya
Kiribati
Kuwait
Kyrgyzstan
Laos PDR
Latvia
Lebanon
Lesotho
Liberia
Age group
<5
5-9
10-14
15-19
20-24

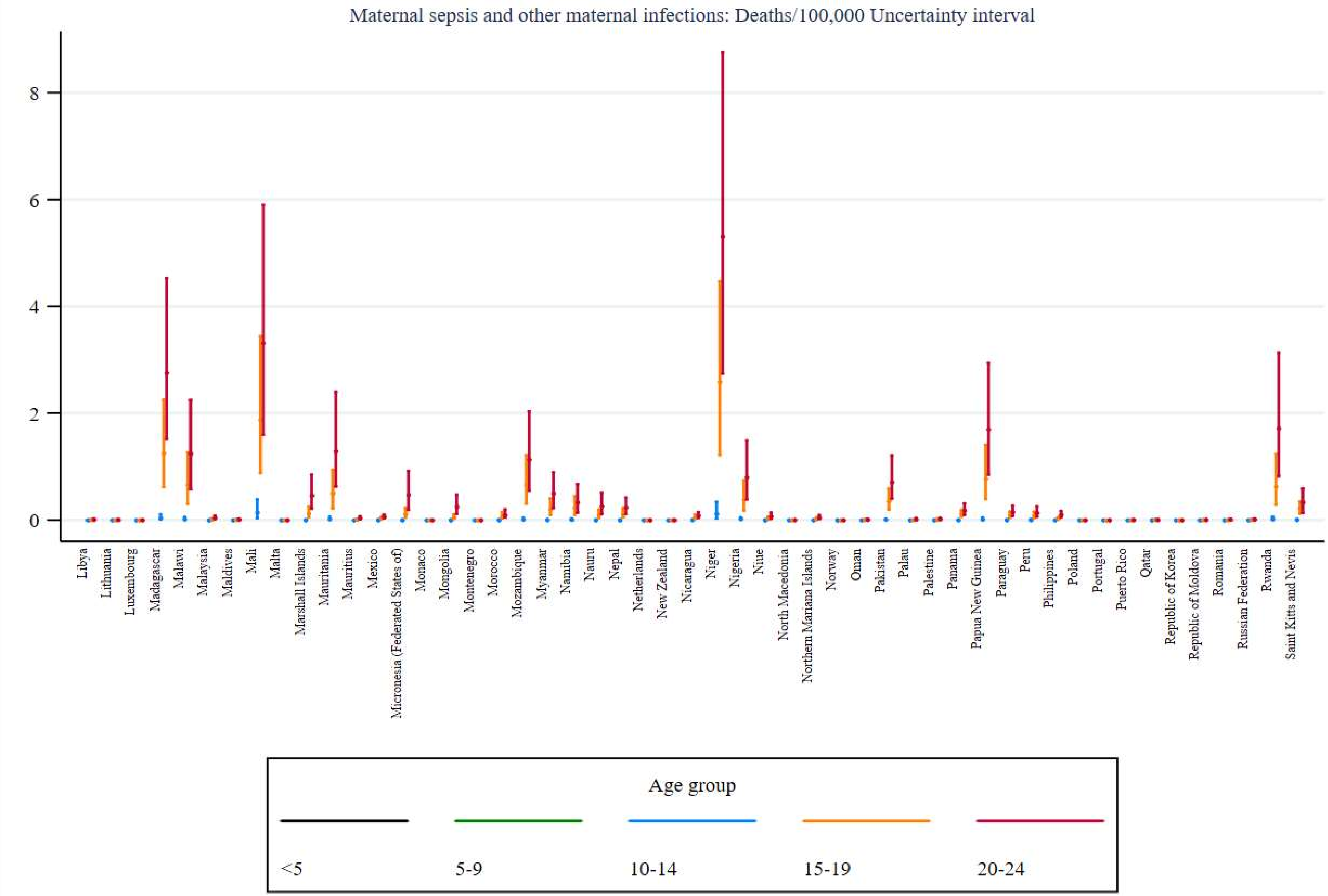
Maternal sepsis and other maternal infections: Deaths/100,000 Uncertainty interval
8
6
4
2
0
Libya
Lithuania
Luxembourg
Madagascar
Malawi
Malaysia
Maldives
Mali
Malta
Marshall Islands
Mauritania
Mauritius
Mexico
Micronesia (Federated States of)
Monaco
Mongolia
Montenegro
Morocco
Mozambique
Myanmar
Namibia
Nauru
Nepal
Netherlands
New Zealand
Nicaragua
Niger
Nigeria
Niue
North Macedonia
Northern Mariana Islands
Norway
Oman
Pakistan
Palau
Palestine
Panama
Papua New Guinea
Paraguay
Peru
Philippines
Poland
Portugal
Puerto Rico
Qatar
Republic of Korea
Republic of Moldova
Romania
Russian Federation
Rwanda
Saint Kitts and Nevis
Age group
<5
5-9
10-14
15-19
20-24

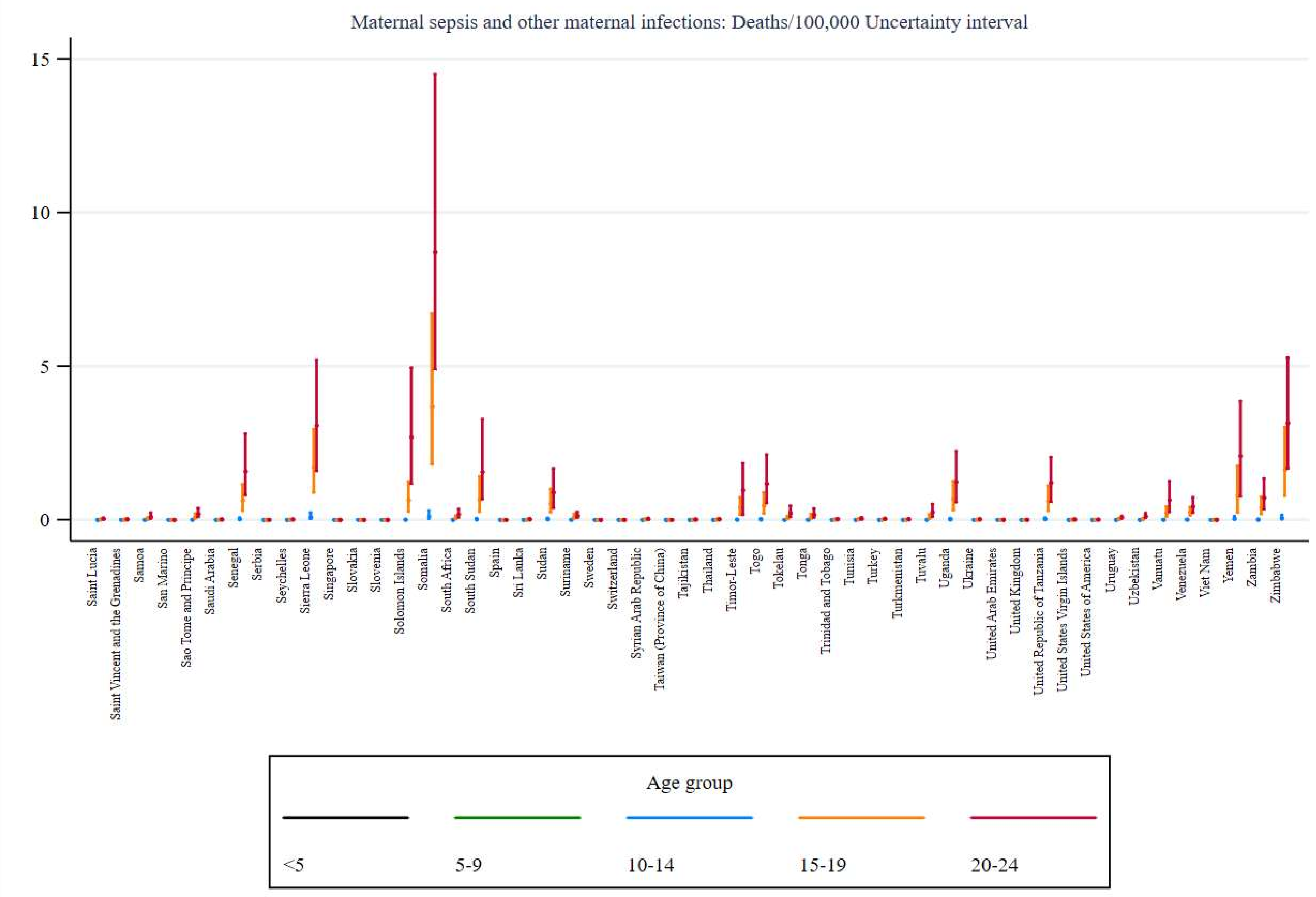
Maternal sepsis and other maternal infections: Deaths/100,000 Uncertainty interval
15
10
5
0
Saint Lucia
Saint Vincent and the Grenadines
Samoa
San Marino
Sao Tome and Principe
Saudi Arabia
Senegal
Serbia
Seychelles
Sierra Leone
Singapore
Slovakia
Slovenia
Solomon Islands
Somalia
South Africa
South Sudan
Spain
Sri Lanka
Sudan
Suriname
Sweden
Switzerland
Syrian Arab Republic
Taiwan (Province of China)
Tajikistan
Thailand
Timor-Leste
Togo
Tokelau
Tonga
Trinidad and Tobago
Tunisia
Turkey
Turkmenistan
Tuvalu
Uganda
Ukraine
United Arab Emirates
United Kingdom
United Republic of Tanzania
United States Virgin Islands
United States of America
Uruguay
Uzbekistan
Vanuatu
Venezuela
Viet Nam
Yemen
Zambia
Zimbabwe
Age group
<5
5-9
10-14
15-19
20-24

S20_5 Part B: Maternal sepsis and other maternal infections DALYs/ 100 000 Uncertainty interval for each age group

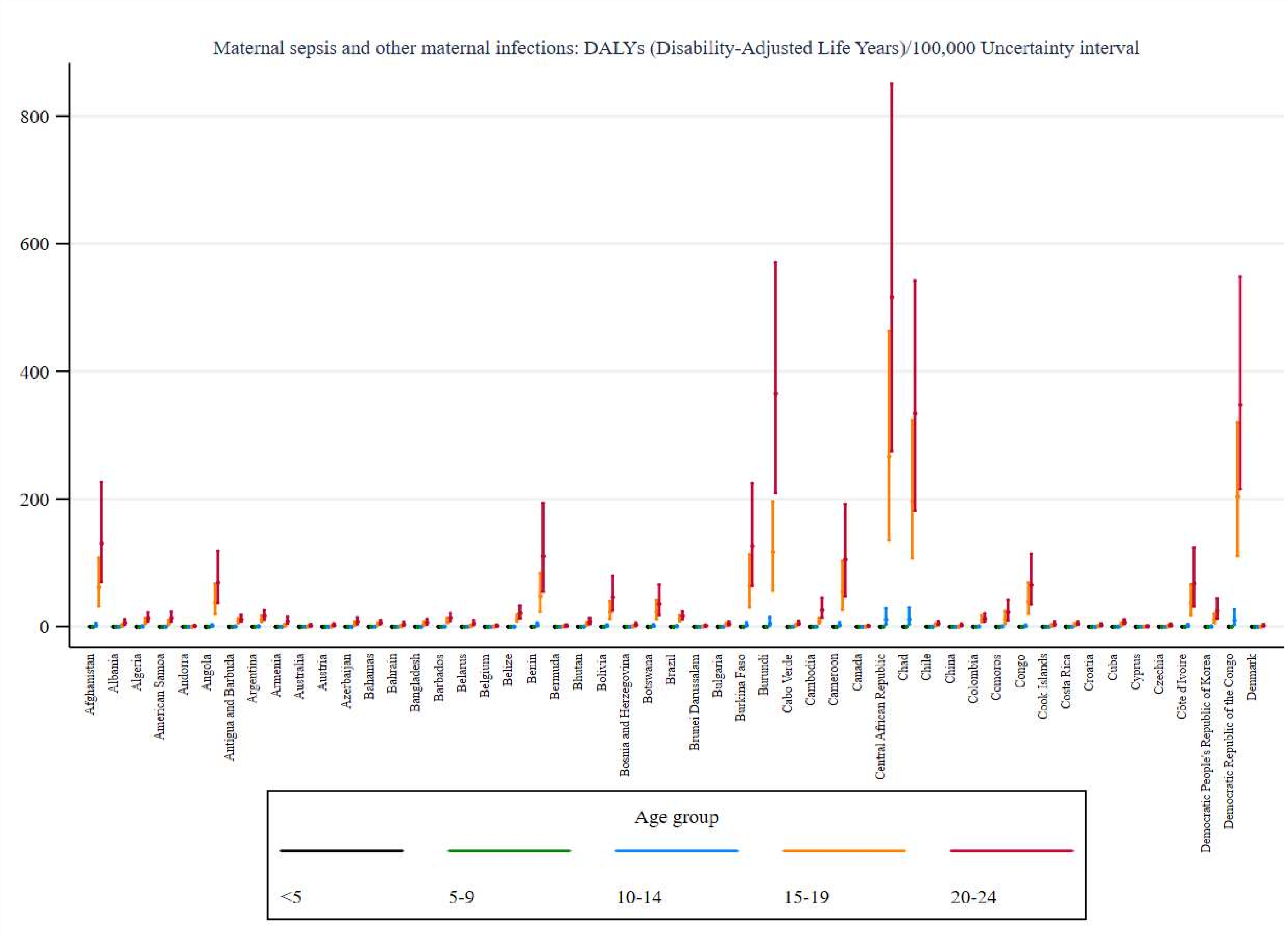

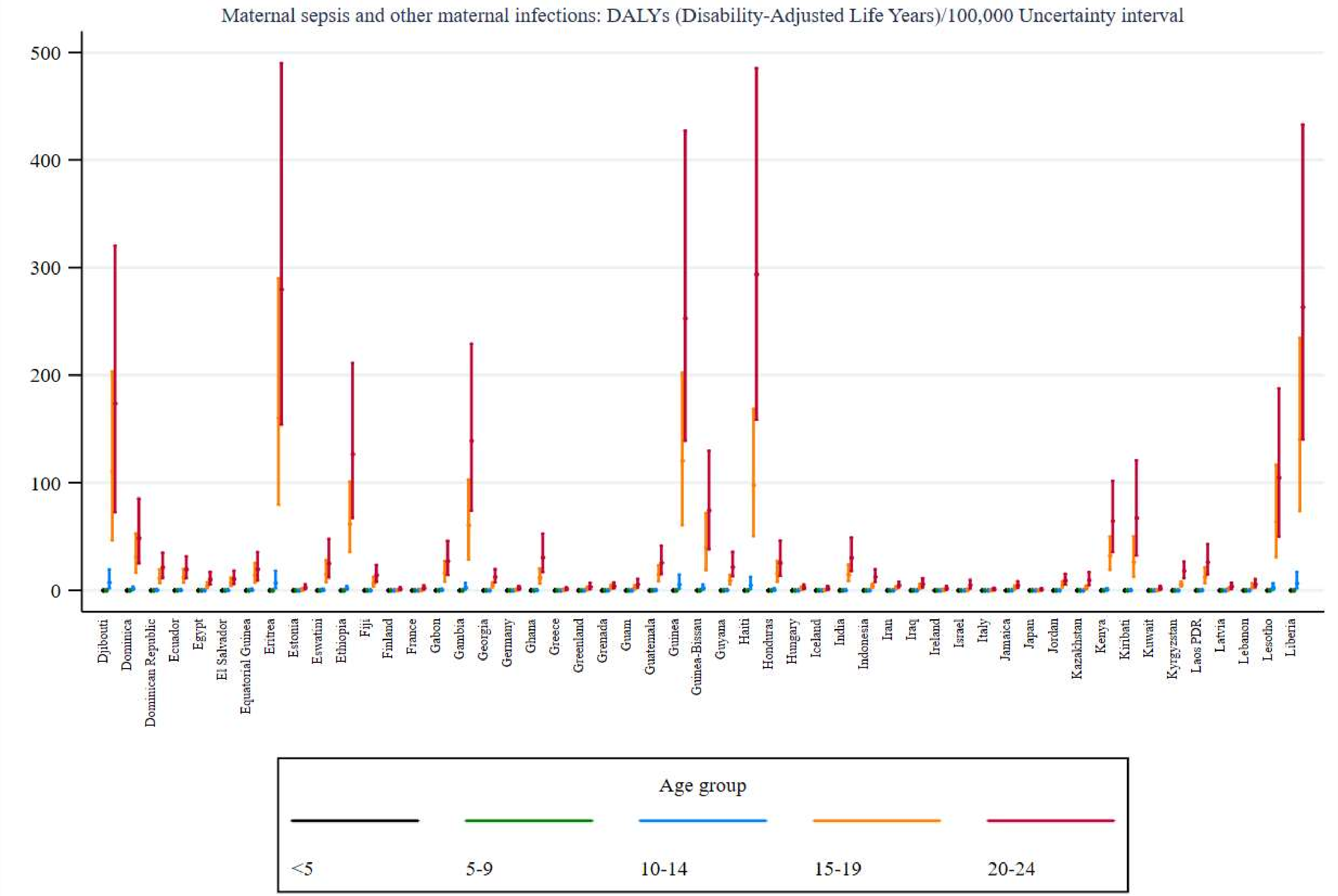

Maternal sepsis and other maternal infections: DALYs (Disability-Adjusted Life Years)/100,000 Uncertainty interval
500
400
300
200
100
0
Djibouti
Dominica
Dominican Republic
Ecuador
Egypt
El Salvador
Equatorial Guinea
Eritrea
Estonia
Eswatini
Ethiopia
Fiji
Finland
France
Gabon
Gambia
Georgia
Germany
Ghana
Greece
Greenland
Grenada
Guam
Guatemala
Guinea
Guinea-Bissau
Guyana
Haiti
Honduras
Hungary
Iceland
India
Indonesia
Iran
Iraq
Ireland
Israel
Italy
Jamaica
Japan
Jordan
Kazakhstan
Kenya
Kiribati
Kuwait
Kyrgyzstan
Laos PDR
Latvia
Lebanon
Lesotho
Liberia
Age group
<5
5-9
10-14
15-19
20-24

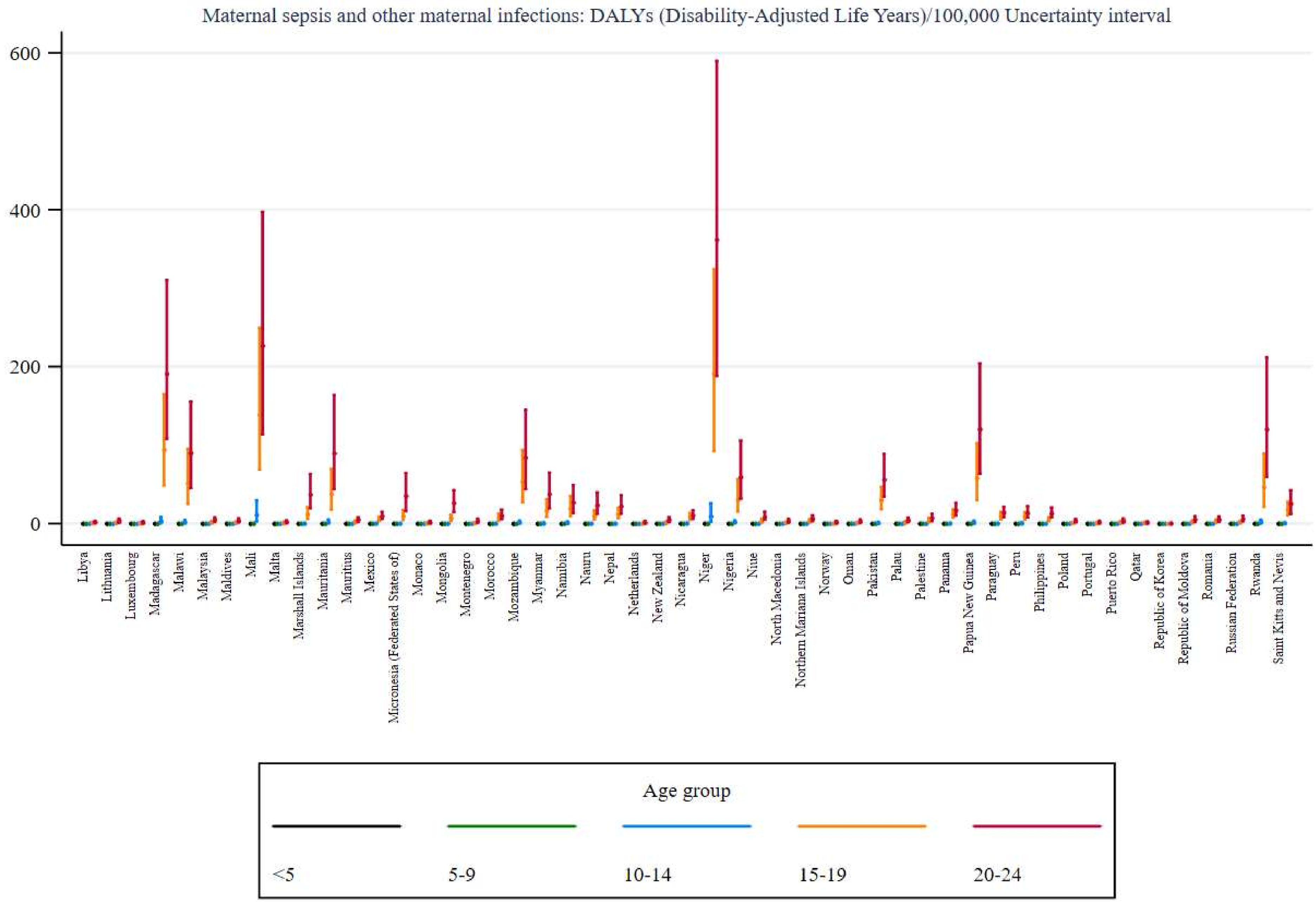
Maternal sepsis and other maternal infections: DALYs (Disability-Adjusted Life Years)/100,000 Uncertainty interval
600
400
200
0
Libya
Lithuania
Luxembourg
Madagascar
Malawi
Malaysia
Maldives
Mali
Malta
Marshall Islands
Mauritania
Mauritius
Mexico
Micronesia (Federated States of)
Monaco
Mongolia
Montenegro
Morocco
Mozambique
Myanmar
Namibia
Nauru
Nepal
Netherlands
New Zealand
Nicaragua
Niger
Nigeria
Niue
North Macedonia
Northern Mariana Islands
Norway
Oman
Pakistan
Palau
Palestine
Panama
Papua New Guinea
Paraguay
Peru
Philippines
Poland
Portugal
Puerto Rico
Qatar
Republic of Korea
Republic of Moldova
Romania
Russian Federation
Rwanda
Saint Kitts and Nevis
Age group
<5
5-9
10-14
15-19
20-24

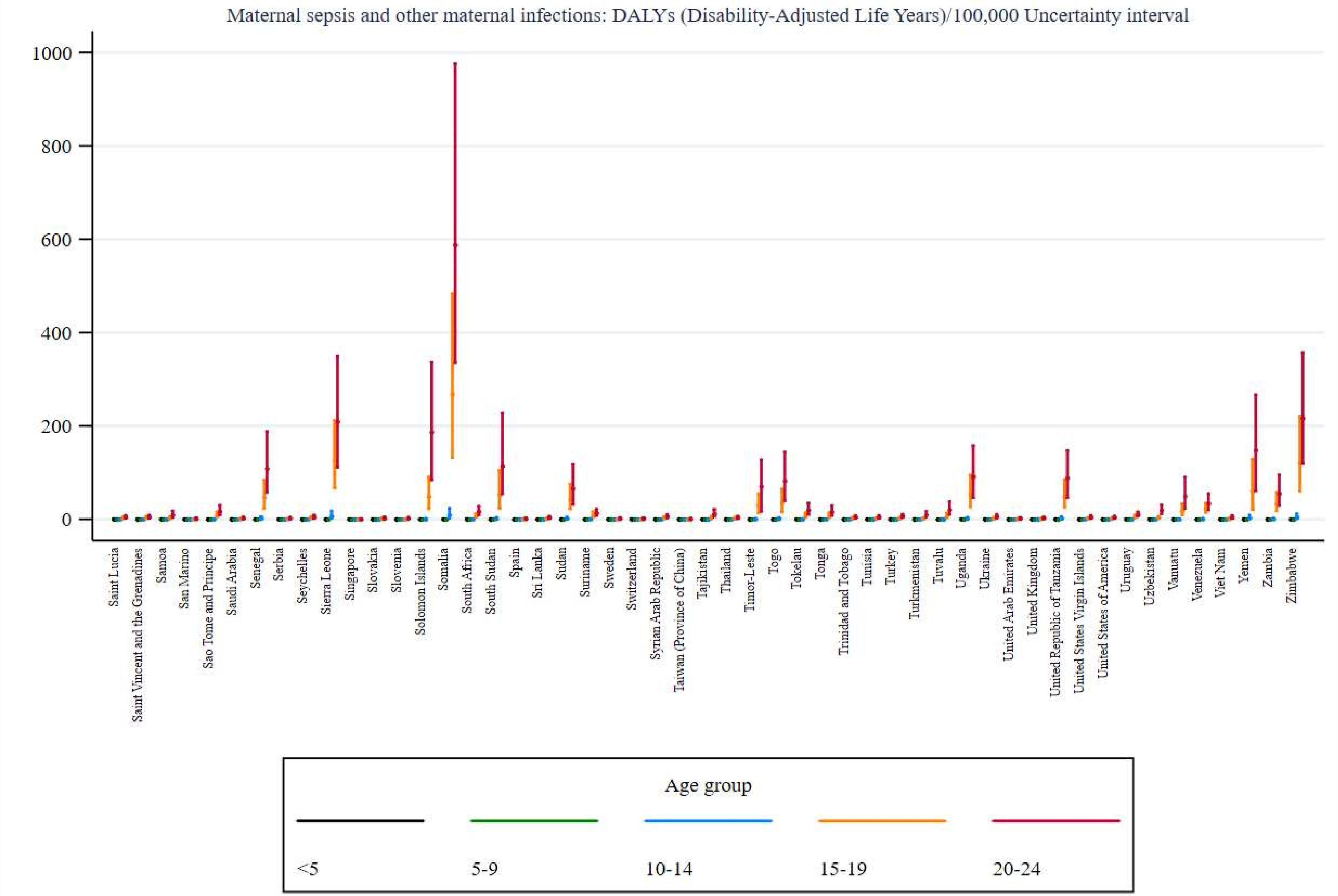
Maternal sepsis and other maternal infections: DALYs (Disability-Adjusted Life Years)/100,000 Uncertainty interval
1000
800
600
400
200
0
Saint Lucia
Saint Vincent and the Grenadines
Samoa
San Marino
Sao Tome and Principe
Saudi Arabia
Senegal
Serbia
Seychelles
Sierra Leone
Singapore
Slovakia
Slovenia
Solomon Islands
Somalia
South Africa
South Sudan
Spain
Sri Lanka
Sudan
Suriname
Sweden
Switzerland
Syrian Arab Republic
Taiwan (Province of China)
Tajikistan
Thailand
Timor-Leste
Togo
Tokelau
Tonga
Trinidad and Tobago
Tunisia
Turkey
Turkmenistan
Tuvalu
Uganda
Ukraine
United Arab Emirates
United Kingdom
United Republic of Tanzania
United States Virgin Islands
United States of America
Uruguay
Uzbekistan
Vanuatu
Venezuela
Viet Nam
Yemen
Zambia
Zimbabwe
Age group
<5
5-9
10-14
15-19
20-24

S20_6 Part A: Neonatal sepsis and other neonatal infections Deaths/ 100 000 Uncertainty interval for each age group

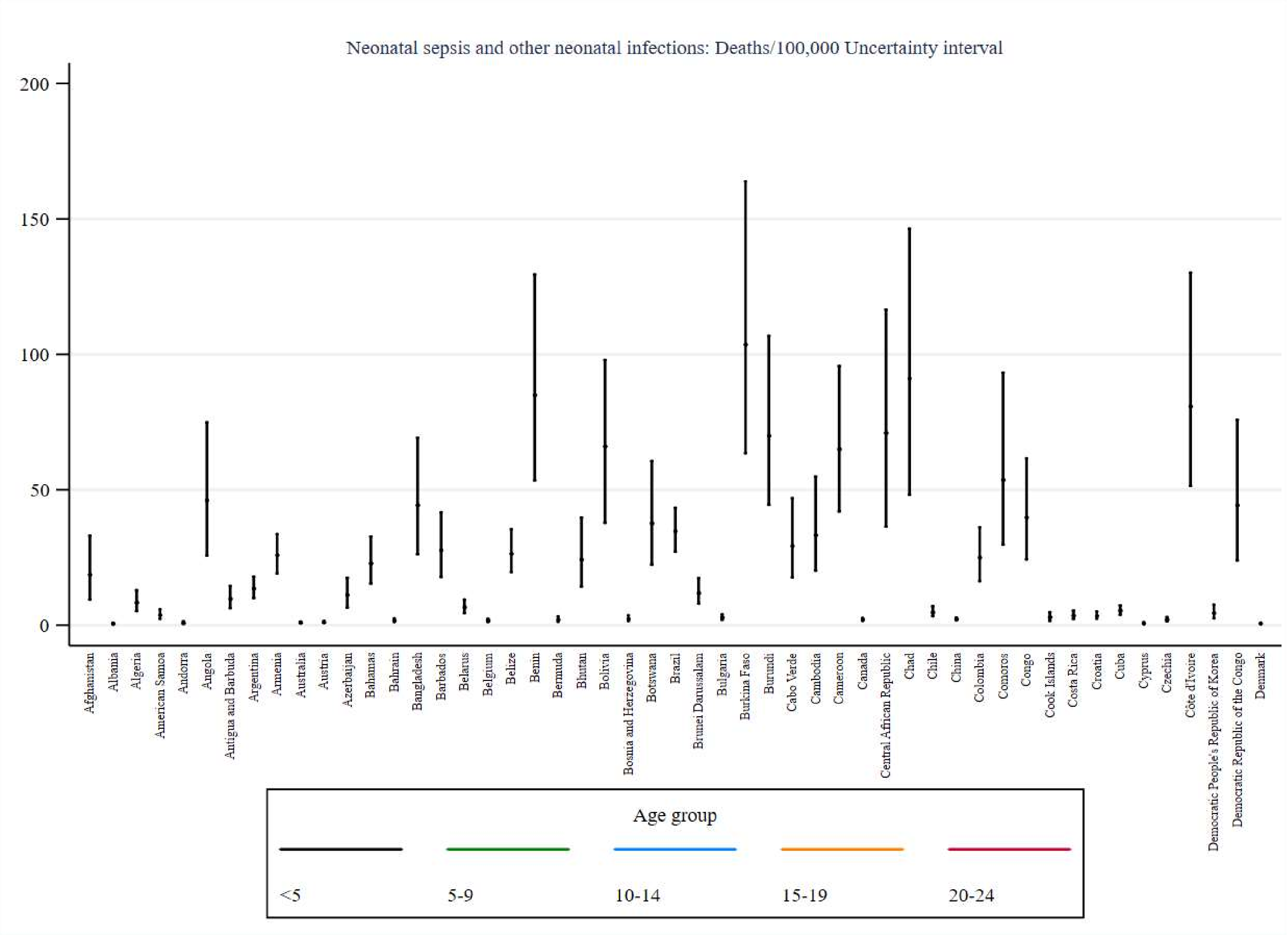

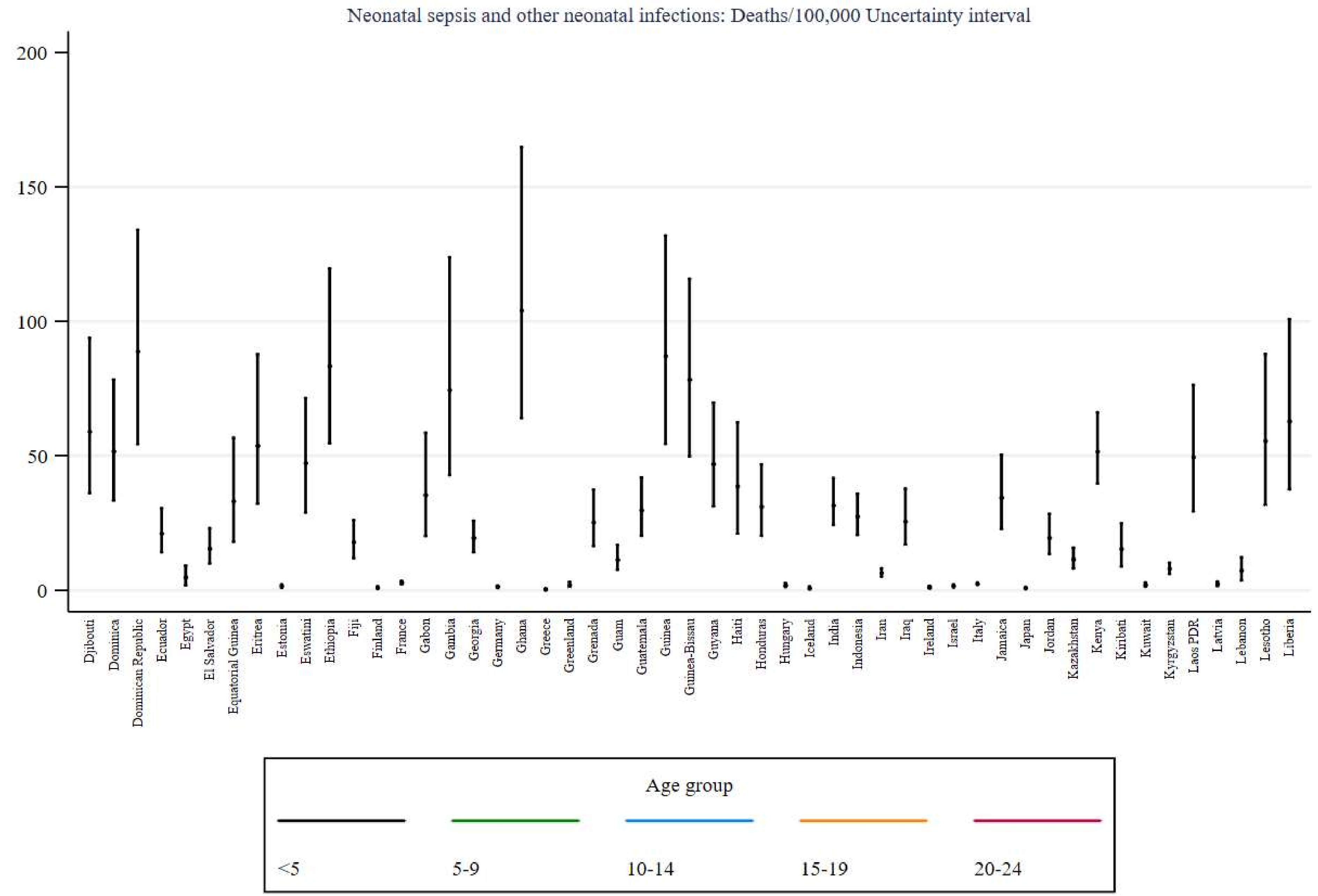
Neonatal sepsis and other neonatal infections: Deaths/100,000 Uncertainty interval
200
150
100
50
0
Djibouti
Dominica
Dominican Republic
Ecuador
Egypt
El Salvador
Equatorial Guinea
Eritrea
Estonia
Eswatini
Ethiopia
Fiji
Finland
France
Gabon
Gambia
Georgia
Germany
Ghana
Greece
Greenland
Grenada
Guam
Guatemala
Guinea
Guinea-Bissau
Guyana
Haiti
Honduras
Hungary
Iceland
India
Indonesia
Iran
Iraq
Ireland
Israel
Italy
Jamaica
Japan
Jordan
Kazakhstan
Kenya
Kiribati
Kuwait
Kyrgyzstan
Laos PDR
Latvia
Lebanon
Lesotho
Liberia
Age group
<5
5-9
10-14
15-19
20-24

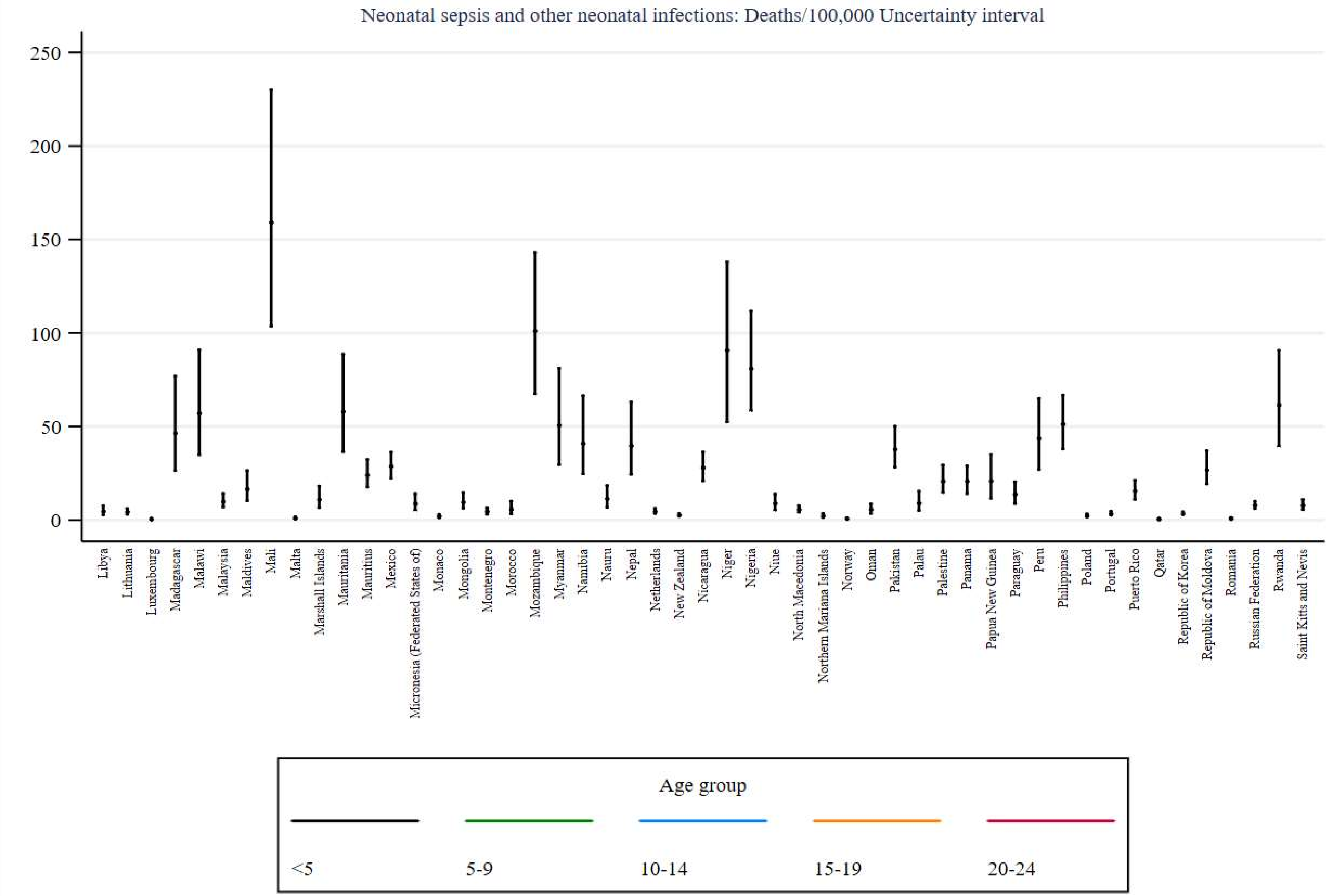
Neonatal sepsis and other neonatal infections: Deaths/100,000 Uncertainty interval
250
200
150
100
50
0
Libya
Lithuania
Luxembourg
Madagascar
Malawi
Malaysia
Maldives
Mali
Malta
Marshall Islands
Mauritania
Mauritius
Mexico
Micronesia (Federated States of)
Monaco
Mongolia
Montenegro
Morocco
Mozambique
Myanmar
Namibia
Nauru
Nepal
Netherlands
New Zealand
Nicaragua
Niger
Nigeria
Niue
North Macedonia
Northern Mariana Islands
Norway
Oman
Pakistan
Palau
Palestine
Panama
Papua New Guinea
Paraguay
Peru
Philippines
Poland
Portugal
Puerto Rico
Qatar
Republic of Korea
Republic of Moldova
Romania
Russian Federation
Rwanda
Saint Kitts and Nevis
Age group
<5
5-9
10-14
15-19
20-24

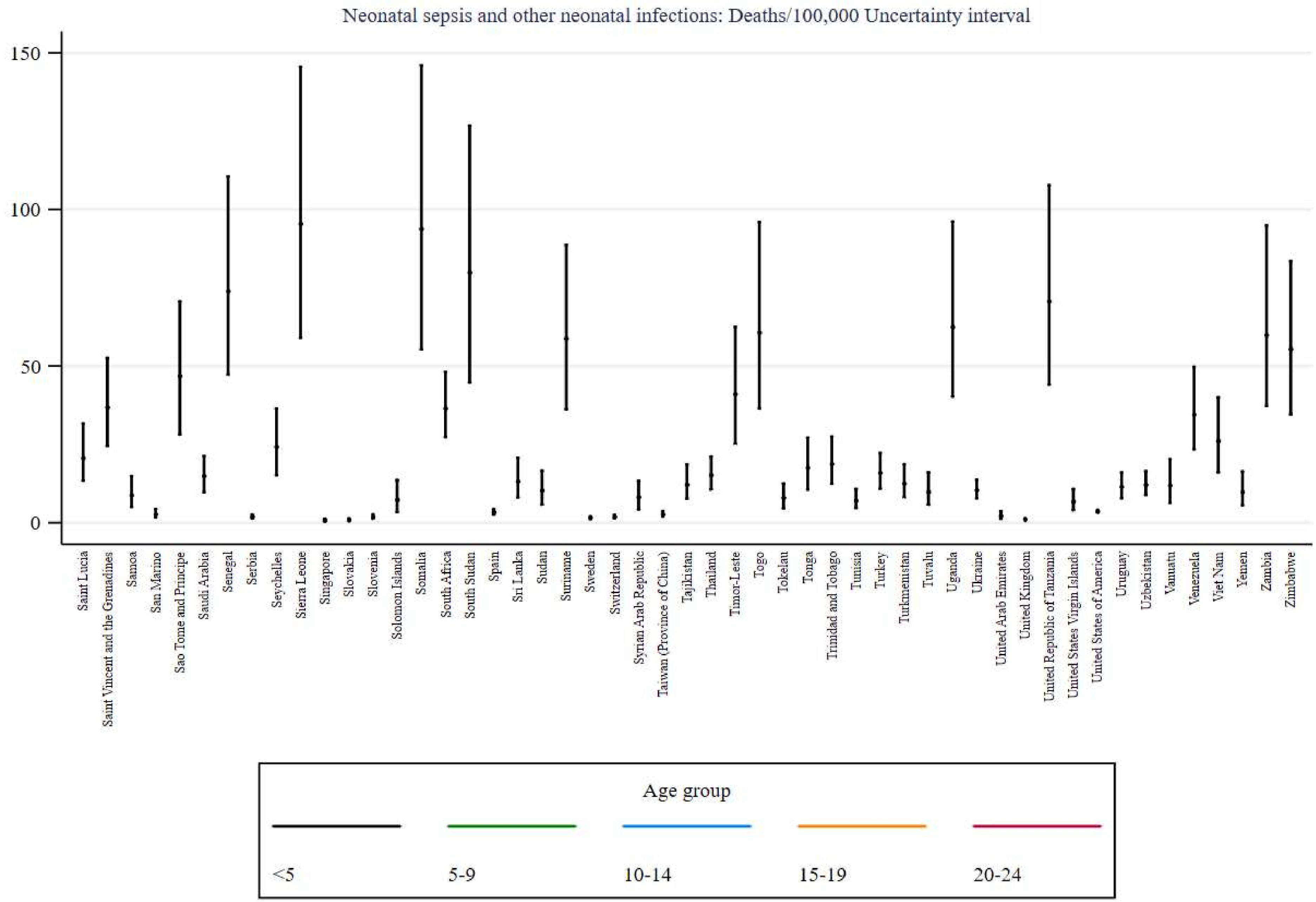

Neonatal sepsis and other neonatal infections: Deaths/100,000 Uncertainty interval
150
100
50
0
Saint Lucia
Saint Vincent and the Grenadines
Samoa
San Marino
Sao Tome and Principe
Saudi Arabia
Senegal
Serbia
Seychelles
Sierra Leone
Singapore
Slovakia
Slovenia
Solomon Islands
Somalia
South Africa
South Sudan
Spain
Sri Lanka
Sudan
Suriname
Sweden
Switzerland
Syrian Arab Republic
Taiwan (Province of China)
Tajikistan
Thailand
Timor-Leste
Togo
Tokelau
Tonga
Trinidad and Tobago
Tunisia
Turkey
Turkmenistan
Tuvalu
Uganda
Ukraine
United Arab Emirates
United Kingdom
United Republic of Tanzania
United States Virgin Islands
United States of America
Uruguay
Uzbekistan
Vanuatu
Venezuela
Viet Nam
Yemen
Zambia
Zimbabwe
Age group
<5
5-9
10-14
15-19
20-24

S20_6 Part B: Neonatal sepsis and other neonatal infections DALYs/ 100 000 Uncertainty interval for each age group

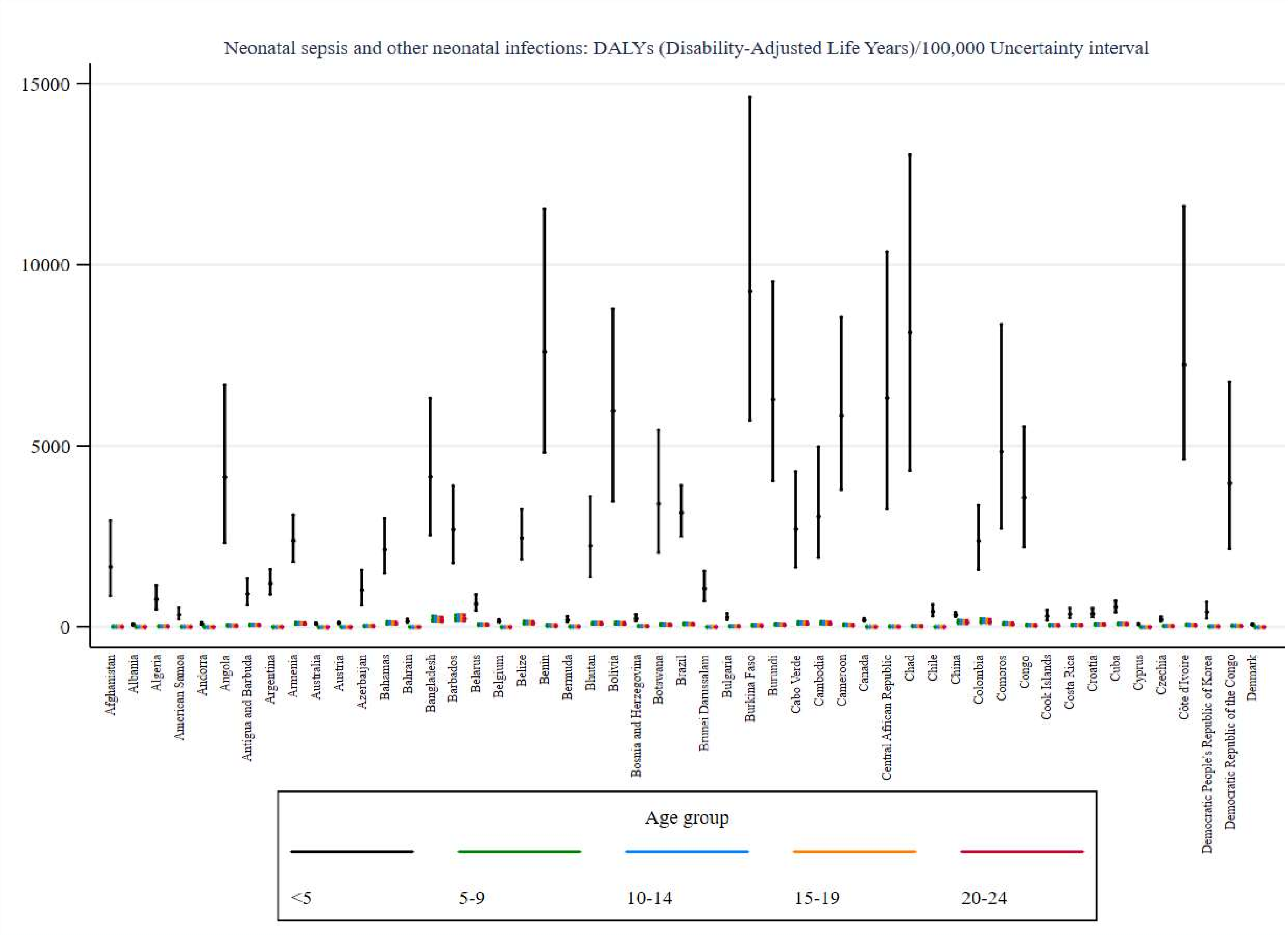

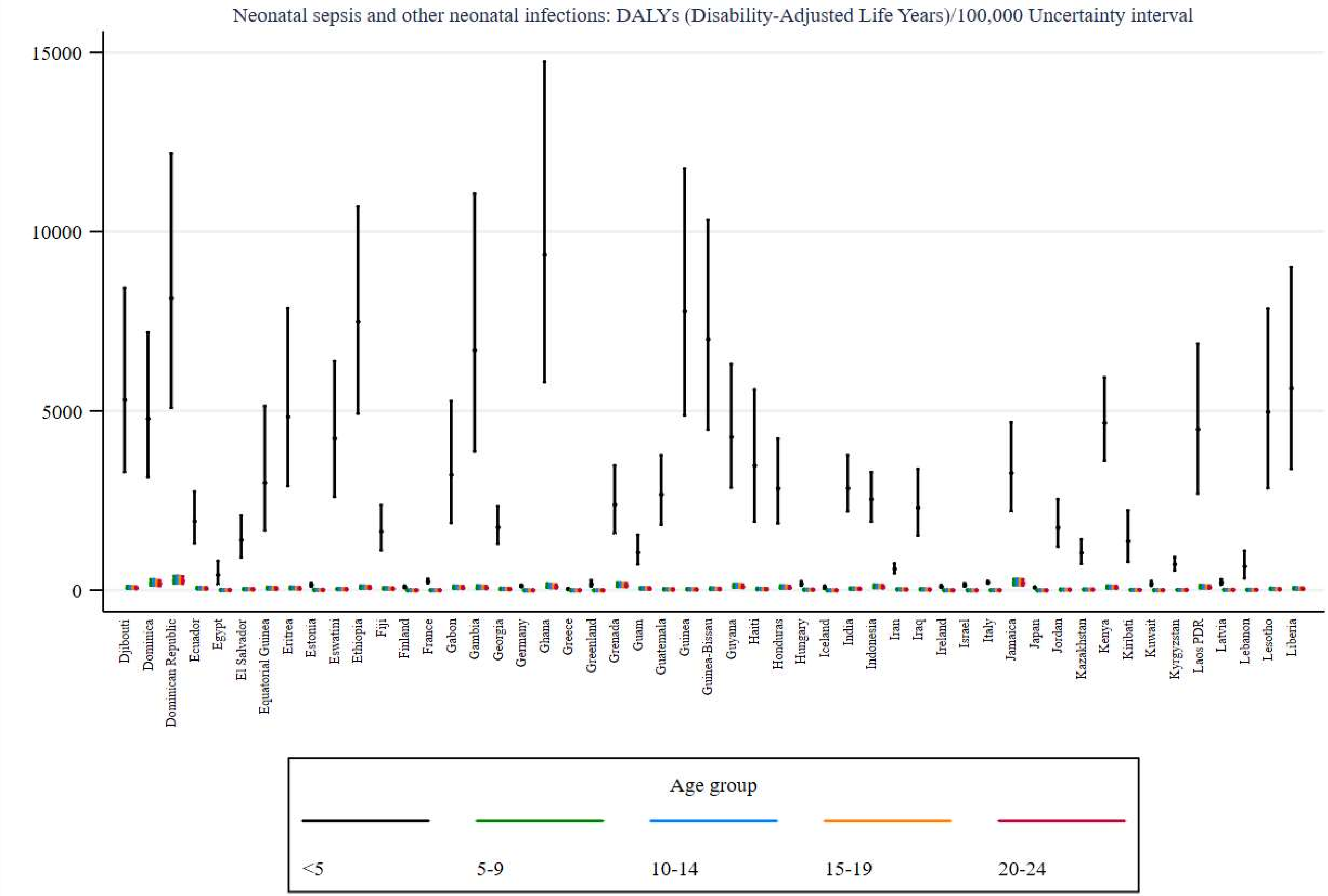

Neonatal sepsis and other neonatal infections: DALYs (Disability-Adjusted Life Years)/100,000 Uncertainty interval
15000
10000
5000
0
Djibouti
Dominica
Dominican Republic
Ecuador
Egypt
El Salvador
Equatorial Guinea
Eritrea
Estonia
Eswatini
Ethiopia
Fiji
Finland
France
Gabon
Gambia
Georgia
Germany
Ghana
Greece
Greenland
Grenada
Guam
Guatemala
Guinea
Guinea-Bissau
Guyana
Haiti
Honduras
Hungary
Iceland
India
Indonesia
Iran
Iraq
Ireland
Israel
Italy
Jamaica
Japan
Jordan
Kazakhstan
Kenya
Kiribati
Kuwait
Kyrgyzstan
Laos PDR
Latvia
Lebanon
Lesotho
Liberia
Age group
<5
5-9
10-14
15-19
20-24

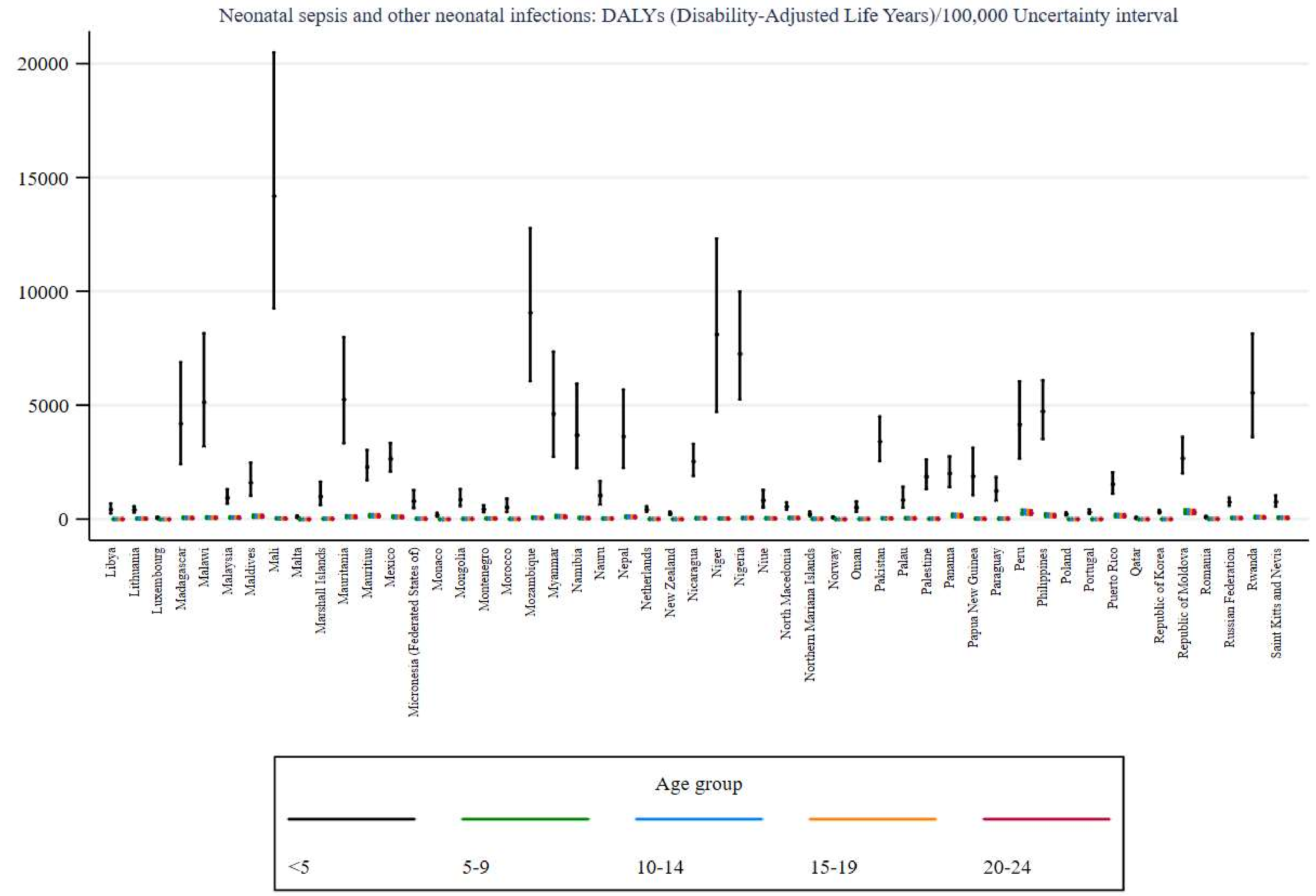
Neonatal sepsis and other neonatal infections: DALYs (Disability-Adjusted Life Years)/100,000 Uncertainty interval
20000
15000
10000
5000
0
Libya
Lithuania
Luxembourg
Madagascar
Malawi
Malaysia
Maldives
Mali
Malta
Marshall Islands
Mauritania
Mauritius
Mexico
Micronesia (Federated States of)
Monaco
Mongolia
Montenegro
Morocco
Mozambique
Myanmar
Namibia
Nauru
Nepal
Netherlands
New Zealand
Nicaragua
Niger
Nigeria
Niue
North Macedonia
Northern Mariana Islands
Norway
Oman
Pakistan
Palau
Palestine
Panama
Papua New Guinea
Paraguay
Peru
Philippines
Poland
Portugal
Puerto Rico
Qatar
Republic of Korea
Republic of Moldova
Romania
Russian Federation
Rwanda
Saint Kitts and Nevis
Age group
<5
5-9
10-14
15-19
20-24

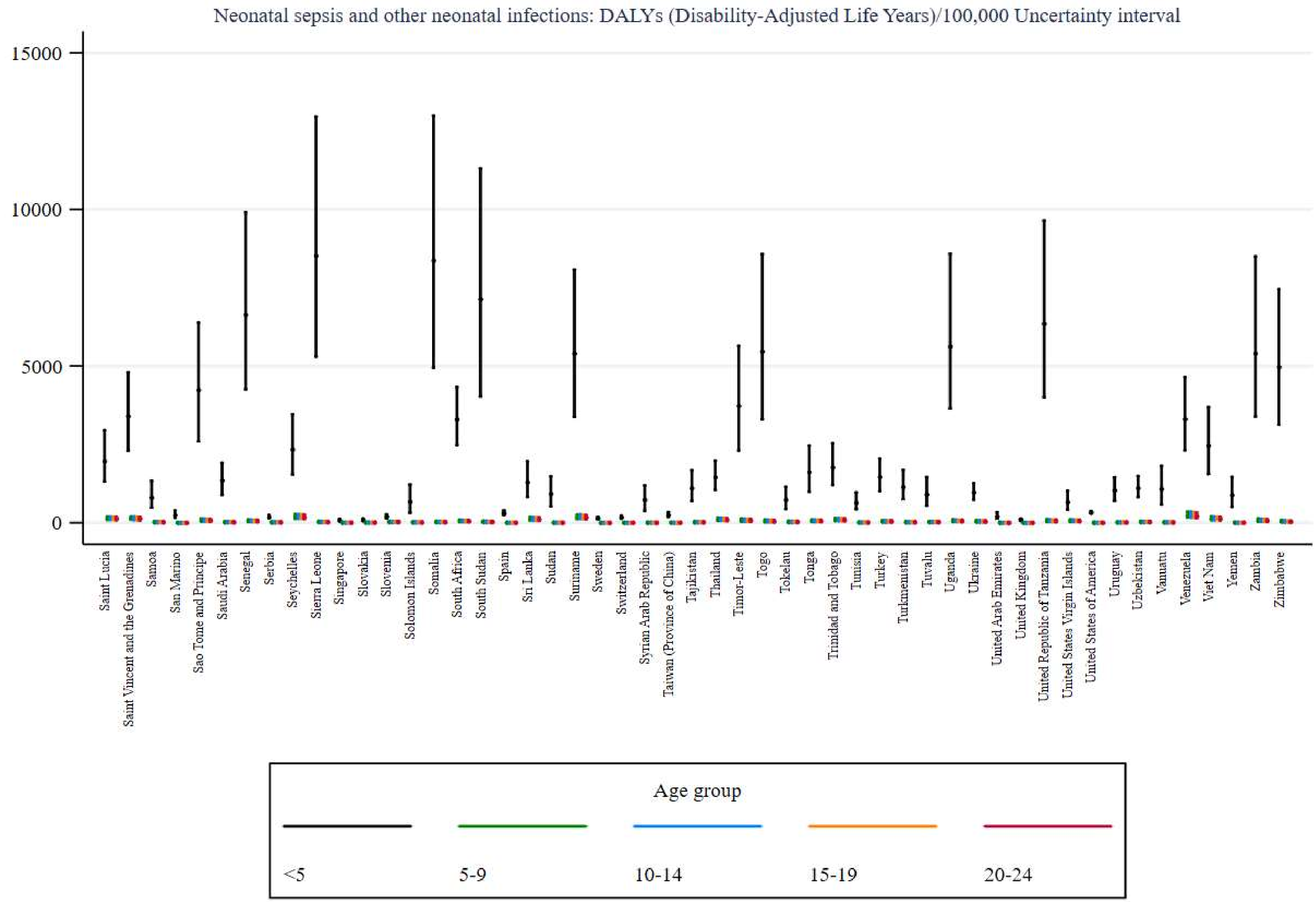
Neonatal sepsis and other neonatal infections: DALYs (Disability-Adjusted Life Years)/100,000 Uncertainty interval
15000
10000
5000
0
Saint Lucia
Saint Vincent and the Grenadines
Samoa
San Marino
Sao Tome and Principe
Saudi Arabia
Senegal
Serbia
Seychelles
Sierra Leone
Singapore
Slovakia
Slovenia
Solomon Islands
Somalia
South Africa
South Sudan
Spain
Sri Lanka
Sudan
Suriname
Sweden
Switzerland
Syrian Arab Republic
Taiwan (Province of China)
Tajikistan
Thailand
Timor-Leste
Togo
Tokelau
Tonga
Trinidad and Tobago
Tunisia
Turkey
Turkmenistan
Tuvalu
Uganda
Ukraine
United Arab Emirates
United Kingdom
United Republic of Tanzania
United States Virgin Islands
United States of America
Uruguay
Uzbekistan
Vanuatu
Venezuela
Viet Nam
Yemen
Zambia
Zimbabwe
Age group
<5
5-9
10-14
15-19
20-24

S20_7 Part A: Other unspecified infectious diseases Deaths/ 100 000 Uncertainty interval for each age group

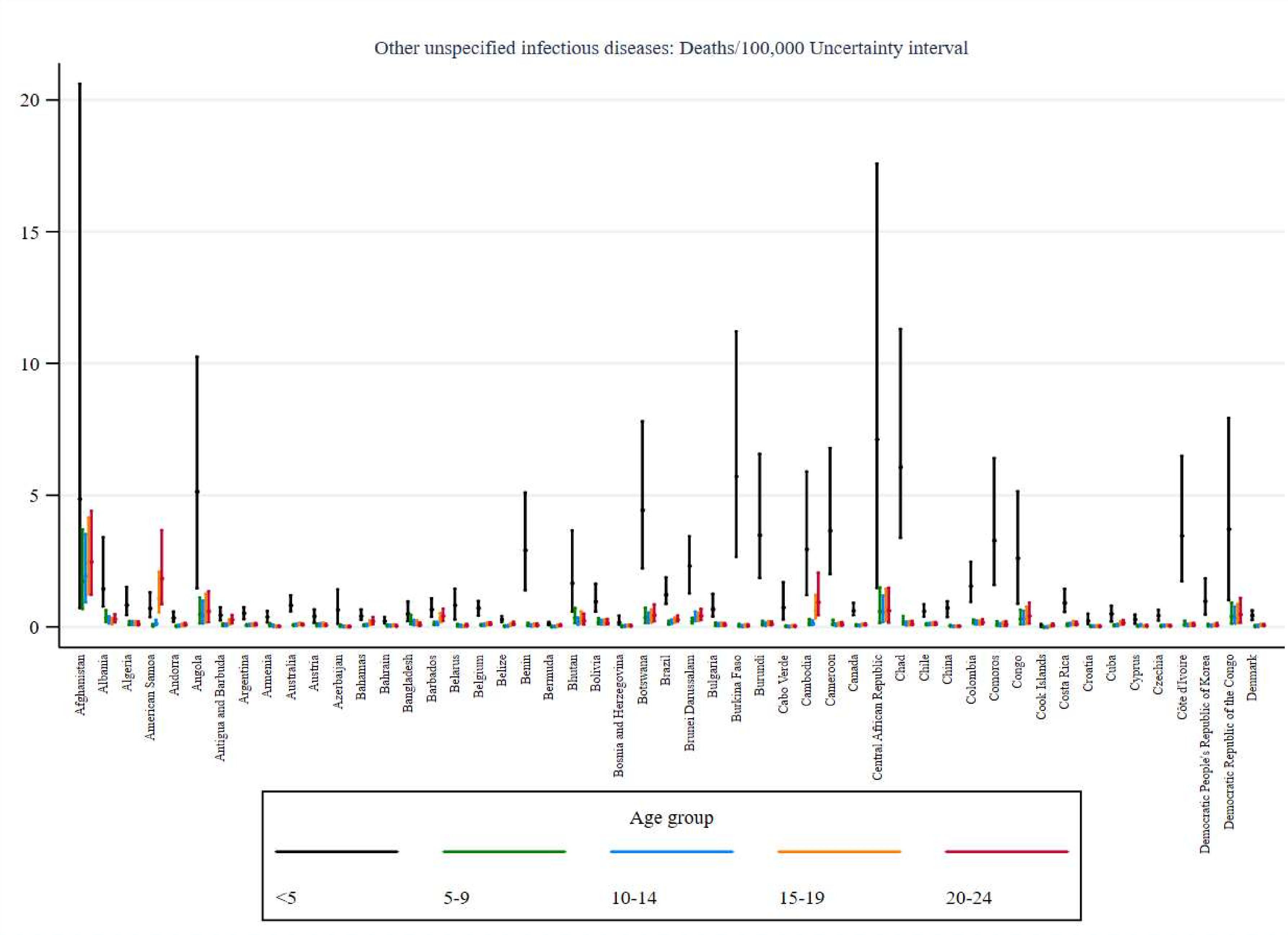

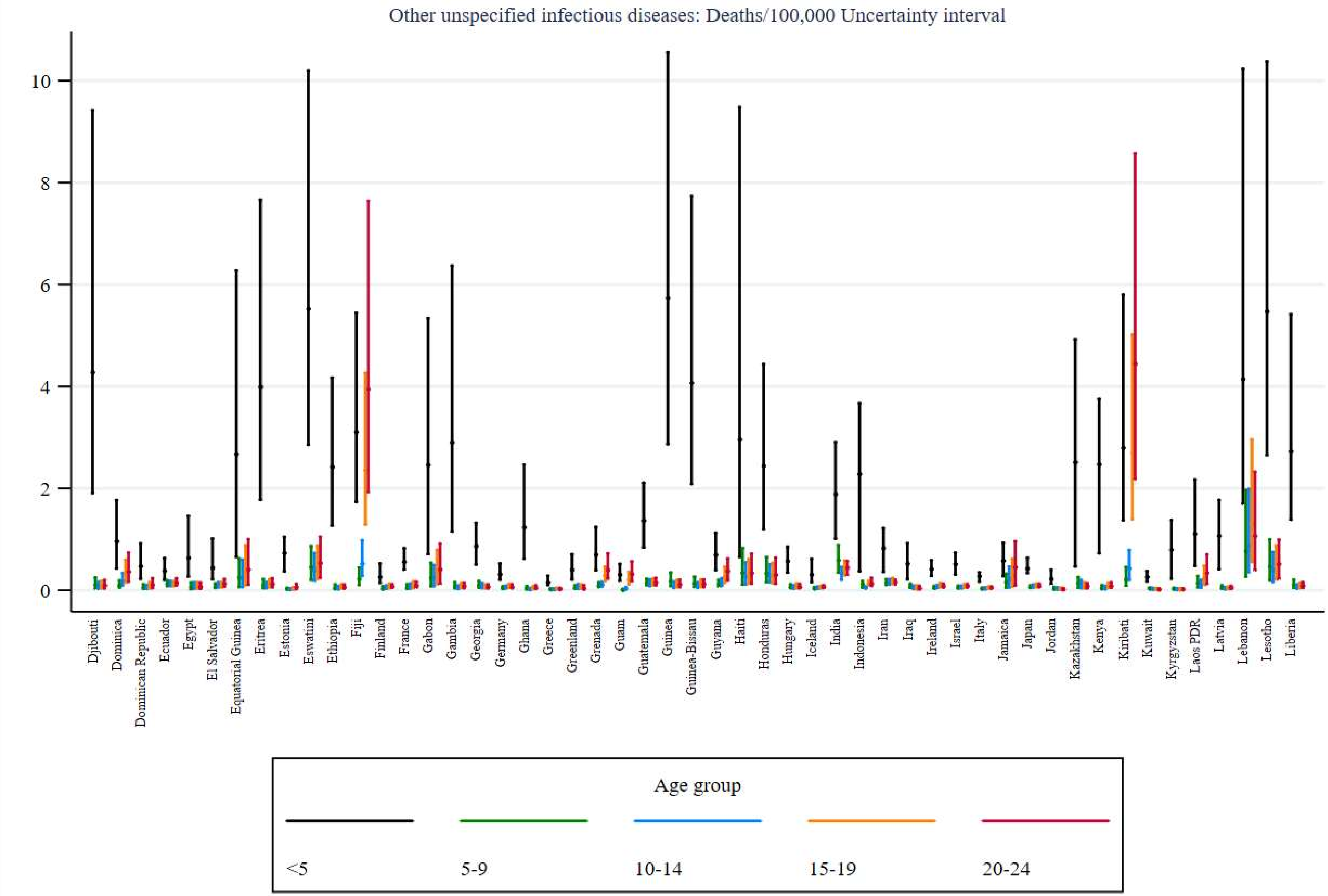

Other unspecified infectious diseases: Deaths/100,000 Uncertainty interval
0
2
4
6
8
10
Djibouti
Dominica
Dominican Republic
Ecuador
Egypt
El Salvador
Equatorial Guinea
Eritrea
Estonia
Eswatini
Ethiopia
Fiji
Finland
France
Gabon
Gambia
Georgia
Germany
Ghana
Greece
Greenland
Grenada
Guam
Guatemala
Guinea
Guinea-Bissau
Guyana
Haiti
Honduras
Hungary
Iceland
India
Indonesia
Iran
Iraq
Ireland
Israel
Italy
Jamaica
Japan
Jordan
Kazakhstan
Kenya
Kiribati
Kuwait
Kyrgyzstan
Laos PDR
Latvia
Lebanon
Lesotho
Liberia
Age group
<5
5-9
10-14
15-19
20-24

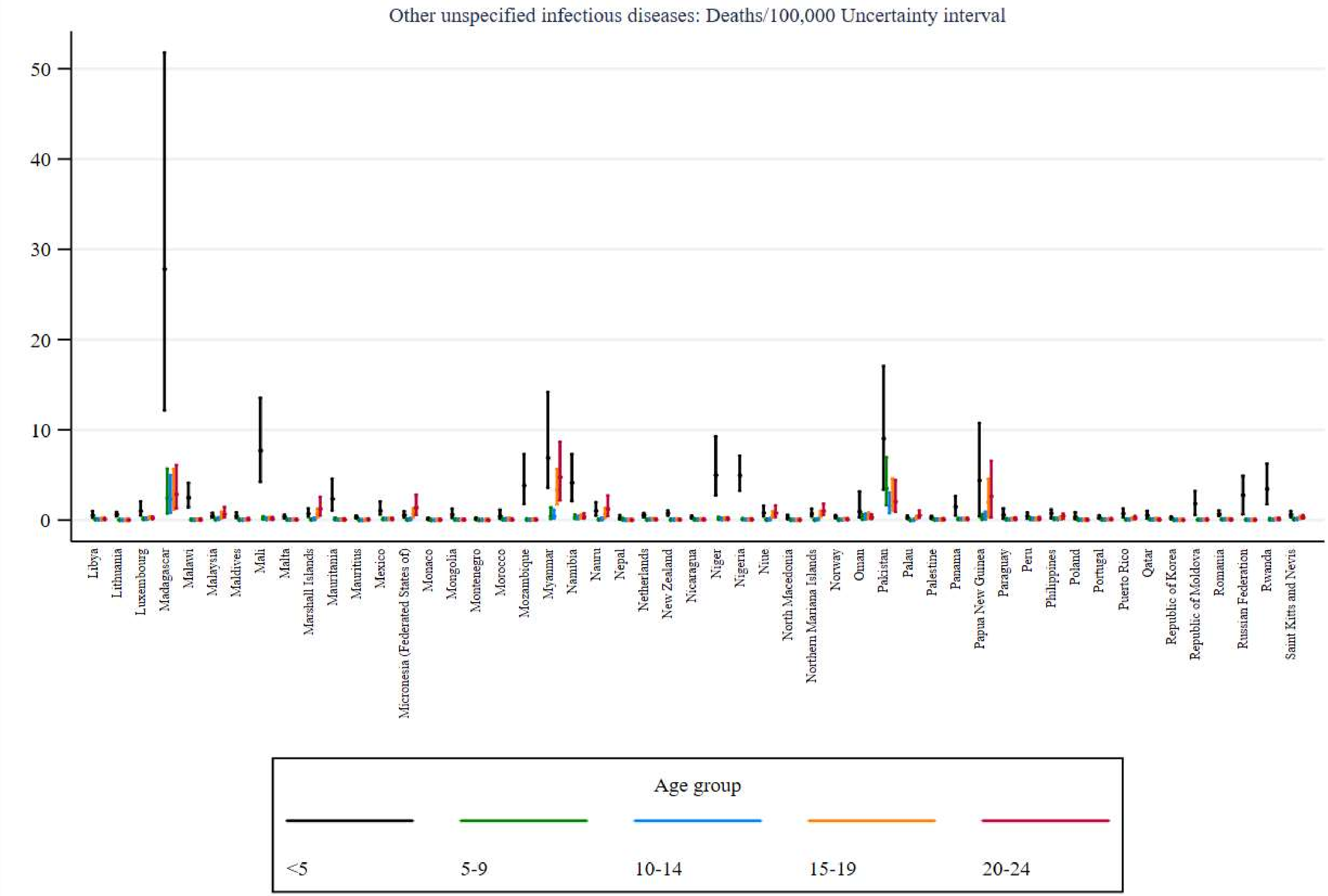
Other unspecified infectious diseases: Deaths/100,000 Uncertainty interval
50
40
30
20
10
0
Libya
Lithuania
Luxembourg
Madagascar
Malawi
Malaysia
Maldives
Mali
Malta
Marshall Islands
Mauritania
Mauritius
Mexico
Micronesia (Federated States of)
Monaco
Mongolia
Montenegro
Morocco
Mozambique
Myanmar
Namibia
Nauru
Nepal
Netherlands
New Zealand
Nicaragua
Niger
Nigeria
Niue
North Macedonia
Northern Mariana Islands
Norway
Oman
Pakistan
Palau
Palestine
Panama
Papua New Guinea
Paraguay
Peru
Philippines
Poland
Portugal
Puerto Rico
Qatar
Republic of Korea
Republic of Moldova
Romania
Russian Federation
Rwanda
Saint Kitts and Nevis
Age group
<5
5-9
10-14
15-19
20-24

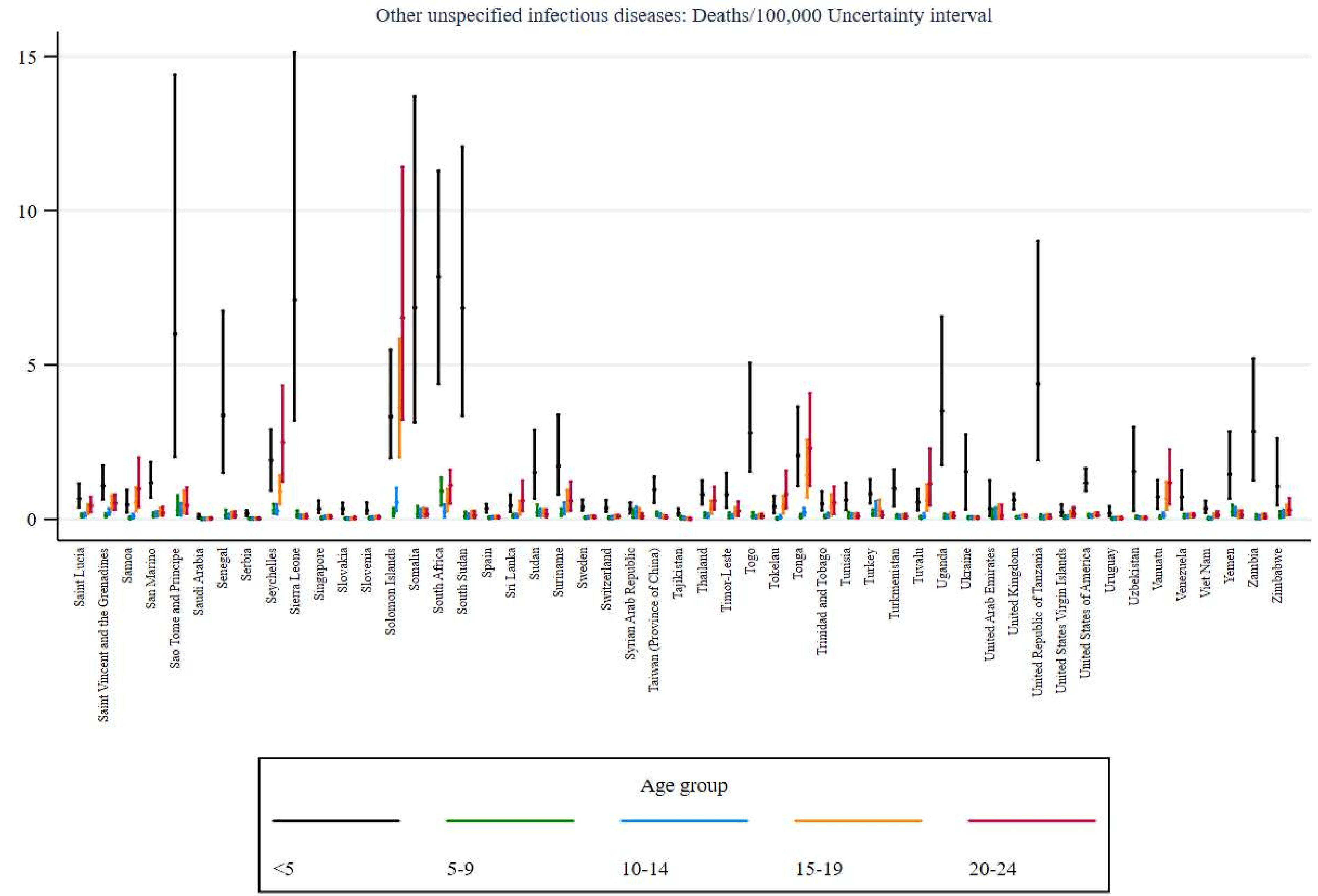

Other unspecified infectious diseases: Deaths/100,000 Uncertainty interval
15
10
5
0
Saint Lucia
Saint Vincent and the Grenadines
Samoa
San Marino
Sao Tome and Principe
Saudi Arabia
Senegal
Serbia
Seychelles
Sierra Leone
Singapore
Slovakia
Slovenia
Solomon Islands
Somalia
South Africa
South Sudan
Spain
Sri Lanka
Sudan
Suriname
Sweden
Switzerland
Syrian Arab Republic
Taiwan (Province of China)
Tajikistan
Thailand
Timor-Leste
Togo
Tokelau
Tonga
Trinidad and Tobago
Tunisia
Turkey
Turkmenistan
Tuvalu
Uganda
Ukraine
United Arab Emirates
United Kingdom
United Republic of Tanzania
United States Virgin Islands
United States of America
Uruguay
Uzbekistan
Vanuatu
Venezuela
Viet Nam
Yemen
Zambia
Zimbabwe
Age group
<5
5-9
10-14
15-19
20-24

S20_7 Part B: Other unspecified infectious diseases DALYs/ 100 000 Uncertainty interval for each age group

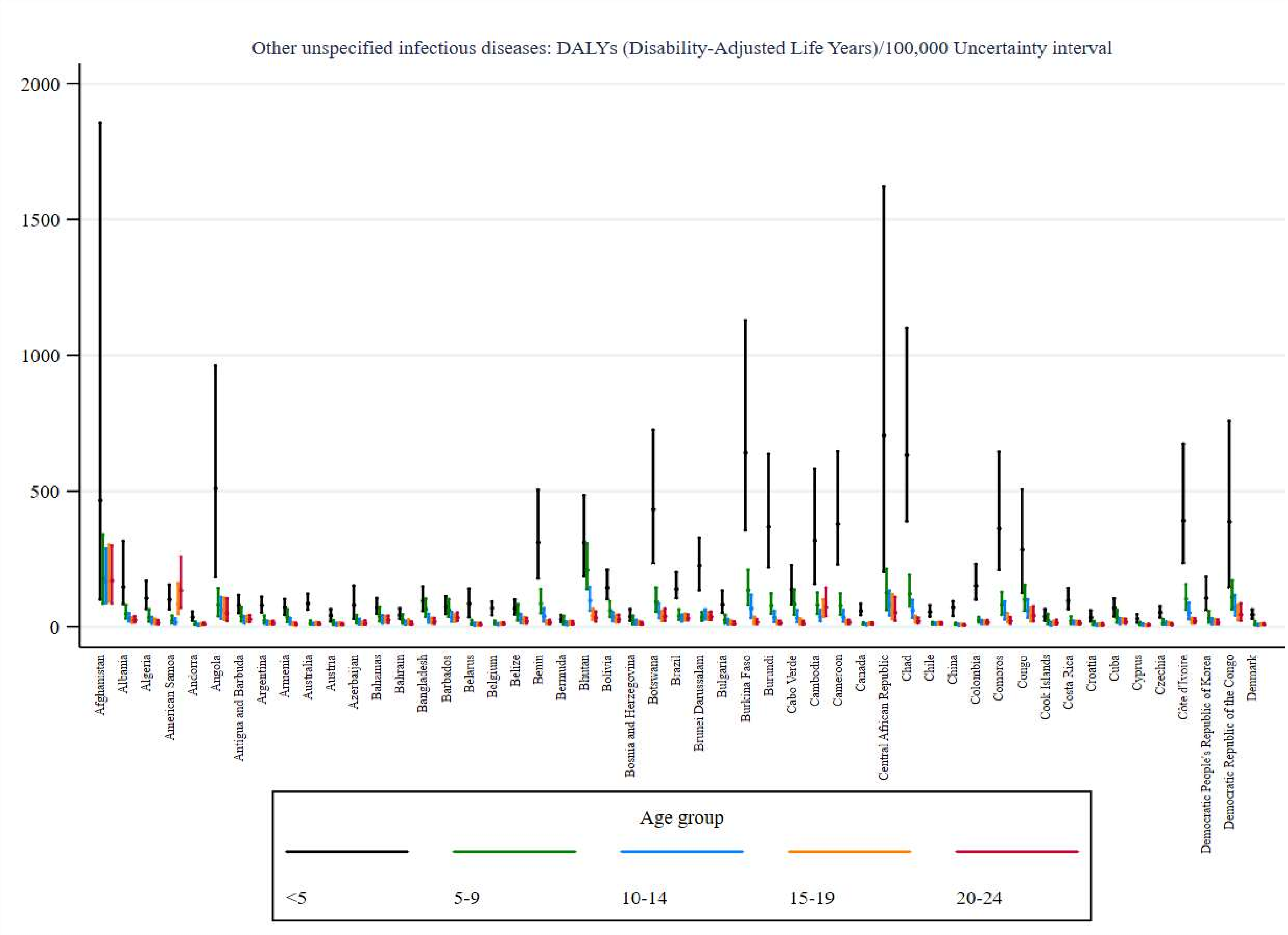

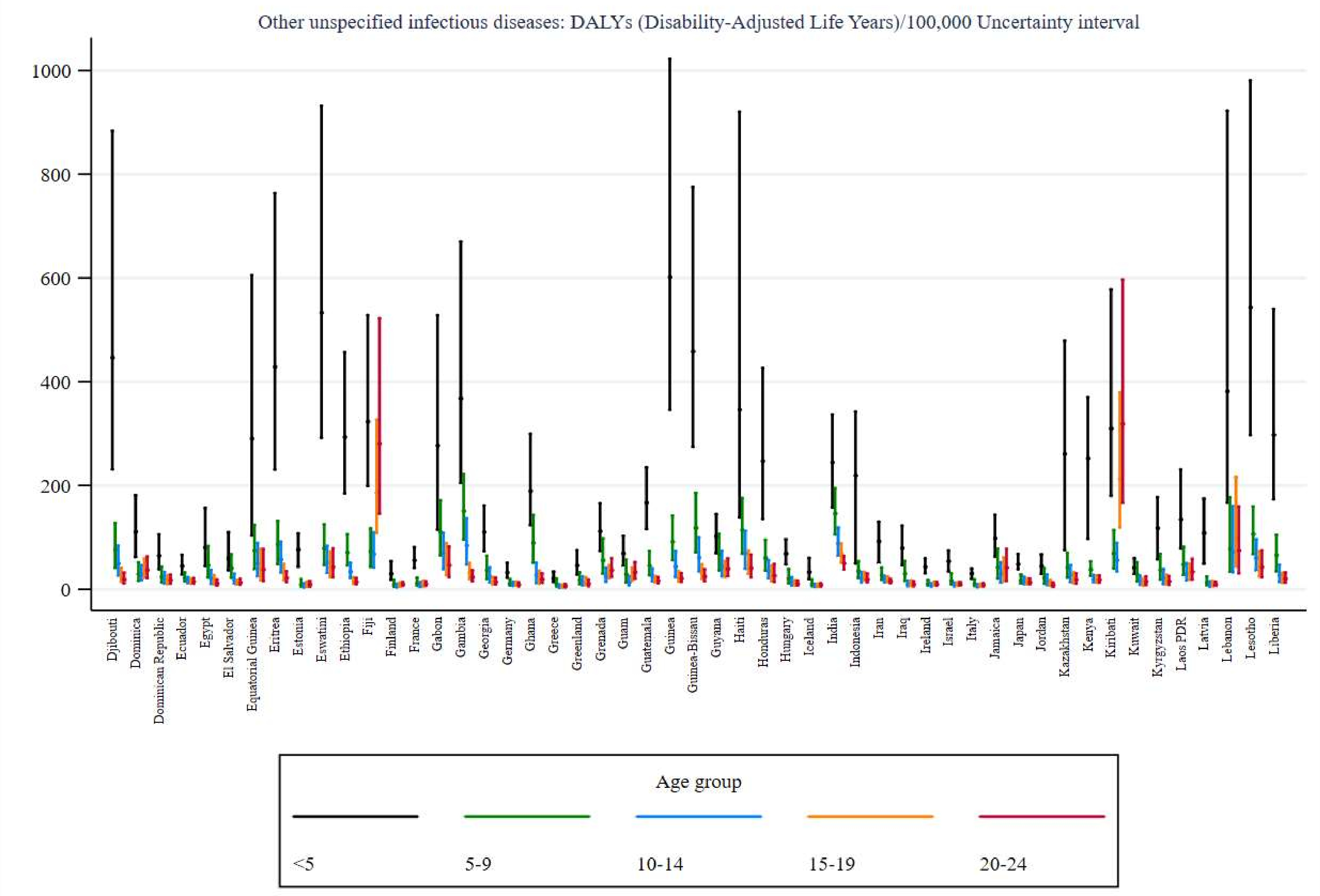

Other unspecified infectious diseases: DALYs (Disability-Adjusted Life Years)/100,000 Uncertainty interval
1000
800
600
400
200
0
Djibouti
Dominica
Dominican Republic
Ecuador
Egypt
El Salvador
Equatorial Guinea
Eritrea
Estonia
Eswatini
Ethiopia
Fiji
Finland
France
Gabon
Gambia
Georgia
Germany
Ghana
Greece
Greenland
Grenada
Guam
Guatemala
Guinea
Guinea-Bissau
Guyana
Haiti
Honduras
Hungary
Iceland
India
Indonesia
Iran
Iraq
Ireland
Israel
Italy
Jamaica
Japan
Jordan
Kazakhstan
Kenya
Kiribati
Kuwait
Kyrgyzstan
Laos PDR
Latvia
Lebanon
Lesotho
Liberia
Age group
<5
5-9
10-14
15-19
20-24

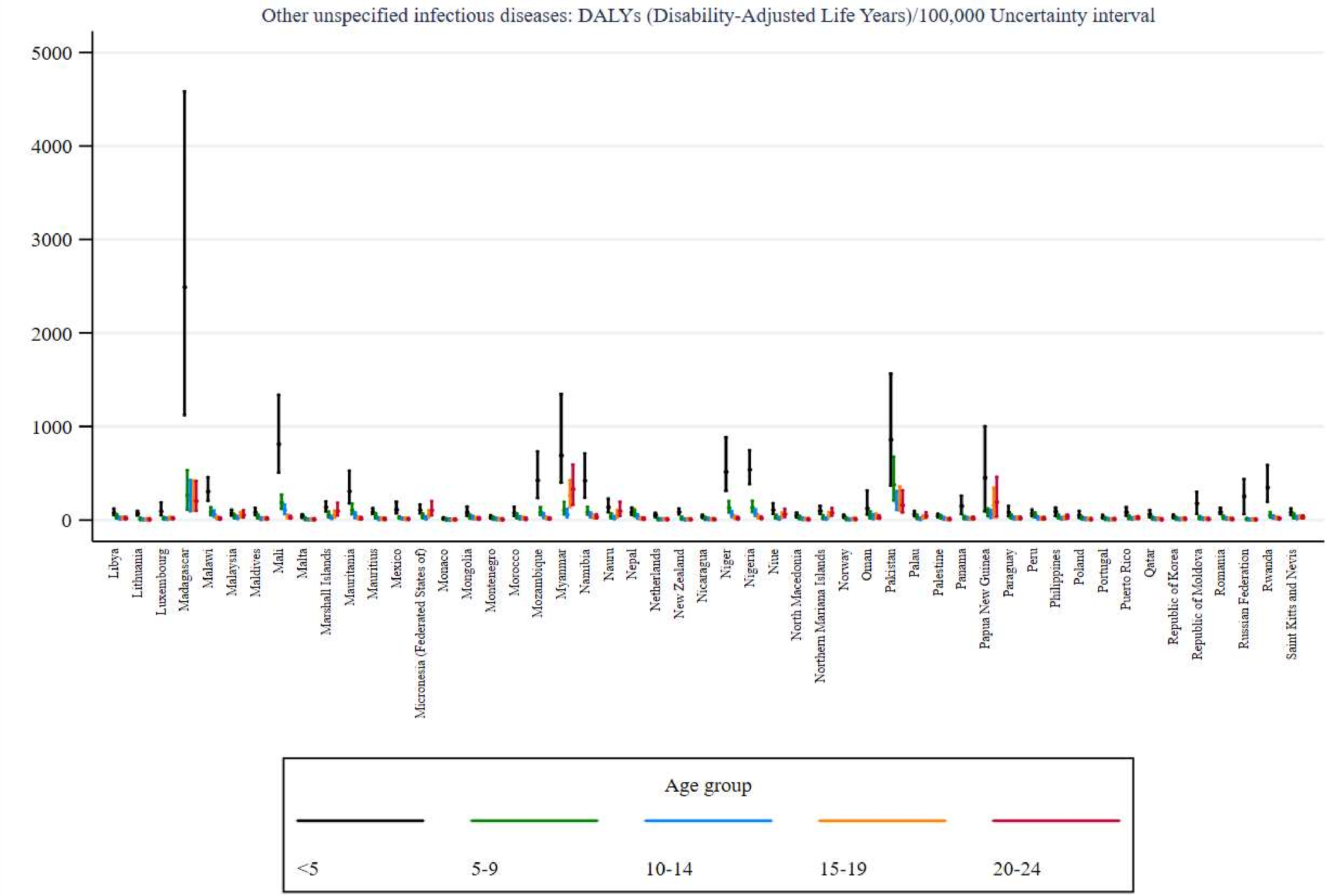

Other unspecified infectious diseases: DALYs (Disability-Adjusted Life Years)/100,000 Uncertainty interval
5000
4000
3000
2000
1000
0
Libya
Lithuania
Luxembourg
Madagascar
Malawi
Malaysia
Maldives
Mali
Malta
Marshall Islands
Mauritania
Mauritius
Mexico
Micronesia (Federated States of)
Monaco
Mongolia
Montenegro
Morocco
Mozambique
Myanmar
Namibia
Nauru
Nepal
Netherlands
New Zealand
Nicaragua
Niger
Nigeria
Niue
North Macedonia
Northern Mariana Islands
Norway
Oman
Pakistan
Palau
Palestine
Panama
Papua New Guinea
Paraguay
Peru
Philippines
Poland
Portugal
Puerto Rico
Qatar
Republic of Korea
Republic of Moldova
Romania
Russian Federation
Rwanda
Saint Kitts and Nevis
Age group
<5
5-9
10-14
15-19
20-24

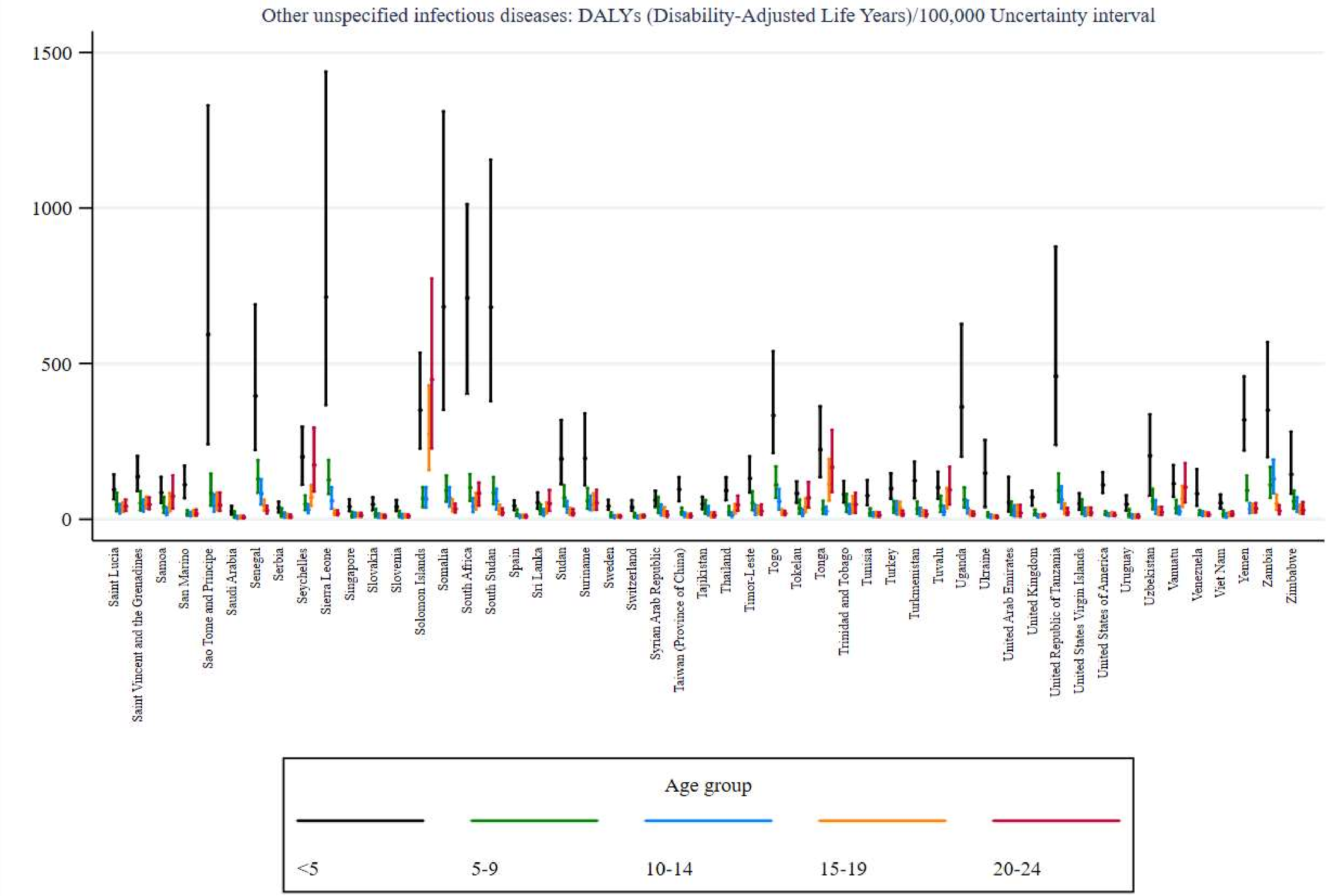
Other unspecified infectious diseases: DALYs (Disability-Adjusted Life Years)/100,000 Uncertainty interval
1500
1000
500
0
Saint Lucia
Saint Vincent and the Grenadines
Samoa
San Marino
Sao Tome and Principe
Saudi Arabia
Senegal
Serbia
Seychelles
Sierra Leone
Singapore
Slovakia
Slovenia
Solomon Islands
Somalia
South Africa
South Sudan
Spain
Sri Lanka
Sudan
Suriname
Sweden
Switzerland
Syrian Arab Republic
Taiwan (Province of China)
Tajikistan
Thailand
Timor-Leste
Togo
Tokelau
Tonga
Trinidad and Tobago
Tunisia
Turkey
Turkmenistan
Tuvalu
Uganda
Ukraine
United Arab Emirates
United Kingdom
United Republic of Tanzania
United States Virgin Islands
United States of America
Uruguay
Uzbekistan
Vanuatu
Venezuela
Viet Nam
Yemen
Zambia
Zimbabwe
Age group
<5
5-9
10-14
15-19
20-24

S20_8 Part A: Rheumatic heart disease Deaths/ 100 000 Uncertainty interval for each age group

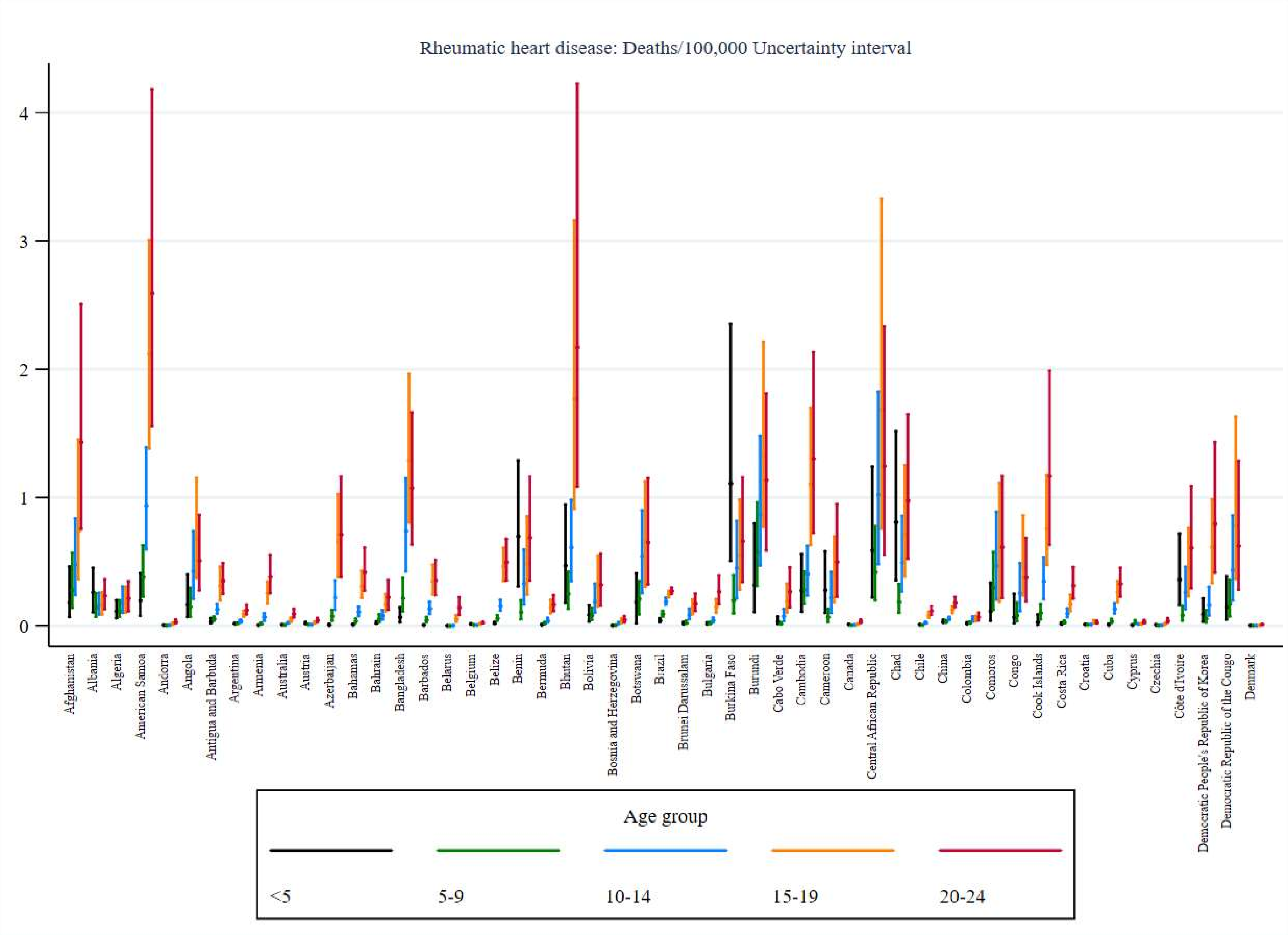

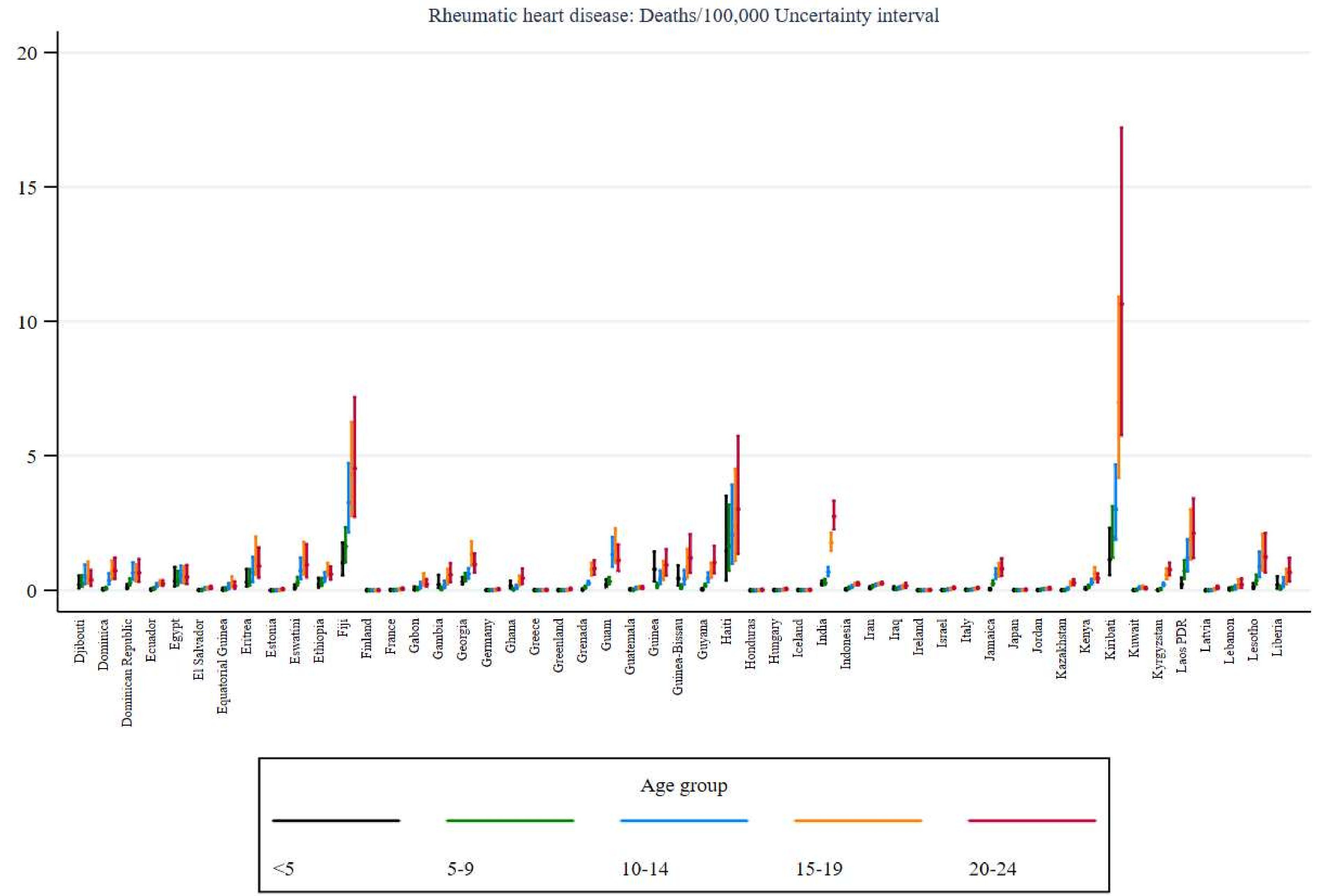

Rheumatic heart disease: Deaths/100,000 Uncertainty interval
20
15
10
5
0
Djibouti
Dominica
Dominican Republic
Ecuador
Egypt
El Salvador
Equatorial Guinea
Eritrea
Estonia
Eswatini
Ethiopia
Fiji
Finland
France
Gabon
Gambia
Georgia
Germany
Ghana
Greece
Greenland
Grenada
Guam
Guatemala
Guinea
Guinea-Bissau
Guyana
Haiti
Honduras
Hungary
Iceland
India
Indonesia
Iran
Iraq
Ireland
Israel
Italy
Jamaica
Japan
Jordan
Kazakhstan
Kenya
Kiribati
Kuwait
Kyrgyzstan
Laos PDR
Latvia
Lebanon
Lesotho
Liberia
Age group
<5
5-9
10-14
15-19
20-24

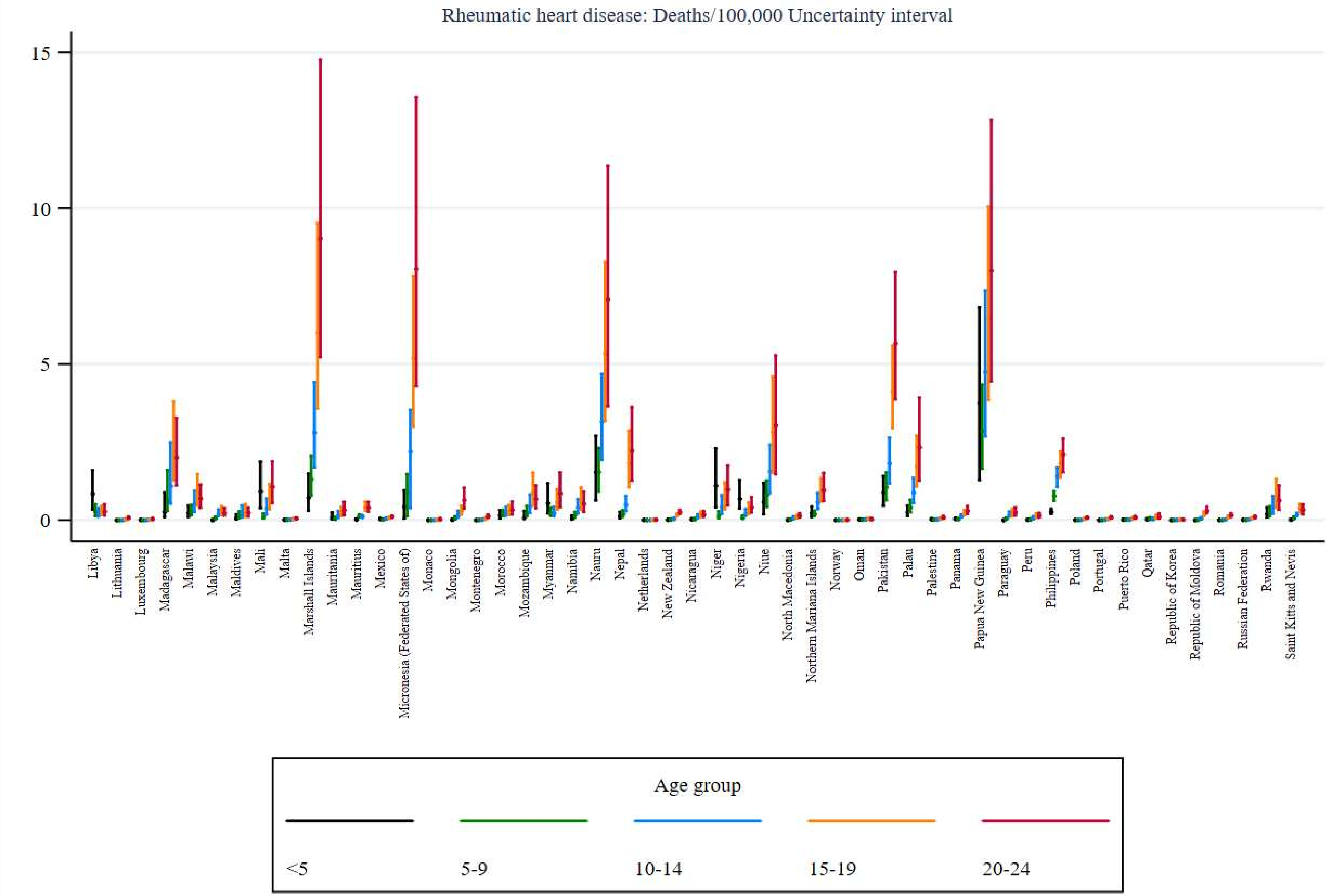

Rheumatic heart disease: Deaths/100,000 Uncertainty interval
15
10
5
0
Libya
Lithuania
Luxembourg
Madagascar
Malawi
Malaysia
Maldives
Mali
Malta
Marshall Islands
Mauritania
Mauritius
Mexico
Micronesia (Federated States of)
Monaco
Mongolia
Montenegro
Morocco
Mozambique
Myanmar
Namibia
Nauru
Nepal
Netherlands
New Zealand
Nicaragua
Niger
Nigeria
Niue
North Macedonia
Northern Mariana Islands
Norway
Oman
Pakistan
Palau
Palestine
Panama
Papua New Guinea
Paraguay
Peru
Philippines
Poland
Portugal
Puerto Rico
Qatar
Republic of Korea
Republic of Moldova
Romania
Russian Federation
Rwanda
Saint Kitts and Nevis
Age group
<5
5-9
10-14
15-19
20-24

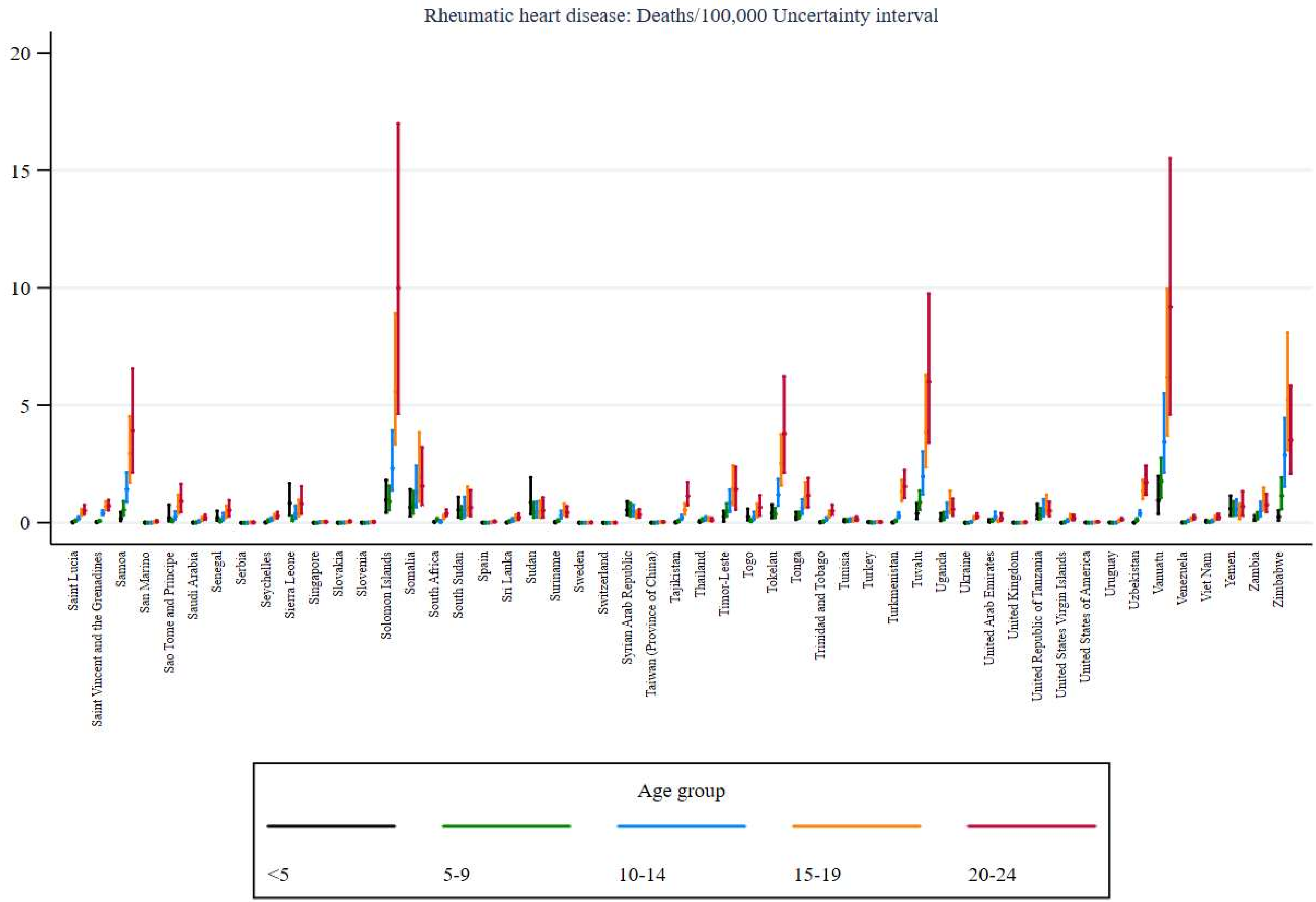
Rheumatic heart disease: Deaths/100,000 Uncertainty interval
20
15
10
5
0
Saint Lucia
Saint Vincent and the Grenadines
Samoa
San Marino
Sao Tome and Principe
Saudi Arabia
Senegal
Serbia
Seychelles
Sierra Leone
Singapore
Slovakia
Slovenia
Solomon Islands
Somalia
South Africa
South Sudan
Spain
Sri Lanka
Sudan
Suriname
Sweden
Switzerland
Syrian Arab Republic
Taiwan (Province of China)
Tajikistan
Thailand
Timor-Leste
Togo
Tokelau
Tonga
Trinidad and Tobago
Tunisia
Turkey
Turkmenistan
Tuvalu
Uganda
Ukraine
United Arab Emirates
United Kingdom
United Republic of Tanzania
United States Virgin Islands
United States of America
Uruguay
Uzbekistan
Vanuatu
Venezuela
Viet Nam
Yemen
Zambia
Zimbabwe
Age group
<5
5-9
10-14
15-19
20-24

S20_8 Part B: Rheumatic heart disease DALYs/ 100 000 Uncertainty interval for each age group

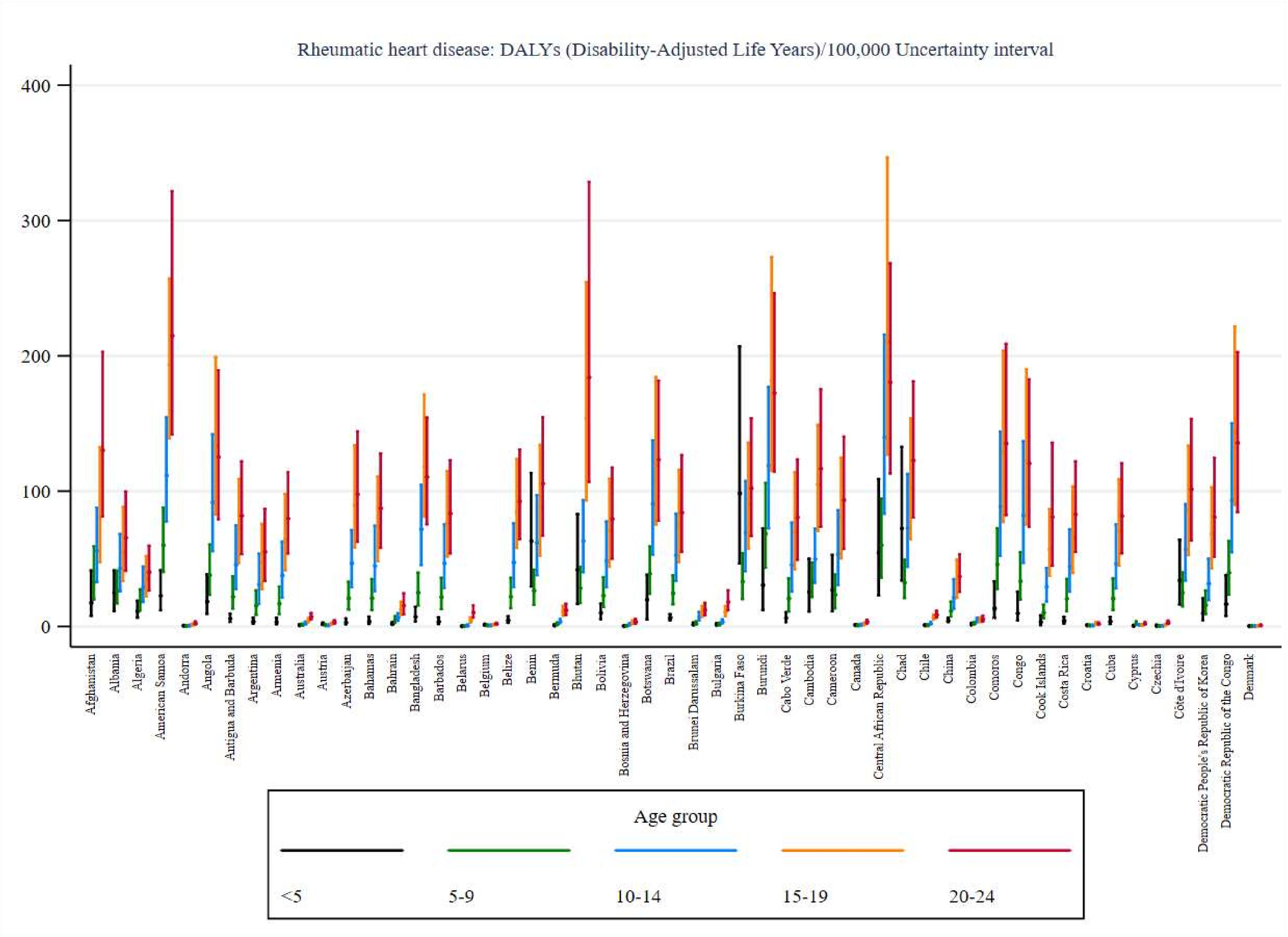

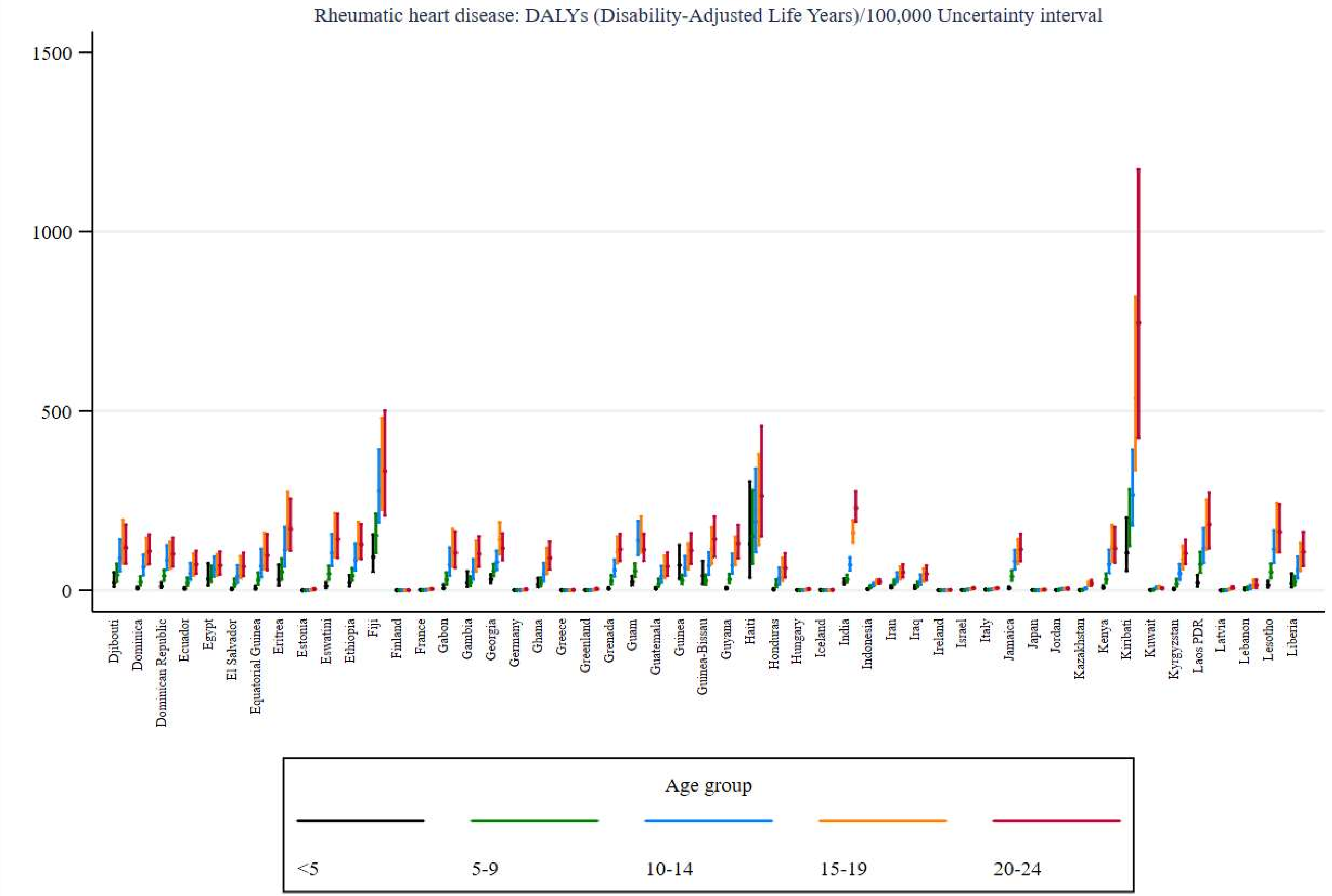

Rheumatic heart disease: DALYs (Disability-Adjusted Life Years)/100,000 Uncertainty interval
1500
1000
500
0
Djibouti
Dominica
Dominican Republic
Ecuador
Egypt
El Salvador
Equatorial Guinea
Eritrea
Estonia
Eswatini
Ethiopia
Fiji
Finland
France
Gabon
Gambia
Georgia
Germany
Ghana
Greece
Greenland
Grenada
Guam
Guatemala
Guinea
Guinea-Bissau
Guyana
Haiti
Honduras
Hungary
Iceland
India
Indonesia
Iran
Iraq
Ireland
Israel
Italy
Jamaica
Japan
Jordan
Kazakhstan
Kenya
Kiribati
Kuwait
Kyrgyzstan
Laos PDR
Latvia
Lebanon
Lesotho
Liberia
Age group
<5
5-9
10-14
15-19
20-24

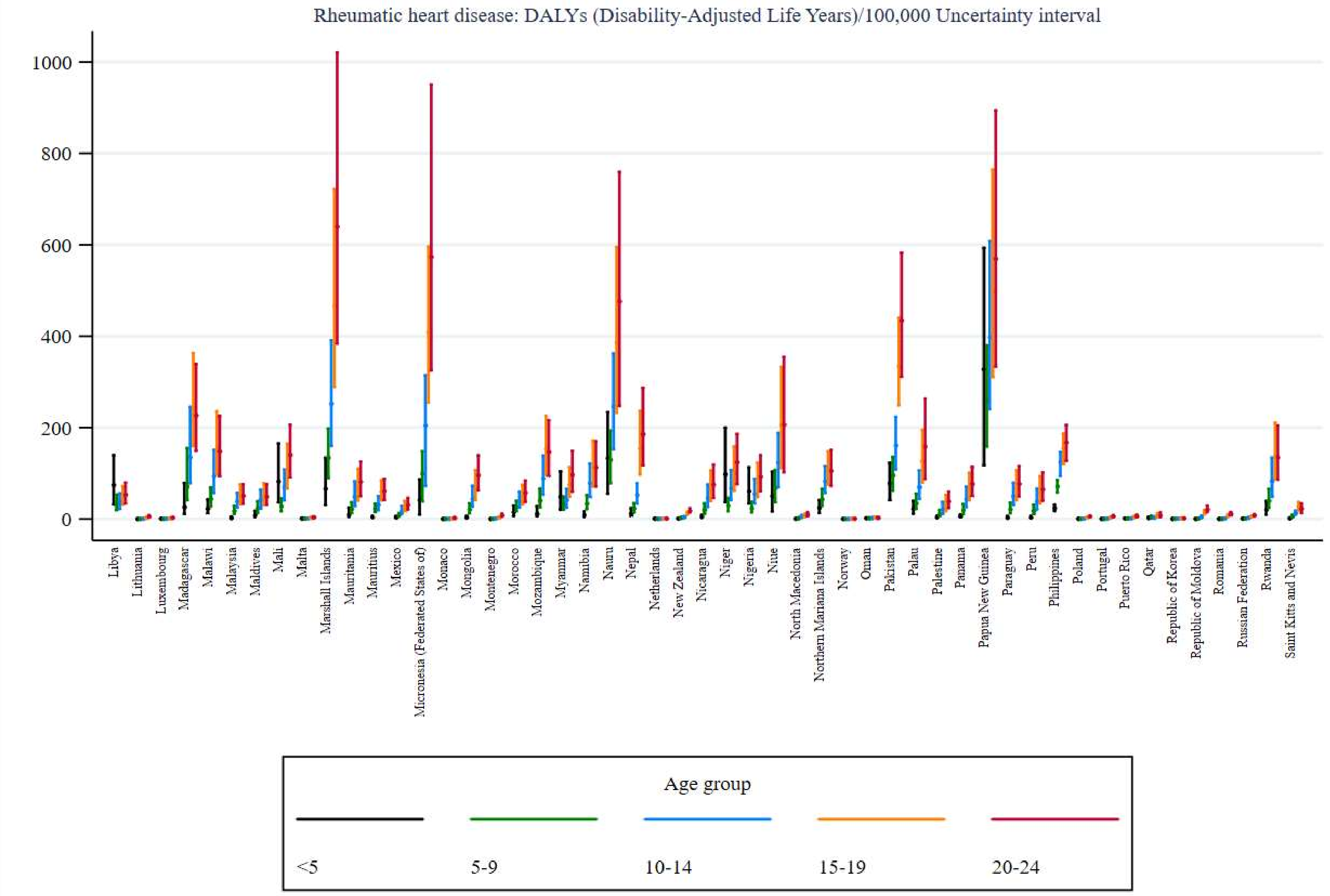

Rheumatic heart disease: DALYs (Disability-Adjusted Life Years)/100,000 Uncertainty interval
1000
800
600
400
200
0
Libya
Lithuania
Luxembourg
Madagascar
Malawi
Malaysia
Maldives
Mali
Malta
Marshall Islands
Mauritania
Mauritius
Mexico
Micronesia (Federated States of)
Monaco
Mongolia
Montenegro
Morocco
Mozambique
Myanmar
Namibia
Nauru
Nepal
Netherlands
New Zealand
Nicaragua
Niger
Nigeria
Niue
North Macedonia
Northern Mariana Islands
Norway
Oman
Pakistan
Palau
Palestine
Panama
Papua New Guinea
Paraguay
Peru
Philippines
Poland
Portugal
Puerto Rico
Qatar
Republic of Korea
Republic of Moldova
Romania
Russian Federation
Rwanda
Saint Kitts and Nevis
Age group
<5
5-9
10-14
15-19
20-24

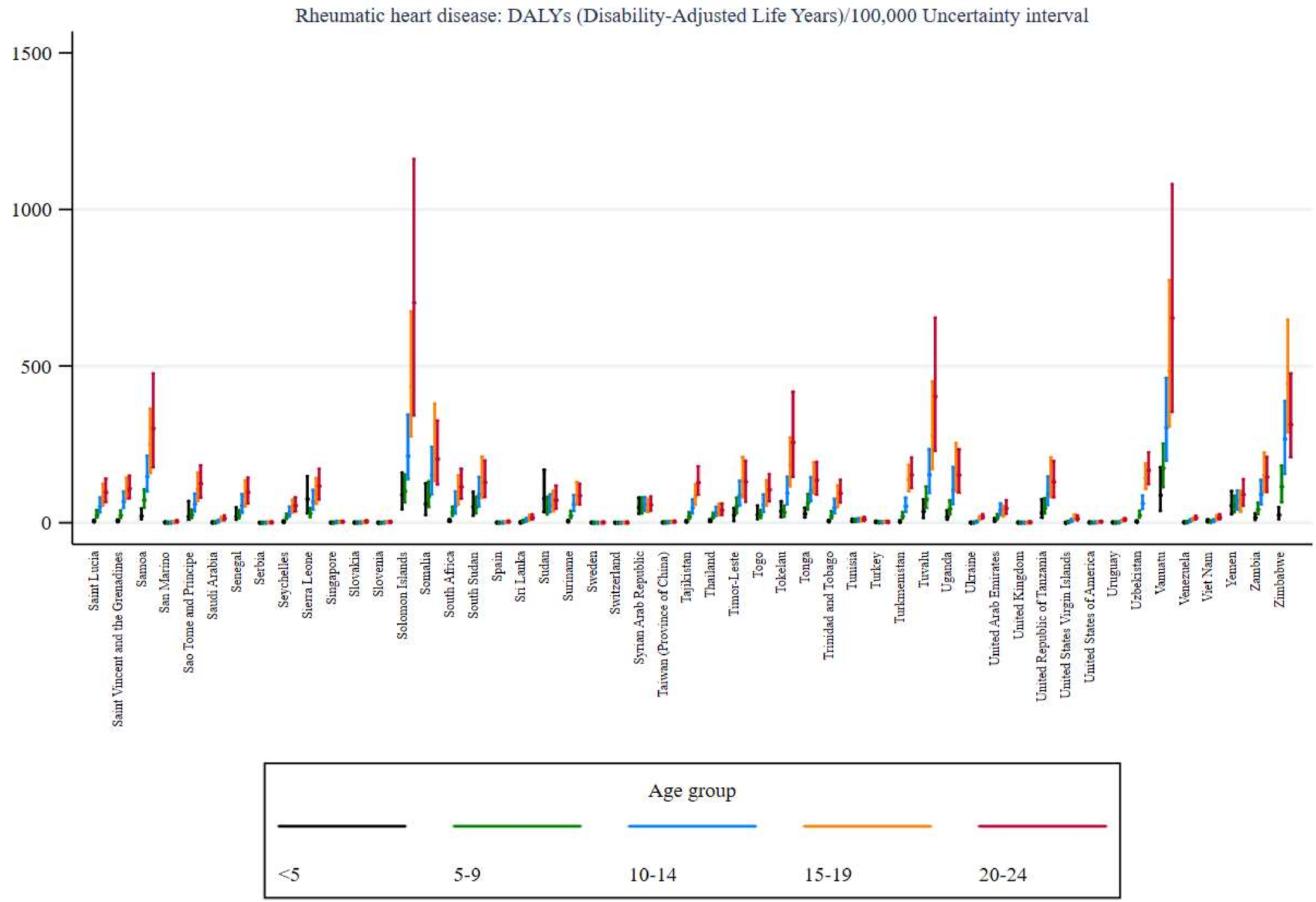
Rheumatic heart disease: DALYs (Disability-Adjusted Life Years)/100,000 Uncertainty interval
1500
1000
500
0
Saint Lucia
Saint Vincent and the Grenadines
Samoa
San Marino
Sao Tome and Principe
Saudi Arabia
Senegal
Serbia
Seychelles
Sierra Leone
Singapore
Slovakia
Slovenia
Solomon Islands
Somalia
South Africa
South Sudan
Spain
Sri Lanka
Sudan
Suriname
Sweden
Switzerland
Syrian Arab Republic
Taiwan (Province of China)
Tajikistan
Thailand
Timor-Leste
Togo
Tokelau
Tonga
Trinidad and Tobago
Tunisia
Turkey
Turkmenistan
Tuvalu
Uganda
Ukraine
United Arab Emirates
United Kingdom
United Republic of Tanzania
United States Virgin Islands
United States of America
Uruguay
Uzbekistan
Vanuatu
Venezuela
Viet Nam
Yemen
Zambia
Zimbabwe
Age group
<5
5-9
10-14
15-19
20-24

S20_9 Part A: Sexually transmitted infections excluding HIV Deaths/ 100 000 Uncertainty interval for each age group

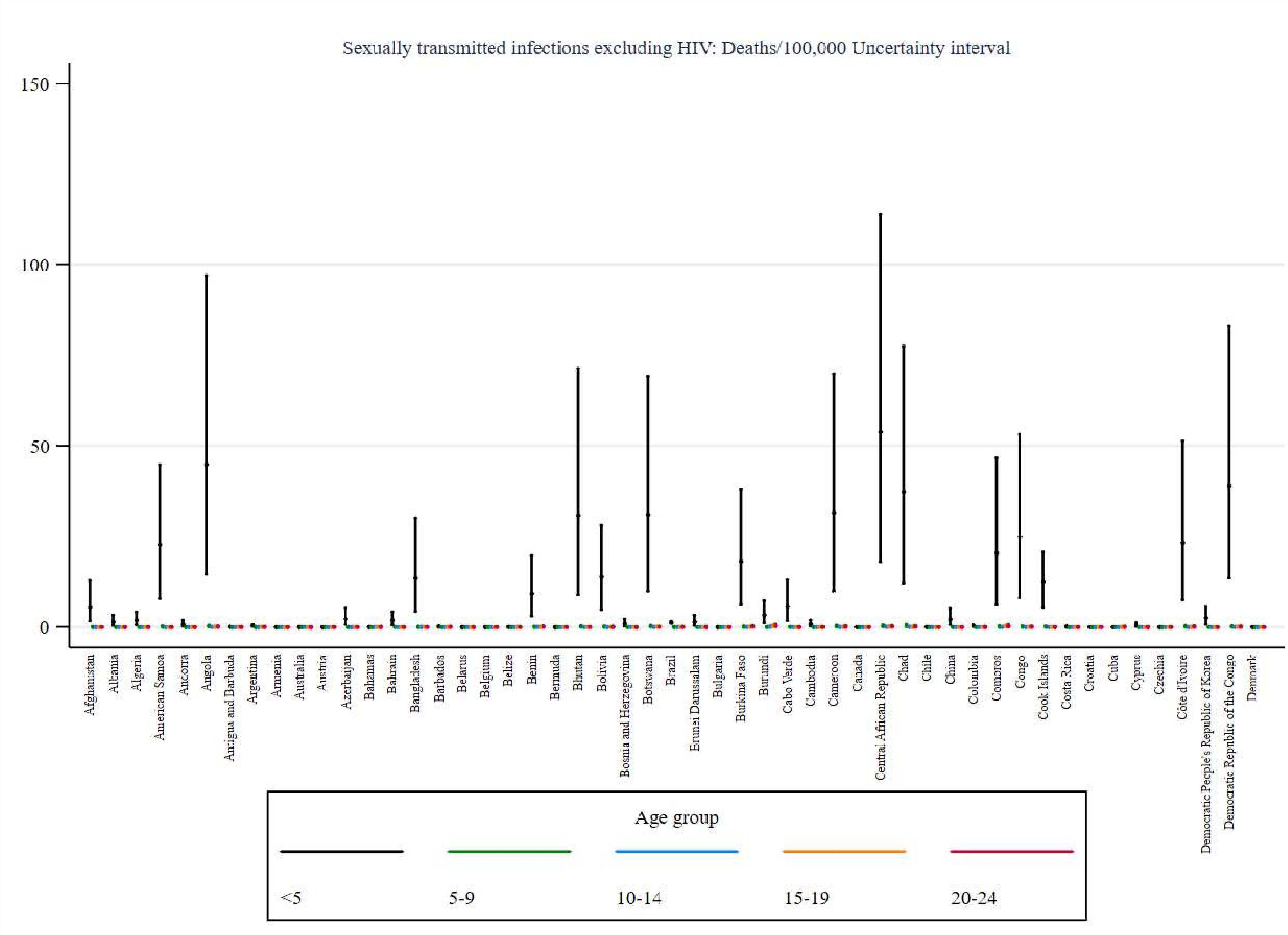

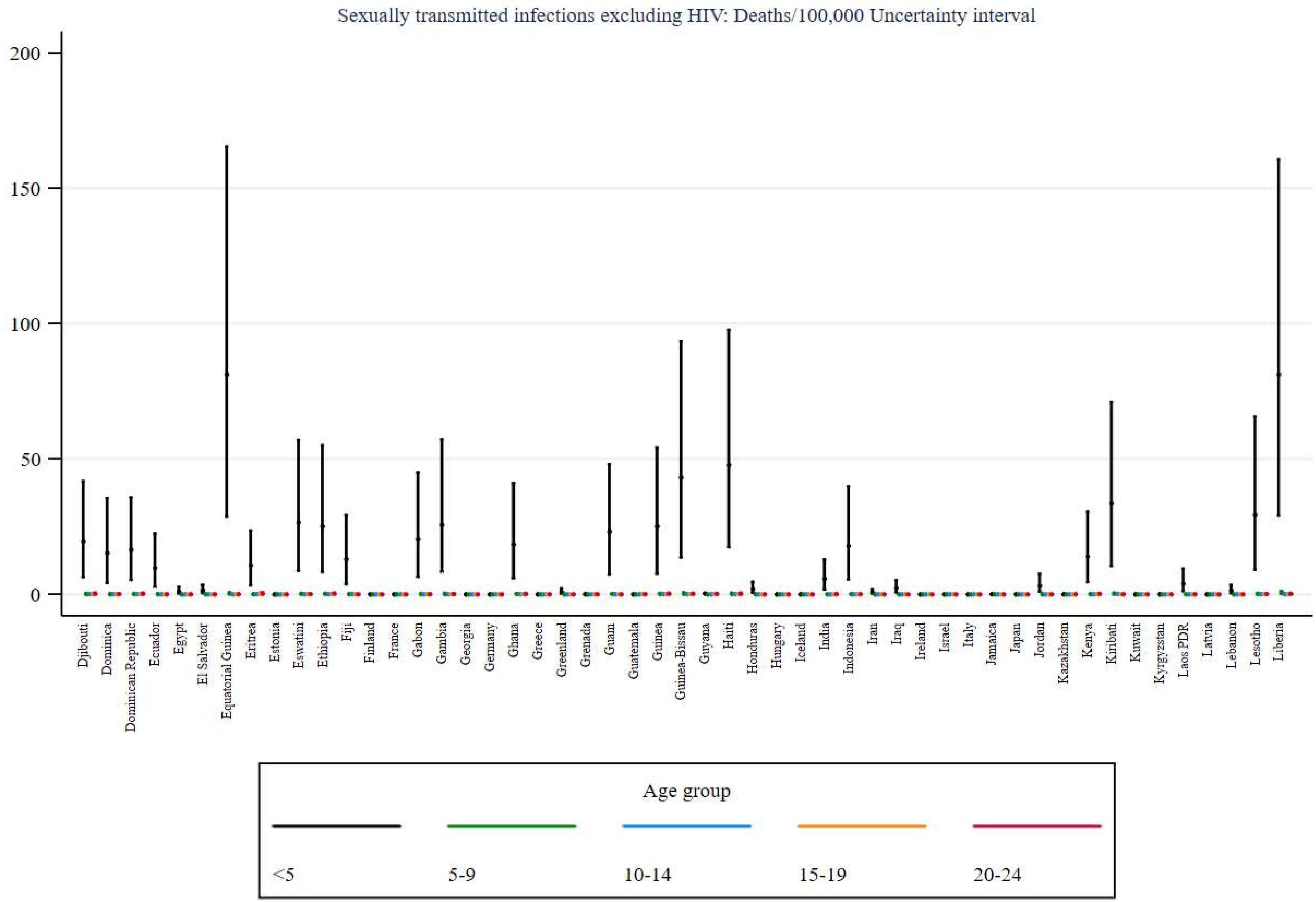

Sexually transmitted infections excluding HIV: Deaths/100,000 Uncertainty interval
200
150
100
50
0
Djibouti
Dominica
Dominican Republic
Ecuador
Egypt
El Salvador
Equatorial Guinea
Eritrea
Estonia
Eswatini
Ethiopia
Fiji
Finland
France
Gabon
Gambia
Georgia
Germany
Ghana
Greece
Greenland
Grenada
Guam
Guatemala
Guinea
Guinea-Bissau
Guyana
Haiti
Honduras
Hungary
Iceland
India
Indonesia
Iran
Iraq
Ireland
Israel
Italy
Jamaica
Japan
Jordan
Kazakhstan
Kenya
Kiribati
Kuwait
Kyrgyzstan
Laos PDR
Latvia
Lebanon
Lesotho
Liberia
Age group
<5
5-9
10-14
15-19
20-24

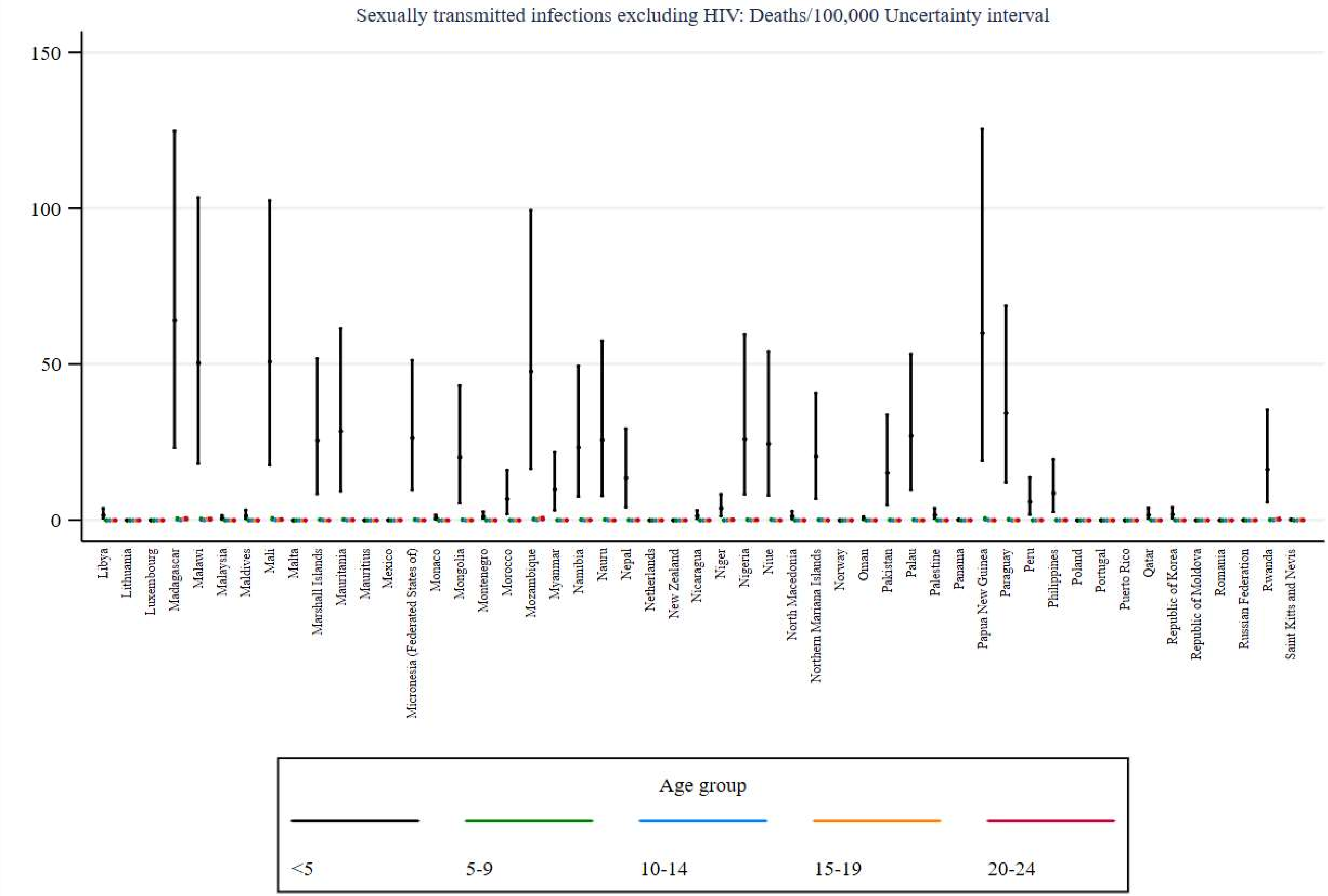
Sexually transmitted infections excluding HIV: Deaths/100,000 Uncertainty interval
150
100
50
0
Libya
Lithuania
Luxembourg
Madagascar
Malawi
Malaysia
Maldives
Mali
Malta
Marshall Islands
Mauritania
Mauritius
Mexico
Micronesia (Federated States of)
Monaco
Mongolia
Montenegro
Morocco
Mozambique
Myanmar
Namibia
Nauru
Nepal
Netherlands
New Zealand
Nicaragua
Niger
Nigeria
Niue
North Macedonia
Northern Mariana Islands
Norway
Oman
Pakistan
Palau
Palestine
Panama
Papua New Guinea
Paraguay
Peru
Philippines
Poland
Portugal
Puerto Rico
Qatar
Republic of Korea
Republic of Moldova
Romania
Russian Federation
Rwanda
Saint Kitts and Nevis
Age group
<5
5-9
10-14
15-19
20-24

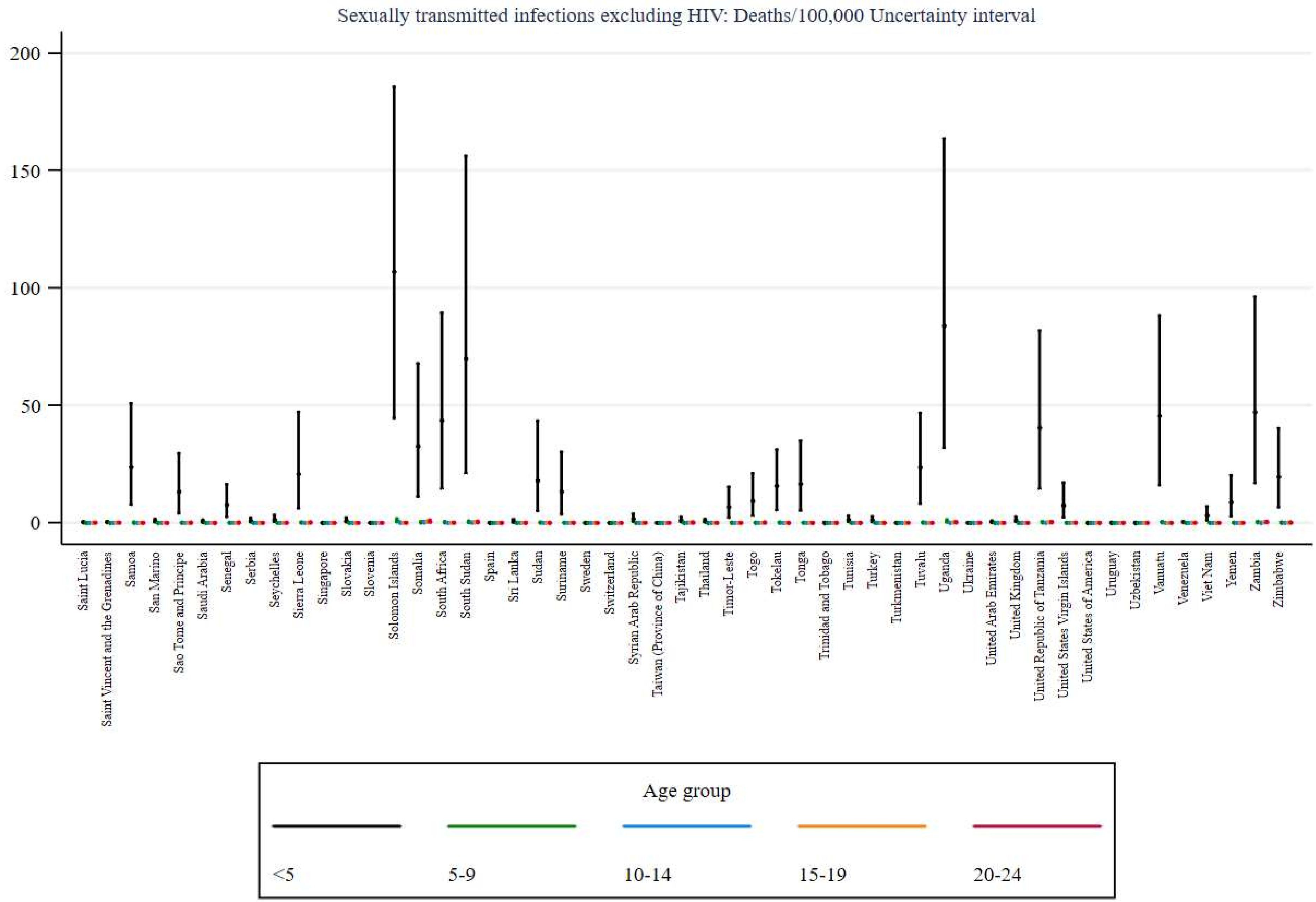
Sexually transmitted infections excluding HIV: Deaths/100,000 Uncertainty interval
200
150
100
50
0
Saint Lucia
Saint Vincent and the Grenadines
Samoa
San Marino
Sao Tome and Principe
Saudi Arabia
Senegal
Serbia
Seychelles
Sierra Leone
Singapore
Slovakia
Slovenia
Solomon Islands
Somalia
South Africa
South Sudan
Spain
Sri Lanka
Sudan
Suriname
Sweden
Switzerland
Syrian Arab Republic
Taiwan (Province of China)
Tajikistan
Thailand
Timor-Leste
Togo
Tokelau
Tonga
Trinidad and Tobago
Tunisia
Turkey
Turkmenistan
Tuvalu
Uganda
Ukraine
United Arab Emirates
United Kingdom
United Republic of Tanzania
United States Virgin Islands
United States of America
Uruguay
Uzbekistan
Vanuatu
Venezuela
Viet Nam
Yemen
Zambia
Zimbabwe
Age group
<5
5-9
10-14
15-19
20-24

S20_9 Part B: Sexually transmitted infections excluding HIV DALYs/ 100 000 Uncertainty interval for each age group

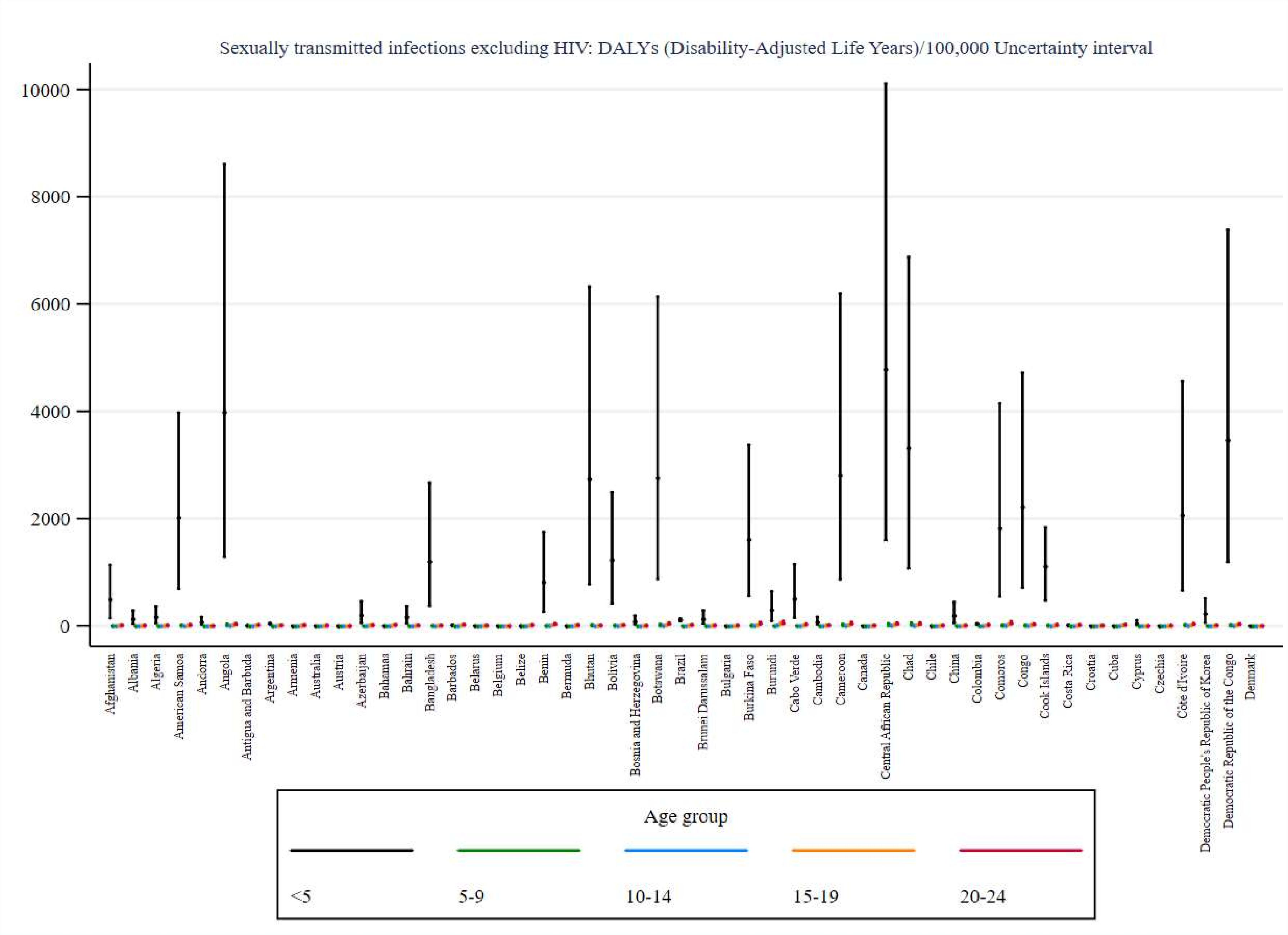

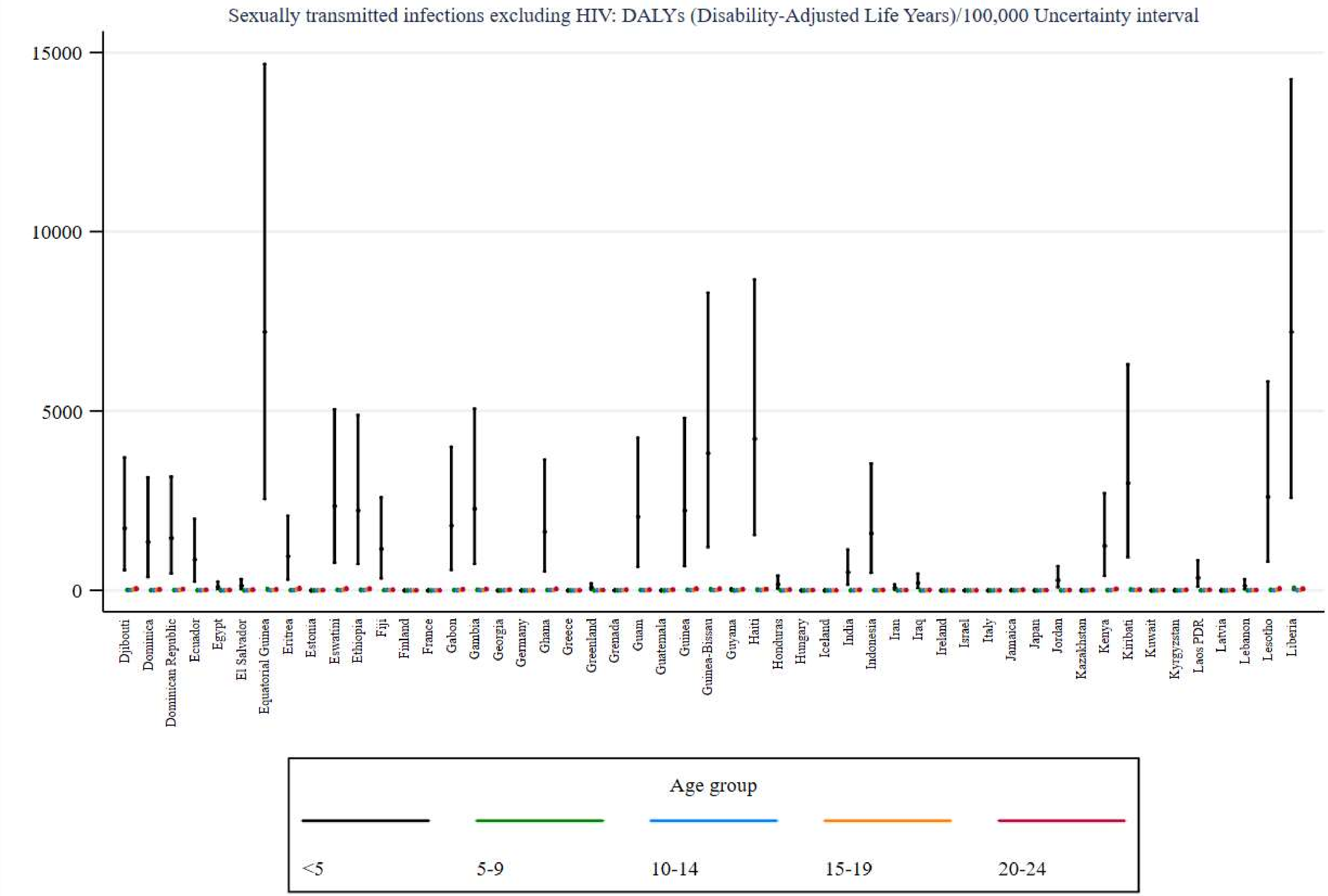

Sexually transmitted infections excluding HIV: DALYs (Disability-Adjusted Life Years)/100,000 Uncertainty interval
15000
10000
5000
0
Djibouti
Dominica
Dominican Republic
Ecuador
Egypt
El Salvador
Equatorial Guinea
Eritrea
Estonia
Eswatini
Ethiopia
Fiji
Finland
France
Gabon
Gambia
Georgia
Germany
Ghana
Greece
Greenland
Grenada
Guam
Guatemala
Guinea
Guinea-Bissau
Guyana
Haiti
Honduras
Hungary
Iceland
India
Indonesia
Iran
Iraq
Ireland
Israel
Italy
Jamaica
Japan
Jordan
Kazakhstan
Kenya
Kiribati
Kuwait
Kyrgyzstan
Laos PDR
Latvia
Lebanon
Lesotho
Liberia
Age group
<5
5-9
10-14
15-19
20-24

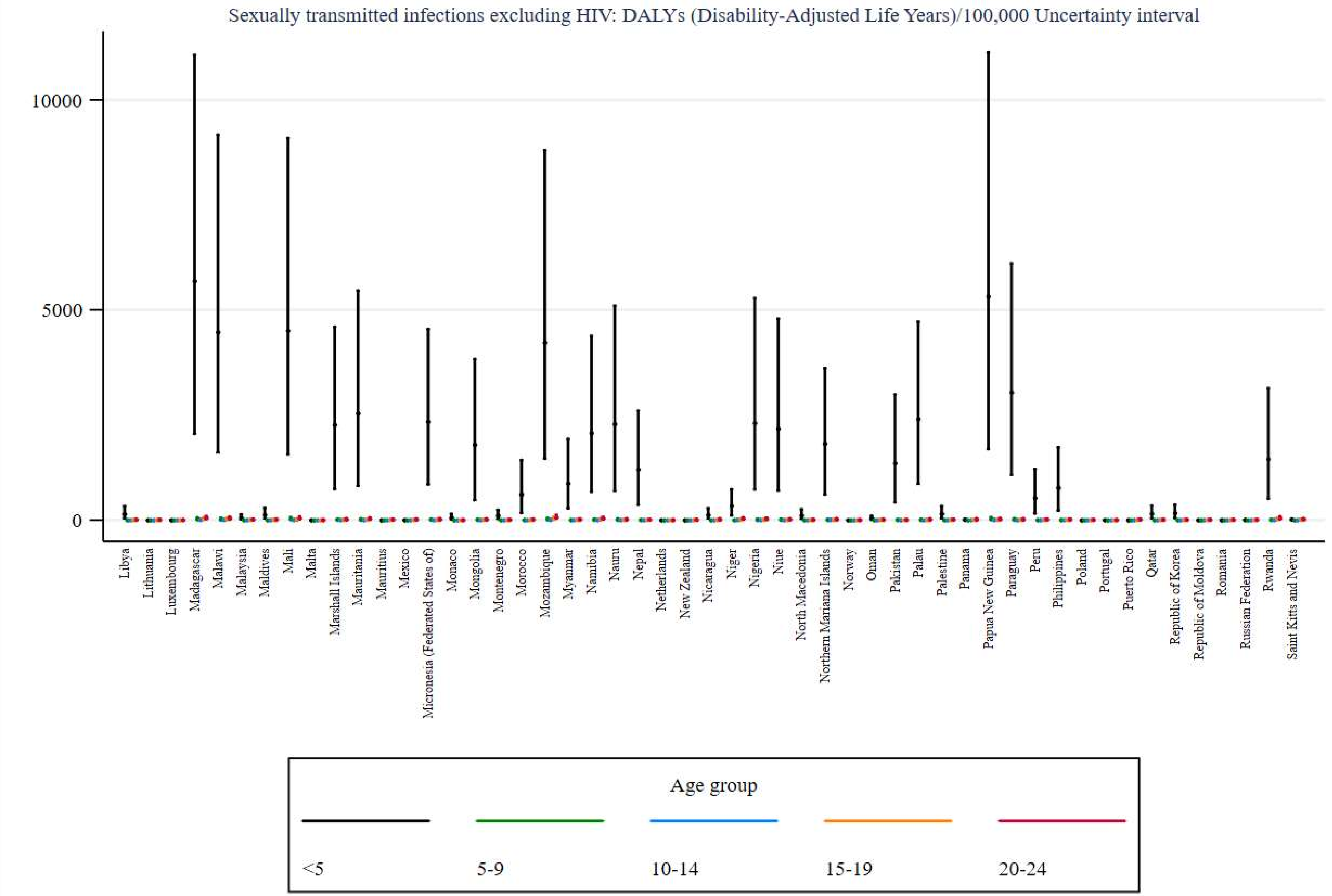

Sexually transmitted infections excluding HIV: DALYs (Disability-Adjusted Life Years)/100,000 Uncertainty interval
10000
5000
0
Libya
Lithuania
Luxembourg
Madagascar
Malawi
Malaysia
Maldives
Mali
Malta
Marshall Islands
Mauritania
Mauritius
Mexico
Micronesia (Federated States of)
Monaco
Mongolia
Montenegro
Morocco
Mozambique
Myanmar
Namibia
Nauru
Nepal
Netherlands
New Zealand
Nicaragua
Niger
Nigeria
Niue
North Macedonia
Northern Mariana Islands
Norway
Oman
Pakistan
Palau
Palestine
Panama
Papua New Guinea
Paraguay
Peru
Philippines
Poland
Portugal
Puerto Rico
Qatar
Republic of Korea
Republic of Moldova
Romania
Russian Federation
Rwanda
Saint Kitts and Nevis
Age group
<5
5-9
10-14
15-19
20-24

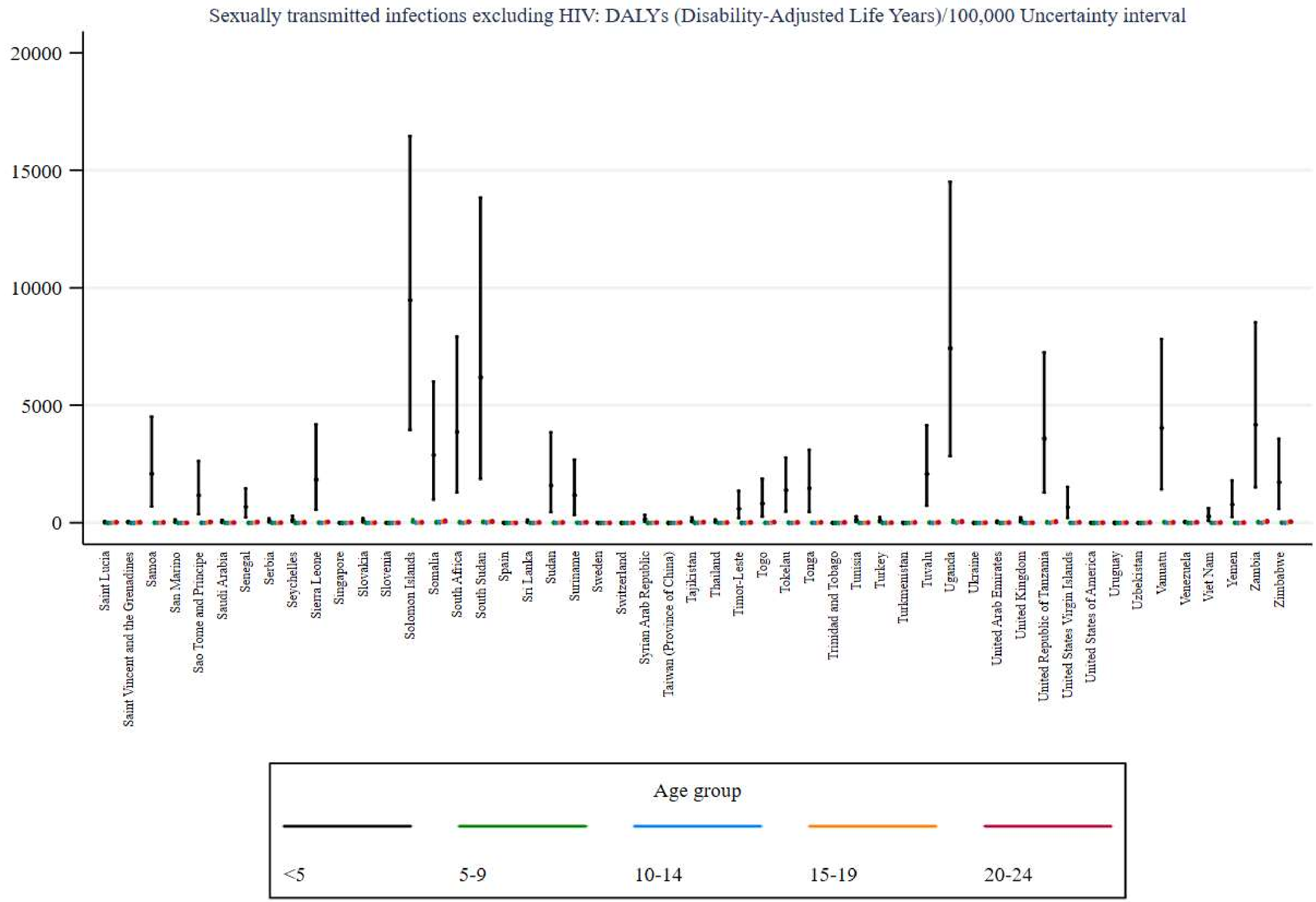
Sexually transmitted infections excluding HIV: DALYs (Disability-Adjusted Life Years)/100,000 Uncertainty interval
20000
15000
10000
5000
0
Saint Lucia
Saint Vincent and the Grenadines
Samoa
San Marino
Sao Tome and Principe
Saudi Arabia
Senegal
Serbia
Seychelles
Sierra Leone
Singapore
Slovakia
Slovenia
Solomon Islands
Somalia
South Africa
South Sudan
Spain
Sri Lanka
Sudan
Suriname
Sweden
Switzerland
Syrian Arab Republic
Taiwan (Province of China)
Tajikistan
Thailand
Timor-Leste
Togo
Tokelau
Tonga
Trinidad and Tobago
Tunisia
Turkey
Turkmenistan
Tuvalu
Uganda
Ukraine
United Arab Emirates
United Kingdom
United Republic of Tanzania
United States Virgin Islands
United States of America
Uruguay
Uzbekistan
Vanuatu
Venezuela
Viet Nam
Yemen
Zambia
Zimbabwe
Age group
<5
5-9
10-14
15-19
20-24

S20_10 Part A: Tuberculosis Deaths/ 100 000 Uncertainty interval for each age group

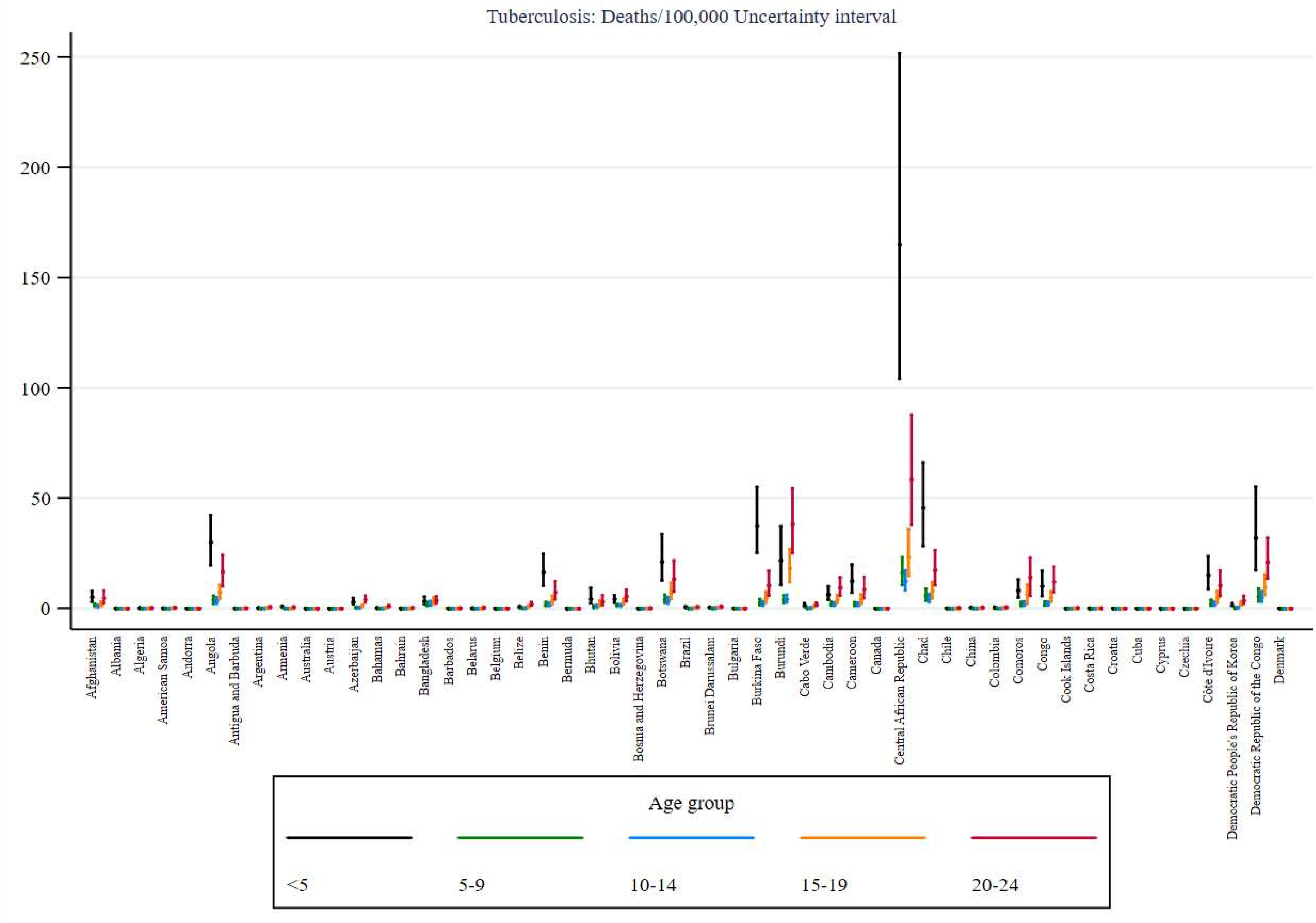

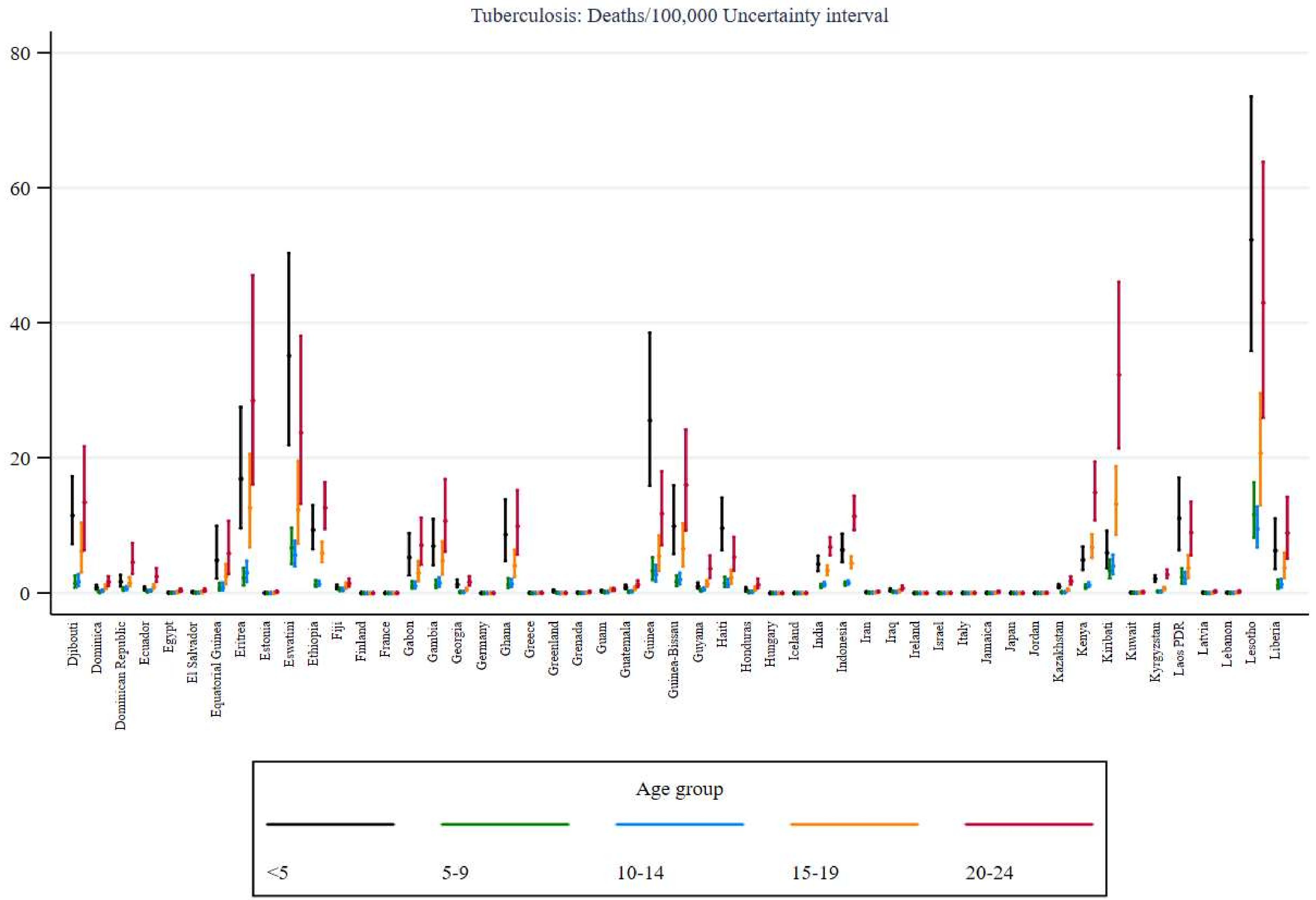

Tuberculosis: Deaths/100,000 Uncertainty interval
80
60
40
20
0
Djibouti
Dominica
Dominican Republic
Ecuador
Egypt
El Salvador
Equatorial Guinea
Eritrea
Estonia
Eswatini
Ethiopia
Fiji
Finland
France
Gabon
Gambia
Georgia
Germany
Ghana
Greece
Greenland
Grenada
Guam
Guatemala
Guinea
Guinea-Bissau
Guyana
Haiti
Honduras
Hungary
Iceland
India
Indonesia
Iran
Iraq
Ireland
Israel
Italy
Jamaica
Japan
Jordan
Kazakhstan
Kenya
Kiribati
Kuwait
Kyrgyzstan
Laos PDR
Latvia
Lebanon
Lesotho
Liberia
Age group
<5
5-9
10-14
15-19
20-24

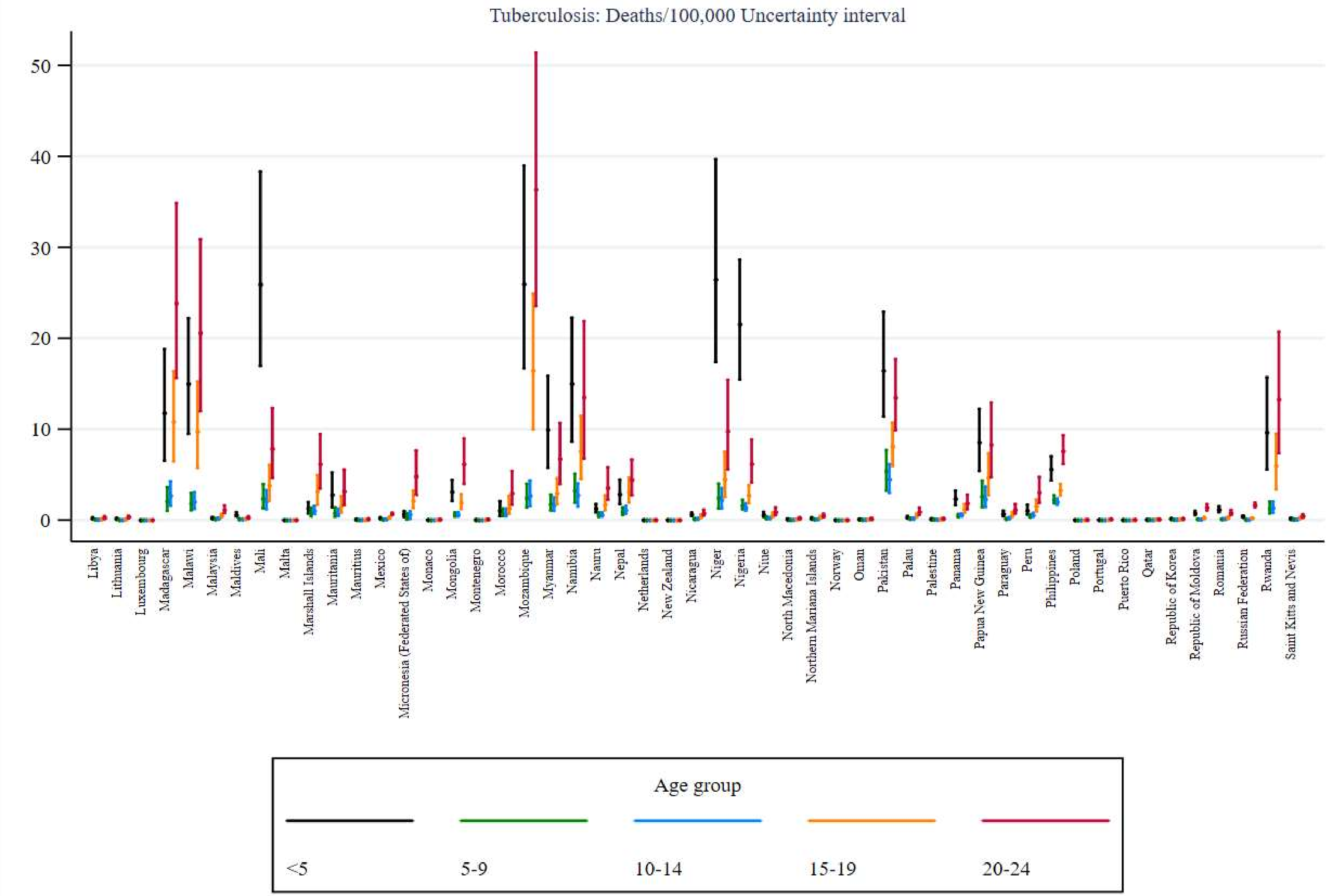

Tuberculosis: Deaths/100,000 Uncertainty interval
50
40
30
20
10
0
Libya
Lithuania
Luxembourg
Madagascar
Malawi
Malaysia
Maldives
Mali
Malta
Marshall Islands
Mauritania
Mauritius
Mexico
Micronesia (Federated States of)
Monaco
Mongolia
Montenegro
Morocco
Mozambique
Myanmar
Namibia
Nauru
Nepal
Netherlands
New Zealand
Nicaragua
Niger
Nigeria
Niue
North Macedonia
Northern Mariana Islands
Norway
Oman
Pakistan
Palau
Palestine
Panama
Papua New Guinea
Paraguay
Peru
Philippines
Poland
Portugal
Puerto Rico
Qatar
Republic of Korea
Republic of Moldova
Romania
Russian Federation
Rwanda
Saint Kitts and Nevis
Age group
<5
5-9
10-14
15-19
20-24

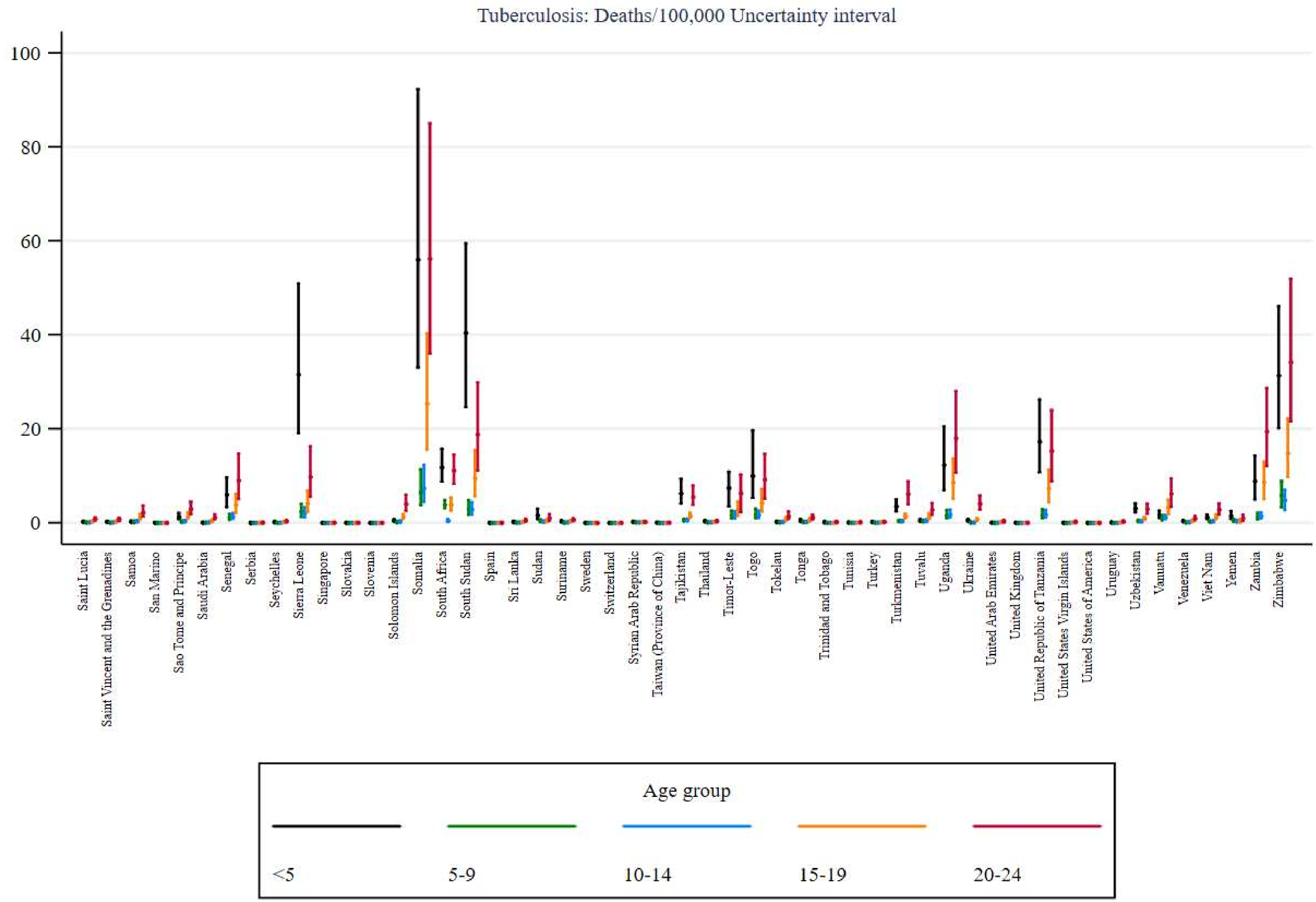
Tuberculosis: Deaths/100,000 Uncertainty interval
0
20
40
60
80
100
Saint Lucia
Saint Vincent and the Grenadines
Samoa
San Marino
Sao Tome and Principe
Saudi Arabia
Senegal
Serbia
Seychelles
Sierra Leone
Singapore
Slovakia
Slovenia
Solomon Islands
Somalia
South Africa
South Sudan
Spain
Sri Lanka
Sudan
Suriname
Sweden
Switzerland
Syrian Arab Republic
Taiwan (Province of China)
Tajikistan
Thailand
Timor-Leste
Togo
Tokelau
Tonga
Trinidad and Tobago
Tunisia
Turkey
Turkmenistan
Tuvalu
Uganda
Ukraine
United Arab Emirates
United Kingdom
United Republic of Tanzania
United States Virgin Islands
United States of America
Uruguay
Uzbekistan
Vanuatu
Venezuela
Viet Nam
Yemen
Zambia
Zimbabwe
Age group
<5
5-9
10-14
15-19
20-24

S20_10 Part B: Tuberculosis DALYs/ 100 000 Uncertainty interval for each age group

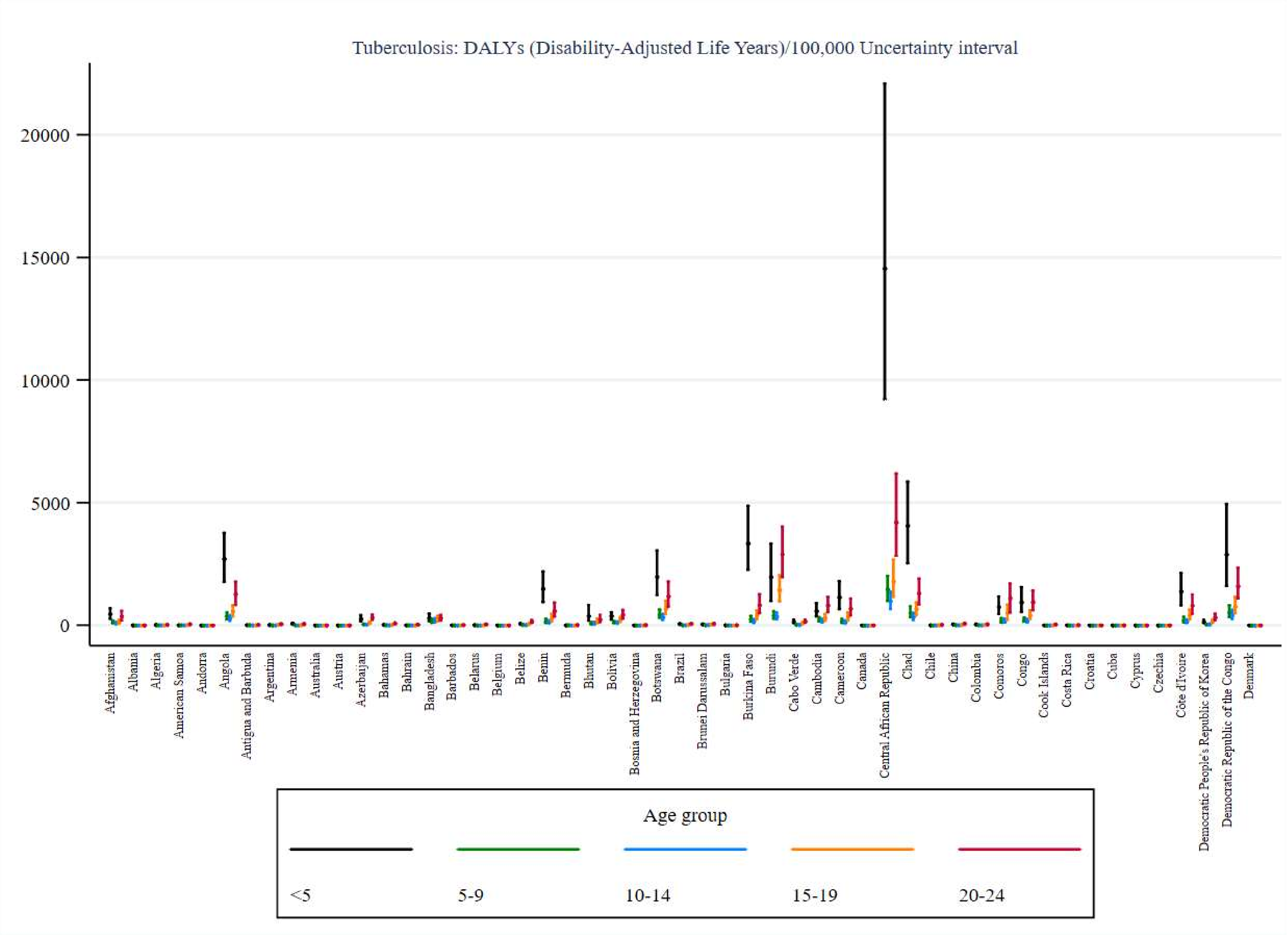

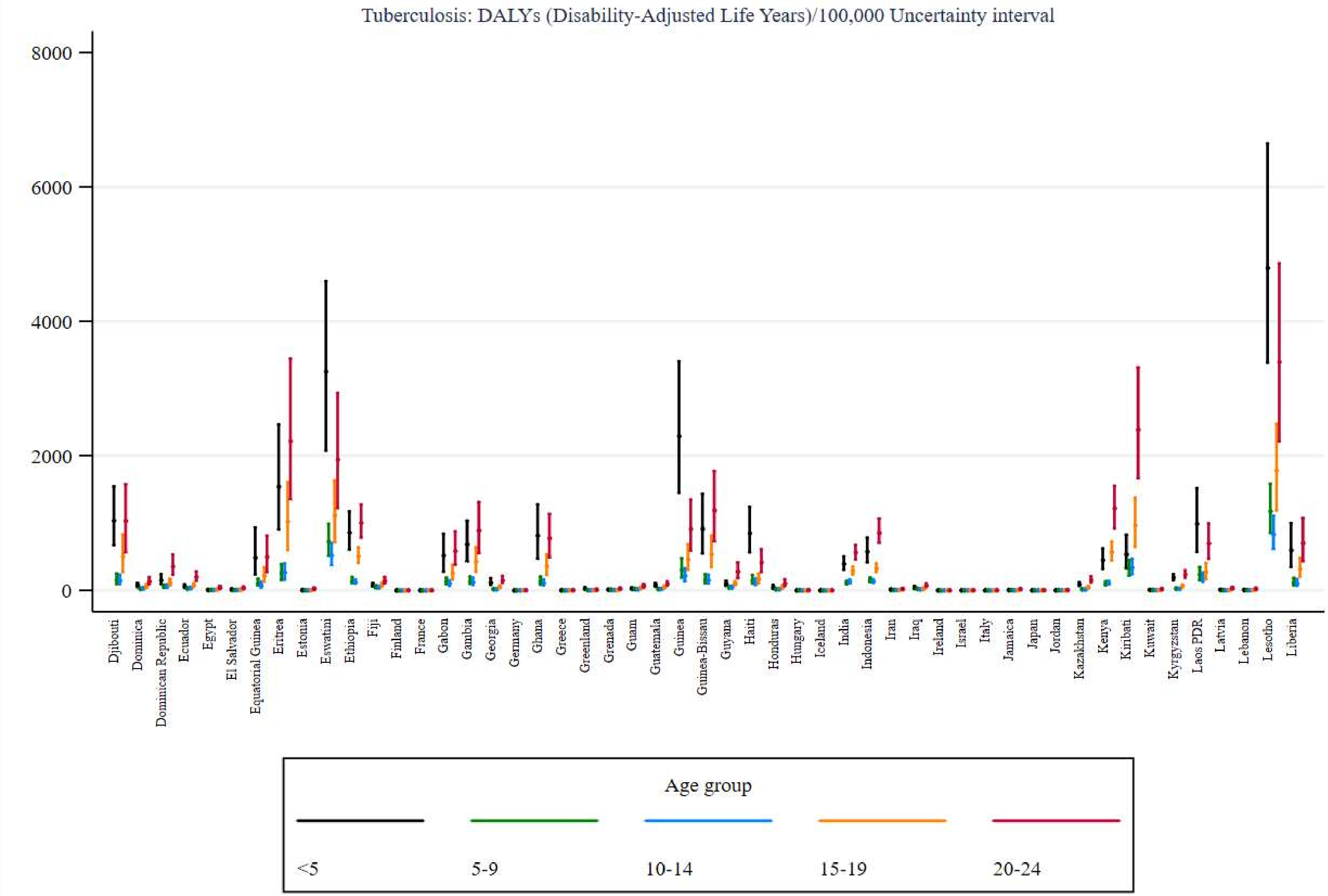

Tuberculosis: DALYs (Disability-Adjusted Life Years)/100,000 Uncertainty interval
8000
6000
4000
2000
0
Djibouti
Dominica
Dominican Republic
Ecuador
Egypt
El Salvador
Equatorial Guinea
Eritrea
Estonia
Eswatini
Ethiopia
Fiji
Finland
France
Gabon
Gambia
Georgia
Germany
Ghana
Greece
Greenland
Grenada
Guam
Guatemala
Guinea
Guinea-Bissau
Guyana
Haiti
Honduras
Hungary
Iceland
India
Indonesia
Iran
Iraq
Ireland
Israel
Italy
Jamaica
Japan
Jordan
Kazakhstan
Kenya
Kiribati
Kuwait
Kyrgyzstan
Laos PDR
Latvia
Lebanon
Lesotho
Liberia
Age group
<5
5-9
10-14
15-19
20-24

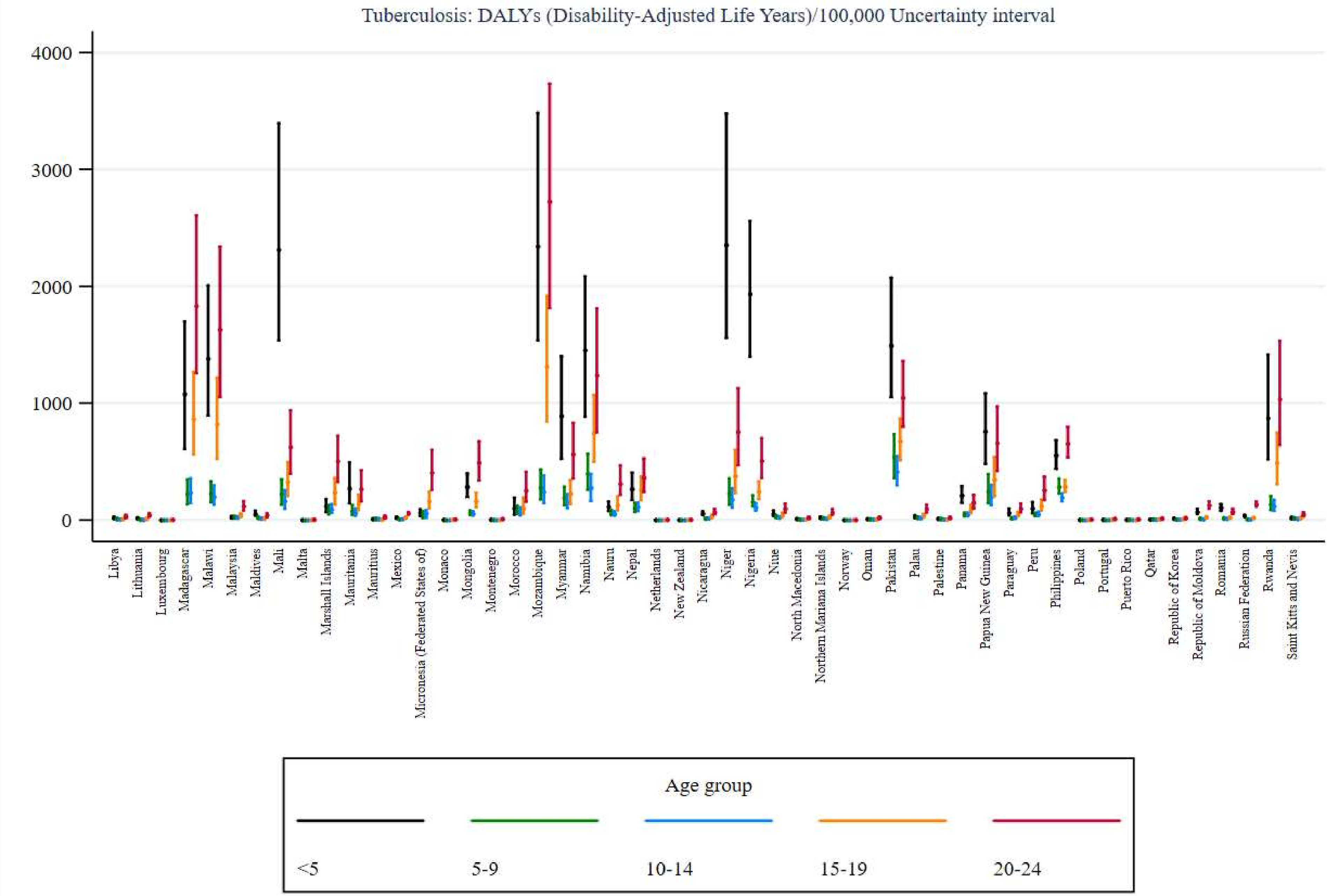
Tuberculosis: DALYs (Disability-Adjusted Life Years)/100,000 Uncertainty interval
4000
3000
2000
1000
0
Libya
Lithuania
Luxembourg
Madagascar
Malawi
Malaysia
Maldives
Mali
Malta
Marshall Islands
Mauritania
Mauritius
Mexico
Micronesia (Federated States of)
Monaco
Mongolia
Montenegro
Morocco
Mozambique
Myanmar
Namibia
Nauru
Nepal
Netherlands
New Zealand
Nicaragua
Niger
Nigeria
Niue
North Macedonia
Northern Mariana Islands
Norway
Oman
Pakistan
Palau
Palestine
Panama
Papua New Guinea
Paraguay
Peru
Philippines
Poland
Portugal
Puerto Rico
Qatar
Republic of Korea
Republic of Moldova
Romania
Russian Federation
Rwanda
Saint Kitts and Nevis
Age group
<5
5-9
10-14
15-19
20-24

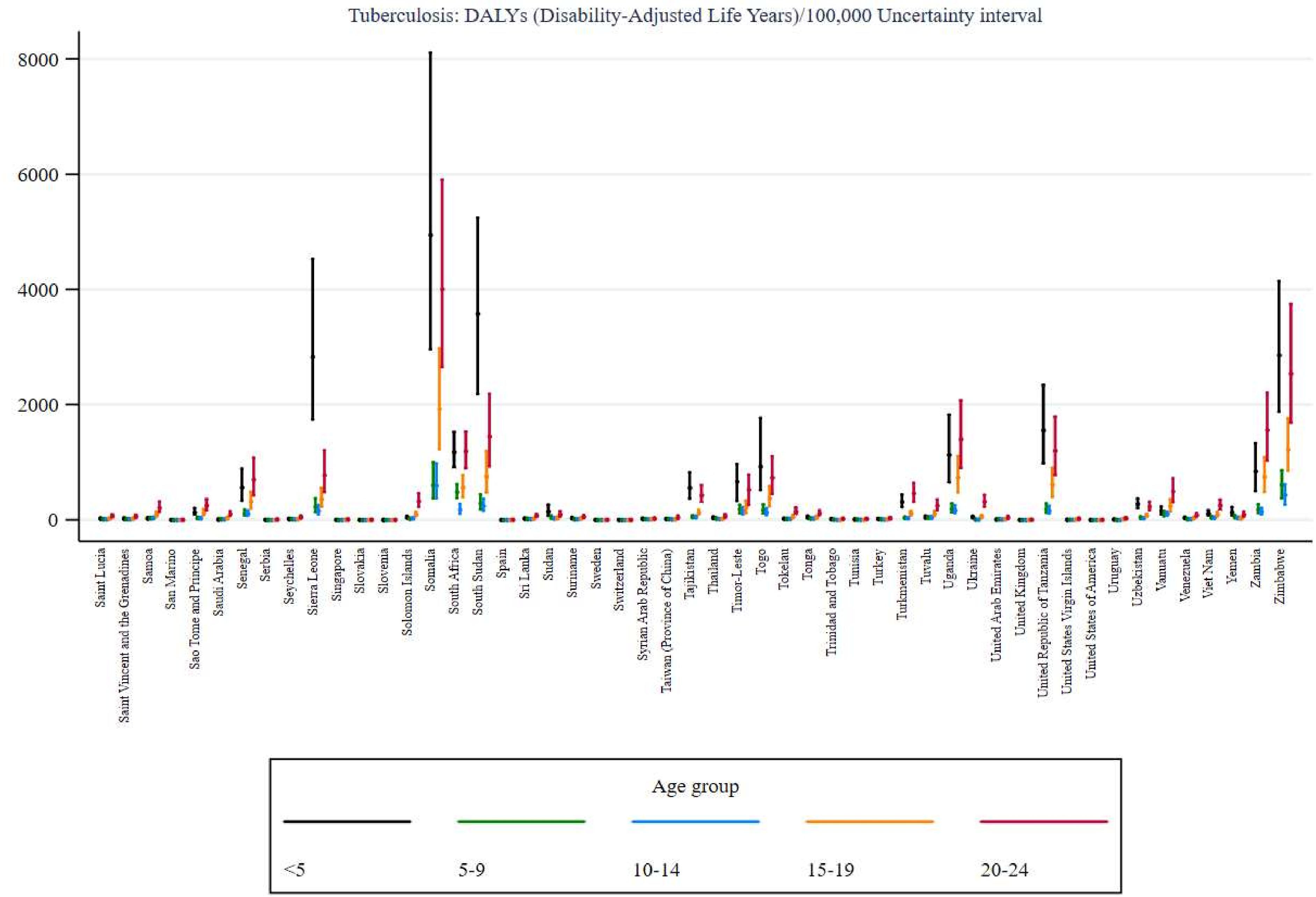

Tuberculosis: DALYs (Disability-Adjusted Life Years)/100,000 Uncertainty interval
8000
6000
4000
2000
0
Saint Lucia
Saint Vincent and the Grenadines
Samoa
San Marino
Sao Tome and Principe
Saudi Arabia
Senegal
Serbia
Seychelles
Sierra Leone
Singapore
Slovakia
Slovenia
Solomon Islands
Somalia
South Africa
South Sudan
Spain
Sri Lanka
Sudan
Suriname
Sweden
Switzerland
Syrian Arab Republic
Taiwan (Province of China)
Tajikistan
Thailand
Timor-Leste
Togo
Tokelau
Tonga
Trinidad and Tobago
Tunisia
Turkey
Turkmenistan
Tuvalu
Uganda
Ukraine
United Arab Emirates
United Kingdom
United Republic of Tanzania
United States Virgin Islands
United States of America
Uruguay
Uzbekistan
Vanuatu
Venezuela
Viet Nam
Yemen
Zambia
Zimbabwe
Age group
<5
5-9
10-14
15-19
20-24

S21: Author contributions

Writing the first draft of the manuscript

Peter S Azzopardi, Jessica A Kerr, Kate Louise Francis, Susan M Sawyer, Elissa Clare Kennedy, Andrew C Steer, Stephen Michael Graham, Russell M Viner, Joseph L Ward, Julie Hennegan, Minh D Pham, Christine Marie D Habito, Jaameeta Kurji, Karly I Cini, James G Beeson, Alex Brown, Christopher J L Murray, Simon I Hay, and George C Patton.

Providing data or critical feedback on data sources

Peter S Azzopardi, Kate Louise Francis, Russell M Viner, Joseph L Ward, Karly I Cini, James G Beeson, Christopher J L Murray, Hassan Abolhassani, Victor Adekanmbi, Muktar Beshir Ahmed, Tareq Mohammed Ali AL-Ahdal, Amir Anoushiravani, Davood Anvari, Jalal Arabloo, Seyyed Shamsadin Athari, Sara Bagherieh, Sandra Barteit, Sanjay Basu, Melaku Ashagrie Belete, Endeshaw Chekol Abebe, Natália Cruz-Martins, Xiaochen Dai, Lalit Dandona, Rakhi Dandona, Andreas K Demetriades, Alemayehu Anley Demlash, Mostafa Dianatinasab, Saeid Doaei, Fariba Dorostkar, Firooz Esmaeilzadeh, Ali Fatehizadeh, Getahun Fetensa, Santosh Gaihre, Lemma Getacher, Pouya Goleij, Mohamad Golitaleb, Vijai Kumar Gupta, Hadi Hassankhani, Demisu Zenbaba Heyi, Gaetano Isola, Jalil Jaafari, Jost B Jonas, Jacek Jerzy Jozwiak, Mikk Jürisson, Zubair Kabir, Rohollah Kalhor, Harkiran Kaur, Gbenga A Kayode, Mohammad Khammarnia, Moien AB Khan, Khaled Khatab, G Anil Kumar, Hmwe Hmwe Kyu, Stephen S Lim, Razzagh Mahmoudi, Fariborz Mansour-Ghanaei, Fereshteh Mehri, Awoke Misganaw, Abdollah Mohammadian-Hafshejani, Shafiu Mohammed, Ali H Mokdad, Lorenzo Monasta, Maryam Moradi, Ulrich Otto Mueller, Francesk Mulita, Getaneh Baye B Mulu, Ganesh R Naik, Henok Biresaw Netsere, Bogdan Oancea, Ayodipupo Sikiru Oguntade, Adrian Otoiu, Anamika Pandey, Shahina Pardhan, Romil R Parikh, Jay Patel, Prince Peprah, Maarten J Postma, Fakher Rahim, Vafa Rahimi-Movaghar, Salman Rawaf, Luca Ronfani, Siamak Sabour, Yashendra Sethi, Bogdan Socea, Mohammad Sadegh Soltani-Zangbar, Rafael Tabarés-Seisdedos, Razieh Tavakoli Oliaee, Amir Tiyuri, Marcos Roberto Tovani-Palone, Abdul Rohim Tualeka, Jef Van den Eynde, Tommi Juhani Vasankari, Theo Vos, Ronny Westerman, Sanni Yaya, Iman Zare, Mohammad Zoladl, and Alimuddin Zumla.

Developing methods or computational machinery

Peter S Azzopardi, Kate Louise Francis, Christopher J L Murray, Muktar Beshir Ahmed, Mostafa Akbarzadeh-Khiavi, Tareq Mohammed Ali AL-Ahdal, Shohreh Alian Samakkhah, Davood Anvari, Xiaochen Dai, Mostafa Dianatinasab, Saeid Doaei, Firooz Esmaeilzadeh, Ali Fatehizadeh, Mohammad Heidari, Mohammad Khammarnia, Razzagh Mahmoudi, Ali H Mokdad, Francesk Mulita, Koushik Roy Pramanik, Yashendra Sethi, Razieh Tavakoli Oliaee, Fei-Long Wei, and Ronny Westerman.

Providing critical feedback on methods or results

Peter S Azzopardi, Jessica A Kerr, Kate Louise Francis, Susan M Sawyer, Elissa Clare Kennedy, Andrew C Steer, Stephen Michael Graham, Russell M Viner, Joseph L Ward, Julie Hennegan, Minh D Pham, Christine Marie D Habito, Jaameeta Kurji, Karly I Cini, James G Beeson, Alex Brown, Christopher J L Murray, Simon I Hay, George C Patton, Hassan Abolhassani, Victor Adekanmbi, Suneth Buddhika Agampodi, Muktar Beshir Ahmed, Hossein Akbarialiabad, Mostafa Akbarzadeh-Khiavi, Tareq Mohammed Ali AL-Ahdal, Musa Mohammed Ali, Yousef Alimohamadi, Vahid Alipour, Adel Al-Jumaily, Sohrab Amiri, Amir Anoushiravani, Davood Anvari, Jalal Arabloo, Morteza Arab-Zozani, Mesay Arkew, Benedetta Armocida, Ali A Asadi-Pooya, Zatollah Asemi, Seyyed Shamsadin Athari, Hiva Azami, Mohammadreza Azangou-Khyavy, Hosein Azizi, Nader Bagheri, Sara Bagherieh, Sandra Barteit, Sanjay Basu, Melaku Ashagrie Belete, Alemshet Yirga Berhie,

Ali Bijani, Boris Bikbov, Katrin Burkart, Periklis Charalampous, Endeshaw Chekol Abebe, Natália Cruz-Martins, Xiaochen Dai, Lalit Dandona, Rakhi Dandona, Sayih Mehari Degualem, Andreas K Demetriades, Alemayehu Anley Demlash, Abebaw Alemayehu Desta, Mostafa Dianatinasab, Saeid Doaei, Fariba Dorostkar, Diyan Ermawan Effendi, Amir Emami, Luchuo Engelbert Bain, Firooz Esmaeilzadeh, Ali Faramarzi, Ali Fatehizadeh, Pietro Ferrara, Getahun Fetensa, Florian Fischer, Luisa S Flor, Masoud Foroutan, Santosh Gaihre, Nasrin Galehdar, Rupesh K Gautam, Mesfin Gebrehiwot, Teferi Gebru Gebremeskel, Motuma Erena Getachew, Mohammad Ghasemi Nour, Mohamad Golitaleb, Vijai Kumar Gupta, Hadi Hassankhani, Demisu Zenbaba Heyi, Gaetano Isola, Jalil Jaafari, Fatemeh Javanmardi, Jost B Jonas, Jacek Jerzy Jozwiak, Mikk Jürisson, Ali Kabir, Zubair Kabir, Laleh R Kalankesh, Rohollah Kalhor, Joonas H Kauppila, Harkiran Kaur, Gbenga A Kayode, Leila Keikavoosi-Arani, Mohammad Khammarnia, Moien AB Khan, Khaled Khatab, Hamid Reza Khayat Kashani, Ali-Asghar Kolahi, Hamid Reza Koohestani, Ai Koyanagi, G Anil Kumar, Om P Kurmi, Hmwe Hmwe Kyu, Carlo La Vecchia, Tea Lallukka, Stephen S Lim, Joana A Loureiro, Razzagh Mahmoudi, Azeem Majeed, Elaheh Malakan Rad, Fariborz Mansour-Ghanaei, Abdoljalal Marjani, Alexander G Mathioudakis, Alexios-Fotios A Mentis, Tomislav Mestrovic, Andreea Mirica, Awoke Misganaw, Abdollah Mohammadian-Hafshejani, Shafiu Mohammed, Ali H Mokdad, Peyman Mokhtarzadehazar, Maryam Moradi, Maliheh Moradzadeh, Negar Morovatdar, Ulrich Otto Mueller, Francesk Mulita, Getaneh Baye B Mulu, Saravanan Muthupandian, Ganesh R Naik, Abdulqadir J J Nashwan, Seyed Aria Nejadghaderi, Henok Biresaw Netsere, Maryam Noori, Bogdan Oancea, Ayodipupo Sikiru Oguntade, Hassan Okati-Aliabad, Adrian Otoiu, Anamika Pandey, Shahina Pardhan, Romil R Parikh, Jay Patel, Prince Peprah, Maarten J Postma, Fakher Rahim, Vafa Rahimi-Movaghar, Shayan Rahmani, Vahid Rahmanian, Salman Rawaf, Iman Razeghian-Jahromi, Misganu Teshoma Regasa, Mohsen Rezaeian, Abanoub Riad, Esperanza Romero-Rodríguez, Koushik Roy Pramanik, Siamak Sabour, Saeid Sadeghian, Mohammad Reza Saeb, Azam Safary, Biniyam Sahiledengle, Sara Samadzadeh, Arash Sarveazad, Yashendra Sethi, Saeed Shahabi, Mehran Shams-Beyranvand, Kiomars Sharafi, Nigussie Tadesse Sharew, Aziz Sheikh, Rahim Ali Sheikhi, Rahman Shiri, Bogdan Socea, Mohammad Sadegh Soltani-Zangbar, Rafael Tabarés-Seisdedos, Shima Tabatabai, Moslem Taheri Soodejani, Razieh Tavakoli Oliaee, Amir Tiyuri, Marcos Roberto Tovani-Palone, Jef Van den Eynde, Theo Vos, Mandaras Tariku Walde, Yanzhong Wang, Fei-Long Wei, Ronny Westerman, Vikas Yadav, Sanni Yaya, Bin Zhu, Mohammad Zoladl, and Alimuddin Zumla.

Drafting the work or revising is critically for important intellectual content

Peter S Azzopardi, Jessica A Kerr, Kate Louise Francis, Susan M Sawyer, Elissa Clare Kennedy, Andrew C Steer, Stephen Michael Graham, Russell M Viner, Joseph L Ward, Julie Hennegan, Minh D Pham, Christine Marie D Habito, Jaameeta Kurji, Karly I Cini, James G Beeson, Alex Brown, Christopher J L Murray, Simon I Hay, George C Patton, Mohsen Abbasi-Kangevari, Hassan Abolhassani, Victor Adekanmbi, Suneth Buddhika Agampodi, Muktar Beshir Ahmed, Marjan Ajami, Hossein Akbarialiabad, Tareq Mohammed Ali AL-Ahdal, Musa Mohammed Ali, Shohreh Alian Samakkhah, Sohrab Amiri, Mohammad Hosein Amirzade-Iranaq, Jalal Arabloo, Morteza Arab-Zozani, Mesay Arkew, Benedetta Armocida, Ali A Asadi-Pooya, Saeed Asgary, Seyyed Shamsadin Athari, Hiva Azami, Mohammadreza Azangou-Khyavy, Nader Bagheri, Sara Bagherieh, Francesco Barone-Adesi, Sanjay Basu, Melaku Ashagrie Belete, Luis Belo, Boris Bikbov, Giulia Carreras, Natália Cruz-Martins, Andreas K Demetriades, Mostafa Dianatinasab, Saeid Doaei, Firooz Esmaeilzadeh, Ali Faramarzi, Ali Fatehizadeh, Pietro Ferrara, Florian Fischer, Ali Forouhari, Masoud Foroutan, Santosh Gaihre, Nasrin Galehdar, Silvano Gallus, Teferi Gebru Gebremeskel, Lemma Getacher, Motuma Erena Getachew, Seyyed-Hadi Ghamari, Mohammad

Ghasemi Nour, Mohamad Golitaleb, Giuseppe Gorini, Maryam Hashemian, Mohammad Heidari, Demisu Zenbaba Heyi, Gaetano Isola, Jost B Jonas, Jacek Jerzy Jozwiak, Mikk Jürisson, Ali Kabir, Laleh R Kalankesh, Joonas H Kauppila, Gbenga A Kayode, Mohammad Khammarnia, Moien AB Khan, Khaled Khatab, Ai Koyanagi, Om P Kurmi, Carlo La Vecchia, Tea Lallukka, Joana A Loureiro, Soleiman Mahjoub, Razzagh Mahmoudi, Azeem Majeed, Elaheh Malakan Rad, Afshin Maleki, Abdoljalal Marjani, Alexander G Mathioudakis, Alexios-Fotios A Mentis, Tomislav Mestrovic, Awoke Misganaw, Abdollah Mohammadian-Hafshejani, Hussen Mohammed, Shafiu Mohammed, Ali H Mokdad, Peyman Mokhtarzadehazar, Lorenzo Monasta, Maryam Moradi, Maliheh Moradzadeh, Negar Morovatdar, Ulrich Otto Mueller, Getaneh Baye B Mulu, Seyed Aria Nejadghaderi, Nurulamin M Noor, Bogdan Oancea, Adrian Otoiu, Alicia Padron-Monedero, Reza Pakzad, Shahina Pardhan, Romil R Parikh, Jay Patel, Umberto Pensato, Norberto Perico, Dimitri Poddighe, Maarten J Postma, Fakher Rahim, Vafa Rahimi-Movaghar, Shayan Rahmani, Vahid Rahmanian, Salman Rawaf, Iman Razeghian-Jahromi, Misganu Teshoma Regasa, Giuseppe Remuzzi, Abanoub Riad, Esperanza Romero-Rodríguez, Luca Ronfani, Koushik Roy Pramanik, Siamak Sabour, Amirhossein Sahebkar, Biniyam Sahiledengle, Sara Samadzadeh, Yashendra Sethi, Saeed Shahabi, Fariba Shahraki-Sanavi, Mehran Shams-Beyranvand, Bogdan Socea, Shima Tabatabai, Razieh Tavakoli Oliaee, Marcos Roberto Tovani-Palone, Rohollah Valizadeh, Jef Van den Eynde, Tommi Juhani Vasankari, Yanzhong Wang, Fei-Long Wei, Ronny Westerman, Vikas Yadav, Iman Zare, Bin Zhu, Mohammad Zoladl, and Alimuddin Zumla.

Managing the estimation or publications process

Peter S Azzopardi, Kate Louise Francis, Susan M Sawyer, Christine Marie D Habito, Karly I Cini, Christopher J L Murray, Simon I Hay, George C Patton, Saeid Doaei, Firooz Esmaeilzadeh, Ali Fatehizadeh, Gaetano Isola, Razzagh Mahmoudi, Ali H Mokdad, and Razieh Tavakoli Oliaee.
